# Multiple mechanisms drive genomic adaptation to extreme O_2_ levels in *Drosophila melanogaster*

**DOI:** 10.1038/s41467-021-21281-6

**Published:** 2021-02-12

**Authors:** Arya Iranmehr, Tsering Stobdan, Dan Zhou, Huiwen Zhao, Sergey Kryazhimskiy, Vineet Bafna, Gabriel G. Haddad

**Affiliations:** 1 Department of Electrical & Computer Engineering, University of California, San Diego, La Jolla, CA USA; 2 Division of Respiratory Medicine, Department of Pediatrics, University of California, San Diego, La Jolla, CA USA; 3 Division of Biological Sciences, University of California, San Diego, La Jolla, CA USA; 4 Department of Computer Science & Engineering, University of California, San Diego, La Jolla, CA USA; 5 Department of Neurosciences, University of California, San Diego, La Jolla, CA USA; 6 Rady Children’s Hospital, San Diego, CA USA

**Keywords:** Experimental evolution, Evolutionary biology

## Abstract

To detect the genomic mechanisms underlying evolutionary dynamics of adaptation in sexually reproducing organisms, we analyze multigenerational whole genome sequences of *Drosophila melanogaster* adapting to extreme O_2_ conditions over an experiment conducted for nearly two decades. We develop methods to analyze time-series genomics data and predict adaptive mechanisms. Here, we report a remarkable level of synchronicity in both hard and soft selective sweeps in replicate populations as well as the arrival of favorable de novo mutations that constitute a few asynchronized sweeps. We additionally make direct experimental observations of rare recombination events that combine multiple alleles on to a single, better-adapted haplotype. Based on the analyses of the genes in genomic intervals, we provide a deeper insight into the mechanisms of genome adaptation that allow complex organisms to survive harsh environments.

## Abstract (web summary)

The genomic details of adaptation to extreme environments remain challenging to characterize. Using new methods to analyze flies experimentally evolved to survive extreme O_2_ conditions, the authors find a surprising level of synchronicity in selective sweeps, de novo mutations and adaptive recombination events.

## Introduction

Evolution under natural selection is manifested by the fact that, in each generation, individuals carrying mutations favored by the environmental niche are more likely to survive and reproduce. The mechanisms of adaptation under strong selection pressure, however, are subject to some debate. For instance, adaptation could be mediated by extant (and originally drifting) polymorphisms or cryptic genetic variation^1^, or by de novo mutations, all yielding a fitness advantage in the challenging environments. For sexually reproducing organisms, multiple favored variants can also be acquired on a single haplotype via recombination that prevents clonal interference and accelerates adaptation^2,3^. Because of the difficulties of directly observing evolution in action, there is a huge gap in our understanding of how, and even if, these mechanisms are co-opted by adapting populations.

There has been much debate about the benefits of sexual reproduction despite its obvious costs^4^. Fisher and Muller proposed that sex could accelerate adaptation by bringing beneficial alleles that arose in different genetic backgrounds onto the same haplotype, i.e., reduce what later became known as “clonal interference”^5–7^, Hill and Robertson (1966) used two locus computer simulations to confirm this prediction^8,9^. Recombination can also help purge deleterious mutations that may otherwise accumulate in asexual populations due to stochastic or deterministic reasons^3,10–13^.

Multiple experiments on yeast by Gray and Goddard and colleagues have empirically demonstrated that sex increases the efficacy of natural selection, unlinks beneficial from deleterious mutations and allows yeast to adapt to specific evolutionary niches^14–16^. McDonald et al. found that sex alters the spectrum of mutations that are fixed in yeast and reduces clonal interference to speed up adaptation^2^. More recently, Leu and colleagues studied the dynamics of adaptation in sexual and asexual yeast populations subjected to extreme temperature over 1400 generations^17^. They found that both sexual and asexual adaptation occurred at similar rates, but showed significant differences between the two. Notably, these previous studies did not directly investigate the molecular mechanisms used for specific selective sweeps. Addressing that question would require time-series sequencing of intermediate generations, and recent efforts aim to do exactly that to better elucidate the mechanisms underlying selection even for complex organisms with longer generation times^18–21^.

In this work, we detect the genomic mechanisms underlying evolutionary dynamics of adaptation in a sexually reproducing organism. We first generate multiple *Drosophila melanogaster* populations adapting to extreme O_2_ conditions through laboratory evolution. We then perform whole-genome sequencing at multiple generations and develop methods to determine the adaptive mechanisms by analyzing these time-series genomic data. We find a remarkable level of synchronicity in both hard and soft selective sweeps in replicate populations as well as the arrival of favorable de novo mutations that constitute a few asynchronized sweeps. Additionally, we obtain direct experimental evidence of rare recombination events combining multiple alleles on to single, better-adapted haplotype. Bioinformatic mining of the genes located in the evolving genomic intervals provide a deeper insight into the mechanisms that allow complex organisms to survive harsh O_2_ environments, including glutamate receptor activity, Notch signaling, PI3K activity, Rho guanyl-nucleotide exchange factor activity as well as VEGF signaling.

## Results

**Fig. 1:**
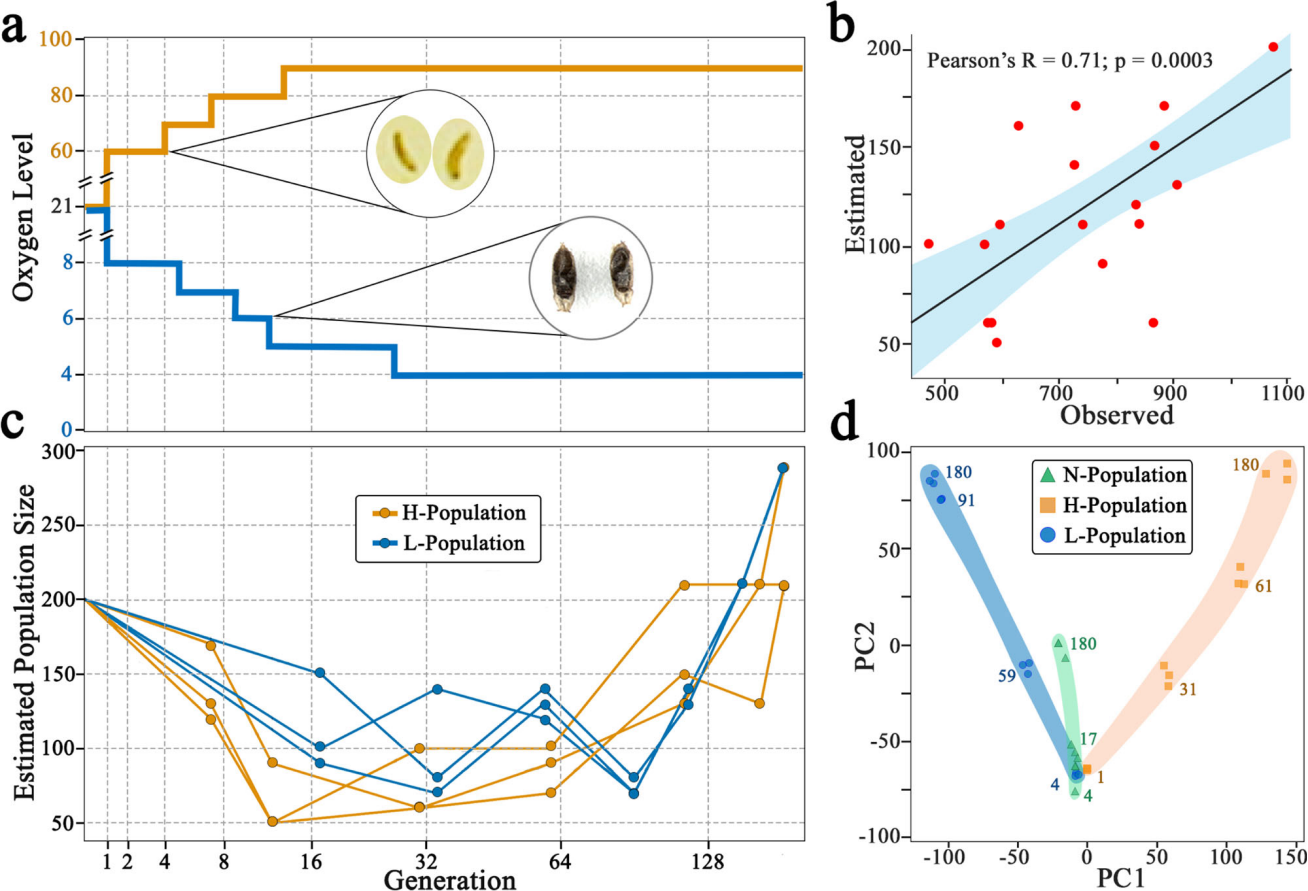
Strong environmental selection pressure leading to the various alterations in the L- and H-population. **a** Plot depicting oxygen level and the generations for L-population (blue line) and H-populations (orange line) (Source data are provided as Fig. 1a Source Data), **b** Estimated vs observed population size and error bands correspond to 95% confidence interval for the regression coefficient (Pearson’s R) (Source data are provided as Fig. 1b Source Data), **c** Estimated population size at different generations under selection pressure of L and H environments (Source data are provided as Fig. 1c Source Data), and **d** Principal Component Analysis (PCA) depicting PC1 and PC2 for the three replicates of L-, H-, and N-populations explains 45% of the variance (Supplementary Fig. 1a). The PCA was performed using only extant single nucleotide polymorphisms (SNPs) (Source data are provided as Fig. 1d Source Data). L-population, the population evolving in hypoxic environments; H-population, the population evolving in hyperoxic environments; N-population, the population maintained in normoxic environment.

We conducted an experiment (>290 generations) over >18 years to determine the effect of selection pressure on their genomes through a change in environmental O_2_. We were motivated in part by the remarkable and recent adaptation of humans who have maintained O_2_ homeostasis and have survived over hundreds of generations, while facing very low O_2_ environments in multiple high-altitude locations^22^. We performed the experiment by chronically exposing multiple fly populations to decreasing or increasing O_2_ levels using a pool of 27 isogenic founder lines as the parental population. Nine offspring populations (the F1 generation), containing similar numbers of embryos (2000–3000 embryos), were collected and allowed to evolve independently in the culture chambers supplied with gradually decreasing O_2_ levels (L-populations, *n* = 3) or increasing O_2_ levels (H-populations, *n* = 3). And three populations were maintained under normal O_2_^23–25^. In order to determine the starting O_2_ concentration to initiate the low or high O_2_-directed evolution, we tested the reproductive feasibility and tolerance of the parental lines to low or high O_2_ environments. For the low O_2_ environment, we tested culture conditions with O_2_ concentrations ≤8%. We found that the eclosion rate was dramatically reduced to ~5% under 5% O_2_; and 4% O_2_ environment was lethal. For the high O_2_ environment, we tested culture conditions with O_2_ concentrations ≥60%. We discovered that 80% or greater O_2_ level was lethal. Hence, we initiated the low O_2_-directed laboratory evolution at 8% O_2_, and the high O_2_-directed evolution at 60% O_2_ (Fig. 1a). The concentration of O_2_ was decreased or increased every 3-5 generations (or until the population size was in a steady state) to keep the selection pressure on the *Drosophila* population (Fig. 1a). As the experiments progressed, we observed bottlenecks with severe reduction of population size in both L- and H-populations with every change, i.e., 1% O_2_ drop and 10% increase respectively, in O_2_ level (Fig. 1b and c). The sharp reduction in population size gradually recovered in subsequent generations (Fig. 1b and c). It is important to note that low or high O_2_ selection happened at different developmental stages: low O_2_ induced lethality at the pupal stage, whereas high O_2_ triggered death of 1st and 2nd instar larvae (Fig. 1a, insert), suggesting that different genetic and molecular mechanisms are evoked to regulate adaptation to L- or H- O_2_ environments. We then took advantage of fly generational time-series samples and performed whole-genome sequencing (WGS) analysis of three L-fly populations (at generations 4, 17, 34, 59, 91, 117, 149, 180) and three H-fly populations (at generations 1, 7, 12, 31, 61, 114, 162, 180) with balanced pool of samples representing each population replicates (*n* = 200). A WGS analysis of N-populations, considered as controls, was performed at generations 4, 17, and 180^26^.

We used a Wright-Fisher Markov-Chain-based method on pooled WGS data to estimate the effective population size directly from changes in allele frequencies (Supplementary Methods)^27,28^. The results were highly concordant with a manual census (Fig. 1b, Pearson’s R = 0.71; *p*-value = 0.0003), demonstrating the reliability of the computational estimates. When applied to the time-series data, the estimates suggested a significant population bottleneck in all 3 L-populations and 3 H-populations, followed by recovery as the populations adapted (Fig. 1c). The bottleneck in the L-populations was most severe when the O_2_ level was reduced to 5% at the 13th generation and 4% at the 32nd generation. The bottleneck in the H-populations was most severe when the level of O_2_ was increased to 90% at the 13th generation. In both cases, the recovery was gradual, occurring over 100 generations thereafter.

A principal component analysis (PCA) using only extant allele frequencies was performed to examine the temporal evolution of the populations. We found that the populations were well separated by the top two principal components (Fig. 1d), explaining 45% of the total variance (Supplementary Fig. 1a). As the PCA was performed using only extant single nucleotide polymorphisms (SNPs), the increasing divergence from the starting populations in each of H-, L-, and N-populations over 180 generations in PC2 could be attributed to genetic drift. In contrast, the separation along PC1 corresponded largely to environmental changes (i.e., the level of O_2_ in the environments), with L- and H- populations diverged in opposite directions, while the N-population remained relatively unchanged suggesting a genome-wide impact of selection. Notably, the physically isolated population replicates were clustered remarkably tightly at each generation along evolution in either H-, L-, or N-environment resulting in three clear trajectories of evolution for each of the three different O_2_ conditions. The results demonstrated that, in each environmental condition, the impact of the selection pressure on genomes was similar in the isolated populations that arose from the same founder populations. Genetic divergence due to de novo mutations likely occurred in localized regions of the genome, and did not significantly change population structure. To test this, we repeated the PCA analysis using all (de novo and extant) SNPs and did not see any changes to the PCA clusters (Supplementary Fig. 1b).

Strong selection on the populations is likely to induce selective sweeps of mutations, in localized regions that are favored in the hypoxic or hyperoxic environments with hitchhiking mutations linked to them, causing a rapid change in frequency upon onset of selection until fixation. Post-fixation, the populations should drift again while maintaining the favored mutations. Consistent with this hypothesis, the divergence in the first 60 generations of the H-population during adaptation exceeded the divergence in the next 120 generations by 1.49-fold. Similarly, the divergence in the first 90 generations of the L-populations was 2.71-fold the divergence in the next 90 generations (Supplementary Fig. 2).

**Fig. 2:**
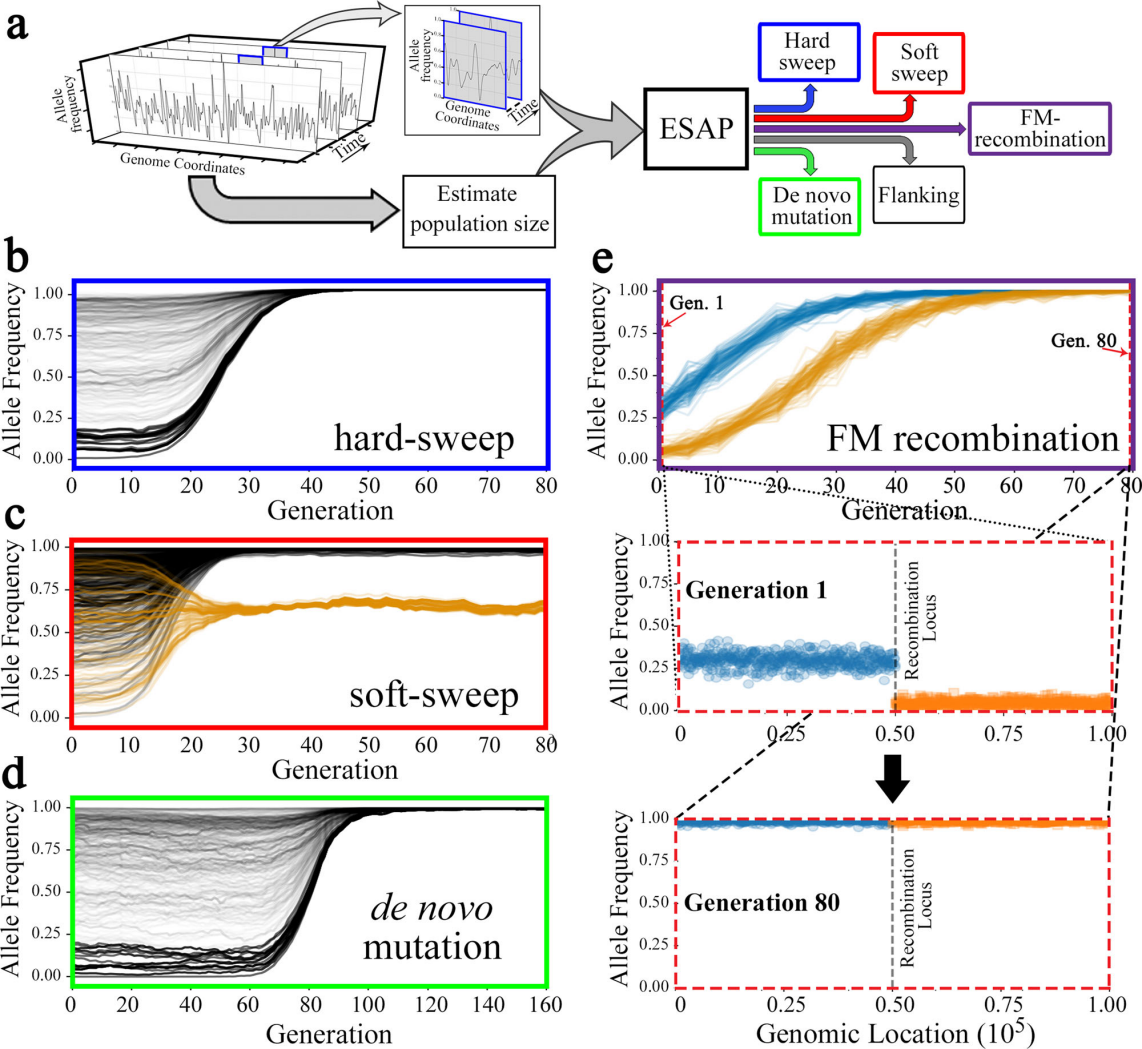
Deciphering the underlying mechanisms of selection using the Experimental Evolution Selection Analysis Pipeline (ESAP). **a** ESAP takes a time-series interval of pooled frequencies from a genomic region and predicts the mechanism of the selection sweeps based on allele-frequency trajectories. **b** The characteristic allele-frequency trajectory for a hard-sweep simulation, when the favored mutation lies on a homogeneous background. The trajectories of all linked mutations on that haplotype converge to fixation. **c** The characteristic allele-frequency trajectory in simulations of a soft sweep due to standing variation. The favored mutation itself is fixed along with tightly linked mutations (black lines). However, the favored mutation is carried by multiple haplotypes, which drift at intermediate frequencies (orange lines). **d** Simulated instance of favored de novo mutation in a ‘late’ sweep. **e** Simulation of an ‘FM recombination’ that combines beneficial mutations onto a single haplotype. Starting at generation 80 from a cluster (haplotype) of fixed alleles, and tracing back in time, ESAP identifies two distinct clusters/haplotypes colored blue and orange (top panel). The blue and orange circles in the middle and lower panels provide the allele frequencies and genomic location of the mutations in these haplotypes at generation 1 (middle panel), and generation 80 (lower panel). Note the perfect separation of the two flanking haplotypes around ChrX:7,750,000 i.e., the recombination locus.

To identify genomic loci involved in the adaptation using pooled WGS time-series data, we used a previously described ‘Composition of Likelihood for Evolve and Resequencing Experiment’ (CLEAR) statistic^28^. CLEAR relies on the statistical separation^29^ between the trajectory of the mutation (and linked hitchhikers) favored by the selective sweep versus the trajectory of drifting mutations (Supplementary Fig. 3). As the effective population sizes in our populations were small (Fig. 1c), and genetic drift in small populations (effective population size, Ne < 200) could easily lead to large fluctuation in allele frequencies over large time intervals (Supplementary Fig. 4), we applied the CLEAR method in shorter time intervals ranging from 30 to 120 generations (Fig. 2a, Supplementary Fig. 5).

While the CLEAR method was sufficient to identify genomic loci under selection, it was silent on the underlying mechanism of selection. To identify the mechanisms, we developed an Experimental Evolution Selection Analysis Pipeline (ESAP) (Fig. 2a, Supplementary Fig. 5). ESAP starts with the genomic loci identified by CLEAR as undergoing selective sweeps in each time interval. We first considered cases of replicated sweeps where the selective sweep was observed in a genomic region in all three replicates due to extant (standing) variation, and subsequently cases of individual sweeps when the sweep (likely due to de novo events) was not observed in the three replicates.

Replicated sweeps can occur due to multiple mechanisms. When the favored mutation in an early sweep is carried on a homogeneous background (single haplotype), the trajectories of all linked mutations on that haplotype converge to fixation in a ‘hard’ sweep (Fig. 2b). However, when the favored mutation is present on more than one (carrier) haplotype, mutations common to all carrier haplotypes undergo a hard sweep, while mutations on specific carrier haplotypes converge to an intermediate frequency and drift (Fig. 2c), providing a signature for a soft sweep with standing variation. ESAP classified replicated sweeps as hard and soft with standing variation using a chi-square test (Supplementary Methods).

Individual sweeps appearing early could be attributed to extant variation, which was favored by selection but failed to establish in some replicates. However, sweeps occurring many generations after the onset of selection in individual replicates are unlikely to occur on extant variation. Using simulations (Supplementary Methods), we computed a *p*-value for observing extant mutations going into selective sweeps with selection pressure ‘s’, at least ‘*t’* generations after onset of selection. ESAP classified the sweep as late if p(t,s) < 10^−3^, and early otherwise. The occurrence of a nonreplicated late sweep suggested either a de novo favored mutation (Fig. 2d) or a recombination event that created a highly beneficial haplotype by either combined multiple favored mutations or off-loaded some deleterious mutations. We refer to the latter cause as the Fisher–Muller recombination event (or ‘FM recombination’ for short). To distinguish between these two origins of a late individual sweep, ESAP traced the fixed variants back in time to identify distinct clusters of mutations M and M’ along the genome that formed a single haplotype at fixation. If the mutations originated from clusters that were also spatially segregated along the genome (Fig. 2e), then the recombination ESAP classified such sweep as an instance of ‘FM recombination’. Otherwise, the sweep was attributed to de novo mutation.

**Fig. 3:**
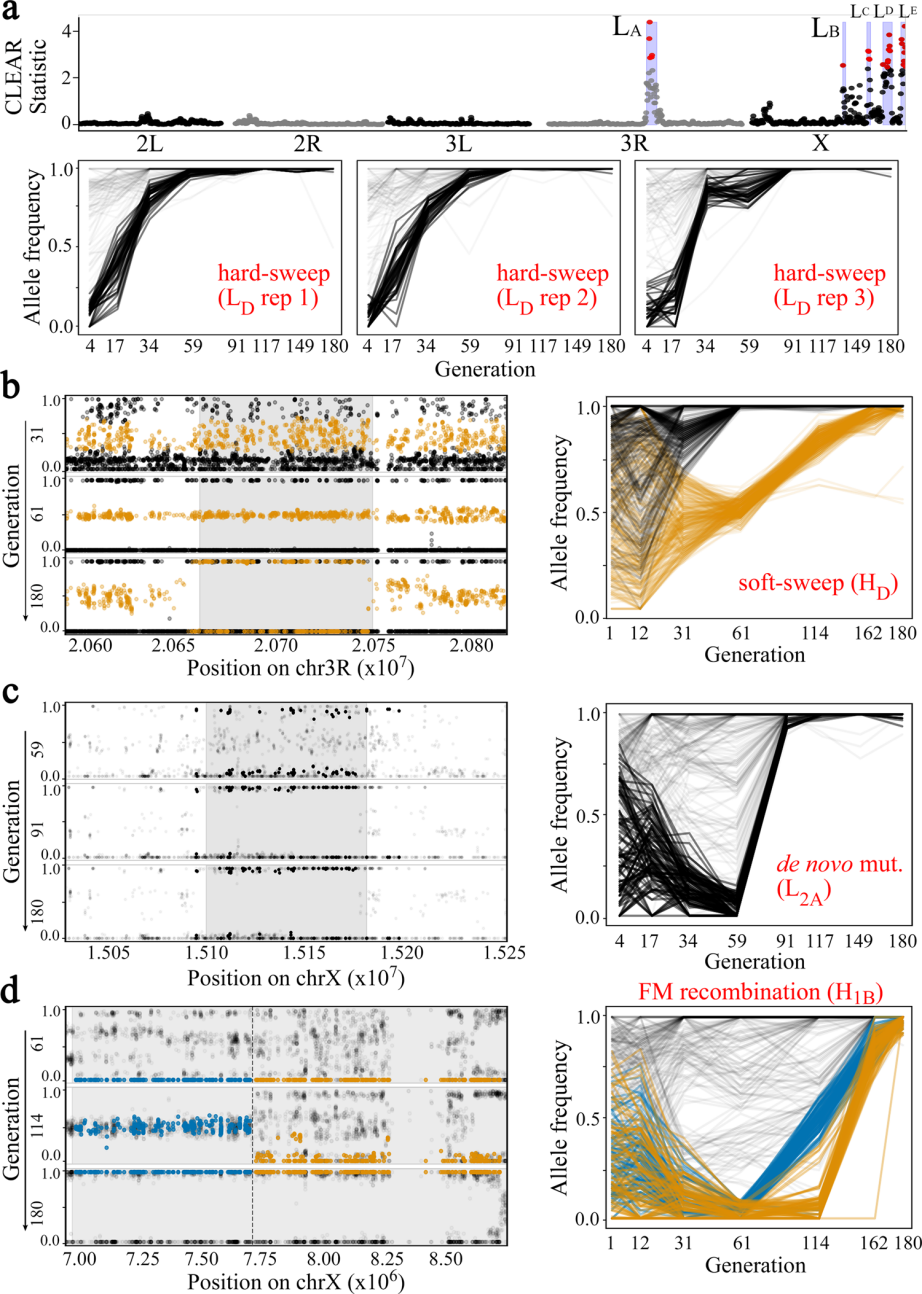
Mechanisms of genetic adaptation utilized by *Drosophila melanogaster* in extreme O_2_ environments. **a** Manhattan plot showing five replicated sweeps in the L-population. Allele-frequency trajectories of an early hard sweep (region L_D_) in the three replicates of populations evolving under low O_2_. The *x*-axis is on a logarithmic scale and shows fixation by generation 34. **b** A replicated soft sweep due to standing variation in interval H_D_. The favored allele was fixed at generation 61 while a second, tightly linked cluster (orange color) remains at intermediate frequency. **c** A de novo mutation in interval L_2A_ in one replicate of the L-population. **d** An FM-recombination in interval H_1B_ in one replicate of the H-population. Alleles that are fixated in generation 180 form two distinct clusters at generation 114 (orange and blue colors, respectively) that are spatially separated on chromosome X (position ChrX:7,750,000).

Applying ESAP to the fly populations, we identified five intervals in the L-population (labeled L_A_ through L_E_, see Fig. 3a, Table 1 and Supplementary Fig. 6) and four intervals in the H-population (labeled H_A_ through H_D_, see Table 1 and Supplementary Fig. 7) with a selective sweep in all three replicate populations.

**Table 1:** Selected intervals that have frequency distribution synchronized in all three biological replicates (replicated sweeps).

Name	Coordinate (fixed)	Length	Selection	Generations	Kind	Coordinate (adjusted)
L_A_	chr3R:15.11–16.47 Mb	1360 Kb	Low oxygen	59–91	Hard sweep	chr3R:15.12–16.48 Mb
L_B_	chrX:13.01–13.36 Mb	355 Kb	Low oxygen	59–91	Hard sweep	chrX:13.03–13.07 Mb
L_C_	chrX:16.12–16.57 Mb	445 Kb	Low oxygen	4–34	Hard sweep	chrX:16.27–16.48 Mb
L_D_	chrX:18.16–19.35 Mb	1190 Kb	Low oxygen	4–34	Hard sweep	chrX:18.17–19.23 Mb
L_E_	chrX:20.49–21.20 Mb	710 Kb	Low oxygen	59–91	Soft sweep	chrX:20.52–21.13 Mb
H_A_	chrX:4.51–4.76 Mb	248 Kb	High oxygen	1–31	Hard sweep	chrX:4.52–4.73 Mb
H_B_	chrX:16.57–16.95 Mb	380 Kb	High oxygen	12–61	Hard sweep	chrX:16.67–17.18 Mb
H_C_	chr2L:10.35–10.62 Mb	273 Kb	High oxygen	31–114	Hard sweep	chr2L:10.42–10.93 Mb
H_D_	chr3R:20.66–20.75 Mb	90 Kb	High oxygen	12–61	Soft sweep	chr3R:20.68–20.73 Mb

All intervals picked by the adjusted size regime (Coordinate (adjusted)) corresponding to intervals selected by the fixed size regime (Coordinate (fixed)).

Before analyzing these results further, we tested for any confounding factors that could result in a false signal. First, we determined if the use of fixed population size with CLEAR was appropriate for scenarios with varying and small population sizes. To check, we used a version of CLEAR that uses estimated population sizes (Supplementary Methods), and found complete concordance between CLEAR signals with fixed population and adjusted population sizes (Table 1, Supplementary Figs. 6–8). Next, we investigated whether background selection as purifying selection can distort the allele-frequency spectrum (AFS). Of note, the number of SNPs with *i* mutant alleles is inversely proportional to *i* for neutrally evolving populations^30^, suggesting hyperbolically decreasing intermediate allele frequencies, which disappear or diminish under positive selection. Therefore, we investigated the AFS at generation 180 in every selected region of the L and H-populations and compared it to the AFS in the N-population at the same locus. Expectedly, we observed intermediate frequency alleles in the N-populations but their complete absence in the corresponding H- and L- populations in every selected region (Supplementary Fig. 9), confirming that the signal in L and H-populations was not due to background selection.

Remarkably, and supporting the notion that the signal is due to selection pressure, we found that the allele-frequency trajectories were completely time-synchronized across all three replicates in each of the intervals L_A_–L_E_ and H_A_–H_D_ (Fig. 3a, Supplementary Figs. 10–18). Expectedly, these sweeps on standing variation started early for the most part. However, three sweeps, all in the L-population, started close to generation 60 but were still synchronized across replicates (Fig. 3a, Supplementary Figs. 10, 11, and 14). These results suggest that a change in the low-O_2_ environment favored extant mutations.

The most significant interval in the H-populations, H_A_ (Supplementary Fig. 15), was indicative of a hard sweep involving the rapid and synchronous fixation of 987 mutations in three populations and elimination of 1196 mutations (Supplementary Fig. 15). In contrast, interval H_D_ was identified as a replicated soft-sweep signal in which 914 extant SNPs were fixed while 449 extant SNPs that were present on different haplotypes remained polymorphic at intermediate frequencies (Z-statistic *p*-value < 6.91E-177; Fig. 3b).

ESAP-analysis also identified six late sweeps that were seen in only one replicate (T-statistic *p*-value < 1E-4), and likely involved de novo events (Fig. 3c, Table 2, and Supplementary Fig. 19–29). Remarkably, one of the late sweeps (H_1B_) showed the characteristic signature of FM-recombination (Fig. 3d, Supplementary Fig. 30). Specifically, consider all alleles in the fixed haplotype in generations 162–180. Tracing back in time to generation 114, the alleles split into two distinct haplotypes, with frequencies 0.2 (orange) and 0.5 (blue), respectively. In contrast to a de novo mutation where the variants in the two haplotypes would be distributed throughout the region, we observed that the two haplotypes were well separated on either side of ChrX:7,750,000 (Fig. 3d and Supplementary Fig. 30; *p*-value = 2.4E-32, Y statistic). To our knowledge, this is the first direct observation of an FM-recombination event in a multicellular species.

**Table 2:** Selected intervals where the selected intervals appear in one of the three biological replicates (individual sweeps).

Name	Coordinate	Length	Selection	Generations	Kind	Origin
L_1,A_	chrX:14.51–14.75 Mb	240 Kb	Low oxygen	59–91	Hard sweep	De novo mutation
L_2,A_	chrX:15.10–15.18 Mb	75 Kb	Low oxygen	59–91	Hard sweep	De novo mutation
H_1,A_	chr3R:4.47–5.08 Mb	610 Kb	High oxygen	12–61	Hard sweep	Standing or de novo variation
H_1,B_	chrX:6.95–8.65 Mb	1700 Kb	High oxygen	61–162	Hard sweep	Recombination
H_2,A_	chrX:19.25–19.98 Mb	730 Kb	High oxygen	61–114	Hard sweep	De novo mutation
H_3,A_	chr3R:24.00–25.02 Mb	1025 Kb	High oxygen	31–114	Hard sweep	De novo mutation

This experiment generated a wealth of information on genes likely to be involved in O_2_ homeostasis. However, identifying specific favored mutations and genes is difficult because only one, or a few, mutated gene(s) in each interval is likely to be favored by selection. While these candidate genes should be systematically explored in future work, we compared our initial findings against known evidence. Specifically, we observed that the interval with the strongest signal (L_A_) contains the *cic* gene, whose human ortholog, *CIC*, has been reported to be involved in Ethiopian highlander adaptation^31^. Additionally, knocking down the *cic* gene, using RNAi lines, led to a higher eclosion rate at 5% O_2_ in flies^31^. Likewise, the human ortholog of *bnl* (i.e., fibroblast growth factor (FGF) family) is reported to have a role in altitude adaptation in humans^26,32,33^ and in highland animals^34,35^ with its expression measured using Affymetrix microarray was upregulated >2-fold in flies when exposed to 5% O_2_^36^.

To test for ‘network adaptation’, involving multiple genes from the same pathway, we looked for common biological processes and molecular functions i.e., GO terms, that were shared between the five L-intervals (433 genes) and the four H-intervals (215 genes) (Supplementary Table 1). ‘ATP binding’ (GO:0005524), was shared by all nine intervals (five from L-intervals and four from H-intervals; *p*-value = 1E-13). Similarly, ‘Oxidation-reduction process’ (GO:0055114) was shared by all L-intervals (*p*-value = 1E-11). Among other examples, and genes regulating Notch signaling pathway were identified in three intervals; (*p*-value = 6.0256E-10), specifically *Dl* (*Delta*) and *H* (*Hairless*) in L_A_, *sno* (*strawberry notch*) in L_B_ and *htk* (*hat-trick*) in L_D_ (Supplementary Table 2).

**Fig. 4:**
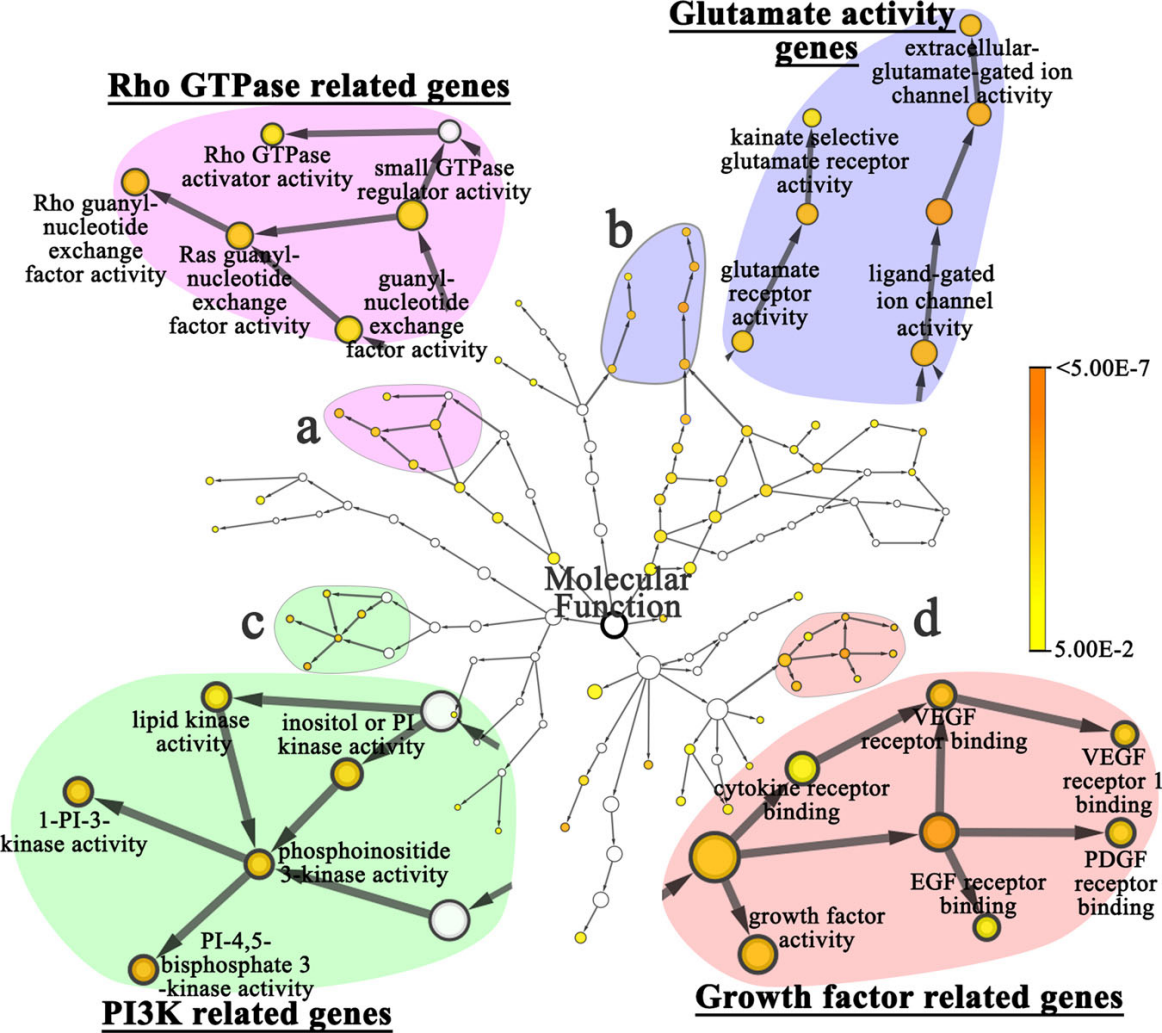
Representative molecular functions of the candidate genes depicting overrepresentation of four major signaling pathways critical for regulating hypoxia tolerance. **a** Rho guanyl-nucleotide exchange factor activity (pink area, *p*-value = 5.65E-06), **b** glutamate receptor activity (purple area, *p*-value = 2.31-06), **c** PI3K activity (green area, *p*-value = 2.24E-06) and **d** Vascular endothelial growth factor (VEGF) signaling (orange area, *p*-value = 4.45E-06). The *p*-value was calculated using Hypergeometric test and corrected using Benjamini & Hochberg False Discovery Rate (FDR). A *p*-value < 0.05 as significant level.

Investigations of highlander populations in Tibet, Ethiopia, and Andean mountains of Peru and Bolivia^22,37^, including our own^26,31,33^, have identified ~1085 genes playing a role in low O_2_ adaptation. Remarkably, we found that 80 of the 433 L-interval fly genes were orthologous to 99 human genes previously reported in human high-altitude adaptation (*p*-value = 2.8E-12). The 80 fly genes include 26 genes located in L_A_ (34 human orthologs), 12 genes in L_B_ (12 orthologs), 14 genes in L_C_ (22 orthologs), 20 genes in L_D_ (32 orthologs), and 8 genes in L_E_ (10 orthologs). The 99 human genes enrich signaling pathways critical for regulating hypoxia response or tolerance, including VEGF signaling (*p*-value = 4.45E-06), glutamate receptor activity (*p*-value = 2.31E-06), Rho guanyl-nucleotide exchange factor activity (*p*-value = 5.65E-06) as well as PI3K activity (*p*-value = 2.24E-06) (Fig. 4, Supplementary Tables 3 and 4).

We investigated individual SNPs in L_A_ and identified (Supplementary Methods: SNP prioritization) 28 SNPs that were (a) functional; (b) evolutionarily conserved with an identical reference allele in 12 *Drosophila* species^38^; and (c) showed 3-way replication of the alternate allele rising to fixation. Three of the 28 were de novo variants, i.e., variants that were absent in the initial generation. Remarkably 2 of the 3 were located in *Ire1* and *CG31213*, which participate in ATP binding (GO:0005524). The other 25 SNPs included one located in *CG17199* (GO:0055114; Redox process), one SNP in *cic* gene, previously reported in hypoxia adaptation^31^ and two SNPs in the *H* gene, a candidate gene of Notch canonical pathway in *Drosophila*^39^.

Unlike the adaptation to low O_2_ levels that has been studied in multiple species including humans^22,31,33^, adaptation to oxidant stress such as in high O_2_ has not previously been studied. In order to validate some of the candidate genes in intervals displaying a selective sweep under high O_2_ levels, we genetically manipulated 15 candidate genes located in the H_A_ interval (chrX:4465000-4775000, Supplementary Figs. 7 and 15), and tested the survival rate of flies under high O_2_ (i.e., 80% O_2_ conditions). In exactly one of 15 candidate genes tested, *CG15472*, a knockdown and loss of expression led to a significantly higher eclosion rate (Supplementary Fig. 31). The gene and its orthologs have not previously been functionally characterized.

## Discussion

In spite of a voluminous literature on cellular protection against low oxygen supply^40^ or high oxidant burden in various tissues^41,42^, there have not been major advances for therapeutic interventions to preserve cells, especially in sensitive organs, such as the heart and brain^42,43^. One discovery of the past few decades (at both organismal and molecular levels) that had a potential for therapy was the decrease in metabolism during hypoxia, a response that attempts to minimize the mismatch between O_2_ supply and demand^44–47^. This discovery, however, did not materialize into a real effective therapy, as the clinical trials of brain cooling, for example, to lower brain metabolism in patients suffering from brain hypoxia or ischemia, were largely inconclusive^48^. Another discovery that focused our efforts on understanding high-altitude adaptation is that some of the genes obtained from these studies played a substantial role in protecting mammalian organs from injury when severely deprived of oxygen^49,50^. Hence, the importance of this current experiment stems from two ideas: (a) it has spanned a period of >18 years in our laboratory using *Drosophila melanogaster* to “shrink” tens of thousands of years, the time that mammalian generations might take for adaptation, and (b) there is conservation of disease genes in *Drosophila*^51–53^ allowing us to explore the role of human orthologs in understanding adaptation and potentially developing effective therapeutic modalities.

In order to take advantage of the uniqueness of this current experiment, we developed powerful computational methods that helped reveal mechanisms of adaptation in sexually reproducing populations of multicellular organisms using pooled time-series data. Key to our methods was an exploitation of the fact that alleles nearing fixation must lie on the same haplotype. Therefore, pooled-sequencing (as opposed to individual sequencing) is sufficient to identify favored haplotypes. In addition, the availability of time-series data allowed us to trace the history of those alleles going back in time, and predict mechanisms, including hard and soft sweeps due to extant variation, arrival of de novo favored mutations, as well as FM-recombinations. For example, one of the key observations here is that we could identify intervals with remarkable consistencies of allele-frequency dynamics synchronized through time/generations between the reproductively isolated populations subjected to a specific environmental pressure. The fact that these selected intervals are present only in one type of environment (i.e., either only in L-population or in H-population) plausibly indicates its environment-specific functional significance. Remarkably, these selected intervals consisted of both standing variations and de novo mutations that go into fixation, predictably providing a base for selection. Additional examples include individual instances of de novo mutations in certain isolated populations and a rare event of an FM recombination.

The presence of late, replicated sweeps in our data present an interesting and unexpected result. Replication in the three cohorts suggest a favored mutation that was present at the onset of selection, but, surprisingly, did not confer a beneficial advantage until many generations later. We cannot rule out the possibility that changing O_2_ concentration had a potential effect and the benefit of an extant variation was realized only upon reaching that concentration or potential threshold. However, we note that O_2_ levels in our experiments were fixed after generation 15 for High O_2_, and generation 31 for Low O_2_, while the designated ‘late’ sweeps started after generation 60. Therefore, we conjecture that nonreplicated late sweeps are best explained by a de novo mutation or recombination while replicated late sweeps suggest that the benefit of an extant mutation manifested only after a previous sweep was completed.

These data collectively demonstrate that under extreme environmental selection, organisms use every available mechanism to adapt. In the critical early period right after the onset of selection, they rely largely on existing diversity and use existing mutations that provide a fitness advantage. However, in subsequent generations, they also incorporate de novo mutations and recombination to evolve genotypes that improve the fitness.

It is interesting to note that a significant number of the selected intervals (four out of five in the L-populations and two out of four in the H-populations) are located on the X chromosome, well in excess of its size, which represents 20% of the *Drosophila* genome. Indeed, previous studies have suggested that, due to its hemizygosity in males, selection acts more efficiently on X -chromosome genes than genes located on the autosomes^54–56^. We speculate that for complex adaptations, multiple genes in a pathway can play an adaptive role, and the most efficient path is chosen. Furthermore, in each of the nine genomic intervals (five in L-populations and four in H-populations), hard and soft sweeps were not only reproduced, but also time-synchronized in three replicate populations in both high and low O_2_ environments. This remarkable reproducibility of outcome suggests that any molecular determinant of adaptation that could be identified must have functional implications for survival and would reveal insights regarding O_2_ homeostasis and survival to extreme O_2_ environment. Our experiments suggest that similar methodology could be deployed in other models of experimental evolution of sexually reproducing populations.

Identification of the functional basis of the genes involved in adaptation to oxidative stress is challenging because each interval encodes a large number of genes and only one of those could be carrying the favored mutation. In addition, there is a relative paucity of studies related to oxidative stress in *Drosophila*. However, for the low O_2_ environment, we were aided first by the fact that ~1505 genes have been identified in humans as being involved in hypoxia response or adaptation, and we could identify 80 fly genes in L-intervals as being orthologous to 93 of the human genes, including *Ire1*, *CG31213* and *cic*. Moreover, the 80 fly genes were distributed across all five intervals providing us with a rich source of genes to connect with human genes involved in hypoxia tolerance. Second, these intervals were enriched in specific pathways such as VEGF signaling, glutamate receptor activity, Rho guanyl-nucleotide exchange factor activity, and PI3K activity. Of importance is that these pathways and networks of genes have been linked to hypoxia tolerance in humans^57–60^. Taken altogether, our results provide a comprehensive demonstration of how multicellular organisms adapt to harsh environments by co-opting all possible genomic mechanisms aimed at enhancing specific families of genes in order to favor a variety of biological functions and systems that work synchronously for survival.

## Methods

### Oxygen-directed experimental evolution of *Drosophila melanogaster*

A total of 27 isofemale DMN (*Drosophila menalogaster* Netherlands) lines descended from individual *Drosophila melanogaster* females caught at fruit baits at a single location in Leiden, Netherlands (52^◦^01′ N 4^◦^29′ E) during October 1999 (kindly provided by Dr. Andrew Davis) were used to create a single laboratory cage population (founding population) with 20 males and 20 virgin females from each line (1080 flies in total). As the isofemale lines used in our population were caught in the wild, considerable genetic diversity existed in the founding population. Indeed, as previously described, diverse levels of hypoxia tolerance have been found between these DMN lines^23^. These parental lines had different responses to acute anoxia challenge and different eclosion rates under chronic hypoxic conditions^23,61^. Embryos from this parental population were collected as F1 and subjected to experimental evolution in low or high oxygen (O_2_) environments (oxygen-directed evolutions, three populations per condition), or under room air (control experiment for genetic drift, three populations). This constituted nine offspring populations (the F1 generation), containing 2000–3000 embryos, that were collected and allowed to evolve independently in the culture chambers supplied with gradually decreasing O_2_ levels (L-populations, *n* = 3) or increasing O_2_ levels (H-populations, *n* = 3). And three populations were maintained under normal O_2_ (normoxic) condition as controls (N-populations, *n* = 3). The experimental evolution in the low O_2_ environment was started at 8% O_2_, and this concentration was gradually decreased by 1% each 3–5 generations to keep the selection pressure. The evolution under the high O_2_ conditions was started at 60% O_2_, and this concentration was gradually increased by 10% to maintain the selection pressure. In house designed population chambers (26 × 16 × 16 cm) were used for the experiments. These chambers were connected to either O_2_ balanced with N_2_ at certain O_2_ concentration (for the oxygen-directed evolution experiments) or to room air (21% O_2_, for the control experiments). The humidity in the chambers was maintained by passing the gas through water prior to going into the chambers. The gas was supplied to the chambers with a constant flow rate that was monitored by 565 Glass Tube Flowmeter (Concoa, Virginia Beach, VA), and the O_2_ level within the chamber was monitored with Diamond General 733 Clark Style Electrode (Diamond General Development Corp., Ann Arbor, MI). Embryos, 3rd instar larvae and adult flies were collected from each generation and stored at −80 °C for subsequent analyses. Briefly, 200–300 embryos, 100 wandering 3rd instar larvae and all adult flies (2000–3000) per population were collected at each generation. The adult samples were collected at the end of each generation after they laid eggs to start the next generation. These numbers of sampling did not apply to the bottleneck and some generations right after. The number of adult flies in a population at the end of each generation was used to estimate the physical size of the L-, H-, and N-populations.

### Whole-genome resequencing and data processing

Please see supplementary methods for details. Briefly, genomic DNA was isolated from a pool of 100 male and 100 female adult flies collected from each population at multiple generations by standard phenol:chloroform extraction followed by treatment with DNase-free RNase. DNA quality was assessed by using Bioanalyzer 2100 with DNA 1000 Assay Kit (Agilent Technologies, Santa Clara, CA), and DNA degradation or potential contamination was tested using agarose gel electrophoresis. Whole-genome sequencing (paired-end 150nt (PE150)) was performed using Illumina HiSeq X Ten Platform (Illumina, San Diego, CA).

Following quality control (QC) and read filtering, the sequences were mapped to *Drosophila melanogaster* genome (release 5.37) with BWA-MEM (version 0.7.8). GATK was used to generate gVCF files for each sample to call bases and extract reference and alternate allele counts for biallelic SNPs. Principal Component Analysis (PCA) was used to visualize the dynamics of population structure across environments and generations. An Experimental evolution Selection Analysis Pipeline (ESAP) and composition of likelihoods for evolve-and-resequence (CLEAR) software were developed^62^ and applied to estimate population size, calculate the likelihood ratio statistic for selection and time of fixation as well as to determine hard or soft sweeps and de novo mutations. Molecular interaction networks were integrated and visualized with BiNGO (Biological Network Gene Ontology) version 3.0.3 plugin on an open-source bioinformatics software platform Cytoscape 3.8.0 (https://cytoscape.org/). The GO term for ‘molecular functions’ was used to test for enrichment.

### High O_2_ tolerance test

The genes from the top interval (i.e., HA interval) were selected for validation. The RNAi fly lines for the selected candidate genes (Supplementary Table 7) were purchased from Bloomington *Drosophila* Stock Center (BDSC, Indiana University). The *da-Gal4* driver was used to ubiquitously knockdown the candidate gene in the F1 progeny. The [UAS-*RNAi*] × [*da-Gal4*] crosses were considered as experimental. The *y*^*1*^*v*^1^, *da-Gal4*, and *RNAi* were ‘self-crossed’ and used as negative controls, and the H-population flies were used as positive control. Three to 5-day-old *da-Gal4* males (*n* = 10) were crossed to female UAS-*RNAi* line (female, *n* = 10) targeting-specific gene. Sufficient time was given (3 days) for the flies to mate/cross and these are referred to as ‘cross’. Each set of crosses were in triplicate. The vials were kept under ambient conditions for 48 hour so that the flies can lay sufficient number of fertilized eggs. After 48 hour, the adults were transferred to a new vial. For the hyperoxia tolerance test and the original vials were then transferred to a computer controlled high O_2_ chamber, constantly maintained at 80% O_2_. Chambers were in the same room as ambient O_2_ controls with 12/12 hours light/dark cycle (temperature 22 °C). The adults from the new vials i.e., from the second batch of vials, were discarded after 48 hour and the vials with the fertilized eggs were kept at ambient O_2_ conditions (21% O_2_) also with 12/12 hours light/dark cycle (temperature 22 °C). These were the control vials. After 21 days, the ratio of the empty pupae (eclosed) to the total number of pupae formed (eclosed + uneclosed) in each vial was calculated to determine the eclosion rate. The differences in eclosion rate at 80% O_2_ between the *RNAi* × *da-Gal4* and all the controls were assessed using paired sample *t*-test. A *p*-value of <0.05 was considered statistically significant. Each fly crosses were performed in triplicates.

### Reporting summary

Further information on research design is available in the Nature Research Reporting Summary linked to this article.

## Supplementary information

Supplementary Information

Peer Review

Reporting Summary

## Data Availability

Whole-genome sequence data of *n* = 59 pooled samples are available at https://trace.ncbi.nlm.nih.gov/Traces/study/?acc=PRJNA657615&o=acc_s%3Aa. Source data are provided with this paper.

## Source data

Source Data

## Code availability

CLEAR software, all the scripts and Jupyter notebooks, and preprocessed dataset for reproducing the results are available at https://github.com/airanmehr/ESAP (doi: 10.5281/zenodo.4362601).
