# Supplementary material for: Multiple mechanisms drive genomic adaptation to extreme O2 levels in Drosophila melanogaster
Source: Nat Commun. 2021 Feb 12;12:997. doi: 10.1038/s41467-021-21281-6 (PMC7881140; doi:10.1038/s41467-021-21281-6)
Supplement: Supplementary file 1 — Supplementary Information [file 41467_2021_21281_MOESM1_ESM.pdf]

Supplemental Methods

Multiple mechanisms drive genomic adaptation to extreme O_2 levels in *Drosophila melanogaster*

Arya Iranmehr[1, ‡], Tsering Stobdan[2, ‡], Dan Zhou[2, ‡, *], Huiwen Zhao[2],
Sergey Kryazhimskiy[3], Vineet Bafna[4, †, *] and Gabriel G. Haddad[2, 5, 6, †, *]

[1] Department of Electrical & Computer Engineering, University of California San Diego, La Jolla, CA 92093, USA.

[2] Division of Respiratory Medicine, Department of Pediatrics, University of California San Diego, La Jolla, CA 92093, USA.

[3] Division of Biological Sciences, University of California San Diego, La Jolla, CA 92093, USA.

[4] Department of Computer Science & Engineering, University of California San Diego, La Jolla, CA 92093, USA.

[5] Department of Neurosciences, University of California San Diego, La Jolla, CA 92093, USA.

[6] Rady Children's Hospital, San Diego, CA 92123, USA.

[‡], [†] equal contribution

*, correspondence to: ghaddad@health.ucsd.edu, d2zhou@health.ucsd.edu and vbafna@ucsd.edu

Contents

1 Sampling times 3
2 Pre-processing 3
2.1 Read filtering 3
2.2 Variant-calling and Alignment 4
2.3 QC and filtering 4
3 PCA based population substructure 5
4 Experimental evolution Selection Analysis Pipeline (ESAP) 6
4.1 Estimating Population Size 6
4.2 CLEAR Statistic 6
4.3 Fine-Mapping 8
4.4 Fixation Time 9
4.5 Soft Sweep 9
4.5.1 Testing for a single haplotype at fixation using a *Z*-statistic 10
4.5.2 Non-Uniformity (*U*-statistic) 10
4.6 Non-replicated late signals suggest *de novo* events 11
4.6.1 *T*-Statistic for detecting *de novo mutations* 11
4.6.2 Identifying Beneficial Recombination using ANOVA (*F*-statistic) 12
5 Biological Network analysis 12
6 SNP prioritization 13
7 *Drosophila* lines and culture for functional validation 13
8 High O_2 tolerance test 14
9 References 15

1 Sampling times

In determining the time points for sampling, we were guided by some general principles:

1. Increased number of samples in the early part of the cycle (first 100 generations) as selection for such a strong signal would start early and the sweep time would be small based on extensive simulations (Supplementary Fig. 32 which shows the time span of selective sweep (red) versus drift (blue) for multiple values of selection coefficients and starting allele frequencies v of the favored allele. Most of the fixation happens on very early regimen).
2. Sample the L-populations at some later times relative to H-populations, because it took longer for populations to adapt and stabilize in low-oxygen environments (retrospectively shown in Fig. 1c)
3. Availability of high-quality samples at a specific generation.

Based on these considerations, we acquired and sequenced the following samples: N-Population: [4, 17, 180]; H-Population: [1, 7, 12, 31, 61, 114, 162, 180]; and L - Population: [4, 17, 34, 59, 91, 117, 149, 180], each of which had three laboratory replicates. This constitutes a dataset of 56 sample associated with all 3 replicates of L, H and L populations at different generations.

2 Pre-processing

2.1 Read filtering

The sequences were filtered by Novogene and followed standard QC practices,

1. Trim adapters and remove reads containing an excess of adapter. The following Adapter sequences were used:
   - 5’ Adapter

     5’-AATGATACG GCGACCACCG AGATCTACAC TCTTTCCCTA CACGACGCTC TTCCGATCT- 3’

- 3’ Adapter (The underlined 6bp bases is Index)

    5’-GATCGGAAGA GCACACGTC TGAACTCCA GTCACATCACG ATCTCGTAT GCCGTCTTC TGCTTG-3’

2. Remove reads containing excess of N’s (>10%; N represents bases that could not be determined).

3. Remove reads if the number of low quality (Qscore ≤ 5) was over 50% of the total length.

2.2 Variant-calling and Alignment

The sequences were then mapped to a reference *Drosophila melanogaster* genome (release 5.37): ftp://ftp.flybase.net/genomes/Drosophila_melanogaster/dmel_r5.57_FB2014_03/fasta/.

BWA-MEM software version 0.7.8 was used to align the reads to the reference genome. GATK version 3.7 was used to generate gVCF files separately for each sample to call all the bases. The following parameters –T HaplotypeCaller –variant_index_type LINEAR –variant_index_parameter 128000 --emitRefConfidence GVCF were used for gVCF calling. Next, GATK with the parameter -T GenotypeGVCFs integrated all 56 gVCF files to aggregate call polymorphisms based on gVCF likelihoods. Finally, the command:

bcftools filter -i "N _ALT=1 & TYPE=’snp’" joinVariantCalls.vcf | bcftools query -f’%CHROM %POS[%AD]’ was used to extract reference and alternate allele counts for biallelic SNPs.

2.3 QC and filtering

We used Picard tools 2.9.0 with CollectVariantCallingMetrics option to extract metrics summary (Supplementary Table 5). We excluded all variants except the biallelic SNPs that were observed in dbSNP build 137. We first removed the variants within each sample if their coverage was outside the (0.5%, 99.5%) range of genomewide coverage. Next, we removed spurious variants that oscillated between frequency of 0 and 1. We identified them by calculating absolute sum of increments for each base and filtered those that had absolute increments ≥3 (0.99975th quantile). Out of 2,636,334 variants, 1,963,121 variants passed our filtering criteria.

Supplementary Table 5: Picard tools variant metrics summary output

**Category**	**Counts**
TOTAL _SNPS	2636333
NUM _IN DB _SNP	2043558
NOVEL SNPS	592775
FILTERED SNPS	0
PCT DBSNP	0
DBSNP TITV	1
NOVEL TITV	0
TOTAL _INDELS	545561
NOVEL INDELS	544721
FILTERED INDELS	0
PCT DBSNP INDELS	0
NUM _IN DB _SNP INDELS	840
DBSNP INS DEL RATIO	0
NOVEL INS DEL RATIO	0
TOTAL _MULTIALLELIC SNPS	89643
NUM _IN DB _SNP MULTIALLELIC	75618
TOTAL _COMPLEX INDELS	97766
NUM _IN DB _SNP COMPLEX INDELS	178
SNP REFERENCE BIAS	0
NUM _SINGLETONS	201045

3 PCA based population substructure

We used Principal Component Analysis (PCA) to visualize the dynamics of population structure across environments and generations. Specifically, we performed PCA on the matrix M ($m \times n$), where M_{ij} entry corresponds to the allele frequency at j^{th} locus in the ith population. To avoid confounding results, we balanced the number of populations per environment and the number of SNPs for PCA analysis among population. In particular, since N, H, and L-populations have different number of sequenced samples, (9, 26 and 24 respectively), PCA analysis of all samples jointly, would bias results towards the population environments with the most samples. Additionally, populations under selection may have significantly reduced genetic variability in the late generations. Therefore, we chose 9, 12, and 12 samples from N, H and L populations. For each choice of population,

we randomly selected 20,000 polymorphic loci with MAF ≥ 0.1 and performed PCA on the union of 525,644 SNPs.

4 Experimental evolution Selection Analysis Pipeline (ESAP)

4.1 Estimating Population Size

Consider two sampling time points $t_1, t_2 (t_1 < t_2)$, and allele frequency information described by (c_{t_1}, d_{t_1}) and (c_{t_2}, d_{t_2}), where c_t, d_t represent the counts of alternate allele and sequencing coverage, respectively, at time *t*. We used a Wright-Fisher Hidden Markov chain model[1] that assumes fixed population size in a time span, and attributes any change in alternate allele frequency solely to genetic drift. Using the model, we computed the maximum likelihood estimate of the population size.

$$\widehat{N} = \arg\max_{N} \mathcal{L}\left(N \middle| \left[\left(c_{t_1}, d_{t_1}\right), \left(c_{t_2}, d_{t_2}\right)\right]\right) \qquad (1)$$

where the likelihood was calculated using forward-algorithm[2]. The population estimates between each pair of consecutive samples were computed using 20,000 (unlinked) polymorphic loci.

4.2 CLEAR Statistic

We extended the Wright-Fisher Hidden Markov chain to incorporate selection coefficient and over-dominance parameter[3], and calculate a likelihood ratio statistic for selection. For a single locus sampled at *k* time points (*a time-interval*; see below) from the set $\tau = \{t_1 < \cdots < t_k\}$, the *likelihood-ratio* statistic was given by:

$$H_\tau(.) = \frac{\mathcal{L}\left(\hat{s}, \hat{h}, \widehat{N} \middle| \left[\left(c_{t_1}, d_{t_1}\right), \ldots, \left(c_{t_k}, d_{t_k}\right)\right]\right)}{\mathcal{L}\left(0, 0, \widehat{N} \middle| \left[\left(c_{t_1}, d_{t_1}\right), \ldots, \left(c_{t_k}, d_{t_k}\right)\right]\right),} \qquad (2)$$

which was used to calculate CLEAR statistic for window *w* using:

$$\mathcal{H}_{\tau,w} = \frac{1}{|w|} \sum_{i \in w} H_\tau(i) \qquad (3)$$

We averaged the statistic over all loci in sliding windows *w* of width 100kbp, to obtain a statistic $\mathcal{H}_{\tau,w}$ for the window. $\mathcal{H}_{\tau,w}$ was computed for sliding windows using a step size of 50kbp, and over a range of time-intervals τ.

Adjusting for variable population size. While CLEAR suited for fixed population size populations, we made adjustments in calculating likelihood to account for change in population sizes due to bottleneck and recovery. For adjustments, instead of using a single estimate of population size, $\widehat{N}$, we provide CLEAR with a sequence of estimates $\{\widehat{N}_t\} = [\widehat{N}_{t_1-t_2}, \widehat{N}_{t_2-t_3}, \dots, \widehat{N}_{t_{k-1}-t_k}]$ as follows:

$$H_\tau(.) = \frac{\mathcal{L}\left(\hat{s},\hat{h},\{\widehat{N}_t\}\middle|\left[\left(c_{t_1},d_{t_1}\right),\dots,\left(c_{t_k},d_{t_k}\right)\right]\right)}{\mathcal{L}\left(0,0,\{\widehat{N}_t\}\middle|\left[\left(c_{t_1},d_{t_1}\right),\dots,\left(c_{t_k},d_{t_k}\right)\right]\right)}, \qquad (4)$$

Where

$$\mathcal{L}\left(s,h,\{\widehat{N}_t\}\middle|\left[\left(c_{t_1},d_{t_1}\right),\dots,\left(c_{t_k},d_{t_k}\right)\right]\right) = \prod_{i=1,j=i+1}^{k-1} \mathcal{L}\left(s,h,\widehat{N}_{t_i-t_j}\middle|\left[\left(c_{t_i},d_{t_i}\right),\left(c_{t_j},d_{t_j}\right)\right]\right) \quad (5)$$

Choosing time-intervals for CLEAR computation. In choosing appropriate time intervals, we were guided by three considerations. First, in a Wright Fisher model with population size N, an allele could reach fixation simply via genetic drift in approximately 2N generations. Given the small effective population sizes relative to the length of the experiment (Fig 1c), we limited the time-intervals to 120 generations (Supplementary Fig. 4). Second, a selective sweep could start anytime during evolution, initially with extant alleles that were favored by solution, and subsequently due to *de novo* mutations or recombination events. However, considering all possible time intervals would reduce statistical power to multiple testing. Therefore, we only investigated time intervals beginning at generations $g \in \{1,3,60\}$ and limited the length of interval to be $\sim$120 generations. Specifically, for H and L-populations, we used the time intervals described in the Supplementary Table 6.

Supplementary Table 6: Time-windows in which genome-wide scans of selection are performed.

Population	Start Generation	End Generation	Length
L-population	4	117	113

L-population	34	149	115
L-population	59	180	121
H-population	1	114	113
H-population	31	162	131
H-population	61	180	119

Enforcing Fixation. CLEAR considers the rapid increase of allele frequency as a signal of selection, and its power has been previously validated on short experiments[3]. In our data, however, we also observed non-monotonic trajectories, where many mutations arose in frequency to near fixation and then went down in frequency. As an example, the region shown in Supplementary Fig. 24 had a highly significant CLEAR signal in the interval 4-117. However, the mutations did not fixate and the signal was weakened after generation 117, suggesting a spurious signal. To emphasize fixation at generation 180, we used a weighted CLEAR statistic. For time interval τ and genomic window w, we defined $\Lambda_{\tau,w}$, as a scaling of the CLEAR statistic by the proportion of non-polymorphic variants at generation 180: (Supplementary Fig 33):

$$\Lambda_{\tau,w} = \mathcal{H}_{\tau,w}.\beta_w, \text{where } \beta_w = \frac{\left|\left\{y_i^{(180)} \middle| i \in w, y_i^{(180)} < 0.01\right\}\right|}{|w|} \qquad (6)$$

where $y_i^{(180)}$ is the minor allele frequency of the variant at generation 180.

Genome-wide significance. As the initial haplotypes and linkage blocks and effective population size are unknown, and populations undergo massive demographic changes, estimating empirical null distribution of the CLEAR statistic is difficult. Instead, for a time interval τ, we computed the mean and variance of the CLEAR statistic $\mathcal{H}_{\tau,w}$ across all genomic windows, and selected all windows with a CLEAR score 5.5 standard deviations or higher than the mean (red dotted lines).

4.3 Fine-Mapping

The genome-wide scan was performed using a *coarse-search*: sliding windows of 100Kb and step size of 50Kb. While this choice of parameters was good for capturing dominating genome-wide scans, they did not properly separate the core selected haplotype(s) from

flanking regions. Therefore, each region selected in the coarse-search was subjected to a local scan using steps of 1kb to identify an appropriately sized window maximizing the local signal.

4.4 Fixation Time

For time interval τ_0, denote beg[τ_0] and end[τ_0] as the times of the first and last sampling in τ_0. For a significant CLEAR statistic $\mathcal{H}_{\tau 0,w}$ over a genomic window w and time-interval τ_0, we identified a new 'end of time-interval' at which the CLEAR statistic was maximized to give the *fixation time*, $T_{\text{fix}}[w]$. Specifically,

$$T_{fix}[\tau, w] = \underset{\text{beg}[\tau] < t_e \leq 180}{\arg\max} \mathcal{H}_{\tau,w} \text{ for } \text{beg}[\tau] = \text{beg}[\tau_0], \text{end}[\tau] = t_e. \quad (7)$$

4.5 Soft Sweep

Unlike hard-sweep (Fig. 2b), in which mutations on a single genetic background drive adaptation, soft sweep due to standing variation has a distinct signature at fixation. While the favored mutation reaches fixation, distinct haplotypes carrying the favored mutation persist and interfere with each other. These haplotypes are manifested as clusters of strongly correlated mutation in the time-series data (Fig. 2c). To test a CLEAR identified region (τ, w) for soft-sweep with standing variation, we used the following steps:

1. Compute Fixation time $T_{\text{fix}}[\tau, w]$ (Eqn. 7).
2. Used a Z-statistic (below) to test the Null hypothesis of a hard-sweep, which suggests that at $T_{\text{fix}}[\tau, w]$, all mutations in w cluster into a single frequency near fixation. A significant deviation from the Null hypothesis was indicative of a soft-sweep with standing variation.
3. If the Z-statistic was significant, we used a U-statistic (below) to distinguish a soft-sweep from flanking regions of a hard sweep ('soft-shoulders'[4]).
4. If the Z-statistic significant and U-statistic was not significant, the region was identified as a soft-sweep with standing variation; otherwise, it was identified as a shoulder of a proximal hard-sweep.

4.5.1 Testing for a single haplotype at fixation using a *Z*-statistic.

To determine if a region under selection adapted using a hard sweep or soft sweep on standing variation, we tested if a single haplotype in the region fixated in the population (Null hypothesis). Let $x_i = c_i/d_i$ denote the allele frequency at locus *i* at *fixation time*, and we 'fold' it to obtain y_i:

$$x_i = {}^{c_i}/_{d_i} \qquad \text{observed allele frequency} \qquad (8)$$

$$y_i = \begin{cases} x_i & x_i \leq 0.5 \\ 1 - x_i & x_i > 1 \end{cases} \text{observed MAF} \qquad (9)$$

Under the Null hypothesis of a hard-sweep, all the mutations should have the same folded allele frequency nearing 0 at fixation, and any variability in actual observations can be attributed to sampling. We therefore calculate a Z-statistic for a region as:

$$\bar{y} = \frac{1}{n}\sum_i^n y_i \qquad (10)$$

$$Z = \sum_i^n \frac{(\bar{y} - y_i)^2}{\bar{y}} \qquad (11)$$

$$Z \sim \mathcal{X}_{n-1}^2 \qquad (12)$$

To avoid inflated statistic due to variant calling errors, we simply removed outliers by excluding the top 1% of the MAFs. A sharp deviation of *Z* away from 0 near fixation indicated that we could reject the null hypothesis of a single haplotype being driven to fixation and predict a putative soft sweep due to standing variation.

4.5.2 Non-Uniformity (*U*-statistic)

A hard-sweep is often characterized by strong haplotype homozygosity as measured by the Z-statistic. However, in the flanking (soft-shoulders) regions, the core haplotypes sometimes diverge into multiple haplotypes similar to soft-sweep with standing variation[4].

To distinguish between a soft-sweep and soft-shoulders of a hard-sweep, we observed that under a soft-sweep, the collection of mutations due to standing variation would be interleaved in location with the clusters of mutations that were fixed. In contrast, the haplotypes in the flanking regions of a hard sweep would be interrupted in the middle by a single fixed cluster of mutations tightly linked to the favored mutation. For example, the

interval L_D in Fig. 3a, shaded with blue color, shows a hard sweep where the dominant haplotype is fixating in the later generations. Conspicuously, at generation 91 and later, the flanking haplotype is being disrupted by a large non-polymorphic region in the middle.

We defined the null hypothesis to be soft sweep and under null, we expect the fixed mutations and the intermediate frequency mutations due to standing variation to be randomly distributed and interleaved. A depletion of standing variation (core region of a hard sweep) would be result in a significant deviation from the Null hypothesis. We ordered the standing variations and the fixed mutations by position and used a Mann-Whitney *U* test to compute the randomness of interleaving. If a significant P-value was obtained, the region was designated as the shoulder of a hard sweep; otherwise, it was identified as a soft sweep.

4.6 Non-replicated late signals suggest *de novo* events

Due to a small underlying mutation rate in small populations, the probability of 3 *de novo* favored mutations in the same region occurring synchronously in 3 replicates is infinitesimal. Therefore, almost surely, we designated 3-way replicated sweep signals to be driven by standing variation.

Unreplicated signals of a hard sweep, however, could be due to two reasons. First, they could be due to a rare, unreplicated *de novo* favored mutation in one of the populations. Second, they could be due to standing variation which existed in all replicated populations but was lost early due to genetic drift prior to establishment.

We reasoned, however, that standing variation would either be lost early, or reach fixation soon after onset of selection, and the occurrence of an unreplicated hard sweep *long after* the onset of selection was indicative of a *de novo* mutation, which could be tested using a *T*-statistic.

4.6.1 *T*-Statistic for detecting *de novo mutations*

Consider a genomic window w and time-interval τ with a beg[τ] = t_1 and end[τ] = t_2. L_A and s^* denote the CLEAR likelihood and ML estimate of selection pressure s based on the alternative hypothesis of the sweep occurring in time-interval marked by boundaries [t_1,t_2].

We contrast this with likelihood L_N of the null hypothesis– the sweep occurred in time-interval $[0,t_2]$, or began with the onset of selection. Defining the T-statistic as

$$T = 2[log(\mathcal{L}_A(data|s^*) - log(\mathcal{L}_N(data|s^*)),] \qquad (13)$$

we used the χ^2 test to compute a p-value for rejecting the null hypothesis. A significant *p*-value suggested that the sweep started 'late' and could be attributed to a *de novo* event.

Supplementary Fig. 34 illustrates the test for 3 simulated replicates under experimental evolution for 200 generations with selective sweeps starting at different time points. In tests sampling at generations {0,50,100,150,200}, the T-statistic correctly distinguished late from early sweeps. Sweeps that were designated late and not replicated were attributed to *de novo* events, and a Y -statistic was used to identify the specific type of event.

4.6.2 Identifying Beneficial Recombination using ANOVA (*F*-statistic)

A *late hard sweep* identified by ESAP that is not replicated is indicative of a *de novo* mutation, or a beneficial recombination event. We term these as FM-recombination events. To distinguish between the two possibilities, we traced the fixed mutations back to their ancestral generations. Specifically, we identified a cluster X of mutations on the haplotype that was close to fixation (all alleles have frequencies > 0.9. In previous generations, the minor allele frequencies of these mutations will diverge into multiple clusters. The key distinction between *de novo* mutations and a beneficial recombination event is that in the recombination case, the two clusters would represent different haplotypes that are separated by the point of recombination. For each genomic location x in a window under a late hard-sweep, we partitioned mutations in X on either side of x into the clusters L_x and R_x and performed an F-test for significant reduction in Var(L_x)+Var(R_x) relative to Var(X). If the P-value was significant after Bonferroni correction, then the sweep was classified as an FM-recombination.

5 Biological Network analysis

To determine which Gene Ontology (GO) categories are statistically overrepresented in a set of genes, we used software tool BiNGO (Biological Network Gene Ontology) version 3.0.3 plugin on Cytoscape 3.8.0, an open-source bioinformatics software platform for

visualizing and integrating molecular interaction networks. Here we used the 99 previously reported human high-altitude genes that were ortholog to 80 of the 433 L-interval fly genes. We used the GO term for ‘molecular functions’ to test for enrichment. The statistically overrepresented GO terms were color coded (Fig 4).

6 SNP prioritization

In order to prioritize important SNPs we followed a series of filters, selecting SNPs that (a) were at fixation in all chambers; (b) represented non-synonymous coding mutations; and, (c) the reference allele was conserved in multiple *Drosophila* species. For example, for the L_A interval 17,876 out of 37,269 total SNPs had frequencies that were < 20% or > 80% at the 180th generation in all the three biological replicates (chambers). 293 SNPs were identified as non-synonymous, and 61 had the reference nucleotide conserved in 11 of the 12 *Drosophila* species from *Drosophila* 12 Genomes Consortium[5]. The corresponding numbers for L_B were 7,899 (total), 3,570 (fixated), 85 (non-synonymous) and 19 (conserved).

7 *Drosophila* lines and culture for functional validation

For the functional validation, we took an unbiased approach. The genes from the top interval i.e., H_A interval, for which an *RNAi* fly line were selected for validation. The *RNAi* fly lines for the selected candidate genes, as indicated in Supplementary Table 7, were purchased from Bloomington *Drosophila* Stock Center (BDSC), Indiana University. In order to ubiquitously knock-down the candidate gene in the F1 progeny the *da-Gal4* driver was also obtained from BDSC. The *UAS-RNAi* × *da-Gal4* crosses were considered as experimental and the individual *RNAi* lines as specific controls. This method was previously utilized to validate the role of *CIC* gene we found selected in high altitude human population (mentioned in the discussion section). Since the *RNAi* lines are built on a y^1v^1 genetic background, we used the y^1v^1 x *da-Gal4* along with y^1v^1 and *da-Gal4* as background control. All the stock lines were raised at 22°C and maintained on standard corn meal. Both experimental and controls were first cultured in normoxia to determine

whether the *RNAi*-mediated knockdown of each candidate gene by itself has any effect on development.

Supplementary Table 7: *Drosophila* RNAi lines used for functional validation

Gene	Flybase stock ID	Genotype
peb	28735	y[1] v[1]; PTRiP.JF03162attP2
CG12184	63721	y[1] v[1]; PTRiP.HMJ30289attP40
pon	35046	y[1] sc[*] v[1]; PTRiP.HMS01460attP2
CG3062	64013	y[1] v[1]; PTRiP.HMJ30326attP40
HLH4C	25976	y[1] v[1]; PTRiP.JF01998attP2
Torsin	50620	y[1] v[1]; PTRiP.HMC02987attP2/TM3, Sb[1]
CG3009	31129	y[1] v[1]; PTRiP.JF01602attP2
Pp2C1	40827	y[1] sc[*] v[1]; PTRiP.HMS01887attP2
ctp	44044	y[1] sc[*] v[1]; PTRiP.HMS02760attP2
CG7024	62226	y[1] sc[*] v[1]; PTRiP.HMC05233attP40
Proc-R	29414	y[1] v[1]; PTRiP.JF03350attP2
CG15472	62977	y[1] v[1]; PTRiP.HMJ30054attP40
Klf15	27075	y[1] v[1]; PTRiP.JF02420attP2
CG2861	50931	y[1] v[1]; PTRiP.HMJ21025attP40
CG42594	25888	y[1] v[1]; PTRiP.JF01929attP2

8 High O_2 tolerance test

Three to five day-old *da-Gal4* males (n=10) were crossed to female *UAS-RNAi* line (female, n=10) targeting specific gene. Sufficient time was given (3 days) for the flies to mate/cross and these are referred to as ‘cross’. Simultaneously the y^1v^1, *da-Gal4* and *RNAi* were ‘self-crossed’ and the $y^1v^1 \times$ *da-Gal4* (control). We utilized H population flies as the positive controls. Each set of crosses were in triplicate. The vials were kept under ambient conditions for 48 hours so that the flies can lay sufficient number of fertilized eggs. After 48 hours, the adults were transferred to a new vial. For the hyperoxia tolerance test and the original vials were then transferred to a computer controlled high O_2 chamber, constantly maintained at 80% O_2. Chambers were in the same room as ambient O_2 controls with 12/12 hours light/dark cycle (temperature 22˚C). The adults from the new vials i.e., from the second batch of vials, were discarded after 48 hours and the vials with the fertilized eggs were kept at ambient O_2 conditions (21% O_2) also with 12/12 hours light/dark cycle (temperature 22˚C). These were the control vials. After 21 days, the ratio of the empty

pupae (eclosed) to the total number of pupae formed (eclosed + uneclosed) in each vial was calculated to determine the eclosion rate. The differences in eclosion rate at 80% O_2 between the *RNAi* × *daGal4* and all the controls were assessed using paired sample t-test. A p value of <0.05 was considered statistically significant.

References

1 Bollback, J. P., York, T. L. & Nielsen, R. Estimation of 2Nes from temporal allele frequency data. *Genetics* **179**, 497–502 (2008).

2 Durbin, R., Eddy, S. R., Krogh, A. & Mitchison G. *Biological sequence analysis: probabilistic models of proteins and nucleic acids*. Cambridge university press (1998).

3 Iranmehr, A., Akbari, A., Schl¨otterer, C & Bafna, V. CLEAR: Composition of likelihoods for evolve and resequence experiments. *Genetics* **206,** 2 (2017).

4 Schrider, D. R., Mendes, F. K., Hahn, M. W. & Kern, A. D. Soft shoulders ahead: spurious signatures of soft and partial selective sweeps result from linked hard sweeps. *Genetics* **200**, 267–284 (2015).

5 *Drosophila* 12 Genomes Consortium. Evolution of genes and genomes on the *Drosophila* phylogeny. *Nature* **450**, 203-218 (2007).

Supplementary Fig. 1. PCA analysis using all SNPs and the percentage of explained variance.

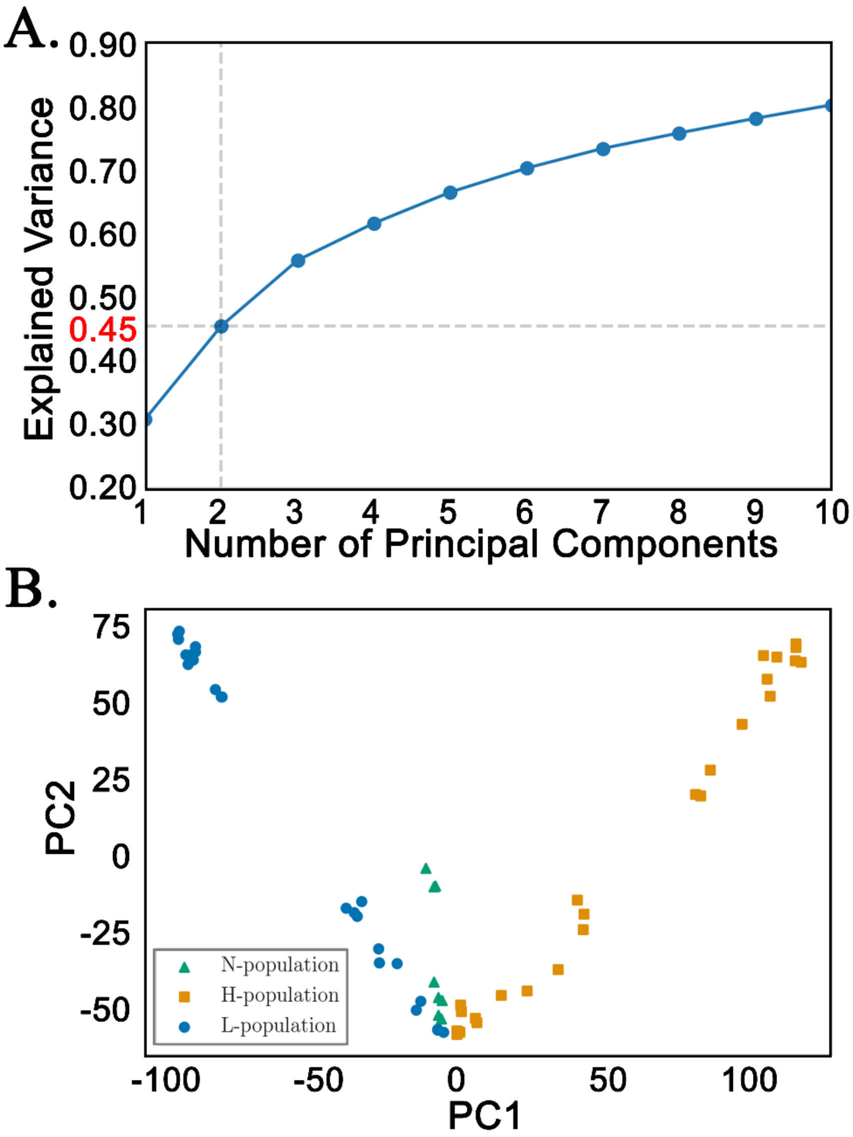

Supplementary Fig. 1. Principal component analysis and the percentage of explained variance. (A) Plot depicting the percentage of explained variance by different numbers of principal components. The percentage of explained variance by PC1+PC2 is 0.454. Source data are provided as Supplementary Fig 1A Source Data. (B) PCA analysis using all (*de novo* and extant) SNPs matches PCA analysis using only extant SNPs (Fig. 1D). Source data are provided as Supplementary Fig 1B Source Data.

Supplementary Fig. 2. Rate of evolution measured by Fst per generation

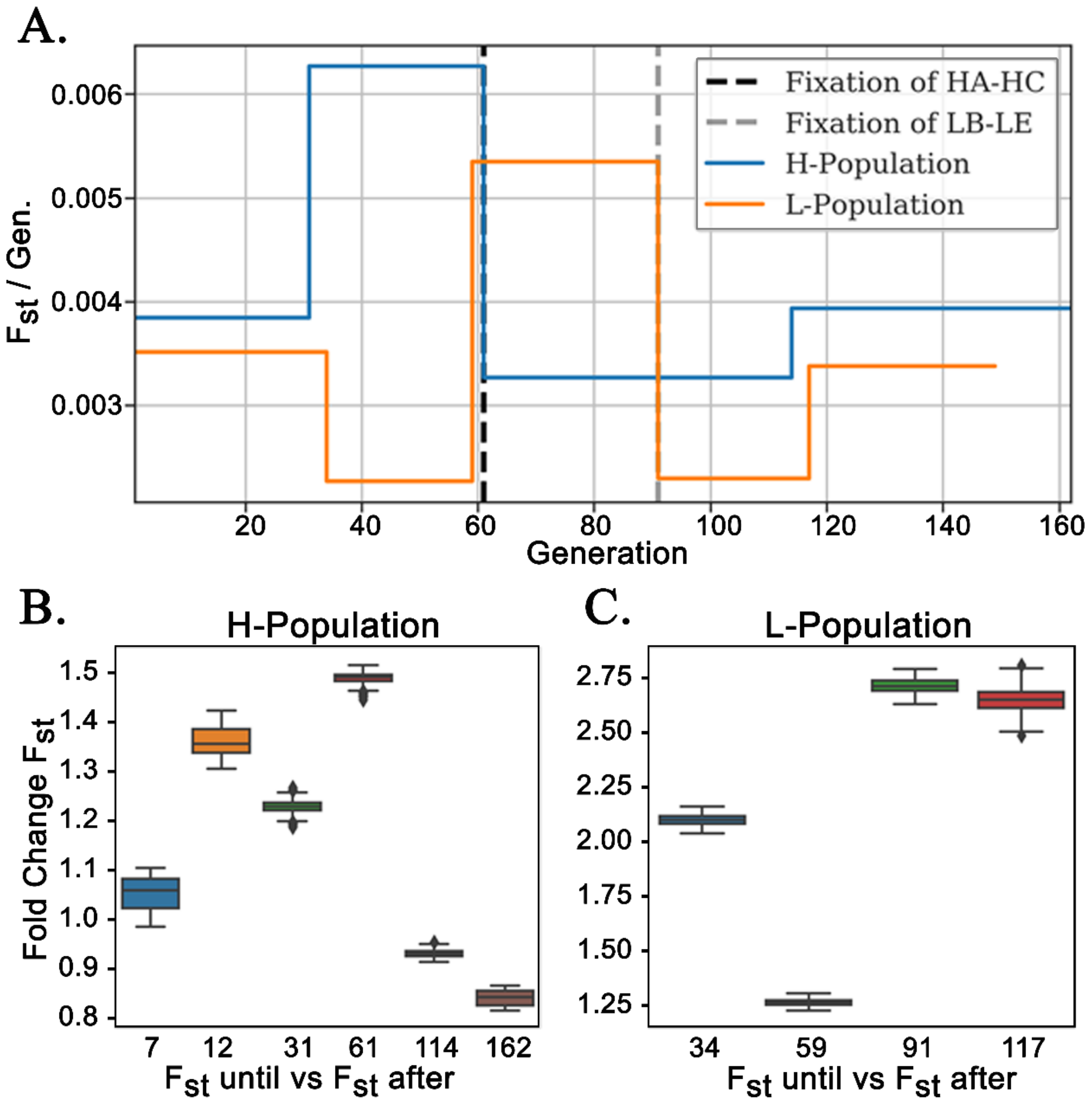

Supplementary Fig. 2. Rate of evolution measured by F_{st} per generation. (A) Pairwise F_{st} between consecutive generations representing genomewide divergence along time. Source data are provided as Supplementary Fig 2A Source Data. (B) and (C) For each sampled generation, the ratio of F_{st} change prior to that generation to F_{st} change subsequently is plotted. The ratio is maximized at generation 61 for H-populations (B) and 91 for L-populations (C). The divergence in the first 60 generations of the H-population during adaptation exceeded the divergence in the next 120 generations by 1.49-fold. Similarly, the divergence in the first 90 generations of the L-populations was 2.71-fold the divergence in the next 90 generations. The whiskers extend from the minimum value and maximum value. N=400 variants per generation for each of the boxes in (B) and (C). Source data are provided as Supplementary Fig 2B Source Data and Supplementary Fig 2C Source Data.

Supplementary Fig. 3. Drift and Selection Model

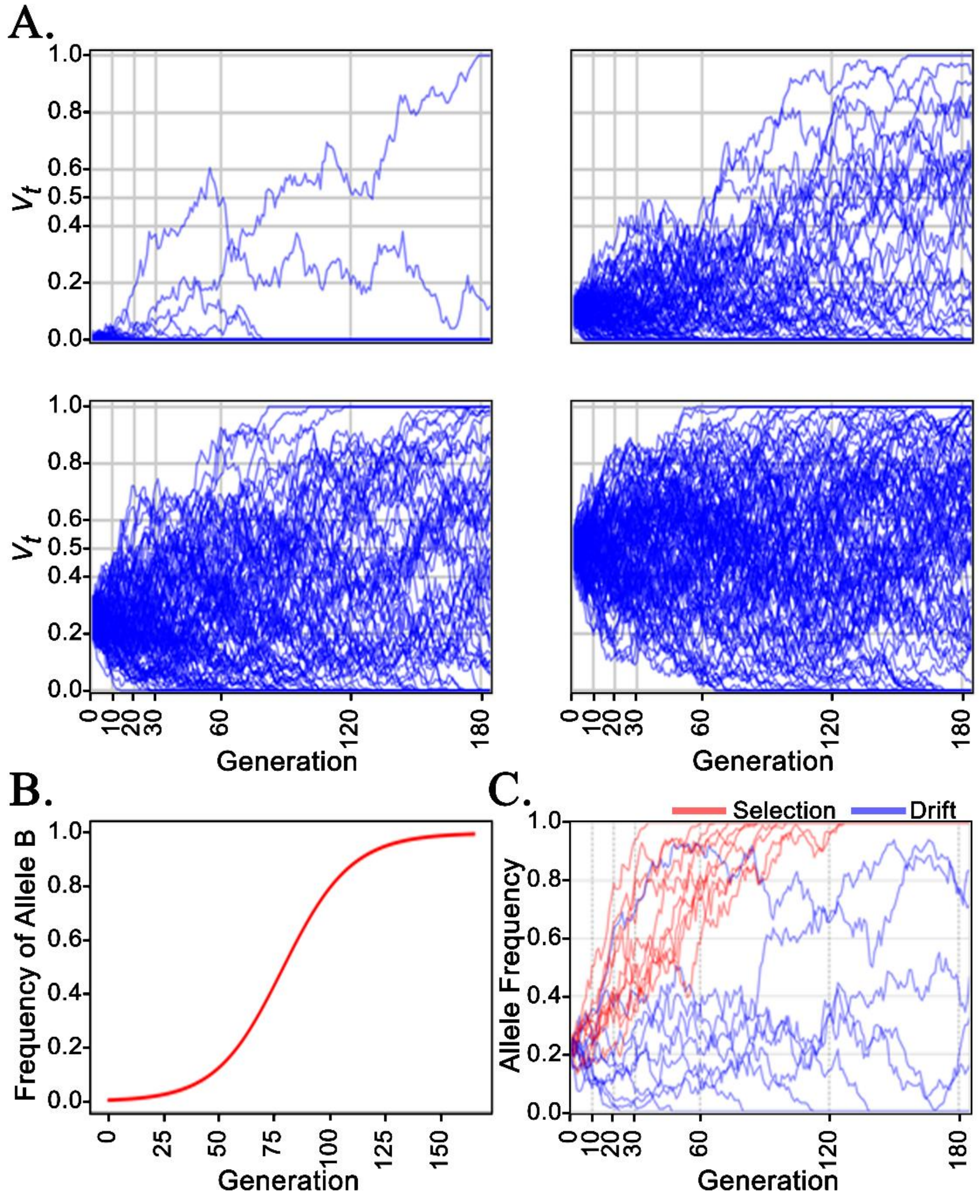

Supplementary Fig. 3. Model depicting trajectories of the mutation (and linked hitchhikers) favored by the selective sweep versus the trajectory of drifting mutations. (A) Instantiation of genetic drift for a population size of N=200 starting at 1/N, 0.1, 0.3, 0.5 frequency. (B) Logistic growth model for selection. (C) Trajectories of the mutations in the case of genetic drift vs selection sweep in a finite population size. Dominance coefficient = 0.5.

Supplementary Fig. 4. Power of test of selection decreases in longer time-intervals

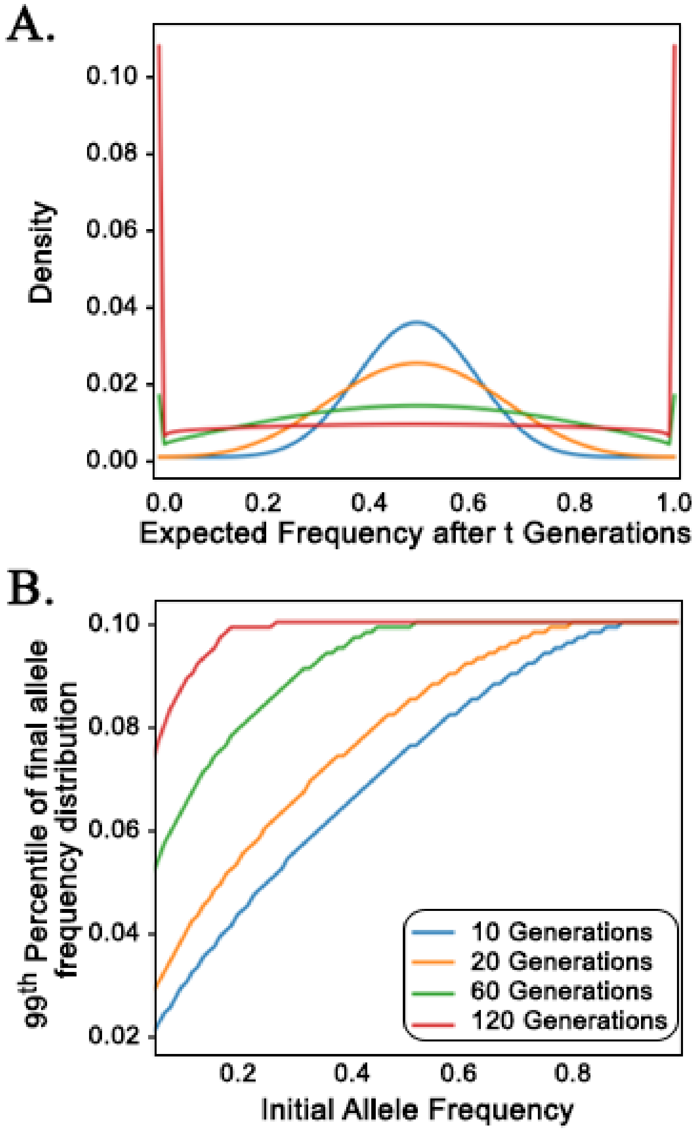

Supplementary Fig. 4. Power of test of selection decreases in longer time-intervals. (A) Distribution of allele frequencies due to genetic drift. All simulations start with allele frequency 0.5, and were sampled after 10, 20, 60 and120 generations for Ne=200. (B) Final allele frequencies as a function of starting allele frequencies. The y-axis represents the 99th percentile of distribution of allele frequencies after 10,20,60,120 generations under genetic drift (Null Hypothesis). Note that 1% of *de novo* mutations reach a frequency of 0.75 after 120 generations reducing the power of test of selections over a 120 generation time-interval. However, 99% of drifting *de novo* mutations remain below 52% after 60 generations.

Supplementary Fig. 5. Schematic of Evolution Selection Analysis Pipeline (ESAP).

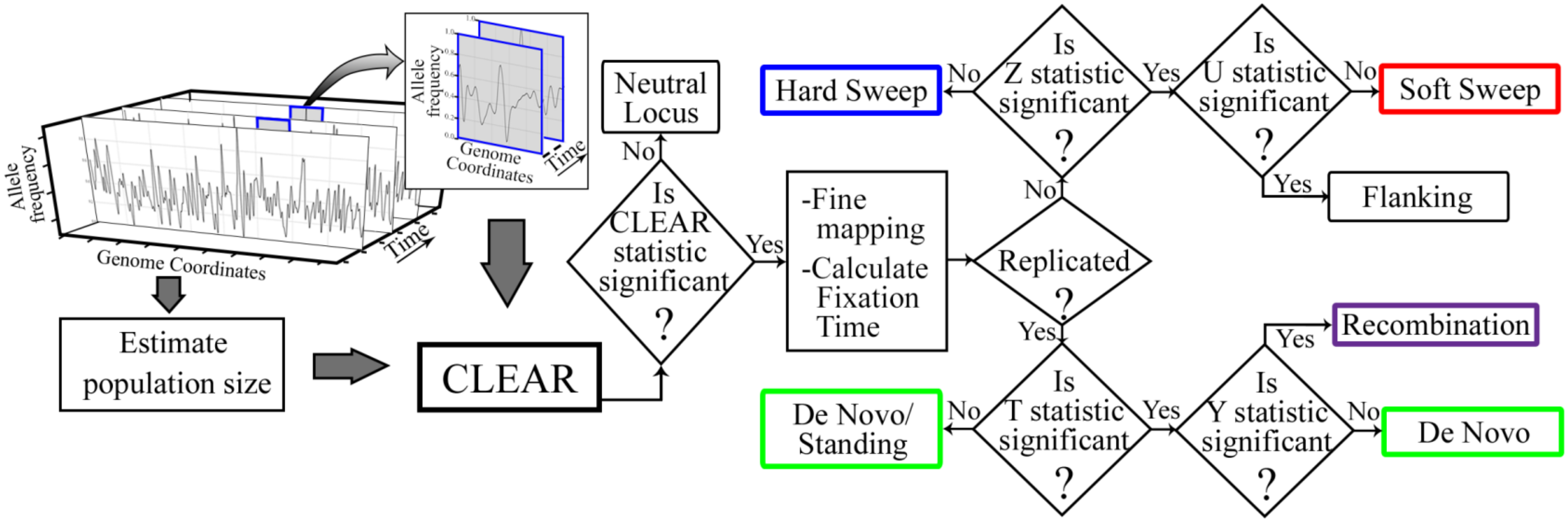

Supplementary Fig. 5. Schematic of Evolution Selection Analysis Pipeline (ESAP). The Experimental Evolution Selection Analysis Pipeline (ESAP) was developed to identify the mechanisms of selection. The CLEAR method identifies genomic loci under selection. ESAP starts with the genomic window and a time interval slice identified by CLEAR as undergoing selective sweeps in each time interval, and outputs the mechanism of selection as a hard-sweep, a soft-sweep on standing variation, *de novo* mutation, flanking soft-shoulder of a hard-sweep, or an FM-recombination.

Supplementary Fig. 6. L-Population Replicated Sweeps

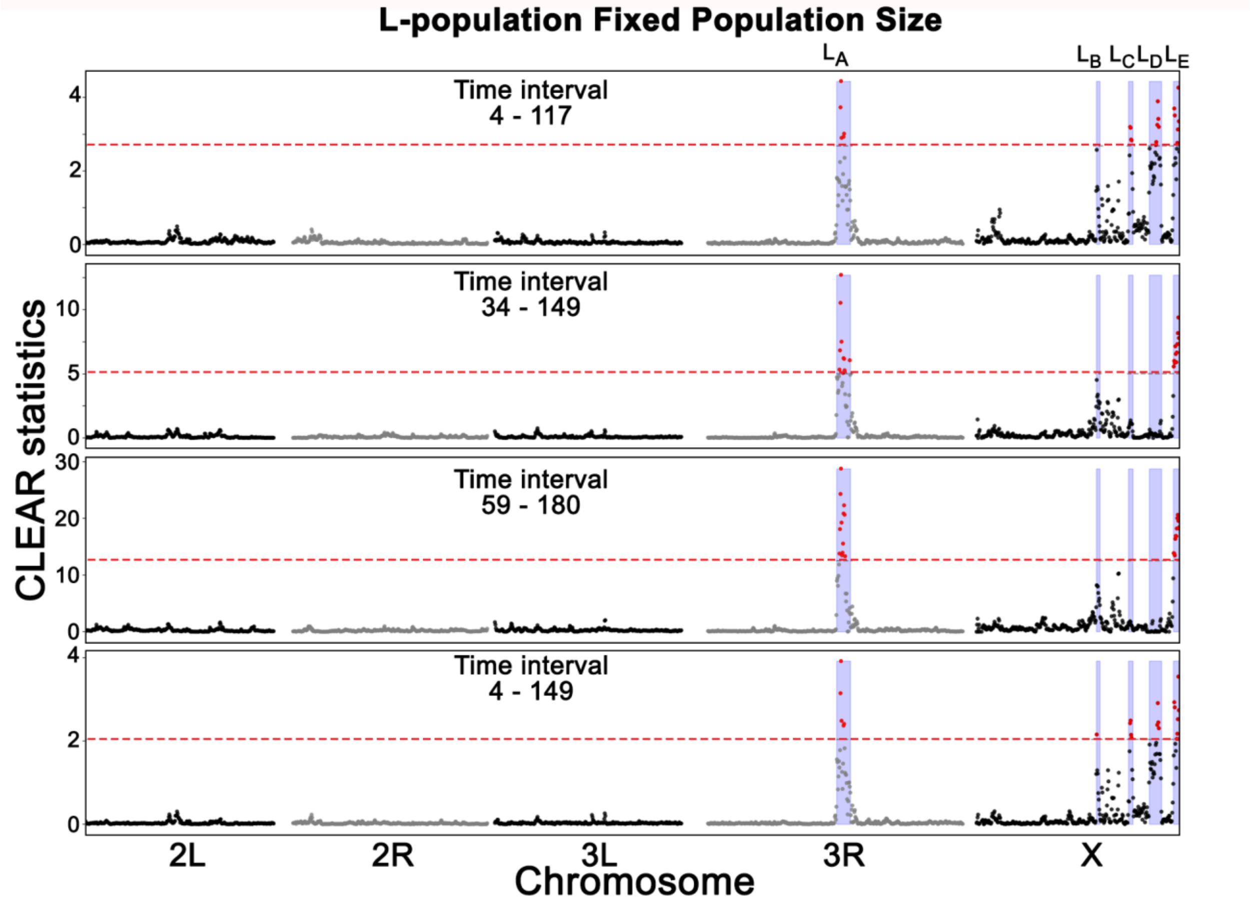

Supplementary Fig. 6. L-Population Replicated Sweeps. The four panels show 4 time intervals and the average of CLEAR statistics (y-axis of each panel) from the three replicates, with peaks corresponding to replicated sweeps in the respective time intervals. Five replicated sweeps i.e., LA-LE, where LA is located on chromosome 3 and remaining intervals on chromosome X were identified in the L-population. Additional details for individual sweeps depicting their allele frequency trajectories are provided in Supplementary Fig. 10-S14. The red dotted line depicts the cut-off threshold of 5.5 standard deviation from the mean.

Supplementary Fig. 7. H-Population Replicated Sweeps

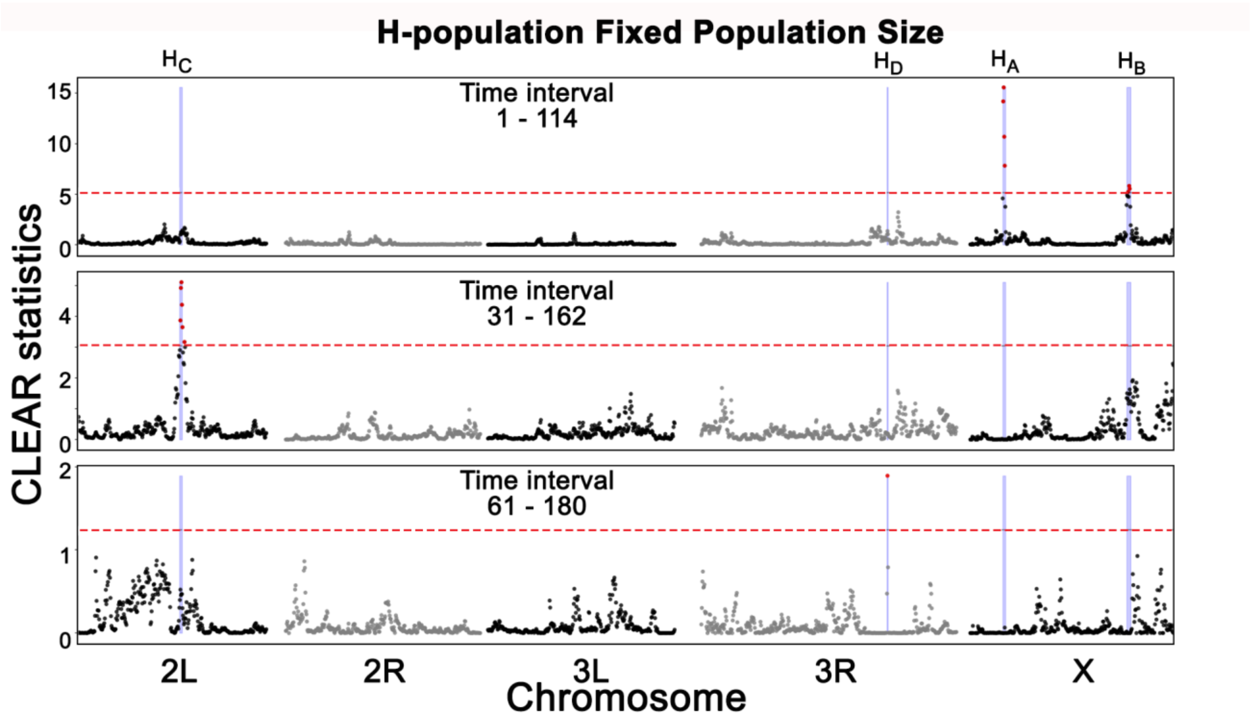

Supplementary Fig. 7. H-Population Replicated Sweeps. The three panels show 3 time intervals and the average of CLEAR statistics (y-axis of each panel) from the three replicates, with peaks corresponding to replicated sweeps in the respective time intervals. Four replicated sweeps i.e., H_A-H_D, were identified in the H-population. Additional details for individual sweeps depicting their allele frequency trajectories are provided in Supplementary Fig. 15-S18. The red dotted line depicts the cut-off threshold of 5.5 standard deviation from the mean.

Supplementary Fig. 8. CLEAR statistics Manhattan plot for L-Population (A) and H-Population (B) Replicated Sweeps with adjusted population size.

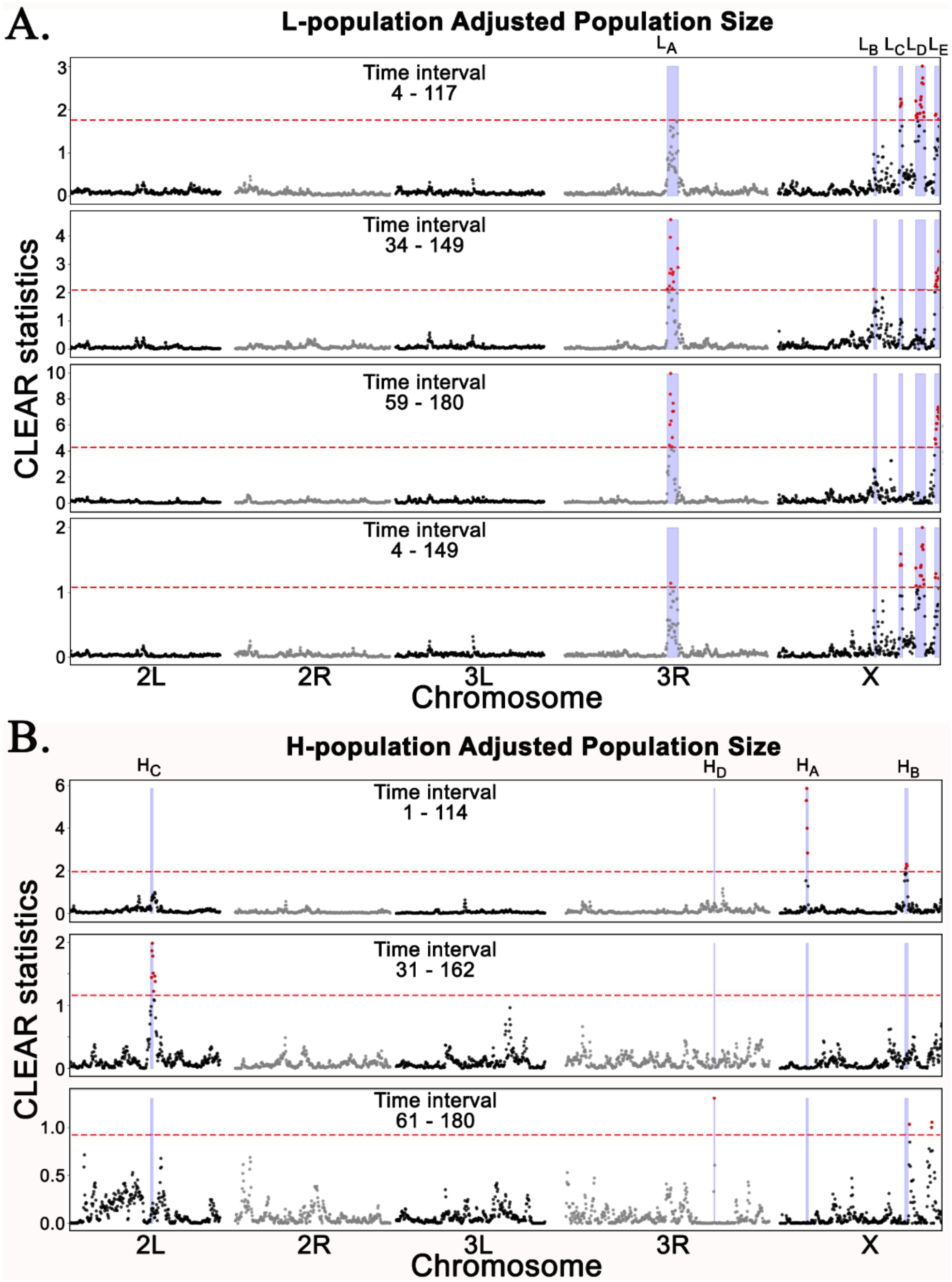

Supplementary Fig. 8. CLEAR statistics for L-Population and H-Population with adjusted population size. The five replicated sweeps for L_A through L_E (A) and four replicated sweeps for H_A through H_D (B) remain significant after adjusting for changes in population size. The cut-offs for both statistics was the 5.5 standard deviation from the mean. All selected regions i.e., above the red dotted line, remained significant in both fixed (as depicted in Supplementary Figure 6 and 7) and adjusted population size regime, due to strength of signal and stringency of cutoffs.

Supplementary Fig. 9. Major allele frequency spectra (AFS) at generation 180 in every selected region of the L and H populations and the corresponding AFS of the N population at the same locus.

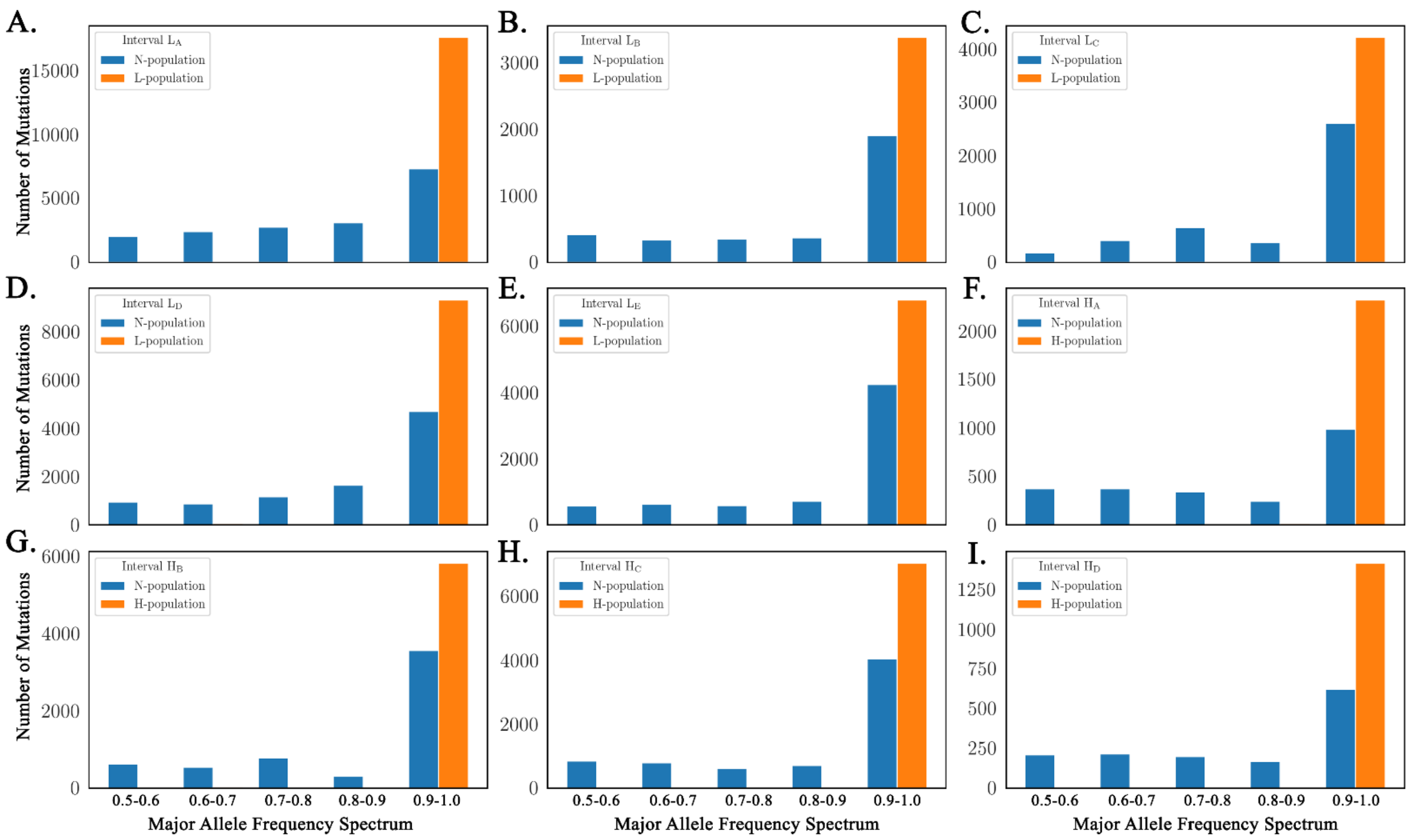

Supplementary Fig. 9. Allele frequency spectra (AFS) at generation 180 in every selected region of the L and H populations and the corresponding AFS in the N population at the same locus. The substantial polymorphic variations in the N-populations are completely fixed in the corresponding H- and L- populations in every selected region indicated in each panel (A to I), suggesting that background selection is not determining the selection signals in L and H populations. Source data are provided as Supplementary Fig 9 Source Data.

Supplementary Fig. 10. Interval L_A

A.

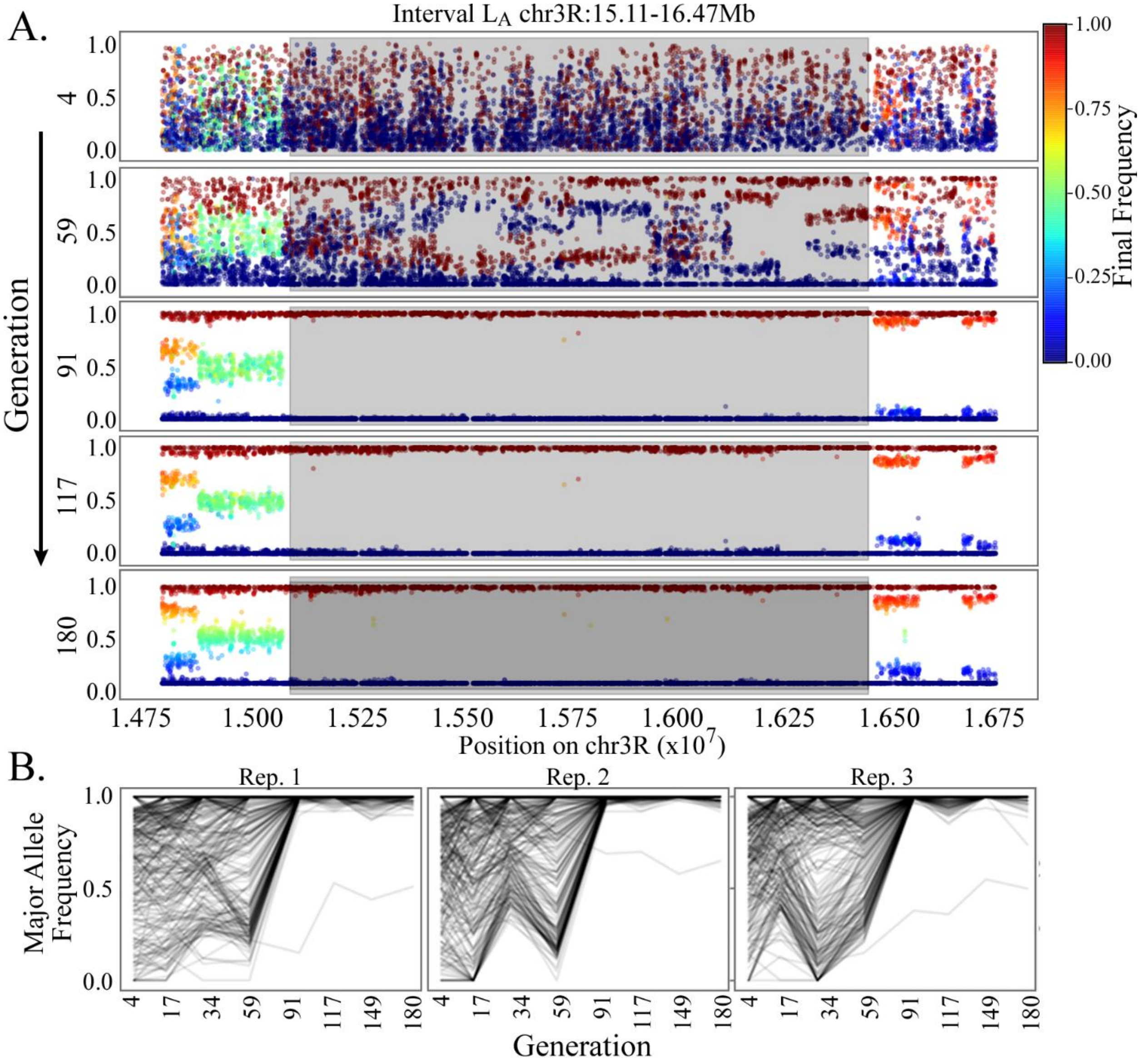

Supplementary Fig. 10. Interval L_A. (A) Spatial and frequency distribution of alleles in interval L_A. The color depicts the frequency at generation 180 of the respective allele. (B) The allele frequency trajectories for the SNPs in interval L_A in each individual replicates. Note the synchronization in replicate chambers.

Supplementary Fig. 11. Interval L_B

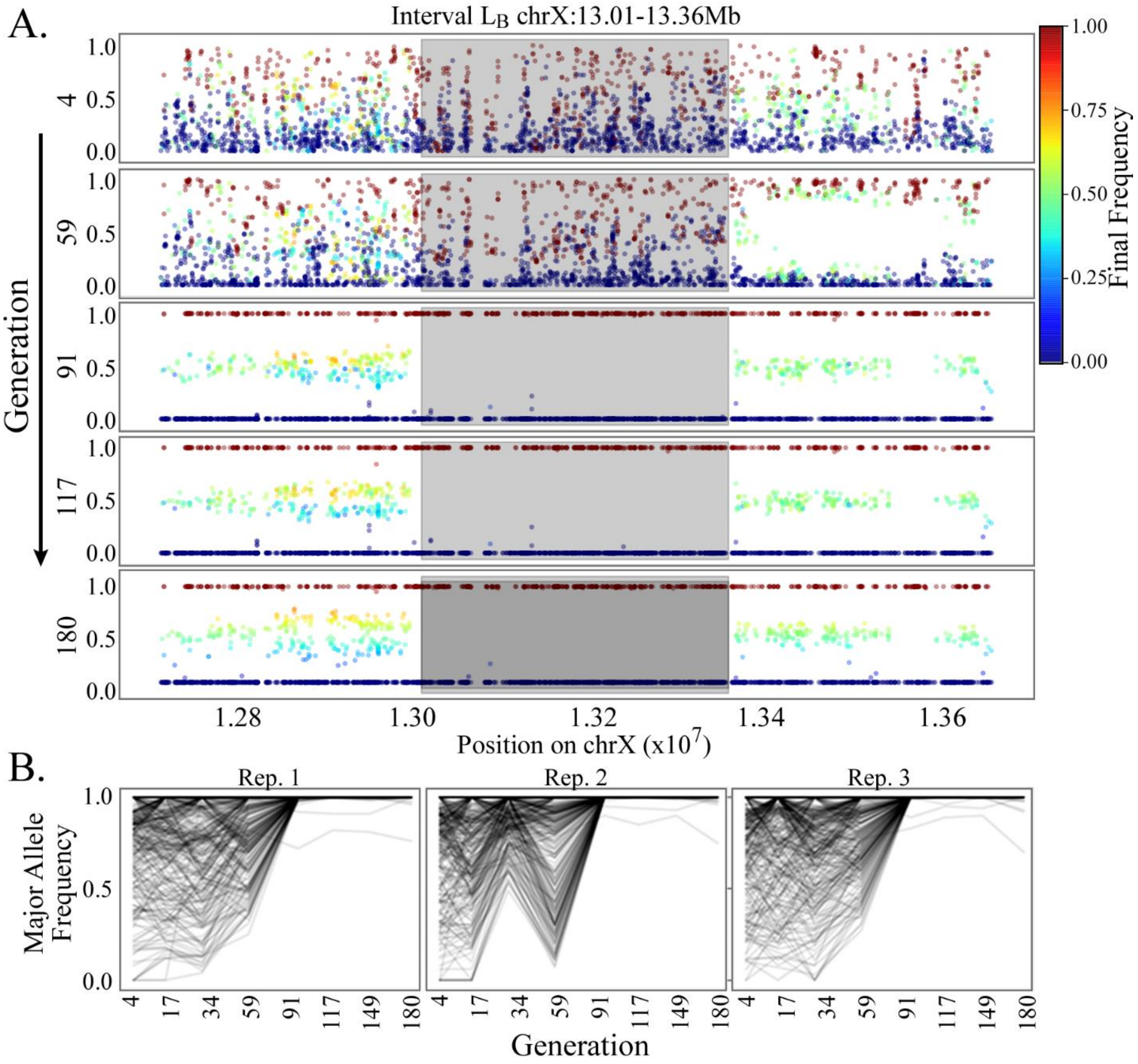

Supplementary Fig. 11. Interval L_B. (A) Spatial and frequency distribution of alleles in interval L_B. The color depicts the frequency at generation 180 of the respective allele. (B) The allele frequency trajectories for the SNPs in interval L_B in each individual replicates. Note the synchronization in replicate chambers.

Supplementary Fig. 12. Interval L_C

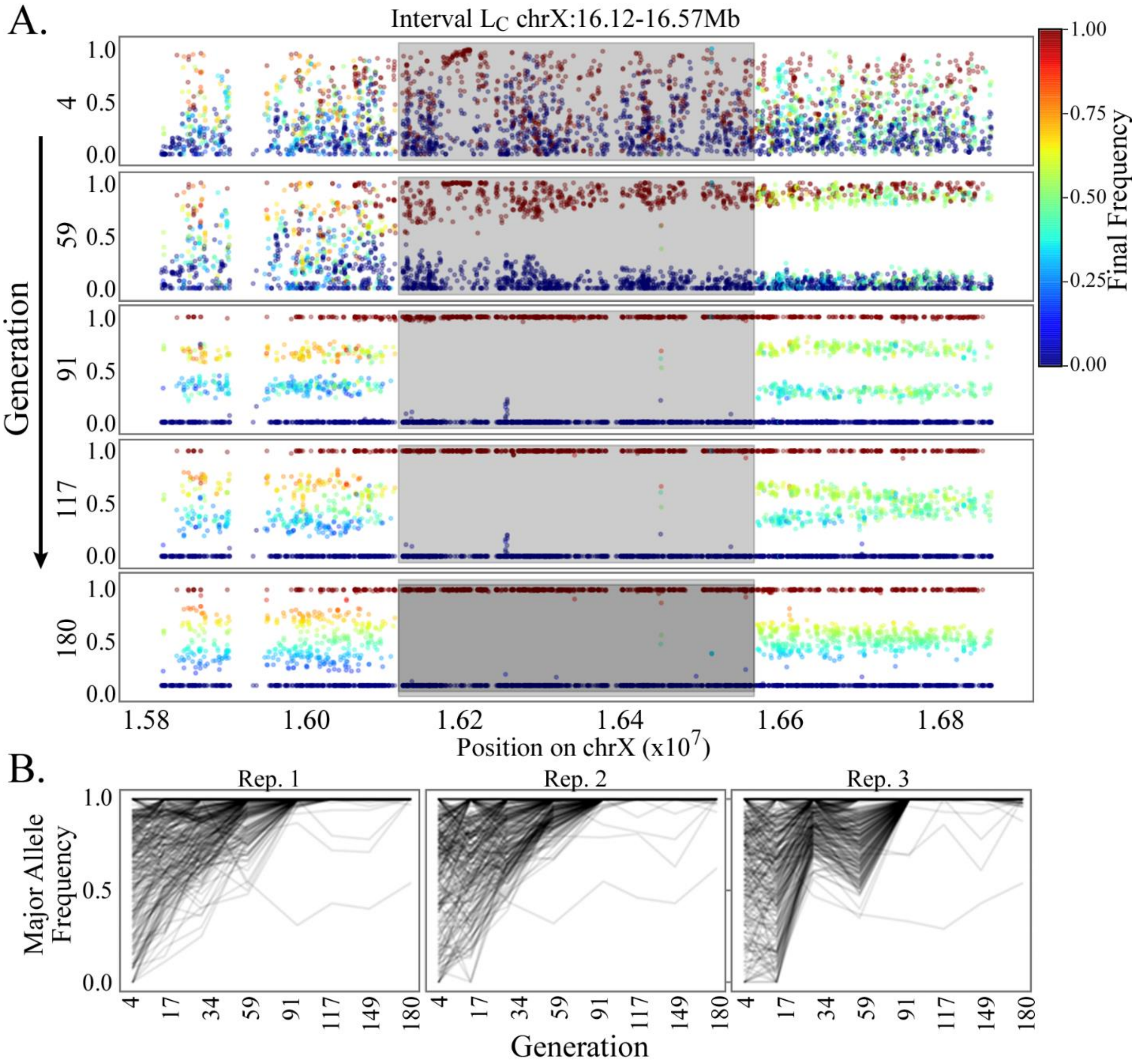

Supplementary Fig. 12. Interval L_C. (A) Spatial and frequency distribution of alleles in interval L_C. The color depicts the frequency at generation 180 of the respective allele. (B) The allele frequency trajectories for the SNPs in interval L_C in each individual replicates. Note the synchronization in replicate chambers.

Supplementary Fig. 13. Interval L_D

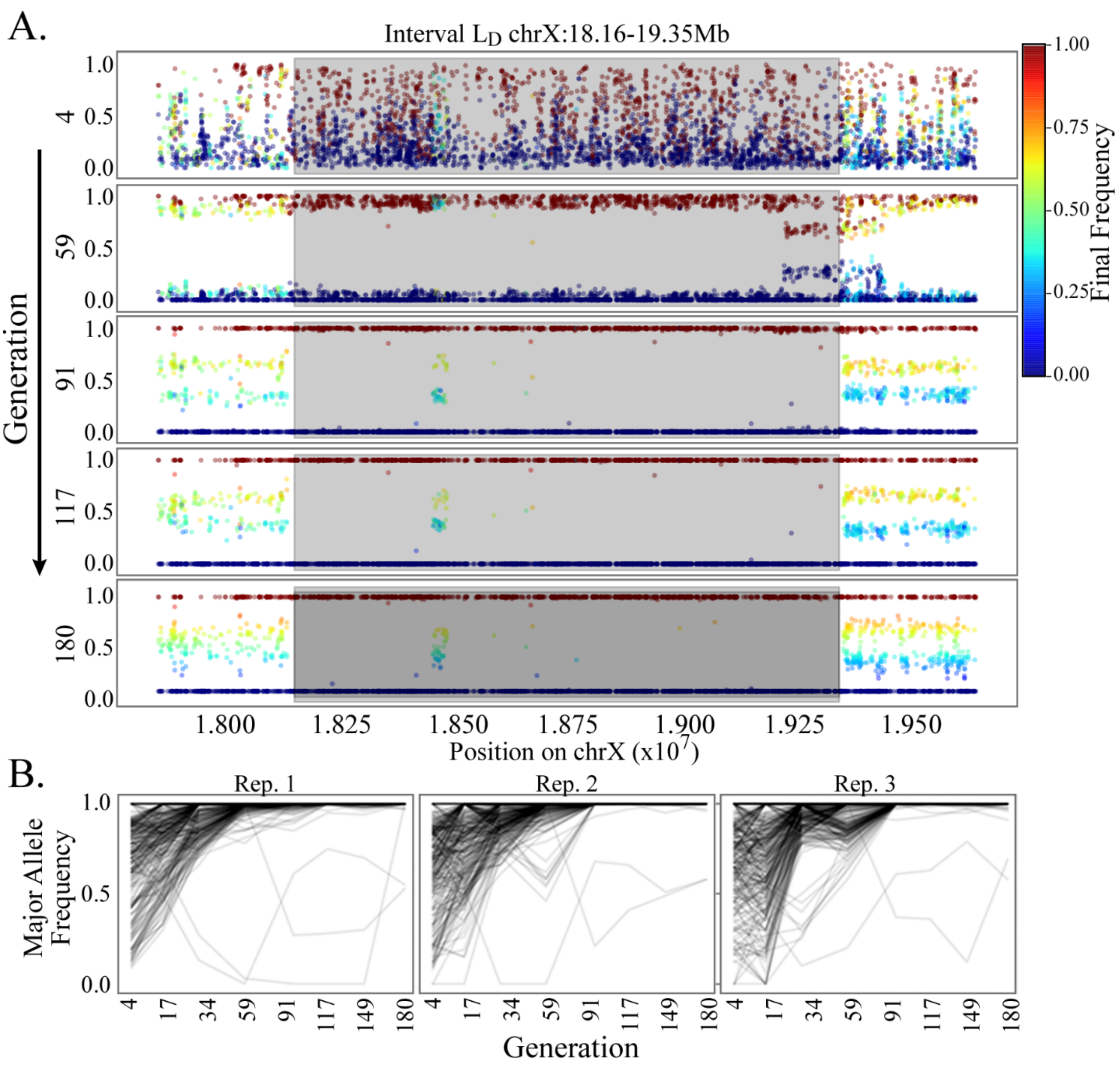

Supplementary Fig. 13. Interval L_D. (A) Spatial and frequency distribution of alleles in interval L_D. The color depicts the frequency at generation 180 of the respective allele. (B) The allele frequency trajectories for the SNPs in interval L_D in each individual replicates. Note the synchronization in replicate chambers.

Supplementary Fig. 14. Interval L_E

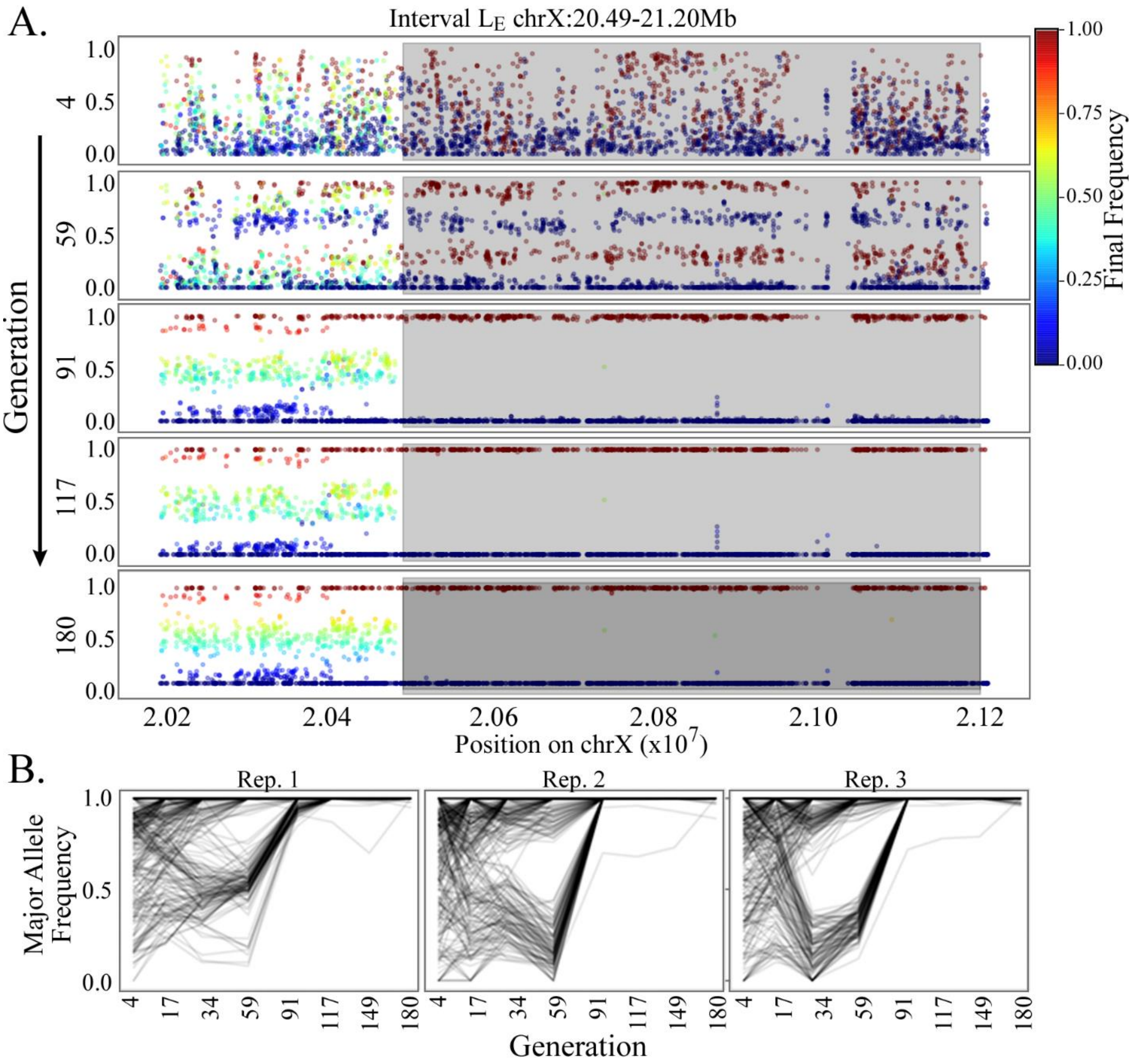

Supplementary Fig. 14. Interval L_E. (A) Spatial and frequency distribution of alleles in interval L_E. The color depicts the frequency at generation 180 of the respective allele. (B) The allele frequency trajectories for the SNPs in interval L_E in each individual replicates. Note the synchronization in replicate chambers.

Supplementary Fig. 15. Interval H_A

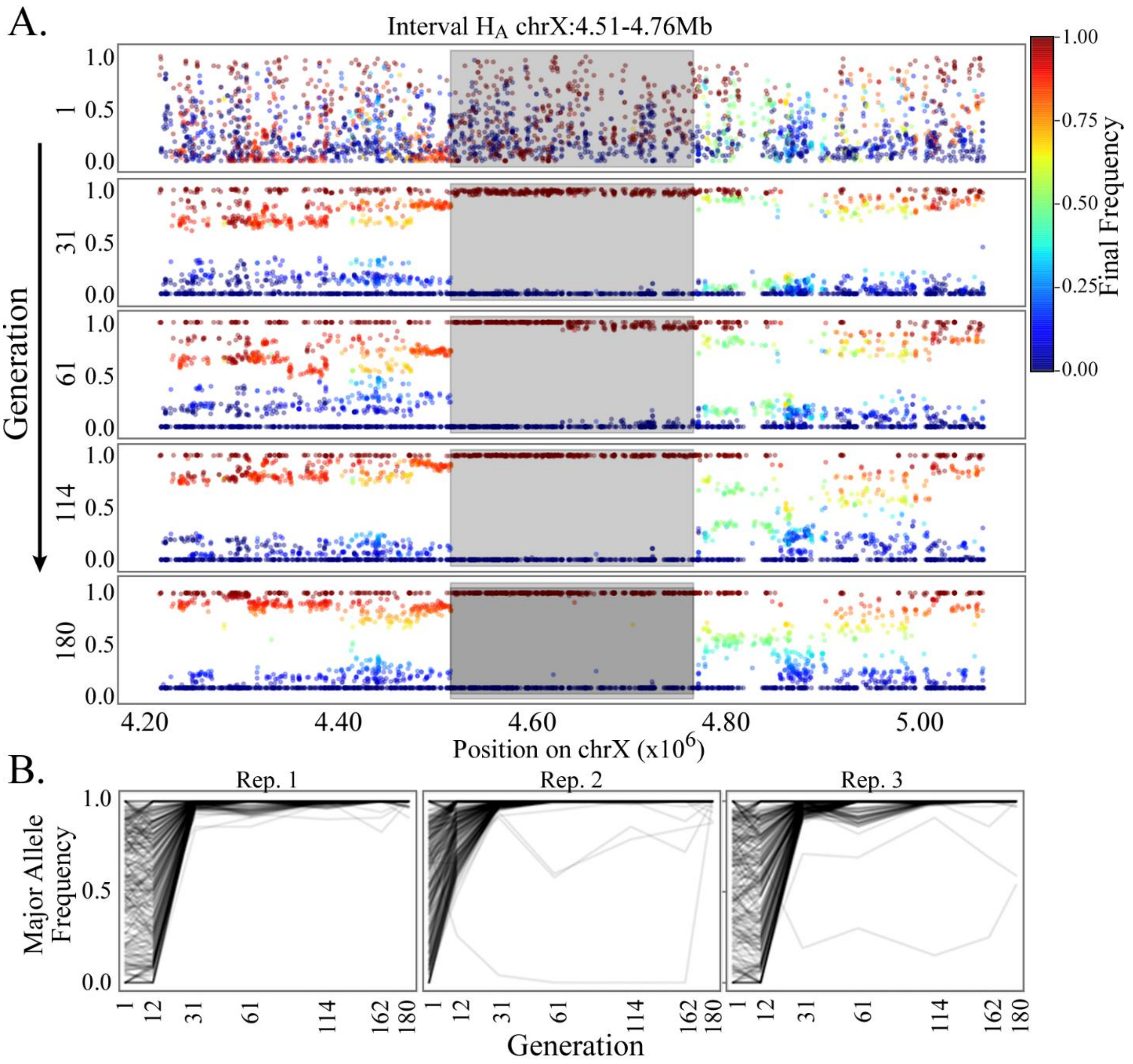

Supplementary Fig. 15. Interval H_A. (A) Spatial and frequency distribution of alleles in interval H_A. The color depicts the frequency at generation 180 of the respective allele. (B) The allele frequency trajectories for the SNPs in interval H_A in each individual replicates. Note the synchronization in replicate chambers.

Supplementary Fig. 16. Interval H_B

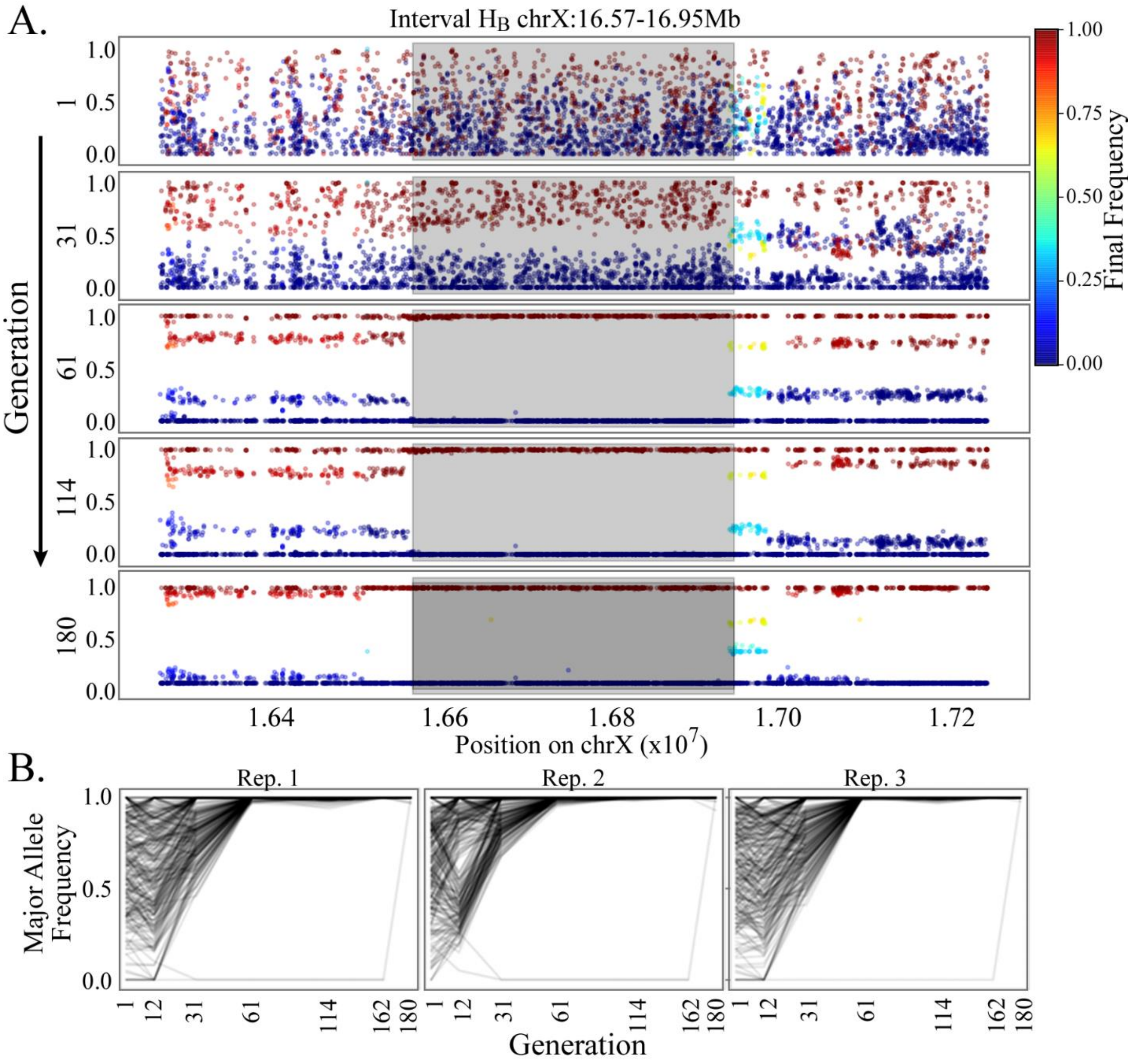

Supplementary Fig. 16. Interval H_B. (A) Spatial and frequency distribution of alleles in interval H_B. The color depicts the frequency at generation 180 of the respective allele. (B) The allele frequency trajectories for the SNPs in interval H_B in each individual replicates. Note the synchronization in replicate chambers.

Supplementary Fig. 17. Interval H_C

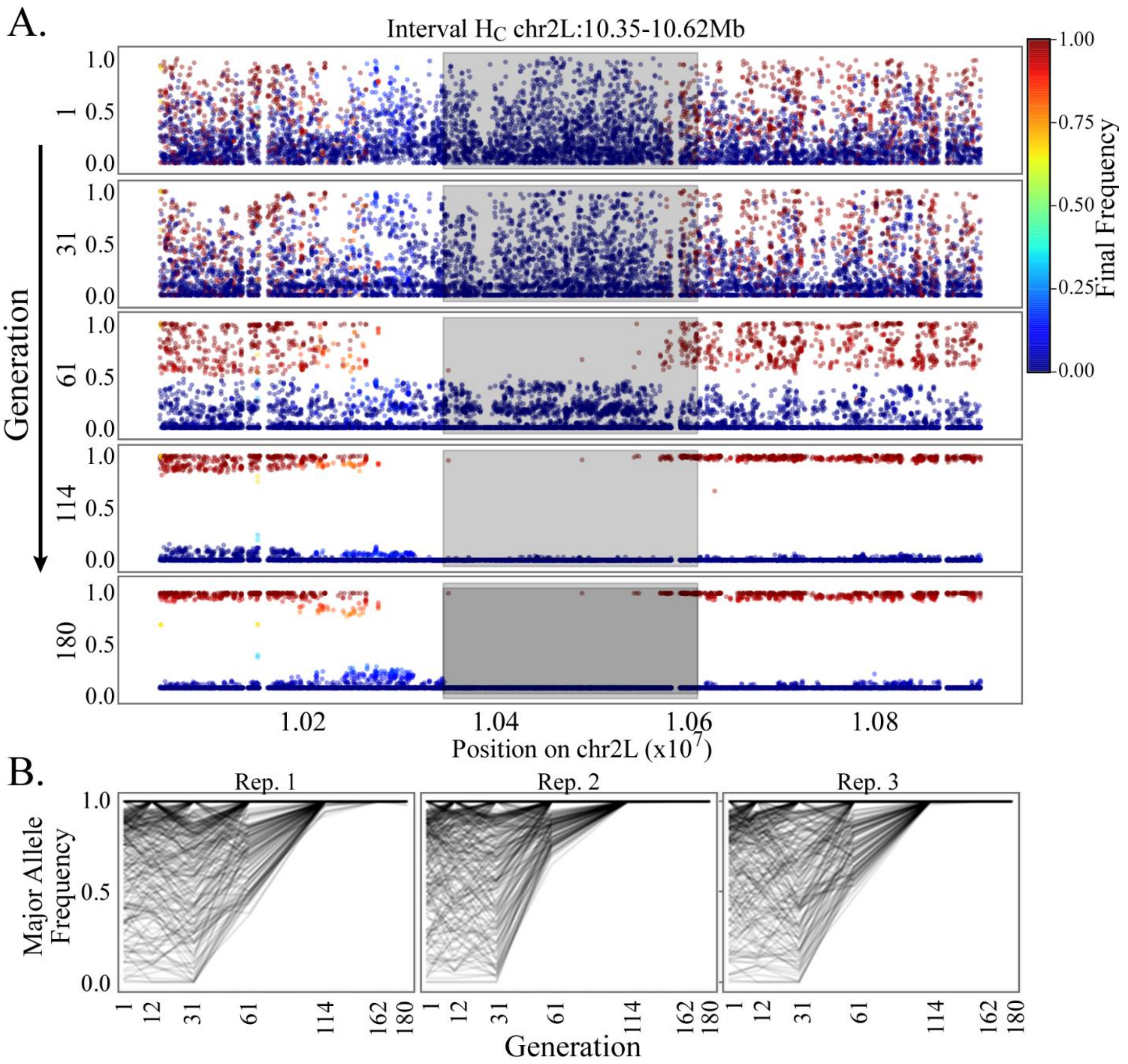

Supplementary Fig. 17. Interval H_C. (A) Spatial and frequency distribution of alleles in interval H_C. The color depicts the frequency at generation 180 of the respective allele. (B) The allele frequency trajectories for the SNPs in interval H_C in each individual replicates. Note the synchronization in replicate chambers.

Supplementary Fig. 18. Interval H_D

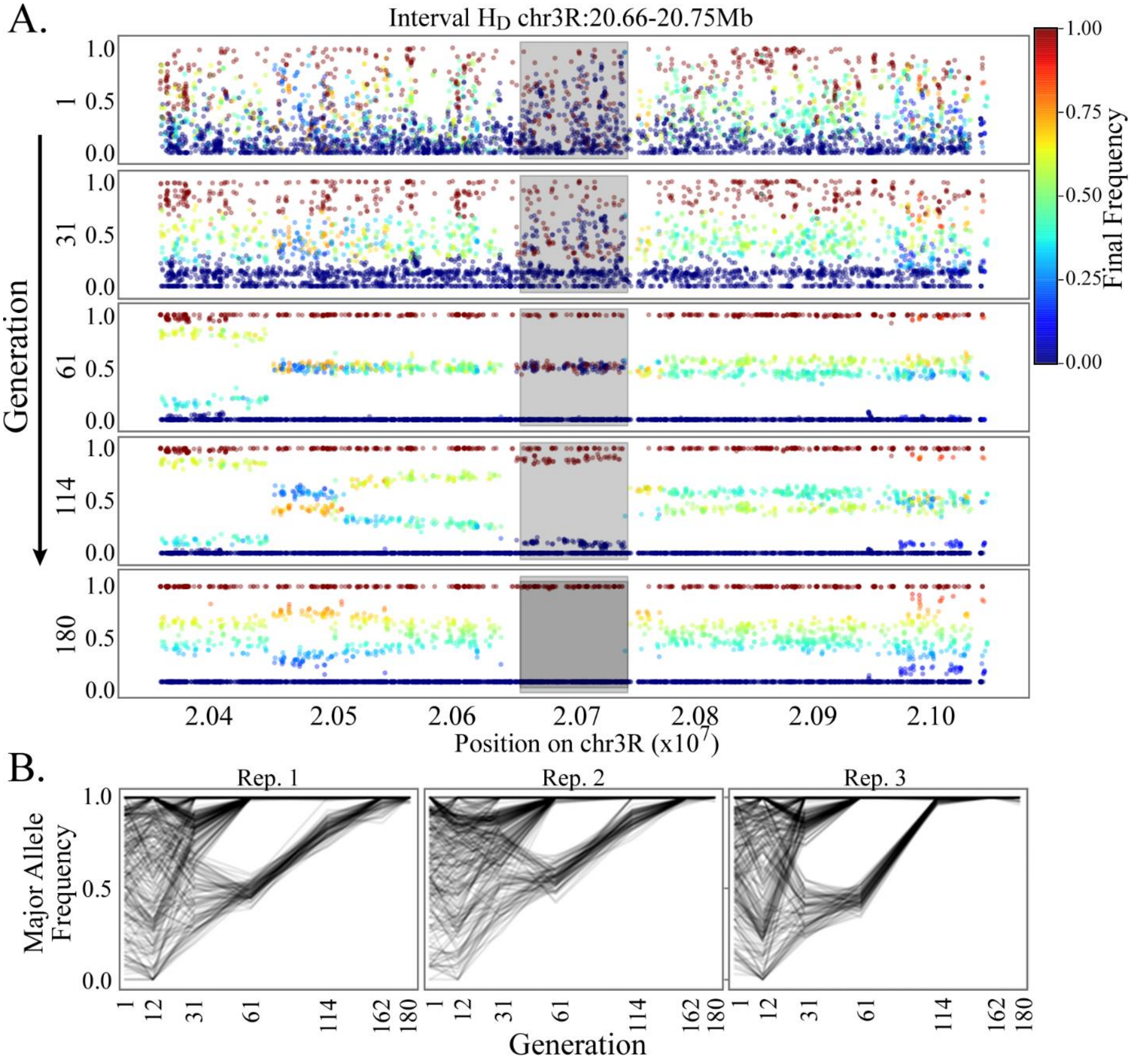

Supplementary Fig. 18. Interval H_D. Top: Spatial and frequency distribution of alleles in interval H_D at different generations. The color depict the final frequency i.e., frequency at 180 generation, of the respective allele. Bottom: The allele frequency trajectories for alleles in interval H_D interval in each replicate. The presence of a haplotype of intermediate frequency at generation 61 while a second haplotype has fixated indicates a soft-sweep on standing variation.

Supplementary Fig. 19. L-Population Individual Sweeps, Replicate 1

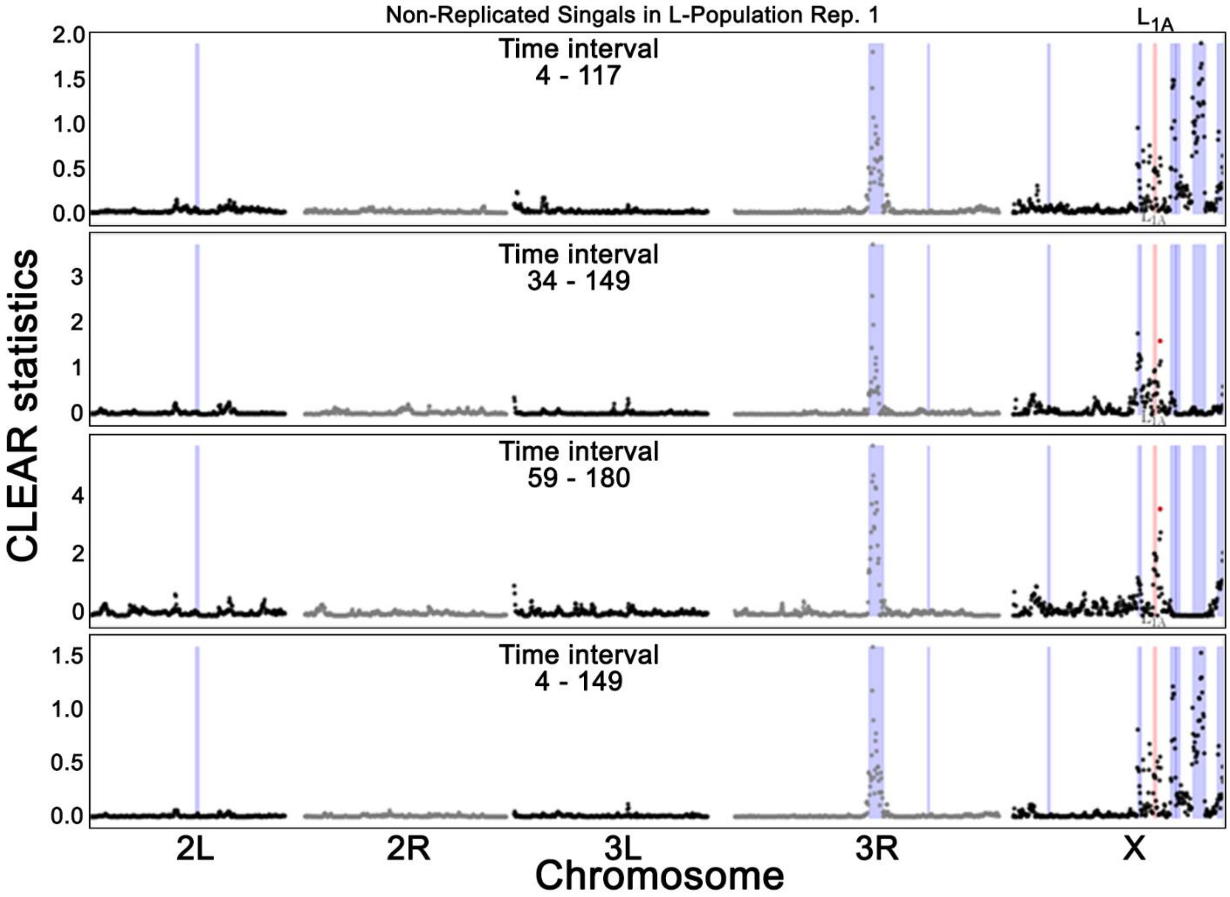

Supplementary Fig. 19. L-Population Individual Sweeps in replicate 1. Individual sweeps i.e., L_{1A}, was detected in only replicate 1 of the L population and was located on chromosome X (red). The blue highlighted intervals are regions constituting replicated sweeps of L_A-L_E depicted in Supplemental Fig. 6 and H_A-H_D depicted in Supplemental Fig. 7.

Supplementary Fig. 20. L-Population Individual Sweeps, Replicate 2

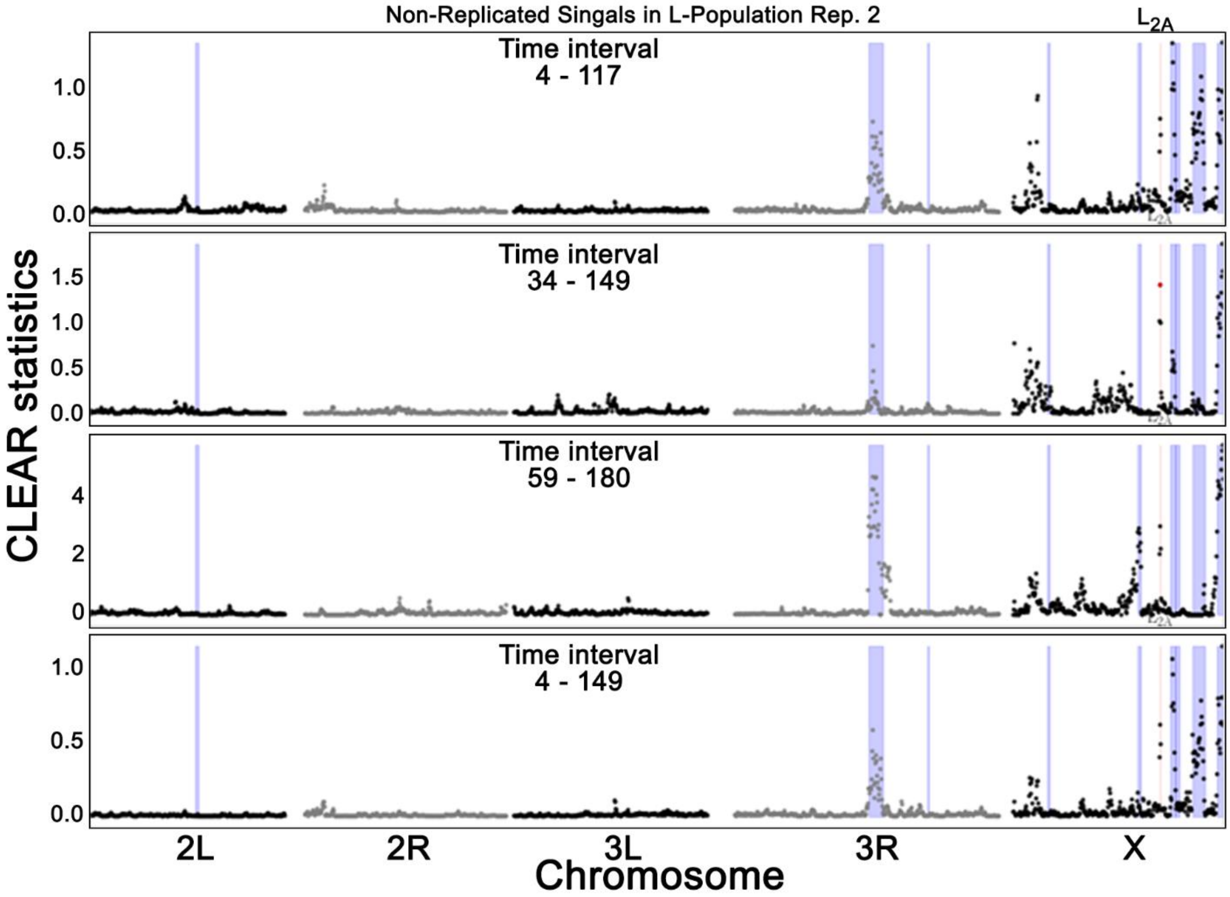

Supplementary Fig. 20. L-Population Individual Sweeps in replicate 2. Single individual sweeps i.e., L_{2A}, was detected in replicate 2 of the L population and was located on chromosome X (red). The blue highlights are regions constituting replicated sweeps in both H_A-H_D and L_A-L_E identified in the H and L-populations. The blue highlighted intervals are regions constituting replicated sweeps of L_A-L_E depicted in Supplemental Fig. 6 and H_A-H_D depicted in Supplemental Fig. 7.

Supplementary Fig. 21. H-Population Individual Sweeps, Replicate 1

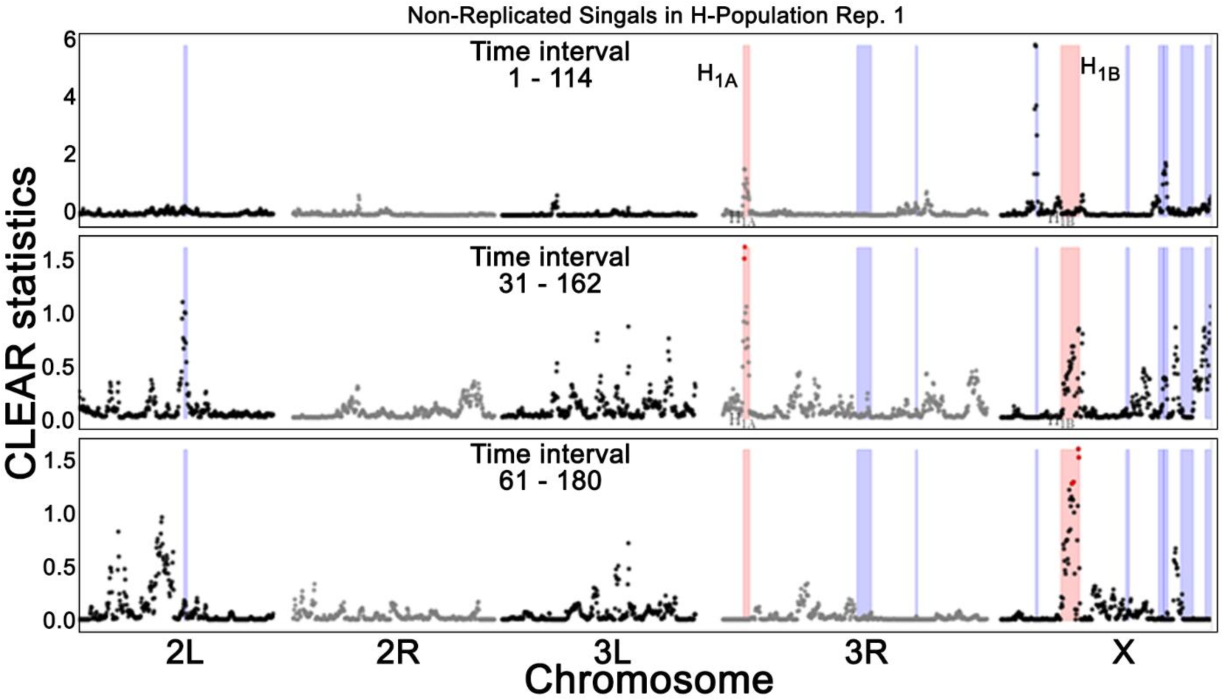

Supplementary Fig. 21. H-Population Individual Sweeps in replicate 1. Two individual sweeps were detected in replicate 1 of the H-population i.e., H_{1A} and H_{1B}. One individual sweep was located on chromosome 3R and other on chromosome X (red). The blue highlighted intervals are regions constituting replicated sweeps of L_A-L_E depicted in Supplemental Fig. 6 and H_A-H_D depicted in Supplemental Fig. 7.

Supplementary Fig. 22. H-Population Individual Sweeps, Replicate 2

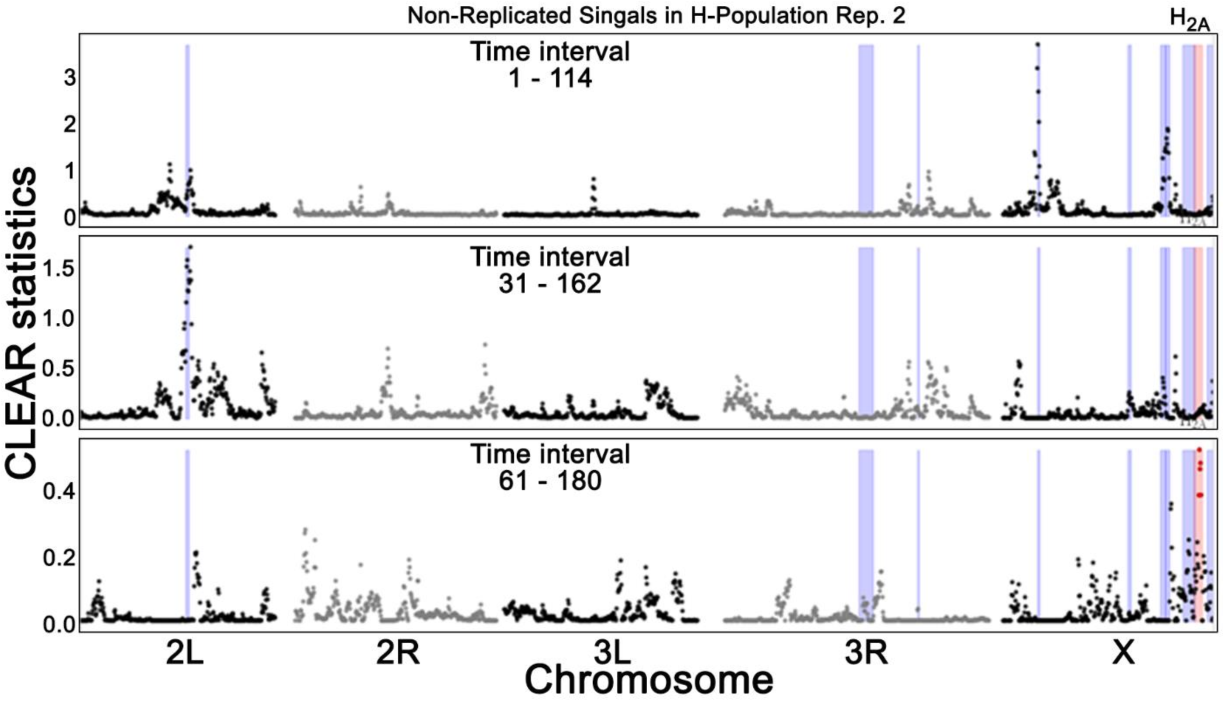

Supplementary Fig. 22. H-Population Individual Sweeps in replicate 2. One individual sweep was detected in replicate 2 of the H-population i.e., H_{2A}. The single individual sweep was located on chromosome X (red). The blue highlighted intervals are regions constituting replicated sweeps of L_A-L_E depicted in Supplemental Fig. 6 and H_A-H_D depicted in Supplemental Fig. 7.

Supplementary Fig. 23. H-Population Individual Sweeps, Replicate 3

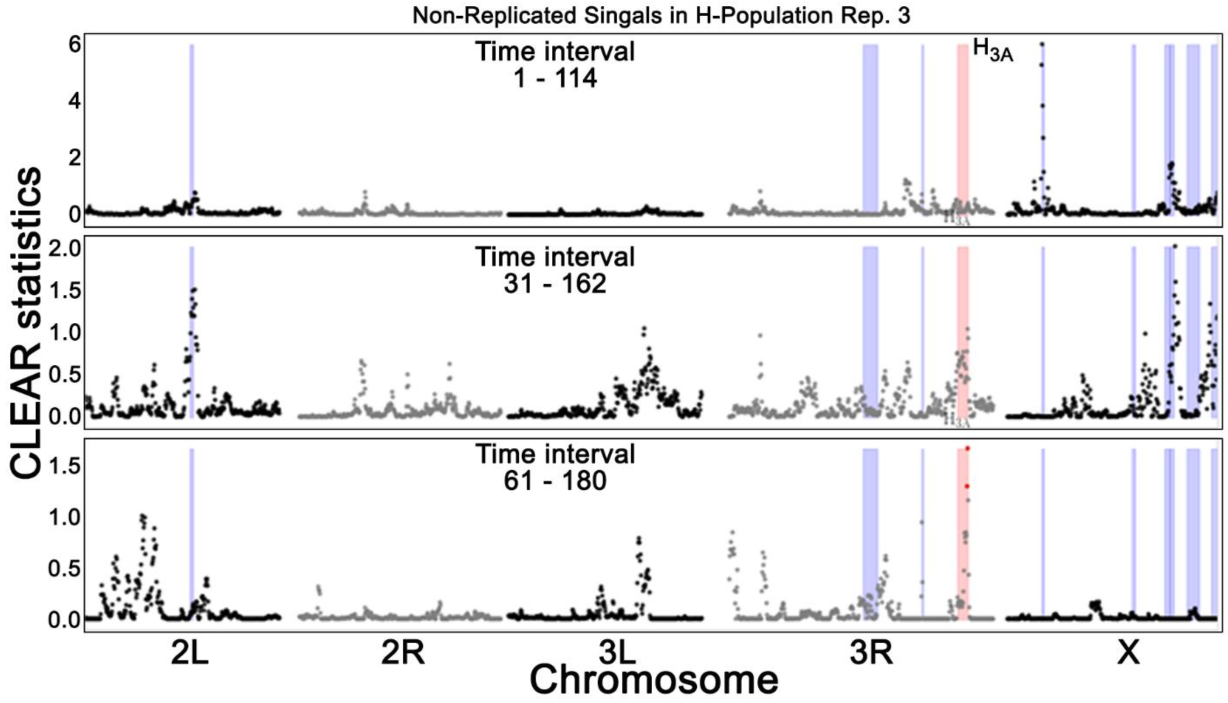

Supplementary Fig. 23. H-Population Individual Sweep in replicate 3. An individual sweep was detected in replicate 3 of the H-population i.e., H3A. The individual sweep was located on chromosome 3R (red). The blue highlighted intervals are regions constituting replicated sweeps of LA-LE depicted in Supplemental Fig. 6 and HA-HD depicted in Supplemental Fig. 7.

Supplementary Fig. 24. Interval L_{1A}

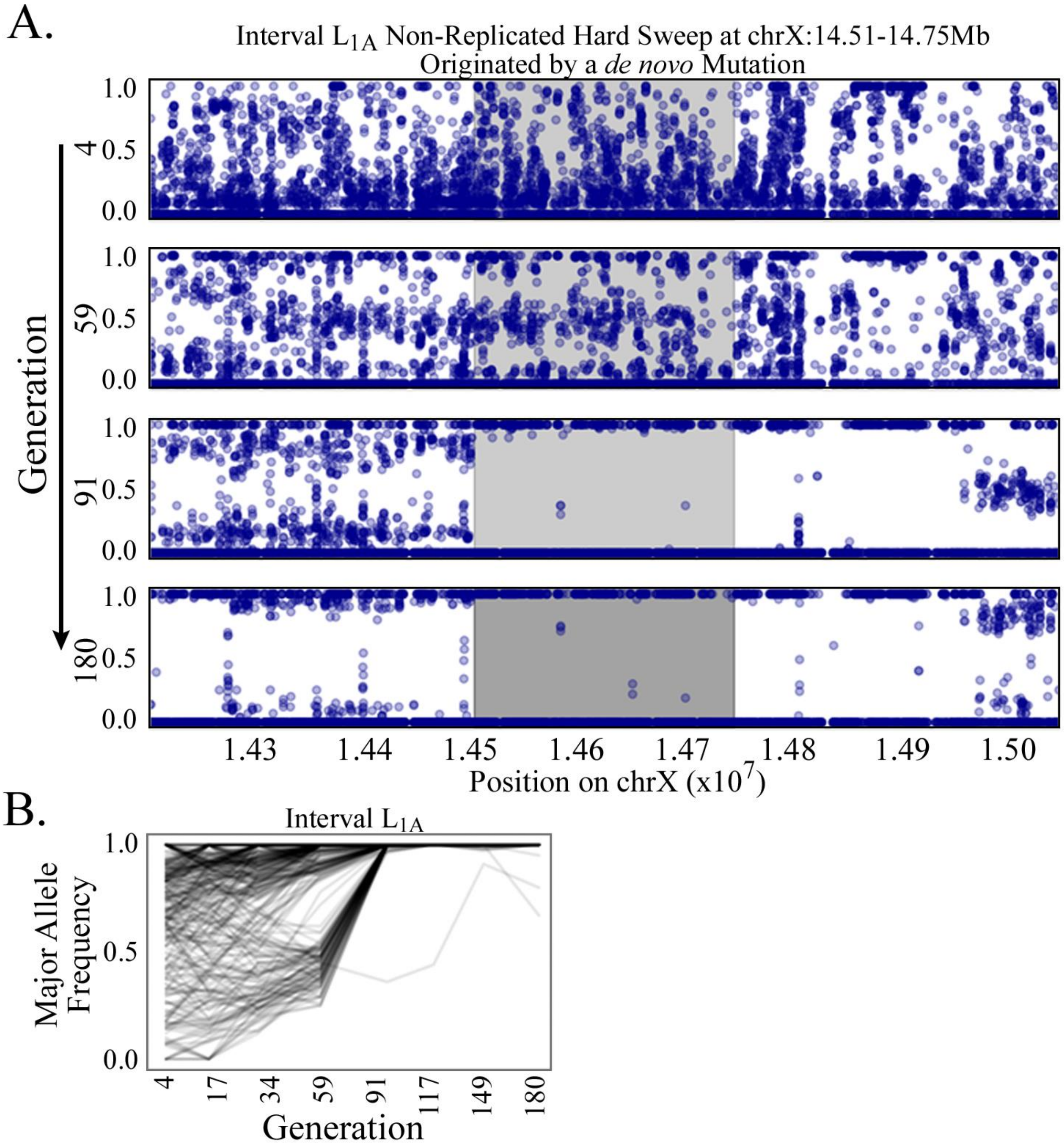

Supplementary Fig. 24. Allele frequency trajectory of interval L_{1A}. (A) Spatial and frequency distribution of alleles in interval L_{1A}, a late individual hard sweep at chrX:14.51-14.75Mb originated by a *de novo* mutation. (B) The trajectory suggests the advent of *de novo* mutation.

Supplementary Fig. 25. Interval L_{2A}

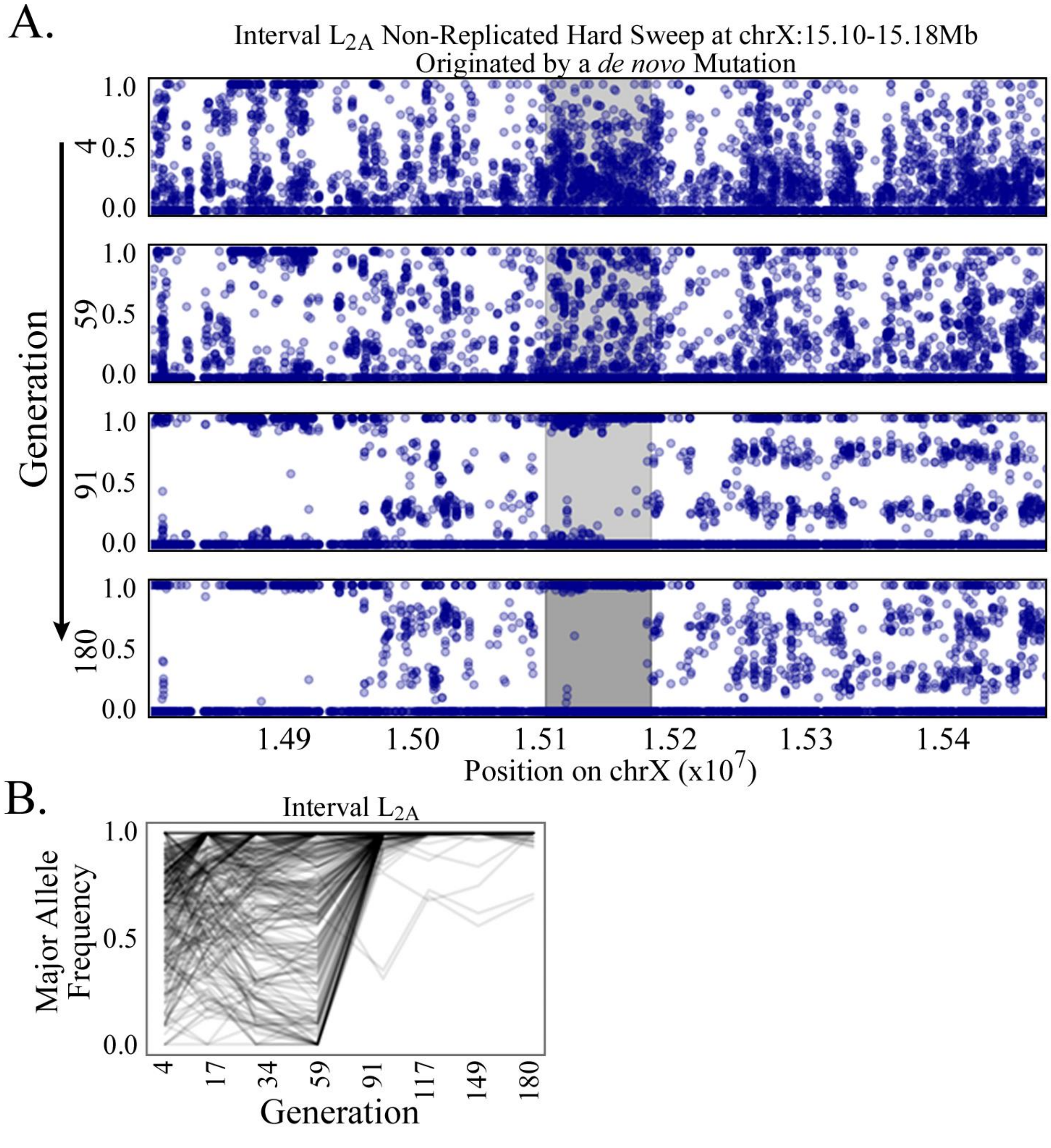

Supplementary Fig. 25. Allele frequency trajectory of interval L_{2A}. (A) Spatial and frequency distribution of alleles in interval L_{2A}, a late individual hard sweep at chrX:15.10-15.18Mb originated by a *de novo* mutation. (B) The trajectory suggests the advent of *de novo* mutation.

Supplementary Fig. 26. Interval H_{1A}

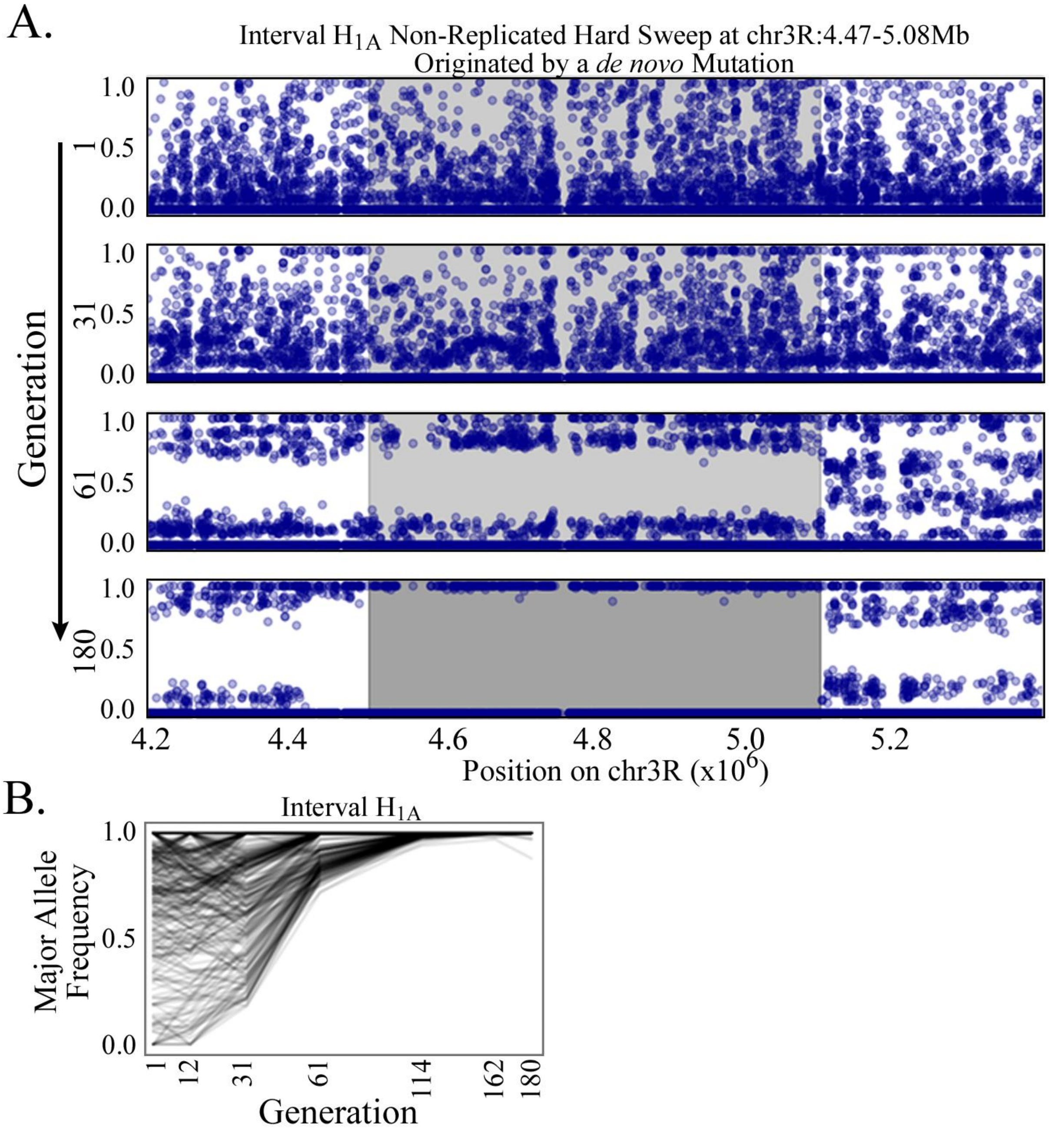

Supplementary Fig. 26. Allele frequency trajectory of interval H_{1A}. (A) Spatial and frequency distribution of alleles in interval H_{1A}, a late individual hard sweep at chr3R:4.47-5.08Mb originated by a *de novo* mutation. (B) The trajectory suggests the advent of *de novo* mutation.

Supplementary Fig. 27. Interval H_{1B}

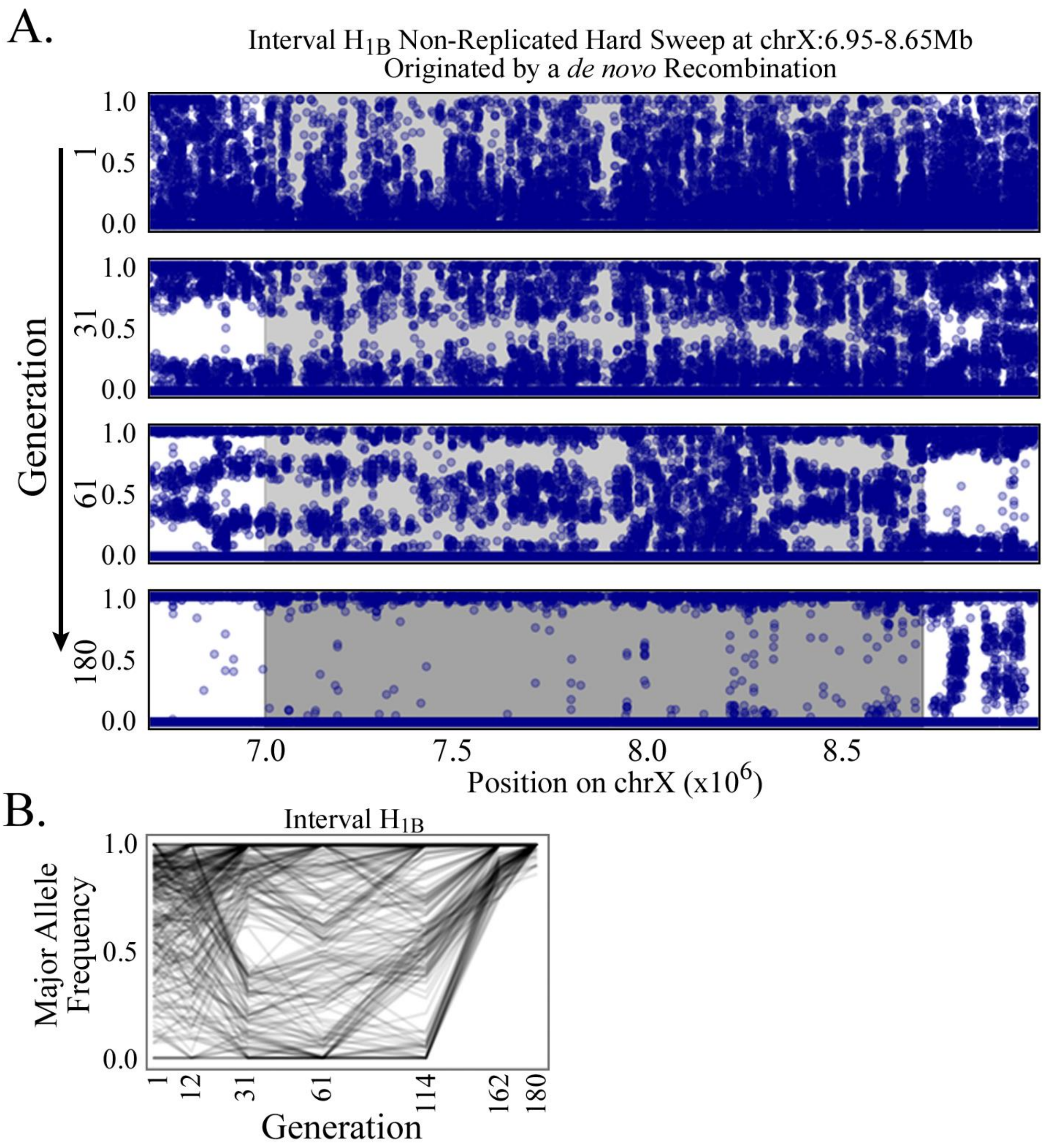

Supplementary Fig. 27. Allele frequency trajectory of interval H_{1B}. (A) Spatial and frequency distribution of alleles in interval H_{1B}, a late individual hard sweep at chrX:6.95-8.65Mb originated by a *de novo* recombination. (B) The trajectory suggests the advent of *de novo* recombination.

Supplementary Fig. 28. Interval H_{2A}

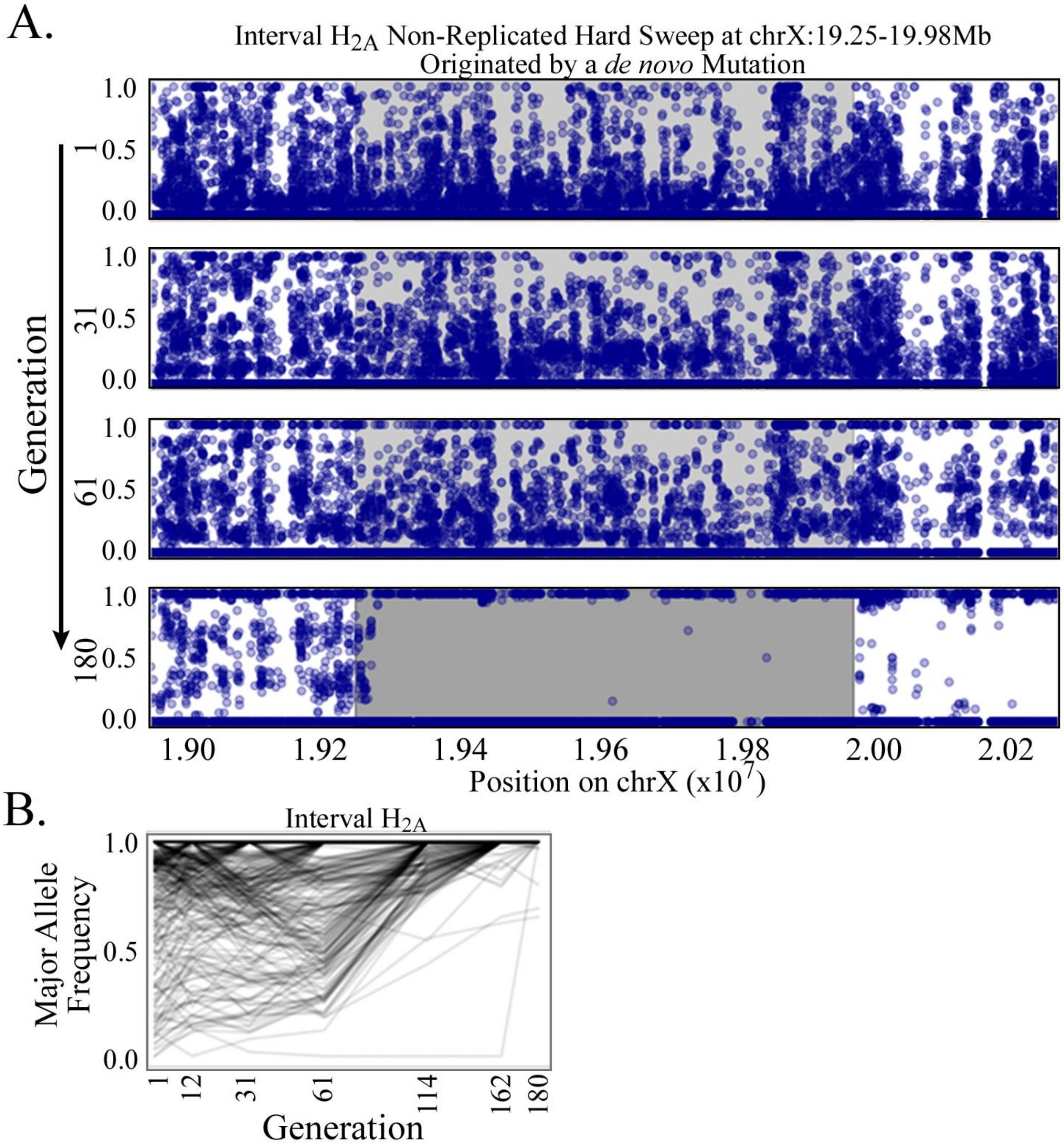

Supplementary Fig. 28. Allele frequency trajectory of interval H_{2A}. (A) Spatial and frequency distribution of alleles in interval H_{2A}, a late individual hard sweep at chrX:19.25-19.98Mb originated by a *de novo* mutation. (B) The trajectory suggests the advent of *de novo* mutation.

Supplementary Fig. 29. Interval H_{3A}

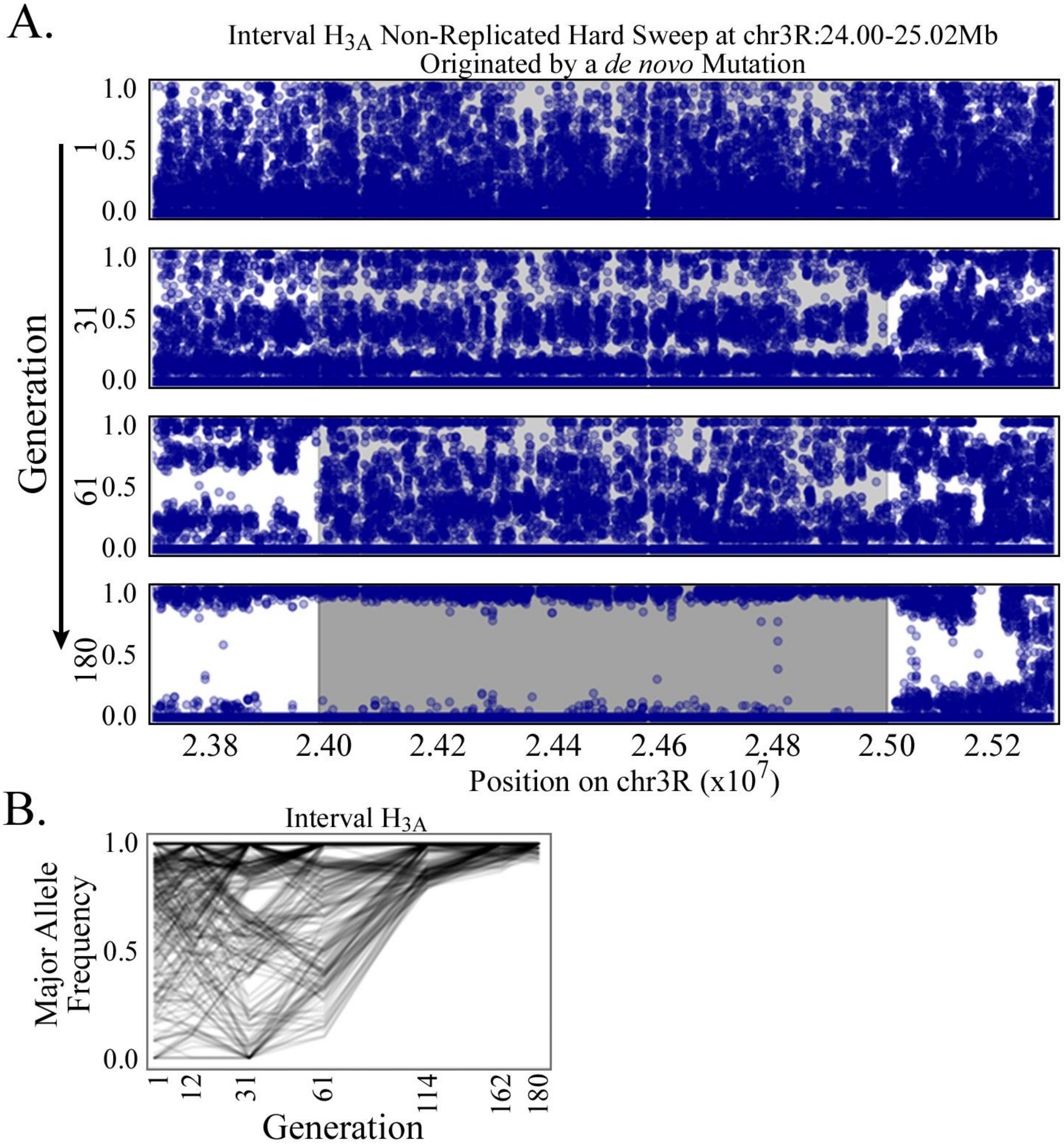

Supplementary Fig. 29. Allele frequency trajectory of interval H_{3A}. (A) Spatial and frequency distribution of alleles in interval H_{3A}, a late individual hard sweep at chr3R:24.00-25.02Mb originated by a *de novo* mutation. (B) The trajectory suggests the advent of *de novo* mutation.

Supplementary Fig. 30. Interval H_{1B}, FM recombination

A.

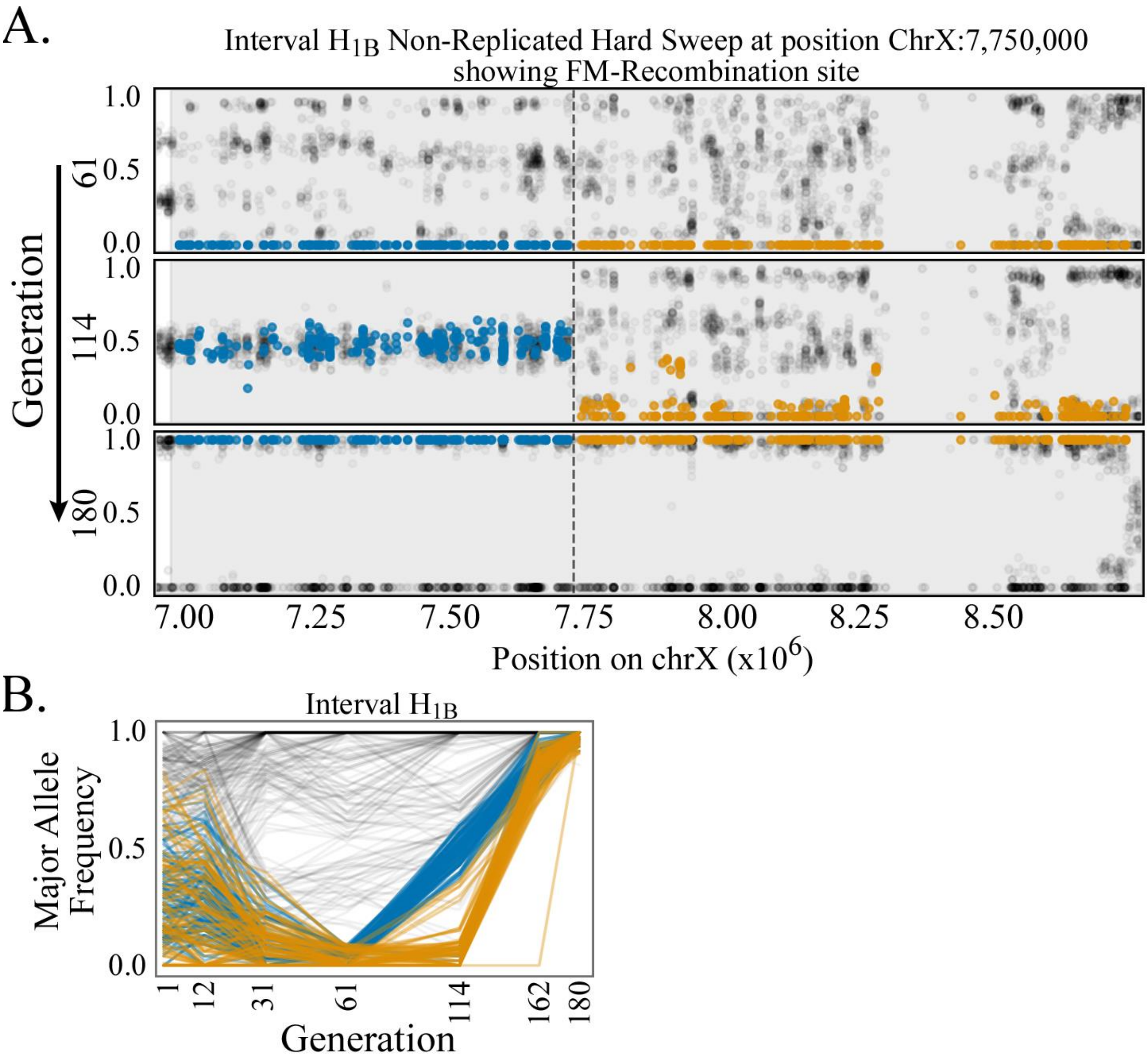

Supplementary Fig. 30. An FM recombination in H_{1B}. (A) A single haplotype with fixation at generation 180 is traced back in time to reveal two distinct clusters (haplotypes) at generation 114, which have two frequencies and are spatially segregated at position 7,750,000 on Chr. X, indicating that they were brought together by a recombination at that position that overcame the Fisher Muller ratchet by combining two beneficial mutations on to a single haplotype. The orange and blue circles depicts major allele frequencies segregated at position 7,750,000 on Chr. X. (B) The trajectory suggests the advent of FM recombination. The blue and orange lines depicts the major allele frequency trajectories on either side of the spatially segregated position 7,750,000 on Chr. X.

Supplementary Fig. 31. Fly eclosion rate at 80% O_2.

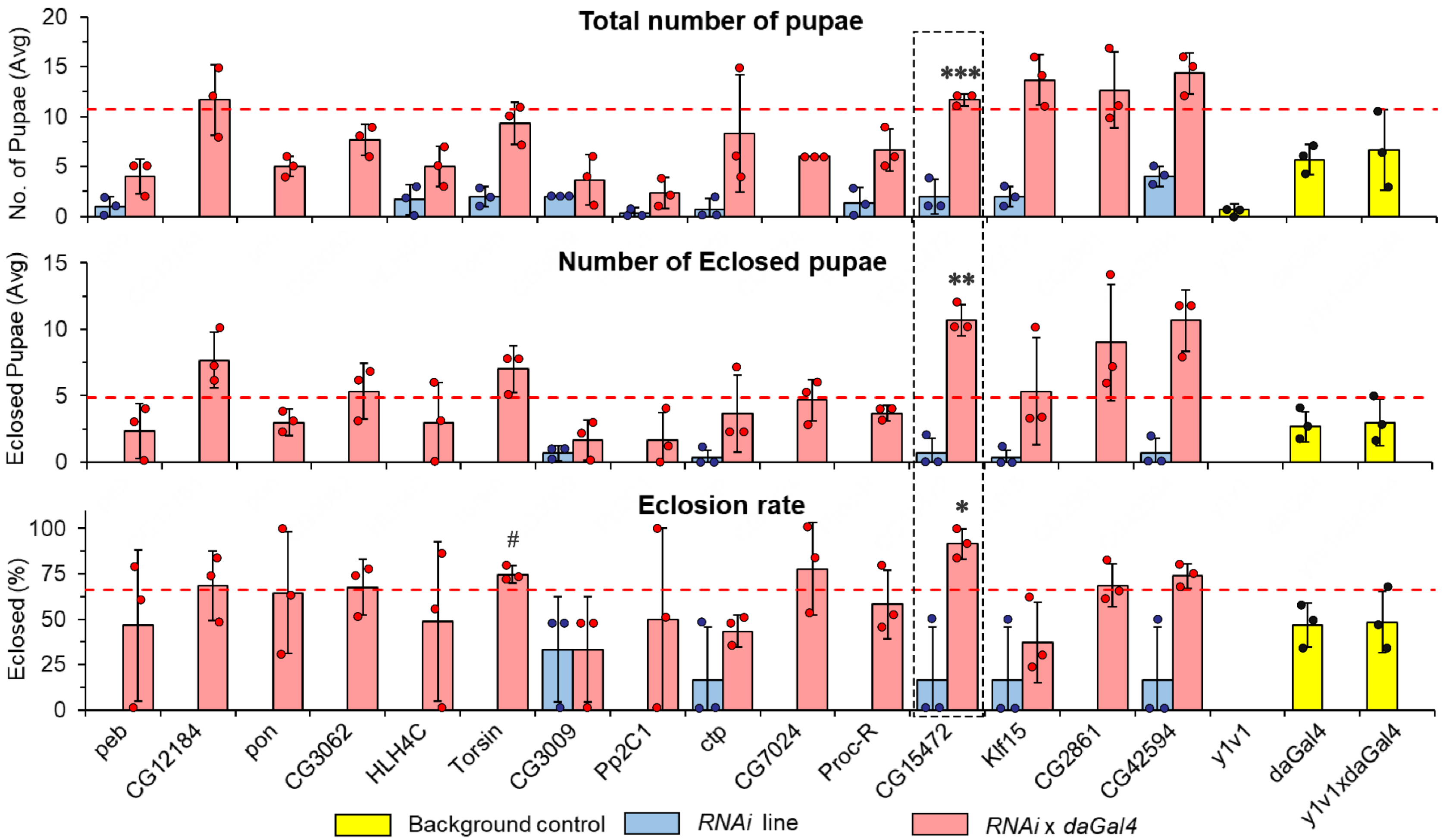

Supplementary Fig. 31. Tolerance test to 80% O_2 level. The total number of pupae (A), number of eclosed pupae (B) and the percentage eclosion rate (C) together indicate fly tolerance. The control flies are indicated in yellow bars. The eclosion rates of the background control *daGal4* line was 46.8 ±12.2% and for y^1v^1 x *daGal4* cross was 48.5 ±16.9%. No eclosion was detected in the y^1v^1

line (0%). The eclosion rate of the H-population was >95%. The eclosion rate of the candidate genes' knockdown of *Torsin* i.e., '*Torsin-RNAi* x *daGal4*' and *CG15472* i.e., '*CG15472-RNAi* x *daGal4*' are significantly higher than the controls (#, '*Torsin-RNAi* x *daGal4*' vs *Torsin-RNAi*, p-value = 0.0013; '*Torsin-RNAi* x *daGal4*' vs y^1v^1, p-value = 0.0446; '*Torsin-RNAi* x *daGal4*' vs *daGal4*, p-value = 0.0446, and *, '*CG15472-RNAi* x *daGal4*' vs *CG15472-RNAi*, p-value = 0.03767; '*CG15472-RNAi* x *daGal4*' vs y^1v^1, p-value = 0.009 and '*CG15472-RNAi* x *daGal4*' vs *daGal4*, p-value = 0.009. However, the eclosion rate was significantly higher only for '*CG15472-RNAi x daGal4*' when compared with background crosses 'y^1v^1 x *daGal4*', p-value = 0.0303. Similarly, the total number of pupae and the total number of eclosed pupae is consistently higher for *CG15472* (***, p-values are 0.006, 1.9992E-05, 0.0124 and 0.1629 and for total number of pupae and **, p-values are 0.0004, 0.0039, 0.001, 0.0049 for '*CG15472-RNAi* x *daGal4*' vs *CG15472-RNAi*, '*CG15472-RNAi* x *daGal4*' vs y^1v^1, '*CG15472-RNAi* x *daGal4*' vs *daGal4*, '*CG15472-RNAi x daGal4*' va 'y^1v^1 x *daGal4*' respectively). Each experiments constitutes n=3 different vials, each vial constiting of 10 males and 10 females (details in methods: High O2 tolerance test section). The error bars represent ± standard deviation. P-value is calculated using two-tail Student's t-Test on excel.

Supplementary Fig. 32. Extensive simulations to capture the time interval of fixation.

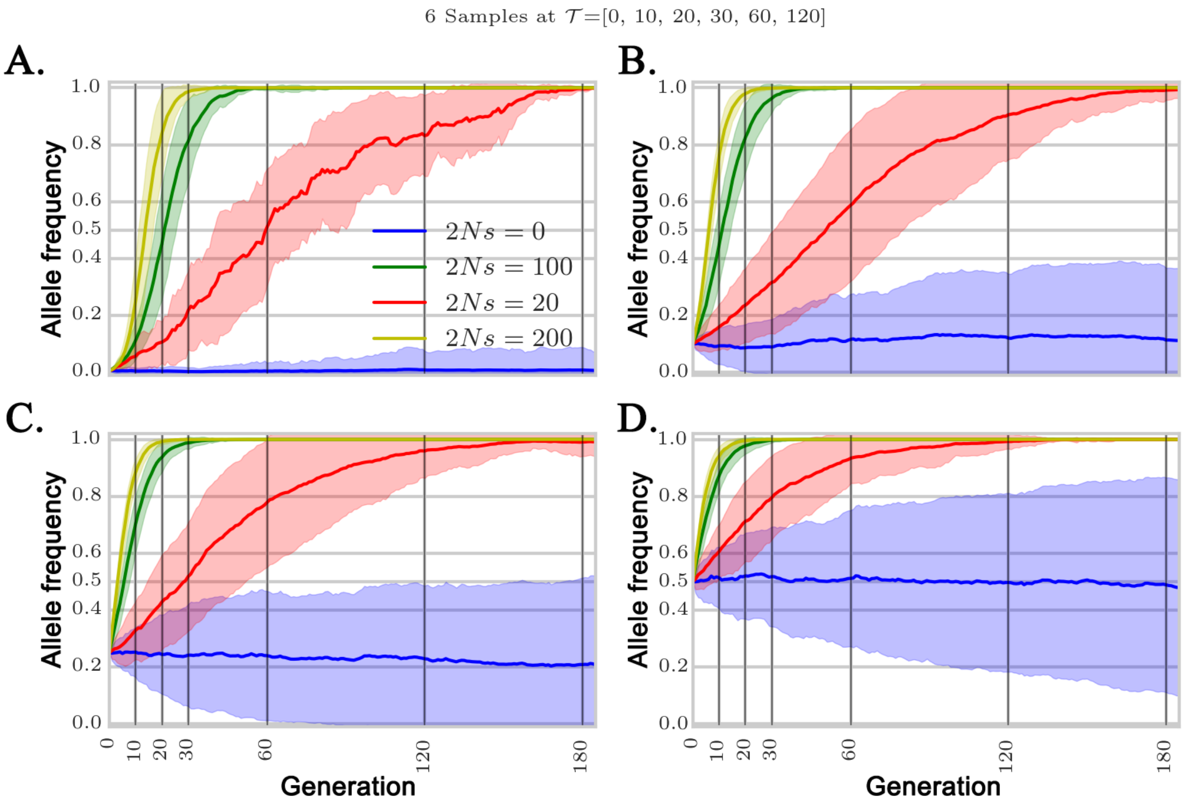

Supplementary Fig. 32. Extensive simulations to capture the time interval of fixation. Trajectories (*N*=100) were simulated to capture the time interval of fixation for multiple scenarios including genetic drift (2*Ns*=0), weak selection (2*Ns* = 20), moderate selection (2*Ns* = 100) and strong selection (2*Ns* = 200). Solid line and shaded area represent the mean and 95% confidence intervals based on 100 simulations. Six sampling times were chosen so that dynamics of selection could be capture for multiple initial allele frequencies of 0.01 (A), 0.1 (B), 0.25 (C), 0.5 (D).

Supplementary Fig. 33. Confounding signal of CLEAR: Lack of Final Fixation.

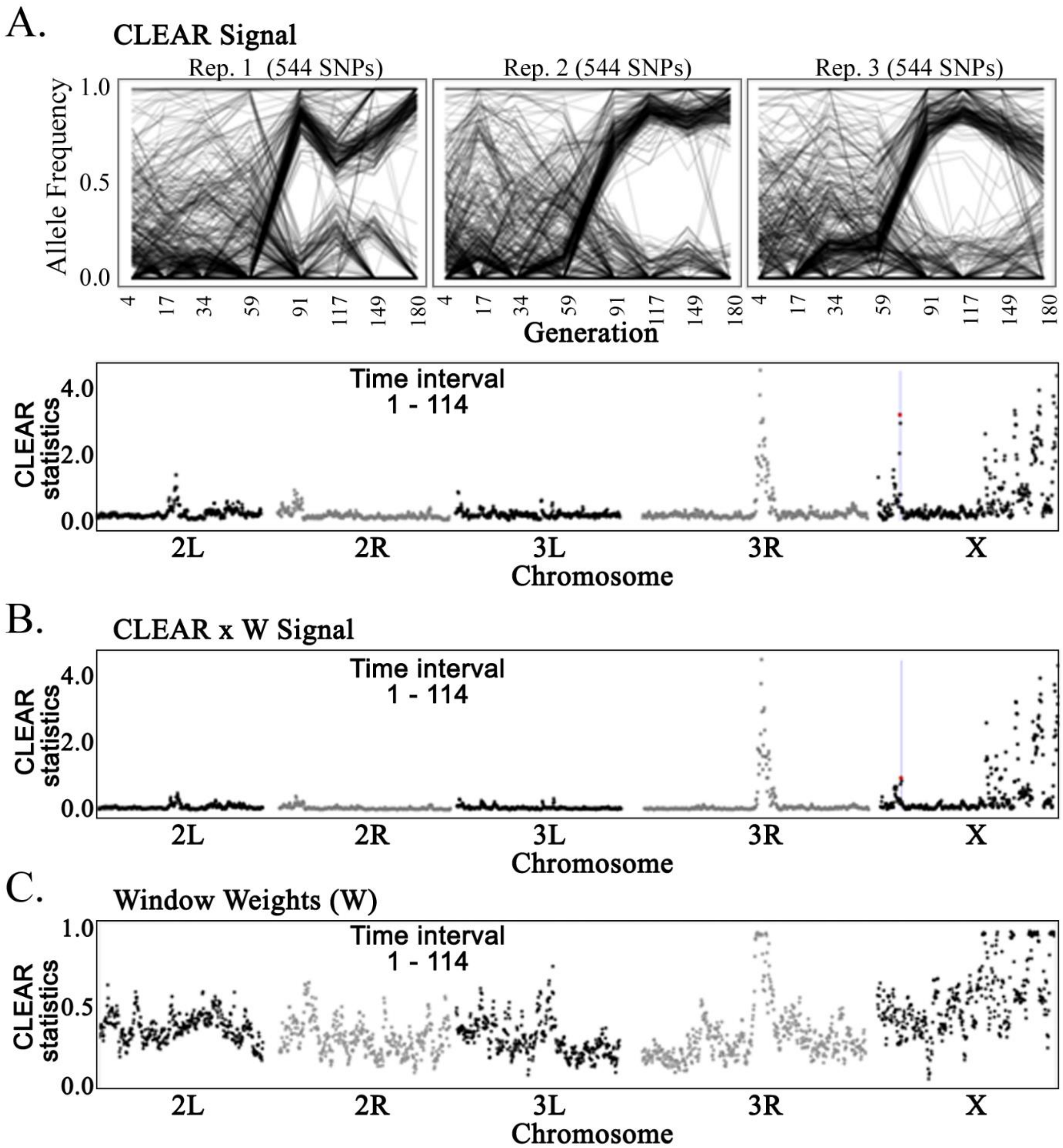

Supplementary Fig. 33. Confounding of CLEAR depicting a lack of final fixation. (A) A false positive example resulted from high CLEAR score up to 117th generation. However, most of the mutations drifted away from fixation suggesting that this was a false positive signal. (B) Weighted CLEAR statistic. (C) Weight of each window.

Supplementary Fig. 34. Late Sweep.

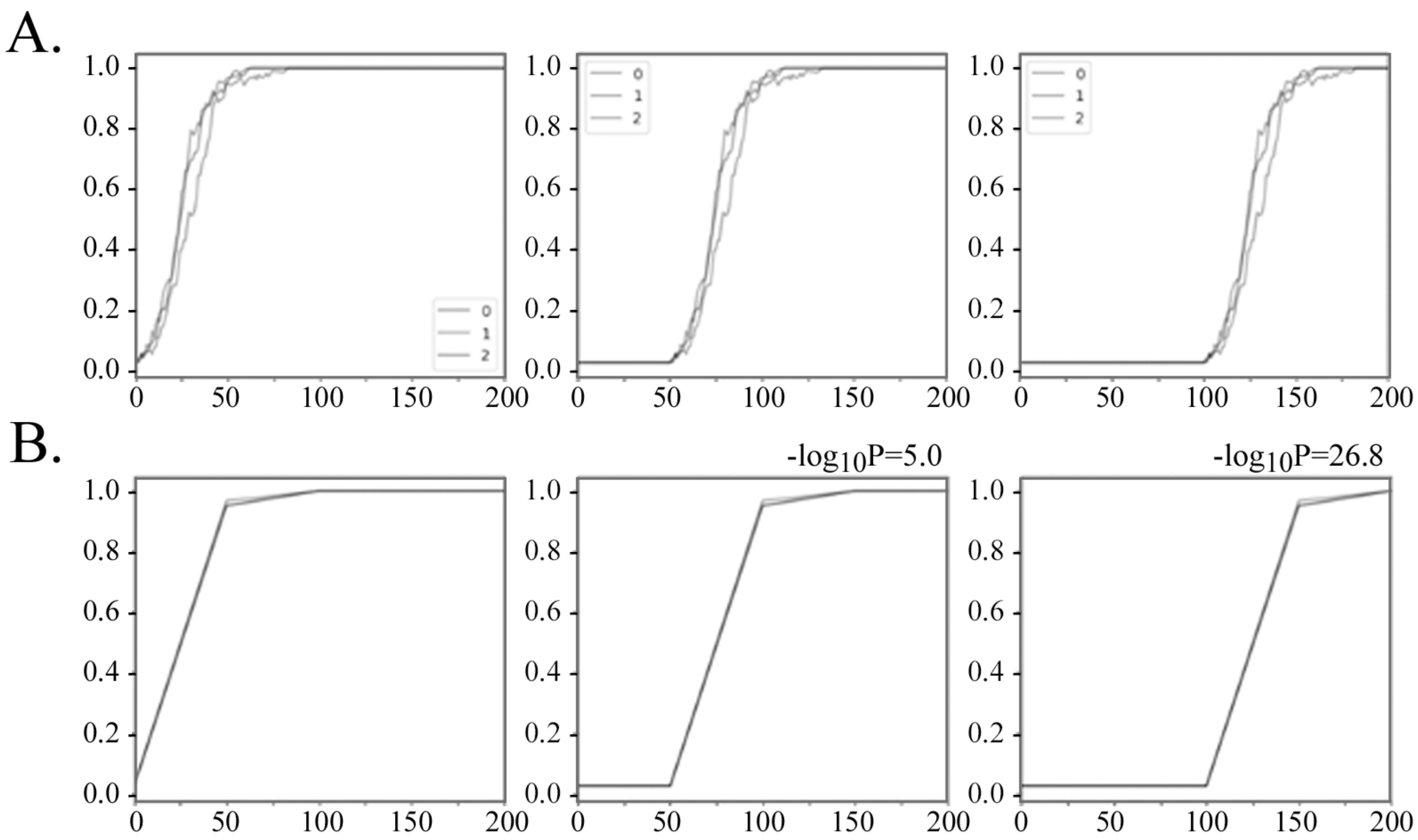

Supplementary Fig. 34: Late sweep. (A) Trajectory of a selective sweep starting at generation 0, 50 and 100. (B) Trajectory of a selective sweep starting at generations 0, 50 and 100 sampled every 50 generations. In each case, the fixation event happens within 25 generations for the specific sweep, and the Z-statistic test rejects the null hypothesis of an early sweep for sweeps occurring at generations 50 (P<1E-5) and 100 (P<1E-26.8).

Supplementary Fig. 35. Interval H_D , Standing Variation at the Time of Fixation.

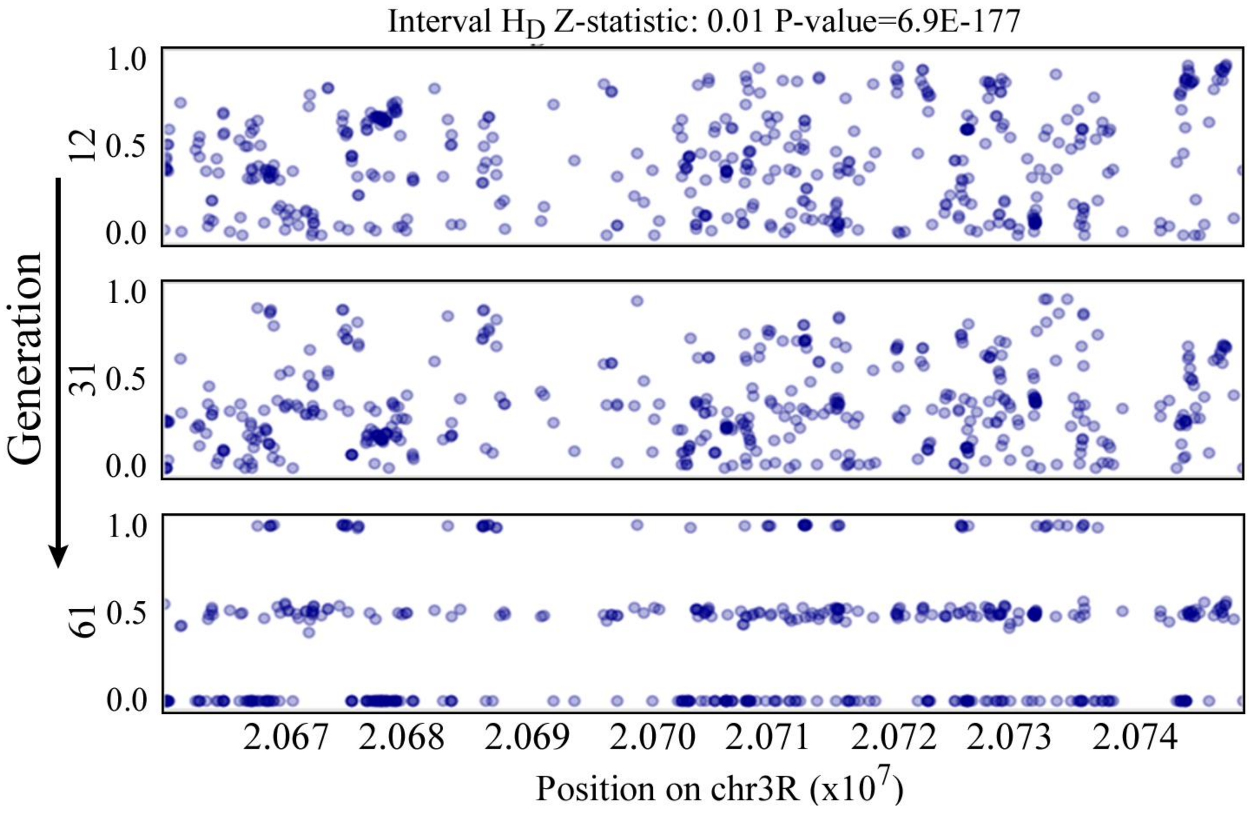

Supplementary Fig. 35. Interval H_D depicting uniformity of standing variations of soft sweep at the time of fixation. Soft sweeps, characterized by haplotypes at intermediate frequencies, can be confounded by flanking regions of hard-sweep. However, uniformity of intermediate frequencies in the entirety of the region can conclusively identify the region as undergoing a soft-sweep. Z-statistic P-value=6.9E-177.

Supplementary Table 1. List of genes within the selected intervals and their human orthologs

A) Genes within the L-intervals

Interval	Gene	FBID_KEY	ANNOTATION_SYMBOL	NAME	SYMBOL	GO_BIOLOGICAL_PROCESS	GO_CELLULAR_COMPONENT	GO_MOLECULAR_FUNCTION	H_SAPIENS_ORTHOLOGS	LOCATION_ARM
L1	Dl	FBgn0000463	CG3619	Delta	Dl	mesoderm development ; GO:0007498 | inferred from mutant phenotype <newline> antennal morphogenesis ; GO:0048800 | inferred from mutant phenotype <newline> stem cell differentiation ; GO:0048863 | inferred from mutant phenotype <newline> imaginal disc-derived wing margin morphogenesis ; GO:0008587 | inferred from mutant phenotype <newline> Notch signaling pathway ; GO:0007219 | inferred from genetic interaction with N <newline> ovarian follicle cell stalk formation ; GO:0030713 | inferred from mutant phenotype <newline> dorsal/ventral lineage restriction, imaginal disc ; GO:0007451 | traceable author statement <newline> Notch signaling pathway ; GO:0007219 | inferred from mutant phenotype <newline> Notch signaling pathway ; GO:0007219 | inferred from genetic interaction with dx <newline> oocyte localization involved in germarium-derived egg [illegible]	adherens junction ; GO:0005912 | inferred from direct assay <newline> cytoplasmic vesicle ; GO:0031410 | inferred from direct assay <newline> plasma membrane ; GO:0005886 | inferred from direct assay <newline> cell surface ; GO:0009986 | inferred from direct assay <newline> integral component of membrane ; GO:0016021 | inferred from direct assay <newline> cytoplasmic vesicle ; GO:0031410 | colocalizes_with inferred from direct assay <newline> endosome ; GO:0005768 | inferred from direct assay <newline> plasma membrane ; GO:0005886 | inferred from biological aspect of ancestor with PANTHER:PTN002372732 <newline> apical cortex ; GO:0045179 | inferred from direct assay <newline> endocytic vesicle ; GO:0030139 | inferred from direct assay <newline> subapical complex [illegible]	calcium ion binding ; GO:0005509 | inferred from electronic annotation with InterPro:IPR001881, InterPro:IPR018097 <newline> receptor ligand activity ; GO:0048018 | inferred from direct assay <newline> Notch binding ; GO:0005112 | inferred from direct assay <newline> Notch binding ; GO:0005112 | inferred from biological aspect of ancestor with PANTHER:PTN002371879 <newline> protein binding ; GO:0005515 | inferred from physical interaction with UniProtKB:Q9VUX2 <newline> Notch binding ; GO:0005112 | inferred from physical interaction with N <newline> glycosphingolipid binding ; GO:0043208 | inferred from direct assay	Hsap\NOTCH4 <newline> Hsap\DLL1 <newline> Hsap\JAG2 <newline> Hsap\DLL3 <newline> Hsap\JAG1 <newline> Hsap\DLL4	3R
L1	CG43203	FBgn0262839	CG43203	-	CG43203	biological_process ; GO:0008150 | no biological data available	cellular_component ; GO:0005575 | no biological data available	molecular_function ; GO:0003674 | no biological data available	-	3R

L1	Ino80	FBgn0086613	CG31212	INO80 complex subunit	Ino80	DNA repair ; GO:0006281 | inferred from biological aspect of ancestor with PANTHER:PTN001521025 <newline> ATP-dependent chromatin remodeling ; GO:0043044 | inferred from biological aspect of ancestor with PANTHER:PTN002904112 <newline> regulation of gene expression ; GO:0010468 | inferred from mutant phenotype <newline> regulation of transcription from RNA polymerase II promoter in response to stress ; GO:0043618 | inferred from biological aspect of ancestor with PANTHER:PTN001521025 <newline> chromatin remodeling ; GO:0006338 | inferred from biological aspect of ancestor with PANTHER:PTN002904112 <newline> regulation of development, heterochronic ; GO:0040034 | inferred from mutant phenotype <newline> negative regulation of transcription, DNA-templated ; GO:0045892 | inferred from mutant phenotype <newline> nucleosome [illegible]	nucleus ; GO:0005634 | inferred from direct assay <newline> polytene chromosome ; GO:0005700 | inferred from direct assay <newline> Ino80 complex ; GO:0031011 | inferred from direct assay <newline> nuclear chromatin ; GO:0000790 | inferred from direct assay <newline> nucleus ; GO:0005634 | inferred from high throughput direct assay <newline> Ino80 complex ; GO:0031011 | inferred from biological aspect of ancestor with PANTHER:PTN001521025	ATP binding ; GO:0005524 | inferred from electronic annotation with InterPro:IPR000330 <newline> DNA-dependent ATPase activity ; GO:0008094 | inferred from sequence or structural similarity with UniProtKB:Q9ULG1 <newline> DNA binding ; GO:0003677 | inferred from sequence or structural similarity with UniProtKB:Q9ULG1 <newline> transcription regulatory region DNA binding ; GO:0044212 | inferred from direct assay <newline> ATPase activity ; GO:0016887 | inferred from biological aspect of ancestor with PANTHER:PTN002904112 <newline> protein binding ; GO:0005515 | inferred from physical interaction with UniProtKB:Q8ST83 <newline> histone binding ; GO:0042393 | inferred from biological aspect of ancestor with PANTHER:PTN002904112	Hsap\INO80	3R
L1	CG3581	FBgn0038697	CG3581	-	CG3581	-	cellular_component ; GO:0005575 | no biological data available	molecular_function ; GO:0003674 | no biological data available	-	3R
L1	CG31404	FBgn0051404	CG31404	-	CG31404	biological_process ; GO:0008150 | no biological data available	cellular_component ; GO:0005575 | no biological data available	molecular_function ; GO:0003674 | no biological data available	-	3R
L1	CG31245	FBgn0051245	CG31245	-	CG31245	-	-	-	-	3R
L1	CG3734	FBgn0038700	CG3734	-	CG3734	proteolysis ; GO:0006508 | inferred from biological aspect of ancestor with PANTHER:PTN000110021	-	serine-type peptidase activity ; GO:0008236 | inferred from electronic annotation with InterPro:IPR008758 <newline> dipeptidyl-peptidase activity ; GO:0008239 | inferred from biological aspect of ancestor with PANTHER:PTN000110021	Hsap\PRSS16 <newline> Hsap\DPP7	3R
L1	CG18493	FBgn0038701	CG18493	-	CG18493	proteolysis ; GO:0006508 | inferred from biological aspect of ancestor with PANTHER:PTN000110021	-	serine-type peptidase activity ; GO:0008236 | inferred from electronic annotation with InterPro:IPR008758 <newline> dipeptidyl-peptidase activity ; GO:0008239 | inferred from biological aspect of ancestor with PANTHER:PTN000110021	Hsap\PRSS16	3R

L1	CG3739	FBgn0038702	CG3739	-	CG3739	proteolysis ; GO:0006508 | inferred from biological aspect of ancestor with PANTHER:PTN000110021	-	serine-type peptidase activity ; GO:0008236 | inferred from electronic annotation with InterPro:IPR008758 <newline> dipeptidyl-peptidase activity ; GO:0008239 | inferred from biological aspect of ancestor with PANTHER:PTN000110021	Hsap\PRSS16	3R
L1	CG31244	FBgn0051244	CG31244	-	CG31244	biological_process ; GO:0008150 | no biological data available	mitochondrion ; GO:0005739 | inferred from biological aspect of ancestor with PANTHER:PTN000283242	protein phosphatase 1 binding ; GO:0008157 | inferred from physical interaction with flw inferred from physical interaction with Pp1-13C inferred from physical interaction with Pp1α-96A inferred from physical interaction with Pp1-87B	Hsap\INO80 <newline> Hsap\TACO1	3R
L1	CG5316	FBgn0038704	CG5316	-	CG5316	single strand break repair ; GO:0000012 | inferred from biological aspect of ancestor with PANTHER:PTN000281062 <newline> single strand break repair ; GO:0000012 | inferred from sequence or structural similarity with UniProtKB:Q7Z2E3-1 <newline> mRNA splicing, via spliceosome ; GO:0000398 | inferred by curator from GO:0071011 <newline> double-strand break repair ; GO:0006302 | inferred from biological aspect of ancestor with PANTHER:PTN000281062	nucleus ; GO:0005634 | inferred from biological aspect of ancestor with PANTHER:PTN000281062 <newline> precatalytic spliceosome ; GO:0071011 | inferred from high throughput direct assay	single-strand break-containing DNA binding ; GO:1990165 | inferred from biological aspect of ancestor with PANTHER:PTN000281062 <newline> double-stranded RNA binding ; GO:0003725 | inferred from biological aspect of ancestor with PANTHER:PTN000281062 <newline> single-stranded DNA binding ; GO:0003697 | inferred from biological aspect of ancestor with PANTHER:PTN000281062 <newline> damaged DNA binding ; GO:0003684 | inferred from sequence or structural similarity with UniProtKB:Q7Z2E3 <newline> DNA 5'-adenosine monophosphate hydrolase activity ; GO:0033699 | inferred from biological aspect of ancestor with PANTHER:PTN000281062 <newline> mismatched DNA binding ; GO:0030983 | inferred from biological aspect of ancestor with PANTHER:PTN000281062	Hsap\PNKP <newline> Hsap\APTX	3R
L1	CG11626	FBgn0038705	CG11626	-	CG11626	proteolysis ; GO:0006508 | inferred from biological aspect of ancestor with PANTHER:PTN000110021	-	serine-type peptidase activity ; GO:0008236 | inferred from electronic annotation with InterPro:IPR008758 <newline> dipeptidyl-peptidase activity ; GO:0008239 | inferred from biological aspect of ancestor with PANTHER:PTN000110021	Hsap\PRSS16 <newline> Hsap\DPP7	3R

L1	CG31221	FBgn0051221	CG31221	-	CG31221	-	-	-	Hsap\LRP1B	3R
L1	CG3517	FBgn0038706	CG3517	-	CG3517	biological_process ; GO:0008150 | no biological data available	cellular_component ; GO:0005575 | no biological data available	molecular_function ; GO:0003674 | no biological data available	-	3R
L1	CG6255	FBgn0038708	CG6255	Succinyl-coenzyme A synthetase alpha subunit 2	Scsalpha2	succinyl-CoA metabolic process ; GO:0006104 | inferred from biological aspect of ancestor with PANTHER:PTN000122012 <newline> tricarboxylic acid cycle ; GO:0006099 | inferred from biological aspect of ancestor with PANTHER:PTN000122012 <newline> nucleoside triphosphate biosynthetic process ; GO:0009142 | inferred from biological aspect of ancestor with PANTHER:PTN000122012 <newline> succinate metabolic process ; GO:0006105 | inferred from biological aspect of ancestor with PANTHER:PTN000122012	cytosol ; GO:0005829 | inferred from biological aspect of ancestor with PANTHER:PTN000122012 <newline> mitochondrion ; GO:0005739 | inferred from biological aspect of ancestor with PANTHER:PTN000122014	cofactor binding ; GO:0048037 | inferred from electronic annotation with InterPro:IPR003781 <newline> succinate-CoA ligase (ADP-forming) activity ; GO:0004775 | inferred from biological aspect of ancestor with PANTHER:PTN000122012 <newline> nucleoside diphosphate kinase activity ; GO:0004550 | inferred from biological aspect of ancestor with PANTHER:PTN000122012	Hsap\SUCLG1	3R
L1	Dys	FBgn0260003	CG34157	Dystrophin	Dys	imaginal disc-derived wing vein morphogenesis ; GO:0008586 | inferred from mutant phenotype <newline> muscle cell cellular homeostasis ; GO:0046716 | inferred from mutant phenotype <newline> regulation of neurotransmitter secretion ; GO:0046928 | inferred from direct assay <newline> imaginal disc-derived wing vein specification ; GO:0007474 | inferred from mutant phenotype <newline> regulation of short-term neuronal synaptic plasticity ; GO:0048172 | inferred from direct assay <newline> establishment of cell polarity ; GO:0030010 | inferred from mutant phenotype <newline> regulation of neurotransmitter secretion ; GO:0046928 | inferred from mutant phenotype <newline> neuromuscular synaptic transmission ; GO:0007274 | inferred from direct assay <newline> muscle organ development ; GO:0007517 | [illegible]	dystrophin-associated glycoprotein complex ; GO:0016010 | inferred from physical interaction with Dg <newline> cytoskeleton ; GO:0005856 | inferred from direct assay <newline> cytoplasm ; GO:0005737 | inferred from high throughput direct assay <newline> dystrophin-associated glycoprotein complex ; GO:0016010 | inferred from sequence or structural similarity with HGNC:12635 inferred from sequence or structural similarity with HGNC:2928 inferred from sequence or structural similarity with HGNC:3032 <newline> cell cortex ; GO:0005938 | inferred from high throughput direct assay <newline> synapse ; GO:0045202 | inferred from direct assay <newline> cytoplasm ; GO:0005737 | inferred from direct assay	zinc ion binding ; GO:0008270 | inferred from electronic annotation with InterPro:IPR000433 <newline> WW domain binding ; GO:0050699 | inferred from physical interaction with Dg <newline> structural constituent of muscle ; GO:0008307 | inferred from sequence or structural similarity with HGNC:12635 inferred from sequence or structural similarity with HGNC:2928 inferred from sequence or structural similarity with HGNC:3032 <newline> actin binding ; GO:0003779 | inferred from sequence or structural similarity <newline> protein binding ; GO:0005515 | inferred from physical interaction with UniProtKB:A1ZA89	Hsap\DMD <newline> Hsap\SPTBN1 <newline> Hsap\ACTN1 <newline> Hsap\SPTBN2 <newline> Hsap\SPTB <newline> Hsap\ACTN3 <newline> Hsap\DRP2 <newline> Hsap\UTRN <newline> Hsap\SPTBN4 <newline> Hsap\ACTN2 <newline> Hsap\ACTN4	3R
L1	CG15025	FBgn0038709	CG15025	-	CG15025	-	-	-	-	3R

L1	Cpr92A	FBgn0038714	CG6240	Cuticular protein 92A	Cpr92A	chitin-based cuticle development ; GO:0040003 | inferred from sequence model	extracellular matrix ; GO:0031012 | inferred from biological aspect of ancestor with PANTHER:PTN000955699 <newline> chitin-based extracellular matrix ; GO:0062129 | inferred from sequence model	structural constituent of cuticle ; GO:0042302 | inferred from electronic annotation with InterPro:IPR000618 <newline> structural constituent of chitin-based larval cuticle ; GO:0008010 | inferred from sequence model	-	3R
L1	CG7333	FBgn0038715	CG7333	-	CG7333	transmembrane transport ; GO:0055085 | inferred from electronic annotation with InterPro:IPR005828	integral component of membrane ; GO:0016021 | inferred from electronic annotation with InterPro:IPR005828	transmembrane transporter activity ; GO:0022857 | inferred from electronic annotation with InterPro:IPR005828	Hsap\SLC22A23 <newline> Hsap\SLC22A15 <newline> Hsap\SLC22A24 <newline> Hsap\SLC22A17 <newline> Hsap\SLC22A14 <newline> Hsap\SLC22A9 <newline> Hsap\SLC22A10 <newline> Hsap\SLC22A5 <newline> Hsap\SLC22A31 <newline> Hsap\SLC22A20P <newline> Hsap\SLC22A4 <newline> Hsap\SLC22A7 <newline> Hsap\SLC22A2 <newline> Hsap\SLC22A12 <newline> Hsap\SLC22A25 <newline> Hsap\SLC22A11 <newline> Hsap\SLC22A13 <newline> Hsap\SLC22A6 <newline> Hsap\SLC22A1 <newline> Hsap\SLC22A8	3R

L1	CG7342	FBgn0038716	CG7342	-	CG7342	transmembrane transport ; GO:0055085 | inferred from electronic annotation with InterPro:IPR005828	integral component of membrane ; GO:0016021 | inferred from electronic annotation with InterPro:IPR005828	transmembrane transporter activity ; GO:0022857 | inferred from electronic annotation with InterPro:IPR005828	Hsap\SLC22A17 <newline><br>Hsap\SLC22A7 <newline><br>Hsap\SLC22A4 <newline><br>Hsap\SLC22A10 <newline><br>Hsap\SLC22A9 <newline><br>Hsap\SLC22A14 <newline><br>Hsap\SLC22A31 <newline><br>Hsap\SLC22A15 <newline><br>Hsap\SLC22A13 <newline><br>Hsap\SLC22A24 <newline><br>Hsap\SLC22A20P <newline><br>Hsap\SLC22A8 <newline><br>Hsap\SLC22A25 <newline><br>Hsap\SLC22A12 <newline><br>Hsap\SLC22A23 <newline><br>Hsap\SLC22A1 <newline><br>Hsap\SLC22A6 <newline><br>Hsap\SLC22A5 <newline><br>Hsap\SLC22A2 <newline><br>Hsap\SLC22A11	3R

L1	CG17751	FBgn0038717	CG17751	-	CG17751	transmembrane transport ; GO:0055085 | inferred from electronic annotation with InterPro:IPR011701	integral component of membrane ; GO:0016021 | inferred from electronic annotation with InterPro:IPR011701	-		Hsap\SLC22A10 <newline><br>Hsap\SLC22A23 <newline><br>Hsap\SLC2A11 <newline><br>Hsap\SLC22A25 <newline><br>Hsap\SLC2A14 <newline><br>Hsap\SLC2A5 <newline><br>Hsap\SLC2A4 <newline><br>Hsap\SLC2A8 <newline><br>Hsap\SLC22A5 <newline><br>Hsap\SLC22A24 <newline><br>Hsap\SLC22A2 <newline><br>Hsap\SLC2A2 <newline><br>Hsap\SLC2A10 <newline><br>Hsap\SLC22A9 <newline><br>Hsap\SLC22A1 <newline><br>Hsap\SLC2A6 <newline><br>Hsap\SLC22A8 <newline><br>Hsap\SLC2A13 <newline><br>Hsap\SLC22A14 <newline><br>Hsap\SLC2A3 <newline><br>Hsap\SLC22A17 <newline><br>Hsap\SLC22A31 <newline><br>Hsap\SLC22A12 <newline><br>Hsap\SLC22A13 <newline><br>Hsap\SLC2A1 <newline><br>Hsap\SLC22A4 <newline><br>Hsap\SLC22A11 <newline><br>[illegible]	3R

L1	CG17752	FBgn0038718	CG17752	-	CG17752	transmembrane transport ; GO:0055085 | inferred from electronic annotation with InterPro:IPR005828	integral component of membrane ; GO:0016021 | inferred from electronic annotation with InterPro:IPR005828	transmembrane transporter activity ; GO:0022857 | inferred from electronic annotation with InterPro:IPR005828	Hsap\SLC22A8 <newline><br>Hsap\SLC2A6 <newline><br>Hsap\SLC2A5 <newline><br>Hsap\SLC2A7 <newline><br>Hsap\SLC2A9 <newline><br>Hsap\SLC22A24 <newline><br>Hsap\SLC22A9 <newline><br>Hsap\SLC22A11 <newline><br>Hsap\SLC22A23 <newline><br>Hsap\SLC2A10 <newline><br>Hsap\SLC22A4 <newline><br>Hsap\SLC22A17 <newline><br>Hsap\SLC22A20P <newline><br>Hsap\SLC2A1 <newline><br>Hsap\SLC22A15 <newline><br>Hsap\SLC2A4 <newline><br>Hsap\SLC22A5 <newline><br>Hsap\SLC2A13 <newline><br>Hsap\SLC2A14 <newline><br>Hsap\SLC2A2 <newline><br>Hsap\SLC22A6 <newline><br>Hsap\SLC22A2 <newline><br>Hsap\SLC2A12 <newline><br>Hsap\SLC22A7 <newline><br>Hsap\SLC2A11 <newline><br>Hsap\SLC22A31 <newline><br>Hsap\SLC22A1 <newline><br>[illegible]	3R

L1	CG16727	FBgn0038719	CG16727	-	CG16727	transmembrane transport ; GO:0055085 | inferred from electronic annotation with InterPro:IPR005828	integral component of membrane ; GO:0016021 | inferred from electronic annotation with InterPro:IPR005828	transmembrane transporter activity ; GO:0022857 | inferred from electronic annotation with InterPro:IPR005828	Hsap\SLC2A4 <newline><br>Hsap\SLC22A9 <newline><br>Hsap\SLC2A9 <newline><br>Hsap\SVOP <newline><br>Hsap\SLC22A13 <newline><br>Hsap\SLC2A1 <newline><br>Hsap\SV2C <newline><br>Hsap\SLC22A25 <newline><br>Hsap\SLC2A7 <newline><br>Hsap\SLC22A6 <newline><br>Hsap\SLC22A7 <newline><br>Hsap\SLC22A11 <newline><br>Hsap\SLC22A1 <newline><br>Hsap\SLC22A4 <newline><br>Hsap\SLC22A5 <newline><br>Hsap\SLC2A12 <newline><br>Hsap\SLC22A14 <newline><br>Hsap\SLC2A2 <newline><br>Hsap\SLC22A2 <newline><br>Hsap\SLC2A14 <newline><br>Hsap\SLC22A20P <newline><br>Hsap\SV2B <newline><br>Hsap\SLC22A12 <newline><br>Hsap\SLC2A3 <newline><br>Hsap\SLC2A8 <newline><br>Hsap\SLC22A10 <newline><br>Hsap\SLC2A5 <newline><br>[illegible]	3R

L1	CG6231	FBgn0038720	CG6231	-	CG6231	transmembrane transport ; GO:0055085 | inferred from electronic annotation with InterPro:IPR005828	integral component of membrane ; GO:0016021 | inferred from electronic annotation with InterPro:IPR005828	transmembrane transporter activity ; GO:0022857 | inferred from electronic annotation with InterPro:IPR005828	Hsap\SV2B <newline> Hsap\SLC22A4 <newline> Hsap\SVOP <newline> Hsap\SLC22A24 <newline> Hsap\SLC22A15 <newline> Hsap\SLC22A9 <newline> Hsap\SLC22A13 <newline> Hsap\SLC22A23 <newline> Hsap\SLC22A8 <newline> Hsap\SLC22A17 <newline> Hsap\SLC22A31 <newline> Hsap\SLC22A7 <newline> Hsap\SLC22A11 <newline> Hsap\SLC22A12 <newline> Hsap\SLC22A20P <newline> Hsap\SLC22A1 <newline> Hsap\SLC22A2 <newline> Hsap\SLC22A5 <newline> Hsap\SLC22A6 <newline> Hsap\SLC22A14 <newline> Hsap\SLC22A10 <newline> Hsap\SLC22A25 <newline> Hsap\SV2C	3R
L1	CG16718	FBgn0038721	CG16718	subdued	subdued	chloride transport ; GO:0006821 | inferred from direct assay <newline> defense response to Gram-negative bacterium ; GO:0050829 | inferred from mutant phenotype <newline> detection of temperature stimulus involved in sensory perception of pain ; GO:0050965 | inferred from mutant phenotype	-	protein dimerization activity ; GO:0046983 | inferred from electronic annotation with InterPro:IPR032394 <newline> intracellular calcium activated chloride channel activity ; GO:0005229 | inferred from direct assay <newline> voltage-gated chloride channel activity ; GO:0005247 | inferred from direct assay <newline> temperature-gated ion channel activity ; GO:0097603 | inferred from direct assay	Hsap\ANO1 <newline> Hsap\ANO3 <newline> Hsap\ANO8 <newline> Hsap\ANO9 <newline> Hsap\ANO2 <newline> Hsap\ANO4 <newline> Hsap\ANO5 <newline> Hsap\ANO7 <newline> Hsap\ANO6	3R
L1	CG34138	FBgn0083974	CG34138	-	CG34138	-	-	-	-	3R

L1	Vha13	FBgn0283536	CG6213	Vacuolar H[+] ATPase 13kD subunit	Vha13	proton transmembrane transport ; GO:1902600 | inferred from electronic annotation with InterPro:IPR005124 <newline> liquid clearance, open tracheal system ; GO:0035002 | inferred from high throughput mutant phenotype <newline> terminal branching, open tracheal system ; GO:0007430 | inferred from high throughput mutant phenotype <newline> terminal branching, open tracheal system ; GO:0007430 | inferred from mutant phenotype <newline> proton transmembrane transport ; GO:1902600 | inferred by curator from GO:0033181	plasma membrane proton-transporting V-type ATPase complex ; GO:0033181 | inferred from mutant phenotype <newline> vacuolar proton-transporting V-type ATPase complex ; GO:0016471 | inferred from biological aspect of ancestor with PANTHER:PTN000295490	proton-transporting ATPase activity, rotational mechanism ; GO:0046961 | contributes_to inferred by curator from GO:0033181 <newline> proton-exporting ATPase activity, phosphorylative mechanism ; GO:0008553 | inferred from biological aspect of ancestor with PANTHER:PTN000295490	Hsap\ATP6V1G3 <newline> Hsap\ATP6V1G1 <newline> Hsap\ATP6V1G2	3R
L1	Nup58	FBgn0038722	CG7360	Nucleoporin 58kD	Nup58	nucleocytoplasmic transport ; GO:0006913 | inferred from sequence or structural similarity with UniProtKB:P70581	nuclear pore ; GO:0005643 | inferred from biological aspect of ancestor with PANTHER:PTN001412288 <newline> nuclear envelope ; GO:0005635 | inferred from direct assay <newline> nuclear pore ; GO:0005643 | inferred from sequence or structural similarity with UniProtKB:P70581	structural constituent of nuclear pore ; GO:0017056 | inferred from biological aspect of ancestor with PANTHER:PTN001412288 <newline> nuclear localization sequence binding ; GO:0008139 | inferred from biological aspect of ancestor with PANTHER:PTN001412288 <newline> structural constituent of nuclear pore ; GO:0017056 | inferred from sequence or structural similarity with UniProtKB:P70581	Hsap\NUP62 <newline> Hsap\NUP98 <newline> Hsap\NUP58	3R
L1	CG6195	FBgn0038723	CG6195	-	CG6195	cytoplasmic translation ; GO:0002181 | inferred from biological aspect of ancestor with PANTHER:PTN000193732	cytoplasm ; GO:0005737 | inferred from biological aspect of ancestor with PANTHER:PTN000193732	GTP binding ; GO:0005525 | inferred from biological aspect of ancestor with PANTHER:PTN000193732	Hsap\DRG2 <newline> Hsap\DRG1	3R
L1	CG31220	FBgn0051220	CG31220	-	CG31220	proteolysis ; GO:0006508 | inferred from electronic annotation with InterPro:IPR001254, InterPro:IPR001314, InterPro:IPR018114	-	serine-type endopeptidase activity ; GO:0004252 | inferred from electronic annotation with InterPro:IPR001254, InterPro:IPR001314, InterPro:IPR018114 <newline> serine-type endopeptidase activity ; GO:0004252 | inferred from sequence model	Hsap\CMA1 <newline> Hsap\KLK1	3R

L1	CG31219	FBgn0051219	CG31219	-	CG31219	proteolysis ; GO:0006508 | inferred from electronic annotation with InterPro:IPR001254, InterPro:IPR001314, InterPro:IPR018114 <newline> proteolysis ; GO:0006508 | inferred from sequence model	-	serine-type endopeptidase activity ; GO:0004252 | inferred from electronic annotation with InterPro:IPR001254, InterPro:IPR001314 <newline> serine-type endopeptidase activity ; GO:0004252 | inferred from sequence model	-	3R
L1	CG6184	FBgn0038725	CG6184	-	CG6184	-	-	-	Hsap\FAM234B <newline> Hsap\FAM234A	3R
L1	ort	FBgn0003011	CG7411	ora transientless	ort	signal transduction ; GO:0007165 | inferred from biological aspect of ancestor with PANTHER:PTN000434994 <newline> chemical synaptic transmission ; GO:0007268 | inferred from mutant phenotype <newline> chloride transport ; GO:0006821 | inferred from direct assay <newline> chemical synaptic transmission ; GO:0007268 | inferred from biological aspect of ancestor with PANTHER:PTN000434994 <newline> nervous system process ; GO:0050877 | inferred from biological aspect of ancestor with PANTHER:PTN000434994 <newline> chloride transmembrane transport ; GO:1902476 | inferred from biological aspect of ancestor with PANTHER:PTN001055382 <newline> ion transmembrane transport ; GO:0034220 | inferred from biological aspect of ancestor with PANTHER:PTN000434994 <newline> regulation of membrane potential ; GO:0042391 | inferred from [illegible]	synapse ; GO:0045202 | inferred from biological aspect of ancestor with PANTHER:PTN000434994 <newline> histamine-gated chloride channel complex ; GO:0019183 | inferred from direct assay <newline> integral component of membrane ; GO:0016021 | inferred from sequence model <newline> integral component of plasma membrane ; GO:0005887 | inferred from biological aspect of ancestor with PANTHER:PTN000434994 <newline> neuron projection ; GO:0043005 | inferred from biological aspect of ancestor with PANTHER:PTN000434994	transmembrane signaling receptor activity ; GO:0004888 | inferred from electronic annotation with InterPro:IPR006028, InterPro:IPR006201 <newline> histamine-gated chloride channel activity ; GO:0019182 | inferred from direct assay <newline> extracellular ligand-gated ion channel activity ; GO:0005230 | contributes_to inferred from biological aspect of ancestor with PANTHER:PTN000434994 <newline> histamine-gated chloride channel activity ; GO:0019182 | inferred from sequence or structural similarity <newline> chloride channel activity ; GO:0005254 | contributes_to inferred from biological aspect of ancestor with PANTHER:PTN001055382 inferred from biological aspect of ancestor with PANTHER:PTN000435804	Hsap\GLRA1 <newline> Hsap\GLRA3 <newline> Hsap\GLRA4 <newline> Hsap\GLRB <newline> Hsap\GLRA2	3R
L1	CG7432	FBgn0038727	CG7432	-	CG7432	proteolysis ; GO:0006508 | inferred from electronic annotation with InterPro:IPR001254, InterPro:IPR001314, InterPro:IPR018114 <newline> proteolysis ; GO:0006508 | inferred from sequence model	-	serine-type endopeptidase activity ; GO:0004252 | inferred from electronic annotation with InterPro:IPR001254, InterPro:IPR001314, InterPro:IPR018114 <newline> serine-type endopeptidase activity ; GO:0004252 | inferred from sequence model	Hsap\MST1L <newline> Hsap\PLAT <newline> Hsap\PLG <newline> Hsap\MST1 <newline> Hsap\F2 <newline> Hsap\TMPRSS15 <newline> Hsap\HGF <newline> Hsap\TMPRSS9	3R

L1	Naam	FBgn0051216	CG31216	Nicotinamide amidase	Naam	negative regulation of neuron apoptotic process ; GO:0043524 | inferred from direct assay <newline> determination of adult lifespan ; GO:0008340 | inferred from mutant phenotype <newline> response to oxidative stress ; GO:0006979 | inferred from mutant phenotype	-	calcium ion binding ; GO:0005509 | inferred from electronic annotation with InterPro:IPR002048 <newline> nicotinamidase activity ; GO:0008936 | inferred from direct assay	-	3R
L1	CR31214	FBgn0012003	CR31214	transfer RNA:Valine-CAC 2-5	tRNA:Val-CAC-2-5	translation ; GO:0006412 | inferred by curator from GO:0033452	cytosol ; GO:0005829 | inferred by curator from GO:0033452	GUG codon-amino acid adaptor activity ; GO:0033452 | inferred from sequence model	-	3R
L1	CG34286	FBgn0085315	CG34286	-	CG34286	biological_process ; GO:0008150 | no biological data available	cellular_component ; GO:0005575 | no biological data available	molecular_function ; GO:0003674 | no biological data available	-	3R
L1	GluClalpha	FBgn0024963	CG7535	GluClalpha	GluClalpha	chloride transmembrane transport ; GO:1902476 | inferred from biological aspect of ancestor with PANTHER:PTN001055382 <newline> signal transduction ; GO:0007165 | inferred from biological aspect of ancestor with PANTHER:PTN000434994 <newline> chloride transport ; GO:0006821 | inferred from direct assay <newline> ion transmembrane transport ; GO:0034220 | inferred from biological aspect of ancestor with PANTHER:PTN000434994 <newline> chemical synaptic transmission ; GO:0007268 | inferred from biological aspect of ancestor with PANTHER:PTN000434994 <newline> regulation of membrane potential ; GO:0042391 | inferred from biological aspect of ancestor with PANTHER:PTN000434994 <newline> nervous system process ; GO:0050877 | inferred from biological aspect of ancestor with PANTHER:PTN000434994	integral component of plasma membrane ; GO:0005887 | inferred from biological aspect of ancestor with PANTHER:PTN000434994 <newline> synapse ; GO:0045202 | inferred from biological aspect of ancestor with PANTHER:PTN000434994 <newline> neuron projection ; GO:0043005 | inferred from biological aspect of ancestor with PANTHER:PTN000434994 <newline> integral component of membrane ; GO:0016021 | inferred by curator from GO:0008068	transmembrane signaling receptor activity ; GO:0004888 | inferred from electronic annotation with InterPro:IPR006028, InterPro:IPR006201 <newline> extracellular ligand-gated ion channel activity ; GO:0005230 | contributes_to inferred from biological aspect of ancestor with PANTHER:PTN000434994 <newline> extracellularly glutamate-gated chloride channel activity ; GO:0008068 | inferred from direct assay <newline> chloride channel activity ; GO:0005254 | contributes_to inferred from biological aspect of ancestor with PANTHER:PTN001055382 inferred from biological aspect of ancestor with PANTHER:PTN000435804	Hsap\GABRA3 <newline> Hsap\GABRB1 <newline> Hsap\GABRA5 <newline> Hsap\GABRA6 <newline> Hsap\GABRR1 <newline> Hsap\ZACN <newline> Hsap\GABRA4 <newline> Hsap\GABRQ <newline> Hsap\GABRD <newline> Hsap\GABRR3 <newline> Hsap\GLRA3 <newline> Hsap\GABRG2 <newline> Hsap\GLRA1 <newline> Hsap\GLRB <newline> Hsap\GABRB2 <newline> Hsap\GLRA4 <newline> Hsap\GLRA2 <newline> Hsap\GABRB3 <newline> Hsap\GABRE <newline> Hsap\GABRP <newline> Hsap\GABRR2 <newline> Hsap\GABRG3 <newline> Hsap\GABRG1 <newline> Hsap\GABRA2 <newline> Hsap\GABRA1	3R

L1	CG6300	FBgn0038730	CG6300	-	CG6300	fatty acid biosynthetic process ; GO:0006633 | traceable author statement	-	catalytic activity ; GO:0003824 | inferred from electronic annotation with InterPro:IPR000873 <newline> CoA-ligase activity ; GO:0016405 | traceable author statement <newline> fatty acid ligase activity ; GO:0015645 | traceable author statement	Hsap\ACSF3	3R
L1	CG11659	FBgn0038731	CG11659	-	CG11659	fatty acid biosynthetic process ; GO:0006633 | traceable author statement	-	catalytic activity ; GO:0003824 | inferred from electronic annotation with InterPro:IPR000873 <newline> CoA-ligase activity ; GO:0016405 | traceable author statement <newline> fatty acid ligase activity ; GO:0015645 | traceable author statement	Hsap\ACSF3 <newline> Hsap\ACSF2	3R
L1	CG11391	FBgn0038732	CG11391	-	CG11391	fatty acid biosynthetic process ; GO:0006633 | traceable author statement	-	catalytic activity ; GO:0003824 | inferred from electronic annotation with InterPro:IPR000873 <newline> CoA-ligase activity ; GO:0016405 | traceable author statement <newline> fatty acid ligase activity ; GO:0015645 | traceable author statement	Hsap\SLC27A4 <newline> Hsap\ACSF3	3R
L1	CG11407	FBgn0038733	CG11407	-	CG11407	fatty acid biosynthetic process ; GO:0006633 | traceable author statement	-	catalytic activity ; GO:0003824 | inferred from electronic annotation with InterPro:IPR000873 <newline> fatty acid ligase activity ; GO:0015645 | traceable author statement <newline> CoA-ligase activity ; GO:0016405 | traceable author statement	Hsap\ACSF3 <newline> Hsap\ACSF2	3R
L1	CG11453	FBgn0038734	CG11453	-	CG11453	fatty acid biosynthetic process ; GO:0006633 | traceable author statement	-	catalytic activity ; GO:0003824 | inferred from electronic annotation with InterPro:IPR000873 <newline> CoA-ligase activity ; GO:0016405 | traceable author statement <newline> fatty acid ligase activity ; GO:0015645 | traceable author statement	Hsap\AACS <newline> Hsap\ACSF3	3R

L1	CR31430	FBgn0012004	CR31430	transfer RNA:Valine-CAC 2-6	tRNA:Val-CAC-2-6	translation ; GO:0006412 | inferred by curator from GO:0033452	cytosol ; GO:0005829 | inferred by curator from GO:0033452	GUG codon-amino acid adaptor activity ; GO:0033452 | inferred from sequence model	-	3R
L1	CR31215	FBgn0051215	CR31215	transfer RNA:Alanine-AGC 2-11	tRNA:Ala-AGC-2-11	translation ; GO:0006412 | inferred by curator from GO:0033453	cytosol ; GO:0005829 | inferred by curator from GO:0033453	GCU codon-amino acid adaptor activity ; GO:0033453 | inferred from sequence model	-	3R
L1	bnl	FBgn0014135	CG4608	branchless	bnl	fibroblast growth factor receptor signaling pathway ; GO:0008543 | inferred from genetic interaction with btl <newline> cytoneme assembly ; GO:0035231 | inferred from direct assay <newline> genital disc development ; GO:0035215 | inferred from expression pattern <newline> fibroblast growth factor receptor signaling pathway ; GO:0008543 | inferred from mutant phenotype <newline> terminal branching, open tracheal system ; GO:0007430 | inferred from mutant phenotype <newline> epithelial cell migration, open tracheal system ; GO:0007427 | inferred from mutant phenotype <newline> positive regulation of ERK1 and ERK2 cascade ; GO:0070374 | inferred from expression pattern <newline> regulation of epithelial cell migration, open tracheal system ; GO:2000274 | inferred from mutant phenotype <newline> primary branching, open tracheal system ; GO:0007428 | inferred from mutant [illegible]	extracellular space ; GO:0005615 | inferred from sequence or structural similarity with UniProtKB:P05230	growth factor activity ; GO:0008083 | inferred from electronic annotation with InterPro:IPR002209 <newline> fibroblast growth factor receptor binding ; GO:0005104 | inferred from mutant phenotype <newline> chemoattractant activity ; GO:0042056 | inferred from genetic interaction with btl <newline> fibroblast growth factor receptor binding ; GO:0005104 | traceable author statement	Hsap\FGF3 <newline> Hsap\FGF22 <newline> Hsap\FGF12 <newline> Hsap\FGF2 <newline> Hsap\FGF9 <newline> Hsap\FGF21 <newline> Hsap\FGF14 <newline> Hsap\FGF20 <newline> Hsap\FGF19 <newline> Hsap\FGF18 <newline> Hsap\FGF13 <newline> Hsap\FGF10 <newline> Hsap\FGF17 <newline> Hsap\FGF5 <newline> Hsap\FGF11 <newline> Hsap\FGF1 <newline> Hsap\FGF7 <newline> Hsap\FGF4 <newline> Hsap\FGF16 <newline> Hsap\FGF23 <newline> Hsap\FGF6 <newline> Hsap\FGF8	3R
L1	CR31471	FBgn0051471	CR31471	transfer RNA:Threonine-AGT 1-7	tRNA:Thr-AGT-1-7	translation ; GO:0006412 | inferred by curator from GO:0033437	cytosol ; GO:0005829 | inferred by curator from GO:0033437	ACU codon-amino acid adaptor activity ; GO:0033437 | inferred from sequence model	-	3R
L1	CG31459	FBgn0051459	CG31459	-	CG31459	biological_process ; GO:0008150 | no biological data available	cellular_component ; GO:0005575 | no biological data available	molecular_function ; GO:0003674 | no biological data available	-	3R
L1	CG4662	FBgn0038735	CG4662	Mitochondrial calcium uptake 3	MICU3	mitochondrial calcium ion transmembrane transport ; GO:0006851 | inferred from electronic annotation with InterPro:IPR039800	-	calcium ion binding ; GO:0005509 | inferred from electronic annotation with InterPro:IPR002048	Hsap\MICU2 <newline> Hsap\MICU3 <newline> Hsap\MICU1	3R

L1	Ire1	FBgn0261984	CG4583	Inositol-requiring enzyme-1	Ire1	mRNA processing ; GO:0006397 | inferred from electronic annotation with InterPro:IPR010513 <newline> protein phosphorylation ; GO:0006468 | inferred from electronic annotation with InterPro:IPR000719, InterPro:IPR008271 <newline> endoplasmic reticulum unfolded protein response ; GO:0030968 | inferred from biological aspect of ancestor with PANTHER:PTN000359335 <newline> response to endoplasmic reticulum stress ; GO:0034976 | inferred from direct assay <newline> IRE1-mediated unfolded protein response ; GO:0036498 | inferred from biological aspect of ancestor with PANTHER:PTN000359335 <newline> compound eye photoreceptor fate commitment ; GO:0001752 | NOT inferred from mutant phenotype <newline> regulation of RNA splicing ; GO:0043484 | inferred from direct assay <newline> endoplasmic [illegible]	IRE1-TRAF2-ASK1 complex ; GO:1990604 | inferred from biological aspect of ancestor with PANTHER:PTN000359335 <newline> endoplasmic reticulum membrane ; GO:0005789 | inferred from biological aspect of ancestor with PANTHER:PTN000359335	ATP binding ; GO:0005524 | inferred from electronic annotation with InterPro:IPR000719 <newline> protein kinase activity ; GO:0004672 | inferred from biological aspect of ancestor with PANTHER:PTN000359335 <newline> endoribonuclease activity ; GO:0004521 | inferred from mutant phenotype <newline> protein serine/threonine kinase activity ; GO:0004674 | inferred from biological aspect of ancestor with PANTHER:PTN000359335 <newline> unfolded protein binding ; GO:0051082 | inferred from biological aspect of ancestor with PANTHER:PTN000359335 <newline> endoribonuclease activity ; GO:0004521 | inferred from biological aspect of ancestor with PANTHER:PTN000359335 <newline> endoribonuclease activity ; GO:0004521 | inferred from direct assay	Hsap\ERN2 <newline> Hsap\RNASEL <newline> Hsap\ERN1	3R
L1	CG11447	FBgn0038737	CG11447	-	CG11447	RNA methylation ; GO:0001510 | inferred from electronic annotation with InterPro:IPR015507	mitochondrion ; GO:0005739 | inferred from biological aspect of ancestor with PANTHER:PTN000100456	rRNA (uridine-2'-O-)-methyltransferase activity ; GO:0008650 | inferred from biological aspect of ancestor with PANTHER:PTN000100455	Hsap\FTSJ3 <newline> Hsap\MRM2	3R
L1	CG4572	FBgn0038738	CG4572	-	CG4572	dsRNA transport ; GO:0033227 | inferred from mutant phenotype <newline> proteolysis involved in cellular protein catabolic process ; GO:0051603 | inferred from biological aspect of ancestor with PANTHER:PTN000210642	-	serine-type carboxypeptidase activity ; GO:0004185 | inferred from biological aspect of ancestor with PANTHER:PTN000210642	Hsap\CTSA <newline> Hsap\SCPEP1 <newline> Hsap\CPVL	3R

L1	CG4562	FBgn0038740	CG4562	-	CG4562	transmembrane transport ; GO:0055085 | inferred from biological aspect of ancestor with PANTHER:PTN000657997	integral component of membrane ; GO:0016021 | inferred from electronic annotation with InterPro:IPR011527, InterPro:IPR036640 <newline> membrane ; GO:0016020 | inferred from biological aspect of ancestor with PANTHER:PTN000657997	ATP binding ; GO:0005524 | inferred from electronic annotation with InterPro:IPR003439, InterPro:IPR011527, InterPro:IPR017871 <newline> ATPase activity ; GO:0016887 | inferred from electronic annotation with InterPro:IPR003439, InterPro:IPR017871 <newline> ATPase-coupled transmembrane transporter activity ; GO:0042626 | inferred from biological aspect of ancestor with PANTHER:PTN000657997	Hsap\CFTR <newline> Hsap\ABCC10 <newline> Hsap\ABCC11 <newline> Hsap\ABCC5 <newline> Hsap\ABCC4 <newline> Hsap\ABCC3 <newline> Hsap\ABCC1 <newline> Hsap\ABCC9 <newline> Hsap\LOC105369239 <newline> Hsap\ABCC12 <newline> Hsap\ABCC2 <newline> Hsap\ABCC8 <newline> Hsap\ABCC6	3R
L1	CG4686	FBgn0038739	CG4686	-	CG4686	-	-	-	Hsap\RPS29 <newline> Hsap\TMEM256	3R
L1	CG17186	FBgn0038741	CG17186	-	CG17186	biological_process ; GO:0008150 | no biological data available	cellular_component ; GO:0005575 | no biological data available	nucleic acid binding ; GO:0003676 | inferred from electronic annotation with InterPro:IPR013087 <newline> molecular_function ; GO:0003674 | no biological data available	Hsap\ZNF385C <newline> Hsap\ZNF385B <newline> Hsap\ZNF385A <newline> Hsap\ZNF385D <newline> Hsap\STRBP <newline> Hsap\ILF3 <newline> Hsap\ZFR2 <newline> Hsap\ZFR	3R
L1	Arc42	FBgn0038742	CG4703	Arc42	Arc42	oxidation-reduction process ; GO:0055114 | inferred from electronic annotation with InterPro:IPR006089, InterPro:IPR006091, InterPro:IPR009075, InterPro:IPR009100, InterPro:IPR013786 <newline> butyrate catabolic process ; GO:0046359 | inferred from biological aspect of ancestor with PANTHER:PTN000097838	cellular_component ; GO:0005575 | no biological data available	acyl-CoA dehydrogenase activity ; GO:0003995 | inferred from electronic annotation with InterPro:IPR006089, InterPro:IPR006091 <newline> flavin adenine dinucleotide binding ; GO:0050660 | inferred from electronic annotation with InterPro:IPR013786 <newline> butyryl-CoA dehydrogenase activity ; GO:0004085 | inferred from biological aspect of ancestor with PANTHER:PTN000097838	Hsap\ACAD9 <newline> Hsap\ACADVL <newline> Hsap\ACAD8 <newline> Hsap\ACADS <newline> Hsap\IVD <newline> Hsap\ACADSB <newline> Hsap\ACADM	3R

L1	CG4720	FBgn0014006	CG4720	Apoptotic signal-regulating kinase 1	Ask1	regulation of adult chitin-containing cuticle pigmentation ; GO:0048082 | inferred from mutant phenotype <newline> MAPK cascade ; GO:0000165 | inferred from sequence or structural similarity with HGNC:6857 <newline> protein phosphorylation ; GO:0006468 | inferred from sequence or structural similarity <newline> positive regulation of JNK cascade ; GO:0046330 | inferred from direct assay <newline> positive regulation of stress-activated MAPK cascade ; GO:0032874 | inferred from mutant phenotype <newline> positive regulation of stress-activated MAPK cascade ; GO:0032874 | inferred from direct assay inferred from mutant phenotype <newline> activation of MAPKK activity ; GO:0000186 | inferred from sequence or structural similarity with HGNC:6857	protein kinase complex ; GO:1902911 | inferred from sequence or structural similarity with HGNC:6857	ATP binding ; GO:0005524 | inferred from electronic annotation with InterPro:IPR000719, InterPro:IPR002290, InterPro:IPR017441 <newline> MAP kinase kinase kinase activity ; GO:0004709 | inferred from sequence or structural similarity <newline> protein kinase activity ; GO:0004672 | inferred from biological aspect of ancestor with PANTHER:PTN000172316 <newline> protein binding ; GO:0005515 | inferred from physical interaction with UniProtKB:O46084 <newline> MAP kinase kinase kinase activity ; GO:0004709 | inferred from sequence or structural similarity with HGNC:6857	Hsap\MAP3K15 <newline> Hsap\MAP3K5 <newline> Hsap\MAP3K1 <newline> Hsap\MAP3K6	3R

L1	ninaE	FBgn0002940	CG4550	neither inactivation nor afterpotential E	ninaE	visual perception ; GO:0007601 | inferred from electronic annotation with InterPro:IPR001760 <newline> negative regulation of compound eye retinal cell programmed cell death ; GO:0046673 | inferred from mutant phenotype <newline> phototransduction ; GO:0007602 | non-traceable author statement <newline> cellular response to light stimulus ; GO:0071482 | inferred from mutant phenotype <newline> detection of UV ; GO:0009589 | inferred from mutant phenotype <newline> phospholipase C-activating rhodopsin mediated signaling pathway ; GO:0030265 | inferred from mutant phenotype <newline> phototransduction ; GO:0007602 | inferred from sequence or structural similarity <newline> thermotaxis ; GO:0043052 | inferred from mutant phenotype <newline> cellular response to light stimulus ; GO:0071482 | inferred from biological aspect of ancestor with [illegible]	subrhabdomeral cisterna ; GO:0016029 | inferred from direct assay <newline> rhabdomere ; GO:0016028 | inferred from direct assay <newline> multivesicular body ; GO:0005771 | inferred from direct assay <newline> integral component of membrane ; GO:0016021 | inferred from sequence or structural similarity <newline> integral component of plasma membrane ; GO:0005887 | inferred from biological aspect of ancestor with PANTHER:PTN000662115 <newline> early endosome ; GO:0005769 | inferred from direct assay <newline> secondary lysosome ; GO:0005767 | inferred from direct assay <newline> inaD signaling complex ; GO:0016027 | traceable author statement <newline> perinuclear endoplasmic reticulum ; GO:0097038 | colocalizes_with inferred from direct assay <newline> inaD signaling complex ; [illegible]	protein binding ; GO:0005515 | inferred from physical interaction with Arr2 <newline> G protein-coupled photoreceptor activity ; GO:0008020 | inferred from biological aspect of ancestor with PANTHER:PTN000662115 <newline> G protein-coupled photoreceptor activity ; GO:0008020 | non-traceable author statement <newline> G protein-coupled photoreceptor activity ; GO:0008020 | inferred from sequence or structural similarity <newline> G protein-coupled photoreceptor activity ; GO:0008020 | inferred from mutant phenotype	Hsap\OPN3 <newline> Hsap\RHO <newline> Hsap\OPN1MW2 <newline> Hsap\OPN1LW <newline> Hsap\OPN1MW <newline> Hsap\OPN1SW <newline> Hsap\RRH <newline> Hsap\OPN5 <newline> Hsap\OPN4 <newline> Hsap\OPN1MW3 <newline> Hsap\RGR	3R
L1	CG4733	FBgn0038744	CG4733	-	CG4733	protein dephosphorylation ; GO:0006470 | inferred from biological aspect of ancestor with PANTHER:PTN000363548	protein phosphatase type 2A complex ; GO:0000159 | inferred from biological aspect of ancestor with PANTHER:PTN000363548	calcium ion binding ; GO:0005509 | inferred from electronic annotation with InterPro:IPR002048 <newline> protein phosphatase regulator activity ; GO:0019888 | inferred from biological aspect of ancestor with PANTHER:PTN000363548	Hsap\PPP2R3A <newline> Hsap\PPP2R3B <newline> Hsap\PPP2R3C	3R

L1	CG4538	FBgn0038745	CG4538	-	CG4538	protein folding ; GO:0006457 | inferred from electronic annotation with InterPro:IPR004487 <newline> proteolysis ; GO:0006508 | inferred from sequence or structural similarity with UniProtKB:O76031 <newline> protein catabolic process ; GO:0030163 | inferred from biological aspect of ancestor with PANTHER:PTN000137292	mitochondrial matrix ; GO:0005759 | inferred from biological aspect of ancestor with PANTHER:PTN000137292 <newline> mitochondrion ; GO:0005739 | inferred from sequence or structural similarity with UniProtKB:O76031	unfolded protein binding ; GO:0051082 | inferred from electronic annotation with InterPro:IPR004487 <newline> serine-type endopeptidase activity ; GO:0004252 | inferred from sequence or structural similarity with UniProtKB:O76031 <newline> ATP binding ; GO:0005524 | inferred from biological aspect of ancestor with PANTHER:PTN000137292 <newline> ATP-dependent peptidase activity ; GO:0004176 | inferred from biological aspect of ancestor with PANTHER:PTN000137292	Hsap\CLPX	3R
L1	Surf6	FBgn0038746	CG4510	Surfeit 6	Surf6	ventral cord development ; GO:0007419 | inferred from high throughput mutant phenotype <newline> ribosomal small subunit biogenesis ; GO:0042274 | inferred from biological aspect of ancestor with PANTHER:PTN000372642 <newline> ribosome biogenesis ; GO:0042254 | inferred from sequence or structural similarity with MGI:MGI:98447 <newline> ribosomal large subunit biogenesis ; GO:0042273 | inferred from biological aspect of ancestor with PANTHER:PTN000372642	nucleolus ; GO:0005730 | inferred from biological aspect of ancestor with PANTHER:PTN000372642 <newline> nucleolus ; GO:0005730 | inferred from sequence or structural similarity with MGI:MGI:98447	DNA binding ; GO:0003677 | inferred from biological aspect of ancestor with PANTHER:PTN000372642 <newline> nucleic acid binding ; GO:0003676 | inferred from sequence or structural similarity with MGI:MGI:98447 <newline> RNA binding ; GO:0003723 | inferred from biological aspect of ancestor with PANTHER:PTN000372642	Hsap\SURF6	3R

L1	RhoGAP92B	FBgn0038747	CG4755	Rho GTPase activating protein at 92B	RhoGAP92B	signal transduction ; GO:0007165 | inferred from electronic annotation with InterPro:IPR000198, InterPro:IPR008936 <newline> regulation of actin cytoskeleton organization ; GO:0032956 | inferred from biological aspect of ancestor with PANTHER:PTN001797409 <newline> negative regulation of actin filament polymerization ; GO:0030837 | inferred from mutant phenotype <newline> cellular protein localization ; GO:0034613 | inferred from mutant phenotype <newline> neuromuscular synaptic transmission ; GO:0007274 | inferred from mutant phenotype <newline> imaginal disc-derived leg morphogenesis ; GO:0007480 | inferred from mutant phenotype <newline> positive regulation of synaptic growth at neuromuscular junction ; GO:0045887 | inferred from mutant phenotype <newline> positive regulation of GTPase activity ; GO:0043547 | inferred [illegible]	cytoplasm ; GO:0005737 | inferred from electronic annotation with InterPro:IPR004148 <newline> neuromuscular junction ; GO:0031594 | inferred from direct assay	phospholipid binding ; GO:0005543 | inferred from sequence or structural similarity with HGNC:29096 <newline> SH3 domain binding ; GO:0017124 | inferred from physical interaction with Cip4 <newline> GTPase activator activity ; GO:0005096 | inferred from biological aspect of ancestor with PANTHER:PTN001797409 <newline> GTPase activator activity ; GO:0005096 | inferred from direct assay <newline> Rac GTPase binding ; GO:0048365 | inferred from biological aspect of ancestor with PANTHER:PTN001797409	Hsap\INPP5B <newline> Hsap\SH3BP1 <newline> Hsap\FAM13A <newline> Hsap\ARHGAP25 <newline> Hsap\ARHGAP17 <newline> Hsap\PDXP <newline> Hsap\ARHGAP22 <newline> Hsap\FAM13B <newline> Hsap\ARHGAP44 <newline> Hsap\ARHGAP24	3R
L1	CG4468	FBgn0038749	CG4468	exit protein of rhodopsin and TRP A	Xport-A	chaperone-mediated protein transport ; GO:0072321 | inferred from direct assay <newline> protein localization to rhabdomere ; GO:1990146 | inferred from mutant phenotype <newline> retina homeostasis ; GO:0001895 | inferred from mutant phenotype <newline> phototransduction, visible light ; GO:0007603 | inferred from mutant phenotype <newline> cellular response to light stimulus ; GO:0071482 | inferred from mutant phenotype <newline> rhodopsin biosynthetic process ; GO:0016063 | inferred from mutant phenotype <newline> positive regulation of protein targeting to membrane ; GO:0090314 | inferred from mutant phenotype	perinuclear endoplasmic reticulum ; GO:0097038 | inferred from direct assay	-	-	3R

L1	CG42508	FBgn0260234	CG42508	exit protein of rhodopsin and TRP B	Xport-B	protein localization to rhabdomere ; GO:1990146 | inferred from mutant phenotype	subrhabdomeral cisterna ; GO:0016029 | inferred from direct assay <newline> integral component of membrane ; GO:0016021 | inferred from direct assay <newline> endoplasmic reticulum ; GO:0005783 | inferred from direct assay	molecular_function ; GO:0003674 | no biological data available	-	3R
L1	CG4465	FBgn0038750	CG4465	-	CG4465	transmembrane transport ; GO:0055085 | inferred from electronic annotation with InterPro:IPR011701	integral component of membrane ; GO:0016021 | inferred from electronic annotation with InterPro:IPR011701	-	Hsap\SLC22A2 <newline> Hsap\SLC22A7 <newline> Hsap\SLC22A10 <newline> Hsap\SLC22A6 <newline> Hsap\SLC22A15 <newline> Hsap\SLC22A24 <newline> Hsap\SLC22A8 <newline> Hsap\SLC22A11 <newline> Hsap\SV2B <newline> Hsap\SLC22A25 <newline> Hsap\SLC22A23 <newline> Hsap\SLC22A9 <newline> Hsap\SVOP <newline> Hsap\SV2C <newline> Hsap\SLC22A12 <newline> Hsap\SLC22A1	3R
L1	CG4770	FBgn0038751	CG4770	-	CG4770	long-chain fatty-acyl-CoA metabolic process ; GO:0035336 | inferred from biological aspect of ancestor with PANTHER:PTN000110226	peroxisome ; GO:0005777 | inferred from biological aspect of ancestor with PANTHER:PTN000110227	fatty-acyl-CoA reductase (alcohol-forming) activity ; GO:0080019 | inferred from biological aspect of ancestor with PANTHER:PTN000110226 <newline> fatty-acyl-CoA reductase (alcohol-forming) activity ; GO:0080019 | inferred from sequence or structural similarity with HGNC:26222	Hsap\FAR2 <newline> Hsap\FAR1	3R

L1	CG4462	FBgn0038752	CG4462	-	CG4462	transmembrane transport ; GO:0055085 | inferred from electronic annotation with InterPro:IPR011701	integral component of membrane ; GO:0016021 | inferred from electronic annotation with InterPro:IPR011701	-	Hsap\SLC22A13 <newline> Hsap\SLC22A15 <newline> Hsap\SLC22A4 <newline> Hsap\SLC22A6 <newline> Hsap\SLC22A1 <newline> Hsap\SLC22A2 <newline> Hsap\SLC22A14 <newline> Hsap\SLC22A10 <newline> Hsap\SLC22A23 <newline> Hsap\SLC22A11 <newline> Hsap\SV2B <newline> Hsap\SLC22A7 <newline> Hsap\SLC22A9 <newline> Hsap\SLC22A8 <newline> Hsap\SLC22A24 <newline> Hsap\SLC22A25 <newline> Hsap\SLC22A20P <newline> Hsap\SLC22A5 <newline> Hsap\SLC22A12 <newline> Hsap\SV2C <newline> Hsap\SVOP	3R

L1	CG4459	FBgn0038753	CG4459	-	CG4459	transmembrane transport ; GO:0055085 | inferred from electronic annotation with InterPro:IPR011701	integral component of membrane ; GO:0016021 | inferred from electronic annotation with InterPro:IPR011701	-	Hsap\SLC22A10 <newline> Hsap\SVOP <newline> Hsap\SLC22A5 <newline> Hsap\SLC22A14 <newline> Hsap\SLC22A9 <newline> Hsap\SV2B <newline> Hsap\SLC22A15 <newline> Hsap\SLC22A20P <newline> Hsap\SLC22A24 <newline> Hsap\SLC22A12 <newline> Hsap\SLC22A7 <newline> Hsap\SLC22A4 <newline> Hsap\SLC22A6 <newline> Hsap\SV2C <newline> Hsap\SLC22A1 <newline> Hsap\SLC22A23 <newline> Hsap\SLC22A11 <newline> Hsap\SLC22A8 <newline> Hsap\SLC22A2 <newline> Hsap\SLC22A25 <newline> Hsap\SLC22A13	3R

L1	Hs6st	FBgn0038755	CG4451	Heparan sulfate 6-O-sulfotransferase	Hs6st	heparan sulfate proteoglycan biosynthetic process, enzymatic modification ; GO:0015015 | inferred from biological aspect of ancestor with PANTHER:PTN000301790 <newline> imaginal disc-derived wing margin morphogenesis ; GO:0008587 | inferred from mutant phenotype <newline> open tracheal system development ; GO:0007424 | inferred from mutant phenotype inferred from genetic interaction with Hs2st <newline> chaeta development ; GO:0022416 | inferred from mutant phenotype <newline> heparan sulfate proteoglycan biosynthetic process ; GO:0015012 | inferred from mutant phenotype <newline> regulation of imaginal disc growth ; GO:0045570 | inferred from mutant phenotype	integral component of membrane ; GO:0016021 | inferred from electronic annotation with InterPro:IPR005331, InterPro:IPR010635	heparan sulfate 6-O-sulfotransferase activity ; GO:0017095 | inferred from biological aspect of ancestor with PANTHER:PTN000301790	Hsap\HS6ST1 <newline> Hsap\HS6ST3 <newline> Hsap\HS6ST2	3R

L1	mira	FBgn0021776	CG12249	miranda	mira	asymmetric protein localization involved in cell fate determination ; GO:0045167 | inferred from mutant phenotype <newline> neuroblast fate determination ; GO:0007400 | traceable author statement <newline> neuroblast proliferation ; GO:0007405 | inferred from mutant phenotype <newline> oogenesis ; GO:0048477 | inferred from genetic interaction with cup <newline> ventral cord development ; GO:0007419 | inferred from high throughput mutant phenotype <newline> regulation of proteolysis ; GO:0030162 | inferred from mutant phenotype <newline> asymmetric protein localization involved in cell fate determination ; GO:0045167 | traceable author statement <newline> protein localization to cell cortex ; GO:0072697 | inferred from genetic interaction with fray, Lkb1 <newline> anterior/posterior axis specification, embryo ; GO:0008595 | inferred from mutant phenotype [illegible]	basal cortex ; GO:0045180 | inferred from direct assay <newline> centrosome ; GO:0005813 | inferred from direct assay <newline> basal cortex ; GO:0045180 | traceable author statement <newline> apical part of cell ; GO:0045177 | inferred from direct assay <newline> cell cortex ; GO:0005938 | inferred from direct assay <newline> apical part of cell ; GO:0045177 | traceable author statement <newline> basal part of cell ; GO:0045178 | inferred from direct assay <newline> basal plasma membrane ; GO:0009925 | inferred from direct assay <newline> apical cortex ; GO:0045179 | inferred from direct assay <newline> apical plasma membrane ; GO:0016324 | inferred from direct assay <newline> aster ; GO:0005818 | colocalizes_with inferred from direct assay <newline> basal cortex ; GO:0045180 | non-traceable author statement [illegible]	myosin binding ; GO:0017022 | inferred from physical interaction with jar <newline> protein binding ; GO:0005515 | inferred from physical interaction with flfl <newline> protein binding ; GO:0005515 | inferred from physical interaction with stau	Hsap\SLMAP	3R
L1	CG4783	FBgn0038756	CG4783	-	CG4783	-	-	-	-	3R

L1	CG31213	FBgn0051213	CG31213	-	CG31213	transmembrane transport ; GO:0055085 | inferred from electronic annotation with InterPro:IPR026082 <newline> lipid transport ; GO:0006869 | inferred from biological aspect of ancestor with PANTHER:PTN000442469	integral component of membrane ; GO:0016021 | inferred from sequence or structural similarity with UniProtKB:Q9BZC7 <newline> intracellular membrane-bounded organelle ; GO:0043231 | inferred from biological aspect of ancestor with PANTHER:PTN000442469	ATP binding ; GO:0005524 | inferred from electronic annotation with InterPro:IPR003439, InterPro:IPR017871 <newline> ATPase activity ; GO:0016887 | inferred from electronic annotation with InterPro:IPR003439, InterPro:IPR017871 <newline> ATPase-coupled transmembrane transporter activity ; GO:0042626 | inferred from sequence or structural similarity with UniProtKB:O94911 <newline> ATPase-coupled transmembrane transporter activity ; GO:0042626 | inferred from biological aspect of ancestor with PANTHER:PTN000442469 <newline> lipid transporter activity ; GO:0005319 | inferred from biological aspect of ancestor with PANTHER:PTN000442469	Hsap\ABCA12 <newline> Hsap\ABCA13 <newline> Hsap\ABCA9 <newline> Hsap\ABCA2 <newline> Hsap\ABCA1 <newline> Hsap\ABCA5 <newline> Hsap\ABCA8 <newline> Hsap\ABCA3 <newline> Hsap\ABCA10 <newline> Hsap\ABCA4 <newline> Hsap\ABCA6 <newline> Hsap\ABCA7	3R
L1	MED25	FBgn0038760	CG12254	Mediator complex subunit 25	MED25	nervous system development ; GO:0007399 | inferred from mutant phenotype <newline> regulation of transcription by RNA polymerase II ; GO:0006357 | inferred from sequence or structural similarity with UniProtKB:Q9W5P1 <newline> positive regulation of transcription by RNA polymerase II ; GO:0045944 | inferred from biological aspect of ancestor with PANTHER:PTN000277285 <newline> regulation of transcription by RNA polymerase II ; GO:0006357 | inferred from sequence or structural similarity with UniProtKB:Q71SY5 <newline> positive regulation of antibacterial peptide biosynthetic process ; GO:0006963 | inferred from mutant phenotype	nuclear transcription factor complex ; GO:0044798 | inferred from biological aspect of ancestor with PANTHER:PTN000277285 <newline> nucleoplasm ; GO:0005654 | inferred from sequence or structural similarity with UniProtKB:Q71SY5 <newline> mediator complex ; GO:0016592 | inferred from sequence or structural similarity with UniProtKB:Q9W5P1 <newline> mediator complex ; GO:0016592 | inferred from biological aspect of ancestor with PANTHER:PTN000277285	transcription coregulator activity ; GO:0003712 | inferred from sequence or structural similarity with UniProtKB:Q9W5P1	Hsap\MED25 <newline> Hsap\PTOV1	3R

L1	CG17190	FBgn0038761	CG17190	-	CG17190	biological_process ; GO:0008150 | no biological data available	cellular_component ; GO:0005575 | no biological data available	molecular_function ; GO:0003674 | no biological data available	-	3R
L1	CG4836	FBgn0270925	CG4836	-	CG4836	oxidation-reduction process ; GO:0055114 | inferred from electronic annotation with InterPro:IPR002085, InterPro:IPR013154	-	-	Hsap\SORD	3R
L1	CG4433	FBgn0038763	CG4433	Phosphatidylinositol glycan anchor biosynthesis class L	PIG-L	GPI anchor biosynthetic process ; GO:0006506 | inferred from sequence or structural similarity with RGD:620437	cellular_component ; GO:0005575 | no biological data available	N-acetylglucosaminylphosphatidylinositol deacetylase activity ; GO:0000225 | inferred from sequence or structural similarity with RGD:620437	Hsap\PIGL	3R
L1	psidin	FBgn0243511	CG4845	phagocyte signaling impaired	psidin	border follicle cell migration ; GO:0007298 | inferred from mutant phenotype <newline> negative regulation of neuron apoptotic process ; GO:0043524 | inferred from mutant phenotype inferred from genetic interaction with tsr <newline> N-terminal peptidyl-methionine acetylation ; GO:0017196 | inferred from biological aspect of ancestor with PANTHER:PTN000511059 <newline> immune response ; GO:0006955 | inferred from mutant phenotype <newline> sensory neuron axon guidance ; GO:0097374 | inferred from mutant phenotype <newline> positive regulation of lamellipodium assembly ; GO:0010592 | inferred from mutant phenotype <newline> actin filament depolymerization ; GO:0030042 | inferred from genetic interaction with tsr <newline> activation of immune response ; GO:0002253 | inferred from mutant phenotype <newline> phagocytosis, engulfment ; GO:0006911 | inferred	NatB complex ; GO:0031416 | inferred from biological aspect of ancestor with PANTHER:PTN000511060 <newline> lysosome ; GO:0005764 | inferred from direct assay <newline> cytoplasm ; GO:0005737 | inferred from biological aspect of ancestor with PANTHER:PTN000511059 <newline> NatB complex ; GO:0031416 | inferred from physical interaction with Naa20A <newline> neuron projection ; GO:0043005 | inferred from direct assay <newline> growth cone lamellipodium ; GO:1990761 | inferred from direct assay <newline> cytoplasm ; GO:0005737 | inferred from direct assay	actin filament binding ; GO:0051015 | inferred from direct assay <newline> peptide alpha-N-acetyltransferase activity ; GO:0004596 | contributes_to inferred from biological aspect of ancestor with PANTHER:PTN000511059	Hsap\NAA25	3R

L1	CG4424	FBgn0038765	CG4424	-	CG4424	regulation of transcription, DNA-templated ; GO:0006355 | inferred from sequence or structural similarity with UniProtKB:Q05516	nucleus ; GO:0005634 | inferred from biological aspect of ancestor with PANTHER:PTN001227969 <newline> nucleus ; GO:0005634 | inferred from sequence or structural similarity with UniProtKB:Q05516	zinc ion binding ; GO:0008270 | inferred from electronic annotation with InterPro:IPR012934 <newline> transcription regulatory region sequence-specific DNA binding ; GO:0000976 | inferred from sequence or structural similarity with UniProtKB:Q05516 <newline> DNA-binding transcription factor activity ; GO:0003700 | inferred from sequence or structural similarity with UniProtKB:Q05516	Hsap\ZBTB16 <newline> Hsap\ZNF771 <newline> Hsap\ZNF787	3R
L1	CG4854	FBgn0038766	CG4854	-	CG4854	regulation of transcription, DNA-templated ; GO:0006355 | inferred from sequence or structural similarity with UniProtKB:P41696	nucleus ; GO:0005634 | inferred from sequence or structural similarity with UniProtKB:P41696	zinc ion binding ; GO:0008270 | inferred from electronic annotation with InterPro:IPR012934 <newline> transcription regulatory region sequence-specific DNA binding ; GO:0000976 | inferred from sequence or structural similarity with UniProtKB:P41696 <newline> DNA-binding transcription factor activity ; GO:0003700 | inferred from sequence or structural similarity with UniProtKB:P41696	Hsap\ZNF785 <newline> Hsap\ZNF623 <newline> Hsap\ZNF263 <newline> Hsap\ZSCAN4 <newline> Hsap\RBAK-RBAKDN <newline> Hsap\ZSCAN2 <newline> Hsap\ZNF233 <newline> Hsap\ZNF850 <newline> Hsap\ZNF492 <newline> Hsap\ZSCAN32 <newline> Hsap\ZNF792 <newline> Hsap\ZSCAN30 <newline> Hsap\ZNF730 <newline> Hsap\ZNF570 <newline> Hsap\ZNF527 <newline> Hsap\ZNF43 <newline> Hsap\ZNF107 <newline> Hsap\ZNF845 <newline> Hsap\ZNF816 <newline> Hsap\ZNF681 <newline> Hsap\ZNF766 <newline> Hsap\ZNF28 <newline> Hsap\ZNF595 <newline> Hsap\ZSCAN20 <newline> Hsap\ZNF239 <newline> Hsap\ZNF76 <newline> Hsap\ZSCAN1 [illegible]	3R

L1	trem	FBgn0038767	CG4413	trade embargo	trem	meiotic DNA double-strand break formation involved in reciprocal meiotic recombination ; GO:0010780 | inferred from mutant phenotype <newline> meiotic DNA double-strand break processing involved in reciprocal meiotic recombination ; GO:0010705 | inferred from mutant phenotype <newline> meiotic cell cycle ; GO:0051321 | inferred from mutant phenotype	nuclear chromatin ; GO:0000790 | colocalizes_with inferred from direct assay <newline> nucleus ; GO:0005634 | inferred from direct assay	zinc ion binding ; GO:0008270 | inferred from electronic annotation with InterPro:IPR012934 <newline> nucleic acid binding ; GO:0003676 | inferred from electronic annotation with InterPro:IPR013087	Hsap\ZNF527 <newline> Hsap\ZNF708 <newline> Hsap\ZNF766 <newline> Hsap\ZNF239 <newline> Hsap\RBAK-RBAKDN <newline> Hsap\ZNF254 <newline> Hsap\OVOL3 <newline> Hsap\ZNF70 <newline> Hsap\ZNF721 <newline> Hsap\ZSCAN29 <newline> Hsap\ZNF25 <newline> Hsap\ZSCAN2 <newline> Hsap\ZSCAN31 <newline> Hsap\ZNF76 <newline> Hsap\OVOL1 <newline> Hsap\ZNF816 <newline> Hsap\ZNF558 <newline> Hsap\ZNF526 <newline> Hsap\ZNF764 <newline> Hsap\ZNF850 <newline> Hsap\ZFP28 <newline> Hsap\ZNF610 <newline> Hsap\ZNF480 <newline> Hsap\ZNF891 <newline> Hsap\ZNF281 <newline> Hsap\ZNF485 <newline> Hsap\ZSCAN32 [illegible]	3R

L1	CG4936	FBgn0038768	CG4936	-	CG4936	positive regulation of gene expression ; GO:0010628 | inferred from mutant phenotype <newline> regulation of transcription, DNA-templated ; GO:0006355 | inferred from sequence or structural similarity with UniProtKB:P41696	nucleus ; GO:0005634 | inferred from sequence or structural similarity with UniProtKB:P41696	zinc ion binding ; GO:0008270 | inferred from electronic annotation with InterPro:IPR012934 <newline> DNA-binding transcription factor activity ; GO:0003700 | inferred from sequence or structural similarity with UniProtKB:P41696 <newline> transcription regulatory region sequence-specific DNA binding ; GO:0000976 | inferred from sequence or structural similarity with UniProtKB:P41696	Hsap\ZNF611 <newline> Hsap\ZNF850 <newline> Hsap\ZNF852 <newline> Hsap\ZNF764 <newline> Hsap\ZNF527 <newline> Hsap\ZFP62 <newline> Hsap\ZFP28 <newline> Hsap\ZSCAN2 <newline> Hsap\ZNF254 <newline> Hsap\ZNF568 <newline> Hsap\ZNF528 <newline> Hsap\ZSCAN32 <newline> Hsap\ZNF623 <newline> Hsap\ZNF730 <newline> Hsap\ZNF160 <newline> Hsap\ZNF721 <newline> Hsap\ZSCAN4 <newline> Hsap\ZNF570 <newline> Hsap\ZSCAN20 <newline> Hsap\ZNF595 <newline> Hsap\ZNF492 <newline> Hsap\ZNF681 <newline> Hsap\ZNF485 <newline> Hsap\ZNF107 <newline> Hsap\ZNF679 <newline> Hsap\ZNF610 <newline> Hsap\ZNF708 <newline> [illegible]	3R
L1	CG10889	FBgn0038769	CG10889	Regnase 1	Regnase-1	-	-	metal ion binding ; GO:0046872 | inferred from electronic annotation with InterPro:IPR000571	Hsap\KHNYN <newline> Hsap\ZC3H12D <newline> Hsap\NYNRIN <newline> Hsap\ZC3H12B <newline> Hsap\ZC3H12A <newline> Hsap\ZC3H12C <newline> Hsap\CCDC59 <newline> Hsap\N4BP1	3R
L1	CG17193	FBgn0040571	CG17193	-	CG17193	biological_process ; GO:0008150 | no biological data available	cellular_component ; GO:0005575 | no biological data available	molecular_function ; GO:0003674 | no biological data available	-	3R

L1	Indy-2	FBgn0260466	CG33933	I'm not dead yet 2	Indy-2	sodium ion transport ; GO:0006814 | inferred from electronic annotation with InterPro:IPR001898 <newline> transmembrane transport ; GO:0055085 | inferred from electronic annotation with InterPro:IPR001898 <newline> citrate transport ; GO:0015746 | inferred from biological aspect of ancestor with PANTHER:PTN000031937 <newline> pyruvate transport ; GO:0006848 | inferred from sequence or structural similarity with UniProtKB:Q9VVT2 <newline> determination of adult lifespan ; GO:0008340 | inferred from sequence or structural similarity with UniProtKB:Q9VVT2 <newline> succinate transport ; GO:0015744 | inferred from sequence or structural similarity with UniProtKB:Q9VVT2 <newline> citrate transport ; GO:0015746 | inferred from sequence or structural similarity with UniProtKB:Q9VVT2	integral component of membrane ; GO:0016021 | inferred from biological aspect of ancestor with PANTHER:PTN000031793 <newline> integral component of plasma membrane ; GO:0005887 | inferred from sequence or structural similarity with UniProtKB:Q9VVT2	succinate transmembrane transporter activity ; GO:0015141 | inferred from biological aspect of ancestor with PANTHER:PTN000031937 <newline> citrate transmembrane transporter activity ; GO:0015137 | inferred from biological aspect of ancestor with PANTHER:PTN000031937 <newline> succinate transmembrane transporter activity ; GO:0015141 | inferred from sequence or structural similarity with UniProtKB:Q9VVT2 <newline> pyruvate transmembrane transporter activity ; GO:0050833 | inferred from sequence or structural similarity with UniProtKB:Q9VVT2 <newline> citrate transmembrane transporter activity ; GO:0015137 | inferred from sequence or structural similarity with UniProtKB:Q9VVT2	Hsap\SLC13A2 <newline> Hsap\SLC13A3 <newline> Hsap\SLC13A1 <newline> Hsap\SLC13A5 <newline> Hsap\SLC13A4	3R
L1	CG33934	FBgn0064119	CG33934	-	CG33934	sodium ion transport ; GO:0006814 | inferred from electronic annotation with InterPro:IPR001898 <newline> transmembrane transport ; GO:0055085 | inferred from electronic annotation with InterPro:IPR001898 <newline> citrate transport ; GO:0015746 | inferred from biological aspect of ancestor with PANTHER:PTN000031937	integral component of membrane ; GO:0016021 | inferred from biological aspect of ancestor with PANTHER:PTN000031793	succinate transmembrane transporter activity ; GO:0015141 | inferred from biological aspect of ancestor with PANTHER:PTN000031937 <newline> citrate transmembrane transporter activity ; GO:0015137 | inferred from biological aspect of ancestor with PANTHER:PTN000031937	Hsap\SLC13A5 <newline> Hsap\SLC13A1 <newline> Hsap\SLC13A3 <newline> Hsap\SLC13A4 <newline> Hsap\SLC13A2	3R
L1	CG4390	FBgn0038771	CG4390	-	CG4390	formaldehyde catabolic process ; GO:0046294 | inferred from electronic annotation with InterPro:IPR014186	cytosol ; GO:0005829 | inferred from biological aspect of ancestor with PANTHER:PTN000006872	S-formylglutathione hydrolase activity ; GO:0018738 | inferred from biological aspect of ancestor with PANTHER:PTN000006872	Hsap\CCDC39 <newline> Hsap\ESD	3R

L1	CG4973	FBgn0038772	CG4973	midlife crisis	mdlc	snoRNA splicing ; GO:0034247 | inferred from biological aspect of ancestor with PANTHER:PTN000309255 <newline> neuron differentiation ; GO:0030182 | inferred from mutant phenotype <newline> mRNA splicing, via spliceosome ; GO:0000398 | inferred from sequence or structural similarity with SGD:S000004315 <newline> regulation of RNA splicing ; GO:0043484 | inferred from direct assay	U2-type spliceosomal complex ; GO:0005684 | inferred from sequence or structural similarity with SGD:S000004315 <newline> U2-type spliceosomal complex ; GO:0005684 | inferred from biological aspect of ancestor with PANTHER:PTN000309255 <newline> nucleus ; GO:0005634 | inferred from direct assay	metal ion binding ; GO:0046872 | inferred from electronic annotation with InterPro:IPR000571 <newline> zinc ion binding ; GO:0008270 | inferred from sequence model	Hsap\NAA50 <newline> Hsap\RNF113B <newline> Hsap\RNF113A	3R
L1	Rh3	FBgn0003249	CG10888	Rhodopsin 3	Rh3	visual perception ; GO:0007601 | inferred from electronic annotation with InterPro:IPR000856 <newline> phototransduction, UV ; GO:0007604 | traceable author statement <newline> absorption of UV light ; GO:0016039 | inferred from mutant phenotype <newline> phototransduction, UV ; GO:0007604 | non-traceable author statement <newline> G protein-coupled receptor signaling pathway ; GO:0007186 | inferred from sequence or structural similarity <newline> detection of UV ; GO:0009589 | traceable author statement <newline> phototransduction, UV ; GO:0007604 | inferred from direct assay <newline> cellular response to light stimulus ; GO:0071482 | inferred from biological aspect of ancestor with PANTHER:PTN000662115 <newline> phototransduction ; GO:0007602 | inferred from biological aspect of ancestor with	integral component of plasma membrane ; GO:0005887 | inferred from biological aspect of ancestor with PANTHER:PTN000662115 <newline> integral component of membrane ; GO:0016021 | inferred from sequence or structural similarity	G protein-coupled photoreceptor activity ; GO:0008020 | traceable author statement <newline> G protein-coupled photoreceptor activity ; GO:0008020 | non-traceable author statement <newline> G protein-coupled photoreceptor activity ; GO:0008020 | inferred from biological aspect of ancestor with PANTHER:PTN000662115 <newline> G protein-coupled photoreceptor activity ; GO:0008020 | inferred from sequence or structural similarity <newline> G protein-coupled photoreceptor activity ; GO:0008020 | inferred from direct assay <newline> G protein-coupled photoreceptor activity ; GO:0008020 | inferred from sequence or structural similarity with ninaE	Hsap\OPN1LW <newline> Hsap\OPN3 <newline> Hsap\RGR <newline> Hsap\OPN1MW <newline> Hsap\RRH <newline> Hsap\OPN1SW <newline> Hsap\OPN5 <newline> Hsap\OPN1MW2 <newline> Hsap\RHO <newline> Hsap\OPN1MW3 <newline> Hsap\OPN4	3R
L1	CG31206	FBgn0051206	CG31206	-	CG31206	biological_process ; GO:0008150 | no biological data available	cellular_component ; GO:0005575 | no biological data available	molecular_function ; GO:0003674 | no biological data available	-	3R

L1	CG10887	FBgn0038773	CG10887	-	CG10887	histone modification ; GO:0016570 | inferred from electronic annotation with InterPro:IPR007149 <newline> positive regulation of transcription elongation from RNA polymerase II promoter ; GO:0032968 | inferred from biological aspect of ancestor with PANTHER:PTN000567381	Cdc73/Paf1 complex ; GO:0016593 | inferred from electronic annotation with InterPro:IPR007149 <newline> nucleus ; GO:0005634 | inferred from biological aspect of ancestor with PANTHER:PTN000567381	RNA polymerase II C-terminal domain phosphoserine binding ; GO:1990269 | inferred from biological aspect of ancestor with PANTHER:PTN000567381	Hsap\LEO1	3R
L1	Gr92a	FBgn0045471	CG31208	Gustatory receptor 92a	Gr92a	sensory perception of taste ; GO:0050909 | inferred from electronic annotation with InterPro:IPR013604 <newline> sensory perception of taste ; GO:0050909 | non-traceable author statement	integral component of membrane ; GO:0016021 | inferred from electronic annotation with InterPro:IPR013604 <newline> dendrite ; GO:0030425 | inferred from biological aspect of ancestor with PANTHER:PTN000475107 <newline> integral component of membrane ; GO:0016021 | non-traceable author statement <newline> neuronal cell body ; GO:0043025 | inferred from biological aspect of ancestor with PANTHER:PTN000475107 <newline> axon ; GO:0030424 | inferred from biological aspect of ancestor with PANTHER:PTN000475107	taste receptor activity ; GO:0008527 | inferred from sequence or structural similarity with Gr5a <newline> taste receptor activity ; GO:0008527 | non-traceable author statement <newline> sweet taste receptor activity ; GO:0033041 | inferred from biological aspect of ancestor with PANTHER:PTN002721890	-	3R
L1	CG5023	FBgn0038774	CG5023	-	CG5023	actomyosin structure organization ; GO:0031032 | inferred from electronic annotation with InterPro:IPR001997	-	actin binding ; GO:0003779 | inferred from electronic annotation with InterPro:IPR001997 <newline> actin binding ; GO:0003779 | inferred from sequence or structural similarity	Hsap\CNN1 <newline> Hsap\CNN3 <newline> Hsap\TAGLN <newline> Hsap\CNN2 <newline> Hsap\TAGLN2 <newline> Hsap\TAGLN3 <newline> Hsap\IQGAP1 <newline> Hsap\IQGAP3	3R

L1	CG34139	FBgn0083975	CG34139	Neuroligin 4	Nlg4	synaptic vesicle endocytosis ; GO:0048488 | inferred from biological aspect of ancestor with PANTHER:PTN000168538 <newline> sleep ; GO:0030431 | inferred from mutant phenotype <newline> synaptic transmission, GABAergic ; GO:0051932 | inferred from direct assay <newline> neuron cell-cell adhesion ; GO:0007158 | inferred from biological aspect of ancestor with PANTHER:PTN000168538 <newline> modulation of chemical synaptic transmission ; GO:0050804 | inferred from biological aspect of ancestor with PANTHER:PTN000168538 <newline> postsynaptic membrane assembly ; GO:0097104 | inferred from biological aspect of ancestor with PANTHER:PTN000168538 <newline> presynaptic membrane assembly ; GO:0097105 | inferred from biological aspect of ancestor with PANTHER:PTN000168538	integral component of plasma membrane ; GO:0005887 | inferred from biological aspect of ancestor with PANTHER:PTN000168538 <newline> cell surface ; GO:0009986 | inferred from biological aspect of ancestor with PANTHER:PTN000168538 <newline> synapse ; GO:0045202 | inferred from biological aspect of ancestor with PANTHER:PTN000168538	signaling receptor activity ; GO:0038023 | inferred from biological aspect of ancestor with PANTHER:PTN000168538 <newline> neurexin family protein binding ; GO:0042043 | inferred from biological aspect of ancestor with PANTHER:PTN000168538	Hsap\NLGN2 <newline> Hsap\CEL <newline> Hsap\CES2 <newline> Hsap\CES4A <newline> Hsap\NLGN1 <newline> Hsap\CES1 <newline> Hsap\NLGN4Y <newline> Hsap\CES5A <newline> Hsap\NLGN4X <newline> Hsap\NLGN3 <newline> Hsap\CES3 <newline> Hsap\BCHE	3R
L1	CG17199	FBgn0038775	CG17199	-	CG17199	oxidation-reduction process ; GO:0055114 | inferred from electronic annotation with InterPro:IPR003767	-	oxidoreductase activity ; GO:0016491 | inferred from electronic annotation with InterPro:IPR003767	-	3R

L1	cic	FBgn0262582	CG43122	capicua	cic	negative regulation of transcription by RNA polymerase II ; GO:0000122 | inferred from direct assay <newline> negative regulation of cell growth ; GO:0030308 | inferred from mutant phenotype <newline> eye morphogenesis ; GO:0048592 | inferred from mutant phenotype <newline> terminal region determination ; GO:0007362 | inferred from mutant phenotype <newline> imaginal disc-derived wing vein specification ; GO:0007474 | inferred from mutant phenotype <newline> epidermal growth factor receptor signaling pathway ; GO:0007173 | inferred from mutant phenotype <newline> dorsal appendage formation ; GO:0046843 | inferred from mutant phenotype <newline> negative regulation of transcription by RNA polymerase II ; GO:0000122 | inferred from biological aspect of ancestor with PANTHER:PTN000316564 <newline> negative regulation of transcription	nucleus ; GO:0005634 | inferred from direct assay <newline> cytosol ; GO:0005829 | inferred from direct assay <newline> nucleus ; GO:0005634 | inferred from biological aspect of ancestor with PANTHER:PTN000316564	DNA-binding transcription repressor activity, RNA polymerase II-specific ; GO:0001227 | inferred from mutant phenotype <newline> repressing transcription factor binding ; GO:0070491 | inferred from physical interaction with gro <newline> RNA polymerase II regulatory region sequence-specific DNA binding ; GO:0000977 | inferred from direct assay <newline> sequence-specific DNA binding ; GO:0043565 | inferred from direct assay	Hsap\SOX7 <newline> Hsap\SOX6 <newline> Hsap\SOX2 <newline> Hsap\SOX21 <newline> Hsap\CIC <newline> Hsap\SOX14 <newline> Hsap\HBP1 <newline> Hsap\SOX4 <newline> Hsap\SOX5 <newline> Hsap\SOX17 <newline> Hsap\SOX18 <newline> Hsap\SRY <newline> Hsap\SOX30 <newline> Hsap\SOX1 <newline> Hsap\SOX12 <newline> Hsap\SOX3 <newline> Hsap\SOX11 <newline> Hsap\SOX13 <newline> Hsap\SOX15	3R
L1	CG4367	FBgn0038783	CG4367	-	CG4367	-	-	-	-	3R
L1	CG4362	FBgn0038784	CG4362	-	CG4362	-	-	-	-	3R
L1	CG42668	FBgn0261550	CG42668	-	CG42668	phospholipid transport ; GO:0015914 | inferred from biological aspect of ancestor with PANTHER:PTN000861426	membrane ; GO:0016020 | inferred from biological aspect of ancestor with PANTHER:PTN000104007 <newline> intracellular membrane-bounded organelle ; GO:0043231 | inferred from biological aspect of ancestor with PANTHER:PTN000104007 <newline> cytosol ; GO:0005829 | inferred from biological aspect of ancestor with PANTHER:PTN000104007	cholesterol binding ; GO:0015485 | inferred from biological aspect of ancestor with PANTHER:PTN000861474 <newline> sterol binding ; GO:0032934 | inferred from biological aspect of ancestor with PANTHER:PTN000104007 <newline> lipid binding ; GO:0008289 | inferred from biological aspect of ancestor with PANTHER:PTN000104007	Hsap\OSBPL11 <newline> Hsap\OSBPL10 <newline> Hsap\OSBPL5 <newline> Hsap\OSBPL9 <newline> Hsap\OSBPL8	3R

L1	CG4360	FBgn0038787	CG4360	-	CG4360	negative regulation of transcription by RNA polymerase II ; GO:0000122 | inferred from sequence or structural similarity with UniProtKB:Q9H165	nucleus ; GO:0005634 | inferred from sequence or structural similarity with UniProtKB:Q9H165-2	RNA polymerase II proximal promoter sequence-specific DNA binding ; GO:0000978 | inferred from sequence or structural similarity with UniProtKB:Q9H165 <newline> DNA-binding transcription repressor activity, RNA polymerase II-specific ; GO:0001227 | inferred from sequence or structural similarity with UniProtKB:Q9H165	Hsap\ZNF48 <newline> Hsap\ZNF254 <newline> Hsap\ZNF726 <newline> Hsap\BCL11A <newline> Hsap\ZNF681 <newline> Hsap\ZNF728 <newline> Hsap\ZNF551 <newline> Hsap\ZKSCAN5 <newline> Hsap\ZNF626 <newline> Hsap\ZNF93 <newline> Hsap\ZNF595 <newline> Hsap\RBAK-RBAKDN <newline> Hsap\ZNF429 <newline> Hsap\ZNF296	3R
L1	Sirt2	FBgn0038788	CG5085	Sirtuin 2	Sirt2	protein deacetylation ; GO:0006476 | inferred from direct assay <newline> histone deacetylation ; GO:0016575 | inferred from mutant phenotype <newline> determination of adult lifespan ; GO:0008340 | inferred from mutant phenotype	cytoplasm ; GO:0005737 | inferred from sequence or structural similarity with HGNC:10886 <newline> nucleus ; GO:0005634 | inferred from biological aspect of ancestor with PANTHER:PTN000119159 <newline> nucleus ; GO:0005634 | inferred from sequence or structural similarity with HGNC:10886 <newline> cytoplasm ; GO:0005737 | inferred from biological aspect of ancestor with PANTHER:PTN000119159	zinc ion binding ; GO:0008270 | inferred from electronic annotation with InterPro:IPR017328 <newline> NAD-dependent histone deacetylase activity ; GO:0017136 | inferred from direct assay <newline> NAD+ binding ; GO:0070403 | inferred from biological aspect of ancestor with PANTHER:PTN000119154 <newline> histone deacetylase activity ; GO:0004407 | inferred from mutant phenotype <newline> NAD-dependent histone deacetylase activity ; GO:0017136 | inferred from biological aspect of ancestor with PANTHER:PTN000119154	Hsap\SIRT2 <newline> Hsap\SIRT1 <newline> Hsap\SIRT3	3R

L1	Ir92a	FBgn0038789	CG15685	Ionotropic receptor 92a	Ir92a	detection of chemical stimulus involved in sensory perception of smell ; GO:0050911 | inferred from direct assay <newline> cellular response to ammonia ; GO:1903718 | inferred from direct assay <newline> detection of chemical stimulus involved in sensory perception of smell ; GO:0050911 | inferred from mutant phenotype <newline> positive regulation of calcium-mediated signaling ; GO:0050850 | inferred from direct assay <newline> cellular response to amine stimulus ; GO:0071418 | inferred from direct assay	membrane ; GO:0016020 | inferred from electronic annotation with InterPro:IPR001320 <newline> integral component of membrane ; GO:0016021 | inferred from sequence model	ionotropic glutamate receptor activity ; GO:0004970 | inferred from electronic annotation with InterPro:IPR001320 <newline> extracellular ammonia-gated ion channel activity ; GO:0036081 | inferred from direct assay <newline> olfactory receptor activity ; GO:0004984 | inferred from direct assay	Hsap\GRID2	3R
L1	MtnC	FBgn0038790	CG5097	Metallothionein C	MtnC	response to metal ion ; GO:0010038 | inferred from direct assay <newline> metal ion homeostasis ; GO:0055065 | inferred from mutant phenotype <newline> response to metal ion ; GO:0010038 | inferred from sequence or structural similarity with MtnE	cellular_component ; GO:0005575 | no biological data available	metal ion binding ; GO:0046872 | inferred from direct assay	-	3R
L1	CG43224	FBgn0262869	CG43224	Glial cell line-derived neurotrophic family receptor-like	Gfrl	nervous system development ; GO:0007399 | inferred from biological aspect of ancestor with PANTHER:PTN001306067	receptor complex ; GO:0043235 | inferred from biological aspect of ancestor with PANTHER:PTN001306067 <newline> external side of plasma membrane ; GO:0009897 | inferred from biological aspect of ancestor with PANTHER:PTN001306067 <newline> anchored component of external side of plasma membrane ; GO:0031362 | inferred from direct assay	signaling receptor activity ; GO:0038023 | inferred from electronic annotation with InterPro:IPR003438 <newline> cell adhesion molecule binding ; GO:0050839 | inferred from physical interaction with Fas2	Hsap\GFRA1 <newline> Hsap\GFRAL <newline> Hsap\GFRA3 <newline> Hsap\GFRA4 <newline> Hsap\GFRA2	3R
L1	CG4335	FBgn0038795	CG4335	-	CG4335	oxidation-reduction process ; GO:0055114 | inferred from electronic annotation with InterPro:IPR003819, InterPro:IPR012776 <newline> carnitine biosynthetic process ; GO:0045329 | inferred from biological aspect of ancestor with PANTHER:PTN000075473	mitochondrion ; GO:0005739 | inferred from biological aspect of ancestor with PANTHER:PTN000075473 <newline> mitochondrion ; GO:0005739 | inferred from sequence or structural similarity with UniProtKB:Q9NVH6-1	iron ion binding ; GO:0005506 | inferred from electronic annotation with InterPro:IPR012776 <newline> trimethyllysine dioxygenase activity ; GO:0050353 | inferred from sequence or structural similarity with UniProtKB:Q9NVH6-1	Hsap\BBOX1 <newline> Hsap\TMLHE	3R

L1	CG10881	FBgn0038796	CG10881	eukaryotic translation initiation factor 3 subunit g2	eIF3g2	translational initiation ; GO:0006413 | inferred from sequence or structural similarity	eukaryotic translation initiation factor 3 complex ; GO:0005852 | inferred from electronic annotation with InterPro:IPR017334 <newline> eukaryotic translation initiation factor 3 complex ; GO:0005852 | inferred from sequence or structural similarity	translation initiation factor activity ; GO:0003743 | inferred from electronic annotation with InterPro:IPR017334 <newline> mRNA binding ; GO:0003729 | inferred from sequence or structural similarity <newline> translation initiation factor activity ; GO:0003743 | inferred from sequence or structural similarity	Hsap\EIF3G	3R
L1	Dic2	FBgn0038797	CG4323	Dicarboxylate carrier 2	Dic2	thiosulfate transport ; GO:0015709 | inferred from biological aspect of ancestor with PANTHER:PTN000756618 <newline> phosphate ion transmembrane transport ; GO:0035435 | inferred from biological aspect of ancestor with PANTHER:PTN000756618 <newline> succinate transmembrane transport ; GO:0071422 | inferred from biological aspect of ancestor with PANTHER:PTN000756618 <newline> sulfate transport ; GO:0008272 | inferred from biological aspect of ancestor with PANTHER:PTN000756618 <newline> malate transmembrane transport ; GO:0071423 | inferred from biological aspect of ancestor with PANTHER:PTN000756618 <newline> oxaloacetate transport ; GO:0015729 | inferred from biological aspect of ancestor with PANTHER:PTN000756618	mitochondrion ; GO:0005739 | inferred from direct assay	sulfate transmembrane transporter activity ; GO:0015116 | inferred from biological aspect of ancestor with PANTHER:PTN000756618 <newline> antiporter activity ; GO:0015297 | inferred from biological aspect of ancestor with PANTHER:PTN000756618 <newline> succinate transmembrane transporter activity ; GO:0015141 | inferred from biological aspect of ancestor with PANTHER:PTN000756618 <newline> oxaloacetate transmembrane transporter activity ; GO:0015131 | inferred from biological aspect of ancestor with PANTHER:PTN000756618 <newline> malate transmembrane transporter activity ; GO:0015140 | inferred from biological aspect of ancestor with PANTHER:PTN000756618 <newline> thiosulfate transmembrane transporter activity ; GO:0015117 | inferred [illegible]	Hsap\SLC25A11 <newline> Hsap\SLC25A10 <newline> Hsap\MRPL12	3R
L1	MtnB	FBgn0002869	CG4312	Metallothionein B	MtnB	response to metal ion ; GO:0010038 | inferred from direct assay <newline> metal ion homeostasis ; GO:0055065 | inferred from mutant phenotype <newline> response to metal ion ; GO:0010038 | inferred from sequence or structural similarity with MtnE	cellular_component ; GO:0005575 | no biological data available	copper ion binding ; GO:0005507 | inferred from direct assay <newline> metal ion binding ; GO:0046872 | inferred from direct assay	-	3R

L1	Or92a	FBgn0038798	CG17916	Odorant receptor 92a	Or92a	detection of chemical stimulus involved in sensory perception of smell ; GO:0050911 | inferred from biological aspect of ancestor with PANTHER:PTN000474402	dendrite membrane ; GO:0032590 | inferred from sequence or structural similarity with Or22a <newline> plasma membrane ; GO:0005886 | inferred from biological aspect of ancestor with PANTHER:PTN000474402 <newline> integral component of membrane ; GO:0016021 | inferred from biological aspect of ancestor with PANTHER:PTN000474402	odorant binding ; GO:0005549 | inferred from sequence or structural similarity with Or43b <newline> olfactory receptor activity ; GO:0004984 | inferred from biological aspect of ancestor with PANTHER:PTN000474402	-	3R
L1	CG4288	FBgn0038799	CG4288	Major Facilitator Superfamily Transporter 9	MFS9	transmembrane transport ; GO:0055085 | inferred from electronic annotation with InterPro:IPR011701	integral component of membrane ; GO:0016021 | inferred from biological aspect of ancestor with PANTHER:PTN000184222	-	Hsap\SLC17A8 <newline> Hsap\SLC17A2 <newline> Hsap\SLC17A5 <newline> Hsap\SLC17A1 <newline> Hsap\SLC17A4 <newline> Hsap\SLC17A7 <newline> Hsap\SLC17A3 <newline> Hsap\SLC17A9 <newline> Hsap\SLC17A6	3R
L1	MtnE	FBgn0262146	CG42872	Metallothionein E	MtnE	response to copper ion ; GO:0046688 | inferred from direct assay <newline> detoxification of cadmium ion ; GO:0071585 | inferred from direct assay <newline> response to silver ion ; GO:0010272 | inferred from direct assay <newline> response to mercury ion ; GO:0046689 | inferred from direct assay <newline> response to zinc ion ; GO:0010043 | inferred from direct assay	cellular_component ; GO:0005575 | no biological data available	metal ion binding ; GO:0046872 | inferred from direct assay	-	3R
L1	MtnD	FBgn0053192	CG33192	Metallothionein D	MtnD	response to metal ion ; GO:0010038 | inferred from sequence or structural similarity with MtnE <newline> metal ion homeostasis ; GO:0055065 | inferred from mutant phenotype <newline> response to metal ion ; GO:0010038 | inferred from direct assay <newline> response to copper ion ; GO:0046688 | inferred from direct assay	cellular_component ; GO:0005575 | no biological data available	metal ion binding ; GO:0046872 | inferred from direct assay	-	3R

L1	Stat92E	FBgn0016917	CG4257	Signal-transducer and activator of transcription protein at 92E	Stat92E	stem cell division ; GO:0017145 | inferred from mutant phenotype <newline> lymph gland development ; GO:0048542 | inferred from mutant phenotype <newline> negative regulation of antimicrobial humoral response ; GO:0008348 | inferred from mutant phenotype inferred from genetic interaction with Rel <newline> oogenesis ; GO:0048477 | inferred from mutant phenotype <newline> positive regulation of defense response to virus by host ; GO:0002230 | inferred from mutant phenotype <newline> blastoderm segmentation ; GO:0007350 | inferred from mutant phenotype <newline> border follicle cell migration ; GO:0007298 | inferred from mutant phenotype <newline> cell dedifferentiation ; GO:0043697 | inferred from expression pattern <newline> compound eye development ; GO:0048749 | traceable author statement <newline> defense response ; [illegible]	cytosol ; GO:0005829 | inferred from biological aspect of ancestor with PANTHER:PTN000210448 <newline> nucleus ; GO:0005634 | inferred from direct assay <newline> cytoplasm ; GO:0005737 | inferred from direct assay <newline> cytoplasmic side of apical plasma membrane ; GO:0098592 | inferred from direct assay <newline> cytosol ; GO:0005829 | inferred from direct assay <newline> nucleus ; GO:0005634 | inferred from biological aspect of ancestor with PANTHER:PTN000210448	DNA-binding transcription activator activity, RNA polymerase II-specific ; GO:0001228 | inferred from biological aspect of ancestor with PANTHER:PTN000210448 <newline> RNA polymerase II proximal promoter sequence-specific DNA binding ; GO:0000978 | inferred from biological aspect of ancestor with PANTHER:PTN000210448 <newline> protein binding ; GO:0005515 | inferred from physical interaction with UniProtKB:A1Z7P5 <newline> DNA-binding transcription activator activity, RNA polymerase II-specific ; GO:0001228 | inferred from direct assay <newline> cytokine receptor binding ; GO:0005126 | inferred from physical interaction with UniProtKB:M9NE35 inferred from physical interaction with UniProtKB:Q9VWE0 <newline> histone binding ; GO:0042393 | inferred from direct assay <newline> DNA binding ; [illegible]	Hsap\STAT3 <newline> Hsap\STAT1 <newline> Hsap\STAT2 <newline> Hsap\STAT5B <newline> Hsap\STAT4 <newline> Hsap\STAT5A <newline> Hsap\STAT6	3R
L1	CG4241	FBgn0067783	CG4241	Dephosphocoenzyme A carrier	DPCoAC	mitochondrial transmembrane transport ; GO:1990542 | inferred from direct assay <newline> defense response to Gram-negative bacterium ; GO:0050829 | inferred from high throughput mutant phenotype <newline> positive regulation of innate immune response ; GO:0045089 | inferred from high throughput mutant phenotype	mitochondrial inner membrane ; GO:0005743 | inferred from electronic annotation with InterPro:IPR002167 <newline> integral component of membrane ; GO:0016021 | inferred from sequence model <newline> integral component of mitochondrial membrane ; GO:0032592 | inferred by curator from GO:0015297,GO:1990542	antiporter activity ; GO:0015297 | inferred from direct assay	Hsap\SLC25A43 <newline> Hsap\SLC25A25 <newline> Hsap\SLC25A24 <newline> Hsap\SLC25A23 <newline> Hsap\SLC25A16 <newline> Hsap\SLC25A41 <newline> Hsap\SLC25A42 <newline> Hsap\SLC25A19	3R
L1	att-ORFB	FBgn0067782	CG33488	alternative testis transcripts open reading frame B	att-ORFB	biological_process ; GO:0008150 | no biological data available	cellular_component ; GO:0005575 | no biological data available	molecular_function ; GO:0003674 | no biological data available	-	3R
L1	CG5180	FBgn0043457	CG5180	-	CG5180	-	-	-	-	3R
L1	CG15922	FBgn0040575	CG15922	-	CG15922	-	-	-	-	3R
L1	CG5191	FBgn0038803	CG5191	-	CG5191	-	-	-	Hsap\FAAH2 <newline> Hsap\FAAH	3R

L1	CG10877	FBgn0038804	CG10877	-	CG10877	-	-	succinate-hydroxymethylglutarate CoA-transferase activity ; GO:0047369 | inferred from sequence or structural similarity with UniProtKB:Q9HAC7	Hsap\SUGCT <newline> Hsap\AMACR	3R
L1	TFAM	FBgn0038805	CG4217	mitochondrial transcription factor A	TFAM	positive regulation of transcription, DNA-templated ; GO:0045893 | inferred from sequence or structural similarity with UniProtKB:Q00059 <newline> response to oxidative stress ; GO:0006979 | inferred from direct assay	mitochondrion ; GO:0005739 | inferred from high throughput direct assay <newline> mitochondrion ; GO:0005739 | inferred from sequence or structural similarity with UniProtKB:Q00059 <newline> mitochondrion ; GO:0005739 | inferred from direct assay	DNA-binding transcription factor activity ; GO:0003700 | inferred from sequence or structural similarity with UniProtKB:Q00059	Hsap\TOX2 <newline> Hsap\HMGB3 <newline> Hsap\SSRP1 <newline> Hsap\HMG20B <newline> Hsap\TOX <newline> Hsap\TFAM <newline> Hsap\TOX3 <newline> Hsap\HMG20A <newline> Hsap\UBTF <newline> Hsap\TOX4 <newline> Hsap\HMGB2 <newline> Hsap\HMGB1 <newline> Hsap\HMGB4	3R
L1	CG5412	FBgn0038806	CG5412	-	CG5412	response to retinoic acid ; GO:0032526 | inferred from biological aspect of ancestor with PANTHER:PTN001103703	nucleus ; GO:0005634 | inferred from biological aspect of ancestor with PANTHER:PTN000512658 <newline> cytoplasm ; GO:0005737 | inferred from biological aspect of ancestor with PANTHER:PTN000512658	-	Hsap\OVCA2 <newline> Hsap\PAK6 <newline> Hsap\DHFR	3R
L1	CG4204	FBgn0023212	CG4204	Elongin B	EloB	transcription elongation from RNA polymerase II promoter ; GO:0006368 | inferred from electronic annotation with InterPro:IPR039049 <newline> negative regulation of receptor signaling pathway via JAK-STAT ; GO:0046426 | inferred from mutant phenotype <newline> imaginal disc-derived wing vein specification ; GO:0007474 | inferred from mutant phenotype inferred from genetic interaction with corto <newline> larval somatic muscle development ; GO:0007526 | inferred from expression pattern inferred from mutant phenotype	elongin complex ; GO:0070449 | inferred from direct assay <newline> VCB complex ; GO:0030891 | inferred from biological aspect of ancestor with PANTHER:PTN000997498 <newline> elongin complex ; GO:0070449 | inferred from biological aspect of ancestor with PANTHER:PTN000997498	-	Hsap\ELOB	3R

L1	Srp14	FBgn0038808	CG5417	Signal recognition particle protein 14	Srp14	SRP-dependent cotranslational protein targeting to membrane ; GO:0006614 | inferred from electronic annotation with InterPro:IPR003210, InterPro:IPR009018 <newline> protein targeting to ER ; GO:0045047 | inferred from biological aspect of ancestor with PANTHER:PTN000245647	signal recognition particle, endoplasmic reticulum targeting ; GO:0005786 | inferred from biological aspect of ancestor with PANTHER:PTN000245647	endoplasmic reticulum signal peptide binding ; GO:0030942 | inferred from electronic annotation with InterPro:IPR003210 <newline> 7S RNA binding ; GO:0008312 | inferred from electronic annotation with InterPro:IPR003210, InterPro:IPR009018	Hsap\SRP14	3R
L1	CG34008	FBgn0054008	CG34008	-	CG34008	biological_process ; GO:0008150 | no biological data available	cellular_component ; GO:0005575 | no biological data available	molecular_function ; GO:0003674 | no biological data available	-	3R
L1	CG16953	FBgn0038809	CG16953	-	CG16953	-	-	-	-	3R
L1	CG4173	FBgn0014029	CG4173	Septin 2	43710	cellularization ; GO:0007349 | traceable author statement <newline> growth of a germarium-derived egg chamber ; GO:0007295 | inferred from mutant phenotype <newline> imaginal disc development ; GO:0007444 | inferred from genetic interaction with Sep5 <newline> mitotic cytokinesis ; GO:0000281 | inferred from mutant phenotype <newline> regulation of cell cycle ; GO:0051726 | inferred from genetic interaction with Sep5 <newline> cytoskeleton-dependent cytokinesis ; GO:0061640 | inferred from biological aspect of ancestor with PANTHER:PTN000430171	septin complex ; GO:0031105 | inferred from direct assay <newline> septin ring ; GO:0005940 | inferred from biological aspect of ancestor with PANTHER:PTN000430171 <newline> septin complex ; GO:0031105 | inferred from biological aspect of ancestor with PANTHER:PTN000430171 <newline> septin complex ; GO:0031105 | inferred from physical interaction with pnut, Sep2 <newline> septin complex ; GO:0031105 | inferred from physical interaction with Sep1, pnut <newline> microtubule cytoskeleton ; GO:0015630 | inferred from biological aspect of ancestor with PANTHER:PTN000430171	GTP binding ; GO:0005525 | inferred from electronic annotation with InterPro:IPR016491, InterPro:IPR030379 <newline> GTPase activity ; GO:0003924 | inferred from biological aspect of ancestor with PANTHER:PTN000430171 <newline> GTPase activity ; GO:0003924 | NOT inferred from direct assay <newline> protein binding ; GO:0005515 | inferred from physical interaction with sip2 <newline> GTPase activity ; GO:0003924 | inferred from direct assay <newline> protein homodimerization activity ; GO:0042803 | inferred from direct assay <newline> GTPase activity ; GO:0003924 | inferred from sequence or structural similarity with pnut	Hsap\SEPT5 <newline> Hsap\SEPT11 <newline> Hsap\SEPT10 <newline> Hsap\SEPT12 <newline> Hsap\TMEM250 <newline> Hsap\SEPT2 <newline> Hsap\SEPT8 <newline> Hsap\SEPT4 <newline> Hsap\SEPT7 <newline> Hsap\SEPT3 <newline> Hsap\SEPT9 <newline> Hsap\SEPT1 <newline> Hsap\SEPT14 <newline> Hsap\SEPT6	3R
L1	Srp72	FBgn0038810	CG5434	Signal recognition particle protein 72	Srp72	SRP-dependent cotranslational protein targeting to membrane ; GO:0006614 | inferred from electronic annotation with InterPro:IPR013699, InterPro:IPR026270 <newline> dsRNA transport ; GO:0033227 | inferred from mutant phenotype	signal recognition particle, endoplasmic reticulum targeting ; GO:0005786 | inferred from biological aspect of ancestor with PANTHER:PTN000363482	ribosome binding ; GO:0043022 | contributes_to inferred from biological aspect of ancestor with PANTHER:PTN000363482 <newline> 7S RNA binding ; GO:0008312 | inferred from biological aspect of ancestor with PANTHER:PTN000363482	Hsap\SRP72	3R

L1	CG4159	FBgn0038811	CG4159	Pseudouridine synthase 1	Pus1	mRNA pseudouridine synthesis ; GO:1990481 | inferred from biological aspect of ancestor with PANTHER:PTN000124736 <newline> tRNA pseudouridine synthesis ; GO:0031119 | inferred from biological aspect of ancestor with PANTHER:PTN000124732	nucleus ; GO:0005634 | inferred from biological aspect of ancestor with PANTHER:PTN000124736	RNA binding ; GO:0003723 | inferred from electronic annotation with InterPro:IPR001406, InterPro:IPR020094, InterPro:IPR020095, InterPro:IPR020097, InterPro:IPR020103 <newline> pseudouridine synthase activity ; GO:0009982 | inferred from biological aspect of ancestor with PANTHER:PTN000124732	Hsap\PUS1	3R
L1	bon	FBgn0023097	CG5206	bonus	bon	chromatin organization ; GO:0006325 | inferred from mutant phenotype <newline> axon guidance ; GO:0007411 | inferred from mutant phenotype <newline> protein ubiquitination ; GO:0016567 | inferred from sequence or structural similarity with HGNC:11812	nuclear chromatin ; GO:0000790 | colocalizes_with inferred from direct assay <newline> nucleus ; GO:0005634 | inferred from direct assay	zinc ion binding ; GO:0008270 | inferred from electronic annotation with InterPro:IPR000315, InterPro:IPR001841, InterPro:IPR001965 <newline> ubiquitin-protein transferase activity ; GO:0004842 | inferred from sequence or structural similarity with HGNC:11812 <newline> zinc ion binding ; GO:0008270 | inferred from sequence model <newline> chromatin binding ; GO:0003682 | inferred from direct assay	Hsap\TRIM66 <newline> Hsap\TRIM28 <newline> Hsap\SP140L <newline> Hsap\SP140 <newline> Hsap\TRIM24 <newline> Hsap\PML <newline> Hsap\TRIM33 <newline> Hsap\AIRE <newline> Hsap\TRIM56	3R
L1	CR31506	FBgn0051506	CR31506	transfer RNA:Leucine-TAA 1-1	tRNA:Leu-TAA-1-1	translation ; GO:0006412 | inferred by curator from GO:0033403	cytosol ; GO:0005829 | inferred by curator from GO:0033403	UUA codon-amino acid adaptor activity ; GO:0033403 | inferred from sequence model	-	3R
L1	CG15923	FBgn0038814	CG15923	-	CG15923	negative regulation of centrosome duplication ; GO:0010826 | inferred from electronic annotation with InterPro:IPR019170 <newline> cilium assembly ; GO:0060271 | inferred from electronic annotation with InterPro:IPR019170	MKS complex ; GO:0036038 | inferred from electronic annotation with InterPro:IPR019170	-	Hsap\TMEM67	3R
L1	CG5466	FBgn0038815	CG5466	-	CG5466	histone H3-K79 methylation ; GO:0034729 | inferred from electronic annotation with InterPro:IPR030445 <newline> regulation of cell cycle ; GO:0051726 | inferred from electronic annotation with InterPro:IPR030445	-	histone methyltransferase activity (H3-K79 specific) ; GO:0031151 | inferred from electronic annotation with InterPro:IPR030445	Hsap\AES	3R

L1	H	FBgn0001169	CG5460	Hairless	H	somatic stem cell population maintenance ; GO:0035019 | inferred from mutant phenotype <newline> wing disc dorsal/ventral pattern formation ; GO:0048190 | inferred from mutant phenotype <newline> negative regulation of transcription by RNA polymerase II ; GO:0000122 | inferred from direct assay <newline> negative regulation of Notch signaling pathway ; GO:0045746 | inferred from mutant phenotype <newline> negative regulation of Notch signaling pathway ; GO:0045746 | inferred from genetic interaction with N <newline> intestinal stem cell homeostasis ; GO:0036335 | inferred from genetic interaction with mir-305 <newline> sensory organ boundary specification ; GO:0008052 | inferred from mutant phenotype <newline> regulation of imaginal disc-derived wing size ; GO:0044719 | inferred from mutant phenotype <newline> sensory organ precursor cell fate determination ; [illegible]	RNA polymerase II transcription repressor complex ; GO:0090571 | inferred from physical interaction with Su(H)	protein binding ; GO:0005515 | inferred from physical interaction with asf1 <newline> transcription corepressor activity ; GO:0003714 | inferred from direct assay <newline> transcription corepressor activity ; GO:0003714 | inferred from genetic interaction with Su(H)	-	3R

L1	Pi3K92E	FBgn0015279	CG4141	Pi3K92E	Pi3K92E	regulation of multicellular organism growth ; GO:0040014 | inferred from mutant phenotype <newline> phosphatidylinositol 3-kinase signaling ; GO:0014065 | inferred from mutant phenotype <newline> oogenesis ; GO:0048477 | inferred from mutant phenotype <newline> cellular response to starvation ; GO:0009267 | inferred from mutant phenotype <newline> positive regulation of cell size ; GO:0045793 | inferred from mutant phenotype <newline> metamorphosis ; GO:0007552 | inferred from mutant phenotype <newline> synapse maturation ; GO:0060074 | inferred from direct assay <newline> positive regulation of cell growth ; GO:0030307 | inferred from mutant phenotype <newline> larval salivary gland morphogenesis ; GO:0007436 | inferred from mutant phenotype <newline> insulin receptor signaling pathway ; GO:0008286 | inferred from mutant phenotype <newline> regulation of cell population [illegible]	phosphatidylinositol 3-kinase complex, class IA ; GO:0005943 | inferred from direct assay <newline> plasma membrane ; GO:0005886 | inferred from biological aspect of ancestor with PANTHER:PTN000005674 <newline> phosphatidylinositol 3-kinase complex, class IA ; GO:0005943 | inferred from physical interaction with Pi3K21B <newline> membrane ; GO:0016020 | inferred from biological aspect of ancestor with PANTHER:PTN000005673 <newline> phosphatidylinositol 3-kinase complex ; GO:0005942 | inferred from biological aspect of ancestor with PANTHER:PTN000005674 <newline> cytoplasm ; GO:0005737 | inferred from biological aspect of ancestor with PANTHER:PTN000005673	1-phosphatidylinositol-3-kinase activity ; GO:0016303 | inferred from biological aspect of ancestor with PANTHER:PTN000005674 <newline> 1-phosphatidylinositol-4-phosphate 3-kinase activity ; GO:0035005 | inferred from biological aspect of ancestor with PANTHER:PTN000005674 <newline> phosphatidylinositol-4,5-bisphosphate 3-kinase activity ; GO:0046934 | inferred from direct assay <newline> phosphatidylinositol 3-kinase activity ; GO:0035004 | inferred from direct assay <newline> 1-phosphatidylinositol-3-kinase activity ; GO:0016303 | inferred from direct assay <newline> 1-phosphatidylinositol-4-phosphate 3-kinase activity ; GO:0035005 | inferred from direct assay	Hsap\PIK3CG <newline> Hsap\PIK3CB <newline> Hsap\PIK3CA <newline> Hsap\PIK3CD	3R

L1	Lrrk	FBgn0038816	CG5483	Leucine-rich repeat kinase	Lrrk	axo-dendritic transport ; GO:0008088 | inferred from mutant phenotype <newline> cellular response to starvation ; GO:0009267 | inferred from mutant phenotype <newline> regulation of microtubule cytoskeleton organization ; GO:0070507 | inferred from mutant phenotype <newline> negative regulation of dendrite morphogenesis ; GO:0050774 | inferred from mutant phenotype <newline> intracellular distribution of mitochondria ; GO:0048312 | inferred from mutant phenotype <newline> regulation of autophagosome assembly ; GO:2000785 | inferred from mutant phenotype <newline> regulation of synapse maturation ; GO:0090128 | inferred from mutant phenotype <newline> regulation of terminal button organization ; GO:2000331 | inferred from mutant phenotype <newline> autophagy ; GO:0006914 | inferred from mutant phenotype <newline> synapse organization ; [illegible]	neuromuscular junction ; GO:0031594 | inferred from direct assay <newline> terminal bouton ; GO:0043195 | inferred from direct assay <newline> lysosomal membrane ; GO:0005765 | inferred from direct assay <newline> dendritic shaft ; GO:0043198 | inferred from direct assay <newline> Golgi stack ; GO:0005795 | inferred from direct assay <newline> subsynaptic reticulum ; GO:0071212 | inferred from direct assay <newline> type I terminal bouton ; GO:0061174 | inferred from direct assay <newline> cytoplasm ; GO:0005737 | inferred from direct assay <newline> dendritic branch point ; GO:1990033 | inferred from direct assay <newline> plasma membrane ; GO:0005886 | inferred from biological aspect of ancestor with PANTHER:PTN000701462 <newline> late endosome membrane ; GO:0031902 | [illegible]	ATP binding ; GO:0005524 | inferred from electronic annotation with InterPro:IPR000719, InterPro:IPR002290, InterPro:IPR017441 <newline> protein kinase activity ; GO:0004672 | inferred from direct assay <newline> Rab GTPase binding ; GO:0017137 | inferred from physical interaction with Rab5 inferred from physical interaction with Rab7 <newline> protein serine/threonine kinase activity ; GO:0004674 | inferred from direct assay <newline> protein serine/threonine kinase activity ; GO:0004674 | inferred from mutant phenotype <newline> protein binding ; GO:0005515 | inferred from physical interaction with UniProtKB:Q9W596 <newline> Rab GTPase binding ; GO:0017137 | inferred from physical interaction with Rab18	Hsap\RIPK1 <newline> Hsap\LRRK2 <newline> Hsap\PBK <newline> Hsap\RIPK2 <newline> Hsap\MAP3K7 <newline> Hsap\MLKL <newline> Hsap\MAP3K10 <newline> Hsap\MAP3K11 <newline> Hsap\RIPK3 <newline> Hsap\RIPK4 <newline> Hsap\MAP3K21 <newline> Hsap\MAP3K9 <newline> Hsap\MAP3K20 <newline> Hsap\DSTYK <newline> Hsap\MOS <newline> Hsap\LRRK1	3R

L1	GluRIIE	FBgn0051201	CG31201	Glutamate receptor IIE	GluRIIE	calcium ion transport ; GO:0006816 | inferred from direct assay <newline> neuromuscular synaptic transmission ; GO:0007274 | inferred from expression pattern <newline> synaptic transmission, glutamatergic ; GO:0035249 | inferred by curator from GO:0007274,GO:0008328 <newline> modulation of chemical synaptic transmission ; GO:0050804 | inferred from biological aspect of ancestor with PANTHER:PTN001826301 <newline> synaptic transmission, glutamatergic ; GO:0035249 | inferred from biological aspect of ancestor with PANTHER:PTN001826301 <newline> regulation of synaptic activity ; GO:0060025 | inferred from sequence or structural similarity with GluRIIA	plasma membrane ; GO:0005886 | inferred from biological aspect of ancestor with PANTHER:PTN000437926 <newline> ionotropic glutamate receptor complex ; GO:0008328 | inferred from mutant phenotype <newline> postsynaptic density membrane ; GO:0098839 | inferred from biological aspect of ancestor with PANTHER:PTN001826301 <newline> postsynaptic membrane ; GO:0045211 | inferred from biological aspect of ancestor with PANTHER:PTN001826301 <newline> muscle cell postsynaptic specialization ; GO:0097482 | inferred from direct assay	ionotropic glutamate receptor activity ; GO:0004970 | inferred by curator from GO:0008328 <newline> glutamate receptor activity ; GO:0008066 | inferred from biological aspect of ancestor with PANTHER:PTN001826301 <newline> glutamate-gated calcium ion channel activity ; GO:0022849 | inferred from direct assay <newline> transmitter-gated ion channel activity involved in regulation of postsynaptic membrane potential ; GO:1904315 | inferred from biological aspect of ancestor with PANTHER:PTN001826301	Hsap\GRIK1 <newline> Hsap\GRIN3B <newline> Hsap\GRIK4 <newline> Hsap\GRIK3 <newline> Hsap\GRID1 <newline> Hsap\GRIA2 <newline> Hsap\GRIA1 <newline> Hsap\GRID2 <newline> Hsap\GRIA3 <newline> Hsap\GRIN1 <newline> Hsap\GRIN3A <newline> Hsap\GRIK2 <newline> Hsap\GRIK5 <newline> Hsap\GRIA4	3R

L1	CG18039	FBgn0028422	CG18039	Glutamate receptor IID	GluRIID	synaptic transmission, glutamatergic ; GO:0035249 | inferred from biological aspect of ancestor with PANTHER:PTN001826301 <newline> calcium ion transport ; GO:0006816 | inferred from direct assay <newline> rhythmic excitation ; GO:0043179 | inferred from mutant phenotype <newline> synaptic transmission, glutamatergic ; GO:0035249 | inferred by curator from GO:0007274,GO:0008328 <newline> neuromuscular synaptic transmission ; GO:0007274 | inferred from expression pattern <newline> neuromuscular synaptic transmission ; GO:0007274 | inferred from mutant phenotype <newline> modulation of chemical synaptic transmission ; GO:0050804 | inferred from biological aspect of ancestor with PANTHER:PTN001826301	muscle cell postsynaptic specialization ; GO:0097482 | inferred from direct assay <newline> plasma membrane ; GO:0005886 | inferred from biological aspect of ancestor with PANTHER:PTN000437926 <newline> ionotropic glutamate receptor complex ; GO:0008328 | inferred from mutant phenotype <newline> postsynaptic membrane ; GO:0045211 | inferred from biological aspect of ancestor with PANTHER:PTN001826301 <newline> postsynaptic density membrane ; GO:0098839 | inferred from biological aspect of ancestor with PANTHER:PTN001826301	glutamate receptor activity ; GO:0008066 | inferred from biological aspect of ancestor with PANTHER:PTN001826301 <newline> ionotropic glutamate receptor activity ; GO:0004970 | inferred from mutant phenotype <newline> ionotropic glutamate receptor activity ; GO:0004970 | inferred by curator from GO:0008328 <newline> glutamate-gated calcium ion channel activity ; GO:0022849 | inferred from direct assay <newline> kainate selective glutamate receptor activity ; GO:0015277 | inferred from sequence or structural similarity <newline> transmitter-gated ion channel activity involved in regulation of postsynaptic membrane potential ; GO:1904315 | inferred from biological aspect of ancestor with PANTHER:PTN001826301	Hsap\GRIA1 <newline> Hsap\GRIK3 <newline> Hsap\GRIK1 <newline> Hsap\GRIA3 <newline> Hsap\GRID1 <newline> Hsap\GRIK2 <newline> Hsap\GRIA4 <newline> Hsap\GRIA2 <newline> Hsap\GRIK5 <newline> Hsap\GRIK4 <newline> Hsap\GRID2	3R
L1	Nep4	FBgn0038818	CG4058	Neprilysin 4	Nep4	proteolysis ; GO:0006508 | inferred from sequence model <newline> proteolysis ; GO:0006508 | inferred from direct assay	integral component of membrane ; GO:0016021 | inferred from sequence model <newline> extracellular space ; GO:0005615 | inferred from direct assay <newline> sarcoplasmic reticulum ; GO:0016529 | inferred from direct assay	metalloendopeptidase activity ; GO:0004222 | inferred from electronic annotation with InterPro:IPR000718, InterPro:IPR018497 <newline> metallopeptidase activity ; GO:0008237 | inferred from sequence model <newline> endopeptidase activity ; GO:0004175 | inferred from direct assay	Hsap\KEL <newline> Hsap\ECE1 <newline> Hsap\ECEL1 <newline> Hsap\MMEL1 <newline> Hsap\ECE2 <newline> Hsap\PHEX <newline> Hsap\MME	3R

L2	hep	FBgn0010303	CG4353	hemipterous	hep	ovarian follicle cell development ; GO:0030707 | inferred from mutant phenotype <newline> melanization defense response ; GO:0035006 | inferred from mutant phenotype <newline> imaginal disc fusion, thorax closure ; GO:0046529 | inferred from mutant phenotype inferred from genetic interaction with Rab30 <newline> response to heat ; GO:0009408 | inferred from mutant phenotype <newline> positive regulation of actin filament bundle assembly ; GO:0032233 | inferred from mutant phenotype <newline> positive regulation of JNK cascade ; GO:0046330 | inferred from mutant phenotype <newline> positive regulation of autophagy ; GO:0010508 | inferred from mutant phenotype <newline> imaginal disc-derived male genitalia morphogenesis ; GO:0048803 | inferred from mutant phenotype <newline> wound healing ; GO:0042060 | inferred from mutant phenotype <newline> positive [illegible]	axon ; GO:0030424 | inferred from direct assay	ATP binding ; GO:0005524 | inferred from electronic annotation with InterPro:IPR000719, InterPro:IPR002290, InterPro:IPR017441 <newline> JUN kinase kinase activity ; GO:0008545 | inferred from sequence or structural similarity <newline> JUN kinase kinase activity ; GO:0008545 | traceable author statement <newline> protein binding ; GO:0005515 | inferred from physical interaction with Cka <newline> protein kinase binding ; GO:0019901 | inferred from physical interaction with UniProtKB:Q9W0K0 <newline> MAP kinase kinase activity ; GO:0004708 | traceable author statement <newline> MAP-kinase scaffold activity ; GO:0005078 | inferred from direct assay <newline> protein kinase activity ; GO:0004672 | inferred from direct assay	Hsap\MAP2K5 <newline> Hsap\MAP2K1 <newline> Hsap\MAP2K7 <newline> Hsap\MAP2K2 <newline> Hsap\MAP2K6 <newline> Hsap\MAP2K4 <newline> Hsap\MAP2K3	X

L2	lic	FBgn0261524	CG12244	licorne	lic	activation of MAPK activity ; GO:0000187 | inferred from direct assay <newline> p38MAPK cascade ; GO:0038066 | inferred from mutant phenotype <newline> positive regulation of cell size ; GO:0045793 | inferred from mutant phenotype inferred from genetic interaction with gig <newline> MAPK cascade ; GO:0000165 | inferred from direct assay <newline> mucosal immune response ; GO:0002385 | inferred from mutant phenotype <newline> signal transduction by protein phosphorylation ; GO:0023014 | inferred from biological aspect of ancestor with PANTHER:PTN000684825 <newline> negative regulation of canonical Wnt signaling pathway ; GO:0090090 | inferred from mutant phenotype <newline> oocyte axis specification ; GO:0007309 | inferred from mutant phenotype <newline> oocyte anterior/posterior axis specification ; GO:0007314 | non-traceable [illegible]	cytoplasm ; GO:0005737 | inferred from sequence or structural similarity with SGD:S000003664 <newline> cytoplasm ; GO:0005737 | inferred from biological aspect of ancestor with PANTHER:PTN000684825	ATP binding ; GO:0005524 | inferred from electronic annotation with InterPro:IPR000719 <newline> mitogen-activated protein kinase kinase kinase binding ; GO:0031435 | inferred from physical interaction with Mekk1 <newline> protein kinase activity ; GO:0004672 | inferred from direct assay <newline> MAP kinase kinase activity ; GO:0004708 | traceable author statement <newline> MAP kinase kinase activity ; GO:0004708 | inferred from direct assay <newline> MAP kinase kinase activity ; GO:0004708 | inferred from biological aspect of ancestor with PANTHER:PTN000684494 <newline> protein serine/threonine kinase activity ; GO:0004674 | inferred from biological aspect of ancestor with PANTHER:PTN000684825	Hsap\MAP2K4 <newline> Hsap\LOC100996792 <newline> Hsap\MAP2K3 <newline> Hsap\MAP2K1 <newline> Hsap\MAP2K5 <newline> Hsap\MAP2K7 <newline> Hsap\MAP2K2 <newline> Hsap\MAP2K6	X
L2	CG2200	FBgn0030447	CG2200	-	CG2200	proteolysis ; GO:0006508 | inferred from electronic annotation with InterPro:IPR005320	-	serine-type peptidase activity ; GO:0008236 | inferred from electronic annotation with InterPro:IPR005320 <newline> peptidase activity ; GO:0008233 | inferred from biological aspect of ancestor with PANTHER:PTN001073551	-	X
L2	CG43313	FBgn0263005	CG43313	-	CG43313	-	Golgi cisterna membrane ; GO:0032580 | inferred from electronic annotation with InterPro:IPR008428	acetylgalactosaminyltransferase activity ; GO:0008376 | inferred from electronic annotation with InterPro:IPR008428	Hsap\CHPF2 <newline> Hsap\CHPF	X

L2	Fer3HCH	FBgn0030449	CG4349	Ferritin 3 heavy chain homologue	Fer3HCH	iron ion import across plasma membrane ; GO:0098711 | inferred from biological aspect of ancestor with PANTHER:PTN000153324 <newline> detoxification of iron ion ; GO:1990461 | inferred from biological aspect of ancestor with PANTHER:PTN000153324 <newline> cellular response to iron ion starvation ; GO:0010106 | inferred from biological aspect of ancestor with PANTHER:PTN000893123 <newline> cellular response to iron ion starvation ; GO:0010106 | inferred from mutant phenotype <newline> intracellular sequestering of iron ion ; GO:0006880 | inferred from biological aspect of ancestor with PANTHER:PTN000153092	intracellular ferritin complex ; GO:0008043 | inferred from biological aspect of ancestor with PANTHER:PTN000153324 <newline> cytoplasm ; GO:0005737 | inferred from biological aspect of ancestor with PANTHER:PTN000153092 <newline> Golgi apparatus ; GO:0005794 | inferred from biological aspect of ancestor with PANTHER:PTN000153324	ferroxidase activity ; GO:0004322 | inferred from biological aspect of ancestor with PANTHER:PTN000153092 <newline> ferrous iron binding ; GO:0008198 | inferred from biological aspect of ancestor with PANTHER:PTN000153092 <newline> identical protein binding ; GO:0042802 | inferred from biological aspect of ancestor with PANTHER:PTN000153092 <newline> ferric iron binding ; GO:0008199 | inferred from biological aspect of ancestor with PANTHER:PTN000153092 <newline> iron ion binding ; GO:0005506 | inferred from biological aspect of ancestor with PANTHER:PTN000153092	Hsap\FTH1 <newline> Hsap\FTL <newline> Hsap\FTH1P18 <newline> Hsap\FTHL17 <newline> Hsap\FTMT	X
L2	CG42237	FBgn0250862	CG42237	-	CG42237	phospholipid metabolic process ; GO:0006644 | inferred from electronic annotation with InterPro:IPR016090 <newline> arachidonic acid secretion ; GO:0050482 | inferred from electronic annotation with InterPro:IPR016090	-	calcium-dependent phospholipase A2 activity ; GO:0047498 | inferred from biological aspect of ancestor with PANTHER:PTN000956767	Hsap\PROCA1 <newline> Hsap\PLA2G3	X
L2	CG32638	FBgn0052638	CG32638	-	CG32638	angiotensin-activated signaling pathway ; GO:0038166 | inferred from electronic annotation with InterPro:IPR009436	-	-	Hsap\AGTRAP	X
L2	CG15717	FBgn0030451	CG15717	-	CG15717	oxidation-reduction process ; GO:0055114 | inferred from electronic annotation with InterPro:IPR016161	-	oxidoreductase activity ; GO:0016491 | inferred from electronic annotation with InterPro:IPR016161	-	X
L2	MFS10	FBgn0030452	CG4330	Major Facilitator Superfamily Transporter 10	MFS10	transmembrane transport ; GO:0055085 | inferred from electronic annotation with InterPro:IPR011701	integral component of membrane ; GO:0016021 | inferred from biological aspect of ancestor with PANTHER:PTN000184222	-	Hsap\SLC17A8 <newline> Hsap\SLC17A2 <newline> Hsap\SLC17A3 <newline> Hsap\SLC17A4 <newline> Hsap\SLC17A9 <newline> Hsap\SLC17A6 <newline> Hsap\SLC17A7 <newline> Hsap\SLC17A5 <newline> Hsap\SLC17A1	X

L2	CG32645	FBgn0052645	CG32645	-	CG32645	-	-	transferase activity, transferring acyl groups other than amino-acyl groups ; GO:0016747 | inferred from electronic annotation with InterPro:IPR002656	-	X
L2	CG4318	FBgn0030455	CG4318	-	CG4318	regulation of transcription, DNA-templated ; GO:0006355 | inferred from sequence or structural similarity with UniProtKB:P41696	nucleus ; GO:0005634 | inferred from sequence or structural similarity with UniProtKB:P41696	zinc ion binding ; GO:0008270 | inferred from electronic annotation with InterPro:IPR012934 <newline> DNA-binding transcription factor activity ; GO:0003700 | inferred from sequence or structural similarity with UniProtKB:P41696 <newline> transcription regulatory region sequence-specific DNA binding ; GO:0000976 | inferred from sequence or structural similarity with UniProtKB:P41696	Hsap\ZNF581 <newline> Hsap\ZNF524 <newline> Hsap\ZNF358 <newline> Hsap\ZNF480 <newline> Hsap\ZNF362	X
L2	CG4332	FBgn0030456	CG4332	-	CG4332	-	integral component of membrane ; GO:0016021 | inferred from electronic annotation with InterPro:IPR008429 <newline> endomembrane system ; GO:0012505 | inferred from high throughput direct assay	-	Hsap\CLPTM1 <newline> Hsap\CLPTM1L	X

L2	Bap60	FBgn0025463	CG4303	Brahma associated protein 60kD	Bap60	positive regulation of transcription, DNA-templated ; GO:0045893 | inferred from direct assay <newline> negative regulation of neuroblast proliferation ; GO:0007406 | inferred from mutant phenotype <newline> positive regulation of heterochromatin assembly ; GO:0031453 | inferred from mutant phenotype <newline> positive regulation of gene expression ; GO:0010628 | inferred from genetic interaction with brm <newline> regulation of transcription by RNA polymerase II ; GO:0006357 | inferred from mutant phenotype	SWI/SNF complex ; GO:0016514 | inferred from electronic annotation with InterPro:IPR038041 <newline> npBAF complex ; GO:0071564 | inferred from electronic annotation with InterPro:IPR038041 <newline> nBAF complex ; GO:0071565 | inferred from electronic annotation with InterPro:IPR038041 <newline> nuclear euchromatin ; GO:0005719 | inferred from direct assay <newline> brahma complex ; GO:0035060 | inferred from direct assay <newline> nucleus ; GO:0005634 | inferred from direct assay <newline> RSC-type complex ; GO:0016586 | inferred from direct assay	transcription coactivator activity ; GO:0003713 | contributes_to inferred from mutant phenotype <newline> DNA binding ; GO:0003677 | inferred from direct assay <newline> DNA-binding transcription activator activity, RNA polymerase II-specific ; GO:0001228 | contributes_to inferred from mutant phenotype <newline> protein domain specific binding ; GO:0019904 | inferred from physical interaction with UniProtKB:Q9VQ56 <newline> myosin binding ; GO:0017022 | inferred from physical interaction with Myo10A <newline> RNA polymerase II transcription factor binding ; GO:0001085 | inferred from physical interaction with sisA inferred from physical interaction with sc <newline> protein binding ; GO:0005515 | inferred from physical interaction with mor <newline> transcription factor binding ; GO:0008134 | inferred from sequence or structural [illegible]	Hsap\ASIC2 <newline> Hsap\ASIC1 <newline> Hsap\SMARCD1 <newline> Hsap\SMARCD3 <newline> Hsap\SMARCD2	X
L2	CG12096	FBgn0030457	CG12096	-	CG12096	proteasome assembly ; GO:0043248 | inferred from electronic annotation with InterPro:IPR019538	proteasome regulatory particle, base subcomplex ; GO:0008540 | inferred from biological aspect of ancestor with PANTHER:PTN000339132	-	Hsap\PSMD5	X

L2	CG34411	FBgn0085440	CG34411	Leucine-rich repeat-containing G protein-coupled receptor 4	Lgr4	activation of adenylate cyclase activity ; GO:0007190 | inferred from biological aspect of ancestor with PANTHER:PTN001224574 <newline> hormone-mediated signaling pathway ; GO:0009755 | inferred from biological aspect of ancestor with PANTHER:PTN001224574 <newline> G protein-coupled receptor signaling pathway ; GO:0007186 | inferred from sequence model <newline> G protein-coupled receptor signaling pathway ; GO:0007186 | inferred from sequence or structural similarity <newline> adenylate cyclase-activating G protein-coupled receptor signaling pathway ; GO:0007189 | inferred from biological aspect of ancestor with PANTHER:PTN001224574	integral component of membrane ; GO:0016021 | inferred from sequence or structural similarity <newline> integral component of membrane ; GO:0016021 | inferred from sequence model <newline> integral component of plasma membrane ; GO:0005887 | inferred from biological aspect of ancestor with PANTHER:PTN001224574	neuropeptide receptor activity ; GO:0008188 | inferred from sequence model <newline> G protein-coupled peptide receptor activity ; GO:0008528 | inferred from biological aspect of ancestor with PANTHER:PTN001224574 <newline> protein-hormone receptor activity ; GO:0016500 | inferred from sequence or structural similarity	Hsap\FSHR <newline> Hsap\LHCGR <newline> Hsap\RXFP1 <newline> Hsap\RXFP2 <newline> Hsap\FLRT2 <newline> Hsap\LGR6 <newline> Hsap\TSHR <newline> Hsap\LGR4 <newline> Hsap\LGR5 <newline> Hsap\LRFN5	X
L2	CG32640	FBgn0052640	CG32640	-	CG32640	-	-	-	Hsap\DNAJB5 <newline> Hsap\DNAJB1 <newline> Hsap\DNAJB13 <newline> Hsap\DNAJC18 <newline> Hsap\DNAJB12 <newline> Hsap\DNAJB14 <newline> Hsap\DNAJB4 <newline> Hsap\DNAJA3	X
L2	CG32641	FBgn0052641	CG32641	-	CG32641	-	-	-	Hsap\DNAJB13 <newline> Hsap\DNAJC25-GNG10 <newline> Hsap\DNAJA3 <newline> Hsap\DNAJB5 <newline> Hsap\DNAJB1 <newline> Hsap\DNAJB4 <newline> Hsap\DNAJC24	X
L2	CG12723	FBgn0030459	CG12723	-	CG12723	biological_process ; GO:0008150 | no biological data available	cellular_component ; GO:0005575 | no biological data available	molecular_function ; GO:0003674 | no biological data available	Hsap\MARCO	X
L2	CG2453	FBgn0030460	CG2453	Coenzyme Q5	Coq5	ubiquinone biosynthetic process ; GO:0006744 | inferred from sequence or structural similarity with UniProtKB:P49017	mitochondrion ; GO:0005739 | inferred from biological aspect of ancestor with PANTHER:PTN000010481 <newline> mitochondrion ; GO:0005739 | inferred from sequence or structural similarity with UniProtKB:P49017	2-hexaprenyl-6-methoxy-1,4-benzoquinone methyltransferase activity ; GO:0043334 | inferred from sequence or structural similarity with UniProtKB:P49017	Hsap\COQ5	X

L2	sno	FBgn0265630	CG44436	strawberry notch	sno	imaginal disc-derived wing margin morphogenesis ; GO:0008587 | inferred from mutant phenotype <newline> photoreceptor cell development ; GO:0042461 | inferred from mutant phenotype <newline> Notch signaling pathway ; GO:0007219 | inferred from mutant phenotype <newline> positive regulation of transcription by RNA polymerase II ; GO:0045944 | inferred from mutant phenotype <newline> positive regulation of Notch signaling pathway ; GO:0045747 | inferred from genetic interaction with H inferred from genetic interaction with Su(H) <newline> wing disc dorsal/ventral pattern formation ; GO:0048190 | inferred from mutant phenotype	nucleus ; GO:0005634 | inferred from biological aspect of ancestor with PANTHER:PTN000295173 <newline> nucleus ; GO:0005634 | inferred from direct assay	histone binding ; GO:0042393 | inferred from biological aspect of ancestor with PANTHER:PTN000295173 <newline> chromatin DNA binding ; GO:0031490 | inferred from biological aspect of ancestor with PANTHER:PTN000295173	Hsap\SBNO1 <newline> Hsap\SBNO2	X
L2	REG	FBgn0029133	CG1591	Proteasome regulator gamma	REG	positive regulation of endopeptidase activity ; GO:0010950 | inferred from biological aspect of ancestor with PANTHER:PTN000069920 <newline> regulation of proteasomal protein catabolic process ; GO:0061136 | inferred from direct assay <newline> regulation of proteasomal protein catabolic process ; GO:0061136 | inferred from biological aspect of ancestor with PANTHER:PTN000069920 <newline> regulation of G1/S transition of mitotic cell cycle ; GO:2000045 | inferred from mutant phenotype <newline> mRNA splicing, via spliceosome ; GO:0000398 | inferred by curator from GO:0071011,GO:0071013 <newline> regulation of G1/S transition of mitotic cell cycle ; GO:2000045 | inferred from biological aspect of ancestor with PANTHER:PTN000069920	proteasome activator complex ; GO:0008537 | inferred from electronic annotation with InterPro:IPR003185, InterPro:IPR003186, InterPro:IPR009077 <newline> nucleus ; GO:0005634 | inferred from direct assay <newline> polytene chromosome puff ; GO:0005703 | inferred from direct assay <newline> cytoplasm ; GO:0005737 | colocalizes_with inferred from direct assay <newline> precatalytic spliceosome ; GO:0071011 | inferred from high throughput direct assay <newline> catalytic step 2 spliceosome ; GO:0071013 | inferred from high throughput direct assay <newline> nucleus ; GO:0005634 | colocalizes_with inferred from direct assay <newline> nucleoplasm ; GO:0005654 | inferred from biological aspect of ancestor with PANTHER:PTN000069920 <newline> polytene chromosome [illegible]	endopeptidase activator activity ; GO:0061133 | inferred from direct assay <newline> endopeptidase inhibitor activity ; GO:0004866 | inferred from direct assay <newline> endopeptidase activator activity ; GO:0061133 | inferred from biological aspect of ancestor with PANTHER:PTN000069920	Hsap\PSME1 <newline> Hsap\PSME2 <newline> Hsap\PSME3	X

L2	mew	FBgn0004456	CG1771	multiple edematous wings	mew	salivary gland boundary specification ; GO:0007432 | inferred from mutant phenotype <newline> cell-cell adhesion ; GO:0098609 | traceable author statement <newline> substrate adhesion-dependent cell spreading ; GO:0034446 | inferred from direct assay <newline> maintenance of epithelial integrity, open tracheal system ; GO:0035160 | inferred from genetic interaction with if <newline> cell adhesion ; GO:0007155 | inferred from mutant phenotype <newline> muscle attachment ; GO:0016203 | inferred from genetic interaction with Grip inferred from genetic interaction with kon <newline> sensory perception of smell ; GO:0007608 | inferred from genetic interaction with sws <newline> ectodermal digestive tract morphogenesis ; GO:0048567 | traceable author statement <newline> cell adhesion mediated by integrin ; GO:0033627 | inferred from mutant phenotype	integral component of plasma membrane ; GO:0005887 | inferred from direct assay <newline> integrin complex ; GO:0008305 | inferred from direct assay <newline> integrin complex ; GO:0008305 | non-traceable author statement <newline> lateral plasma membrane ; GO:0016328 | inferred from direct assay <newline> basal plasma membrane ; GO:0009925 | inferred from direct assay <newline> integrin complex ; GO:0008305 | inferred from sequence or structural similarity with HGNC:6139 <newline> focal adhesion ; GO:0005925 | inferred from direct assay <newline> apical plasma membrane ; GO:0016324 | inferred from direct assay	cell adhesion molecule binding ; GO:0050839 | inferred from mutant phenotype <newline> extracellular matrix binding ; GO:0050840 | inferred from direct assay <newline> protein heterodimerization activity ; GO:0046982 | inferred from physical interaction with mys	Hsap\ITGA7 <newline> Hsap\ITGA6 <newline> Hsap\ITGA3	X
L2	CG32639	FBgn0052639	CG32639	-	CG32639	biological_process ; GO:0008150 | no biological data available	cellular_component ; GO:0005575 | no biological data available	molecular_function ; GO:0003674 | no biological data available	-	X
L2	CG15742	FBgn0030462	CG15742	-	CG15742	-	-	-	-	X

L2	comt	FBgn0000346	CG1618	comatose	comt	SNARE complex disassembly ; GO:0035494 | inferred from direct assay <newline> autophagy ; GO:0006914 | inferred from mutant phenotype <newline> Golgi organization ; GO:0007030 | inferred from mutant phenotype <newline> neurotransmitter secretion ; GO:0007269 | non-traceable author statement <newline> regulation of neuromuscular synaptic transmission ; GO:1900073 | inferred from genetic interaction with Dysb <newline> synaptic vesicle priming ; GO:0016082 | non-traceable author statement <newline> regulation of short-term neuronal synaptic plasticity ; GO:0048172 | inferred from direct assay <newline> synaptic vesicle fusion to presynaptic active zone membrane ; GO:0031629 | inferred from mutant phenotype <newline> neuron cellular homeostasis ; GO:0070050 | inferred from mutant phenotype <newline> Golgi vesicle [illegible]	synaptic vesicle ; GO:0008021 | colocalizes_with inferred from direct assay <newline> cytoplasm ; GO:0005737 | inferred from direct assay <newline> cytoplasm ; GO:0005737 | non-traceable author statement <newline> Golgi stack ; GO:0005795 | inferred from biological aspect of ancestor with PANTHER:PTN000554891 <newline> terminal bouton ; GO:0043195 | inferred from direct assay <newline> neuromuscular junction of somatic muscle ; GO:0098527 | inferred from direct assay	ATP binding ; GO:0005524 | inferred from electronic annotation with InterPro:IPR003959, InterPro:IPR003960 <newline> ATPase activity ; GO:0016887 | inferred from biological aspect of ancestor with PANTHER:PTN000554891 <newline> ATPase activity ; GO:0016887 | traceable author statement	Hsap\NSF	X

L2	HDAC4	FBgn0041210	CG1770	Histone deacetylase 4	HDAC4	insulin receptor signaling pathway ; GO:0008286 | inferred from mutant phenotype <newline> histone deacetylation ; GO:0016575 | inferred from mutant phenotype <newline> regulation of transcription, DNA-templated ; GO:0006355 | inferred from direct assay <newline> positive regulation of DNA-binding transcription factor activity ; GO:0051091 | inferred from mutant phenotype <newline> histone acetylation ; GO:0016573 | inferred from direct assay <newline> memory ; GO:0007613 | inferred from mutant phenotype <newline> histone deacetylation ; GO:0016575 | inferred from direct assay <newline> cellular response to starvation ; GO:0009267 | inferred from expression pattern <newline> long-term memory ; GO:0007616 | inferred from mutant phenotype	histone deacetylase complex ; GO:0000118 | inferred from electronic annotation with InterPro:IPR017320 <newline> nucleus ; GO:0005634 | inferred from direct assay <newline> cytosol ; GO:0005829 | inferred from direct assay <newline> cytoplasm ; GO:0005737 | inferred from direct assay	histone deacetylase activity ; GO:0004407 | inferred from direct assay <newline> histone deacetylase activity ; GO:0004407 | inferred from mutant phenotype <newline> protein deacetylase activity ; GO:0033558 | inferred from direct assay <newline> SUMO transferase activity ; GO:0019789 | inferred from sequence or structural similarity with UniProtKB:P56524	Hsap\HDAC6 <newline> Hsap\HDAC10 <newline> Hsap\HDAC5 <newline> Hsap\HDAC4 <newline> Hsap\HDAC9 <newline> Hsap\HDAC7	X

L2	CG15743	FBgn0030465	CG15743	-	CG15743	phosphatidylinositol phosphorylation ; GO:0046854 | inferred from electronic annotation with InterPro:IPR000760, InterPro:IPR020550 <newline> signal transduction ; GO:0007165 | inferred from sequence or structural similarity with UniProtKB:P29218 <newline> phosphate-containing compound metabolic process ; GO:0006796 | inferred from sequence or structural similarity with UniProtKB:P29218 <newline> dephosphorylation ; GO:0016311 | inferred from sequence or structural similarity with RGD:621833 <newline> phosphatidylinositol biosynthetic process ; GO:0006661 | inferred from sequence or structural similarity with UniProtKB:P29218	endomembrane system ; GO:0012505 | inferred from high throughput direct assay <newline> cytoplasm ; GO:0005737 | inferred from sequence or structural similarity with UniProtKB:P29218	inositol-1,4-bisphosphate 1-phosphatase activity ; GO:0004441 | inferred from sequence or structural similarity with RGD:621833 <newline> inositol monophosphate 1-phosphatase activity ; GO:0008934 | inferred from sequence or structural similarity with UniProtKB:P29218 <newline> 3'-nucleotidase activity ; GO:0008254 | inferred from biological aspect of ancestor with PANTHER:PTN001073847	Hsap\BPNT1 <newline> Hsap\IMPAD1 <newline> Hsap\INPP1 <newline> Hsap\UBE2W	X
L2	CG15744	FBgn0030466	CG15744	-	CG15744	G protein-coupled receptor signaling pathway ; GO:0007186 | inferred from electronic annotation with InterPro:IPR000832 <newline> cell surface receptor signaling pathway ; GO:0007166 | inferred from electronic annotation with InterPro:IPR017981	integral component of membrane ; GO:0016021 | inferred from electronic annotation with InterPro:IPR000832	G protein-coupled receptor activity ; GO:0004930 | inferred from electronic annotation with InterPro:IPR000832, InterPro:IPR001879	Hsap\LGI2 <newline> Hsap\LGI4 <newline> Hsap\LGI1 <newline> Hsap\ADGRA1 <newline> Hsap\ADGRA2 <newline> Hsap\ADGRA3 <newline> Hsap\ADGRG4 <newline> Hsap\LGI3	X
L2	CG1764	FBgn0030467	CG1764	-	CG1764	positive regulation of nitric oxide biosynthetic process ; GO:0045429 | inferred from biological aspect of ancestor with PANTHER:PTN000978164 <newline> citrulline metabolic process ; GO:0000052 | inferred from biological aspect of ancestor with PANTHER:PTN000978164 <newline> arginine metabolic process ; GO:0006525 | inferred from biological aspect of ancestor with PANTHER:PTN000296881	-	dimethylargininase activity ; GO:0016403 | inferred from biological aspect of ancestor with PANTHER:PTN000978164 <newline> amino acid binding ; GO:0016597 | inferred from biological aspect of ancestor with PANTHER:PTN000296881	Hsap\DDAH1 <newline> Hsap\DDAH2	X

L2	CG1622	FBgn0030468	CG1622	-	CG1622	mRNA splicing, via spliceosome ; GO:0000398 | inferred by curator from GO:0071011	precatalytic spliceosome ; GO:0071011 | inferred from high throughput direct assay <newline> precatalytic spliceosome ; GO:0071011 | inferred from biological aspect of ancestor with PANTHER:PTN000567228	-	Hsap\PRPF38B	X
L2	CG15745	FBgn0283680	CG45017	Inositol 1,4,5-triphosphate kinase 2	IP3K2	salivary gland cell autophagic cell death ; GO:0035071 | inferred from mutant phenotype inferred from genetic interaction with mir-14 <newline> inositol phosphate biosynthetic process ; GO:0032958 | inferred from direct assay	cytoplasm ; GO:0005737 | inferred from direct assay	calcium-dependent protein binding ; GO:0048306 | inferred from direct assay <newline> calmodulin binding ; GO:0005516 | inferred from direct assay <newline> inositol-1,4,5-trisphosphate 3-kinase activity ; GO:0008440 | inferred from direct assay	Hsap\ITPKB <newline> Hsap\ITPKC <newline> Hsap\ITPKA	X
L2	IP3K2	FBgn0283680	CG45017	Inositol 1,4,5-triphosphate kinase 2	IP3K2	salivary gland cell autophagic cell death ; GO:0035071 | inferred from mutant phenotype inferred from genetic interaction with mir-14 <newline> inositol phosphate biosynthetic process ; GO:0032958 | inferred from direct assay	cytoplasm ; GO:0005737 | inferred from direct assay	calcium-dependent protein binding ; GO:0048306 | inferred from direct assay <newline> calmodulin binding ; GO:0005516 | inferred from direct assay <newline> inositol-1,4,5-trisphosphate 3-kinase activity ; GO:0008440 | inferred from direct assay	Hsap\ITPKB <newline> Hsap\ITPKC <newline> Hsap\ITPKA	X

L2	Jafrac1	FBgn0040309	CG1633	thioredoxin peroxidase 1	Jafrac1	oxidation-reduction process ; GO:0055114 | inferred from electronic annotation with InterPro:IPR000866, InterPro:IPR019479 <newline> response to oxidative stress ; GO:0006979 | inferred from biological aspect of ancestor with PANTHER:PTN000073874 <newline> germ cell migration ; GO:0008354 | inferred from mutant phenotype <newline> cell redox homeostasis ; GO:0045454 | inferred from direct assay <newline> response to starvation ; GO:0042594 | inferred from mutant phenotype <newline> cell redox homeostasis ; GO:0045454 | inferred from biological aspect of ancestor with PANTHER:PTN000073874 <newline> hydrogen peroxide catabolic process ; GO:0042744 | inferred from direct assay <newline> determination of adult lifespan ; GO:0008340 | inferred from mutant phenotype <newline> hydrogen peroxide catabolic process ; [illegible]	cytosol ; GO:0005829 | inferred from biological aspect of ancestor with PANTHER:PTN000073874 <newline> cytosol ; GO:0005829 | inferred from direct assay	thioredoxin peroxidase activity ; GO:0008379 | inferred from direct assay <newline> thioredoxin peroxidase activity ; GO:0008379 | non-traceable author statement <newline> thioredoxin peroxidase activity ; GO:0008379 | inferred from biological aspect of ancestor with PANTHER:PTN000073874	Hsap\PRDX6 <newline> Hsap\PRDX2 <newline> Hsap\PRDX4 <newline> Hsap\PRDX1 <newline> Hsap\PRDX3	X
L2	RpS15Aa	FBgn0010198	CG2033	Ribosomal protein S15Aa	RpS15Aa	translation ; GO:0006412 | inferred from electronic annotation with InterPro:IPR000630 <newline> cytoplasmic translation ; GO:0002181 | inferred by curator from GO:0022626 <newline> cytoplasmic translation ; GO:0002181 | traceable author statement	cytosolic ribosome ; GO:0022626 | inferred from high throughput direct assay <newline> cytosolic small ribosomal subunit ; GO:0022627 | traceable author statement <newline> cytosolic small ribosomal subunit ; GO:0022627 | inferred from biological aspect of ancestor with PANTHER:PTN000204723	structural constituent of ribosome ; GO:0003735 | inferred from high throughput direct assay <newline> structural constituent of ribosome ; GO:0003735 | traceable author statement <newline> structural constituent of ribosome ; GO:0003735 | inferred from biological aspect of ancestor with PANTHER:PTN000204723	Hsap\RPS15A	X
L2	CG15747	FBgn0030474	CG15747	-	CG15747	mRNA splicing, via spliceosome ; GO:0000398 | inferred by curator from GO:0071011,GO:0071013	precatalytic spliceosome ; GO:0071011 | inferred from high throughput direct assay <newline> catalytic step 2 spliceosome ; GO:0071013 | inferred from high throughput direct assay	-	Hsap\NSRP1	X

L2	Syt12	FBgn0261085	CG10617	Synaptotagmin 12	Syt12	cellular response to calcium ion ; GO:0071277 | inferred from biological aspect of ancestor with PANTHER:PTN000001283 <newline> regulation of dopamine secretion ; GO:0014059 | inferred from biological aspect of ancestor with PANTHER:PTN000001283 <newline> regulation of calcium ion-dependent exocytosis ; GO:0017158 | inferred from biological aspect of ancestor with PANTHER:PTN000001283 <newline> vesicle-mediated transport ; GO:0016192 | inferred from biological aspect of ancestor with PANTHER:PTN000001284 <newline> calcium-ion regulated exocytosis ; GO:0017156 | inferred from biological aspect of ancestor with PANTHER:PTN000001283 <newline> long-term synaptic potentiation ; GO:0060291 | inferred from electronic annotation with InterPro:IPR030537 <newline> regulation of neurotransmitter secretion ; GO:0046928 | inferred from electronic annotation with	integral component of membrane ; GO:0016021 | inferred from sequence model <newline> plasma membrane ; GO:0005886 | inferred from biological aspect of ancestor with PANTHER:PTN000001284 <newline> exocytic vesicle ; GO:0070382 | inferred from biological aspect of ancestor with PANTHER:PTN000001284	clathrin binding ; GO:0030276 | inferred from biological aspect of ancestor with PANTHER:PTN000001283 <newline> calcium ion binding ; GO:0005509 | inferred from biological aspect of ancestor with PANTHER:PTN000001283 <newline> syntaxin binding ; GO:0019905 | inferred from biological aspect of ancestor with PANTHER:PTN000797811 <newline> calcium-dependent phospholipid binding ; GO:0005544 | inferred from biological aspect of ancestor with PANTHER:PTN000001283 <newline> SNARE binding ; GO:0000149 | inferred from biological aspect of ancestor with PANTHER:PTN000001283 <newline> phosphatidylserine binding ; GO:0001786 | inferred from biological aspect of ancestor with PANTHER:PTN000001283	Hsap\SYT10 <newline> Hsap\SYT12 <newline> Hsap\SYT6 <newline> Hsap\SYT3 <newline> Hsap\SYT9	X
L2	dmrt11E	FBgn0030477	CG15749	doublesex-Mab related 11E	dmrt11E	regulation of transcription by RNA polymerase II ; GO:0006357 | inferred from sequence or structural similarity with UniProtKB:P23023 <newline> spermatogenesis ; GO:0007283 | inferred from mutant phenotype	nucleus ; GO:0005634 | inferred from electronic annotation with InterPro:IPR026607	DNA-binding transcription activator activity, RNA polymerase II-specific ; GO:0001228 | inferred from sequence or structural similarity with UniProtKB:P23023 <newline> RNA polymerase II regulatory region sequence-specific DNA binding ; GO:0000977 | inferred from sequence or structural similarity with UniProtKB:P23023	Hsap\DMRT3 <newline> Hsap\DMRTA2 <newline> Hsap\DMRTC1 <newline> Hsap\DMRT1 <newline> Hsap\DMRTB1 <newline> Hsap\DMRTC2 <newline> Hsap\DMRTA1 <newline> Hsap\DMRT2	X

L2	CG1640	FBgn0030478	CG1640	-	CG1640	biosynthetic process ; GO:0009058 | inferred from electronic annotation with InterPro:IPR004839	cytosol ; GO:0005829 | inferred from high throughput direct assay <newline> mitochondrion ; GO:0005739 | inferred from high throughput direct assay	pyridoxal phosphate binding ; GO:0030170 | inferred from electronic annotation with InterPro:IPR004839 <newline> L-alanine:2-oxoglutarate aminotransferase activity ; GO:0004021 | inferred from sequence or structural similarity with UniProtKB:Q8TD30 <newline> L-alanine:2-oxoglutarate aminotransferase activity ; GO:0004021 | inferred from biological aspect of ancestor with PANTHER:PTN002272157	Hsap\GPT2 <newline> Hsap\GPT	X
L2	Rbp1-like	FBgn0030479	CG1987	Rbp1-like	Rbp1-like	regulation of mRNA 3'-end processing ; GO:0031440 | inferred from mutant phenotype <newline> mRNA splicing, via spliceosome ; GO:0000398 | inferred by curator from GO:0071011 <newline> regulation of gene expression ; GO:0010468 | inferred from mutant phenotype <newline> regulation of alternative mRNA splicing, via spliceosome ; GO:0000381 | inferred from mutant phenotype <newline> mRNA cis splicing, via spliceosome ; GO:0045292 | inferred from biological aspect of ancestor with PANTHER:PTN000567445 <newline> RNA splicing ; GO:0008380 | inferred from mutant phenotype <newline> regulation of transcriptional start site selection at RNA polymerase II promoter ; GO:0001178 | inferred from mutant phenotype <newline> regulation of alternative mRNA splicing, via spliceosome ; GO:0000381 | inferred from biological aspect of	precatalytic spliceosome ; GO:0071011 | inferred from high throughput direct assay <newline> nucleus ; GO:0005634 | inferred from biological aspect of ancestor with PANTHER:PTN000567445 <newline> nuclear speck ; GO:0016607 | inferred from biological aspect of ancestor with PANTHER:PTN000567445	RNA binding ; GO:0003723 | inferred from biological aspect of ancestor with PANTHER:PTN000567445 <newline> mRNA binding ; GO:0003729 | inferred from direct assay	Hsap\RBM4 <newline> Hsap\RBM14-RBM4 <newline> Hsap\RBM15B <newline> Hsap\SRSF5 <newline> Hsap\SPEN <newline> Hsap\SRSF9 <newline> Hsap\SRSF3 <newline> Hsap\BBS1 <newline> Hsap\PSPC1 <newline> Hsap\RBM4B <newline> Hsap\SRSF7 <newline> Hsap\RBM15 <newline> Hsap\SFPQ <newline> Hsap\SRSF6 <newline> Hsap\SRSF4 <newline> Hsap\NONO	X
L2	Tim9a	FBgn0030480	CG1660	Tim9a	Tim9a	protein targeting to mitochondrion ; GO:0006626 | inferred from sequence or structural similarity with UniProtKB:Q9Y5J7	TIM23 mitochondrial import inner membrane translocase complex ; GO:0005744 | inferred from sequence or structural similarity with UniProtKB:Q9Y5J7	P-P-bond-hydrolysis-driven protein transmembrane transporter activity ; GO:0015450 | inferred from sequence or structural similarity with UniProtKB:Q9Y5J7	Hsap\TIMM9 <newline> Hsap\TIMM10B	X

L2	Yippee	FBgn0026749	CG1989	Yippee	Yippee	biological_process ; GO:0008150 | no biological data available	ubiquitin ligase complex ; GO:0000151 | inferred from biological aspect of ancestor with PANTHER:PTN002302768	metal ion binding ; GO:0046872 | non-traceable author statement	Hsap\YPEL5 <newline> Hsap\YPEL1 <newline> Hsap\YPEL3 <newline> Hsap\YPEL2 <newline> Hsap\YPEL4	X
L2	CG1662	FBgn0030481	CG1662	-	CG1662	mitochondrial ribosome assembly ; GO:0061668 | inferred from biological aspect of ancestor with PANTHER:PTN000137585	integral component of membrane ; GO:0016021 | inferred from electronic annotation with InterPro:IPR007248 <newline> cytoplasm ; GO:0005737 | inferred from biological aspect of ancestor with PANTHER:PTN000745045	-	Hsap\MPV17L <newline> Hsap\PXMP2 <newline> Hsap\MPV17 <newline> Hsap\MPV17L2	X
L2	CG1673	FBgn0030482	CG1673	-	CG1673	leucine biosynthetic process ; GO:0009098 | inferred from biological aspect of ancestor with PANTHER:PTN000214538 <newline> valine biosynthetic process ; GO:0009099 | inferred from biological aspect of ancestor with PANTHER:PTN000214538 <newline> branched-chain amino acid biosynthetic process ; GO:0009082 | inferred from sequence or structural similarity with UniProtKB:O15382	mitochondrion ; GO:0005739 | inferred from sequence or structural similarity with UniProtKB:O15382 <newline> mitochondrion ; GO:0005739 | inferred from biological aspect of ancestor with PANTHER:PTN000214537 <newline> cytoplasm ; GO:0005737 | inferred from sequence or structural similarity with UniProtKB:O15382	branched-chain-amino-acid transaminase activity ; GO:0004084 | inferred from sequence or structural similarity with UniProtKB:O15382 <newline> branched-chain-amino-acid transaminase activity ; GO:0004084 | inferred from biological aspect of ancestor with PANTHER:PTN000214538	Hsap\BCAT2 <newline> Hsap\BCAT1	X
L2	CG12725	FBgn0030483	CG12725	-	CG12725	biological_process ; GO:0008150 | no biological data available	cellular_component ; GO:0005575 | no biological data available	molecular_function ; GO:0003674 | no biological data available	-	X
L2	CG1681	FBgn0030484	CG1681	Glutathione S transferase T4	GstT4	glutathione metabolic process ; GO:0006749 | inferred from biological aspect of ancestor with PANTHER:PTN001679972	cytoplasm ; GO:0005737 | inferred from sequence or structural similarity with RGD:70952	glutathione transferase activity ; GO:0004364 | inferred from biological aspect of ancestor with PANTHER:PTN001679972 <newline> glutathione peroxidase activity ; GO:0004602 | inferred from sequence or structural similarity with UniProtKB:P30711	Hsap\GSTT2B <newline> Hsap\GSTT2 <newline> Hsap\MARS <newline> Hsap\VARS <newline> Hsap\LOC107985559 <newline> Hsap\LOC100652871 <newline> Hsap\GSTT1 <newline> Hsap\EEF1G <newline> Hsap\EEF1E1	X
L2	CG1998	FBgn0030485	CG1998	-	CG1998	oxidation-reduction process ; GO:0055114 | inferred from electronic annotation with InterPro:IPR006694 <newline> sterol biosynthetic process ; GO:0016126 | inferred from biological aspect of ancestor with PANTHER:PTN000221246	endoplasmic reticulum membrane ; GO:0005789 | inferred from biological aspect of ancestor with PANTHER:PTN000221169	iron ion binding ; GO:0005506 | inferred from electronic annotation with InterPro:IPR006694 <newline> C-4 methylsterol oxidase activity ; GO:0000254 | inferred from biological aspect of ancestor with PANTHER:PTN000221246	Hsap\MSMO1 <newline> Hsap\CH25H <newline> Hsap\FAXDC2	X

L2	Set2	FBgn0030486	CG1716	SET domain containing 2	Set2	histone methylation ; GO:0016571 | inferred from sequence or structural similarity with UniProtKB:Q9BZ95 <newline> instar larval development ; GO:0002168 | inferred from mutant phenotype <newline> regulation of transcription, DNA-templated ; GO:0006355 | inferred from biological aspect of ancestor with PANTHER:PTN002739645 <newline> wing disc development ; GO:0035220 | inferred from mutant phenotype <newline> ecdysone receptor-mediated signaling pathway ; GO:0035076 | inferred from genetic interaction with EcR <newline> histone H3-K36 methylation ; GO:0010452 | inferred from mutant phenotype	chromatin ; GO:0000785 | inferred from biological aspect of ancestor with PANTHER:PTN002739645 <newline> nucleus ; GO:0005634 | inferred from direct assay <newline> nucleus ; GO:0005634 | inferred from biological aspect of ancestor with PANTHER:PTN002739645	DNA binding ; GO:0003677 | inferred from electronic annotation with InterPro:IPR017956 <newline> histone-lysine N-methyltransferase activity ; GO:0018024 | inferred from sequence or structural similarity with UniProtKB:Q9BZ95 <newline> histone methyltransferase activity (H3-K36 specific) ; GO:0046975 | inferred from biological aspect of ancestor with PANTHER:PTN002739645 <newline> histone methyltransferase activity (H3-K36 specific) ; GO:0046975 | inferred from mutant phenotype <newline> phosphoprotein binding ; GO:0051219 | inferred from physical interaction with RpII215	Hsap\KMT2C <newline> Hsap\ASH1L <newline> Hsap\KMT2D <newline> Hsap\NSD3 <newline> Hsap\KMT2E <newline> Hsap\SETD1B <newline> Hsap\NSD2 <newline> Hsap\PRR34 <newline> Hsap\PHF20L1 <newline> Hsap\SETD5 <newline> Hsap\PHF20 <newline> Hsap\SETD2 <newline> Hsap\SETD1A <newline> Hsap\NSD1 <newline> Hsap\DIDO1	X
L2	Neto	FBgn0265416	CG44328	Neuropilin and tolloid-like	Neto	glutamate receptor clustering ; GO:0097688 | inferred from mutant phenotype <newline> postsynaptic density organization ; GO:0097106 | inferred from mutant phenotype <newline> hatching behavior ; GO:0035187 | inferred from mutant phenotype <newline> neuromuscular junction development ; GO:0007528 | inferred from mutant phenotype <newline> neuromuscular synaptic transmission ; GO:0007274 | inferred from mutant phenotype <newline> locomotion ; GO:0040011 | inferred from mutant phenotype <newline> receptor clustering ; GO:0043113 | inferred from mutant phenotype <newline> postsynaptic membrane organization ; GO:0001941 | inferred from mutant phenotype <newline> flight ; GO:0060361 | inferred from mutant phenotype	ionotropic glutamate receptor complex ; GO:0008328 | inferred from direct assay <newline> neuromuscular junction ; GO:0031594 | inferred from direct assay <newline> muscle cell postsynaptic specialization ; GO:0097482 | inferred from direct assay	molecular_function ; GO:0003674 | no biological data available	Hsap\NETO1 <newline> Hsap\MFRP <newline> Hsap\NETO2 <newline> Hsap\CDCP2	X

L2	CG34324	FBgn0085353	CG34324	-	CG34324	chitin metabolic process ; GO:0006030 | inferred from electronic annotation with InterPro:IPR002557	extracellular region ; GO:0005576 | inferred from electronic annotation with InterPro:IPR002557	chitin binding ; GO:0008061 | inferred from electronic annotation with InterPro:IPR002557	-	X
L3	disco	FBgn0000459	CG9908	disconnected	disco	photoreceptor cell maintenance ; GO:0045494 | non-traceable author statement <newline> positive regulation of transcription, DNA-templated ; GO:0045893 | inferred from mutant phenotype <newline> brain development ; GO:0007420 | inferred from mutant phenotype <newline> leg disc proximal/distal pattern formation ; GO:0007479 | inferred from mutant phenotype <newline> circadian rhythm ; GO:0007623 | non-traceable author statement <newline> locomotor rhythm ; GO:0045475 | inferred from mutant phenotype <newline> eclosion rhythm ; GO:0008062 | inferred from mutant phenotype <newline> regulation of gene expression ; GO:0010468 | inferred from mutant phenotype <newline> antennal development ; GO:0007469 | inferred from mutant phenotype <newline> positive regulation of transcription by RNA polymerase II ; GO:0045944 | inferred from direct assay	nucleus ; GO:0005634 | inferred from direct assay	RNA polymerase II regulatory region sequence-specific DNA binding ; GO:0000977 | inferred from direct assay <newline> DNA binding ; GO:0003677 | inferred from direct assay	Hsap\BNC2 <newline> Hsap\BNC1	X
L3	CG12507	FBgn0030729	CG12507	-	CG12507	biological_process ; GO:0008150 | no biological data available	cellular_component ; GO:0005575 | no biological data available	molecular_function ; GO:0003674 | no biological data available	-	X
L3	kat80	FBgn0040207	CG13956	katanin 80	kat80	dorsal appendage formation ; GO:0046843 | inferred from mutant phenotype <newline> microtubule severing ; GO:0051013 | inferred from electronic annotation with InterPro:IPR026962	katanin complex ; GO:0008352 | inferred from biological aspect of ancestor with PANTHER:PTN000457152 <newline> cytosol ; GO:0005829 | inferred from high throughput direct assay	microtubule binding ; GO:0008017 | inferred from electronic annotation with InterPro:IPR026962 <newline> microtubule-severing ATPase activity ; GO:0008568 | contributes_to inferred from biological aspect of ancestor with PANTHER:PTN000457152	Hsap\KATNBL1 <newline> Hsap\KATNB1	X

L3	Mfe2	FBgn0030731	CG3415	peroxisomal Multifunctional enzyme type 2	Mfe2	fatty acid beta-oxidation using acyl-CoA oxidase ; GO:0033540 | inferred by curator from GO:0004300,GO:0080023	peroxisome ; GO:0005777 | inferred from sequence or structural similarity with HGNC:5213 inferred from sequence model <newline> peroxisome ; GO:0005777 | inferred by curator from GO:0004300,GO:0080023	protein homodimerization activity ; GO:0042803 | inferred from direct assay <newline> 3-hydroxyacyl-CoA dehydrogenase activity ; GO:0003857 | inferred from sequence or structural similarity with UniProtKB:P51659 <newline> 3R-hydroxyacyl-CoA dehydratase activity ; GO:0080023 | inferred from direct assay <newline> enoyl-CoA hydratase activity ; GO:0004300 | inferred from direct assay <newline> 17-beta-hydroxysteroid dehydrogenase (NAD+) activity ; GO:0044594 | inferred from sequence or structural similarity with UniProtKB:P51659	Hsap\SCP2D1 <newline> Hsap\HSD17B4 <newline> Hsap\RPP14	X
L3	CG3422	FBgn0004066	CG3422	Proteasome alpha4 subunit	Prosalpha4	proteasome-mediated ubiquitin-dependent protein catabolic process ; GO:0043161 | inferred by curator from GO:0005839 <newline> proteasomal ubiquitin-independent protein catabolic process ; GO:0010499 | inferred from biological aspect of ancestor with PANTHER:PTN000174701 <newline> proteasomal protein catabolic process ; GO:0010498 | inferred from biological aspect of ancestor with PANTHER:PTN000174701 <newline> proteasome-mediated ubiquitin-dependent protein catabolic process ; GO:0043161 | inferred from biological aspect of ancestor with PANTHER:PTN000174701	proteasome core complex, alpha-subunit complex ; GO:0019773 | inferred from biological aspect of ancestor with PANTHER:PTN000175000 <newline> proteasome complex ; GO:0000502 | inferred from direct assay <newline> nucleus ; GO:0005634 | inferred from biological aspect of ancestor with PANTHER:PTN000174701 <newline> proteasome core complex ; GO:0005839 | inferred from biological aspect of ancestor with PANTHER:PTN000174701 <newline> proteasome core complex ; GO:0005839 | inferred from direct assay <newline> cytoplasm ; GO:0005737 | inferred from biological aspect of ancestor with PANTHER:PTN000174701 <newline> proteasome core complex, alpha-subunit complex ; GO:0019773 | inferred from sequence or structural similarity with UniProtKB:P40302	threonine-type endopeptidase activity ; GO:0004298 | inferred from electronic annotation with InterPro:IPR001353, InterPro:IPR023332 <newline> endopeptidase activity ; GO:0004175 | inferred from biological aspect of ancestor with PANTHER:PTN000174701 <newline> endopeptidase activity ; GO:0004175 | contributes_to inferred by curator from GO:0005839	Hsap\PSMA7 <newline> Hsap\PSMA2 <newline> Hsap\PSMA8 <newline> Hsap\PSMA5	X

L3	eas	FBgn0000536	CG3525	easily shocked	eas	associative learning ; GO:0008306 | traceable author statement <newline> mechanosensory behavior ; GO:0007638 | inferred from mutant phenotype <newline> habituation ; GO:0046959 | traceable author statement <newline> mushroom body development ; GO:0016319 | traceable author statement <newline> asymmetric neuroblast division ; GO:0055059 | inferred from mutant phenotype <newline> long-term memory ; GO:0007616 | traceable author statement <newline> mushroom body development ; GO:0016319 | inferred from mutant phenotype <newline> response to mechanical stimulus ; GO:0009612 | inferred from genetic interaction with kcc <newline> response to mechanical stimulus ; GO:0009612 | inferred from mutant phenotype <newline> phosphatidylethanolamine metabolic process ; GO:0046337 | inferred from direct assay [illegible]	cytoplasm ; GO:0005737 | inferred from sequence or structural similarity with SGD:S000004123	ethanolamine kinase activity ; GO:0004305 | inferred from direct assay <newline> choline kinase activity ; GO:0004103 | traceable author statement	Hsap\CHKA <newline> Hsap\CHKB <newline> Hsap\ETNK1 <newline> Hsap\ETNK2	X
L3	CG32576	FBgn0052576	CG32576	-	CG32576	vesicle-mediated transport ; GO:0016192 | inferred from electronic annotation with InterPro:IPR007305	endoplasmic reticulum ; GO:0005783 | inferred from biological aspect of ancestor with PANTHER:PTN002328730	molecular_function ; GO:0003674 | no biological data available	Hsap\GOLT1B <newline> Hsap\GOLT1A	X

L3	CG3560	FBgn0030733	CG3560	Ubiquinol-cytochrome c reductase 14 kDa subunit	UQCR-14	mitochondrial electron transport, ubiquinol to cytochrome c ; GO:0006122 | inferred from biological aspect of ancestor with PANTHER:PTN000246311 <newline> aerobic respiration ; GO:0009060 | inferred from biological aspect of ancestor with PANTHER:PTN000246311 <newline> mitochondrial respiratory chain complex III assembly ; GO:0034551 | inferred from biological aspect of ancestor with PANTHER:PTN000246311 <newline> mitochondrial electron transport, ubiquinol to cytochrome c ; GO:0006122 | inferred from sequence or structural similarity with UniProtKB:P14927 <newline> cellular respiration ; GO:0045333 | inferred from mutant phenotype	mitochondrial respiratory chain complex III ; GO:0005750 | inferred from sequence or structural similarity with UniProtKB:P14927 <newline> mitochondrial respiratory chain complex III ; GO:0005750 | inferred from biological aspect of ancestor with PANTHER:PTN000246311	ubiquinol-cytochrome-c reductase activity ; GO:0008121 | inferred from sequence or structural similarity with UniProtKB:P14927 <newline> ubiquinol-cytochrome-c reductase activity ; GO:0008121 | contributes_to inferred from biological aspect of ancestor with PANTHER:PTN000246311	Hsap\UQCRB	X
L3	caz	FBgn0285954	CG3606	cabeza	caz	regulation of transcription, DNA-templated ; GO:0006355 | inferred from electronic annotation with InterPro:IPR034870 <newline> adult locomotory behavior ; GO:0008344 | inferred from mutant phenotype <newline> transcription initiation from RNA polymerase II promoter ; GO:0006367 | inferred from sequence or structural similarity with HGNC:11545 <newline> mRNA splicing, via spliceosome ; GO:0000398 | inferred by curator from GO:0071013 <newline> positive regulation of synaptic growth at neuromuscular junction ; GO:0045887 | inferred from mutant phenotype <newline> larval locomotory behavior ; GO:0008345 | inferred from mutant phenotype <newline> synaptic growth at neuromuscular junction ; GO:0051124 | inferred from mutant phenotype <newline> compound eye development ; GO:0048749 | inferred from mutant phenotype	nucleus ; GO:0005634 | inferred from biological aspect of ancestor with PANTHER:PTN000579998 <newline> transcription factor TFIID complex ; GO:0005669 | inferred from sequence or structural similarity with HGNC:11545 <newline> nucleus ; GO:0005634 | inferred from direct assay <newline> transcriptionally active chromatin ; GO:0035327 | colocalizes_with inferred from direct assay <newline> nucleoplasm ; GO:0005654 | inferred from direct assay <newline> catalytic step 2 spliceosome ; GO:0071013 | inferred from high throughput direct assay	RNA binding ; GO:0003723 | inferred from electronic annotation with InterPro:IPR034870 <newline> chromatin binding ; GO:0003682 | inferred from direct assay <newline> mRNA binding ; GO:0003729 | inferred from sequence or structural similarity	Hsap\EWSR1 <newline> Hsap\RBMY1D <newline> Hsap\RBMY1F <newline> Hsap\RBMX <newline> Hsap\RBMY1J <newline> Hsap\RBMY1B <newline> Hsap\RBMXL1 <newline> Hsap\CIRBP <newline> Hsap\TAF15 <newline> Hsap\RBMY1A1 <newline> Hsap\RBMY1E <newline> Hsap\RBMXL3 <newline> Hsap\FUS <newline> Hsap\RBM3 <newline> Hsap\RBMXL2	X

L3	CG9911	FBgn0030734	CG9911	-	CG9911	cell redox homeostasis ; GO:0045454 | inferred from electronic annotation with InterPro:IPR013766	endomembrane system ; GO:0012505 | inferred from high throughput direct assay	-	Hsap\ERP44	X
L3	CG3632	FBgn0030735	CG3632	-	CG3632	peptidyl-tyrosine dephosphorylation ; GO:0035335 | inferred from sequence or structural similarity with HGNC:7451	cytoplasm ; GO:0005737 | inferred from sequence or structural similarity with HGNC:7451	metal ion binding ; GO:0046872 | inferred from electronic annotation with InterPro:IPR000306 <newline> protein serine/threonine phosphatase activity ; GO:0004722 | inferred from sequence or structural similarity with HGNC:7451 <newline> protein tyrosine phosphatase activity ; GO:0004725 | inferred from sequence or structural similarity with HGNC:7451	Hsap\MTMR4 <newline> Hsap\MTMR3 <newline> Hsap\MTMR2 <newline> Hsap\MTMR7 <newline> Hsap\SBF2 <newline> Hsap\MTMR8 <newline> Hsap\MTMR1 <newline> Hsap\MTMR9 <newline> Hsap\MTM1 <newline> Hsap\MTMR6	X
L3	CG3679	FBgn0027521	CG3679	-	CG3679	-	-	-	-	X
L3	CG9914	FBgn0286508	CG9914	beta Hydroxy acid dehydrogenase 1	Had1	oxidation-reduction process ; GO:0055114 | inferred from electronic annotation with InterPro:IPR006108, InterPro:IPR006176, InterPro:IPR006180, InterPro:IPR008927, InterPro:IPR013328, InterPro:IPR022694 <newline> fatty acid metabolic process ; GO:0006631 | inferred from electronic annotation with InterPro:IPR006108, InterPro:IPR006176, InterPro:IPR006180, InterPro:IPR022694	-	3-hydroxyacyl-CoA dehydrogenase activity ; GO:0003857 | inferred from electronic annotation with InterPro:IPR006108, InterPro:IPR006176, InterPro:IPR006180, InterPro:IPR022694 <newline> NAD+ binding ; GO:0070403 | inferred from electronic annotation with InterPro:IPR022694 <newline> L-gulonate 3-dehydrogenase activity ; GO:0050104 | inferred from direct assay <newline> L-gulonate 3-dehydrogenase activity ; GO:0050104 | inferred from mutant phenotype	Hsap\CRYL1	X

L3	CG9915	FBgn0030738	CG9915	-	CG9915	regulation of mRNA export from nucleus ; GO:0010793 | inferred from biological aspect of ancestor with PANTHER:PTN001114632 <newline> regulation of mRNA processing ; GO:0050684 | inferred from biological aspect of ancestor with PANTHER:PTN001114632 <newline> regulation of histone H3-K36 trimethylation ; GO:2001253 | inferred from biological aspect of ancestor with PANTHER:PTN001114632	nucleus ; GO:0005634 | inferred from biological aspect of ancestor with PANTHER:PTN001114632	-	Hsap\IWS1	X
L3	CG13957	FBgn0030739	CG13957	Strumpellin and WASH-interacting protein	SWIP	endosomal transport ; GO:0016197 | inferred from biological aspect of ancestor with PANTHER:PTN000776639 <newline> endosome organization ; GO:0007032 | inferred from biological aspect of ancestor with PANTHER:PTN000776639 <newline> positive regulation of cell adhesion ; GO:0045785 | inferred from mutant phenotype	WASH complex ; GO:0071203 | inferred from sequence or structural similarity with UniProtKB:Q2M389 <newline> WASH complex ; GO:0071203 | inferred from direct assay <newline> WASH complex ; GO:0071203 | inferred from biological aspect of ancestor with PANTHER:PTN000776639 <newline> endosome ; GO:0005768 | inferred from biological aspect of ancestor with PANTHER:PTN000776639	-	Hsap\WASHC4	X
L3	Cyp1	FBgn0004432	CG9916	Cyclophilin 1	Cyp1	protein peptidyl-prolyl isomerization ; GO:0000413 | inferred from electronic annotation with InterPro:IPR002130 <newline> protein refolding ; GO:0042026 | inferred from biological aspect of ancestor with PANTHER:PTN002594282	cytosol ; GO:0005829 | inferred from sequence or structural similarity <newline> cytoplasm ; GO:0005737 | inferred from high throughput direct assay <newline> nucleus ; GO:0005634 | inferred from high throughput direct assay	peptidyl-prolyl cis-trans isomerase activity ; GO:0003755 | inferred from biological aspect of ancestor with PANTHER:PTN002594282 <newline> peptidyl-prolyl cis-trans isomerase activity ; GO:0003755 | inferred from sequence or structural similarity <newline> unfolded protein binding ; GO:0051082 | inferred from biological aspect of ancestor with PANTHER:PTN002594282 <newline> cyclosporin A binding ; GO:0016018 | inferred from biological aspect of ancestor with PANTHER:PTN002594282	Hsap\PPIAL4A <newline> Hsap\RGPD2 <newline> Hsap\PPIH <newline> Hsap\PPIF <newline> Hsap\PPIL6 <newline> Hsap\PPIAL4E <newline> Hsap\PPIAL4G <newline> Hsap\LOC105371242 <newline> Hsap\NKTR <newline> Hsap\PPIAL4D <newline> Hsap\RGPD6 <newline> Hsap\PPID <newline> Hsap\PPIC <newline> Hsap\PPIE <newline> Hsap\RGPD8 <newline> Hsap\RGPD4 <newline> Hsap\RANBP2 <newline> Hsap\PPIG <newline> Hsap\PPIAL4F <newline> Hsap\PPIA <newline> Hsap\PPIAL4C <newline> Hsap\PPIB	X
L3	CG9917	FBgn0030740	CG9917	-	CG9917	-	-	-	-	X

L3	CG32579	FBgn0052579	CG32579	-	CG32579	engulfment of apoptotic cell ; GO:0043652 | inferred from biological aspect of ancestor with PANTHER:PTN000411029 <newline> phosphatidylserine exposure on apoptotic cell surface ; GO:0070782 | inferred from biological aspect of ancestor with PANTHER:PTN000411029 <newline> apoptotic process involved in development ; GO:1902742 | inferred from biological aspect of ancestor with PANTHER:PTN000411029	integral component of membrane ; GO:0016021 | inferred from electronic annotation with InterPro:IPR018629 <newline> plasma membrane ; GO:0005886 | inferred from biological aspect of ancestor with PANTHER:PTN000411029 <newline> membrane ; GO:0016020 | inferred from biological aspect of ancestor with PANTHER:PTN001281162	-	Hsap\XKR9 <newline> Hsap\XKR4 <newline> Hsap\XKR5 <newline> Hsap\XKR7 <newline> Hsap\XKR8 <newline> Hsap\XKR6	X
L3	CalpC	FBgn0260450	CG3692	Calpain C	CalpC	proteolysis ; GO:0006508 | NOT non-traceable author statement inferred from key residues	cytoplasm ; GO:0005737 | inferred from biological aspect of ancestor with PANTHER:PTN000021568 <newline> cytoplasm ; GO:0005737 | inferred from direct assay <newline> cytoplasm ; GO:0005737 | non-traceable author statement	calcium ion binding ; GO:0005509 | inferred from electronic annotation with InterPro:IPR002048 <newline> calcium-dependent cysteine-type endopeptidase activity ; GO:0004198 | inferred from biological aspect of ancestor with PANTHER:PTN000021568 <newline> calcium-dependent cysteine-type endopeptidase activity ; GO:0004198 | NOT non-traceable author statement inferred from key residues	Hsap\CAPN10 <newline> Hsap\CAPN8 <newline> Hsap\CAPN15 <newline> Hsap\CAPN2 <newline> Hsap\CAPN6 <newline> Hsap\CAPNS2 <newline> Hsap\CAPN12 <newline> Hsap\CAPN13 <newline> Hsap\CAPN1 <newline> Hsap\CAPN5 <newline> Hsap\CAPN11 <newline> Hsap\CAPNS1 <newline> Hsap\CAPN9 <newline> Hsap\CAPN3 <newline> Hsap\CAPN7 <newline> Hsap\CAPN14 <newline> Hsap\ADGB	X
L3	CG9919	FBgn0030742	CG9919	-	CG9919	-	-	-	-	X
L3	CG9921	FBgn0030743	CG9921	-	CG9921	-	-	-	Hsap\HIGD1A <newline> Hsap\HIGD1B <newline> Hsap\HIGD2A <newline> Hsap\HIGD1C	X

L3	Dsp1	FBgn0278608	CG12223	Dorsal switch protein 1	Dsp1	DNA recombination ; GO:0006310 | inferred from biological aspect of ancestor with PANTHER:PTN001009623 <newline> positive regulation of DNA binding ; GO:0043388 | inferred from direct assay <newline> chromatin remodeling ; GO:0006338 | inferred from mutant phenotype <newline> negative regulation of antimicrobial humoral response ; GO:0008348 | inferred from mutant phenotype inferred from genetic interaction with Rel <newline> developmental process ; GO:0032502 | inferred from mutant phenotype <newline> negative regulation of transcription, DNA-templated ; GO:0045892 | inferred from direct assay <newline> positive regulation of transcription by RNA polymerase II ; GO:0045944 | inferred from biological aspect of ancestor with PANTHER:PTN001009623 <newline> negative regulation of transcription by RNA polymerase II ; GO:0000122 | inferred from mutant phenotype [illegible]	cytoplasm ; GO:0005737 | inferred from biological aspect of ancestor with PANTHER:PTN001009623 <newline> nucleus ; GO:0005634 | inferred from direct assay <newline> nucleus ; GO:0005634 | non-traceable author statement <newline> nuclear chromatin ; GO:0000790 | inferred from biological aspect of ancestor with PANTHER:PTN000346026 <newline> nucleus ; GO:0005634 | inferred from high throughput direct assay <newline> polytene chromosome ; GO:0005700 | inferred from direct assay	DNA binding, bending ; GO:0008301 | inferred from biological aspect of ancestor with PANTHER:PTN000345179 <newline> protein binding ; GO:0005515 | inferred from physical interaction with UniProtKB:P23497-3 <newline> transcription factor binding ; GO:0008134 | inferred from biological aspect of ancestor with PANTHER:PTN000345179 <newline> transcription factor binding ; GO:0008134 | inferred from physical interaction with dl <newline> TBP-class protein binding ; GO:0017025 | inferred from physical interaction with Tbp <newline> DNA binding, bending ; GO:0008301 | inferred from direct assay <newline> DNA binding ; GO:0003677 | inferred from direct assay <newline> single-stranded DNA binding ; GO:0003697 | non-traceable author statement	Hsap\TOX4 <newline> Hsap\HMG20A <newline> Hsap\TOX <newline> Hsap\HMGB2 <newline> Hsap\TFAM <newline> Hsap\SSRP1 <newline> Hsap\TOX3 <newline> Hsap\HMGB3 <newline> Hsap\TOX2 <newline> Hsap\UBTF <newline> Hsap\HMG20B <newline> Hsap\HMGB4 <newline> Hsap\HMGB1	X

L3	sl	FBgn0003416	CG4200	small wing	sl	phospholipid catabolic process ; GO:0009395 | inferred from electronic annotation with InterPro:IPR016279 <newline> negative regulation of ERK1 and ERK2 cascade ; GO:0070373 | inferred from genetic interaction with rl <newline> response to insulin ; GO:0032868 | inferred from genetic interaction with InR <newline> negative regulation of cell differentiation ; GO:0045596 | inferred from mutant phenotype <newline> inositol trisphosphate biosynthetic process ; GO:0032959 | inferred from biological aspect of ancestor with PANTHER:PTN000036828 <newline> positive regulation of epithelial cell migration ; GO:0010634 | inferred from biological aspect of ancestor with PANTHER:PTN000741536 <newline> positive regulation of cell growth ; GO:0030307 | inferred from mutant phenotype <newline> negative regulation of R7 cell differentiation ; GO:0045677 | [illegible]	-	phosphatidylinositol phospholipase C activity ; GO:0004435 | inferred from biological aspect of ancestor with PANTHER:PTN000036885	Hsap\PLCH2 <newline> Hsap\PLCL2 <newline> Hsap\PLCD3 <newline> Hsap\PLCD4 <newline> Hsap\PLCG1 <newline> Hsap\PLCG2 <newline> Hsap\PLCD1	X

L3	U2af50	FBgn0005411	CG9998	U2 small nuclear riboprotein auxiliary factor 50	U2af50	regulation of alternative mRNA splicing, via spliceosome ; GO:0000381 | inferred from high throughput mutant phenotype <newline> RNA splicing ; GO:0008380 | inferred from mutant phenotype <newline> mRNA splicing, via spliceosome ; GO:0000398 | inferred by curator from GO:0071011 <newline> positive regulation of RNA export from nucleus ; GO:0046833 | inferred from mutant phenotype <newline> nuclear export ; GO:0051168 | inferred from mutant phenotype <newline> mRNA splicing, via spliceosome ; GO:0000398 | inferred from sequence or structural similarity with SGD:S000001557 <newline> mRNA splicing, via spliceosome ; GO:0000398 | inferred from mutant phenotype	precatalytic spliceosome ; GO:0071011 | inferred from high throughput direct assay <newline> U2AF ; GO:0089701 | inferred from direct assay <newline> U2AF ; GO:0089701 | inferred from biological aspect of ancestor with PANTHER:PTN000566979 <newline> U2-type prespliceosome ; GO:0071004 | inferred from biological aspect of ancestor with PANTHER:PTN000566979 <newline> spliceosomal complex ; GO:0005681 | inferred from sequence or structural similarity with SGD:S000001557 <newline> commitment complex ; GO:0000243 | inferred from biological aspect of ancestor with PANTHER:PTN000566979 <newline> nucleoplasm ; GO:0005654 | inferred from high throughput direct assay <newline> nucleus ; GO:0005634 | inferred by curator from GO:0000381 <newline> nuclear speck ; [illegible]	mRNA binding ; GO:0003729 | inferred from sequence or structural similarity <newline> poly-pyrimidine tract binding ; GO:0008187 | inferred from biological aspect of ancestor with PANTHER:PTN000566979 <newline> poly-pyrimidine tract binding ; GO:0008187 | inferred from direct assay <newline> RNA binding ; GO:0003723 | inferred from mutant phenotype <newline> protein binding ; GO:0005515 | inferred from physical interaction with U2af38 <newline> pre-mRNA 3'-splice site binding ; GO:0030628 | inferred from biological aspect of ancestor with PANTHER:PTN000566979	Hsap\U2AF2 <newline> Hsap\UHMK1	X
L3	CG32578	FBgn0086448	CG32578	Phosphatidylinositol glycan anchor biosynthesis class Q	PIG-Q	GPI anchor biosynthetic process ; GO:0006506 | inferred from sequence or structural similarity with HGNC:14135	integral component of membrane ; GO:0016021 | inferred from electronic annotation with InterPro:IPR007720 <newline> endoplasmic reticulum membrane ; GO:0005789 | inferred from sequence or structural similarity with HGNC:14135 <newline> glycosylphosphatidylinositol-N-acetylglucosaminyltransferase (GPI-GnT) complex ; GO:0000506 | inferred from biological aspect of ancestor with PANTHER:PTN000481757	phosphatidylinositol N-acetylglucosaminyltransferase activity ; GO:0017176 | contributes_to inferred from biological aspect of ancestor with PANTHER:PTN000481757	Hsap\PIGQ	X

L3	nonA	FBgn0004227	CG4211	no on or off transient A	nonA	mRNA splicing, via spliceosome ; GO:0000398 | inferred by curator from GO:0071011,GO:0071013 <newline> male courtship behavior, veined wing generated song production ; GO:0045433 | non-traceable author statement <newline> visual perception ; GO:0007601 | traceable author statement <newline> male courtship behavior, veined wing generated song production ; GO:0045433 | inferred from mutant phenotype <newline> visual perception ; GO:0007601 | inferred from mutant phenotype <newline> phototaxis ; GO:0042331 | non-traceable author statement <newline> male courtship behavior, veined wing generated song production ; GO:0045433 | traceable author statement <newline> visual behavior ; GO:0007632 | non-traceable author statement	nucleus ; GO:0005634 | inferred from direct assay <newline> nucleus ; GO:0005634 | inferred from biological aspect of ancestor with PANTHER:PTN000573351 <newline> precatalytic spliceosome ; GO:0071011 | inferred from high throughput direct assay <newline> nucleus ; GO:0005634 | inferred from high throughput direct assay <newline> catalytic step 2 spliceosome ; GO:0071013 | inferred from high throughput direct assay	mRNA binding ; GO:0003729 | traceable author statement <newline> transcription regulatory region sequence-specific DNA binding ; GO:0000976 | inferred from biological aspect of ancestor with PANTHER:PTN000573360 <newline> mRNA binding ; GO:0003729 | inferred from sequence or structural similarity	Hsap\BBS1 <newline> Hsap\SRSF5 <newline> Hsap\RBM14-RBM4 <newline> Hsap\SRSF4 <newline> Hsap\NONO <newline> Hsap\SRSF6 <newline> Hsap\RBM4 <newline> Hsap\SRSF7 <newline> Hsap\RBM15 <newline> Hsap\SRSF9 <newline> Hsap\SFPQ <newline> Hsap\RBM4B <newline> Hsap\PSPC1 <newline> Hsap\SRSF3 <newline> Hsap\SPEN <newline> Hsap\RBM15B	X
L3	Fur2	FBgn0004598	CG18734	Furin 2	Fur2	negative regulation of secretion ; GO:0051048 | inferred from genetic interaction with Pgant4 <newline> protein processing ; GO:0016485 | inferred from biological aspect of ancestor with PANTHER:PTN002451026 <newline> R8 cell fate specification ; GO:0045464 | inferred from mutant phenotype <newline> peptide hormone processing ; GO:0016486 | inferred from biological aspect of ancestor with PANTHER:PTN000082901	trans-Golgi network ; GO:0005802 | inferred from biological aspect of ancestor with PANTHER:PTN000082856 <newline> integral component of Golgi membrane ; GO:0030173 | inferred from biological aspect of ancestor with PANTHER:PTN000082856 <newline> membrane ; GO:0016020 | inferred from biological aspect of ancestor with PANTHER:PTN002451026 <newline> plasma membrane ; GO:0005886 | inferred from sequence or structural similarity with Fur1	serine-type endopeptidase activity ; GO:0004252 | inferred from biological aspect of ancestor with PANTHER:PTN002451026 <newline> serine-type endopeptidase activity ; GO:0004252 | inferred from genetic interaction with Fur1 <newline> serine-type endopeptidase activity ; GO:0004252 | inferred from direct assay	Hsap\PCSK4 <newline> Hsap\RSPO3 <newline> Hsap\RSPO4 <newline> Hsap\PCSK7 <newline> Hsap\FURIN <newline> Hsap\PCSK2 <newline> Hsap\RSPO1 <newline> Hsap\PCSK1 <newline> Hsap\FRAS1 <newline> Hsap\PCSK5 <newline> Hsap\PCSK6	X
L3	CG9992	FBgn0030744	CG9992	-	CG9992	biological_process ; GO:0008150 | no biological data available	cellular_component ; GO:0005575 | no biological data available	molecular_function ; GO:0003674 | no biological data available	-	X
L3	CG4239	FBgn0030745	CG4239	-	CG4239	monovalent inorganic cation transport ; GO:0015672 | inferred from electronic annotation with InterPro:IPR007866	membrane ; GO:0016020 | inferred from electronic annotation with InterPro:IPR007866	cation channel activity ; GO:0005261 | inferred from electronic annotation with InterPro:IPR007866	Hsap\TMEM38A <newline> Hsap\TMEM38B	X

L3	TH1	FBgn0010416	CG9984	TH1	TH1	negative regulation of transcription elongation from RNA polymerase II promoter ; GO:0034244 | inferred from direct assay <newline> negative regulation of transcription elongation from RNA polymerase II promoter ; GO:0034244 | inferred from biological aspect of ancestor with PANTHER:PTN000254315 <newline> negative regulation of transcription by RNA polymerase II ; GO:0000122 | inferred from direct assay	polytene chromosome interband ; GO:0005705 | inferred from direct assay <newline> NELF complex ; GO:0032021 | inferred from biological aspect of ancestor with PANTHER:PTN000254315 <newline> nucleus ; GO:0005634 | inferred from direct assay <newline> transcription elongation factor complex ; GO:0008023 | inferred from physical interaction with UniProtKB:P92204 <newline> NELF complex ; GO:0032021 | inferred from physical interaction with Nelf-E inferred from physical interaction with NELF-B inferred from physical interaction with Nelf-A <newline> polytene chromosome puff ; GO:0005703 | inferred from direct assay <newline> transcriptional repressor complex ; GO:0017053 | inferred from direct assay	RNA binding ; GO:0003723 | inferred from biological aspect of ancestor with PANTHER:PTN000254315 <newline> RNA binding ; GO:0003723 | inferred from direct assay	Hsap\NELFCD	X

L3	mei-41	FBgn0004367	CG4252	meiotic 41	mei-41	response to radiation ; GO:0009314 | traceable author statement <newline> imaginal disc development ; GO:0007444 | traceable author statement <newline> peptidyl-serine phosphorylation ; GO:0018105 | inferred from sequence or structural similarity with HGNC:882 <newline> regulation of mitotic nuclear division ; GO:0007088 | inferred from mutant phenotype inferred from genetic interaction with dup <newline> cellularization ; GO:0007349 | traceable author statement <newline> double-strand break repair via synthesis-dependent strand annealing ; GO:0045003 | inferred from mutant phenotype <newline> reciprocal meiotic recombination ; GO:0007131 | inferred from mutant phenotype <newline> double-strand break repair ; GO:0006302 | inferred from mutant phenotype <newline> female meiosis chromosome segregation ; [illegible]	nucleus ; GO:0005634 | inferred from sequence or structural similarity with HGNC:882 <newline> nucleus ; GO:0005634 | inferred from biological aspect of ancestor with PANTHER:PTN000124197	protein serine/threonine kinase activity ; GO:0004674 | inferred from sequence or structural similarity with HGNC:882 <newline> protein serine/threonine kinase activity ; GO:0004674 | inferred from biological aspect of ancestor with PANTHER:PTN001673376	Hsap\ATR <newline> Hsap\PRKDC	X
L3	CG9981	FBgn0030746	CG9981	-	CG9981	phospholipid translocation ; GO:0045332 | inferred from sequence or structural similarity with SGD:S000000024 <newline> phospholipid translocation ; GO:0045332 | inferred from biological aspect of ancestor with PANTHER:PTN000642368	integral component of membrane ; GO:0016021 | inferred from electronic annotation with InterPro:IPR001757, InterPro:IPR006539 <newline> plasma membrane ; GO:0005886 | inferred from biological aspect of ancestor with PANTHER:PTN000642368 <newline> integral component of membrane ; GO:0016021 | inferred from sequence model <newline> endoplasmic reticulum ; GO:0005783 | inferred from biological aspect of ancestor with PANTHER:PTN001184474 <newline> trans-Golgi network ; GO:0005802 | inferred from biological aspect of ancestor with PANTHER:PTN001943259	ATP binding ; GO:0005524 | inferred from electronic annotation with InterPro:IPR006539 <newline> magnesium ion binding ; GO:0000287 | inferred from electronic annotation with InterPro:IPR006539 <newline> ATPase-coupled intramembrane lipid transporter activity ; GO:0140326 | inferred from sequence or structural similarity with SGD:S000000024	Hsap\ATP9A <newline> Hsap\ATP11B <newline> Hsap\ATP10B <newline> Hsap\ATP8A1 <newline> Hsap\ATP9B <newline> Hsap\ATP8A2 <newline> Hsap\ATP8B2 <newline> Hsap\ATP8B3 <newline> Hsap\ATP11C <newline> Hsap\ATP8B1 <newline> Hsap\ATP10D <newline> Hsap\ATP10A <newline> Hsap\ATP8B4 <newline> Hsap\ATP11A	X

L3	CG4301	FBgn0030747	CG4301	-	CG4301	phospholipid translocation ; GO:0045332 | inferred from sequence or structural similarity with SGD:S000000024 <newline> phospholipid translocation ; GO:0045332 | inferred from biological aspect of ancestor with PANTHER:PTN000642368	integral component of membrane ; GO:0016021 | inferred from electronic annotation with InterPro:IPR001757, InterPro:IPR006539 <newline> integral component of membrane ; GO:0016021 | inferred from sequence model <newline> trans-Golgi network ; GO:0005802 | inferred from biological aspect of ancestor with PANTHER:PTN001943259 <newline> endoplasmic reticulum ; GO:0005783 | inferred from biological aspect of ancestor with PANTHER:PTN001184474 <newline> plasma membrane ; GO:0005886 | inferred from biological aspect of ancestor with PANTHER:PTN000642368	ATP binding ; GO:0005524 | inferred from electronic annotation with InterPro:IPR006539 <newline> magnesium ion binding ; GO:0000287 | inferred from electronic annotation with InterPro:IPR006539 <newline> ATPase-coupled intramembrane lipid transporter activity ; GO:0140326 | inferred from sequence or structural similarity with SGD:S000000024	Hsap\ATP8B2 <newline> Hsap\ATP8B3 <newline> Hsap\ATP11C <newline> Hsap\ATP8B4 <newline> Hsap\ATP8A1 <newline> Hsap\ATP10B <newline> Hsap\ATP10D <newline> Hsap\ATP10A <newline> Hsap\ATP9B <newline> Hsap\ATP11B <newline> Hsap\ATP8A2 <newline> Hsap\ATP8B1 <newline> Hsap\ATP11A <newline> Hsap\ATP9A	X
L3	Traf-like	FBgn0030748	CG4394	TNF-receptor-associated factor-like	Traf-like	positive regulation of lipophagy ; GO:1904504 | inferred from mutant phenotype	cytoplasm ; GO:0005737 | inferred from sequence or structural similarity with Traf4	protein binding ; GO:0005515 | inferred from physical interaction with UniProtKB:Q9U6M0	Hsap\TRAF6 <newline> Hsap\TRAF4 <newline> Hsap\TRAF1 <newline> Hsap\ERC1 <newline> Hsap\TRAF3 <newline> Hsap\TRAF5 <newline> Hsap\TRAF2	X
L3	CG9968	FBgn0030749	CG9968	Annexin B11	AnxB11	-	cellular_component ; GO:0005575 | no biological data available	calcium ion binding ; GO:0005509 | inferred from electronic annotation with InterPro:IPR001464, InterPro:IPR018252, InterPro:IPR018502 <newline> calcium-dependent phospholipid binding ; GO:0005544 | inferred from sequence or structural similarity with AnxB10	Hsap\ANXA3 <newline> Hsap\ANXA6 <newline> Hsap\ANXA11 <newline> Hsap\ANXA7 <newline> Hsap\ANXA1 <newline> Hsap\ANXA13 <newline> Hsap\ANXA2 <newline> Hsap\ANXA8L1 <newline> Hsap\ANXA10 <newline> Hsap\ANXA4 <newline> Hsap\ANXA5 <newline> Hsap\ANXA8 <newline> Hsap\ANXA9	X

L3	hang	FBgn0026575	CG32575	hangover	hang	behavioral response to ethanol ; GO:0048149 | inferred from mutant phenotype <newline> response to heat ; GO:0009408 | inferred from mutant phenotype <newline> response to ethanol ; GO:0045471 | inferred from mutant phenotype <newline> cAMP-mediated signaling ; GO:0019933 | inferred from mutant phenotype <newline> response to oxidative stress ; GO:0006979 | inferred from mutant phenotype	nucleus ; GO:0005634 | inferred from direct assay	zinc ion binding ; GO:0008270 | inferred from electronic annotation with InterPro:IPR012934 <newline> nucleic acid binding ; GO:0003676 | non-traceable author statement <newline> RNA binding ; GO:0003723 | inferred from direct assay	Hsap\THBS3 <newline> Hsap\PRDM15	X
L3	CG9947	FBgn0030752	CG9947	-	CDC50	phospholipid transport ; GO:0015914 | inferred from electronic annotation with InterPro:IPR030351	Golgi apparatus ; GO:0005794 | inferred from biological aspect of ancestor with PANTHER:PTN000100867 <newline> endoplasmic reticulum ; GO:0005783 | inferred from biological aspect of ancestor with PANTHER:PTN000100867 <newline> plasma membrane ; GO:0005886 | inferred from biological aspect of ancestor with PANTHER:PTN000100867 <newline> integral component of membrane ; GO:0016021 | inferred from electronic annotation with InterPro:IPR030351	-	Hsap\TMEM30B <newline> Hsap\TMEM30A <newline> Hsap\LOC101928242	X
L3	rngo	FBgn0030753	CG4420	rings lost	rngo	proteolysis ; GO:0006508 | inferred from electronic annotation with InterPro:IPR001995, InterPro:IPR019103 <newline> female germline ring canal formation ; GO:0007301 | inferred from mutant phenotype	nucleus ; GO:0005634 | inferred from direct assay <newline> nucleus ; GO:0005634 | inferred from high throughput direct assay <newline> cytoplasm ; GO:0005737 | inferred from high throughput direct assay <newline> cytosol ; GO:0005829 | inferred from high throughput direct assay <newline> cytoplasm ; GO:0005737 | inferred from direct assay	aspartic-type endopeptidase activity ; GO:0004190 | inferred from electronic annotation with InterPro:IPR001995, InterPro:IPR019103 <newline> proteasome binding ; GO:0070628 | inferred from physical interaction with Rpn10 <newline> ubiquitin binding ; GO:0043130 | inferred from direct assay	Hsap\NRIP3 <newline> Hsap\NRIP2 <newline> Hsap\DDI1 <newline> Hsap\DDI2 <newline> Hsap\RSC1A1	X

L3	CG9946	FBgn0261609	CG9946	eukaryotic translation initiation factor 2 subunit alpha	eIF2alpha	translational initiation ; GO:0006413 | inferred from sequence or structural similarity <newline> translational initiation ; GO:0006413 | inferred from sequence or structural similarity with HGNC:3265 inferred from sequence or structural similarity with SGD:S000003767 <newline> translational initiation ; GO:0006413 | inferred from sequence or structural similarity with SGD:S000001734	eukaryotic translation initiation factor 2B complex ; GO:0005851 | inferred from biological aspect of ancestor with PANTHER:PTN000063908 <newline> eukaryotic translation initiation factor 2B complex ; GO:0005851 | inferred from sequence or structural similarity with SGD:S000001734 <newline> eukaryotic translation initiation factor 2 complex ; GO:0005850 | inferred from biological aspect of ancestor with PANTHER:PTN000063908 <newline> eukaryotic translation initiation factor 2 complex ; GO:0005850 | inferred from sequence or structural similarity with HGNC:3265 inferred from sequence or structural similarity with SGD:S000003767 <newline> eukaryotic translation initiation factor 2 complex ; GO:0005850 | inferred from sequence or structural similarity <newline> eukaryotic 48S preinitiation [illegible]	translation initiation factor activity ; GO:0003743 | inferred from sequence or structural similarity <newline> translation initiation factor activity ; GO:0003743 | inferred from sequence or structural similarity with SGD:S000001734 inferred from genetic interaction with SGD:S000001734 <newline> ribosome binding ; GO:0043022 | inferred from biological aspect of ancestor with PANTHER:PTN000063907 <newline> translation initiation factor activity ; GO:0003743 | inferred from sequence or structural similarity with HGNC:3265 inferred from sequence or structural similarity with SGD:S000003767 <newline> translation initiation factor activity ; GO:0003743 | inferred from biological aspect of ancestor with PANTHER:PTN000063907	Hsap\HASPIN <newline> Hsap\EIF2S1	X
L3	CG34015	FBgn0054015	CG34015	-	CG34015	biological_process ; GO:0008150 | no biological data available	cellular_component ; GO:0005575 | no biological data available	catalytic activity ; GO:0003824 | inferred from electronic annotation with InterPro:IPR011146 <newline> molecular_function ; GO:0003674 | no biological data available	Hsap\HINT3	X

L3	CG4429	FBgn0262734	CG4429	eukaryotic translation initiation factor 4H1	eIF4H1	translational initiation ; GO:0006413 | inferred from sequence or structural similarity	polysome ; GO:0005844 | inferred from biological aspect of ancestor with PANTHER:PTN000579859 <newline> cytosol ; GO:0005829 | inferred from sequence or structural similarity	nucleic acid binding ; GO:0003676 | inferred from electronic annotation with InterPro:IPR000504, InterPro:IPR035979 <newline> translation initiation factor activity ; GO:0003743 | inferred from sequence or structural similarity <newline> mRNA binding ; GO:0003729 | inferred from sequence or structural similarity	Hsap\HNRNPC <newline> Hsap\HNRNPA1L2 <newline> Hsap\HNRNPA2B1 <newline> Hsap\BOLL <newline> Hsap\HNRNPA1 <newline> Hsap\DAZ2 <newline> Hsap\EIF4H <newline> Hsap\DAZL <newline> Hsap\SLIRP <newline> Hsap\HNRNPA3 <newline> Hsap\RALY <newline> Hsap\RALYL <newline> Hsap\HNRNPCL1 <newline> Hsap\DAZ4 <newline> Hsap\RBM3 <newline> Hsap\TRNAU1AP <newline> Hsap\EIF4B <newline> Hsap\DAZ3 <newline> Hsap\CIRBP <newline> Hsap\DAZ1 <newline> Hsap\HNRNPAB <newline> Hsap\SRSF10 <newline> Hsap\HNRNPD <newline> Hsap\RBM34	X

L3	para	FBgn0285944	CG9907	paralytic	para	membrane depolarization during action potential ; GO:0086010 | inferred from biological aspect of ancestor with PANTHER:PTN000004140 <newline> sodium ion transmembrane transport ; GO:0035725 | inferred from direct assay <newline> mechanosensory behavior ; GO:0007638 | inferred from mutant phenotype <newline> response to mechanical stimulus ; GO:0009612 | inferred from genetic interaction with kcc <newline> male courtship behavior, veined wing generated song production ; GO:0045433 | inferred from mutant phenotype <newline> regulation of postsynaptic membrane potential ; GO:0060078 | inferred from mutant phenotype <newline> neuronal action potential ; GO:0019228 | inferred from biological aspect of ancestor with PANTHER:PTN000798901 <newline> sodium ion transmembrane transport ; GO:0035725 | inferred [illegible]	plasma membrane ; GO:0005886 | inferred from biological aspect of ancestor with PANTHER:PTN000004140 <newline> integral component of plasma membrane ; GO:0005887 | inferred from direct assay <newline> voltage-gated sodium channel complex ; GO:0001518 | inferred from biological aspect of ancestor with PANTHER:PTN000004137	calcium ion binding ; GO:0005509 | inferred from electronic annotation with InterPro:IPR002048 <newline> voltage-gated sodium channel activity ; GO:0005248 | inferred from direct assay <newline> voltage-gated sodium channel activity ; GO:0005248 | inferred from biological aspect of ancestor with PANTHER:PTN000004137 <newline> sodium channel activity ; GO:0005272 | inferred from mutant phenotype <newline> voltage-gated sodium channel activity ; GO:0005248 | inferred from mutant phenotype	Hsap\SCN9A <newline> Hsap\SCN7A <newline> Hsap\SCN2A <newline> Hsap\CACNA1A <newline> Hsap\SCN5A <newline> Hsap\CACNA1C <newline> Hsap\SCN3A <newline> Hsap\CACNA1F <newline> Hsap\SCN11A <newline> Hsap\NALCN <newline> Hsap\SCN10A <newline> Hsap\SCN4A <newline> Hsap\CACNA1D <newline> Hsap\CACNA1B <newline> Hsap\SCN1A <newline> Hsap\CACNA1S <newline> Hsap\SCN8A	X
L3	Cnx14D	FBgn0264077	CG9906	Calnexin 14D	Cnx14D	endoplasmic reticulum unfolded protein response ; GO:0030968 | inferred from biological aspect of ancestor with PANTHER:PTN000117401 <newline> protein folding ; GO:0006457 | inferred from sequence or structural similarity <newline> protein folding ; GO:0006457 | inferred from biological aspect of ancestor with PANTHER:PTN000117401	endoplasmic reticulum membrane ; GO:0005789 | inferred from sequence or structural similarity <newline> endoplasmic reticulum membrane ; GO:0005789 | inferred from biological aspect of ancestor with PANTHER:PTN000117401	unfolded protein binding ; GO:0051082 | inferred from electronic annotation with InterPro:IPR001580 <newline> calcium ion binding ; GO:0005509 | inferred from sequence or structural similarity <newline> calcium ion binding ; GO:0005509 | inferred from biological aspect of ancestor with PANTHER:PTN000117401 <newline> unfolded protein binding ; GO:0051082 | inferred from sequence or structural similarity	Hsap\CLGN <newline> Hsap\CALR3 <newline> Hsap\CANX <newline> Hsap\CALR	X
L3	CG9903	FBgn0030756	CG9903	-	CG9903	-	membrane ; GO:0016020 | inferred from electronic annotation with InterPro:IPR002657	-	Hsap\SLC10A4 <newline> Hsap\SLC10A5 <newline> Hsap\SLC10A2 <newline> Hsap\SLC10A3 <newline> Hsap\SLC10A1 <newline> Hsap\SLC10A6	X
L3	CG9902	FBgn0030757	CG9902	-	CG9902	-	-	-	-	X

L3	CG9901	FBgn0011742	CG9901	Actin-related protein 2	Arp2	terminal button organization ; GO:0072553 | inferred from mutant phenotype <newline> actin filament organization ; GO:0007015 | inferred from mutant phenotype <newline> Arp2/3 complex-mediated actin nucleation ; GO:0034314 | inferred from biological aspect of ancestor with PANTHER:PTN000233596 <newline> regulation of filopodium assembly ; GO:0051489 | inferred from mutant phenotype <newline> positive regulation of synaptic growth at neuromuscular junction ; GO:0045887 | inferred from mutant phenotype <newline> Arp2/3 complex-mediated actin nucleation ; GO:0034314 | inferred from sequence or structural similarity with HGNC:169 <newline> cell morphogenesis ; GO:0000902 | inferred from mutant phenotype	actin filament ; GO:0005884 | inferred from sequence or structural similarity with SGD:S000002187 <newline> Arp2/3 protein complex ; GO:0005885 | inferred from sequence or structural similarity with HGNC:169 <newline> Arp2/3 protein complex ; GO:0005885 | inferred from biological aspect of ancestor with PANTHER:PTN000233596 <newline> actin cortical patch ; GO:0030479 | inferred from biological aspect of ancestor with PANTHER:PTN000233596	ATP binding ; GO:0005524 | inferred from electronic annotation with InterPro:IPR027306 <newline> actin filament binding ; GO:0051015 | contributes_to inferred from biological aspect of ancestor with PANTHER:PTN000233596 <newline> structural constituent of cytoskeleton ; GO:0005200 | inferred from sequence or structural similarity with HGNC:169	Hsap\ACTR2	X

L3	Pp2B-14D	FBgn0011826	CG9842	Protein phosphatase 2B at 14D	Pp2B-14D	sleep ; GO:0030431 | inferred from direct assay <newline> regulation of embryonic development ; GO:0045995 | inferred from mutant phenotype <newline> meiotic cell cycle ; GO:0051321 | inferred from genetic interaction with sra <newline> protein dephosphorylation ; GO:0006470 | inferred from sequence or structural similarity <newline> female meiotic nuclear division ; GO:0007143 | inferred from mutant phenotype <newline> wing disc development ; GO:0035220 | inferred from mutant phenotype <newline> calcineurin-mediated signaling ; GO:0097720 | inferred from biological aspect of ancestor with PANTHER:PTN000746105	cytoplasm ; GO:0005737 | inferred from biological aspect of ancestor with PANTHER:PTN000746105 <newline> calcineurin complex ; GO:0005955 | inferred from biological aspect of ancestor with PANTHER:PTN000746105 <newline> calcineurin complex ; GO:0005955 | inferred from sequence or structural similarity	protein serine/threonine phosphatase activity ; GO:0004722 | inferred from sequence or structural similarity <newline> calmodulin binding ; GO:0005516 | traceable author statement <newline> calmodulin-dependent protein phosphatase activity ; GO:0033192 | inferred from mutant phenotype <newline> calmodulin binding ; GO:0005516 | inferred from biological aspect of ancestor with PANTHER:PTN000746105 <newline> protein binding ; GO:0005515 | inferred from physical interaction with sra <newline> calcium-dependent protein serine/threonine phosphatase activity ; GO:0004723 | inferred from sequence or structural similarity <newline> protein binding ; GO:0005515 | inferred from physical interaction with UniProtKB:Q9XZL8 <newline> calmodulin-dependent protein phosphatase activity ; GO:0033192 [illegible]	Hsap\PPP3CB <newline> Hsap\PPP3CC <newline> Hsap\PPP3CA	X
L3	CanA-14F	FBgn0267912	CG9819	Calcineurin A at 14F	CanA-14F	calcineurin-mediated signaling ; GO:0097720 | inferred from biological aspect of ancestor with PANTHER:PTN000746105 <newline> sleep ; GO:0030431 | inferred from direct assay	cytoplasm ; GO:0005737 | inferred from biological aspect of ancestor with PANTHER:PTN000746105 <newline> calcineurin complex ; GO:0005955 | inferred from biological aspect of ancestor with PANTHER:PTN000746105	protein binding ; GO:0005515 | inferred from physical interaction with UniProtKB:Q9XZL8 <newline> calmodulin-dependent protein phosphatase activity ; GO:0033192 | inferred from mutant phenotype <newline> calmodulin binding ; GO:0005516 | inferred from biological aspect of ancestor with PANTHER:PTN000746105 <newline> calmodulin-dependent protein phosphatase activity ; GO:0033192 | inferred from biological aspect of ancestor with PANTHER:PTN000746105 <newline> protein serine/threonine phosphatase activity ; GO:0004722 | inferred from sequence or structural similarity	Hsap\PPP3CC <newline> Hsap\PPP3CB <newline> Hsap\PPP3CA	X
L3	CG13014	FBgn0030759	CG13014	-	CG13014	-	-	-	-	X

L3	CG9802	FBgn0015615	CG9802	Structural maintenance of chromosomes 3	SMC3	chromosome organization ; GO:0051276 | inferred from electronic annotation with InterPro:IPR010935, InterPro:IPR036277 <newline> establishment of imaginal disc-derived wing hair orientation ; GO:0001737 | inferred from mutant phenotype <newline> imaginal disc-derived wing morphogenesis ; GO:0007476 | inferred from mutant phenotype	cohesin complex ; GO:0008278 | inferred from direct assay <newline> cohesin complex ; GO:0008278 | non-traceable author statement	ATP binding ; GO:0005524 | inferred from electronic annotation with InterPro:IPR010935 <newline> protein heterodimerization activity ; GO:0046982 | inferred from sequence or structural similarity with HGNC:2468 <newline> chromatin binding ; GO:0003682 | inferred from sequence or structural similarity with MGI:MGI:1339795	Hsap\SMC3	X
L3	CG4443	FBgn0267384	CG4443	Ubiquitin conjugating enzyme 7	Ubc7	protein polyubiquitination ; GO:0000209 | inferred from sequence or structural similarity with HGNC:12482 <newline> positive regulation of innate immune response ; GO:0045089 | inferred from high throughput mutant phenotype <newline> ubiquitin-dependent protein catabolic process ; GO:0006511 | inferred from biological aspect of ancestor with PANTHER:PTN000629793 <newline> protein polyubiquitination ; GO:0000209 | inferred from biological aspect of ancestor with PANTHER:PTN000629793 <newline> ubiquitin-dependent ERAD pathway ; GO:0030433 | inferred from biological aspect of ancestor with PANTHER:PTN000629877 <newline> defense response to Gram-negative bacterium ; GO:0050829 | inferred from high throughput mutant phenotype	cytosol ; GO:0005829 | inferred from biological aspect of ancestor with PANTHER:PTN000629877 <newline> endoplasmic reticulum ; GO:0005783 | inferred from biological aspect of ancestor with PANTHER:PTN000629877	ubiquitin conjugating enzyme activity ; GO:0061631 | inferred from biological aspect of ancestor with PANTHER:PTN000629793 <newline> ubiquitin-protein transferase activity ; GO:0004842 | inferred from sequence or structural similarity with HGNC:12482	Hsap\UBE2G1 <newline> Hsap\UBE2G2	X

L3	CG9784	FBgn0030761	CG9784	-	CG9784	inositol phosphate dephosphorylation ; GO:0046855 | inferred from biological aspect of ancestor with PANTHER:PTN000127779 <newline> positive regulation of flagellated sperm motility ; GO:1902093 | inferred from biological aspect of ancestor with PANTHER:PTN002598040 <newline> phosphatidylinositol dephosphorylation ; GO:0046856 | inferred from biological aspect of ancestor with PANTHER:PTN000127779	nucleus ; GO:0005634 | inferred from biological aspect of ancestor with PANTHER:PTN002598040 <newline> ruffle ; GO:0001726 | inferred from biological aspect of ancestor with PANTHER:PTN000128081 <newline> dendrite ; GO:0030425 | inferred from biological aspect of ancestor with PANTHER:PTN002598040 <newline> axon ; GO:0030424 | inferred from biological aspect of ancestor with PANTHER:PTN002598040 <newline> cytoplasm ; GO:0005737 | inferred from biological aspect of ancestor with PANTHER:PTN000128081 <newline> neuronal cell body ; GO:0043025 | inferred from biological aspect of ancestor with PANTHER:PTN002598040 <newline> non-motile cilium ; GO:0097730 | inferred from biological aspect of ancestor with PANTHER:PTN002598040 [illegible]	phosphatidylinositol-4,5-bisphosphate 5-phosphatase activity ; GO:0004439 | inferred from biological aspect of ancestor with PANTHER:PTN000127779	Hsap\SH2D1B <newline> Hsap\INPPL1 <newline> Hsap\INPP5D <newline> Hsap\INPP5B <newline> Hsap\INPP5K <newline> Hsap\INPP5A <newline> Hsap\INPP5J <newline> Hsap\INPP5E <newline> Hsap\OCRL	X

L3	Nup153	FBgn0061200	CG4453	Nucleoporin 153kD	Nup153	chromatin organization ; GO:0006325 | inferred from mutant phenotype <newline> dosage compensation ; GO:0007549 | inferred from mutant phenotype <newline> positive regulation of transcription by RNA polymerase II ; GO:0045944 | inferred from mutant phenotype <newline> NLS-bearing protein import into nucleus ; GO:0006607 | inferred from mutant phenotype <newline> chromatin remodeling ; GO:0006338 | inferred from mutant phenotype <newline> protein import into nucleus ; GO:0006606 | inferred from mutant phenotype <newline> nuclear pore organization ; GO:0006999 | inferred from mutant phenotype <newline> protein import into nucleus ; GO:0006606 | inferred from biological aspect of ancestor with PANTHER:PTN000573960 <newline> regulation of transcription, DNA-templated ; GO:0006355 | inferred from mutant phenotype	nuclear euchromatin ; GO:0005719 | inferred from direct assay <newline> nuclear periphery ; GO:0034399 | inferred from direct assay <newline> nuclear envelope ; GO:0005635 | inferred from direct assay <newline> nuclear pore nuclear basket ; GO:0044615 | inferred from biological aspect of ancestor with PANTHER:PTN000573960 <newline> nuclear pore central transport channel ; GO:0044613 | inferred from mutant phenotype <newline> nucleus ; GO:0005634 | inferred from direct assay <newline> nuclear pore central transport channel ; GO:0044613 | inferred from biological aspect of ancestor with PANTHER:PTN000573960	nuclear localization sequence binding ; GO:0008139 | inferred from biological aspect of ancestor with PANTHER:PTN000573960 <newline> chromatin DNA binding ; GO:0031490 | inferred from direct assay <newline> structural constituent of nuclear pore ; GO:0017056 | inferred from biological aspect of ancestor with PANTHER:PTN000573960 <newline> structural constituent of nuclear pore ; GO:0017056 | inferred from mutant phenotype	Hsap\NUP153	X

L3	mbt	FBgn0025743	CG18582	mushroom bodies tiny	mbt	compound eye photoreceptor cell differentiation ; GO:0001751 | inferred from mutant phenotype <newline> activation of protein kinase activity ; GO:0032147 | inferred from biological aspect of ancestor with PANTHER:PTN000684825 <newline> regulation of cell-cell adhesion mediated by cadherin ; GO:2000047 | inferred from mutant phenotype <newline> compound eye development ; GO:0048749 | inferred from mutant phenotype <newline> positive regulation of compound eye photoreceptor development ; GO:0045315 | inferred from mutant phenotype <newline> signal transduction by protein phosphorylation ; GO:0023014 | inferred from biological aspect of ancestor with PANTHER:PTN000684825 <newline> peptidyl-serine phosphorylation ; GO:0018105 | inferred from direct assay <newline> activation of MAPK activity ; GO:0000187 | traceable [illegible]	plasma membrane ; GO:0005886 | colocalizes_with inferred from direct assay <newline> cytoplasm ; GO:0005737 | inferred from biological aspect of ancestor with PANTHER:PTN000684825 <newline> cell-cell adherens junction ; GO:0005913 | inferred from direct assay <newline> adherens junction ; GO:0005912 | inferred from direct assay <newline> cytoplasm ; GO:0005737 | inferred from direct assay	ATP binding ; GO:0005524 | inferred from mutant phenotype <newline> protein binding ; GO:0005515 | inferred from physical interaction with UniProtKB:P40793 <newline> Rho GTPase binding ; GO:0017048 | inferred from physical interaction with Cdc42 <newline> protein serine/threonine kinase activity ; GO:0004674 | inferred from biological aspect of ancestor with PANTHER:PTN000684825 <newline> protein serine/threonine kinase activity ; GO:0004674 | inferred from mutant phenotype <newline> protein serine/threonine kinase activity ; GO:0004674 | inferred from direct assay <newline> protein kinase activity ; GO:0004672 | inferred from direct assay	Hsap\PAK4 <newline> Hsap\MAP4K1 <newline> Hsap\STK25 <newline> Hsap\MAP4K3 <newline> Hsap\STK24 <newline> Hsap\STK3 <newline> Hsap\PAK1 <newline> Hsap\MAP4K2 <newline> Hsap\BUB1B-PAK6 <newline> Hsap\PAK6 <newline> Hsap\MAP3K19 <newline> Hsap\STK4 <newline> Hsap\MAP3K1 <newline> Hsap\MAP3K2 <newline> Hsap\TAOK2 <newline> Hsap\MAP3K3 <newline> Hsap\OXSR1 <newline> Hsap\TAOK3 <newline> Hsap\MAP3K4 <newline> Hsap\PAK5 <newline> Hsap\PAK3 <newline> Hsap\STK39 <newline> Hsap\TAOK1 <newline> Hsap\PAK2 <newline> Hsap\MYLK3	X
L3	CG9782	FBgn0030763	CG9782	-	CG9782	-	-	-	-	X

L3	CG9774	FBgn0026181	CG9774	Rho kinase	Rok	regulation of phosphorylation ; GO:0042325 | inferred from direct assay <newline> epidermis development ; GO:0008544 | inferred from mutant phenotype <newline> mitotic cell cycle ; GO:0000278 | inferred from mutant phenotype <newline> regulation of axonogenesis ; GO:0050770 | inferred from genetic interaction with LIMK1 <newline> actin cytoskeleton reorganization ; GO:0031532 | inferred from mutant phenotype <newline> morphogenesis of embryonic epithelium ; GO:0016331 | inferred from mutant phenotype <newline> regulation of planar cell polarity pathway involved in axis elongation ; GO:2000040 | inferred from direct assay <newline> establishment of ommatidial planar polarity ; GO:0042067 | inferred from mutant phenotype <newline> Rho protein signal transduction ; GO:0007266 | inferred from physical interaction with Rho1 <newline> regulation of	cytoskeleton ; GO:0005856 | inferred from biological aspect of ancestor with PANTHER:PTN002743840 <newline> cytoplasm ; GO:0005737 | inferred from biological aspect of ancestor with PANTHER:PTN002743840 <newline> cell cortex ; GO:0005938 | inferred from direct assay <newline> apicomedial cortex ; GO:0106037 | inferred from direct assay <newline> apical cortex ; GO:0045179 | colocalizes_with inferred from direct assay <newline> cell-cell contact zone ; GO:0044291 | inferred from direct assay	ATP binding ; GO:0005524 | inferred from electronic annotation with InterPro:IPR000719, InterPro:IPR000961, InterPro:IPR002290, InterPro:IPR017441 <newline> GTPase binding ; GO:0051020 | inferred from physical interaction with Rho1 <newline> protein binding ; GO:0005515 | inferred from physical interaction with UniProtKB:A1Z9P3 <newline> Rho-dependent protein serine/threonine kinase activity ; GO:0072518 | inferred from biological aspect of ancestor with PANTHER:PTN000542090 <newline> protein serine/threonine kinase activity ; GO:0004674 | inferred from direct assay <newline> GTP-Rho binding ; GO:0017049 | inferred from biological aspect of ancestor with PANTHER:PTN000542090 <newline> protein serine/threonine kinase activity ;	Hsap\TACC3 <newline> Hsap\ROCK1 <newline> Hsap\CDC42BPB <newline> Hsap\ROCK2	X
L3	CG9777	FBgn0030764	CG9777	-	CG9777	biological_process ; GO:0008150 | no biological data available	cellular_component ; GO:0005575 | no biological data available	molecular_function ; GO:0003674 | no biological data available	-	X
L3	RpS19a	FBgn0010412	CG4464	Ribosomal protein S19a	RpS19a	translation ; GO:0006412 | inferred from electronic annotation with InterPro:IPR001266, InterPro:IPR018277 <newline> ribosomal small subunit assembly ; GO:0000028 | inferred from biological aspect of ancestor with PANTHER:PTN000194951 <newline> cytoplasmic translation ; GO:0002181 | traceable author statement <newline> cytoplasmic translation ; GO:0002181 | inferred by curator from GO:0022626	cytosolic ribosome ; GO:0022626 | inferred from high throughput direct assay <newline> cytosolic small ribosomal subunit ; GO:0022627 | inferred from biological aspect of ancestor with PANTHER:PTN000194951 <newline> cytosolic small ribosomal subunit ; GO:0022627 | traceable author statement	structural constituent of ribosome ; GO:0003735 | traceable author statement <newline> structural constituent of ribosome ; GO:0003735 | inferred from high throughput direct assay <newline> structural constituent of ribosome ; GO:0003735 | inferred from biological aspect of ancestor with PANTHER:PTN000194951	Hsap\RPS19	X

L3	CG9742	FBgn0261791	CG9742	Small nuclear ribonucleoprotein G	SNRPG	spliceosomal snRNP assembly ; GO:0000387 | inferred from electronic annotation with InterPro:IPR034098 <newline> mRNA splicing, via spliceosome ; GO:0000398 | inferred by curator from GO:0071011,GO:0071013	U2 snRNP ; GO:0005686 | inferred from biological aspect of ancestor with PANTHER:PTN000058285 <newline> U12-type spliceosomal complex ; GO:0005689 | inferred from biological aspect of ancestor with PANTHER:PTN000058284 <newline> U4 snRNP ; GO:0005687 | inferred from biological aspect of ancestor with PANTHER:PTN000058285 <newline> small nuclear ribonucleoprotein complex ; GO:0030532 | inferred from sequence or structural similarity with SGD:S000002965 <newline> small nuclear ribonucleoprotein complex ; GO:0030532 | inferred from direct assay <newline> nucleus ; GO:0005634 | inferred from direct assay <newline> catalytic step 2 spliceosome ; GO:0071013 | inferred from biological aspect of ancestor with PANTHER:PTN000058284 <newline> precatalytic spliceosome ; GO:0071011 | [illegible]	RNA binding ; GO:0003723 | inferred from sequence or structural similarity with HGNC:11163 <newline> RNA binding ; GO:0003723 | contributes_to inferred from biological aspect of ancestor with PANTHER:PTN000058283	Hsap\SNRPG <newline> Hsap\LSM1 <newline> Hsap\LSM3 <newline> Hsap\LSM8 <newline> Hsap\LSM7 <newline> Hsap\LSM5	X

L3	mthl1	FBgn0030766	CG4521	methuselah-like 1	mthl1	cell surface receptor signaling pathway ; GO:0007166 | inferred from electronic annotation with InterPro:IPR017981 <newline> G protein-coupled receptor signaling pathway ; GO:0007186 | inferred from sequence or structural similarity <newline> gastrulation ; GO:0007369 | inferred from mutant phenotype <newline> determination of adult lifespan ; GO:0008340 | inferred from sequence or structural similarity with UniProtKB:O97148 <newline> G protein-coupled receptor signaling pathway ; GO:0007186 | inferred from direct assay <newline> G protein-coupled receptor signaling pathway ; GO:0007186 | inferred from sequence or structural similarity with mth <newline> mesectoderm development ; GO:0048383 | inferred from mutant phenotype <newline> actin-mediated cell contraction ; GO:0070252 | inferred from genetic interaction with fog [illegible]	integral component of membrane ; GO:0016021 | inferred from sequence or structural similarity <newline> integral component of membrane ; GO:0016021 | inferred from sequence or structural similarity with mth	G protein-coupled receptor activity ; GO:0004930 | inferred from sequence or structural similarity with mth <newline> G protein-coupled receptor activity ; GO:0004930 | inferred from sequence or structural similarity <newline> G protein-coupled receptor activity ; GO:0004930 | inferred from physical interaction with fog	Hsap\ADGRE1 <newline> Hsap\ADGRL4 <newline> Hsap\ADGRD1 <newline> Hsap\ADGRL2 <newline> Hsap\ADGRE3 <newline> Hsap\FBN3 <newline> Hsap\ADGRG2 <newline> Hsap\ADGRF2 <newline> Hsap\ADGRD2 <newline> Hsap\ADGRG5 <newline> Hsap\ADGRG1 <newline> Hsap\ADGRL1 <newline> Hsap\ADGRF1 <newline> Hsap\ADGRE5 <newline> Hsap\ADGRE2 <newline> Hsap\ADGRG6 <newline> Hsap\ADGRG3 <newline> Hsap\ADGRG4 <newline> Hsap\ADGRL3 <newline> Hsap\ADGRF3 <newline> Hsap\ADGRG7 <newline> Hsap\ADGRF4 <newline> Hsap\ADGRF5	X
L3	CG42512	FBgn0260238	CG42512	-	CG42512	-	-	-	-	X
L3	CG32573	FBgn0052573	CG32573	-	CG32573	-	-	-	-	X
L3	CG9723	FBgn0030768	CG9723	-	CG9723	-	nuclear envelope ; GO:0005635 | inferred from biological aspect of ancestor with PANTHER:PTN001006264	-	Hsap\NEMP1 <newline> Hsap\NEMP2	X

<table>
<tr><td>L3</td><td>r</td><td>FBgn0003189</td><td>CG18572</td><td>rudimentary</td><td>r</td><td>de novo' pyrimidine nucleobase biosynthetic process ; GO:0006207 | inferred from mutant phenotype <newline> glutamine metabolic process ; GO:0006541 | inferred by curator from GO:0004088 <newline> citrulline biosynthetic process ; GO:0019240 | inferred from biological aspect of ancestor with PANTHER:PTN000150227 <newline> 'de novo' pyrimidine nucleobase biosynthetic process ; GO:0006207 | inferred from biological aspect of ancestor with PANTHER:PTN000150227 <newline> glutamine metabolic process ; GO:0006541 | inferred from biological aspect of ancestor with PANTHER:PTN000150223 <newline> nitrogen compound metabolic process ; GO:0006807 | inferred from biological aspect of ancestor with PANTHER:PTN000150222 <newline> UTP biosynthetic process ; GO:0006228 | inferred from biological aspect of ancestor with PANTHER:PTN000150227 <newline> [illegible]</td><td>cytoplasm ; GO:0005737 | inferred from biological aspect of ancestor with PANTHER:PTN000150222 <newline> cytosol ; GO:0005829 | inferred from biological aspect of ancestor with PANTHER:PTN000150227</td><td>ATP binding ; GO:0005524 | inferred from electronic annotation with InterPro:IPR005479, InterPro:IPR011761, InterPro:IPR013815, InterPro:IPR013816 <newline> amino acid binding ; GO:0016597 | inferred from electronic annotation with InterPro:IPR006130, InterPro:IPR006131 <newline> metal ion binding ; GO:0046872 | inferred from electronic annotation with InterPro:IPR011761 <newline> dihydroorotase activity ; GO:0004151 | inferred from mutant phenotype <newline> aspartate carbamoyltransferase activity ; GO:0004070 | inferred from biological aspect of ancestor with PANTHER:PTN000150227 <newline> dihydroorotase activity ; GO:0004151 | inferred from biological aspect of ancestor with PANTHER:PTN000150227 [illegible]</td><td>Hsap\CAD <newline> Hsap\CPS1</td><td>X</td></tr>
<tr><td>L3</td><td>CG15865</td><td>FBgn0015336</td><td>CG15865</td><td>-</td><td>CG15865</td><td>-</td><td>-</td><td>-</td><td>Hsap\C2orf54 <newline> Hsap\ITPRIP <newline> Hsap\ITPRIPL2 <newline> Hsap\MAB21L2 <newline> Hsap\MB21D2 <newline> Hsap\ITPRIPL1 <newline> Hsap\MAB21L3 <newline> Hsap\CGAS <newline> Hsap\MAB21L1 <newline> Hsap\TMEM102</td><td>X</td></tr>
<tr><td>L3</td><td>CG13012</td><td>FBgn0030769</td><td>CG13012</td><td>-</td><td>CG13012</td><td>-</td><td>-</td><td>-</td><td>-</td><td>X</td></tr>
<tr><td>L3</td><td>CG13010</td><td>FBgn0030770</td><td>CG13010</td><td>-</td><td>CG13010</td><td>biological_process ; GO:0008150 | no biological data available</td><td>cellular_component ; GO:0005575 | no biological data available</td><td>molecular_function ; GO:0003674 | no biological data available</td><td>-</td><td>X</td></tr>
</table>

L3	sing	FBgn0261245	CG13011	singles bar	sing	adult somatic muscle development ; GO:0007527 | inferred from mutant phenotype <newline> myoblast fusion ; GO:0007520 | inferred from mutant phenotype	integral component of membrane ; GO:0016021 | inferred from sequence model <newline> integral component of membrane ; GO:0016021 | inferred from biological aspect of ancestor with PANTHER:PTN001103553	molecular_function ; GO:0003674 | no biological data available	Hsap\PLP2 <newline> Hsap\CMTM1 <newline> Hsap\MAL <newline> Hsap\CMTM5 <newline> Hsap\CMTM4 <newline> Hsap\CMTM2 <newline> Hsap\MALL <newline> Hsap\CMTM7 <newline> Hsap\CMTM3 <newline> Hsap\PLLP <newline> Hsap\MAL2 <newline> Hsap\MARVELD1 <newline> Hsap\CMTM8 <newline> Hsap\CKLF <newline> Hsap\CMTM6	X
L3	Axs	FBgn0000152	CG9703	Abnormal X segregation	Axs	female meiosis chromosome segregation ; GO:0016321 | inferred from mutant phenotype <newline> distributive segregation ; GO:0032837 | inferred from mutant phenotype inferred from genetic interaction with Mps1 inferred from genetic interaction with ncd <newline> meiotic spindle organization ; GO:0000212 | inferred from mutant phenotype <newline> distributive segregation ; GO:0032837 | inferred from mutant phenotype	integral component of membrane ; GO:0016021 | inferred from sequence or structural similarity <newline> cell cortex ; GO:0005938 | inferred from direct assay <newline> spindle ; GO:0005819 | inferred from direct assay <newline> endoplasmic reticulum ; GO:0005783 | inferred from direct assay <newline> nuclear outer membrane ; GO:0005640 | inferred from direct assay	-	Hsap\ANO10 <newline> Hsap\ANO8	X
L3	Sep4	FBgn0259923	CG9699	Septin 4	Sep4	cytoskeleton-dependent cytokinesis ; GO:0061640 | inferred from biological aspect of ancestor with PANTHER:PTN000430171	microtubule cytoskeleton ; GO:0015630 | inferred from biological aspect of ancestor with PANTHER:PTN000430171 <newline> septin complex ; GO:0031105 | inferred from biological aspect of ancestor with PANTHER:PTN000430171 <newline> septin ring ; GO:0005940 | inferred from biological aspect of ancestor with PANTHER:PTN000430171	GTP binding ; GO:0005525 | inferred from electronic annotation with InterPro:IPR016491, InterPro:IPR030379 <newline> GTPase activity ; GO:0003924 | inferred from sequence or structural similarity with pnut <newline> GTPase activity ; GO:0003924 | inferred from biological aspect of ancestor with PANTHER:PTN000430171	Hsap\SEPT2 <newline> Hsap\SEPT8 <newline> Hsap\SEPT10 <newline> Hsap\SEPT4 <newline> Hsap\SEPT9 <newline> Hsap\SEPT3 <newline> Hsap\SEPT1 <newline> Hsap\SEPT11 <newline> Hsap\SEPT12 <newline> Hsap\SEPT5 <newline> Hsap\SEPT6 <newline> Hsap\TMEM250 <newline> Hsap\SEPT7 <newline> Hsap\SEPT14	X

L3	CG4678	FBgn0030778	CG4678	-	CG4678	peptide metabolic process ; GO:0006518 | inferred from biological aspect of ancestor with PANTHER:PTN000164240 <newline> protein processing ; GO:0016485 | inferred from biological aspect of ancestor with PANTHER:PTN000164240	extracellular space ; GO:0005615 | inferred from biological aspect of ancestor with PANTHER:PTN000164240	zinc ion binding ; GO:0008270 | inferred from electronic annotation with InterPro:IPR000834 <newline> metallocarboxypeptidase activity ; GO:0004181 | inferred from biological aspect of ancestor with PANTHER:PTN000164240 <newline> serine-type carboxypeptidase activity ; GO:0004185 | inferred from electronic annotation with InterPro:IPR015567	Hsap\CPXM1 <newline> Hsap\CPN1 <newline> Hsap\CPXM2 <newline> Hsap\CPD <newline> Hsap\CPM <newline> Hsap\CPE <newline> Hsap\AEBP1 <newline> Hsap\CPZ	X
L4	CG15059	FBgn0030905	CG15059	-	CG15059	-	-	-	-	X
L4	upd3	FBgn0053542	CG33542	unpaired 3	upd3	embryonic development via the syncytial blastoderm ; GO:0001700 | inferred from electronic annotation with InterPro:IPR031901 <newline> receptor signaling pathway via JAK-STAT ; GO:0007259 | inferred from genetic interaction with Stat92E <newline> receptor signaling pathway via JAK-STAT ; GO:0007259 | inferred from mutant phenotype <newline> oogenesis ; GO:0048477 | inferred from mutant phenotype <newline> intestinal epithelial structure maintenance ; GO:0060729 | inferred from mutant phenotype <newline> paracrine signaling ; GO:0038001 | inferred from direct assay <newline> regulation of imaginal disc-derived wing size ; GO:0044719 | inferred from mutant phenotype <newline> positive regulation of cell population proliferation ; GO:0008284 | inferred from mutant phenotype <newline> receptor signaling pathway via JAK-STAT ; GO:0007259 | inferred from direct [illegible]	extracellular space ; GO:0005615 | inferred from direct assay <newline> extracellular space ; GO:0005615 | inferred from sequence model	cytokine activity ; GO:0005125 | inferred from direct assay	-	X

L4	upd1	FBgn0004956	CG5993	unpaired 1	upd1	embryonic development via the syncytial blastoderm ; GO:0001700 | inferred from electronic annotation with InterPro:IPR031901 <newline> long-term memory ; GO:0007616 | inferred from mutant phenotype <newline> negative regulation of innate immune response ; GO:0045824 | inferred from high throughput mutant phenotype <newline> regulation of embryonic cell shape ; GO:0016476 | inferred from mutant phenotype <newline> receptor signaling pathway via JAK-STAT ; GO:0007259 | inferred from direct assay <newline> sex determination, establishment of X:A ratio ; GO:0007540 | non-traceable author statement <newline> ovarian follicle cell development ; GO:0030707 | inferred from mutant phenotype <newline> synaptic target recognition ; GO:0008039 | inferred from mutant phenotype <newline> eye-antennal disc morphogenesis ; GO:0007455 | inferred from mutant [illegible]	extracellular region ; GO:0005576 | inferred from direct assay <newline> apical part of cell ; GO:0045177 | inferred from direct assay <newline> extracellular space ; GO:0005615 | inferred from direct assay	extracellular matrix binding ; GO:0050840 | inferred from direct assay <newline> cytokine activity ; GO:0005125 | inferred from direct assay <newline> cytokine activity ; GO:0005125 | inferred from genetic interaction with dome <newline> protein binding ; GO:0005515 | inferred from physical interaction with UniProtKB:M9NF14	-	X
L4	CG6023	FBgn0030912	CG6023	-	CG6023	-	-	-	-	X
L4	CG6123	FBgn0030913	CG6123	-	CG6123	-	-	-	-	X
L4	CG6106	FBgn0030914	CG6106	-	CG6106	allantoin catabolic process ; GO:0000256 | inferred from electronic annotation with InterPro:IPR017593 <newline> purine nucleobase catabolic process ; GO:0006145 | inferred from biological aspect of ancestor with PANTHER:PTN000909483	cytoplasm ; GO:0005737 | inferred from biological aspect of ancestor with PANTHER:PTN000909483	zinc ion binding ; GO:0008270 | inferred from electronic annotation with InterPro:IPR017593 <newline> cobalt ion binding ; GO:0050897 | inferred from electronic annotation with InterPro:IPR017593 <newline> allantoinase activity ; GO:0004038 | inferred from biological aspect of ancestor with PANTHER:PTN000909483	Hsap\CRMP1 <newline> Hsap\DPYS <newline> Hsap\DPYSL2 <newline> Hsap\DPYSL5 <newline> Hsap\DPYSL3 <newline> Hsap\DPYSL4	X

L4	CG6103	FBgn0265784	CG6103	Cyclic-AMP response element binding protein B	CrebB	thermosensory behavior ; GO:0040040 | inferred from mutant phenotype <newline> long-term memory ; GO:0007616 | inferred from direct assay <newline> long-term memory ; GO:0007616 | inferred from mutant phenotype <newline> long-term memory ; GO:0007616 | inferred from expression pattern <newline> positive regulation of feeding behavior ; GO:2000253 | inferred from genetic interaction with sNPF-R <newline> sleep ; GO:0030431 | inferred from mutant phenotype <newline> positive regulation of transcription by RNA polymerase II ; GO:0045944 | inferred from mutant phenotype <newline> long-term memory ; GO:0007616 | traceable author statement <newline> learning or memory ; GO:0007611 | non-traceable author statement <newline> locomotor rhythm ; GO:0045475 | non-traceable author statement <newline> anesthesia-resistant memory ; GO:0007615 | [illegible]	nucleus ; GO:0005634 | inferred from direct assay <newline> transcription factor complex ; GO:0005667 | inferred from biological aspect of ancestor with PANTHER:PTN001119557	DNA-binding transcription factor activity ; GO:0003700 | inferred from electronic annotation with InterPro:IPR001630, InterPro:IPR004827 <newline> DNA binding ; GO:0003677 | inferred from mutant phenotype <newline> sequence-specific DNA binding ; GO:0043565 | inferred from direct assay	Hsap\ATF2 <newline> Hsap\ATF7 <newline> Hsap\CREB1 <newline> Hsap\ATF1 <newline> Hsap\CREM <newline> Hsap\FOSL2 <newline> Hsap\FOSL1 <newline> Hsap\CREB5 <newline> Hsap\FOS	X

L4	por	FBgn0004957	CG6205	porcupine	por	protein lipidation ; GO:0006497 | inferred from sequence or structural similarity with UniProtKB:Q9JJJ7 <newline> protein maturation ; GO:0051604 | inferred from mutant phenotype <newline> Wnt protein secretion ; GO:0061355 | inferred from mutant phenotype <newline> protein palmitoylation ; GO:0018345 | inferred from mutant phenotype <newline> Wnt signaling pathway ; GO:0016055 | inferred from sequence or structural similarity with UniProtKB:Q9JJJ7 <newline> protein localization ; GO:0008104 | inferred from mutant phenotype <newline> protein secretion ; GO:0009306 | inferred from mutant phenotype <newline> protein palmitoleylation ; GO:0045234 | inferred from sequence or structural similarity with UniProtKB:Q9JJJ7 <newline> regulation of Wnt signaling pathway ; GO:0030111 | inferred from mutant phenotype	integral component of endoplasmic reticulum membrane ; GO:0030176 | non-traceable author statement <newline> endoplasmic reticulum ; GO:0005783 | inferred from direct assay	palmitoyltransferase activity ; GO:0016409 | inferred from mutant phenotype <newline> palmitoleoyltransferase activity ; GO:1990698 | inferred from sequence or structural similarity with UniProtKB:Q9JJJ7 <newline> Wnt-protein binding ; GO:0017147 | inferred from physical interaction with UniProtKB:P09615 inferred from physical interaction with UniProtKB:P28466	Hsap\PORCN	X
L4	CG6179	FBgn0030915	CG6179	-	CG6179	negative regulation of catalytic activity ; GO:0043086 | inferred from sequence or structural similarity with UniProtKB:Q9Y314 <newline> mRNA splicing, via spliceosome ; GO:0000398 | inferred by curator from GO:0071013 <newline> negative regulation of nitric-oxide synthase activity ; GO:0051001 | inferred from sequence or structural similarity with UniProtKB:Q9Y314	nucleus ; GO:0005634 | inferred from sequence or structural similarity with UniProtKB:Q9Y314 <newline> cytoplasm ; GO:0005737 | inferred from sequence or structural similarity with UniProtKB:Q9Y314 <newline> nucleus ; GO:0005634 | inferred from biological aspect of ancestor with PANTHER:PTN000316766 <newline> catalytic step 2 spliceosome ; GO:0071013 | inferred from high throughput direct assay	ubiquitin protein ligase activity ; GO:0061630 | inferred from electronic annotation with InterPro:IPR016818 <newline> RNA binding ; GO:0003723 | inferred from sequence or structural similarity with HGNC:17946	Hsap\NOSIP	X

L4	CG6223	FBgn0008635	CG6223	Coat Protein (coatomer) beta	betaCOP	intracellular protein transport ; GO:0006886 | inferred from electronic annotation with InterPro:IPR002553, InterPro:IPR011710, InterPro:IPR016460 <newline> regulation of lipid storage ; GO:0010883 | inferred from direct assay <newline> positive regulation of innate immune response ; GO:0045089 | inferred from high throughput mutant phenotype <newline> endoplasmic reticulum to Golgi vesicle-mediated transport ; GO:0006888 | inferred from biological aspect of ancestor with PANTHER:PTN000067242 <newline> intra-Golgi vesicle-mediated transport ; GO:0006891 | inferred from biological aspect of ancestor with PANTHER:PTN000067242 <newline> defense response to Gram-negative bacterium ; GO:0050829 | inferred from high throughput mutant phenotype <newline> cell morphogenesis ; GO:0000902 | inferred from mutant	Golgi apparatus ; GO:0005794 | inferred from direct assay <newline> COPI vesicle coat ; GO:0030126 | inferred from biological aspect of ancestor with PANTHER:PTN000067242 <newline> cis-Golgi network membrane ; GO:0033106 |	structural molecule activity ; GO:0005198 | inferred from electronic annotation with InterPro:IPR011710	Hsap\COPB1	X
L4	Tsf1	FBgn0022355	CG6186	Transferrin 1	Tsf1	olfactory behavior ; GO:0042048 | inferred from mutant phenotype <newline> response to fungus ; GO:0009620 | inferred from high throughput expression pattern	extracellular space ; GO:0005615 | inferred from electronic annotation with InterPro:IPR016357 <newline> plasma membrane ; GO:0005886 | inferred from high throughput direct assay <newline> extracellular region ; GO:0005576 | inferred from high throughput direct assay	-	Hsap\MELTF <newline> Hsap\TF <newline> Hsap\LTF	X
L4	CG32549	FBgn0052549	CG32549	-	CG32549	-	-	5'-nucleotidase activity ; GO:0008253 | inferred from biological aspect of ancestor with PANTHER:PTN000250728	Hsap\NT5C2 <newline> Hsap\NT5DC1 <newline> Hsap\NT5DC4	X
L4	CG34328	FBgn0085357	CG34328	-	CG34328	biological_process ; GO:0008150 | no biological data available	cellular_component ; GO:0005575 | no biological data available	molecular_function ; GO:0003674 | no biological data available	-	X

L4	CG15056	FBgn0030918	CG15056	-	CG15056	SCF-dependent proteasomal ubiquitin-dependent protein catabolic process ; GO:0031146 | inferred from biological aspect of ancestor with PANTHER:PTN002547163 <newline> ubiquitin-dependent protein catabolic process ; GO:0006511 | inferred from biological aspect of ancestor with PANTHER:PTN002547163	SCF ubiquitin ligase complex ; GO:0019005 | inferred from biological aspect of ancestor with PANTHER:PTN002547163	ubiquitin-protein transferase activity ; GO:0004842 | contributes_to inferred from biological aspect of ancestor with PANTHER:PTN002547163	Hsap\KDM2A <newline> Hsap\FBXL17 <newline> Hsap\FBXL19 <newline> Hsap\FBXL6 <newline> Hsap\KDM2B <newline> Hsap\FBXL13 <newline> Hsap\FBXL4 <newline> Hsap\LRRC29 <newline> Hsap\FBXL15	X
L4	CG42323	FBgn0259223	CG42323	-	CG42323	biological_process ; GO:0008150 | no biological data available	cellular_component ; GO:0005575 | no biological data available	molecular_function ; GO:0003674 | no biological data available	-	X
L4	CG32548	FBgn0052548	CG32548	-	CG32548	-	-	-	-	X
L4	CG6290	FBgn0030921	CG6290	-	CG6290	biological_process ; GO:0008150 | no biological data available	cellular_component ; GO:0005575 | no biological data available	molecular_function ; GO:0003674 | no biological data available	-	X
L4	CG32551	FBgn0052551	CG32551	-	CG32551	biological_process ; GO:0008150 | no biological data available	cellular_component ; GO:0005575 | no biological data available	molecular_function ; GO:0003674 | no biological data available	-	X
L4	CG43841	FBgn0264393	CG43841	-	CG43841	biological_process ; GO:0008150 | no biological data available	cellular_component ; GO:0005575 | no biological data available	molecular_function ; GO:0003674 | no biological data available	-	X
L4	CG32547	FBgn0052547	CG32547	-	CG32547	G protein-coupled receptor signaling pathway ; GO:0007186 | inferred from electronic annotation with InterPro:IPR000276 <newline> G protein-coupled receptor signaling pathway ; GO:0007186 | inferred from sequence model	integral component of membrane ; GO:0016021 | inferred from electronic annotation with InterPro:IPR000276, InterPro:IPR017452 <newline> integral component of membrane ; GO:0016021 | inferred from sequence model	G protein-coupled receptor activity ; GO:0004930 | inferred from electronic annotation with InterPro:IPR000276 <newline> neuropeptide receptor activity ; GO:0008188 | inferred from sequence model	Hsap\GPR83	X
L4	CG43843	FBgn0264395	CG43843	-	CG43843	biological_process ; GO:0008150 | no biological data available	cellular_component ; GO:0005575 | no biological data available	molecular_function ; GO:0003674 | no biological data available	-	X
L4	CG6361	FBgn0030925	CG6361	Hayan	Hayan	proteolysis ; GO:0006508 | inferred from electronic annotation with InterPro:IPR001254, InterPro:IPR001314, InterPro:IPR018114 <newline> proteolysis ; GO:0006508 | inferred from sequence model <newline> regulation of melanization defense response ; GO:0035007 | inferred from mutant phenotype	-	serine-type endopeptidase activity ; GO:0004252 | inferred from electronic annotation with InterPro:IPR001254, InterPro:IPR001314, InterPro:IPR006604, InterPro:IPR018114 <newline> serine-type endopeptidase activity ; GO:0004252 | inferred from sequence model	-	X

L4	psh	FBgn0030926	CG6367	persephone	psh	positive regulation of antifungal peptide production ; GO:0002804 | inferred from mutant phenotype <newline> positive regulation of Toll signaling pathway ; GO:0045752 | inferred from genetic interaction with nec <newline> cellular response to molecule of bacterial origin ; GO:0071219 | inferred from mutant phenotype <newline> innate immune response ; GO:0045087 | inferred from mutant phenotype <newline> positive regulation of Toll signaling pathway ; GO:0045752 | inferred from genetic interaction with SPE <newline> regulation of Toll signaling pathway ; GO:0008592 | inferred from mutant phenotype inferred from genetic interaction with UniProtKB:Q9VER6 <newline> positive regulation of Toll signaling pathway ; GO:0045752 | inferred from mutant phenotype inferred from genetic interaction with grass <newline> defense response to fungus ; GO:0050832 | inferred	extracellular region ; GO:0005576 | inferred from direct assay	serine-type endopeptidase activity ; GO:0004252 | inferred from sequence model <newline> serine-type endopeptidase activity ; GO:0004252 | inferred from mutant phenotype <newline> peptidase activity ; GO:0008233 | inferred from mutant phenotype	-	X
L4	CG15046	FBgn0030927	CG15046	-	CG15046	-	-	-	-	X
L4	CG15044	FBgn0030928	CG15044	-	CG15044	-	-	-	-	X
L4	CG15043	FBgn0030929	CG15043	-	CG15043	-	-	-	-	X
L4	CG6394	FBgn0030930	CG6394	Polypeptide N-Acetylgalactosaminyltransferase 7	Pgant7	oligosaccharide biosynthetic process ; GO:0009312 | inferred from direct assay	Golgi stack ; GO:0005795 | non-traceable author statement <newline> endomembrane system ; GO:0012505 | inferred from high throughput direct assay <newline> Golgi apparatus ; GO:0005794 | inferred from biological aspect of ancestor with PANTHER:PTN000187024	polypeptide N-acetylgalactosaminyltransferase activity ; GO:0004653 | inferred from direct assay	Hsap\POC1B-GALNT4 <newline> Hsap\GALNT18 <newline> Hsap\GALNT6 <newline> Hsap\GALNT10 <newline> Hsap\GALNT9 <newline> Hsap\GALNT15 <newline> Hsap\GALNT3 <newline> Hsap\GALNT13 <newline> Hsap\GALNTL5 <newline> Hsap\GALNT11 <newline> Hsap\GALNT12 <newline> Hsap\GALNT1 <newline> Hsap\GALNT8 <newline> Hsap\GALNT4 <newline> Hsap\GALNT2 <newline> Hsap\GALNT7 <newline> Hsap\GALNT17 <newline> Hsap\GALNT5 <newline> Hsap\GALNTL6 <newline> Hsap\GALNT16 <newline> Hsap\GALNT14	X

L4	CG6318	FBgn0030931	CG6318	X-ray repair cross complementing 2	Xrcc2	DNA repair ; GO:0006281 | inferred from sequence or structural similarity with HGNC:12829 <newline> double-strand break repair via homologous recombination ; GO:0000724 | inferred from biological aspect of ancestor with PANTHER:PTN002505836 <newline> strand invasion ; GO:0042148 | inferred from biological aspect of ancestor with PANTHER:PTN002505836 <newline> DNA recombination ; GO:0006310 | inferred from sequence or structural similarity with HGNC:12829	centrosome ; GO:0005813 | inferred from biological aspect of ancestor with PANTHER:PTN002505836 <newline> Rad51B-Rad51C-Rad51D-XRCC2 complex ; GO:0033063 | inferred from biological aspect of ancestor with PANTHER:PTN002505836 <newline> replication fork ; GO:0005657 | inferred from biological aspect of ancestor with PANTHER:PTN002505836	four-way junction DNA binding ; GO:0000400 | contributes_to inferred from biological aspect of ancestor with PANTHER:PTN002505836 <newline> recombinase activity ; GO:0000150 | inferred from sequence or structural similarity with HGNC:12829	Hsap\XRCC2	X
L4	Wnt5	FBgn0010194	CG6407	Wnt oncogene analog 5	Wnt5	cell fate commitment ; GO:0045165 | inferred from biological aspect of ancestor with PANTHER:PTN000246517 <newline> salivary gland morphogenesis ; GO:0007435 | inferred from mutant phenotype <newline> neuron differentiation ; GO:0030182 | inferred from biological aspect of ancestor with PANTHER:PTN000246517 <newline> chemorepulsion of axon ; GO:0061643 | inferred from mutant phenotype <newline> axon guidance ; GO:0007411 | inferred from mutant phenotype <newline> dendrite guidance ; GO:0070983 | inferred from mutant phenotype <newline> axon guidance ; GO:0007411 | inferred from mutant phenotype inferred from genetic interaction with drl <newline> Wnt signaling pathway ; GO:0016055 | inferred from sequence or structural similarity with wg <newline> determination of muscle attachment site ; GO:0016204 | [illegible]	dendrite ; GO:0030425 | inferred from direct assay <newline> extracellular space ; GO:0005615 | inferred from biological aspect of ancestor with PANTHER:PTN000246517 <newline> extracellular region ; GO:0005576 | inferred from sequence or structural similarity with wg <newline> extracellular region ; GO:0005576 | inferred from direct assay	protein binding ; GO:0005515 | inferred from physical interaction with UniProtKB:Q9VWV9 <newline> frizzled binding ; GO:0005109 | inferred from biological aspect of ancestor with PANTHER:PTN000246517 <newline> signaling receptor binding ; GO:0005102 | inferred from physical interaction with drl <newline> signaling receptor binding ; GO:0005102 | inferred from sequence or structural similarity with wg	Hsap\WNT5B <newline> Hsap\WNT9A <newline> Hsap\WNT1 <newline> Hsap\WNT7B <newline> Hsap\WNT16 <newline> Hsap\WNT10B <newline> Hsap\WNT2 <newline> Hsap\WNT8B <newline> Hsap\WNT6 <newline> Hsap\WNT8A <newline> Hsap\WNT5A <newline> Hsap\WNT11 <newline> Hsap\WNT10A <newline> Hsap\WNT3 <newline> Hsap\WNT4 <newline> Hsap\WNT9B <newline> Hsap\WNT3A <newline> Hsap\WNT7A <newline> Hsap\WNT2B	X

L4	Ggt-1	FBgn0030932	CG6461	gamma-glutamyl transpeptidase	Ggt-1	multicellular organism reproduction ; GO:0032504 | inferred from high throughput expression pattern <newline> glutathione metabolic process ; GO:0006749 | inferred from direct assay <newline> glutathione catabolic process ; GO:0006751 | inferred from biological aspect of ancestor with PANTHER:PTN002269349 <newline> response to light stimulus ; GO:0009416 | inferred from mutant phenotype	extracellular space ; GO:0005615 | inferred from direct assay <newline> plasma membrane ; GO:0005886 | inferred from biological aspect of ancestor with PANTHER:PTN002269349 <newline> extracellular space ; GO:0005615 | inferred from high throughput direct assay	glutathione hydrolase activity ; GO:0036374 | inferred from sequence or structural similarity with HGNC:4250 inferred from direct assay <newline> peptidyltransferase activity ; GO:0000048 | inferred from biological aspect of ancestor with PANTHER:PTN002269349 <newline> glutathione hydrolase activity ; GO:0036374 | inferred from biological aspect of ancestor with PANTHER:PTN002269349	Hsap\LOC102724197 <newline> Hsap\GGT2 <newline> Hsap\LOC102724823 <newline> Hsap\GGT1 <newline> Hsap\GGTLC1 <newline> Hsap\GGT6 <newline> Hsap\GGT7 <newline> Hsap\GGTLC2 <newline> Hsap\GGT5	X
L4	CG6470	FBgn0030933	CG6470	-	CG6470	-	-	nucleic acid binding ; GO:0003676 | inferred from electronic annotation with InterPro:IPR013087	-	X
L4	CG6335	FBgn0027087	CG6335	Histidyl-tRNA synthetase	HisRS	histidyl-tRNA aminoacylation ; GO:0006427 | inferred from biological aspect of ancestor with PANTHER:PTN000159003 <newline> mitochondrial translation ; GO:0032543 | inferred from biological aspect of ancestor with PANTHER:PTN000159004 <newline> histidyl-tRNA aminoacylation ; GO:0006427 | traceable author statement	cytoplasm ; GO:0005737 | inferred from biological aspect of ancestor with PANTHER:PTN000159003 <newline> mitochondrion ; GO:0005739 | inferred from biological aspect of ancestor with PANTHER:PTN000159004 <newline> cytosol ; GO:0005829 | inferred from high throughput direct assay <newline> cytosol ; GO:0005829 | inferred from biological aspect of ancestor with PANTHER:PTN000159003 <newline> mitochondrion ; GO:0005739 | traceable author statement <newline> cytoplasm ; GO:0005737 | traceable author statement	ATP binding ; GO:0005524 | inferred from electronic annotation with InterPro:IPR000738, InterPro:IPR015807 <newline> histidine-tRNA ligase activity ; GO:0004821 | inferred from biological aspect of ancestor with PANTHER:PTN000159003 <newline> histidine-tRNA ligase activity ; GO:0004821 | traceable author statement	Hsap\HARS2 <newline> Hsap\HARS	X

L4	CG15048	FBgn0265598	CG44425	Beadex	Bx	leg disc development ; GO:0035218 | inferred from mutant phenotype <newline> positive regulation of transcription, DNA-templated ; GO:0045893 | inferred from mutant phenotype <newline> imaginal disc-derived wing morphogenesis ; GO:0007476 | inferred from mutant phenotype inferred from genetic interaction with ap <newline> chaeta development ; GO:0022416 | inferred from mutant phenotype <newline> reproductive process ; GO:0022414 | inferred from mutant phenotype <newline> locomotor rhythm ; GO:0045475 | inferred from mutant phenotype <newline> response to cocaine ; GO:0042220 | inferred from mutant phenotype <newline> gastric emptying ; GO:0035483 | inferred from mutant phenotype <newline> imaginal disc-derived leg segmentation ; GO:0036011 | inferred from mutant phenotype	nucleus ; GO:0005634 | non-traceable author statement	protein binding ; GO:0005515 | inferred from physical interaction with pnr inferred from physical interaction with chianti	Hsap\LMO2 <newline> Hsap\LMO3 <newline> Hsap\LMO4 <newline> Hsap\LMO1	X
L4	CG6481	FBgn0030936	CG6481	-	CG6481	-	-	-	-	X
L4	CG15042	FBgn0030937	CG15042	-	CG15042	-	-	-	Hsap\TDRD1 <newline> Hsap\TDRD10 <newline> Hsap\TDRD7 <newline> Hsap\TDRD15 <newline> Hsap\TDRD6	X
L4	CG15047	FBgn0030938	CG15047	-	CG15047	-	-	-	-	X

L4	Bx	FBgn0265598	CG44425	Beadex	Bx	leg disc development ; GO:0035218 | inferred from mutant phenotype <newline> positive regulation of transcription, DNA-templated ; GO:0045893 | inferred from mutant phenotype <newline> imaginal disc-derived wing morphogenesis ; GO:0007476 | inferred from mutant phenotype inferred from genetic interaction with ap <newline> chaeta development ; GO:0022416 | inferred from mutant phenotype <newline> reproductive process ; GO:0022414 | inferred from mutant phenotype <newline> locomotor rhythm ; GO:0045475 | inferred from mutant phenotype <newline> response to cocaine ; GO:0042220 | inferred from mutant phenotype <newline> gastric emptying ; GO:0035483 | inferred from mutant phenotype <newline> imaginal disc-derived leg segmentation ; GO:0036011 | inferred from mutant phenotype	nucleus ; GO:0005634 | non-traceable author statement	protein binding ; GO:0005515 | inferred from physical interaction with pnr inferred from physical interaction with chianti	Hsap\LMO2 <newline> Hsap\LMO3 <newline> Hsap\LMO4 <newline> Hsap\LMO1	X
L4	CR32546	FBgn0052546	CR32546	transfer RNA:Proline-CGG 2-1	tRNA:Pro-CGG-2-1	translation ; GO:0006412 | inferred by curator from GO:0033424	cytosol ; GO:0005829 | inferred by curator from GO:0033424	CCG codon-amino acid adaptor activity ; GO:0033424 | inferred from sequence model	-	X
L4	CG15040	FBgn0030940	CG15040	-	CG15040	-	-	-	Hsap\SBSN	X
L4	CG43289	FBgn0262981	CR43289	long non-coding RNA:CR43289	lncRNA:CR43289	-	-	-	-	X

L4	wgn	FBgn0030941	CG6531	wengen	wgn	apoptotic process ; GO:0006915 | non-traceable author statement <newline> cell surface receptor signaling pathway ; GO:0007166 | inferred from genetic interaction with egr <newline> photoreceptor cell axon guidance ; GO:0072499 | inferred from mutant phenotype <newline> apoptotic process ; GO:0006915 | inferred from direct assay <newline> negative regulation of neuromuscular synaptic transmission ; GO:1900074 | inferred from genetic interaction with Ank2 <newline> neuron cellular homeostasis ; GO:0070050 | inferred from genetic interaction with Ank2	cell surface ; GO:0009986 | inferred from direct assay <newline> integral component of plasma membrane ; GO:0005887 | inferred from sequence or structural similarity with HGNC:11916 inferred from direct assay	protein binding ; GO:0005515 | inferred from physical interaction with UniProtKB:Q9W3I9 <newline> protein binding ; GO:0005515 | inferred from physical interaction with egr <newline> tumor necrosis factor-activated receptor activity ; GO:0005031 | inferred from mutant phenotype <newline> tumor necrosis factor-activated receptor activity ; GO:0005031 | inferred from sequence or structural similarity with HGNC:11916	Hsap\TNFRSF4 <newline> Hsap\NGFR	X
L4	Rip11	FBgn0027335	CG6606	Rab11 interacting protein	Rip11	regulated exocytosis ; GO:0045055 | inferred from biological aspect of ancestor with PANTHER:PTN000405433 <newline> rhabdomere development ; GO:0042052 | inferred from mutant phenotype	intracellular membrane-bounded organelle ; GO:0043231 | inferred from biological aspect of ancestor with PANTHER:PTN000405433	Rab GTPase binding ; GO:0017137 | inferred from biological aspect of ancestor with PANTHER:PTN000405433 <newline> Rab GTPase binding ; GO:0017137 | inferred from physical interaction with Rab11	Hsap\RAB11FIP1 <newline> Hsap\RAB11FIP2 <newline> Hsap\RAB11FIP5	X
L4	CG6540	FBgn0030943	CG6540	Nucleoporin 35kDa	Nup35	nuclear pore organization ; GO:0006999 | inferred from biological aspect of ancestor with PANTHER:PTN000488224 <newline> NLS-bearing protein import into nucleus ; GO:0006607 | inferred from biological aspect of ancestor with PANTHER:PTN000488224 <newline> nuclear pore organization ; GO:0006999 | inferred from sequence or structural similarity with SGD:S000002246	nuclear membrane ; GO:0031965 | inferred from electronic annotation with InterPro:IPR017389 <newline> nuclear pore nuclear basket ; GO:0044615 | inferred from biological aspect of ancestor with PANTHER:PTN000488224 <newline> nuclear pore central transport channel ; GO:0044613 | inferred from sequence or structural similarity with SGD:S000002246 <newline> nuclear pore central transport channel ; GO:0044613 | inferred from biological aspect of ancestor with PANTHER:PTN000488224	structural constituent of nuclear pore ; GO:0017056 | inferred from biological aspect of ancestor with PANTHER:PTN000488224 <newline> phospholipid binding ; GO:0005543 | inferred from biological aspect of ancestor with PANTHER:PTN000488224 <newline> structural constituent of nuclear pore ; GO:0017056 | inferred from sequence or structural similarity with SGD:S000002246 <newline> single-stranded DNA binding ; GO:0003697 | inferred from biological aspect of ancestor with PANTHER:PTN000488224	Hsap\NUP35	X

L4	CG6617	FBgn0030944	CG6617	-	CG6617	biological_process ; GO:0008150 | no biological data available	cellular_component ; GO:0005575 | no biological data available	molecular_function ; GO:0003674 | no biological data available	Hsap\GID8	X
L4	Ing3	FBgn0030945	CG6632	Inhibitor of growth family, member 3	Ing3	histone exchange ; GO:0043486 | inferred from direct assay <newline> histone acetylation ; GO:0016573 | inferred from direct assay	nucleus ; GO:0005634 | inferred from direct assay <newline> histone acetyltransferase complex ; GO:0000123 | inferred from physical interaction with pont <newline> NuA4 histone acetyltransferase complex ; GO:0035267 | inferred from direct assay <newline> NuA4 histone acetyltransferase complex ; GO:0035267 | inferred from biological aspect of ancestor with PANTHER:PTN000817799	histone acetyltransferase activity ; GO:0004402 | contributes_to inferred from biological aspect of ancestor with PANTHER:PTN000817799 <newline> histone acetyltransferase activity ; GO:0004402 | contributes_to inferred from direct assay	Hsap\ING4 <newline> Hsap\ING2 <newline> Hsap\ING5 <newline> Hsap\TAF3 <newline> Hsap\ING3 <newline> Hsap\ING1	X
L4	CG6659	FBgn0030946	CG6659	-	CG6659	protein C-linked glycosylation via 2'-alpha-mannosyl-L-tryptophan ; GO:0018406 | inferred from biological aspect of ancestor with PANTHER:PTN001266774	integral component of membrane ; GO:0016021 | inferred from electronic annotation with InterPro:IPR018732 <newline> nuclear inner membrane ; GO:0005637 | inferred from biological aspect of ancestor with PANTHER:PTN001266774	mannosyltransferase activity ; GO:0000030 | inferred from biological aspect of ancestor with PANTHER:PTN001266774	Hsap\DPY19L3 <newline> Hsap\DPY19L1 <newline> Hsap\DPY19L4 <newline> Hsap\DPY19L2	X

L4	fu	FBgn0001079	CG6551	fused	fu	smoothened signaling pathway ; GO:0007224 | inferred from mutant phenotype <newline> germarium-derived egg chamber formation ; GO:0007293 | inferred from mutant phenotype <newline> regulation of protein stability ; GO:0031647 | inferred from direct assay <newline> positive regulation of protein ubiquitination ; GO:0031398 | inferred from mutant phenotype <newline> segment polarity determination ; GO:0007367 | inferred from mutant phenotype <newline> positive regulation of nucleocytoplasmic transport ; GO:0046824 | inferred from mutant phenotype <newline> smoothened signaling pathway ; GO:0007224 | inferred from direct assay inferred from mutant phenotype <newline> smoothened signaling pathway ; GO:0007224 | inferred from mutant phenotype inferred from genetic interaction with hh <newline> intraciliary transport ; GO:0042073 | inferred from mutant phenotype [illegible]	cytoplasm ; GO:0005737 | inferred from biological aspect of ancestor with PANTHER:PTN000541535 <newline> protein-containing complex ; GO:0032991 | inferred from physical interaction with Su(fu) <newline> membrane ; GO:0016020 | inferred from direct assay <newline> Hedgehog signaling complex ; GO:0035301 | inferred from physical interaction with cos inferred from physical interaction with ci	ATP binding ; GO:0005524 | inferred from electronic annotation with InterPro:IPR000719, InterPro:IPR002290, InterPro:IPR017441 <newline> protein binding ; GO:0005515 | inferred from physical interaction with Sxl <newline> protein serine/threonine kinase activity ; GO:0004674 | inferred from direct assay <newline> protein homodimerization activity ; GO:0042803 | inferred from direct assay <newline> protein binding ; GO:0005515 | inferred from physical interaction with cos	Hsap\STK36 <newline> Hsap\ULK4	X
L4	CG6696	FBgn0030947	CG6696	-	CG6696	proteolysis ; GO:0006508 | inferred from electronic annotation with InterPro:IPR001506, InterPro:IPR006026	-	zinc ion binding ; GO:0008270 | inferred from electronic annotation with InterPro:IPR006026 <newline> metalloendopeptidase activity ; GO:0004222 | inferred from biological aspect of ancestor with PANTHER:PTN000013301	Hsap\ASTL <newline> Hsap\MEP1B <newline> Hsap\BMP1 <newline> Hsap\TLL2 <newline> Hsap\CUBN <newline> Hsap\ADGRG6 <newline> Hsap\MEP1A <newline> Hsap\TNFAIP6 <newline> Hsap\TLL1	X

L4	phm	FBgn0004959	CG6578	phantom	phm	ecdysone biosynthetic process ; GO:0006697 | inferred from direct assay <newline> ecdysone biosynthetic process ; GO:0006697 | inferred from mutant phenotype <newline> xenobiotic metabolic process ; GO:0006805 | inferred from biological aspect of ancestor with PANTHER:PTN000670347 <newline> exogenous drug catabolic process ; GO:0042738 | inferred from biological aspect of ancestor with PANTHER:PTN000670347 <newline> border follicle cell migration ; GO:0007298 | inferred from mutant phenotype <newline> organic acid metabolic process ; GO:0006082 | inferred from biological aspect of ancestor with PANTHER:PTN000670347 <newline> embryonic development via the syncytial blastoderm ; GO:0001700 | inferred from mutant phenotype <newline> oogenesis ; GO:0048477 | inferred from mutant phenotype <newline> oxidation-reduction process ; GO:0055114 | inferred [illegible]	endoplasmic reticulum ; GO:0005783 | inferred from direct assay <newline> intracellular membrane-bounded organelle ; GO:0043231 | inferred from biological aspect of ancestor with PANTHER:PTN000670347 <newline> cytoplasm ; GO:0005737 | inferred from biological aspect of ancestor with PANTHER:PTN000670347	iron ion binding ; GO:0005506 | inferred from electronic annotation with InterPro:IPR001128, InterPro:IPR002401 <newline> heme binding ; GO:0020037 | inferred from biological aspect of ancestor with PANTHER:PTN000670347 <newline> ecdysteroid 25-hydroxylase activity ; GO:0035302 | inferred from direct assay <newline> oxidoreductase activity, acting on paired donors, with incorporation or reduction of molecular oxygen, reduced flavin or flavoprotein as one donor, and incorporation of one atom of oxygen ; GO:0016712 | inferred from biological aspect of ancestor with PANTHER:PTN000670347 <newline> steroid hydroxylase activity ; GO:0008395 | inferred from biological aspect of ancestor with PANTHER:PTN001210788	Hsap\CYP2A7 <newline> Hsap\CYP2F1 <newline> Hsap\CYP2U1 <newline> Hsap\CYP2R1 <newline> Hsap\CYP2S1 <newline> Hsap\CYP2C8 <newline> Hsap\CYP2D6 <newline> Hsap\CYP2J2 <newline> Hsap\CYP2E1 <newline> Hsap\CYP1A1 <newline> Hsap\CYP2W1 <newline> Hsap\CYP2A6 <newline> Hsap\CYP2C19 <newline> Hsap\CYP1A2 <newline> Hsap\CYP2B6 <newline> Hsap\CYP2C18 <newline> Hsap\CYP2A13 <newline> Hsap\CYP2C9 <newline> Hsap\CYP1B1	X

L4	Cyp18a1	FBgn0010383	CG6816	Cytochrome P450-18a1	Cyp18a1	chorion-containing eggshell formation ; GO:0007304 | inferred from mutant phenotype <newline> organic acid metabolic process ; GO:0006082 | inferred from biological aspect of ancestor with PANTHER:PTN000670347 <newline> ecdysteroid catabolic process ; GO:0046344 | inferred from mutant phenotype <newline> oxidation-reduction process ; GO:0055114 | inferred from biological aspect of ancestor with PANTHER:PTN000670347 <newline> imaginal disc-derived leg morphogenesis ; GO:0007480 | inferred from mutant phenotype <newline> prepupal development ; GO:0035210 | inferred from mutant phenotype <newline> pupation ; GO:0035074 | inferred from mutant phenotype <newline> exogenous drug catabolic process ; GO:0042738 | inferred from biological aspect of ancestor with PANTHER:PTN000670347 <newline> metamorphosis ; GO:0007552 | [illegible]	intracellular membrane-bounded organelle ; GO:0043231 | inferred from biological aspect of ancestor with PANTHER:PTN000670347 <newline> cytoplasm ; GO:0005737 | inferred from biological aspect of ancestor with PANTHER:PTN000670347	iron ion binding ; GO:0005506 | inferred from electronic annotation with InterPro:IPR001128, InterPro:IPR002401 <newline> oxidoreductase activity, acting on paired donors, with incorporation or reduction of molecular oxygen, reduced flavin or flavoprotein as one donor, and incorporation of one atom of oxygen ; GO:0016712 | inferred from biological aspect of ancestor with PANTHER:PTN000670347 <newline> steroid hydroxylase activity ; GO:0008395 | inferred from biological aspect of ancestor with PANTHER:PTN001210788 <newline> steroid hydroxylase activity ; GO:0008395 | inferred from direct assay <newline> heme binding ; GO:0020037 | inferred from biological aspect of ancestor with PANTHER:PTN000670347	Hsap\CYP1A1 <newline> Hsap\CYP2C9 <newline> Hsap\CYP2C8 <newline> Hsap\CYP2C18 <newline> Hsap\CYP2F1 <newline> Hsap\CYP2W1 <newline> Hsap\CYP2S1 <newline> Hsap\CYP2J2 <newline> Hsap\CYP2U1 <newline> Hsap\CYP2D6 <newline> Hsap\CYP2E1 <newline> Hsap\CYP2A7 <newline> Hsap\CYP2B6 <newline> Hsap\CYP2C19 <newline> Hsap\CYP1B1 <newline> Hsap\CYP2A13 <newline> Hsap\CYP2A6 <newline> Hsap\CYP2R1 <newline> Hsap\CYP1A2 <newline> Hsap\CYP17A1	X

L4	CCKLR-17D1	FBgn0259231	CG42301	Cholecystokinin-like receptor at 17D1	CCKLR-17D1	G protein-coupled receptor signaling pathway ; GO:0007186 | inferred from sequence or structural similarity <newline> adult locomotory behavior ; GO:0008344 | inferred from mutant phenotype <newline> larval locomotory behavior ; GO:0008345 | inferred from mutant phenotype <newline> positive regulation of cytosolic calcium ion concentration ; GO:0007204 | inferred from direct assay <newline> neuromuscular junction development ; GO:0007528 | inferred from mutant phenotype <newline> G protein-coupled receptor signaling pathway ; GO:0007186 | inferred from sequence model <newline> neuropeptide signaling pathway ; GO:0007218 | inferred from direct assay <newline> multicellular organismal response to stress ; GO:0033555 | inferred from mutant phenotype	integral component of membrane ; GO:0016021 | inferred from sequence model <newline> integral component of plasma membrane ; GO:0005887 | inferred from direct assay <newline> integral component of membrane ; GO:0016021 | inferred from sequence or structural similarity <newline> terminal bouton ; GO:0043195 | colocalizes_with inferred from direct assay <newline> neuronal cell body ; GO:0043025 | inferred from direct assay	G protein-coupled receptor activity ; GO:0004930 | inferred from sequence or structural similarity <newline> gastrin receptor activity ; GO:0015054 | NOT inferred from direct assay <newline> neuropeptide receptor activity ; GO:0008188 | inferred from physical interaction with Dsk <newline> neuropeptide receptor activity ; GO:0008188 | inferred from sequence model <newline> cholecystokinin receptor activity ; GO:0004951 | NOT inferred from direct assay	Hsap\GPR146 <newline> Hsap\CCKBR <newline> Hsap\CCKAR	X
L4	Cyp308a1	FBgn0030949	CG6585	Cyp308a1	Cyp308a1	oxidation-reduction process ; GO:0055114 | inferred from electronic annotation with InterPro:IPR001128, InterPro:IPR002401, InterPro:IPR017972	-	oxidoreductase activity, acting on paired donors, with incorporation or reduction of molecular oxygen ; GO:0016705 | inferred from electronic annotation with InterPro:IPR001128, InterPro:IPR002401, InterPro:IPR017972 <newline> heme binding ; GO:0020037 | inferred from electronic annotation with InterPro:IPR001128, InterPro:IPR002401 <newline> iron ion binding ; GO:0005506 | inferred from electronic annotation with InterPro:IPR001128, InterPro:IPR002401	Hsap\CYP3A7 <newline> Hsap\TBXAS1 <newline> Hsap\CYP3A4 <newline> Hsap\CYP3A43 <newline> Hsap\CYP3A5 <newline> Hsap\CYP3A7-CYP3A51P	X

<table>
<tr><td>L4</td><td>CG42302</td><td>FBgn0259198</td><td>CG42302</td><td>-</td><td>CG42302</td><td>chaperone-mediated protein transport ; GO:0072321 | inferred from electronic annotation with InterPro:IPR039238 <newline> protein insertion into mitochondrial inner membrane ; GO:0045039 | inferred from biological aspect of ancestor with PANTHER:PTN000451188</td><td>mitochondrial intermembrane space protein transporter complex ; GO:0042719 | inferred from biological aspect of ancestor with PANTHER:PTN000451188</td><td>-</td><td>Hsap\TIMM8B <newline> Hsap\TIMM8A <newline> Hsap\TIMM13</td><td>X</td></tr>
<tr><td>L4</td><td>CG6873</td><td>FBgn0030951</td><td>CG6873</td><td>-</td><td>CG6873</td><td>actin filament depolymerization ; GO:0030042 | inferred from electronic annotation with InterPro:IPR017904</td><td>actin cytoskeleton ; GO:0015629 | inferred from electronic annotation with InterPro:IPR017904</td><td>actin binding ; GO:0003779 | inferred from electronic annotation with InterPro:IPR002108 <newline> actin binding ; GO:0003779 | inferred from sequence or structural similarity</td><td>Hsap\DSTN <newline> Hsap\CFL1 <newline> Hsap\CFL2</td><td>X</td></tr>
<tr><td>L4</td><td>CG12609</td><td>FBgn0030952</td><td>CG12609</td><td>-</td><td>CG12609</td><td>regulation of transcription, DNA-templated ; GO:0006355 | inferred from biological aspect of ancestor with PANTHER:PTN001073606</td><td>nucleus ; GO:0005634 | inferred from biological aspect of ancestor with PANTHER:PTN001073606</td><td>DNA-binding transcription factor activity ; GO:0003700 | inferred from biological aspect of ancestor with PANTHER:PTN001073606 <newline> basal RNA polymerase II transcription machinery binding ; GO:0001099 | inferred from biological aspect of ancestor with PANTHER:PTN001073606 <newline> transcription coactivator activity ; GO:0003713 | inferred from biological aspect of ancestor with PANTHER:PTN001073606</td><td>-</td><td>X</td></tr>
<tr><td>L4</td><td>CCKLR-17D3</td><td>FBgn0030954</td><td>CG32540</td><td>Cholecystokinin-like receptor at 17D3</td><td>CCKLR-17D3</td><td>G protein-coupled receptor signaling pathway ; GO:0007186 | inferred from electronic annotation with InterPro:IPR000276, InterPro:IPR000314, InterPro:IPR009126 <newline> G protein-coupled receptor signaling pathway ; GO:0007186 | inferred from sequence or structural similarity <newline> G protein-coupled receptor signaling pathway ; GO:0007186 | inferred from sequence model <newline> adult locomotory behavior ; GO:0008344 | inferred from mutant phenotype</td><td>integral component of membrane ; GO:0016021 | inferred from electronic annotation with InterPro:IPR000276, InterPro:IPR000314, InterPro:IPR009126, InterPro:IPR017452 <newline> integral component of membrane ; GO:0016021 | inferred from sequence model <newline> integral component of membrane ; GO:0016021 | inferred from sequence or structural similarity</td><td>gastrin receptor activity ; GO:0015054 | inferred from electronic annotation with InterPro:IPR000314 <newline> G protein-coupled receptor activity ; GO:0004930 | inferred from sequence or structural similarity <newline> neuropeptide receptor activity ; GO:0008188 | inferred from sequence model</td><td>Hsap\CCKAR <newline> Hsap\CCKBR</td><td>X</td></tr>
</table>

L4	CG6891	FBgn0030955	CG6891	-	CG6891	regulation of actin filament polymerization ; GO:0030833 | inferred from biological aspect of ancestor with PANTHER:PTN000850943	site of polarized growth ; GO:0030427 | inferred from biological aspect of ancestor with PANTHER:PTN000089187 <newline> cortical actin cytoskeleton ; GO:0030864 | inferred from biological aspect of ancestor with PANTHER:PTN000850943 <newline> plasma membrane ; GO:0005886 | inferred from biological aspect of ancestor with PANTHER:PTN000850943 <newline> actin filament ; GO:0005884 | colocalizes_with inferred from biological aspect of ancestor with PANTHER:PTN000850943	actin filament binding ; GO:0051015 | inferred from biological aspect of ancestor with PANTHER:PTN000850943	Hsap\COTL1	X
L4	CG18259	FBgn0030956	CG18259	-	CG18259	-	-	nucleic acid binding ; GO:0003676 | inferred from electronic annotation with InterPro:IPR000504, InterPro:IPR035979	Hsap\POLDIP3 <newline> Hsap\ALYREF	X
L4	CG6900	FBgn0030958	CR6900	-	CR6900	-	-	-	-	X
L4	CG6961	FBgn0030959	CG6961	-	CG6961	-	-	mRNA binding ; GO:0003729 | inferred from sequence or structural similarity <newline> nucleic acid binding ; GO:0003676 | inferred from electronic annotation with InterPro:IPR000504, InterPro:IPR035979	Hsap\POLDIP3 <newline> Hsap\ALYREF	X
L4	CG7001	FBgn0283473	CG7001	S6 Kinase Like	S6KL	protein phosphorylation ; GO:0006468 | inferred from biological aspect of ancestor with PANTHER:PTN000682065 <newline> protein phosphorylation ; GO:0006468 | inferred from direct assay <newline> intracellular signal transduction ; GO:0035556 | inferred from biological aspect of ancestor with PANTHER:PTN000682065	cytoplasm ; GO:0005737 | inferred from direct assay <newline> cytoplasm ; GO:0005737 | inferred from biological aspect of ancestor with PANTHER:PTN000682065 <newline> nucleus ; GO:0005634 | inferred from biological aspect of ancestor with PANTHER:PTN000682065	ATP binding ; GO:0005524 | inferred from electronic annotation with InterPro:IPR000719, InterPro:IPR002290 <newline> protein serine/threonine kinase activity ; GO:0004674 | inferred from direct assay <newline> ribosomal protein S6 kinase activity ; GO:0004711 | inferred from biological aspect of ancestor with PANTHER:PTN001219259 <newline> protein serine/threonine kinase activity ; GO:0004674 | inferred from biological aspect of ancestor with PANTHER:PTN000682065	Hsap\RPS6KB1 <newline> Hsap\PRKCB <newline> Hsap\PRKCG <newline> Hsap\ATG101 <newline> Hsap\PKN2 <newline> Hsap\PRKCH <newline> Hsap\PKN1 <newline> Hsap\RPS6KB2 <newline> Hsap\PRKCQ <newline> Hsap\PRKCA <newline> Hsap\SGK494 <newline> Hsap\PRKCE <newline> Hsap\PRKCD <newline> Hsap\PKN3	X

L4	CG7053	FBgn0030960	CG7053	Autophagy-related 101	Atg101	positive regulation of autophagy ; GO:0010508 | inferred from mutant phenotype <newline> autophagy ; GO:0006914 | inferred from mutant phenotype <newline> autophagosome assembly ; GO:0000045 | inferred from biological aspect of ancestor with PANTHER:PTN000329035 <newline> larval midgut cell programmed cell death ; GO:0035096 | inferred from mutant phenotype <newline> positive regulation of macroautophagy ; GO:0016239 | inferred from mutant phenotype <newline> larval midgut histolysis ; GO:0035069 | inferred from mutant phenotype	phagophore assembly site ; GO:0000407 | inferred from biological aspect of ancestor with PANTHER:PTN000329035 <newline> Atg1/ULK1 kinase complex ; GO:1990316 | inferred from direct assay	protein domain specific binding ; GO:0019904 | inferred from physical interaction with Atg13 <newline> protein kinase binding ; GO:0019901 | inferred from physical interaction with Atg1	Hsap\ATG101 <newline> Hsap\SGK494	X
L4	bnb	FBgn0001090	CG7088	bangles and beads	bnb	multicellular organism development ; GO:0007275 | inferred from mutant phenotype <newline> gliogenesis ; GO:0042063 | inferred from expression pattern	cellular_component ; GO:0005575 | no biological data available	molecular_function ; GO:0003674 | no biological data available	Hsap\SMTNL1	X
L4	CG7058	FBgn0030961	CG7058	-	CG7058	proteasome-mediated ubiquitin-dependent protein catabolic process ; GO:0043161 | inferred from biological aspect of ancestor with PANTHER:PTN000698163 <newline> regulation of proteolysis ; GO:0030162 | inferred from biological aspect of ancestor with PANTHER:PTN000698163 <newline> ubiquitin-dependent protein catabolic process ; GO:0006511 | inferred from biological aspect of ancestor with PANTHER:PTN000698163	cytoplasm ; GO:0005737 | inferred from biological aspect of ancestor with PANTHER:PTN000698163	ubiquitin protein ligase binding ; GO:0031625 | inferred from biological aspect of ancestor with PANTHER:PTN000698163	Hsap\ZBTB2 <newline> Hsap\SPOPL <newline> Hsap\ZNF131 <newline> Hsap\BTBD19 <newline> Hsap\RHOBTB3 <newline> Hsap\KLHL32 <newline> Hsap\LZTR1 <newline> Hsap\BTBD9 <newline> Hsap\BTBD7 <newline> Hsap\ZBTB25 <newline> Hsap\SPOP <newline> Hsap\KBTBD3 <newline> Hsap\KLHL11 <newline> Hsap\KBTBD4	X

L4	CG42450	FBgn0259927	CG42450	-	CG42450	G protein-coupled receptor signaling pathway ; GO:0007186 | inferred from electronic annotation with InterPro:IPR015898 <newline> intracellular signal transduction ; GO:0035556 | inferred from electronic annotation with InterPro:IPR000591	-	-	Hsap\RGS2 <newline> Hsap\AXIN1 <newline> Hsap\RGS17 <newline> Hsap\RGS21 <newline> Hsap\AXIN2 <newline> Hsap\RGS7 <newline> Hsap\RGS1 <newline> Hsap\RGS14 <newline> Hsap\RGS12 <newline> Hsap\RGS4 <newline> Hsap\RGS20 <newline> Hsap\RGS18 <newline> Hsap\RGS19 <newline> Hsap\RGS8 <newline> Hsap\RGS3 <newline> Hsap\RGS5 <newline> Hsap\RGS16 <newline> Hsap\RGS10 <newline> Hsap\RGS9 <newline> Hsap\RGS13 <newline> Hsap\RGS11 <newline> Hsap\RGS6	X
L4	CG7101	FBgn0030963	CG7101	-	CG7101	transcription, DNA-templated ; GO:0006351 | inferred from sequence or structural similarity with UniProtKB:P39933	nucleus ; GO:0005634 | inferred from sequence or structural similarity with UniProtKB:G5EBU4	transcription regulatory region sequence-specific DNA binding ; GO:0000976 | inferred from sequence or structural similarity with UniProtKB:P39933	Hsap\ZBTB26 <newline> Hsap\ZBTB48 <newline> Hsap\ZBTB6	X

L4	Pvf1	FBgn0030964	CG7103	PDGF- and VEGF-related factor 1	Pvf1	vascular endothelial growth factor receptor signaling pathway ; GO:0048010 | inferred from mutant phenotype <newline> negative regulation of cell death ; GO:0060548 | inferred from mutant phenotype <newline> positive regulation of border follicle cell migration ; GO:1903688 | inferred from mutant phenotype <newline> dorsal closure ; GO:0007391 | inferred from mutant phenotype <newline> salivary gland morphogenesis ; GO:0007435 | inferred from mutant phenotype <newline> cell projection assembly ; GO:0030031 | inferred from mutant phenotype <newline> anterior Malpighian tubule development ; GO:0061327 | inferred from mutant phenotype <newline> vascular endothelial growth factor receptor signaling pathway ; GO:0048010 | inferred from direct assay <newline> wound healing, spreading of epidermal cells ; GO:0035313 | inferred from mutant [illegible]	membrane ; GO:0016020 | inferred from electronic annotation with InterPro:IPR000072 <newline> extracellular space ; GO:0005615 | inferred from biological aspect of ancestor with PANTHER:PTN000908679 <newline> extracellular space ; GO:0005615 | inferred from direct assay	growth factor activity ; GO:0008083 | inferred from electronic annotation with InterPro:IPR000072 <newline> vascular endothelial growth factor receptor binding ; GO:0005172 | inferred from sequence or structural similarity with HGNC:12680 <newline> heparin binding ; GO:0008201 | inferred from direct assay <newline> vascular endothelial growth factor receptor binding ; GO:0005172 | inferred from physical interaction with Pvr <newline> receptor ligand activity ; GO:0048018 | inferred from direct assay	Hsap\VEGFD <newline> Hsap\PDGFB <newline> Hsap\PDGFD <newline> Hsap\VEGFA <newline> Hsap\PDGFC <newline> Hsap\VEGFC <newline> Hsap\PGF <newline> Hsap\PDGFA <newline> Hsap\VEGFB	X
L4	CG34422	FBgn0085451	CG34422	hat-trick	htk	regulation of transcription by RNA polymerase II ; GO:0006357 | inferred from biological aspect of ancestor with PANTHER:PTN000359478 <newline> negative regulation of neuron death ; GO:1901215 | inferred from mutant phenotype	nucleus ; GO:0005634 | inferred from biological aspect of ancestor with PANTHER:PTN000359478 <newline> Sin3-type complex ; GO:0070822 | inferred from direct assay <newline> nucleus ; GO:0005634 | inferred from high throughput direct assay	transcription regulatory region DNA binding ; GO:0044212 | inferred from biological aspect of ancestor with PANTHER:PTN000359478	Hsap\ARID5B <newline> Hsap\ARID3B <newline> Hsap\ARID4B <newline> Hsap\ARID4A <newline> Hsap\ARID2 <newline> Hsap\ARID3A <newline> Hsap\ARID5A <newline> Hsap\ARID3C	X

L4	CG7280	FBgn0030966	CG7280	shopper	shop	oxidation-reduction process ; GO:0055114 | inferred from electronic annotation with InterPro:IPR005066, InterPro:IPR008335 <newline> nitrate assimilation ; GO:0042128 | inferred from electronic annotation with InterPro:IPR000572 <newline> sulfur compound metabolic process ; GO:0006790 | inferred from biological aspect of ancestor with PANTHER:PTN000452516 <newline> sulfur compound metabolic process ; GO:0006790 | inferred from sequence or structural similarity with UniProtKB:P51687	mitochondrion ; GO:0005739 | inferred from direct assay <newline> mitochondrion ; GO:0005739 | inferred from biological aspect of ancestor with PANTHER:PTN000452464 <newline> mitochondrial intermembrane space ; GO:0005758 | inferred from sequence or structural similarity with UniProtKB:P51687	molybdenum ion binding ; GO:0030151 | inferred from electronic annotation with InterPro:IPR005066 <newline> sulfite oxidase activity ; GO:0008482 | inferred from direct assay <newline> sulfite oxidase activity ; GO:0008482 | inferred from sequence or structural similarity with UniProtKB:Q07116 <newline> heme binding ; GO:0020037 | inferred from biological aspect of ancestor with PANTHER:PTN000452516 <newline> sulfite oxidase activity ; GO:0008482 | inferred from biological aspect of ancestor with PANTHER:PTN000452516 <newline> molybdopterin cofactor binding ; GO:0043546 | inferred from biological aspect of ancestor with PANTHER:PTN000452516	Hsap\SUOX <newline> Hsap\CYB5R1 <newline> Hsap\CYB5R2 <newline> Hsap\CYB5R3	X
L4	CG7322	FBgn0030968	CG7322	-	CG7322	glucose metabolic process ; GO:0006006 | inferred from biological aspect of ancestor with PANTHER:PTN001211870 <newline> xylulose metabolic process ; GO:0005997 | inferred from biological aspect of ancestor with PANTHER:PTN001211870	-	L-xylulose reductase (NADP+) activity ; GO:0050038 | inferred from biological aspect of ancestor with PANTHER:PTN001211870 <newline> L-xylulose reductase (NADP+) activity ; GO:0050038 | inferred from sequence or structural similarity with UniProtKB:Q7Z4W1 <newline> carbonyl reductase (NADPH) activity ; GO:0004090 | inferred from biological aspect of ancestor with PANTHER:PTN001211870 <newline> oxidoreductase activity, acting on NAD(P)H, quinone or similar compound as acceptor ; GO:0016655 | inferred from sequence or structural similarity with UniProtKB:Q7Z4W1	Hsap\DCXR	X

L4	CG7288	FBgn0030969	CG7288	Ubiquitin specific protease 39	Usp39	spliceosomal complex assembly ; GO:0000245 | inferred from electronic annotation with InterPro:IPR033809 <newline> protein deubiquitination ; GO:0016579 | inferred from electronic annotation with InterPro:IPR001394 <newline> protein deubiquitination ; GO:0016579 | inferred from sequence model <newline> mRNA splicing, via spliceosome ; GO:0000398 | inferred by curator from GO:0071011	precatalytic spliceosome ; GO:0071011 | inferred from high throughput direct assay	zinc ion binding ; GO:0008270 | inferred from electronic annotation with InterPro:IPR001607 <newline> thiol-dependent ubiquitinyl hydrolase activity ; GO:0036459 | inferred from electronic annotation with InterPro:IPR001394 <newline> thiol-dependent ubiquitin-specific protease activity ; GO:0004843 | inferred from sequence model	Hsap\USP39	X
L4	CG32544	FBgn0052544	CG32544	-	CG32544	biological_process ; GO:0008150 | no biological data available	cellular_component ; GO:0005575 | no biological data available	molecular_function ; GO:0003674 | no biological data available	-	X
L4	CG7326	FBgn0030970	CG7326	-	CG7326	-	-	-	Hsap\UBE3D	X
L4	CG34401	FBgn0085430	CG34401	-	CG34401	regulation of axon guidance ; GO:1902667 | inferred from biological aspect of ancestor with PANTHER:PTN000504318	Cul2-RING ubiquitin ligase complex ; GO:0031462 | inferred from biological aspect of ancestor with PANTHER:PTN000504317	zinc ion binding ; GO:0008270 | inferred from electronic annotation with InterPro:IPR007527	Hsap\ZSWIM8 <newline> Hsap\ZSWIM5 <newline> Hsap\ZSWIM6 <newline> Hsap\ZSWIM4	X
L4	CG7332	FBgn0030973	CG7332	-	CG7332	-	-	cysteine-type carboxypeptidase activity ; GO:0016807 | inferred from biological aspect of ancestor with PANTHER:PTN000279908 <newline> Lys48-specific deubiquitinase activity ; GO:1990380 | inferred from biological aspect of ancestor with PANTHER:PTN000279908	Hsap\MINDY4 <newline> Hsap\MINDY3	X
L4	CG7358	FBgn0030974	CG7358	Fl(2)d-associated complex component	Flacc	regulation of alternative mRNA splicing, via spliceosome ; GO:0000381 | inferred from mutant phenotype <newline> sex determination ; GO:0007530 | inferred from mutant phenotype <newline> regulation of alternative mRNA splicing, via spliceosome ; GO:0000381 | inferred from direct assay <newline> mRNA methylation ; GO:0080009 | inferred from mutant phenotype	nucleus ; GO:0005634 | inferred from direct assay <newline> RNA N6-methyladenosine methyltransferase complex ; GO:0036396 | inferred from direct assay	-	Hsap\ZC3H13	X

L4	CG7349	FBgn0030975	CG7349	Succinate dehydrogenase, subunit B (iron-sulfur)-like	SdhBL	tricarboxylic acid cycle ; GO:0006099 | inferred from electronic annotation with InterPro:IPR004489 <newline> respiratory electron transport chain ; GO:0022904 | inferred from biological aspect of ancestor with PANTHER:PTN000229864 <newline> mitochondrial electron transport, succinate to ubiquinone ; GO:0006121 | inferred from sequence or structural similarity with SdhB <newline> aerobic respiration ; GO:0009060 | inferred from biological aspect of ancestor with PANTHER:PTN000229864	mitochondrial membrane ; GO:0031966 | inferred from biological aspect of ancestor with PANTHER:PTN000229867	electron transfer activity ; GO:0009055 | inferred from electronic annotation with InterPro:IPR001041, InterPro:IPR025192 <newline> 2 iron, 2 sulfur cluster binding ; GO:0051537 | inferred from electronic annotation with InterPro:IPR006058 <newline> succinate dehydrogenase (ubiquinone) activity ; GO:0008177 | inferred from sequence or structural similarity with UniProtKB:P21913	Hsap\SDHB	X
L4	CG7378	FBgn0030976	CG7378	-	CG7378	protein dephosphorylation ; GO:0006470 | inferred from electronic annotation with InterPro:IPR000340, InterPro:IPR020405, InterPro:IPR020417, InterPro:IPR020422	cellular_component ; GO:0005575 | no biological data available	protein tyrosine phosphatase activity ; GO:0004725 | inferred from electronic annotation with InterPro:IPR016130 <newline> protein tyrosine/serine/threonine phosphatase activity ; GO:0008138 | inferred from electronic annotation with InterPro:IPR000340, InterPro:IPR020405, InterPro:IPR020417, InterPro:IPR020422	Hsap\DUSP12 <newline> Hsap\DUSP6 <newline> Hsap\DUSP28 <newline> Hsap\DUSP13 <newline> Hsap\DUSP5 <newline> Hsap\DUSP3 <newline> Hsap\DUPD1 <newline> Hsap\DUSP21 <newline> Hsap\DUSP22 <newline> Hsap\DUSP15 <newline> Hsap\DUSP26 <newline> Hsap\DUSP19 <newline> Hsap\DUSP4 <newline> Hsap\DUSP8 <newline> Hsap\DUSP27 <newline> Hsap\DUSP1 <newline> Hsap\DUSP18 <newline> Hsap\DUSP10 <newline> Hsap\STYX <newline> Hsap\SSH2 <newline> Hsap\DUSP2 <newline> Hsap\SSH1 <newline> Hsap\SSH3 <newline> Hsap\DUSP16 <newline> Hsap\DUSP14 <newline> Hsap\STYXL1 <newline> Hsap\DUSP7 <newline> [illegible]	X
L4	CG43759	FBgn0264090	CG43759	-	CG43759	-	-	-	-	X
L4	Diedel3	FBgn0085358	CG34329	Diedel 3	Diedel3	biological_process ; GO:0008150 | no biological data available	extracellular space ; GO:0005615 | inferred from sequence or structural similarity with Diedel	molecular_function ; GO:0003674 | no biological data available	-	X
L4	CG14190	FBgn0030979	CG14190	-	CG14190	-	-	-	-	X
L4	CG34330	FBgn0085359	CG34330	-	CG34330	-	-	-	-	X

L4	CG7406	FBgn0030980	CG7406	-	CG7406	-	-	-	-	X
L4	CG14191	FBgn0030981	CG14191	-	CG14191	-	-	-	Hsap\KRTAP5-5	X
L4	CG7423	FBgn0030982	CG7423	-	CG7423	positive regulation of cell growth ; GO:0030307 | inferred from sequence or structural similarity with RGD:619806	nucleus ; GO:0005634 | inferred from sequence or structural similarity with RGD:619806 <newline> cytosol ; GO:0005829 | inferred from sequence or structural similarity with RGD:619806	sequence-specific DNA binding ; GO:0043565 | inferred from sequence or structural similarity with RGD:619806	Hsap\ASB12 <newline> Hsap\LUZP6 <newline> Hsap\MTPN <newline> Hsap\IQANK1	X
L4	CG15882	FBgn0030983	CG15882	-	CG15882	biological_process ; GO:0008150 | no biological data available	cellular_component ; GO:0005575 | no biological data available	molecular_function ; GO:0003674 | no biological data available	-	X
L4	tgy	FBgn0030984	CG7440	twiggy	tgy	O-glycan processing, core 1 ; GO:0016267 | inferred from biological aspect of ancestor with PANTHER:PTN000547277	membrane ; GO:0016020 | inferred from electronic annotation with InterPro:IPR003378	glycoprotein-N-acetylgalactosamine 3-beta-galactosyltransferase activity ; GO:0016263 | inferred from biological aspect of ancestor with PANTHER:PTN000547277	Hsap\C1GALT1C1 <newline> Hsap\C1GALT1 <newline> Hsap\C1GALT1C1L	X
L4	Obp18a	FBgn0030985	CG15883	Odorant-binding protein 18a	Obp18a	sensory perception of chemical stimulus ; GO:0007606 | inferred from sequence alignment with UniProtKB:P34174 <newline> sensory perception of chemical stimulus ; GO:0007606 | inferred from sequence or structural similarity with UniProtKB:P34174 <newline> sensory perception of chemical stimulus ; GO:0007606 | inferred from sequence or structural similarity with lush	extracellular region ; GO:0005576 | inferred from sequence model	odorant binding ; GO:0005549 | inferred from sequence or structural similarity with UniProtKB:P34174 <newline> odorant binding ; GO:0005549 | inferred from sequence or structural similarity with lush <newline> odorant binding ; GO:0005549 | inferred from sequence alignment with UniProtKB:P34174	-	X
L4	RhoGAP18B	FBgn0261461	CG42274	Rho GTPase activating protein at 18B	RhoGAP18B	signal transduction ; GO:0007165 | inferred from electronic annotation with InterPro:IPR000198, InterPro:IPR008936 <newline> actin cytoskeleton organization ; GO:0030036 | inferred from mutant phenotype <newline> regulation of GTPase activity ; GO:0043087 | inferred from biological aspect of ancestor with PANTHER:PTN000571279 <newline> positive regulation of GTPase activity ; GO:0043547 | inferred from direct assay	cytoplasm ; GO:0005737 | inferred from biological aspect of ancestor with PANTHER:PTN000571279	GTPase activator activity ; GO:0005096 | inferred from direct assay <newline> Rho GTPase binding ; GO:0017048 | inferred from physical interaction with Rac1 inferred from physical interaction with Cdc42 inferred from physical interaction with Rho1	Hsap\RACGAP1 <newline> Hsap\ARHGAP15 <newline> Hsap\ARHGAP12 <newline> Hsap\ARHGAP9 <newline> Hsap\ARHGAP27	X

L4	CG7537	FBgn0030989	CG7537	Innexin 5	Inx5	intercellular transport ; GO:0010496 | inferred from sequence or structural similarity with Inx3	gap junction ; GO:0005921 | inferred from biological aspect of ancestor with PANTHER:PTN000223951 <newline> integral component of membrane ; GO:0016021 | non-traceable author statement <newline> plasma membrane ; GO:0005886 | inferred from biological aspect of ancestor with PANTHER:PTN000223951 <newline> gap junction ; GO:0005921 | inferred from direct assay	gap junction channel activity ; GO:0005243 | inferred from biological aspect of ancestor with PANTHER:PTN000223951 <newline> gap junction channel activity ; GO:0005243 | inferred from sequence or structural similarity with Inx3	-	X
L4	CG7556	FBgn0030990	CG7556	-	CG7556	-	endomembrane system ; GO:0012505 | inferred from high throughput direct assay	DNA binding ; GO:0003677 | inferred from electronic annotation with InterPro:IPR009057	Hsap\DNAJC1	X
L4	CG7453	FBgn0030991	CG7453	-	CG7453	-	-	-	Hsap\C11orf24 <newline> Hsap\MANSC1	X
L4	CG33253	FBgn0030992	CG33253	-	CG33253	-	plasma membrane ; GO:0005886 | inferred from biological aspect of ancestor with PANTHER:PTN000029715	molecular_function ; GO:0003674 | no biological data available	Hsap\STOM <newline> Hsap\STOML3 <newline> Hsap\STOML1 <newline> Hsap\NPHS2 <newline> Hsap\STOML2	X
L4	Mec2	FBgn0030993	CG7635	Mec2	Mec2	nephrocyte filtration ; GO:0097206 | inferred from mutant phenotype	nephrocyte diaphragm ; GO:0005917 | inferred by curator from GO:0005515 <newline> plasma membrane ; GO:0005886 | inferred from biological aspect of ancestor with PANTHER:PTN000029715	protein binding ; GO:0005515 | inferred from physical interaction with sns	Hsap\STOML2 <newline> Hsap\NPHS2 <newline> Hsap\STOML1 <newline> Hsap\STOM <newline> Hsap\STOML3	X
L4	CG14193	FBgn0030994	CG14193	Heterochromatin Protein 1D3 chromoshadow domain	HP1D3csd	chromosome organization ; GO:0051276 | inferred from sequence or structural similarity with Su(var)205	chromatin ; GO:0000785 | inferred from sequence or structural similarity with Su(var)205	chromatin binding ; GO:0003682 | inferred from sequence or structural similarity with Su(var)205	-	X
L4	CG7914	FBgn0030995	CG7914	-	CG7914	oxidation-reduction process ; GO:0055114 | inferred from electronic annotation with InterPro:IPR001834, InterPro:IPR017927, InterPro:IPR017938	-	oxidoreductase activity ; GO:0016491 | inferred from electronic annotation with InterPro:IPR001834, InterPro:IPR017927	Hsap\OXNAD1 <newline> Hsap\CYB5R1 <newline> Hsap\CYB5RL <newline> Hsap\CYB5R4 <newline> Hsap\CYB5R3 <newline> Hsap\CYB5R2	X
L4	CG14194	FBgn0030996	CG14194	-	CG14194	-	-	-	Hsap\TMEM60 <newline> Hsap\TMEM185A <newline> Hsap\TMEM185B	X

L4	CG7990	FBgn0030997	CG7990	-	CG7990	GPI anchor biosynthetic process ; GO:0006506 | inferred from biological aspect of ancestor with PANTHER:PTN000984623	Golgi membrane ; GO:0000139 | inferred from electronic annotation with InterPro:IPR039545 <newline> endoplasmic reticulum membrane ; GO:0005789 | inferred from biological aspect of ancestor with PANTHER:PTN000984623	-	Hsap\PGAP2 <newline> Hsap\CWH43	X
L4	Ulp1	FBgn0027603	CG12359	Ulp1	Ulp1	central nervous system projection neuron axonogenesis ; GO:0021952 | inferred from mutant phenotype <newline> protein desumoylation ; GO:0016926 | inferred from sequence or structural similarity with SGD:S000005941 <newline> protein desumoylation ; GO:0016926 | inferred from biological aspect of ancestor with PANTHER:PTN000288424 <newline> negative regulation of inflammatory response ; GO:0050728 | inferred from mutant phenotype <newline> negative regulation of Toll signaling pathway ; GO:0045751 | inferred from mutant phenotype <newline> cellular response to hypoxia ; GO:0071456 | inferred from mutant phenotype <newline> dendritic spine morphogenesis ; GO:0060997 | inferred from mutant phenotype <newline> proteolysis ; GO:0006508 | inferred from direct assay <newline> protein desumoylation ; GO:0016926 | inferred from mutant phenotype <newline> protein [illegible]	nuclear pore ; GO:0005643 | inferred from direct assay <newline> nuclear inner membrane ; GO:0005637 | inferred from direct assay <newline> nucleus ; GO:0005634 | inferred from direct assay <newline> cytoplasm ; GO:0005737 | inferred from direct assay <newline> nucleus ; GO:0005634 | inferred from biological aspect of ancestor with PANTHER:PTN000288424	SUMO-specific isopeptidase activity ; GO:0070140 | inferred from direct assay <newline> SUMO-specific isopeptidase activity ; GO:0070140 | inferred from sequence or structural similarity with SGD:S000005941	Hsap\SENP5 <newline> Hsap\SENP3 <newline> Hsap\SENP1 <newline> Hsap\SENP2	X
L4	CG14195	FBgn0030998	CG14195	-	CG14195	-	-	-	-	X
L4	Mur18B	FBgn0030999	CG7874	Mucin related 18B	Mur18B	chitin metabolic process ; GO:0006030 | inferred from electronic annotation with InterPro:IPR002557	extracellular region ; GO:0005576 | inferred from electronic annotation with InterPro:IPR002557 <newline> extracellular matrix ; GO:0031012 | inferred from sequence model	chitin binding ; GO:0008061 | inferred from electronic annotation with InterPro:IPR002557 <newline> extracellular matrix structural constituent ; GO:0005201 | inferred from sequence model	-	X

L4	Muc18B	FBgn0031000	CG7876	Mucin 18B	Muc18B	chitin metabolic process ; GO:0006030 | inferred from electronic annotation with InterPro:IPR002557	extracellular region ; GO:0005576 | inferred from electronic annotation with InterPro:IPR002557 <newline> extracellular matrix ; GO:0031012 | inferred from sequence model	chitin binding ; GO:0008061 | inferred from electronic annotation with InterPro:IPR002557 <newline> extracellular matrix structural constituent ; GO:0005201 | inferred from sequence model	Hsap\HHIPL2 <newline> Hsap\C6orf15	X
L4	CG7884	FBgn0031001	CG7884	-	CG7884	biological_process ; GO:0008150 | no biological data available	cellular_component ; GO:0005575 | no biological data available	molecular_function ; GO:0003674 | no biological data available	-	X
L4	CG14196	FBgn0031002	CG14196	-	CG14196	transmembrane transport ; GO:0055085 | inferred from electronic annotation with InterPro:IPR011701 <newline> monocarboxylic acid transport ; GO:0015718 | inferred from biological aspect of ancestor with PANTHER:PTN000144718	integral component of membrane ; GO:0016021 | inferred from electronic annotation with InterPro:IPR011701	monocarboxylic acid transmembrane transporter activity ; GO:0008028 | inferred from biological aspect of ancestor with PANTHER:PTN000888455	Hsap\SLC16A6 <newline> Hsap\SLC16A12 <newline> Hsap\SLC16A13 <newline> Hsap\SLC16A14 <newline> Hsap\SLC16A7 <newline> Hsap\SLC16A5 <newline> Hsap\SLC16A3 <newline> Hsap\SLC16A1 <newline> Hsap\SLC16A11 <newline> Hsap\SLC16A8 <newline> Hsap\SLC16A4 <newline> Hsap\SLC16A9	X
L4	CG7889	FBgn0031003	CG7889	-	CG7889	peptidyl-lysine trimethylation ; GO:0018023 | inferred from sequence or structural similarity with SGD:S000003890	cytoplasm ; GO:0005737 | inferred from sequence or structural similarity with SGD:S000003890	protein-lysine N-methyltransferase activity ; GO:0016279 | inferred from sequence or structural similarity with SGD:S000003890	Hsap\METTL23 <newline> Hsap\METTL21C <newline> Hsap\EEF1AKMT3 <newline> Hsap\EEF2KMT <newline> Hsap\METTL21A <newline> Hsap\METTL22 <newline> Hsap\VCPKMT <newline> Hsap\FAM86B1 <newline> Hsap\FAM86KP <newline> Hsap\FAM86B2 <newline> Hsap\FAM86C1	X
L4	CG7992	FBgn0031004	CG7992	-	CG7992	-	-	-	-	X

L4	Hs3st-B	FBgn0031005	CG7890	Heparan sulfate 3-O sulfotransferase-B	Hs3st-B	chaeta morphogenesis ; GO:0008407 | inferred from mutant phenotype <newline> imaginal disc-derived wing vein specification ; GO:0007474 | inferred from mutant phenotype <newline> imaginal disc-derived wing vein morphogenesis ; GO:0008586 | inferred from mutant phenotype <newline> imaginal disc-derived leg joint morphogenesis ; GO:0016348 | inferred from mutant phenotype <newline> wing disc dorsal/ventral pattern formation ; GO:0048190 | inferred from mutant phenotype <newline> negative regulation of neurogenesis ; GO:0050768 | inferred from mutant phenotype <newline> lysosome organization ; GO:0007040 | inferred from mutant phenotype <newline> imaginal disc-derived wing morphogenesis ; GO:0007476 | inferred from mutant phenotype <newline> endosome organization ; GO:0007032 | inferred from mutant phenotype <newline> compound eye morphogenesis ; GO:0001745 | [illegible]	-	[heparan sulfate]-glucosamine 3-sulfotransferase 1 activity ; GO:0008467 | inferred from biological aspect of ancestor with PANTHER:PTN000834571 <newline> [heparan sulfate]-glucosamine 3-sulfotransferase 1 activity ; GO:0008467 | inferred from sequence or structural similarity with UniProtKB:O14792 inferred from direct assay	Hsap\HS3ST4 <newline> Hsap\HS3ST1 <newline> Hsap\HS3ST5 <newline> Hsap\HS3ST3A1 <newline> Hsap\HS3ST6 <newline> Hsap\HS3ST2 <newline> Hsap\HS3ST3B1	X

L4	rictor	FBgn0031006	CG8002	rapamycin-insensitive companion of Tor	rictor	cellular response to starvation ; GO:0009267 | inferred from direct assay <newline> positive regulation of transcription by transcription factor localization ; GO:0061586 | inferred from physical interaction with UniProtKB:Q9W328 <newline> stress granule assembly ; GO:0034063 | inferred from mutant phenotype <newline> establishment or maintenance of actin cytoskeleton polarity ; GO:0030950 | inferred from biological aspect of ancestor with PANTHER:PTN000329282 <newline> actin cytoskeleton reorganization ; GO:0031532 | inferred from biological aspect of ancestor with PANTHER:PTN000329282 <newline> positive regulation of protein kinase B signaling ; GO:0051897 | inferred from mutant phenotype <newline> regulation of phosphorylation ; GO:0042325 | inferred from biological aspect of ancestor with PANTHER:PTN000329282 <newline> cellular response to insulin stimulus [illegible]	TORC2 complex ; GO:0031932 | inferred from mutant phenotype <newline> TORC2 complex ; GO:0031932 | inferred from biological aspect of ancestor with PANTHER:PTN000329282	enzyme activator activity ; GO:0008047 | inferred from biological aspect of ancestor with PANTHER:PTN000329282	Hsap\RICTOR	X

L4	CG7893	FBgn0040068	CG7893	Vav guanine nucleotide exchange factor	Vav	regulation of Rho protein signal transduction ; GO:0035023 | inferred from electronic annotation with InterPro:IPR000219 <newline> melanotic encapsulation of foreign target ; GO:0035011 | inferred from mutant phenotype <newline> photoreceptor cell axon guidance ; GO:0072499 | inferred from mutant phenotype <newline> activation of GTPase activity ; GO:0090630 | inferred from direct assay <newline> positive regulation of ERK1 and ERK2 cascade ; GO:0070374 | inferred from mutant phenotype <newline> vascular endothelial growth factor receptor signaling pathway ; GO:0048010 | inferred from direct assay <newline> cell migration ; GO:0016477 | inferred from mutant phenotype <newline> epidermal growth factor receptor signaling pathway ; GO:0007173 | inferred from genetic interaction with Egfr <newline> compound eye morphogenesis ; GO:0001745 | [illegible]	cytosol ; GO:0005829 | inferred from direct assay	Rac guanyl-nucleotide exchange factor activity ; GO:0030676 | inferred from direct assay <newline> Ras guanyl-nucleotide exchange factor activity ; GO:0005088 | inferred from genetic interaction with Rac1 <newline> Ras guanyl-nucleotide exchange factor activity ; GO:0005088 | inferred from direct assay	Hsap\PEX13 <newline> Hsap\PLEKHG1 <newline> Hsap\SPATA13 <newline> Hsap\VAV2 <newline> Hsap\STAC3 <newline> Hsap\VAV1 <newline> Hsap\PLEKHG2 <newline> Hsap\STAC <newline> Hsap\VAV3 <newline> Hsap\PLEKHG3 <newline> Hsap\ARHGEF4 <newline> Hsap\PREX1 <newline> Hsap\ARHGEF9 <newline> Hsap\STAC2	X
L4	CG8010	FBgn0031008	CG8010	-	CG8010	-	-	-	-	X
L4	CG32537	FBgn0052537	CG32537	-	CG32537	biological_process ; GO:0008150 | no biological data available	cellular_component ; GO:0005575 | no biological data available	molecular_function ; GO:0003674 | no biological data available	-	X
L4	CG32536	FBgn0052536	CG32536	-	CG32536	-	secretory granule ; GO:0030141 | inferred from biological aspect of ancestor with PANTHER:PTN001032876	-	Hsap\EBAG9	X
L4	CG8028	FBgn0031010	CG8028	-	CG8028	transmembrane transport ; GO:0055085 | inferred from electronic annotation with InterPro:IPR011701 <newline> renal tubular secretion ; GO:0097254 | inferred from expression pattern <newline> monocarboxylic acid transport ; GO:0015718 | inferred from biological aspect of ancestor with PANTHER:PTN000144718 <newline> response to toxic substance ; GO:0009636 | inferred from expression pattern	integral component of membrane ; GO:0016021 | inferred from electronic annotation with InterPro:IPR011701	monocarboxylic acid transmembrane transporter activity ; GO:0008028 | inferred from biological aspect of ancestor with PANTHER:PTN000888455	Hsap\SLC16A12 <newline> Hsap\SLC16A11 <newline> Hsap\SLC16A13 <newline> Hsap\SLC16A8 <newline> Hsap\SLC16A1 <newline> Hsap\SLC16A5 <newline> Hsap\SLC16A4 <newline> Hsap\SLC16A7 <newline> Hsap\SLC16A6 <newline> Hsap\SLC16A9 <newline> Hsap\SLC16A3 <newline> Hsap\SLC16A14	X

L4	CG8034	FBgn0031011	CG8034	-	CG8034	transmembrane transport ; GO:0055085 | inferred from electronic annotation with InterPro:IPR011701 <newline> monocarboxylic acid transport ; GO:0015718 | inferred from biological aspect of ancestor with PANTHER:PTN000144718	integral component of membrane ; GO:0016021 | inferred from electronic annotation with InterPro:IPR011701	monocarboxylic acid transmembrane transporter activity ; GO:0008028 | inferred from biological aspect of ancestor with PANTHER:PTN000888455	Hsap\SLC16A13 <newline> Hsap\SLC16A6 <newline> Hsap\SLC16A8 <newline> Hsap\SLC16A3 <newline> Hsap\SLC16A9 <newline> Hsap\SLC16A4 <newline> Hsap\SLC16A12 <newline> Hsap\SLC16A14 <newline> Hsap\SLC16A1 <newline> Hsap\SLC16A11 <newline> Hsap\SLC16A7 <newline> Hsap\SLC16A5	X
L4	CG8051	FBgn0031012	CG8051	-	CG8051	transmembrane transport ; GO:0055085 | inferred from electronic annotation with InterPro:IPR011701 <newline> monocarboxylic acid transport ; GO:0015718 | inferred from biological aspect of ancestor with PANTHER:PTN000144718	integral component of membrane ; GO:0016021 | inferred from electronic annotation with InterPro:IPR011701	monocarboxylic acid transmembrane transporter activity ; GO:0008028 | inferred from biological aspect of ancestor with PANTHER:PTN000888455	Hsap\SLC16A10 <newline> Hsap\SLC16A8 <newline> Hsap\SLC16A14 <newline> Hsap\SLC16A12 <newline> Hsap\SLC16A9 <newline> Hsap\SLC16A3 <newline> Hsap\SLC16A11 <newline> Hsap\SLC16A13 <newline> Hsap\SLC16A5 <newline> Hsap\SLC16A7 <newline> Hsap\SLC16A1 <newline> Hsap\SLC16A6 <newline> Hsap\SLC16A4	X
L4	out	FBgn0259834	CG8062	outsiders	out	transmembrane transport ; GO:0055085 | inferred from electronic annotation with InterPro:IPR011701 <newline> germ cell development ; GO:0007281 | inferred from mutant phenotype <newline> ectopic germ cell programmed cell death ; GO:0035234 | traceable author statement <newline> monocarboxylic acid transport ; GO:0015718 | inferred from biological aspect of ancestor with PANTHER:PTN000144718	integral component of membrane ; GO:0016021 | inferred from electronic annotation with InterPro:IPR011701	monocarboxylic acid transmembrane transporter activity ; GO:0008028 | inferred from biological aspect of ancestor with PANTHER:PTN000888455	Hsap\SLC16A3 <newline> Hsap\SLC16A5 <newline> Hsap\SLC16A9 <newline> Hsap\SLC16A7 <newline> Hsap\SLC16A11 <newline> Hsap\SLC16A6 <newline> Hsap\SLC16A1 <newline> Hsap\SLC16A12 <newline> Hsap\SLC16A14 <newline> Hsap\SLC16A13 <newline> Hsap\SLC16A8 <newline> Hsap\SLC16A4 <newline> Hsap\SLC16A10	X

L4	CG32538	FBgn0086778	CG32538	nicotinic Acetylcholine Receptor alpha7	nAChRalpha7	chemical synaptic transmission ; GO:0007268 | inferred from mutant phenotype <newline> synaptic transmission, cholinergic ; GO:0007271 | inferred from expression pattern <newline> jump response ; GO:0007630 | inferred from mutant phenotype <newline> excitatory postsynaptic potential ; GO:0060079 | inferred from mutant phenotype <newline> nervous system process ; GO:0050877 | inferred from biological aspect of ancestor with PANTHER:PTN000434994 <newline> cation transport ; GO:0006812 | inferred from direct assay <newline> regulation of membrane potential ; GO:0042391 | inferred from biological aspect of ancestor with PANTHER:PTN000434994 <newline> visual behavior ; GO:0007632 | inferred from mutant phenotype <newline> signal transduction ; GO:0007165 | inferred from biological aspect of ancestor with	postsynaptic membrane ; GO:0045211 | inferred from electronic annotation with InterPro:IPR002394 <newline> acetylcholine-gated channel complex ; GO:0005892 | inferred from sequence or structural similarity <newline> dendrite ; GO:0030425 | inferred from direct assay <newline> integral component of plasma membrane ; GO:0005887 | inferred from biological aspect of ancestor with PANTHER:PTN000434994 <newline> dendritic spine ; GO:0043197 | inferred from direct assay <newline> postsynaptic density ; GO:0014069 | inferred from direct assay <newline> acetylcholine-gated channel complex ; GO:0005892 | inferred from physical interaction with nAChRα5, nAChRα6 <newline> neuron projection ; GO:0043005 | inferred from biological aspect of ancestor with PANTHER:PTN000434994	transmembrane signaling receptor activity ; GO:0004888 | inferred from electronic annotation with InterPro:IPR006201 <newline> acetylcholine-gated cation-selective channel activity ; GO:0022848 | inferred from direct assay <newline> acetylcholine-gated cation-selective channel activity ; GO:0022848 | inferred from sequence or structural similarity <newline> extracellular ligand-gated ion channel activity ; GO:0005230 | contributes_to inferred from biological aspect of ancestor with PANTHER:PTN000434994	Hsap\CHRNA4 <newline> Hsap\CHRNB1 <newline> Hsap\CHRFAM7A <newline> Hsap\HTR3C <newline> Hsap\CHRNA7 <newline> Hsap\CHRNA1 <newline> Hsap\CHRNA2 <newline> Hsap\HTR3D <newline> Hsap\CHRNB3 <newline> Hsap\CHRND <newline> Hsap\CHRNB2 <newline> Hsap\CHRNA10 <newline> Hsap\HTR3B <newline> Hsap\ZACN <newline> Hsap\CHRNE <newline> Hsap\CHRNA5 <newline> Hsap\CHRNA9 <newline> Hsap\CHRNG <newline> Hsap\CHRNB4 <newline> Hsap\CHRNA6 <newline> Hsap\HTR3A <newline> Hsap\CHRNA3 <newline> Hsap\HTR3E	X

L4	kek5	FBgn0031016	CG12199	kekkon 5	kek5	negative regulation of BMP signaling pathway ; GO:0030514 | inferred from mutant phenotype <newline> negative regulation of epidermal growth factor receptor signaling pathway ; GO:0042059 | NOT inferred from mutant phenotype	integral component of plasma membrane ; GO:0005887 | inferred from sequence model <newline> integral component of plasma membrane ; GO:0005887 | inferred from direct assay	-	Hsap\SLITRK1 <newline> Hsap\SLITRK5 <newline> Hsap\LRRC4B <newline> Hsap\SLITRK2 <newline> Hsap\LRFN2 <newline> Hsap\LINGO1 <newline> Hsap\MXRA5 <newline> Hsap\LRRC55 <newline> Hsap\SLITRK6 <newline> Hsap\LRRC26 <newline> Hsap\CHAD <newline> Hsap\IGSF10 <newline> Hsap\ISLR <newline> Hsap\LRRC19 <newline> Hsap\LRTM1 <newline> Hsap\LRIT2 <newline> Hsap\LRRC38 <newline> Hsap\LRRC4 <newline> Hsap\SLITRK4 <newline> Hsap\LINGO2 <newline> Hsap\LRRC4C <newline> Hsap\FLRT2 <newline> Hsap\LRIT1 <newline> Hsap\LRIT3 <newline> Hsap\ISLR2 <newline> Hsap\LRFN1 <newline> Hsap\LRRC70 <newline> [illegible]	X
L4	CG32533	FBgn0052533	CG32533	-	CG32533	nuclear-transcribed mRNA catabolic process, nonsense-mediated decay ; GO:0000184 | inferred from biological aspect of ancestor with PANTHER:PTN002316265	-	RNA binding ; GO:0003723 | inferred from biological aspect of ancestor with PANTHER:PTN000433338 <newline> helicase activity ; GO:0004386 | inferred from sequence or structural similarity with SGD:S000004802	Hsap\DHX34 <newline> Hsap\DHX40 <newline> Hsap\YTHDC2 <newline> Hsap\DHX8 <newline> Hsap\MRPS14 <newline> Hsap\DHX33	X
L4	CG12200	FBgn0031018	CG12200	narya	narya	synapsis ; GO:0007129 | inferred from biological aspect of ancestor with PANTHER:PTN001099470 <newline> positive regulation of meiotic DNA double-strand break formation ; GO:1903343 | inferred from genetic interaction with UniProtKB:O76908 <newline> protein sumoylation ; GO:0016925 | inferred from biological aspect of ancestor with PANTHER:PTN001099470	chromosome ; GO:0005694 | inferred from direct assay <newline> synaptonemal complex ; GO:0000795 | inferred from biological aspect of ancestor with PANTHER:PTN001099470 <newline> site of double-strand break ; GO:0035861 | inferred from direct assay	zinc ion binding ; GO:0008270 | inferred from sequence model <newline> SUMO transferase activity ; GO:0019789 | inferred from biological aspect of ancestor with PANTHER:PTN001099470	Hsap\RNF212B <newline> Hsap\RNF212	X

L5	RunxA	FBgn0083981	CG34145	Runt related A	RunxA	regulation of transcription, DNA-templated ; GO:0006355 | inferred from electronic annotation with InterPro:IPR000040, InterPro:IPR008967, InterPro:IPR012346, InterPro:IPR013524 <newline> dendrite morphogenesis ; GO:0048813 | inferred from mutant phenotype	nucleus ; GO:0005634 | inferred from electronic annotation with InterPro:IPR000040, InterPro:IPR012346	DNA-binding transcription factor activity ; GO:0003700 | inferred from electronic annotation with InterPro:IPR008967, InterPro:IPR012346, InterPro:IPR013524 <newline> ATP binding ; GO:0005524 | inferred from electronic annotation with InterPro:IPR000040 <newline> DNA binding ; GO:0003677 | inferred from electronic annotation with InterPro:IPR000040, InterPro:IPR012346, InterPro:IPR013524	Hsap\RUNX1 <newline> Hsap\RUNX3 <newline> Hsap\RUNX2	X
L5	Cyp6v1	FBgn0031126	CG1829	Cyp6v1	Cyp6v1	oxidation-reduction process ; GO:0055114 | inferred from electronic annotation with InterPro:IPR001128, InterPro:IPR002401, InterPro:IPR017972 <newline> response to DDT ; GO:0046680 | inferred from biological aspect of ancestor with PANTHER:PTN001958763 <newline> insecticide catabolic process ; GO:0046701 | inferred from biological aspect of ancestor with PANTHER:PTN001958763	-	oxidoreductase activity, acting on paired donors, with incorporation or reduction of molecular oxygen ; GO:0016705 | inferred from electronic annotation with InterPro:IPR001128, InterPro:IPR002401, InterPro:IPR017972 <newline> heme binding ; GO:0020037 | inferred from electronic annotation with InterPro:IPR001128, InterPro:IPR002401 <newline> iron ion binding ; GO:0005506 | inferred from electronic annotation with InterPro:IPR001128, InterPro:IPR002401	Hsap\TBXAS1 <newline> Hsap\CYP3A4 <newline> Hsap\CYP3A43 <newline> Hsap\CYP3A7-CYP3A51P <newline> Hsap\CYP3A7 <newline> Hsap\CYP3A5	X
L5	CG1835	FBgn0031127	CG1835	-	CG1835	-	-	-	-	X
L5	hydra	FBgn0031128	CG1338	hydra	hydra	biological_process ; GO:0008150 | no biological data available	cellular_component ; GO:0005575 | no biological data available	molecular_function ; GO:0003674 | no biological data available	-	X

L5	run	FBgn0003300	CG1849	runt	run	neuroblast fate determination ; GO:0007400 | traceable author statement <newline> eye morphogenesis ; GO:0048592 | inferred from mutant phenotype <newline> germ-band extension ; GO:0007377 | inferred from mutant phenotype <newline> positive regulation of transcription, DNA-templated ; GO:0045893 | inferred from direct assay <newline> positive regulation of transcription, DNA-templated ; GO:0045893 | inferred from mutant phenotype <newline> axon guidance ; GO:0007411 | inferred from mutant phenotype <newline> periodic partitioning by pair rule gene ; GO:0007366 | non-traceable author statement <newline> germ-band extension ; GO:0007377 | traceable author statement <newline> sex determination, establishment of X:A ratio ; GO:0007540 | traceable author statement <newline> sex determination, establishment of X:A ratio ; GO:0007540 | non-traceable [illegible]	nucleus ; GO:0005634 | inferred from direct assay	ATP binding ; GO:0005524 | inferred from electronic annotation with InterPro:IPR000040 <newline> DNA-binding transcription factor activity ; GO:0003700 | inferred from direct assay <newline> sequence-specific DNA binding ; GO:0043565 | inferred from direct assay	Hsap\RUNX2 <newline> Hsap\RUNX1 <newline> Hsap\RUNX3	X
L5	CG1324	FBgn0031129	CG1324	-	CG1324	-	-	-	-	X
L5	CG15452	FBgn0031130	CG15452	-	CG15452	-	-	-	-	X

L5	shakB	FBgn0085387	CG34358	shaking B	shakB	transmembrane transport ; GO:0055085 | inferred from mutant phenotype <newline> intercellular transport ; GO:0010496 | inferred from sequence or structural similarity with Inx3 <newline> jump response ; GO:0007630 | inferred from mutant phenotype <newline> gap junction assembly ; GO:0016264 | inferred from mutant phenotype <newline> phototransduction ; GO:0007602 | inferred from mutant phenotype <newline> regulation of membrane depolarization ; GO:0003254 | inferred from mutant phenotype <newline> cell communication by electrical coupling ; GO:0010644 | inferred from mutant phenotype <newline> response to light stimulus ; GO:0009416 | inferred from mutant phenotype	gap junction ; GO:0005921 | inferred from biological aspect of ancestor with PANTHER:PTN000223951 <newline> plasma membrane ; GO:0005886 | inferred from direct assay <newline> gap junction ; GO:0005921 | inferred from mutant phenotype <newline> plasma membrane ; GO:0005886 | inferred from biological aspect of ancestor with PANTHER:PTN000223951 <newline> gap junction ; GO:0005921 | inferred from direct assay	gap junction channel activity ; GO:0005243 | inferred from mutant phenotype <newline> gap junction channel activity ; GO:0005243 | inferred from biological aspect of ancestor with PANTHER:PTN000223951 <newline> photoreceptor activity ; GO:0009881 | inferred from mutant phenotype	-	X
L5	CG32507	FBgn0052507	CG32507	-	CG32507	biological_process ; GO:0008150 | no biological data available	cellular_component ; GO:0005575 | no biological data available	molecular_function ; GO:0003674 | no biological data available	Hsap\ARMCX5-GPRASP2 <newline> Hsap\GPRASP1 <newline> Hsap\GPRASP2	X
L5	CG15450	FBgn0031132	CG15450	-	CG15450	-	-	transferase activity, transferring acyl groups ; GO:0016746 | inferred from electronic annotation with InterPro:IPR002123	Hsap\GPAT4 <newline> Hsap\AUP1 <newline> Hsap\GPAT3	X
L5	CG43193	FBgn0262822	CR43193	long non-coding RNA:CR43193	lncRNA:CR43193	biological_process ; GO:0008150 | no biological data available	cellular_component ; GO:0005575 | no biological data available	molecular_function ; GO:0003674 | no biological data available	-	X
L5	CG1314	FBgn0031134	CG1314	-	CG1314	-	-	-	-	X

L5	I-3	FBgn0261624	CG42707	Inhibitor-3	I-3	negative regulation of phosphoprotein phosphatase activity ; GO:0032515 | inferred from biological aspect of ancestor with PANTHER:PTN000464544 <newline> ubiquitin-dependent protein catabolic process ; GO:0006511 | inferred from sequence or structural similarity with UniProtKB:O60927 <newline> negative regulation of protein dephosphorylation ; GO:0035308 | inferred from biological aspect of ancestor with PANTHER:PTN000464544	protein phosphatase type 1 complex ; GO:0000164 | colocalizes_with inferred from biological aspect of ancestor with PANTHER:PTN000464544 <newline> nucleus ; GO:0005634 | inferred from biological aspect of ancestor with PANTHER:PTN000464544	protein serine/threonine phosphatase inhibitor activity ; GO:0004865 | inferred from biological aspect of ancestor with PANTHER:PTN000464544 <newline> ubiquitin protein ligase activity ; GO:0061630 | inferred from sequence or structural similarity with UniProtKB:O60927 <newline> protein phosphatase 1 binding ; GO:0008157 | inferred from biological aspect of ancestor with PANTHER:PTN000464544 <newline> protein phosphatase 1 binding ; GO:0008157 | inferred from physical interaction with flw inferred from physical interaction with Pp1-13C inferred from physical interaction with Pp1α-96A inferred from physical interaction with Pp1-87B	Hsap\PPP1R11 <newline> Hsap\HLA-DPA1	X
L5	CG11227	FBgn0031139	CG11227	-	CG11227	-	-	-	-	X
L5	Npc1b	FBgn0261675	CG12092	Niemann-Pick type C-1b	Npc1b	central nervous system development ; GO:0007417 | inferred from mutant phenotype <newline> peripheral nervous system development ; GO:0007422 | inferred from mutant phenotype <newline> intestinal cholesterol absorption ; GO:0030299 | inferred from mutant phenotype <newline> dorsal closure ; GO:0007391 | inferred from mutant phenotype	integral component of membrane ; GO:0016021 | inferred from electronic annotation with InterPro:IPR003392	-	Hsap\NPC1 <newline> Hsap\NPC1L1 <newline> Hsap\SCAP	X
L5	Ser6	FBgn0011834	CG2071	Serine protease 6	Ser6	proteolysis ; GO:0006508 | inferred from electronic annotation with InterPro:IPR001254, InterPro:IPR001314, InterPro:IPR018114 <newline> proteolysis ; GO:0006508 | inferred from sequence model	-	serine-type endopeptidase activity ; GO:0004252 | inferred from sequence model <newline> serine-type endopeptidase activity ; GO:0004252 | inferred from biological aspect of ancestor with PANTHER:PTN001208729	Hsap\TPSD1 <newline> Hsap\KLK14 <newline> Hsap\GZMA <newline> Hsap\PRSS53 <newline> Hsap\CFD <newline> Hsap\PRSS36	X

L5	CG1304	FBgn0031141	CG1304	-	CG1304	proteolysis ; GO:0006508 | inferred from electronic annotation with InterPro:IPR001254, InterPro:IPR001314, InterPro:IPR018114 <newline> proteolysis ; GO:0006508 | inferred from sequence model	-	serine-type endopeptidase activity ; GO:0004252 | inferred from biological aspect of ancestor with PANTHER:PTN001208729 <newline> serine-type endopeptidase activity ; GO:0004252 | inferred from sequence model	Hsap\CFD <newline> Hsap\KLK14 <newline> Hsap\GZMA <newline> Hsap\TPSD1 <newline> Hsap\PRSS53 <newline> Hsap\PRSS36	X
L5	CG11666	FBgn0040648	CG11666	-	CG11666	biological_process ; GO:0008150 | no biological data available	cellular_component ; GO:0005575 | no biological data available	molecular_function ; GO:0003674 | no biological data available	-	X
L5	r-cup	FBgn0031142	CG10998	ryder cup	r-cup	-	-	-	Hsap\TTC25	X
L5	CG1532	FBgn0031143	CG1532	-	CG1532	-	-	-	Hsap\GLOD4	X
L5	CG1529	FBgn0031144	CG1529	-	CG1529	negative regulation of transcription by RNA polymerase II ; GO:0000122 | inferred from sequence or structural similarity with UniProtKB:Q8K2R5	nucleus ; GO:0005634 | inferred from sequence or structural similarity with UniProtKB:G5EDU4	zinc ion binding ; GO:0008270 | inferred from electronic annotation with InterPro:IPR012934 <newline> RNA polymerase II proximal promoter sequence-specific DNA binding ; GO:0000978 | inferred from sequence or structural similarity with UniProtKB:Q8K2R5 <newline> DNA-binding transcription repressor activity, RNA polymerase II-specific ; GO:0001227 | inferred from sequence or structural similarity with UniProtKB:Q8K2R5	Hsap\ZNF560 <newline> Hsap\ZNF142 <newline> Hsap\ZNF697	X
L5	Ntf-2	FBgn0031145	CG1740	Nuclear transport factor-2	Ntf-2	protein import into nucleus ; GO:0006606 | inferred from biological aspect of ancestor with PANTHER:PTN000289032 <newline> positive regulation of antimicrobial peptide biosynthetic process ; GO:0002807 | inferred from mutant phenotype <newline> nucleocytoplasmic transport ; GO:0006913 | inferred from biological aspect of ancestor with PANTHER:PTN000289031	nuclear pore central transport channel ; GO:0044613 | inferred from biological aspect of ancestor with PANTHER:PTN000289031	-	Hsap\NUTF2	X
L5	CG15449	FBgn0031146	CG15449	-	CG15449	septate junction assembly ; GO:0019991 | inferred from electronic annotation with InterPro:IPR038976	integral component of plasma membrane ; GO:0005887 | inferred from biological aspect of ancestor with PANTHER:PTN002195845	-	-	X

L5	Mgstl	FBgn0025814	CG1742	Microsomal glutathione S-transferase-like	Mgstl	oxidation-reduction process ; GO:0055114 | inferred from biological aspect of ancestor with PANTHER:PTN000841525 <newline> prostaglandin biosynthetic process ; GO:0001516 | inferred from biological aspect of ancestor with PANTHER:PTN000074809	mitochondrial outer membrane ; GO:0005741 | inferred from sequence or structural similarity with UniProtKB:P10620 <newline> intracellular membrane-bounded organelle ; GO:0043231 | inferred from sequence or structural similarity with HGNC:7061 <newline> intracellular membrane-bounded organelle ; GO:0043231 | inferred from sequence or structural similarity with UniProtKB:P10620	glutathione peroxidase activity ; GO:0004602 | inferred from sequence or structural similarity with UniProtKB:P10620 <newline> prostaglandin-E synthase activity ; GO:0050220 | inferred from biological aspect of ancestor with PANTHER:PTN000074809 <newline> glutathione transferase activity ; GO:0004364 | inferred from sequence or structural similarity with HGNC:7061	Hsap\MGST1 <newline> Hsap\PTGES	X
L5	Cbs	FBgn0031148	CG1753	Cystathionine beta-synthase	Cbs	cysteine biosynthetic process via cystathionine ; GO:0019343 | inferred from electronic annotation with InterPro:IPR005857 <newline> cysteine biosynthetic process from serine ; GO:0006535 | inferred from biological aspect of ancestor with PANTHER:PTN000034466 <newline> hydrogen sulfide biosynthetic process ; GO:0070814 | inferred from biological aspect of ancestor with PANTHER:PTN000034107 <newline> transsulfuration ; GO:0019346 | inferred from biological aspect of ancestor with PANTHER:PTN000034107 <newline> homocysteine metabolic process ; GO:0050667 | inferred from sequence or structural similarity with UniProtKB:P35520 <newline> response to endoplasmic reticulum stress ; GO:0034976 | inferred from mutant phenotype <newline> homocysteine metabolic process ; GO:0050667 | inferred from biological aspect of ancestor with PANTHER:PTN000034107 <newline> [illegible]	cytoplasm ; GO:0005737 | inferred from biological aspect of ancestor with PANTHER:PTN000034104	cystathionine beta-synthase activity ; GO:0004122 | inferred from electronic annotation with InterPro:IPR005857 <newline> pyridoxal phosphate binding ; GO:0030170 | inferred from biological aspect of ancestor with PANTHER:PTN000034104 <newline> nitrite reductase (NO-forming) activity ; GO:0050421 | inferred from sequence or structural similarity with UniProtKB:P35520 <newline> cysteine synthase activity ; GO:0004124 | inferred from biological aspect of ancestor with PANTHER:PTN000034466	Hsap\CBS <newline> Hsap\CBSL	X

L5	CG1518	FBgn0031149	CG1518	Catalytic subunit 3A of the oligosaccharyltransferase complex	Stt3A	post-translational protein modification ; GO:0043687 | inferred from biological aspect of ancestor with PANTHER:PTN000356208 <newline> protein N-linked glycosylation via asparagine ; GO:0018279 | inferred from biological aspect of ancestor with PANTHER:PTN000356208	membrane ; GO:0016020 | inferred from electronic annotation with InterPro:IPR003674 <newline> endomembrane system ; GO:0012505 | inferred from high throughput direct assay	dolichyl-diphosphooligosaccharide-protein glycotransferase activity ; GO:0004579 | inferred from biological aspect of ancestor with PANTHER:PTN000356208	Hsap\STT3B <newline> Hsap\STT3A	X
L5	bves	FBgn0031150	CG32513	bves	bves	-	sarcolemma ; GO:0042383 | inferred from biological aspect of ancestor with PANTHER:PTN001383178 <newline> membrane ; GO:0016020 | inferred from biological aspect of ancestor with PANTHER:PTN001383178	cAMP binding ; GO:0030552 | inferred from biological aspect of ancestor with PANTHER:PTN001383178	Hsap\POPDC3 <newline> Hsap\POPDC2 <newline> Hsap\BVES	X
L5	CG32512	FBgn0052512	CG32512	-	CG32512	biological_process ; GO:0008150 | no biological data available	cellular_component ; GO:0005575 | no biological data available	molecular_function ; GO:0003674 | no biological data available	Hsap\TMEM205	X
L5	stg1	FBgn0064123	CG33670	stargazin-like protein	stg1	regulation of synaptic transmission, glutamatergic ; GO:0051966 | inferred from direct assay <newline> regulation of AMPA receptor activity ; GO:2000311 | inferred from direct assay	integral component of membrane ; GO:0016021 | inferred from electronic annotation with InterPro:IPR004031 <newline> integral component of membrane ; GO:0016021 | inferred from sequence model	channel regulator activity ; GO:0016247 | inferred from direct assay	Hsap\CACNG7 <newline> Hsap\TMEM235 <newline> Hsap\CACNG4 <newline> Hsap\CACNG3 <newline> Hsap\CACNG8 <newline> Hsap\CACNG5 <newline> Hsap\CACNG2	X
L5	CG11566	FBgn0031159	CG11566	-	CG11566	biological_process ; GO:0008150 | no biological data available	cellular_component ; GO:0005575 | no biological data available	molecular_function ; GO:0003674 | no biological data available	Hsap\CACNG5 <newline> Hsap\CACNG4 <newline> Hsap\CACNG3 <newline> Hsap\CACNG7 <newline> Hsap\CACNG2 <newline> Hsap\CACNG8	X
L5	CG15446	FBgn0031155	CG15446	-	CG15446	-	-	nucleic acid binding ; GO:0003676 | inferred from electronic annotation with InterPro:IPR013087	-	X
L5	CG1503	FBgn0031157	CG1503	-	CG1503	proteolysis ; GO:0006508 | inferred from electronic annotation with InterPro:IPR003653	-	cysteine-type peptidase activity ; GO:0008234 | inferred from electronic annotation with InterPro:IPR003653	Hsap\SENP8	X

L5	unc	FBgn0003950	CG1501	uncoordinated	unc	non-motile cilium assembly ; GO:1905515 | inferred from mutant phenotype <newline> sensory perception of sound ; GO:0007605 | inferred from mutant phenotype <newline> locomotory behavior ; GO:0007626 | inferred from mutant phenotype <newline> spermatogenesis ; GO:0007283 | inferred from mutant phenotype	ciliary basal body ; GO:0036064 | inferred from direct assay <newline> ciliary transition zone ; GO:0035869 | inferred from direct assay <newline> ciliary cap ; GO:0061822 | inferred from direct assay <newline> centriole ; GO:0005814 | inferred from direct assay <newline> ring centriole ; GO:0061823 | inferred from direct assay	-	-	X
L5	CG15445	FBgn0031161	CG15445	-	CG15445	-	-	-	Hsap\NUB1	X
L5	CG34120	FBgn0083956	CG34120	-	CG34120	transmembrane transport ; GO:0055085 | inferred from electronic annotation with InterPro:IPR026082 <newline> lipid transport ; GO:0006869 | inferred from biological aspect of ancestor with PANTHER:PTN000442469	integral component of membrane ; GO:0016021 | inferred from sequence or structural similarity with UniProtKB:Q9BZC7 <newline> intracellular membrane-bounded organelle ; GO:0043231 | inferred from biological aspect of ancestor with PANTHER:PTN000442469	ATP binding ; GO:0005524 | inferred from electronic annotation with InterPro:IPR003439 <newline> ATPase activity ; GO:0016887 | inferred from electronic annotation with InterPro:IPR003439 <newline> lipid transporter activity ; GO:0005319 | inferred from biological aspect of ancestor with PANTHER:PTN000442469 <newline> ATPase-coupled transmembrane transporter activity ; GO:0042626 | inferred from biological aspect of ancestor with PANTHER:PTN000442469 <newline> ATPase-coupled transmembrane transporter activity ; GO:0042626 | inferred from sequence or structural similarity with UniProtKB:O94911	Hsap\ABCA4 <newline> Hsap\ABCA7 <newline> Hsap\ABCA10 <newline> Hsap\ABCA9 <newline> Hsap\ABCA2 <newline> Hsap\ABCA1 <newline> Hsap\ABCA5 <newline> Hsap\ABCA6 <newline> Hsap\ABCA8 <newline> Hsap\ABCA12 <newline> Hsap\ABCA3 <newline> Hsap\ABCA13	X
L5	CG14579	FBgn0031163	CG14579	-	CG14579	-	-	-	-	X

L5	CG1724	FBgn0031164	CG1724	-	CG1724	protein import into mitochondrial matrix ; GO:0030150 | inferred from biological aspect of ancestor with PANTHER:PTN000050380 <newline> protein targeting to mitochondrion ; GO:0006626 | inferred from sequence or structural similarity with UniProtKB:O60830	TIM23 mitochondrial import inner membrane translocase complex ; GO:0005744 | inferred from sequence or structural similarity with UniProtKB:O60830 <newline> TIM23 mitochondrial import inner membrane translocase complex ; GO:0005744 | inferred from biological aspect of ancestor with PANTHER:PTN000050380 <newline> integral component of mitochondrial inner membrane ; GO:0031305 | inferred from biological aspect of ancestor with PANTHER:PTN000050380	protein transmembrane transporter activity ; GO:0008320 | contributes_to inferred from biological aspect of ancestor with PANTHER:PTN000050380 <newline> P-P-bond-hydrolysis-driven protein transmembrane transporter activity ; GO:0015450 | inferred from sequence or structural similarity with UniProtKB:O60830	Hsap\TIMM17B <newline> Hsap\TIMM17A	X
L5	CG32521	FBgn0052521	CG32521	-	CG32521	-	-	-	-	X
L5	CG32523	FBgn0052523	CG32523	-	CG32523	proteolysis ; GO:0006508 | inferred from electronic annotation with InterPro:IPR001254, InterPro:IPR001314, InterPro:IPR018114 <newline> proteolysis ; GO:0006508 | inferred from sequence model	-	serine-type endopeptidase activity ; GO:0004252 | inferred from sequence model <newline> serine-type endopeptidase activity ; GO:0004252 | inferred from biological aspect of ancestor with PANTHER:PTN001208729	Hsap\CFD <newline> Hsap\PRSS36 <newline> Hsap\PRSS53 <newline> Hsap\TPSD1	X
L5	CR32526	FBgn0011954	CR32526	transfer RNA:Arginine-TCG 2-2	tRNA:Arg-TCG-2-2	translation ; GO:0006412 | inferred by curator from GO:0033431	cytosol ; GO:0005829 | inferred by curator from GO:0033431	CGA codon-amino acid adaptor activity ; GO:0033431 | inferred from sequence model	-	X
L5	CG1722	FBgn0031168	CG1722	-	CG1722	-	-	-	-	X

L5	CG1494	FBgn0031169	CG1494	-	CG1494	transmembrane transport ; GO:0055085 | inferred from electronic annotation with InterPro:IPR026082 <newline> lipid transport ; GO:0006869 | inferred from biological aspect of ancestor with PANTHER:PTN000442469	integral component of membrane ; GO:0016021 | inferred from sequence or structural similarity with UniProtKB:Q9BZC7 <newline> intracellular membrane-bounded organelle ; GO:0043231 | inferred from biological aspect of ancestor with PANTHER:PTN000442469	ATP binding ; GO:0005524 | inferred from electronic annotation with InterPro:IPR003439 <newline> ATPase activity ; GO:0016887 | inferred from electronic annotation with InterPro:IPR003439 <newline> lipid transporter activity ; GO:0005319 | inferred from biological aspect of ancestor with PANTHER:PTN000442469 <newline> ATPase-coupled transmembrane transporter activity ; GO:0042626 | inferred from biological aspect of ancestor with PANTHER:PTN000442469 <newline> ATPase-coupled transmembrane transporter activity ; GO:0042626 | inferred from sequence or structural similarity with UniProtKB:O94911	Hsap\ABCA9 <newline> Hsap\ABCA13 <newline> Hsap\ABCA1 <newline> Hsap\ABCA3 <newline> Hsap\ABCA6 <newline> Hsap\ABCA7 <newline> Hsap\ABCA2 <newline> Hsap\ABCA8 <newline> Hsap\ABCA12 <newline> Hsap\ABCA10 <newline> Hsap\ABCA5 <newline> Hsap\ABCA4	X
L5	CG1718	FBgn0031170	CG1718	ATP binding cassette subfamily A	ABCA	transmembrane transport ; GO:0055085 | inferred from electronic annotation with InterPro:IPR026082 <newline> lipid transport ; GO:0006869 | inferred from biological aspect of ancestor with PANTHER:PTN000442469	integral component of membrane ; GO:0016021 | inferred from sequence or structural similarity with UniProtKB:Q9BZC7 <newline> intracellular membrane-bounded organelle ; GO:0043231 | inferred from biological aspect of ancestor with PANTHER:PTN000442469	ATP binding ; GO:0005524 | inferred from electronic annotation with InterPro:IPR003439, InterPro:IPR017871 <newline> ATPase activity ; GO:0016887 | inferred from electronic annotation with InterPro:IPR003439, InterPro:IPR017871 <newline> ATPase-coupled transmembrane transporter activity ; GO:0042626 | inferred from biological aspect of ancestor with PANTHER:PTN000442469 <newline> lipid transporter activity ; GO:0005319 | inferred from biological aspect of ancestor with PANTHER:PTN000442469 <newline> ATPase-coupled transmembrane transporter activity ; GO:0042626 | inferred from sequence or structural similarity with UniProtKB:O94911	Hsap\ABCA12 <newline> Hsap\ABCA1 <newline> Hsap\ABCA7 <newline> Hsap\ABCA4 <newline> Hsap\ABCA13 <newline> Hsap\ABCA10 <newline> Hsap\ABCA5 <newline> Hsap\ABCA2 <newline> Hsap\ABCA8 <newline> Hsap\ABCA9 <newline> Hsap\ABCA3 <newline> Hsap\ABCA6	X

L5	CR32520	FBgn0052520	CR32520	transfer RNA:Tyrosine-GTA 1-1	tRNA:Tyr-GTA-1-1	translation ; GO:0006412 | inferred by curator from GO:0033410	cytosol ; GO:0005829 | inferred by curator from GO:0033410	UAC codon-amino acid adaptor activity ; GO:0033410 | inferred from sequence model	-	X
L5	CR32525	FBgn0052525	CR32525	transfer RNA:Tyrosine-GTA 2-1	tRNA:Tyr-GTA-2-1	translation ; GO:0006412 | inferred by curator from GO:0033410	cytosol ; GO:0005829 | inferred by curator from GO:0033410	UAC codon-amino acid adaptor activity ; GO:0033410 | inferred from sequence model	-	X
L5	CG1801	FBgn0031171	CG1801	-	CG1801	transmembrane transport ; GO:0055085 | inferred from electronic annotation with InterPro:IPR026082 <newline> lipid transport ; GO:0006869 | inferred from biological aspect of ancestor with PANTHER:PTN000442469	integral component of membrane ; GO:0016021 | inferred from sequence or structural similarity with UniProtKB:Q9BZC7 <newline> intracellular membrane-bounded organelle ; GO:0043231 | inferred from biological aspect of ancestor with PANTHER:PTN000442469	ATP binding ; GO:0005524 | inferred from electronic annotation with InterPro:IPR003439 <newline> ATPase activity ; GO:0016887 | inferred from electronic annotation with InterPro:IPR003439 <newline> lipid transporter activity ; GO:0005319 | inferred from biological aspect of ancestor with PANTHER:PTN000442469 <newline> ATPase-coupled transmembrane transporter activity ; GO:0042626 | inferred from biological aspect of ancestor with PANTHER:PTN000442469 <newline> ATPase-coupled transmembrane transporter activity ; GO:0042626 | inferred from sequence or structural similarity with UniProtKB:O94911	Hsap\ABCA5 <newline> Hsap\ABCA10 <newline> Hsap\ABCA7 <newline> Hsap\ABCA3 <newline> Hsap\ABCA8 <newline> Hsap\ABCA12 <newline> Hsap\ABCA6 <newline> Hsap\ABCA4 <newline> Hsap\ABCA1 <newline> Hsap\ABCA13 <newline> Hsap\ABCA2 <newline> Hsap\ABCA9	X

L5	CG1489	FBgn0020369	CG1489	Regulatory particle triple-A ATPase 6	Rpt6	proteasome-mediated ubiquitin-dependent protein catabolic process ; GO:0043161 | inferred from mutant phenotype <newline> positive regulation of RNA polymerase II transcriptional preinitiation complex assembly ; GO:0045899 | inferred from biological aspect of ancestor with PANTHER:PTN000553334 <newline> proteasome-mediated ubiquitin-dependent protein catabolic process ; GO:0043161 | inferred by curator from GO:0005838 <newline> modulation of chemical synaptic transmission ; GO:0050804 | inferred from biological aspect of ancestor with PANTHER:PTN000553337	proteasome regulatory particle, base subcomplex ; GO:0008540 | inferred from sequence or structural similarity with SGD:S000003016 <newline> proteasome regulatory particle ; GO:0005838 | inferred from sequence or structural similarity with HGNC:9552 inferred from direct assay <newline> nucleus ; GO:0005634 | inferred from direct assay <newline> cytoplasm ; GO:0005737 | inferred from direct assay <newline> nuclear proteasome complex ; GO:0031595 | inferred from biological aspect of ancestor with PANTHER:PTN000553334 <newline> proteasome regulatory particle ; GO:0005838 | inferred from direct assay <newline> proteasome regulatory particle, base subcomplex ; GO:0008540 | inferred from biological aspect of ancestor with PANTHER:PTN000553334 <newline> cytosolic proteasome [illegible]	ATP binding ; GO:0005524 | inferred from electronic annotation with InterPro:IPR003959, InterPro:IPR003960 <newline> ATPase activity ; GO:0016887 | inferred from sequence model <newline> TBP-class protein binding ; GO:0017025 | inferred from biological aspect of ancestor with PANTHER:PTN000553334 <newline> hydrolase activity ; GO:0016787 | inferred from electronic annotation with InterPro:IPR005937	Hsap\PSMC6 <newline> Hsap\PSMC2 <newline> Hsap\PSMC5 <newline> Hsap\WDR12	X
L5	CG33713	FBgn0064116	CG33713	anorexia	anox	-	-	fatty-acyl-CoA binding ; GO:0000062 | inferred from electronic annotation with InterPro:IPR000582	Hsap\ACBD4 <newline> Hsap\DBI <newline> Hsap\ECI2 <newline> Hsap\ACBD5 <newline> Hsap\METTL23 <newline> Hsap\ACBD6 <newline> Hsap\ACBD7	X

L5	CG33714	FBgn0064117	CG33714	SRA stem-loop interacting RNA binding protein 1	SLIRP1	mRNA processing ; GO:0006397 | inferred from biological aspect of ancestor with PANTHER:PTN002690849	cytosol ; GO:0005829 | inferred from biological aspect of ancestor with PANTHER:PTN002544150 <newline> ribonucleoprotein complex ; GO:1990904 | inferred from biological aspect of ancestor with PANTHER:PTN002689974 <newline> nucleus ; GO:0005634 | inferred from biological aspect of ancestor with PANTHER:PTN002309896	RNA binding ; GO:0003723 | inferred from biological aspect of ancestor with PANTHER:PTN002689974 <newline> mRNA binding ; GO:0003729 | inferred from sequence or structural similarity <newline> mRNA binding ; GO:0003729 | inferred from biological aspect of ancestor with PANTHER:PTN002690849	Hsap\HNRNPA1L2 <newline> Hsap\EIF4B <newline> Hsap\RBM3 <newline> Hsap\DAZ4 <newline> Hsap\DAZ2 <newline> Hsap\HNRNPA3 <newline> Hsap\DAZ3 <newline> Hsap\SLIRP <newline> Hsap\CIRBP <newline> Hsap\DAZ1 <newline> Hsap\HNRNPD <newline> Hsap\SRSF10 <newline> Hsap\HNRNPC <newline> Hsap\HNRNPA1 <newline> Hsap\EIF4H <newline> Hsap\RALY <newline> Hsap\HNRNPCL1 <newline> Hsap\RALYL <newline> Hsap\TRNAU1AP <newline> Hsap\RBM34 <newline> Hsap\BOLL <newline> Hsap\HNRNPAB <newline> Hsap\HNRNPA2B1 <newline> Hsap\DAZL	X

L5	Dd	FBgn0029067	CG1696	Dullard	Dd	negative regulation of BMP signaling pathway ; GO:0030514 | inferred from genetic interaction with tkv <newline> protein dephosphorylation ; GO:0006470 | inferred from sequence or structural similarity with HGNC:19085 <newline> protein dephosphorylation ; GO:0006470 | inferred from mutant phenotype <newline> protein dephosphorylation ; GO:0006470 | inferred from sequence or structural similarity with UniProtKB:O95476 <newline> imaginal disc-derived wing vein specification ; GO:0007474 | inferred from mutant phenotype <newline> positive regulation of triglyceride biosynthetic process ; GO:0010867 | inferred from sequence or structural similarity with UniProtKB:O95476 <newline> negative regulation of BMP signaling pathway ; GO:0030514 | inferred from mutant phenotype <newline> nuclear envelope organization ; [illegible]	cytoplasm ; GO:0005737 | inferred from sequence or structural similarity with UniProtKB:O95476 <newline> nuclear envelope ; GO:0005635 | inferred from sequence or structural similarity with UniProtKB:O95476 <newline> endoplasmic reticulum membrane ; GO:0005789 | inferred from sequence or structural similarity with UniProtKB:O95476 <newline> Nem1-Spo7 phosphatase complex ; GO:0071595 | inferred from sequence or structural similarity with UniProtKB:O95476 <newline> Nem1-Spo7 phosphatase complex ; GO:0071595 | inferred from sequence or structural similarity with HGNC:19085 <newline> nuclear membrane ; GO:0031965 | inferred from sequence or structural similarity with UniProtKB:O95476 <newline> cytoplasm ; GO:0005737 | inferred from sequence or structural similarity with HGNC:19085	protein serine/threonine phosphatase activity ; GO:0004722 | inferred from sequence or structural similarity with UniProtKB:O95476 <newline> protein serine/threonine phosphatase activity ; GO:0004722 | inferred from sequence or structural similarity with HGNC:19085 <newline> phosphoprotein phosphatase activity ; GO:0004721 | inferred from biological aspect of ancestor with PANTHER:PTN000258719 <newline> protein serine/threonine phosphatase activity ; GO:0004722 | inferred from mutant phenotype	Hsap\CTDSP2 <newline> Hsap\CTDNEP1 <newline> Hsap\UBLCP1 <newline> Hsap\CTDSPL2 <newline> Hsap\CTDSP1 <newline> Hsap\CTDSPL	X
L5	CG1486	FBgn0031174	CG1486	-	CG1486	carboxylic acid metabolic process ; GO:0019752 | inferred from electronic annotation with InterPro:IPR002129 <newline> sphingolipid metabolic process ; GO:0006665 | inferred from biological aspect of ancestor with PANTHER:PTN000242650 <newline> sphingolipid catabolic process ; GO:0030149 | inferred from biological aspect of ancestor with PANTHER:PTN000242650 <newline> ameboidal-type cell migration ; GO:0001667 | inferred from biological aspect of ancestor with PANTHER:PTN000242655	endoplasmic reticulum ; GO:0005783 | inferred from biological aspect of ancestor with PANTHER:PTN000242650	carboxy-lyase activity ; GO:0016831 | inferred from electronic annotation with InterPro:IPR002129 <newline> pyridoxal phosphate binding ; GO:0030170 | inferred from electronic annotation with InterPro:IPR002129 <newline> sphinganine-1-phosphate aldolase activity ; GO:0008117 | inferred from biological aspect of ancestor with PANTHER:PTN000242650	Hsap\PDXDC1 <newline> Hsap\LOC102724985	X

L5	tty	FBgn0015558	CG1693	tweety	tty	chloride transport ; GO:0006821 | inferred from sequence or structural similarity with HGNC:22222	integral component of membrane ; GO:0016021 | inferred from sequence model <newline> plasma membrane ; GO:0005886 | inferred from sequence or structural similarity with UniProtKB:Q9C0H2 <newline> plasma membrane ; GO:0005886 | inferred from biological aspect of ancestor with PANTHER:PTN000965383	intracellular calcium activated chloride channel activity ; GO:0005229 | inferred from biological aspect of ancestor with PANTHER:PTN000965383 <newline> chloride channel activity ; GO:0005254 | inferred from sequence or structural similarity with UniProtKB:Q9C0H2 <newline> volume-sensitive chloride channel activity ; GO:0072320 | inferred from biological aspect of ancestor with PANTHER:PTN000965383 <newline> chloride channel activity ; GO:0005254 | inferred from sequence or structural similarity with HGNC:22222	Hsap\TTYH3 <newline> Hsap\TTYH1 <newline> Hsap\TTYH2	X
L5	fliI	FBgn0000709	CG1484	flightless I	fliI	actin filament severing ; GO:0051014 | inferred from electronic annotation with InterPro:IPR029919 <newline> adult somatic muscle development ; GO:0007527 | inferred from mutant phenotype <newline> myofibril assembly ; GO:0030239 | inferred from mutant phenotype <newline> gastrulation involving germ band extension ; GO:0010004 | inferred from mutant phenotype	-	actin filament binding ; GO:0051015 | inferred from electronic annotation with InterPro:IPR007122 <newline> calcium ion binding ; GO:0005509 | inferred from sequence or structural similarity with HGNC:12690 inferred from sequence or structural similarity with HGNC:4620 <newline> actin binding ; GO:0003779 | inferred from sequence or structural similarity with Gel inferred from sequence or structural similarity with HGNC:12690	Hsap\FLII	X

L5	dod	FBgn0015379	CG17051	dodo	dod	regulation of locomotor rhythm ; GO:1904059 | inferred from mutant phenotype <newline> positive regulation of protein ubiquitination ; GO:0031398 | inferred from direct assay	nucleus ; GO:0005634 | inferred from biological aspect of ancestor with PANTHER:PTN000069608 <newline> nucleus ; GO:0005634 | inferred from direct assay <newline> cytosol ; GO:0005829 | inferred from biological aspect of ancestor with PANTHER:PTN000069608	peptidyl-prolyl cis-trans isomerase activity ; GO:0003755 | inferred from biological aspect of ancestor with PANTHER:PTN000069608 <newline> transcription factor binding ; GO:0008134 | inferred from physical interaction with Cf2 <newline> peptidyl-prolyl cis-trans isomerase activity ; GO:0003755 | inferred from sequence or structural similarity with HGNC:8988 inferred from sequence or structural similarity with SGD:S000003778	Hsap\PIN1	X
L5	CG1685	FBgn0015527	CG1685	penguin	peng	apposition of dorsal and ventral imaginal disc-derived wing surfaces ; GO:0007475 | inferred from mutant phenotype	nucleolus ; GO:0005730 | inferred from biological aspect of ancestor with PANTHER:PTN000333308	RNA binding ; GO:0003723 | inferred from electronic annotation with InterPro:IPR001313, InterPro:IPR012959, InterPro:IPR033133	Hsap\PUM3	X
L5	sol	FBgn0003464	CG1391	small optic lobes	sol	proteolysis ; GO:0006508 | inferred from biological aspect of ancestor with PANTHER:PTN000021568 <newline> nervous system development ; GO:0007399 | inferred from mutant phenotype <newline> proteolysis ; GO:0006508 | inferred from sequence or structural similarity with UniProtKB:Q11002	cytoplasm ; GO:0005737 | inferred from biological aspect of ancestor with PANTHER:PTN000021568 <newline> cytoplasm ; GO:0005737 | non-traceable author statement	calcium-dependent cysteine-type endopeptidase activity ; GO:0004198 | inferred from biological aspect of ancestor with PANTHER:PTN000021568 <newline> calcium-dependent cysteine-type endopeptidase activity ; GO:0004198 | inferred from sequence or structural similarity with UniProtKB:Q11002	Hsap\CAPN9 <newline> Hsap\CAPN14 <newline> Hsap\CAPN10 <newline> Hsap\CAPN3 <newline> Hsap\CAPN6 <newline> Hsap\ADGB <newline> Hsap\CAPN15 <newline> Hsap\CAPN13 <newline> Hsap\CAPN2 <newline> Hsap\CAPN5 <newline> Hsap\CAPN12 <newline> Hsap\CAPN1 <newline> Hsap\CAPN8 <newline> Hsap\CAPN11 <newline> Hsap\CAPN7	X

B) Genes within the H-intervals

Interval	Gene	FBID_KEY	ANNOTATION_SYMBOL	NAME	SYMBOL	GO_BIOLOGICAL_PROCESS	GO_CELLULAR_COMPONENT	GO_MOLECULAR_FUNCTION	H_SAPIENS_ORTHOLOGS	LOCATION_ARM
H1	CG32773	FBgn0052773	CR32773	long non-coding RNA:CR32773	lncRNA:CR32773	biological_process ; GO:0008150 | no biological data available	cellular_component ; GO:0005575 | no biological data available	molecular_function ; GO:0003674 | no biological data available	-	X
H1	CG12684	FBgn0029717	CG12684	-	CG12684	-	-	-	Hsap\CDIP1 <newline> Hsap\LITAF	X

H1	peb	FBgn0003053	CG12212	pebbled	peb	amnioserosa maintenance ; GO:0046665 | traceable author statement <newline> maintenance of epithelial integrity, open tracheal system ; GO:0035160 | inferred from mutant phenotype <newline> establishment of ommatidial planar polarity ; GO:0042067 | inferred from mutant phenotype <newline> germ-band shortening ; GO:0007390 | traceable author statement <newline> photoreceptor cell axon guidance ; GO:0072499 | inferred from mutant phenotype <newline> photoreceptor cell morphogenesis ; GO:0008594 | inferred from mutant phenotype <newline> germ-band shortening ; GO:0007390 | inferred from mutant phenotype <newline> ectoderm development ; GO:0007398 | traceable author statement <newline> negative regulation of protein localization to plasma membrane ; GO:1903077 | inferred from mutant phenotype <newline> negative regulation of JNK cascade ; [illegible]	nucleus ; GO:0005634 | inferred from biological aspect of ancestor with PANTHER:PTN002509549 <newline> nucleus ; GO:0005634 | inferred from direct assay <newline> polytene chromosome ; GO:0005700 | inferred from direct assay	RNA polymerase II regulatory region sequence-specific DNA binding ; GO:0000977 | inferred from biological aspect of ancestor with PANTHER:PTN002509549 <newline> RNA polymerase II proximal promoter sequence-specific DNA binding ; GO:0000978 | inferred from biological aspect of ancestor with PANTHER:PTN002509549 <newline> DNA-binding transcription activator activity, RNA polymerase II-specific ; GO:0001228 | inferred from biological aspect of ancestor with PANTHER:PTN002509549 <newline> DNA-binding transcription factor activity ; GO:0003700 | inferred from biological aspect of ancestor with PANTHER:PTN002509549	Hsap\RREB1 <newline> Hsap\ZNF821	X
H1	CG12184	FBgn0025387	CG12184	-	CG12184	-	-	-	-	X
H1	CG12179	FBgn0025388	CG12179	-	CG12179	biological_process ; GO:0008150 | no biological data available	cellular_component ; GO:0005575 | no biological data available	molecular_function ; GO:0003674 | no biological data available	-	X
H1	pon	FBgn0025739	CG3346	partner of numb	pon	embryonic heart tube development ; GO:0035050 | inferred from genetic interaction with jumu inferred from genetic interaction with CHES-1-like <newline> asymmetric neuroblast division ; GO:0055059 | traceable author statement	cytoplasm ; GO:0005737 | inferred from direct assay <newline> basal part of cell ; GO:0045178 | traceable author statement <newline> cell cortex ; GO:0005938 | inferred from direct assay <newline> basal cortex ; GO:0045180 | non-traceable author statement	-	-	X
H1	CG3081	FBgn0025613	CG3081	-	CG3081	biological_process ; GO:0008150 | no biological data available	cellular_component ; GO:0005575 | no biological data available	molecular_function ; GO:0003674 | no biological data available	-	X
H1	CG3062	FBgn0025612	CG3062	-	CG3062	cilium organization ; GO:0044782 | inferred from biological aspect of ancestor with PANTHER:PTN002161271	ciliary basal body ; GO:0036064 | inferred from biological aspect of ancestor with PANTHER:PTN002161271	molecular_function ; GO:0003674 | no biological data available	Hsap\CFAP126	X

H1	HLH4C	FBgn0011277	CG3052	Helix loop helix protein 4C	HLH4C	regulation of transcription by RNA polymerase II ; GO:0006357 | inferred from biological aspect of ancestor with PANTHER:PTN001015390	nucleus ; GO:0005634 | inferred from biological aspect of ancestor with PANTHER:PTN001015390	protein dimerization activity ; GO:0046983 | inferred from electronic annotation with InterPro:IPR011598 <newline> DNA-binding transcription factor activity, RNA polymerase II-specific ; GO:0000981 | inferred from biological aspect of ancestor with PANTHER:PTN001015390 <newline> RNA polymerase II regulatory region sequence-specific DNA binding ; GO:0000977 | inferred from biological aspect of ancestor with PANTHER:PTN001015390	Hsap\TAL2 <newline> Hsap\ASCL4 <newline> Hsap\HAND2 <newline> Hsap\SCX <newline> Hsap\MESP2 <newline> Hsap\NHLH1 <newline> Hsap\FIGLA <newline> Hsap\ASCL3 <newline> Hsap\MSC <newline> Hsap\TCF21 <newline> Hsap\PTF1A <newline> Hsap\TCF23 <newline> Hsap\TCF24 <newline> Hsap\TCF15 <newline> Hsap\ASCL1 <newline> Hsap\MSGN1 <newline> Hsap\MESP1 <newline> Hsap\NHLH2 <newline> Hsap\ASCL5 <newline> Hsap\HAND1 <newline> Hsap\LYL1 <newline> Hsap\TAL1	X
H1	mRpL30	FBgn0029718	CG7038	mitochondrial ribosomal protein L30	mRpL30	mitochondrial translation ; GO:0032543 | traceable author statement <newline> translation ; GO:0006412 | inferred from sequence or structural similarity with EMBL:AA772463	mitochondrion ; GO:0005739 | inferred from biological aspect of ancestor with PANTHER:PTN001439403 <newline> mitochondrial large ribosomal subunit ; GO:0005762 | inferred from sequence or structural similarity with EMBL:AA772463 <newline> mitochondrial large ribosomal subunit ; GO:0005762 | traceable author statement	structural constituent of ribosome ; GO:0003735 | traceable author statement <newline> structural constituent of ribosome ; GO:0003735 | inferred from sequence or structural similarity with EMBL:AA772463	Hsap\C2orf15 <newline> Hsap\MRPL30	X

H1	Torsin	FBgn0025615	CG3024	Torsin	Torsin	chaperone cofactor-dependent protein refolding ; GO:0051085 | inferred from electronic annotation with InterPro:IPR017378 <newline> positive regulation of GTP cyclohydrolase I activity ; GO:0043104 | inferred from mutant phenotype <newline> negative regulation of phosphatidate phosphatase activity ; GO:1903741 | inferred from mutant phenotype <newline> membrane lipid biosynthetic process ; GO:0046467 | inferred from mutant phenotype <newline> larval fat body development ; GO:0007504 | inferred from mutant phenotype <newline> ribonucleoprotein complex export from nucleus ; GO:0071426 | inferred from mutant phenotype <newline> regulation of dopamine metabolic process ; GO:0042053 | inferred from mutant phenotype <newline> triglyceride homeostasis ; GO:0070328 | inferred from mutant phenotype <newline> positive regulation of cell [illegible]	nuclear envelope ; GO:0005635 | inferred from direct assay <newline> endoplasmic reticulum ; GO:0005783 | inferred from direct assay	ATP binding ; GO:0005524 | inferred from electronic annotation with InterPro:IPR010448, InterPro:IPR017378 <newline> ATPase activity ; GO:0016887 | inferred from biological aspect of ancestor with PANTHER:PTN000844597 <newline> ATPase activity ; GO:0016887 | inferred from sequence model <newline> ATP binding ; GO:0005524 | inferred from sequence model	Hsap\TOR2A <newline> Hsap\TOR3A <newline> Hsap\TOR1B <newline> Hsap\TOR1A <newline> Hsap\TOR4A	X

H1	Cbp80	FBgn0022942	CG7035	cap binding protein 80	Cbp80	mRNA cis splicing, via spliceosome ; GO:0045292 | inferred from electronic annotation with InterPro:IPR027159 <newline> negative regulation of viral genome replication ; GO:0045071 | inferred from mutant phenotype <newline> production of siRNA involved in RNA interference ; GO:0030422 | inferred from mutant phenotype <newline> mRNA splicing, via spliceosome ; GO:0000398 | inferred by curator from GO:0071011,GO:0071013 <newline> nuclear-transcribed mRNA catabolic process, nonsense-mediated decay ; GO:0000184 | inferred from biological aspect of ancestor with PANTHER:PTN000275634 <newline> RNA interference ; GO:0016246 | inferred from mutant phenotype <newline> mRNA splicing, via spliceosome ; GO:0000398 | inferred from sequence or structural similarity with SGD:S000004732 <newline> gene [illegible]	nucleus ; GO:0005634 | inferred from biological aspect of ancestor with PANTHER:PTN000275634 <newline> mRNA cap binding complex ; GO:0005845 | inferred from biological aspect of ancestor with PANTHER:PTN000275634 <newline> precatalytic spliceosome ; GO:0071011 | inferred from high throughput direct assay <newline> catalytic step 2 spliceosome ; GO:0071013 | inferred from high throughput direct assay <newline> nuclear cap binding complex ; GO:0005846 | inferred from biological aspect of ancestor with PANTHER:PTN000275634	RNA cap binding ; GO:0000339 | inferred from biological aspect of ancestor with PANTHER:PTN000275634 <newline> mRNA binding ; GO:0003729 | inferred from biological aspect of ancestor with PANTHER:PTN000275634	Hsap\NCBP1	X
H1	CG15473	FBgn0029719	CG15473	-	CG15473	-	-	-	-	X
H1	CG3009	FBgn0029720	CG3009	-	CG3009	arachidonic acid secretion ; GO:0050482 | inferred from electronic annotation with InterPro:IPR016090 <newline> phospholipid metabolic process ; GO:0006644 | inferred from electronic annotation with InterPro:IPR016090	-	calcium-dependent phospholipase A2 activity ; GO:0047498 | inferred from biological aspect of ancestor with PANTHER:PTN000956767	Hsap\PROCA1 <newline> Hsap\PLA2G3	X

H1	fzr	FBgn0262699	CG3000	fizzy-related	fzr	positive regulation of ubiquitin protein ligase activity ; GO:1904668 | inferred from electronic annotation with InterPro:IPR033010 <newline> glial cell migration ; GO:0008347 | inferred from mutant phenotype <newline> compound eye morphogenesis ; GO:0001745 | inferred from mutant phenotype <newline> protein catabolic process ; GO:0030163 | inferred from mutant phenotype <newline> protein catabolic process ; GO:0030163 | inferred from direct assay <newline> positive regulation of exit from mitosis ; GO:0031536 | inferred from mutant phenotype <newline> eye-antennal disc morphogenesis ; GO:0007455 | inferred from mutant phenotype <newline> exit from mitosis ; GO:0010458 | NOT inferred from mutant phenotype <newline> glial cell differentiation ; GO:0010001 | inferred from direct assay <newline> anaphase-promoting complex-dependent catabolic [illegible]	cytoplasm ; GO:0005737 | inferred from direct assay <newline> centriole ; GO:0005814 | inferred from direct assay <newline> axon ; GO:0030424 | inferred from direct assay <newline> spindle ; GO:0005819 | inferred from direct assay <newline> centrosome ; GO:0005813 | inferred from direct assay <newline> anaphase-promoting complex ; GO:0005680 | inferred from direct assay	anaphase-promoting complex binding ; GO:0010997 | inferred from electronic annotation with InterPro:IPR033010 <newline> ubiquitin-protein transferase activator activity ; GO:0097027 | inferred from electronic annotation with InterPro:IPR033010 <newline> ubiquitin protein ligase activity ; GO:0061630 | inferred from direct assay	Hsap\CDC20B <newline> Hsap\FZR1 <newline> Hsap\CDC20	X

H1	Pp2C1	FBgn0022768	CG2984	Protein phosphatase 2C	Pp2C1	oxidation-reduction process ; GO:0055114 | inferred from electronic annotation with InterPro:IPR003952 <newline> protein dephosphorylation ; GO:0006470 | inferred from sequence or structural similarity with SGD:S000000329 <newline> protein dephosphorylation ; GO:0006470 | inferred from sequence or structural similarity	cytoplasm ; GO:0005737 | inferred from sequence or structural similarity with SGD:S000000329	cation binding ; GO:0043169 | inferred from electronic annotation with InterPro:IPR000222 <newline> oxidoreductase activity ; GO:0016491 | inferred from electronic annotation with InterPro:IPR003952 <newline> protein serine/threonine phosphatase activity ; GO:0004722 | inferred from sequence or structural similarity <newline> magnesium-dependent protein serine/threonine phosphatase activity ; GO:0004724 | inferred from biological aspect of ancestor with PANTHER:PTN000352745 <newline> mitogen-activated protein kinase binding ; GO:0051019 | inferred from biological aspect of ancestor with PANTHER:PTN001014085 <newline> protein serine/threonine phosphatase activity ; GO:0004722 | inferred from sequence or structural similarity with SGD:S000000329 [illegible]	Hsap\ILKAP <newline> Hsap\TAB1 <newline> Hsap\PPM1F <newline> Hsap\PPM1E <newline> Hsap\PP2D1 <newline> Hsap\PPM1D <newline> Hsap\PPM1K <newline> Hsap\PPM1L	X

H1	ctp	FBgn0011760	CG6998	cut up	ctp	sperm individualization ; GO:0007291 | inferred from mutant phenotype <newline> establishment of mitotic spindle orientation ; GO:0000132 | inferred from mutant phenotype <newline> spermatid nucleus elongation ; GO:0007290 | inferred from mutant phenotype <newline> microtubule anchoring at centrosome ; GO:0034454 | inferred from mutant phenotype <newline> imaginal disc-derived wing morphogenesis ; GO:0007476 | inferred from mutant phenotype <newline> spermatogenesis ; GO:0007283 | inferred from mutant phenotype <newline> chaeta development ; GO:0022416 | inferred from mutant phenotype <newline> positive regulation of ATP-dependent microtubule motor activity, plus-end-directed ; GO:2000582 | inferred from biological aspect of ancestor with PANTHER:PTN000223361 <newline> wing disc development ; GO:0035220 | inferred from mutant [illegible]	cytoplasm ; GO:0005737 | inferred from direct assay <newline> cytoplasmic dynein complex ; GO:0005868 | inferred from direct assay <newline> centriole ; GO:0005814 | inferred from direct assay <newline> dynein complex ; GO:0030286 | inferred from biological aspect of ancestor with PANTHER:PTN000223361 <newline> dynein complex ; GO:0030286 | inferred from direct assay	dynein light intermediate chain binding ; GO:0051959 | inferred from direct assay <newline> protein binding ; GO:0005515 | inferred from physical interaction with UniProtKB:Q9V9Y9 <newline> protein homodimerization activity ; GO:0042803 | inferred from direct assay <newline> ATP-dependent microtubule motor activity, plus-end-directed ; GO:0008574 | contributes_to inferred from biological aspect of ancestor with PANTHER:PTN000223361 <newline> dynein light intermediate chain binding ; GO:0051959 | inferred from biological aspect of ancestor with PANTHER:PTN000223361 <newline> dynein intermediate chain binding ; GO:0045505 | inferred from physical interaction with sw <newline> dynein intermediate chain binding ; GO:0045505 | inferred from biological aspect of ancestor with	Hsap\DNAL4 <newline> Hsap\DYNLL2 <newline> Hsap\DYNLL1	X
H1	Pdha	FBgn0028325	CG7010	Pyruvate dehydrogenase E1 alpha subunit	Pdha	acetyl-CoA biosynthetic process from pyruvate ; GO:0006086 | inferred from electronic annotation with InterPro:IPR017597 <newline> pyruvate metabolic process ; GO:0006090 | inferred from sequence or structural similarity with UniProtKB:P29803	mitochondrion ; GO:0005739 | inferred from high throughput direct assay <newline> pyruvate dehydrogenase complex ; GO:0045254 | inferred from sequence or structural similarity with UniProt:P29803 inferred from sequence or structural similarity with UniProtKB:P08559 <newline> mitochondrion ; GO:0005739 | inferred from biological aspect of ancestor with PANTHER:PTN000162186	pyruvate dehydrogenase (acetyl-transferring) activity ; GO:0004739 | inferred from sequence or structural similarity with UniProt:P29803 inferred from sequence or structural similarity with UniProtKB:P08559	Hsap\PDHA1 <newline> Hsap\PDHA2	X
H1	CG7024	FBgn0029722	CG7024	-	CG7024	acetyl-CoA biosynthetic process from pyruvate ; GO:0006086 | inferred from electronic annotation with InterPro:IPR017597	mitochondrion ; GO:0005739 | inferred from biological aspect of ancestor with PANTHER:PTN000162186	pyruvate dehydrogenase (acetyl-transferring) activity ; GO:0004739 | inferred from electronic annotation with InterPro:IPR017597	Hsap\PDHA2 <newline> Hsap\PDHA1	X

H1	Proc-R	FBgn0029723	CG6986	Proctolin receptor	Proc-R	G protein-coupled receptor signaling pathway ; GO:0007186 | inferred from sequence model <newline> G protein-coupled receptor signaling pathway ; GO:0007186 | inferred from sequence or structural similarity <newline> neuropeptide signaling pathway ; GO:0007218 | inferred from direct assay	integral component of membrane ; GO:0016021 | inferred from sequence or structural similarity <newline> integral component of membrane ; GO:0016021 | inferred from sequence model <newline> integral component of plasma membrane ; GO:0005887 | inferred from direct assay	neuropeptide receptor activity ; GO:0008188 | inferred from sequence model <newline> proctolin receptor activity ; GO:0035236 | inferred from physical interaction with Proc <newline> proctolin receptor activity ; GO:0035236 | inferred from direct assay <newline> G protein-coupled receptor activity ; GO:0004930 | inferred from sequence or structural similarity	Hsap\GPR139 <newline> Hsap\CCR1 <newline> Hsap\GPR18	X
H1	CG15472	FBgn0029724	CG15472	-	CG15472	-	-	-	-	X
H1	Klf15	FBgn0025679	CG2932	Kruppel-like factor 15	Klf15	response to silver ion ; GO:0010272 | inferred from mutant phenotype <newline> garland nephrocyte differentiation ; GO:0061321 | inferred from mutant phenotype <newline> nephrocyte diaphragm assembly ; GO:0036059 | inferred from mutant phenotype <newline> regulation of transcription by RNA polymerase II ; GO:0006357 | inferred from biological aspect of ancestor with PANTHER:PTN001145452 <newline> pericardial nephrocyte differentiation ; GO:0061320 | inferred from mutant phenotype <newline> positive regulation of transcription by RNA polymerase II ; GO:0045944 | inferred from sequence or structural similarity with HGNC:14536	nucleus ; GO:0005634 | inferred from sequence or structural similarity with HGNC:14536 <newline> nucleus ; GO:0005634 | inferred from biological aspect of ancestor with PANTHER:PTN001145452	DNA-binding transcription factor activity, RNA polymerase II-specific ; GO:0000981 | inferred from biological aspect of ancestor with PANTHER:PTN001145452 <newline> sequence-specific DNA binding ; GO:0043565 | inferred from direct assay	Hsap\ZNF695 <newline> Hsap\ZNF491 <newline> Hsap\EGR3 <newline> Hsap\ZNF85 <newline> Hsap\KLF15 <newline> Hsap\ZNF626 <newline> Hsap\KLF17 <newline> Hsap\ZNF254 <newline> Hsap\ZNF737 <newline> Hsap\KLF11 <newline> Hsap\ZNF30 <newline> Hsap\ZNF674 <newline> Hsap\EGR1 <newline> Hsap\ZNF331 <newline> Hsap\ZNF140 <newline> Hsap\SP2 <newline> Hsap\EGR2 <newline> Hsap\KLF9 <newline> Hsap\EGR4 <newline> Hsap\OSR1 <newline> Hsap\ZNF492 <newline> Hsap\ZNF273 <newline> Hsap\ZNF100 <newline> Hsap\OSR2	X
H1	CR45515	FBgn0267071	CR45515	long non-coding RNA:CR45515	lncRNA:CR45515	-	-	-	-	X
H1	CG2871	FBgn0029725	CG2871	-	CG2871	biological_process ; GO:0008150 | no biological data available	cellular_component ; GO:0005575 | no biological data available	molecular_function ; GO:0003674 | no biological data available	-	X
H1	CG15471	FBgn0029726	CG15471	-	CG15471	biological_process ; GO:0008150 | no biological data available	cellular_component ; GO:0005575 | no biological data available	molecular_function ; GO:0003674 | no biological data available	Hsap\LYSMD3 <newline> Hsap\LYSMD4	X

H1	CG6978	FBgn0029727	CG6978	-	CG6978	transmembrane transport ; GO:0055085 | inferred from electronic annotation with InterPro:IPR011701	plasma membrane ; GO:0005886 | inferred from biological aspect of ancestor with PANTHER:PTN000910672 <newline> integral component of membrane ; GO:0016021 | inferred from biological aspect of ancestor with PANTHER:PTN000184222	-	Hsap\SLC17A2 <newline> Hsap\SLC17A5 <newline> Hsap\SLC17A1 <newline> Hsap\SLC17A9 <newline> Hsap\SLC17A3 <newline> Hsap\SLC17A8 <newline> Hsap\SLC17A6 <newline> Hsap\SLC17A7 <newline> Hsap\SLC17A4	X
H1	CG2861	FBgn0029728	CG2861	-	CG2861	biological_process ; GO:0008150 | no biological data available	cellular_component ; GO:0005575 | no biological data available	molecular_function ; GO:0003674 | no biological data available	-	X
H1	CR45516	FBgn0267072	CR45516	long non-coding RNA:CR45516	lncRNA:CR45516	-	-	-	-	X
H1	CG12682	FBgn0029729	CG12682	-	CG12682	biological_process ; GO:0008150 | no biological data available	cellular_component ; GO:0005575 | no biological data available	molecular_function ; GO:0003674 | no biological data available	-	X
H1	CG42594	FBgn0260971	CG42594	-	CG42594	potassium ion transmembrane transport ; GO:0071805 | inferred from biological aspect of ancestor with PANTHER:PTN000108561 <newline> potassium ion transmembrane transport ; GO:0071805 | inferred from sequence or structural similarity with Task7, Task6 <newline> stabilization of membrane potential ; GO:0030322 | inferred from biological aspect of ancestor with PANTHER:PTN000108561	integral component of membrane ; GO:0016021 | inferred from sequence or structural similarity with Task7, Task6 <newline> integral component of plasma membrane ; GO:0005887 | inferred from biological aspect of ancestor with PANTHER:PTN000108561	potassium channel activity ; GO:0005267 | inferred from sequence or structural similarity with Task7, Task6 <newline> potassium ion leak channel activity ; GO:0022841 | inferred from biological aspect of ancestor with PANTHER:PTN000108561	Hsap\KCNK16 <newline> Hsap\KCNK17 <newline> Hsap\KCNK1 <newline> Hsap\KCNK5 <newline> Hsap\KCNK18 <newline> Hsap\KCNK2 <newline> Hsap\KCNK10 <newline> Hsap\KCNK6 <newline> Hsap\KCNK4 <newline> Hsap\KCNK7	X
H1	CR43495	FBgn0263506	CR43495	long non-coding RNA:CR43495	lncRNA:CR43495	-	-	-	-	X
H1	boil	FBgn0029730	CG12681	boilerman	boil	-	cellular_component ; GO:0005575 | no biological data available	molecular_function ; GO:0003674 | no biological data available	-	X

H2	mthl1	FBgn0030766	CG4521	methuselah-like 1	mthl1	cell surface receptor signaling pathway ; GO:0007166 | inferred from electronic annotation with InterPro:IPR017981 <newline> G protein-coupled receptor signaling pathway ; GO:0007186 | inferred from sequence or structural similarity <newline> gastrulation ; GO:0007369 | inferred from mutant phenotype <newline> determination of adult lifespan ; GO:0008340 | inferred from sequence or structural similarity with UniProtKB:O97148 <newline> G protein-coupled receptor signaling pathway ; GO:0007186 | inferred from direct assay <newline> G protein-coupled receptor signaling pathway ; GO:0007186 | inferred from sequence or structural similarity with mth <newline> mesectoderm development ; GO:0048383 | inferred from mutant phenotype <newline> actin-mediated cell contraction ; GO:0070252 | inferred from genetic interaction with fog [illegible]	integral component of membrane ; GO:0016021 | inferred from sequence or structural similarity <newline> integral component of membrane ; GO:0016021 | inferred from sequence or structural similarity with mth	G protein-coupled receptor activity ; GO:0004930 | inferred from sequence or structural similarity with mth <newline> G protein-coupled receptor activity ; GO:0004930 | inferred from sequence or structural similarity <newline> G protein-coupled receptor activity ; GO:0004930 | inferred from physical interaction with fog	Hsap\ADGRE1 <newline> Hsap\ADGRL4 <newline> Hsap\ADGRD1 <newline> Hsap\ADGRL2 <newline> Hsap\ADGRE3 <newline> Hsap\FBN3 <newline> Hsap\ADGRG2 <newline> Hsap\ADGRF2 <newline> Hsap\ADGRD2 <newline> Hsap\ADGRG5 <newline> Hsap\ADGRG1 <newline> Hsap\ADGRL1 <newline> Hsap\ADGRF1 <newline> Hsap\ADGRE5 <newline> Hsap\ADGRE2 <newline> Hsap\ADGRG6 <newline> Hsap\ADGRG3 <newline> Hsap\ADGRG4 <newline> Hsap\ADGRL3 <newline> Hsap\ADGRF3 <newline> Hsap\ADGRG7 <newline> Hsap\ADGRF4 <newline> Hsap\ADGRF5	X
H2	CR46147	FBgn0267826	CR46147	long non-coding RNA:CR46147	lncRNA:CR46147	-	-	-	-	X
H2	CG42512	FBgn0260238	CG42512	-	CG42512	-	-	-	-	X
H2	CG32573	FBgn0052573	CG32573	-	CG32573	-	-	-	-	X
H2	CG9723	FBgn0030768	CG9723	-	CG9723	-	nuclear envelope ; GO:0005635 | inferred from biological aspect of ancestor with PANTHER:PTN001006264	-	Hsap\NEMP1 <newline> Hsap\NEMP2	X

H2	r	FBgn0003189	CG18572	rudimentary	r	de novo' pyrimidine nucleobase biosynthetic process ; GO:0006207 | inferred from mutant phenotype <newline> glutamine metabolic process ; GO:0006541 | inferred by curator from GO:0004088 <newline> citrulline biosynthetic process ; GO:0019240 | inferred from biological aspect of ancestor with PANTHER:PTN000150227 <newline> 'de novo' pyrimidine nucleobase biosynthetic process ; GO:0006207 | inferred from biological aspect of ancestor with PANTHER:PTN000150227 <newline> glutamine metabolic process ; GO:0006541 | inferred from biological aspect of ancestor with PANTHER:PTN000150223 <newline> nitrogen compound metabolic process ; GO:0006807 | inferred from biological aspect of ancestor with PANTHER:PTN000150222 <newline> UTP biosynthetic process ; GO:0006228 | inferred from biological aspect of ancestor with PANTHER:PTN000150227 <newline> [illegible]	cytoplasm ; GO:0005737 | inferred from biological aspect of ancestor with PANTHER:PTN000150222 <newline> cytosol ; GO:0005829 | inferred from biological aspect of ancestor with PANTHER:PTN000150227	ATP binding ; GO:0005524 | inferred from electronic annotation with InterPro:IPR005479, InterPro:IPR011761, InterPro:IPR013815, InterPro:IPR013816 <newline> amino acid binding ; GO:0016597 | inferred from electronic annotation with InterPro:IPR006130, InterPro:IPR006131 <newline> metal ion binding ; GO:0046872 | inferred from electronic annotation with InterPro:IPR011761 <newline> dihydroorotase activity ; GO:0004151 | inferred from mutant phenotype <newline> aspartate carbamoyltransferase activity ; GO:0004070 | inferred from biological aspect of ancestor with PANTHER:PTN000150227 <newline> dihydroorotase activity ; GO:0004151 | inferred from biological aspect of ancestor with PANTHER:PTN000150227 [illegible]	Hsap\CAD <newline> Hsap\CPS1	X
H2	CG15865	FBgn0015336	CG15865	-	CG15865	-	-	-	Hsap\C2orf54 <newline> Hsap\ITPRIP <newline> Hsap\ITPRIPL2 <newline> Hsap\MAB21L2 <newline> Hsap\MB21D2 <newline> Hsap\ITPRIPL1 <newline> Hsap\MAB21L3 <newline> Hsap\CGAS <newline> Hsap\MAB21L1 <newline> Hsap\TMEM102	X
H2	CG13012	FBgn0030769	CG13012	-	CG13012	-	-	-	-	X
H2	CG13010	FBgn0030770	CG13010	-	CG13010	biological_process ; GO:0008150 | no biological data available	cellular_component ; GO:0005575 | no biological data available	molecular_function ; GO:0003674 | no biological data available	-	X

H2	sing	FBgn0261245	CG13011	singles bar	sing	adult somatic muscle development ; GO:0007527 | inferred from mutant phenotype <newline> myoblast fusion ; GO:0007520 | inferred from mutant phenotype	integral component of membrane ; GO:0016021 | inferred from sequence model <newline> integral component of membrane ; GO:0016021 | inferred from biological aspect of ancestor with PANTHER:PTN001103553	molecular_function ; GO:0003674 | no biological data available	Hsap\PLP2 <newline> Hsap\CMTM1 <newline> Hsap\MAL <newline> Hsap\CMTM5 <newline> Hsap\CMTM4 <newline> Hsap\CMTM2 <newline> Hsap\MALL <newline> Hsap\CMTM7 <newline> Hsap\CMTM3 <newline> Hsap\PLLP <newline> Hsap\MAL2 <newline> Hsap\MARVELD1 <newline> Hsap\CMTM8 <newline> Hsap\CKLF <newline> Hsap\CMTM6	X
H2	Axs	FBgn0000152	CG9703	Abnormal X segregation	Axs	female meiosis chromosome segregation ; GO:0016321 | inferred from mutant phenotype <newline> distributive segregation ; GO:0032837 | inferred from mutant phenotype inferred from genetic interaction with Mps1 inferred from genetic interaction with ncd <newline> meiotic spindle organization ; GO:0000212 | inferred from mutant phenotype <newline> distributive segregation ; GO:0032837 | inferred from mutant phenotype	integral component of membrane ; GO:0016021 | inferred from sequence or structural similarity <newline> cell cortex ; GO:0005938 | inferred from direct assay <newline> spindle ; GO:0005819 | inferred from direct assay <newline> endoplasmic reticulum ; GO:0005783 | inferred from direct assay <newline> nuclear outer membrane ; GO:0005640 | inferred from direct assay	-	Hsap\ANO10 <newline> Hsap\ANO8	X
H2	Sep4	FBgn0259923	CG9699	Septin 4	43712	cytoskeleton-dependent cytokinesis ; GO:0061640 | inferred from biological aspect of ancestor with PANTHER:PTN000430171	microtubule cytoskeleton ; GO:0015630 | inferred from biological aspect of ancestor with PANTHER:PTN000430171 <newline> septin complex ; GO:0031105 | inferred from biological aspect of ancestor with PANTHER:PTN000430171 <newline> septin ring ; GO:0005940 | inferred from biological aspect of ancestor with PANTHER:PTN000430171	GTP binding ; GO:0005525 | inferred from electronic annotation with InterPro:IPR016491, InterPro:IPR030379 <newline> GTPase activity ; GO:0003924 | inferred from sequence or structural similarity with pnut <newline> GTPase activity ; GO:0003924 | inferred from biological aspect of ancestor with PANTHER:PTN000430171	Hsap\SEPT2 <newline> Hsap\SEPT8 <newline> Hsap\SEPT10 <newline> Hsap\SEPT4 <newline> Hsap\SEPT9 <newline> Hsap\SEPT3 <newline> Hsap\SEPT1 <newline> Hsap\SEPT11 <newline> Hsap\SEPT12 <newline> Hsap\SEPT5 <newline> Hsap\SEPT6 <newline> Hsap\TMEM250 <newline> Hsap\SEPT7 <newline> Hsap\SEPT14	X

H2	CG4678	FBgn0030778	CG4678	-	CG4678	peptide metabolic process ; GO:0006518 | inferred from biological aspect of ancestor with PANTHER:PTN000164240 <newline> protein processing ; GO:0016485 | inferred from biological aspect of ancestor with PANTHER:PTN000164240	extracellular space ; GO:0005615 | inferred from biological aspect of ancestor with PANTHER:PTN000164240	zinc ion binding ; GO:0008270 | inferred from electronic annotation with InterPro:IPR000834 <newline> metallocarboxypeptidase activity ; GO:0004181 | inferred from biological aspect of ancestor with PANTHER:PTN000164240 <newline> serine-type carboxypeptidase activity ; GO:0004185 | inferred from electronic annotation with InterPro:IPR015567	Hsap\CPXM1 <newline> Hsap\CPN1 <newline> Hsap\CPXM2 <newline> Hsap\CPD <newline> Hsap\CPM <newline> Hsap\CPE <newline> Hsap\AEBP1 <newline> Hsap\CPZ	X
H2	CG9676	FBgn0030773	CG9676	-	CG9676	proteolysis ; GO:0006508 | inferred from electronic annotation with InterPro:IPR001254, InterPro:IPR001314, InterPro:IPR018114 <newline> proteolysis ; GO:0006508 | inferred from sequence model	-	serine-type endopeptidase activity ; GO:0004252 | inferred from sequence model <newline> serine-type endopeptidase activity ; GO:0004252 | inferred from biological aspect of ancestor with PANTHER:PTN001208729	Hsap\PRSS36 <newline> Hsap\PRSS53	X
H2	sphe	FBgn0030774	CG9675	spheroide	sphe	proteolysis ; GO:0006508 | inferred from electronic annotation with InterPro:IPR001254, InterPro:IPR001314 <newline> innate immune response ; GO:0045087 | inferred from mutant phenotype <newline> positive regulation of Toll signaling pathway ; GO:0045752 | inferred from mutant phenotype <newline> defense response to Gram-positive bacterium ; GO:0050830 | inferred from mutant phenotype <newline> positive regulation of antibacterial peptide biosynthetic process ; GO:0006963 | inferred from mutant phenotype <newline> defense response to fungus ; GO:0050832 | inferred from mutant phenotype	extracellular region ; GO:0005576 | inferred by curator from GO:0050832	serine-type endopeptidase activity ; GO:0004252 | NOT inferred from key residues	Hsap\PRSS53 <newline> Hsap\PRSS38 <newline> Hsap\PRSS36	X

H2	CG9673	FBgn0030775	CG9673	-	CG9673	proteolysis ; GO:0006508 | inferred from biological aspect of ancestor with PANTHER:PTN001207268	extracellular space ; GO:0005615 | inferred from biological aspect of ancestor with PANTHER:PTN001207268	serine-type endopeptidase activity ; GO:0004252 | inferred from biological aspect of ancestor with PANTHER:PTN001207268 <newline> serine-type endopeptidase activity ; GO:0004252 | NOT inferred from key residues	Hsap\TPSD1	X
H2	CG4653	FBgn0030776	CG4653	-	CG4653	proteolysis ; GO:0006508 | inferred from electronic annotation with InterPro:IPR001254, InterPro:IPR001314, InterPro:IPR018114	-	serine-type endopeptidase activity ; GO:0004252 | inferred from biological aspect of ancestor with PANTHER:PTN001208729 <newline> serine-type endopeptidase activity ; GO:0004252 | NOT inferred from key residues	Hsap\PRSS53 <newline> Hsap\PRSS36 <newline> Hsap\TPSD1	X
H2	CG9672	FBgn0030777	CG9672	-	CG9672	proteolysis ; GO:0006508 | inferred from electronic annotation with InterPro:IPR001254, InterPro:IPR001314	extracellular space ; GO:0005615 | inferred from biological aspect of ancestor with PANTHER:PTN000669108	serine-type endopeptidase activity ; GO:0004252 | NOT inferred from key residues	Hsap\PROC <newline> Hsap\PROZ <newline> Hsap\F10 <newline> Hsap\PRSS56 <newline> Hsap\F7 <newline> Hsap\F9	X
H2	CG13008	FBgn0030780	CG13008	-	CG13008	biological_process ; GO:0008150 | no biological data available	cellular_component ; GO:0005575 | no biological data available	molecular_function ; GO:0003674 | no biological data available	-	X
H2	CG34325	FBgn0085354	CG34325	-	CG34325	-	focal adhesion ; GO:0005925 | inferred from biological aspect of ancestor with PANTHER:PTN001198287 <newline> cytosol ; GO:0005829 | inferred from biological aspect of ancestor with PANTHER:PTN001198287 <newline> plasma membrane ; GO:0005886 | inferred from biological aspect of ancestor with PANTHER:PTN001198287	transcription coregulator activity ; GO:0003712 | inferred from biological aspect of ancestor with PANTHER:PTN001198287	Hsap\PDLIM7 <newline> Hsap\FHL1 <newline> Hsap\FHL2 <newline> Hsap\LIMS4 <newline> Hsap\LDB3 <newline> Hsap\PDLIM1 <newline> Hsap\FHL3 <newline> Hsap\LIMS1 <newline> Hsap\PDLIM4 <newline> Hsap\LIMS2 <newline> Hsap\FHL5 <newline> Hsap\PDLIM5 <newline> Hsap\PDLIM2 <newline> Hsap\PDLIM3 <newline> Hsap\PXN <newline> Hsap\LIMS3 <newline> Hsap\LPXN <newline> Hsap\TGFB1I1	X
H2	CG32572	FBgn0052572	CG32572	-	CG32572	-	-	-	-	X

H2	goe	FBgn0027528	CG9634	gone early	goe	proteolysis ; GO:0006508 | inferred from electronic annotation with InterPro:IPR000718, InterPro:IPR008753 <newline> oogenesis ; GO:0048477 | inferred from mutant phenotype <newline> negative regulation of stem cell differentiation ; GO:2000737 | inferred from mutant phenotype	plasma membrane ; GO:0005886 | inferred from direct assay	metalloendopeptidase activity ; GO:0004222 | inferred from electronic annotation with InterPro:IPR000718	Hsap\ECEL1 <newline> Hsap\ECE1 <newline> Hsap\ECE2 <newline> Hsap\MMEL1 <newline> Hsap\MME <newline> Hsap\KEL <newline> Hsap\PHEX	X
H2	TwdlX	FBgn0052571	CG32571	TweedleX	TwdlX	body morphogenesis ; GO:0010171 | inferred from sequence or structural similarity with TwdlD <newline> chitin-based cuticle development ; GO:0040003 | inferred from sequence or structural similarity with TwdlD	extracellular matrix ; GO:0031012 | inferred from sequence or structural similarity with TwdlD	structural constituent of chitin-based cuticle ; GO:0005214 | inferred from sequence or structural similarity with TwdlD	-	X
H2	TwdlY	FBgn0052570	CG32570	TweedleY	TwdlY	body morphogenesis ; GO:0010171 | inferred from sequence or structural similarity with TwdlD <newline> chitin-based cuticle development ; GO:0040003 | inferred from sequence or structural similarity with TwdlD	extracellular matrix ; GO:0031012 | inferred from sequence or structural similarity with TwdlD	structural constituent of chitin-based cuticle ; GO:0005214 | inferred from sequence or structural similarity with TwdlD	-	X
H2	TwdlZ	FBgn0052569	CG32569	TweedleZ	TwdlZ	chitin-based cuticle development ; GO:0040003 | inferred from sequence or structural similarity with TwdlD <newline> body morphogenesis ; GO:0010171 | inferred from sequence or structural similarity with TwdlD	extracellular matrix ; GO:0031012 | inferred from sequence or structural similarity with TwdlD	structural constituent of chitin-based cuticle ; GO:0005214 | inferred from sequence or structural similarity with TwdlD	-	X
H2	CG32568	FBgn0052568	CG32568	-	CG32568	signal transduction ; GO:0007165 | inferred from electronic annotation with InterPro:IPR002554 <newline> regulation of protein autophosphorylation ; GO:0031952 | inferred from biological aspect of ancestor with PANTHER:PTN002567314 <newline> protein dephosphorylation ; GO:0006470 | inferred from biological aspect of ancestor with PANTHER:PTN002567314	cytosol ; GO:0005829 | inferred from biological aspect of ancestor with PANTHER:PTN002567314 <newline> protein phosphatase type 2A complex ; GO:0000159 | inferred from biological aspect of ancestor with PANTHER:PTN002567314 <newline> nucleus ; GO:0005634 | inferred from biological aspect of ancestor with PANTHER:PTN002567314	protein phosphatase activator activity ; GO:0072542 | inferred from biological aspect of ancestor with PANTHER:PTN002567314	Hsap\PPP2R5B <newline> Hsap\PPP2R5D <newline> Hsap\PPP2R5E <newline> Hsap\PPP2R5C <newline> Hsap\PPP2R5A	X

H2	Twdlalpha	FBgn0052574	CG32574	Tweedlealpha	Twdlalpha	chitin-based cuticle development ; GO:0040003 | inferred from sequence or structural similarity with TwdlD <newline> body morphogenesis ; GO:0010171 | inferred from sequence or structural similarity with TwdlD	extracellular matrix ; GO:0031012 | inferred from sequence or structural similarity with TwdlD	structural constituent of chitin-based cuticle ; GO:0005214 | inferred from sequence or structural similarity with TwdlD	Hsap\PPP2R5A <newline> Hsap\PPP2R5E <newline> Hsap\PPP2R5D <newline> Hsap\PPP2R5C <newline> Hsap\PPP2R5B	X
H2	if	FBgn0001250	CG9623	inflated	if	heterophilic cell-cell adhesion via plasma membrane cell adhesion molecules ; GO:0007157 | traceable author statement <newline> cell adhesion mediated by integrin ; GO:0033627 | inferred from direct assay <newline> maintenance of epithelial integrity, open tracheal system ; GO:0035160 | inferred from genetic interaction with mys <newline> salivary gland development ; GO:0007431 | traceable author statement <newline> imaginal disc-derived wing morphogenesis ; GO:0007476 | traceable author statement <newline> sarcomere organization ; GO:0045214 | inferred from mutant phenotype <newline> substrate adhesion-dependent cell spreading ; GO:0034446 | inferred from direct assay <newline> muscle attachment ; GO:0016203 | inferred from mutant phenotype <newline> imaginal disc-derived wing morphogenesis ; GO:0007476 | inferred from expression pattern [illegible]	integrin complex ; GO:0008305 | inferred from direct assay <newline> basal plasma membrane ; GO:0009925 | inferred from direct assay <newline> sarcolemma ; GO:0042383 | inferred from direct assay <newline> integrin complex ; GO:0008305 | non-traceable author statement <newline> focal adhesion ; GO:0005925 | inferred from direct assay <newline> plasma membrane ; GO:0005886 | inferred from direct assay <newline> integrin complex ; GO:0008305 | inferred from sequence or structural similarity <newline> integral component of plasma membrane ; GO:0005887 | inferred from direct assay <newline> integrin complex ; GO:0008305 | traceable author statement	extracellular matrix binding ; GO:0050840 | inferred from direct assay <newline> extracellular matrix protein binding ; GO:1990430 | contributes_to inferred from physical interaction with Tig <newline> protein heterodimerization activity ; GO:0046982 | inferred from physical interaction with mys	Hsap\ITGAE <newline> Hsap\ITGAD <newline> Hsap\ITGAL <newline> Hsap\ITGA10 <newline> Hsap\ITGAM <newline> Hsap\ITGA2B <newline> Hsap\ITGA8 <newline> Hsap\ITGA2 <newline> Hsap\ITGAV <newline> Hsap\ITGA4 <newline> Hsap\ITGA5 <newline> Hsap\ITGA1 <newline> Hsap\ITGA9 <newline> Hsap\ITGAX <newline> Hsap\ITGA11	X

H2	mRpL22	FBgn0030786	CG4742	mitochondrial ribosomal protein L22	mRpL22	mitochondrial translation ; GO:0032543 | traceable author statement <newline> ribosome assembly ; GO:0042255 | inferred from biological aspect of ancestor with PANTHER:PTN000337444 <newline> translation ; GO:0006412 | inferred from sequence or structural similarity with EMBL:AA772054	mitochondrion ; GO:0005739 | inferred from sequence or structural similarity with UniProtKB:Q3SZX5 <newline> mitochondrial large ribosomal subunit ; GO:0005762 | inferred from sequence or structural similarity with EMBL:AA772054 <newline> large ribosomal subunit ; GO:0015934 | inferred from biological aspect of ancestor with PANTHER:PTN000337444 <newline> mitochondrial large ribosomal subunit ; GO:0005762 | traceable author statement <newline> mitochondrial large ribosomal subunit ; GO:0005762 | inferred from sequence or structural similarity with UniProtKB:Q9NWU5 <newline> mitochondrial large ribosomal subunit ; GO:0005762 | inferred from biological aspect of ancestor with PANTHER:PTN000337445	structural constituent of ribosome ; GO:0003735 | inferred from biological aspect of ancestor with PANTHER:PTN000337444 <newline> structural constituent of ribosome ; GO:0003735 | inferred from sequence or structural similarity with EMBL:AA772054 <newline> structural constituent of ribosome ; GO:0003735 | traceable author statement	Hsap\MRPL22	X
H2	CG9609	FBgn0030787	CG9609	-	CG9609	-	nucleus ; GO:0005634 | inferred from sequence or structural similarity with UniProtKB:P21192	proximal promoter sequence-specific DNA binding ; GO:0000987 | inferred from sequence or structural similarity with UniProtKB:P21192	Hsap\ZNF557 <newline> Hsap\GTF3A	X
H2	Sap30	FBgn0030788	CG4756	SIN3-associated polypeptide 30	Sap30	regulation of transcription, DNA-templated ; GO:0006355 | inferred from biological aspect of ancestor with PANTHER:PTN000328752 <newline> regulation of G2/M transition of mitotic cell cycle ; GO:0010389 | inferred from mutant phenotype	histone deacetylase complex ; GO:0000118 | inferred from biological aspect of ancestor with PANTHER:PTN000328752 <newline> histone deacetylase complex ; GO:0000118 | inferred from sequence or structural similarity with UniProtKB:O75446	histone deacetylase activity ; GO:0004407 | inferred from biological aspect of ancestor with PANTHER:PTN000328752	Hsap\SAP30 <newline> Hsap\SAP30L	X

H2	Rrp45	FBgn0030789	CG9606	Rrp45	Rrp45	U5 snRNA 3'-end processing ; GO:0034476 | inferred from biological aspect of ancestor with PANTHER:PTN000120504 <newline> U4 snRNA 3'-end processing ; GO:0034475 | inferred from biological aspect of ancestor with PANTHER:PTN000120504 <newline> nuclear polyadenylation-dependent tRNA catabolic process ; GO:0071038 | inferred from biological aspect of ancestor with PANTHER:PTN000120504 <newline> U1 snRNA 3'-end processing ; GO:0034473 | inferred from biological aspect of ancestor with PANTHER:PTN000120504 <newline> nuclear polyadenylation-dependent rRNA catabolic process ; GO:0071035 | inferred from biological aspect of ancestor with PANTHER:PTN000120504 <newline> exonucleolytic catabolism of deadenylated mRNA ; GO:0043928 | inferred from biological aspect of ancestor with PANTHER:PTN000120504 <newline> [illegible]	exosome (RNase complex) ; GO:0000178 | inferred from direct assay <newline> nuclear exosome (RNase complex) ; GO:0000176 | inferred from biological aspect of ancestor with PANTHER:PTN000120503 <newline> cytoplasmic exosome (RNase complex) ; GO:0000177 | inferred from biological aspect of ancestor with PANTHER:PTN000120503 <newline> exosome (RNase complex) ; GO:0000178 | inferred from sequence or structural similarity with SGD:S000002688	-	Hsap\EXOSC9 <newline> Hsap\EXOSC8 <newline> Hsap\EXOSC7	X
H2	CG4768	FBgn0030790	CG4768	-	CG4768	-	-	-	Hsap\C16orf72 <newline> Hsap\LOC389895	X
H2	CG9132	FBgn0030791	CG9132	-	CG9132	endocytosis ; GO:0006897 | inferred from electronic annotation with InterPro:IPR012466	clathrin vesicle coat ; GO:0030125 | inferred from biological aspect of ancestor with PANTHER:PTN000303985	molecular_function ; GO:0003674 | no biological data available	Hsap\NECAP2 <newline> Hsap\NECAP1	X
H2	CG4789	FBgn0030792	CG4789	-	CG4789	intracellular protein transport ; GO:0006886 | inferred from biological aspect of ancestor with PANTHER:PTN001292747 <newline> Rab protein signal transduction ; GO:0032482 | inferred from biological aspect of ancestor with PANTHER:PTN001292747	cellular_component ; GO:0005575 | no biological data available	GTP binding ; GO:0005525 | inferred from electronic annotation with InterPro:IPR001806 <newline> GTPase activity ; GO:0003924 | inferred from biological aspect of ancestor with PANTHER:PTN001292747 <newline> GTPase activity ; GO:0003924 | inferred from sequence or structural similarity with SGD:S000001722	Hsap\RABL3	X
H2	CG9125	FBgn0030793	CG9125	-	CG9125	mRNA catabolic process ; GO:0006402 | inferred from sequence or structural similarity with MGI:MGI:1890444	nucleus ; GO:0005634 | inferred from sequence or structural similarity with MGI:MGI:1890444	5'-3' exonuclease activity ; GO:0008409 | inferred from sequence or structural similarity with MGI:MGI:1890444	Hsap\DXO	X

H2	CG13005	FBgn0030794	CG13005	-	CG13005	biological_process ; GO:0008150 | no biological data available	cellular_component ; GO:0005575 | no biological data available	molecular_function ; GO:0003674 | no biological data available	-	X
H2	CR44053	FBgn0264862	CR44053	antisense RNA:CR44053	asRNA:CR44053	-	-	-	-	X
H2	ppk28	FBgn0030795	CG4805	pickpocket 28	ppk28	drinking behavior ; GO:0042756 | inferred from mutant phenotype <newline> response to water ; GO:0009415 | inferred from direct assay <newline> liquid clearance, open tracheal system ; GO:0035002 | inferred from mutant phenotype <newline> sodium ion transport ; GO:0006814 | inferred from sequence or structural similarity with MGI:MGI:101782 <newline> cellular response to water stimulus ; GO:0071462 | inferred from mutant phenotype	integral component of membrane ; GO:0016021 | inferred from sequence or structural similarity with MGI:MGI:101782	sodium channel activity ; GO:0005272 | inferred from sequence or structural similarity with MGI:MGI:101782	Hsap\ASIC1 <newline> Hsap\SCNN1D <newline> Hsap\ASIC4 <newline> Hsap\ASIC5 <newline> Hsap\ASIC2 <newline> Hsap\ASIC3 <newline> Hsap\SCNN1A <newline> Hsap\SCNN1G <newline> Hsap\SCNN1B	X
H2	CG4829	FBgn0030796	CG4829	-	CG4829	glutathione catabolic process ; GO:0006751 | inferred from biological aspect of ancestor with PANTHER:PTN002269349	plasma membrane ; GO:0005886 | inferred from biological aspect of ancestor with PANTHER:PTN002269349	glutathione hydrolase activity ; GO:0036374 | inferred from biological aspect of ancestor with PANTHER:PTN002269349 <newline> peptidyltransferase activity ; GO:0000048 | inferred from biological aspect of ancestor with PANTHER:PTN002269349	Hsap\GGTLC2 <newline> Hsap\GGT7 <newline> Hsap\GGT2 <newline> Hsap\GGT1 <newline> Hsap\GGTLC1 <newline> Hsap\GGT6 <newline> Hsap\GGT5	X
H2	CR44129	FBgn0264960	CR44129	long non-coding RNA:CR44129	lncRNA:CR44129	-	-	-	-	X
H2	CG4829	FBgn0030796	CG4829	-	CG4829	glutathione catabolic process ; GO:0006751 | inferred from biological aspect of ancestor with PANTHER:PTN002269349	plasma membrane ; GO:0005886 | inferred from biological aspect of ancestor with PANTHER:PTN002269349	glutathione hydrolase activity ; GO:0036374 | inferred from biological aspect of ancestor with PANTHER:PTN002269349 <newline> peptidyltransferase activity ; GO:0000048 | inferred from biological aspect of ancestor with PANTHER:PTN002269349	Hsap\GGTLC2 <newline> Hsap\GGT7 <newline> Hsap\GGT2 <newline> Hsap\GGT1 <newline> Hsap\GGTLC1 <newline> Hsap\GGT6 <newline> Hsap\GGT5	X
H2	CG13004	FBgn0030797	CG13004	-	CG13004	-	-	-	-	X
H2	CG46306	FBgn0284222	CG46306	-	CG46306	-	-	-	-	X

H2	RSG7	FBgn0024941	CG9108	Regulator of G-protein signaling 7	RSG7	intracellular signal transduction ; GO:0035556 | inferred from electronic annotation with InterPro:IPR000591 <newline> regulation of G protein-coupled receptor signaling pathway ; GO:0008277 | inferred from sequence or structural similarity with WB:WBGene00001179	-	-	Hsap\RGS19 <newline> Hsap\RGS17 <newline> Hsap\RGS2 <newline> Hsap\RGS9 <newline> Hsap\RGS12 <newline> Hsap\AXIN2 <newline> Hsap\RGS6 <newline> Hsap\RGS5 <newline> Hsap\RGS21 <newline> Hsap\RGS13 <newline> Hsap\RGS1 <newline> Hsap\RGS7 <newline> Hsap\RGS20 <newline> Hsap\RGS16 <newline> Hsap\RGS4 <newline> Hsap\RGS8 <newline> Hsap\RGS3 <newline> Hsap\RGS14 <newline> Hsap\RGS10 <newline> Hsap\RGS18 <newline> Hsap\AXIN1 <newline> Hsap\RGS11	X
H2	CG13003	FBgn0030798	CG13003	-	CG13003	-	-	-	-	X
H2	CG4872	FBgn0030799	CG4872	-	CG4872	negative regulation of transcription by RNA polymerase II ; GO:0000122 | inferred from sequence or structural similarity with HGNC:2418	cytoplasm ; GO:0005737 | inferred from sequence or structural similarity with HGNC:2418 <newline> cytoplasm ; GO:0005737 | inferred from biological aspect of ancestor with PANTHER:PTN000352164	transcription corepressor activity ; GO:0003714 | inferred from sequence or structural similarity with HGNC:2418 <newline> hormone binding ; GO:0042562 | inferred from biological aspect of ancestor with PANTHER:PTN001013561	Hsap\CRYM	X

H2	Nprl2	FBgn0030800	CG9104	Nitrogen permease regulator-like 2	Nprl2	germline cell cycle switching, mitotic to meiotic cell cycle ; GO:0051729 | inferred from genetic interaction with UniProtKB:Q9W0E3 <newline> negative regulation of TOR signaling ; GO:0032007 | inferred from mutant phenotype <newline> negative regulation of cell size ; GO:0045792 | inferred from mutant phenotype <newline> negative regulation of TORC1 signaling ; GO:1904262 | inferred from mutant phenotype <newline> TORC1 signaling ; GO:0038202 | inferred from mutant phenotype <newline> negative regulation of macroautophagy by TORC1 signaling ; GO:1904766 | inferred from mutant phenotype <newline> positive regulation of autophagy ; GO:0010508 | inferred from biological aspect of ancestor with PANTHER:PTN000988255 <newline> positive regulation of triglyceride catabolic process ; GO:0010898 | inferred from mutant phenotype <newline> cellular response to	autolysosome ; GO:0044754 | inferred from direct assay <newline> Seh1-associated complex ; GO:0035859 | inferred from sequence or structural similarity with SGD:S000000788 <newline> cytoplasm ; GO:0005737 | inferred from direct assay <newline> GATOR1 complex ; GO:1990130 | traceable author statement <newline> Seh1-associated complex ; GO:0035859 | inferred from direct assay <newline> GATOR1 complex ; GO:1990130 | inferred from biological aspect of ancestor with PANTHER:PTN000988255 <newline> nucleus ; GO:0005634 | inferred from direct assay	protein binding ; GO:0005515 | inferred from physical interaction with Nprl3	Hsap\NPRL2 <newline> Hsap\ZMYND10	X
H2	Rcp	FBgn0030801	CG4875	Receptor component protein	Rcp	positive regulation of G protein-coupled receptor signaling pathway ; GO:0045745 | inferred from direct assay <newline> transcription initiation from RNA polymerase III promoter ; GO:0006384 | inferred from biological aspect of ancestor with PANTHER:PTN000399700	RNA polymerase III complex ; GO:0005666 | inferred from biological aspect of ancestor with PANTHER:PTN000399700	nucleotide binding ; GO:0000166 | inferred from electronic annotation with InterPro:IPR010997 <newline> RNA polymerase III activity ; GO:0001056 | contributes_to inferred from biological aspect of ancestor with PANTHER:PTN000399700	Hsap\CRCP <newline> Hsap\POLR2D <newline> Hsap\ASL	X

H2	DENR	FBgn0030802	CG9099	Density regulated protein	DENR	formation of translation preinitiation complex ; GO:0001731 | inferred from biological aspect of ancestor with PANTHER:PTN000300443 <newline> positive regulation of insulin receptor signaling pathway ; GO:0046628 | inferred from mutant phenotype <newline> positive regulation of ecdysone receptor-mediated signaling pathway ; GO:0120142 | inferred from mutant phenotype <newline> positive regulation of translational initiation ; GO:0045948 | inferred from mutant phenotype <newline> translation reinitiation ; GO:0002188 | inferred from direct assay <newline> translation reinitiation ; GO:0002188 | inferred from biological aspect of ancestor with PANTHER:PTN000300443 <newline> positive regulation of cell population proliferation ; GO:0008284 | inferred from mutant phenotype	protein-containing complex ; GO:0032991 | inferred from physical interaction with MCTS1	translation initiation factor activity ; GO:0003743 | inferred from electronic annotation with InterPro:IPR001950, InterPro:IPR005872, InterPro:IPR036877 <newline> mRNA binding ; GO:0003729 | inferred from direct assay <newline> mRNA binding ; GO:0003729 | inferred from biological aspect of ancestor with PANTHER:PTN000300443	Hsap\DENR	X
H2	CG4880	FBgn0030803	CG4880	-	CG4880	cellular response to misfolded protein ; GO:0071218 | inferred from biological aspect of ancestor with PANTHER:PTN002583237	endoplasmic reticulum ; GO:0005783 | inferred from electronic annotation with InterPro:IPR030553 <newline> nuclear envelope ; GO:0005635 | inferred from electronic annotation with InterPro:IPR030553	ATP binding ; GO:0005524 | inferred from electronic annotation with InterPro:IPR010448, InterPro:IPR030553 <newline> ATPase activity ; GO:0016887 | inferred from biological aspect of ancestor with PANTHER:PTN000844597	Hsap\TOR1A <newline> Hsap\TOR4A	X
H2	CR43768	FBgn0264268	CR43768	long non-coding RNA:CR43768	lncRNA:CR43768	-	-	-	-	X
H2	CG13002	FBgn0030804	CG13002	-	CG13002	biological_process ; GO:0008150 | no biological data available	cellular_component ; GO:0005575 | no biological data available	molecular_function ; GO:0003674 | no biological data available	-	X
H2	CG33252	FBgn0053252	CG33252	-	CG33252	biological_process ; GO:0008150 | no biological data available	cellular_component ; GO:0005575 | no biological data available	molecular_function ; GO:0003674 | no biological data available	-	X
H2	CR44754	FBgn0265967	CR44754	long non-coding RNA:CR44754	lncRNA:CR44754	-	-	-	-	X

H2	CG4928	FBgn0027556	CG4928	-	CG4928	regulation of potassium ion transport ; GO:0043266 | inferred from biological aspect of ancestor with PANTHER:PTN000455664 <newline> regulation of muscle contraction ; GO:0006937 | inferred from biological aspect of ancestor with PANTHER:PTN000455664	plasma membrane ; GO:0005886 | inferred from biological aspect of ancestor with PANTHER:PTN000455664 <newline> striated muscle dense body ; GO:0055120 | inferred from biological aspect of ancestor with PANTHER:PTN000455664 <newline> plasma membrane ; GO:0005886 | inferred from sequence or structural similarity with UniProtKB:Q86WB7	potassium channel regulator activity ; GO:0015459 | inferred from biological aspect of ancestor with PANTHER:PTN000455664	Hsap\UNC93A <newline> Hsap\UNC93B1	X
H2	wus	FBgn0030805	CG9089	wurst	wus	liquid clearance, open tracheal system ; GO:0035002 | inferred from mutant phenotype <newline> regulation of tube length, open tracheal system ; GO:0035159 | inferred from mutant phenotype <newline> endocytosis ; GO:0006897 | inferred from mutant phenotype <newline> extracellular matrix organization ; GO:0030198 | inferred from mutant phenotype	early endosome ; GO:0005769 | inferred from direct assay <newline> cytoplasmic vesicle ; GO:0031410 | inferred from direct assay <newline> plasma membrane ; GO:0005886 | inferred from direct assay	-	Hsap\DNAJC22 <newline> Hsap\DNAJB9 <newline> Hsap\DNAJB4 <newline> Hsap\DNAJB2 <newline> Hsap\DNAJB13 <newline> Hsap\DNAJB5 <newline> Hsap\DNAJB1 <newline> Hsap\DNAJB14 <newline> Hsap\DNAJB7 <newline> Hsap\DNAJB12 <newline> Hsap\DNAJB6 <newline> Hsap\DNAJC18 <newline> Hsap\DNAJB8	X
H2	CG13001	FBgn0030806	CG13001	-	CG13001	nervous system development ; GO:0007399 | inferred from sequence or structural similarity with HGNC:24931	cytoplasm ; GO:0005737 | inferred from sequence or structural similarity with HGNC:24931 <newline> nucleus ; GO:0005634 | inferred from sequence or structural similarity with HGNC:24931	molecular_function ; GO:0003674 | no biological data available	Hsap\ZC4H2	X
H2	RhoGAP15B	FBgn0030808	CG4937	Rho GTPase activating protein at 15B	RhoGAP15B	signal transduction ; GO:0007165 | inferred from electronic annotation with InterPro:IPR000198, InterPro:IPR008936 <newline> regulation of GTPase activity ; GO:0043087 | inferred from sequence model <newline> imaginal disc-derived leg morphogenesis ; GO:0007480 | inferred from mutant phenotype <newline> positive regulation of GTPase activity ; GO:0043547 | inferred from sequence or structural similarity with HGNC:16925	Golgi apparatus ; GO:0005794 | inferred from sequence or structural similarity with HGNC:16925	phosphatidylinositol-3,4,5-trisphosphate binding ; GO:0005547 | inferred from electronic annotation with InterPro:IPR037858 <newline> GTPase activator activity ; GO:0005096 | inferred from sequence model <newline> GTPase activator activity ; GO:0005096 | inferred from sequence or structural similarity with HGNC:16925	Hsap\ARAP3 <newline> Hsap\ARAP2 <newline> Hsap\ARAP1	X

H2	CG13000	FBgn0030807	CG13000	-	CG13000	-	-	-	-	X
H2	CG9086	FBgn0030809	CG9086	Ubr1 ubiquitin ligase	Ubr1	ubiquitin-dependent protein catabolic process via the N-end rule pathway ; GO:0071596 | inferred from biological aspect of ancestor with PANTHER:PTN000486924 <newline> protein ubiquitination ; GO:0016567 | inferred from biological aspect of ancestor with PANTHER:PTN000486924 <newline> protein ubiquitination ; GO:0016567 | inferred from sequence or structural similarity with SGD:S000003416	ubiquitin ligase complex ; GO:0000151 | inferred from biological aspect of ancestor with PANTHER:PTN000486924 <newline> cytoplasm ; GO:0005737 | inferred from sequence or structural similarity with SGD:S000003416 <newline> cytoplasm ; GO:0005737 | inferred from biological aspect of ancestor with PANTHER:PTN000486924	zinc ion binding ; GO:0008270 | inferred from electronic annotation with InterPro:IPR001841, InterPro:IPR003126 <newline> ubiquitin-protein transferase activity ; GO:0004842 | inferred from sequence or structural similarity with SGD:S000003416 <newline> ubiquitin protein ligase activity ; GO:0061630 | inferred from biological aspect of ancestor with PANTHER:PTN000486924 <newline> zinc ion binding ; GO:0008270 | inferred from sequence model	Hsap\UBR1 <newline> Hsap\UBR2	X
H2	CG9059	FBgn0266354	CG45002	-	CG45002	proteolysis ; GO:0006508 | inferred from electronic annotation with InterPro:IPR001375, InterPro:IPR002469	-	serine-type peptidase activity ; GO:0008236 | inferred from electronic annotation with InterPro:IPR001375	Hsap\APEH <newline> Hsap\DPP4 <newline> Hsap\DPP6 <newline> Hsap\DPP10 <newline> Hsap\FAP	X
H2	CG12433	FBgn0264739	CG43997	-	CG43997	biological_process ; GO:0008150 | no biological data available	cellular_component ; GO:0005575 | no biological data available	molecular_function ; GO:0003674 | no biological data available	-	X
H2	CG34326	FBgn0266354	CG45002	-	CG45002	proteolysis ; GO:0006508 | inferred from electronic annotation with InterPro:IPR001375, InterPro:IPR002469	-	serine-type peptidase activity ; GO:0008236 | inferred from electronic annotation with InterPro:IPR001375	Hsap\APEH <newline> Hsap\DPP4 <newline> Hsap\DPP6 <newline> Hsap\DPP10 <newline> Hsap\FAP	X
H2	CG43077	FBgn0262487	CG43077	-	CG43077	biological_process ; GO:0008150 | no biological data available	cellular_component ; GO:0005575 | no biological data available	molecular_function ; GO:0003674 | no biological data available	-	X
H2	CG8949	FBgn0030812	CG8949	wacky	wcy	negative regulation of autophagy ; GO:0010507 | inferred from mutant phenotype <newline> positive regulation of TORC1 signaling ; GO:1904263 | inferred from mutant phenotype <newline> habituation ; GO:0046959 | inferred from mutant phenotype <newline> histone monoubiquitination ; GO:0010390 | inferred from sequence or structural similarity with UniProtKB:Q9BTA9	lysosome ; GO:0005764 | inferred from direct assay <newline> nucleus ; GO:0005634 | inferred from direct assay	molecular_function ; GO:0003674 | no biological data available	Hsap\WAC	X

H3	CG5037	FBgn0032222	CG5037	-	CG5037	heme O biosynthetic process ; GO:0048034 | inferred from electronic annotation with InterPro:IPR006369 <newline> respiratory chain complex IV assembly ; GO:0008535 | inferred from sequence or structural similarity with HGNC:2260 <newline> heme biosynthetic process ; GO:0006783 | inferred from sequence or structural similarity with UniProtKB:Q12887 <newline> respiratory chain complex IV assembly ; GO:0008535 | inferred from sequence or structural similarity with UniProtKB:Q12887 <newline> cellular respiration ; GO:0045333 | inferred from biological aspect of ancestor with PANTHER:PTN000114243	integral component of membrane ; GO:0016021 | inferred from electronic annotation with InterPro:IPR000537, InterPro:IPR006369 <newline> mitochondrial membrane ; GO:0031966 | inferred from electronic annotation with InterPro:IPR016315 <newline> cytochrome complex ; GO:0070069 | inferred from sequence or structural similarity with HGNC:2260 <newline> mitochondrion ; GO:0005739 | inferred from biological aspect of ancestor with PANTHER:PTN000114245	protoheme IX farnesyltransferase activity ; GO:0008495 | inferred from electronic annotation with InterPro:IPR006369 <newline> cytochrome-c oxidase activity ; GO:0004129 | contributes_to inferred from sequence or structural similarity with HGNC:2260	Hsap\COX10	2L
H3	CG5375	FBgn0032221	CG5375	Schwannomin interacting protein 1	Schip1	positive regulation of protein serine/threonine kinase activity ; GO:0071902 | inferred from mutant phenotype <newline> positive regulation of hippo signaling ; GO:0035332 | inferred from mutant phenotype <newline> negative regulation of growth ; GO:0045926 | inferred from mutant phenotype	adherens junction ; GO:0005912 | inferred from direct assay <newline> apical plasma membrane ; GO:0016324 | inferred from direct assay	protein binding ; GO:0005515 | inferred from physical interaction with UniProtKB:Q0KHQ5	Hsap\SCHIP1 <newline> Hsap\IQCJ-SCHIP1	2L

H3	GATAd	FBgn0032223	CG5034	GATAd	GATAd	regulation of transcription, DNA-templated ; GO:0006355 | inferred from electronic annotation with InterPro:IPR000679, InterPro:IPR013088	nucleus ; GO:0005634 | inferred from direct assay <newline> nucleus ; GO:0005634 | inferred from biological aspect of ancestor with PANTHER:PTN001600628	zinc ion binding ; GO:0008270 | inferred from electronic annotation with InterPro:IPR000679, InterPro:IPR012934, InterPro:IPR013088 <newline> DNA-binding transcription factor activity, RNA polymerase II-specific ; GO:0000981 | inferred from biological aspect of ancestor with PANTHER:PTN001600628 <newline> RNA polymerase II proximal promoter sequence-specific DNA binding ; GO:0000978 | inferred from biological aspect of ancestor with PANTHER:PTN001600628 <newline> DNA-binding transcription factor activity, RNA polymerase II-specific ; GO:0000981 | inferred from sequence or structural similarity with MGI:MGI:107516	Hsap\GATA3 <newline> Hsap\GATA2 <newline> Hsap\TRPS1 <newline> Hsap\GATA6 <newline> Hsap\ZGLP1 <newline> Hsap\GATA5 <newline> Hsap\GATA1 <newline> Hsap\GATA4	2L
H3	SamDC	FBgn0019932	CG5029	S-adenosylmethionine decarboxylase	SamDC	defense response to Gram-negative bacterium ; GO:0050829 | inferred from mutant phenotype <newline> spermidine biosynthetic process ; GO:0008295 | inferred from biological aspect of ancestor with PANTHER:PTN000170807 <newline> spermine biosynthetic process ; GO:0006597 | inferred from biological aspect of ancestor with PANTHER:PTN000170807	cytosol ; GO:0005829 | inferred from biological aspect of ancestor with PANTHER:PTN000170807	adenosylmethionine decarboxylase activity ; GO:0004014 | inferred from biological aspect of ancestor with PANTHER:PTN000170807	Hsap\AMD1	2L
H3	CG31715	FBgn0051715	CG31715	-	CG31715	biological_process ; GO:0008150 | no biological data available	cellular_component ; GO:0005575 | no biological data available	molecular_function ; GO:0003674 | no biological data available	Hsap\ASB12 <newline> Hsap\LUZP6 <newline> Hsap\IQANK1 <newline> Hsap\MTPN	2L

H3	Sps2	FBgn0032224	CG5025	Selenophosphate synthetase 2	Sps2	selenium compound metabolic process ; GO:0001887 | inferred from biological aspect of ancestor with PANTHER:PTN000029003 <newline> tRNA seleno-modification ; GO:0070329 | inferred from biological aspect of ancestor with PANTHER:PTN000029003 <newline> selenocysteine biosynthetic process ; GO:0016260 | inferred from sequence or structural similarity with HGNC:19686 <newline> selenocysteine biosynthetic process ; GO:0016260 | inferred from biological aspect of ancestor with PANTHER:PTN000029003	cytoplasm ; GO:0005737 | inferred from biological aspect of ancestor with PANTHER:PTN000029003	ATP binding ; GO:0005524 | inferred from electronic annotation with InterPro:IPR004536 <newline> catalytic activity ; GO:0003824 | traceable author statement <newline> selenide, water dikinase activity ; GO:0004756 | inferred from biological aspect of ancestor with PANTHER:PTN000029003 <newline> ATP binding ; GO:0005524 | traceable author statement <newline> purine nucleotide binding ; GO:0017076 | inferred from sequence or structural similarity <newline> selenide, water dikinase activity ; GO:0004756 | inferred from sequence or structural similarity with HGNC:19686	Hsap\SEPHS2 <newline> Hsap\SEPHS1	2L
H3	CG5022	FBgn0032225	CG5022	-	CG5022	actomyosin structure organization ; GO:0031032 | inferred from biological aspect of ancestor with PANTHER:PTN001150527	cytoskeleton ; GO:0005856 | inferred from electronic annotation with InterPro:IPR000299	cytoskeletal protein binding ; GO:0008092 | inferred from electronic annotation with InterPro:IPR000798	Hsap\FRMD3 <newline> Hsap\EPB41L4A <newline> Hsap\FRMD5	2L
H3	CG34367	FBgn0085396	CG34367	-	CG34367	regulation of transcription, DNA-templated ; GO:0006355 | inferred from electronic annotation with InterPro:IPR017970	-	sequence-specific DNA binding ; GO:0043565 | inferred from electronic annotation with InterPro:IPR017970	Hsap\PDLIM3 <newline> Hsap\SHOX <newline> Hsap\LHX6 <newline> Hsap\SHOX2 <newline> Hsap\LPXN <newline> Hsap\PDLIM1 <newline> Hsap\LHX8 <newline> Hsap\PAX6 <newline> Hsap\PITX1 <newline> Hsap\LDB3 <newline> Hsap\PDLIM7 <newline> Hsap\HOXB8 <newline> Hsap\GSC2 <newline> Hsap\PITX3 <newline> Hsap\GSC <newline> Hsap\DMBX1	2L

H3	CG5367	FBgn0032228	CG5367	-	CG5367	proteolysis involved in cellular protein catabolic process ; GO:0051603 | inferred from biological aspect of ancestor with PANTHER:PTN000274640	lysosome ; GO:0005764 | inferred from biological aspect of ancestor with PANTHER:PTN000274640 <newline> extracellular space ; GO:0005615 | inferred from biological aspect of ancestor with PANTHER:PTN000274640	cysteine-type endopeptidase activity ; GO:0004197 | inferred from biological aspect of ancestor with PANTHER:PTN000274640	Hsap\CTSH <newline> Hsap\CTSO <newline> Hsap\CTSZ <newline> Hsap\CTSK <newline> Hsap\CTSB <newline> Hsap\CTSC <newline> Hsap\CTSS <newline> Hsap\CTSL <newline> Hsap\CTSW <newline> Hsap\CTSV	2L
H3	CG5045	FBgn0032229	CG5045	Clp protease proteolytic subunit	ClpP	protein quality control for misfolded or incompletely synthesized proteins ; GO:0006515 | inferred from biological aspect of ancestor with PANTHER:PTN000043558 <newline> proteolysis ; GO:0006508 | inferred from sequence or structural similarity with UniProtKB:Q16740	mitochondrion ; GO:0005739 | inferred from sequence or structural similarity with UniProtKB:Q16740 <newline> endopeptidase Clp complex ; GO:0009368 | inferred from biological aspect of ancestor with PANTHER:PTN000043558	ATPase binding ; GO:0051117 | inferred from biological aspect of ancestor with PANTHER:PTN000043558 <newline> ATP-dependent peptidase activity ; GO:0004176 | inferred from biological aspect of ancestor with PANTHER:PTN000043558 <newline> serine-type endopeptidase activity ; GO:0004252 | inferred from biological aspect of ancestor with PANTHER:PTN000043558	Hsap\CLPP	2L
H3	Cand1	FBgn0027568	CG5366	Cullin-associated and neddylation-dissociated 1	Cand1	negative regulation of protein neddylation ; GO:2000435 | inferred from mutant phenotype <newline> protein ubiquitination ; GO:0016567 | inferred from biological aspect of ancestor with PANTHER:PTN000294436 <newline> SCF complex assembly ; GO:0010265 | inferred from biological aspect of ancestor with PANTHER:PTN000294436 <newline> regulation of protein stability ; GO:0031647 | inferred from mutant phenotype	nucleus ; GO:0005634 | inferred from biological aspect of ancestor with PANTHER:PTN000294436	protein binding ; GO:0005515 | inferred from physical interaction with Cul1 inferred from physical interaction with Cul3	Hsap\CAND1 <newline> Hsap\CAND2	2L

H3	pim	FBgn0003087	CG5052	pimples	pim	Malpighian tubule morphogenesis ; GO:0007443 | inferred from mutant phenotype <newline> nervous system development ; GO:0007399 | inferred from mutant phenotype <newline> mitotic sister chromatid separation ; GO:0051306 | inferred from mutant phenotype <newline> mitotic sister chromatid segregation ; GO:0000070 | inferred from mutant phenotype <newline> gonad development ; GO:0008406 | inferred from mutant phenotype <newline> mitotic sister chromatid segregation ; GO:0000070 | non-traceable author statement <newline> mitotic sister chromatid segregation ; GO:0000070 | traceable author statement <newline> digestive tract mesoderm development ; GO:0007502 | inferred from mutant phenotype	nucleus ; GO:0005634 | inferred from electronic annotation with InterPro:IPR006940 <newline> cytoplasm ; GO:0005737 | inferred from direct assay	protein binding ; GO:0005515 | inferred from physical interaction with UniProtKB:P42286	-	2L
H3	lft	FBgn0032230	CG13139	lowfat	lft	autophagy ; GO:0006914 | inferred from biological aspect of ancestor with PANTHER:PTN001579556 <newline> autophagosome maturation ; GO:0097352 | inferred from biological aspect of ancestor with PANTHER:PTN001579556 <newline> imaginal disc-derived wing morphogenesis ; GO:0007476 | inferred from mutant phenotype	cytoplasm ; GO:0005737 | inferred from direct assay <newline> subapical complex ; GO:0035003 | inferred from direct assay <newline> cytoplasm ; GO:0005737 | inferred from biological aspect of ancestor with PANTHER:PTN001579556	-	Hsap\LIX1 <newline> Hsap\LIX1L	2L
H3	CG5056	FBgn0032231	CG5056	-	CG5056	cellular response to hydrogen peroxide ; GO:0070301 | inferred from biological aspect of ancestor with PANTHER:PTN001264638	-	-	Hsap\OSER1	2L

H3	rho-5	FBgn0041723	CG33304	rhomboid-5	rho-5	regulation of epidermal growth factor receptor signaling pathway ; GO:0042058 | inferred from biological aspect of ancestor with PANTHER:PTN001117529 <newline> regulation of protein secretion ; GO:0050708 | inferred from biological aspect of ancestor with PANTHER:PTN001117529 <newline> negative regulation of epidermal growth factor receptor signaling pathway ; GO:0042059 | inferred from mutant phenotype inferred from genetic interaction with Egfr inferred from genetic interaction with aos inferred from genetic interaction with spi inferred from genetic interaction with sty <newline> positive regulation of circadian sleep/wake cycle, wakefulness ; GO:0010841 | inferred from mutant phenotype	integral component of membrane ; GO:0016021 | inferred from electronic annotation with InterPro:IPR002610, InterPro:IPR022764 <newline> endoplasmic reticulum membrane ; GO:0005789 | inferred from direct assay <newline> endoplasmic reticulum membrane ; GO:0005789 | inferred from biological aspect of ancestor with PANTHER:PTN001117529 <newline> endoplasmic reticulum ; GO:0005783 | inferred from direct assay	serine-type endopeptidase activity ; GO:0004252 | NOT inferred from direct assay	Hsap\RHBDF2 <newline> Hsap\RHBDF1 <newline> Hsap\RPN1	2L
H3	CG33303	FBgn0053303	CG33303	-	CG33303	protein N-linked glycosylation via asparagine ; GO:0018279 | inferred from biological aspect of ancestor with PANTHER:PTN000471615	integral component of membrane ; GO:0016021 | inferred from electronic annotation with InterPro:IPR007676 <newline> endomembrane system ; GO:0012505 | inferred from high throughput direct assay <newline> endoplasmic reticulum ; GO:0005783 | inferred from electronic annotation with InterPro:IPR007676	dolichyl-diphosphooligosaccharide-protein glycotransferase activity ; GO:0004579 | inferred from biological aspect of ancestor with PANTHER:PTN000471615	Hsap\RPN1	2L
H3	dpr19	FBgn0032233	CG13140	defective proboscis extension response 19	dpr19	synapse organization ; GO:0050808 | inferred from expression pattern <newline> synapse organization ; GO:0050808 | inferred from biological aspect of ancestor with PANTHER:PTN000589413 <newline> sensory perception of chemical stimulus ; GO:0007606 | inferred from sequence or structural similarity with dpr1	neuron projection membrane ; GO:0032589 | inferred from biological aspect of ancestor with PANTHER:PTN000589413 <newline> integral component of plasma membrane ; GO:0005887 | inferred from sequence model	molecular_function ; GO:0003674 | no biological data available	Hsap\SDK1 <newline> Hsap\PAPLN <newline> Hsap\KIRREL3 <newline> Hsap\IGSF9B <newline> Hsap\LRIT3 <newline> Hsap\LRIT1 <newline> Hsap\KIRREL1 <newline> Hsap\CADM4 <newline> Hsap\CADM2 <newline> Hsap\CADM3 <newline> Hsap\USH2A <newline> Hsap\KIRREL2 <newline> Hsap\CADM1	2L

H3	gny	FBgn0032234	CG5091	garnysstan	gny	dolichol-linked oligosaccharide biosynthetic process ; GO:0006488 | inferred from electronic annotation with InterPro:IPR039488 <newline> protein N-linked glycosylation ; GO:0006487 | inferred from sequence or structural similarity with UniProtKB:Q9Y672 <newline> chitin-based cuticle development ; GO:0040003 | inferred from mutant phenotype <newline> protein N-linked glycosylation ; GO:0006487 | inferred from biological aspect of ancestor with PANTHER:PTN000275691 <newline> oligosaccharide-lipid intermediate biosynthetic process ; GO:0006490 | inferred from biological aspect of ancestor with PANTHER:PTN000275691	endoplasmic reticulum membrane ; GO:0005789 | inferred from biological aspect of ancestor with PANTHER:PTN000275691 <newline> endoplasmic reticulum ; GO:0005783 | inferred from sequence or structural similarity with SGD:S000005528	dolichyl pyrophosphate Man9GlcNAc2 alpha-1,3-glucosyltransferase activity ; GO:0042281 | inferred from biological aspect of ancestor with PANTHER:PTN000275691 <newline> glucosyltransferase activity ; GO:0046527 | inferred from sequence or structural similarity with UniProtKB:Q9Y672	Hsap\ALG6 <newline> Hsap\LOC107984053	2L
H3	CG5096	FBgn0032235	CG5096	-	CG5096	-	-	-	Hsap\LRRC4B <newline> Hsap\LRRC4 <newline> Hsap\LRRC4C	2L

H3	Cdk1	FBgn0004106	CG5363	Cyclin-dependent kinase 1	Cdk1	protein phosphorylation ; GO:0006468 | inferred from direct assay <newline> germarium-derived cystoblast division ; GO:0048142 | inferred from mutant phenotype <newline> G2/M transition of mitotic cell cycle ; GO:0000086 | inferred from mutant phenotype <newline> asymmetric neuroblast division ; GO:0055059 | inferred from mutant phenotype <newline> G1/S transition of mitotic cell cycle ; GO:0000082 | inferred from genetic interaction with SGD:S000000364 <newline> regulation of protein localization ; GO:0032880 | inferred from mutant phenotype <newline> embryonic development via the syncytial blastoderm ; GO:0001700 | inferred from mutant phenotype <newline> ovarian follicle cell development ; GO:0030707 | inferred from mutant phenotype <newline> mitotic G2 DNA damage checkpoint ; GO:0007095 | inferred from mutant phenotype <newline> spermatogonial cell division ; [illegible]	nucleus ; GO:0005634 | inferred from biological aspect of ancestor with PANTHER:PTN000623091	ATP binding ; GO:0005524 | inferred from electronic annotation with InterPro:IPR000719, InterPro:IPR002290, InterPro:IPR017441 <newline> cyclin-dependent protein serine/threonine kinase activity ; GO:0004693 | inferred from sequence or structural similarity with SGD:S000000364 inferred from genetic interaction with SGD:S000000364 <newline> protein binding ; GO:0005515 | inferred from physical interaction with UniProtKB:Q9I7I0 <newline> protein kinase activity ; GO:0004672 | inferred from direct assay <newline> protein serine/threonine kinase activity ; GO:0004674 | inferred from direct assay <newline> cyclin-dependent protein serine/threonine kinase activity ; GO:0004693 | inferred from biological aspect of ancestor with PANTHER:PTN000623091 <newline> RNA polymerase II CTD [illegible]	Hsap\CDK16 <newline> Hsap\CDK20 <newline> Hsap\CDK18 <newline> Hsap\CDK17 <newline> Hsap\CDK6 <newline> Hsap\CDK10 <newline> Hsap\CDK2 <newline> Hsap\CDK7 <newline> Hsap\CDK14 <newline> Hsap\CDK3 <newline> Hsap\CDK1 <newline> Hsap\CDK5 <newline> Hsap\CDK15 <newline> Hsap\CDK4	2L

H3	mRpS7	FBgn0032236	CG5108	mitochondrial ribosomal protein S7	mRpS7	mitochondrial translation ; GO:0032543 | traceable author statement <newline> ribosomal small subunit assembly ; GO:0000028 | inferred from biological aspect of ancestor with PANTHER:PTN000128889 <newline> translation ; GO:0006412 | inferred from biological aspect of ancestor with PANTHER:PTN000128889 <newline> translation ; GO:0006412 | inferred from sequence or structural similarity with UniProtKB:Q9Y2R9 <newline> mitochondrial translation ; GO:0032543 | inferred from sequence or structural similarity with UniProtKB:Q3T040	mitochondrial small ribosomal subunit ; GO:0005763 | inferred from sequence or structural similarity with UniProtKB:Q3T040 inferred from sequence or structural similarity with UniProtKB:Q9Y2R9 <newline> mitochondrial small ribosomal subunit ; GO:0005763 | inferred from sequence or structural similarity with UniProtKB:Q9Y2R9 <newline> mitochondrial small ribosomal subunit ; GO:0005763 | inferred from biological aspect of ancestor with PANTHER:PTN000128969 <newline> mitochondrial small ribosomal subunit ; GO:0005763 | traceable author statement	mRNA binding ; GO:0003729 | inferred from biological aspect of ancestor with PANTHER:PTN000128889 <newline> structural constituent of ribosome ; GO:0003735 | inferred from sequence or structural similarity with UniProtKB:Q9Y2R9 <newline> rRNA binding ; GO:0019843 | inferred from biological aspect of ancestor with PANTHER:PTN000128889 <newline> structural constituent of ribosome ; GO:0003735 | inferred from sequence or structural similarity with UniProtKB:Q3T040 <newline> structural constituent of ribosome ; GO:0003735 | traceable author statement <newline> structural constituent of ribosome ; GO:0003735 | inferred from biological aspect of ancestor with PANTHER:PTN000128889	Hsap\RPS5 <newline> Hsap\MRPS7	2L

H3	da	FBgn0267821	CG5102	daughterless	da	protein stabilization ; GO:0050821 | inferred from direct assay inferred from mutant phenotype <newline> chaeta morphogenesis ; GO:0008407 | inferred from mutant phenotype <newline> neuroblast fate determination ; GO:0007400 | inferred from mutant phenotype <newline> positive regulation of transcription, DNA-templated ; GO:0045893 | inferred from direct assay <newline> positive regulation of transcription by RNA polymerase II ; GO:0045944 | inferred from biological aspect of ancestor with PANTHER:PTN000927455 <newline> somatic stem cell population maintenance ; GO:0035019 | inferred from mutant phenotype <newline> oogenesis ; GO:0048477 | inferred from mutant phenotype <newline> Malpighian tubule tip cell differentiation ; GO:0061382 | inferred from mutant phenotype <newline> female sex determination ; GO:0030237 | inferred from mutant phenotype [illegible]	nucleus ; GO:0005634 | inferred from direct assay <newline> transcription factor complex ; GO:0005667 | inferred from biological aspect of ancestor with PANTHER:PTN000927455 <newline> transcription factor complex ; GO:0005667 | inferred from physical interaction with l(1)sc <newline> nucleus ; GO:0005634 | inferred from biological aspect of ancestor with PANTHER:PTN000927455	sequence-specific DNA binding ; GO:0043565 | inferred from direct assay <newline> protein heterodimerization activity ; GO:0046982 | inferred from physical interaction with ac inferred from physical interaction with l(1)sc inferred from physical interaction with sc <newline> RNA polymerase II regulatory region sequence-specific DNA binding ; GO:0000977 | contributes_to inferred from physical interaction with ase inferred from physical interaction with l(1)sc inferred from physical interaction with sc <newline> DNA-binding transcription activator activity, RNA polymerase II-specific ; GO:0001228 | inferred from direct assay <newline> protein heterodimerization activity ; GO:0046982 | inferred from physical interaction with amos <newline> protein homodimerization activity ; GO:0042803 | inferred from [illegible]	Hsap\TCF4 <newline> Hsap\TCF3 <newline> Hsap\TCF12	2L
H3	CR45789	FBgn0267439	CR45789	antisense RNA:CR45789	asRNA:CR45789	-	-	-	-	2L

H3	Mdh1	FBgn0262782	CG5362	Malate dehydrogenase 1	Mdh1	carbohydrate metabolic process ; GO:0005975 | inferred from electronic annotation with InterPro:IPR015955 <newline> tricarboxylic acid cycle ; GO:0006099 | inferred from biological aspect of ancestor with PANTHER:PTN000600336 <newline> NADH metabolic process ; GO:0006734 | inferred from biological aspect of ancestor with PANTHER:PTN000600336 <newline> oxaloacetate metabolic process ; GO:0006107 | inferred from biological aspect of ancestor with PANTHER:PTN000600336 <newline> malate metabolic process ; GO:0006108 | inferred from biological aspect of ancestor with PANTHER:PTN000600336 <newline> oxidation-reduction process ; GO:0055114 | inferred from direct assay	cytosol ; GO:0005829 | inferred from direct assay <newline> cytosol ; GO:0005829 | inferred from biological aspect of ancestor with PANTHER:PTN001157397 <newline> peroxisome ; GO:0005777 | inferred from sequence model	L-malate dehydrogenase activity ; GO:0030060 | inferred from biological aspect of ancestor with PANTHER:PTN000600336 <newline> malate dehydrogenase activity ; GO:0016615 | inferred from direct assay <newline> L-malate dehydrogenase activity ; GO:0030060 | inferred from direct assay	Hsap\MDH1 <newline> Hsap\MDH1B	2L
H3	Cnot4	FBgn0051716	CG31716	CCR4-NOT transcription complex subunit 4	Cnot4	protein ubiquitination ; GO:0016567 | inferred from sequence or structural similarity with SGD:S000000870 <newline> positive regulation of receptor signaling pathway via JAK-STAT ; GO:0046427 | inferred from mutant phenotype <newline> protein ubiquitination ; GO:0016567 | inferred from biological aspect of ancestor with PANTHER:PTN000288208	CCR4-NOT complex ; GO:0030014 | inferred from biological aspect of ancestor with PANTHER:PTN000288208 <newline> cytoplasm ; GO:0005737 | inferred from sequence or structural similarity with SGD:S000000870	nucleic acid binding ; GO:0003676 | inferred from electronic annotation with InterPro:IPR000504 <newline> metal ion binding ; GO:0046872 | inferred from electronic annotation with InterPro:IPR000571 <newline> ubiquitin-protein transferase activity ; GO:0004842 | inferred from sequence or structural similarity with SGD:S000000870 <newline> ubiquitin-protein transferase activity ; GO:0004842 | inferred from biological aspect of ancestor with PANTHER:PTN000288208 <newline> zinc ion binding ; GO:0008270 | inferred from sequence model	Hsap\CNOT4	2L

H3	CG17768	FBgn0032240	CG17768	-	CG17768	nuclear-transcribed mRNA catabolic process ; GO:0000956 | inferred from electronic annotation with InterPro:IPR034101 <newline> spliceosomal snRNP assembly ; GO:0000387 | inferred from biological aspect of ancestor with PANTHER:PTN000596745 <newline> mRNA splicing, via spliceosome ; GO:0000398 | inferred from sequence or structural similarity with SGD:S000000914 <newline> cytoplasmic mRNA processing body assembly ; GO:0033962 | inferred from biological aspect of ancestor with PANTHER:PTN000596817	P-body ; GO:0000932 | inferred from biological aspect of ancestor with PANTHER:PTN000596817 <newline> U4/U6 x U5 tri-snRNP complex ; GO:0046540 | inferred from sequence or structural similarity with SGD:S000000914 <newline> U6 snRNP ; GO:0005688 | inferred from biological aspect of ancestor with PANTHER:PTN000596817	U6 snRNA binding ; GO:0017070 | inferred from biological aspect of ancestor with PANTHER:PTN000596817 <newline> U6 snRNA binding ; GO:0017070 | inferred from sequence or structural similarity with SGD:S000000914 <newline> RNA binding ; GO:0003723 | inferred from biological aspect of ancestor with PANTHER:PTN000596745	Hsap\SNRPD3 <newline> Hsap\ZNF337 <newline> Hsap\LSM4 <newline> Hsap\LSM5	2L
H3	LSm-4	FBgn0067622	CG33677	Like Sm protein 4	LSm-4	regulation of alternative mRNA splicing, via spliceosome ; GO:0000381 | inferred from mutant phenotype	-	-	Hsap\LOC101928879 <newline> Hsap\COMMD5 <newline> Hsap\COMMD9	2L
H3	Ir31a	FBgn0051718	CG31718	Ionotropic receptor 31a	Ir31a	detection of chemical stimulus involved in sensory perception ; GO:0050907 | inferred from expression pattern	membrane ; GO:0016020 | inferred from electronic annotation with InterPro:IPR001320 <newline> integral component of membrane ; GO:0016021 | inferred from sequence model	ionotropic glutamate receptor activity ; GO:0004970 | inferred from electronic annotation with InterPro:IPR001320 <newline> ligand-gated ion channel activity ; GO:0015276 | inferred from sequence model	Hsap\GRIA1 <newline> Hsap\GRIA3 <newline> Hsap\GRID1 <newline> Hsap\GRIK4 <newline> Hsap\GRIA2 <newline> Hsap\GRIA4	2L
H3	Ip259	FBgn0025366	CG5277	Intronic Protein 259	Ip259	-	preribosome, large subunit precursor ; GO:0030687 | inferred from biological aspect of ancestor with PANTHER:PTN000291059	-	Hsap\NSA2	2L

H3	RpS27A	FBgn0003942	CG5271	Ribosomal protein S27A	RpS27A	translation ; GO:0006412 | inferred from electronic annotation with InterPro:IPR002906, InterPro:IPR011332 <newline> cytoplasmic translation ; GO:0002181 | traceable author statement <newline> modification-dependent protein catabolic process ; GO:0019941 | inferred from biological aspect of ancestor with PANTHER:PTN000071508 <newline> protein ubiquitination ; GO:0016567 | inferred from biological aspect of ancestor with PANTHER:PTN000071508 <newline> protein ubiquitination ; GO:0016567 | inferred from sequence or structural similarity with SGD:S000003962	nucleus ; GO:0005634 | inferred from biological aspect of ancestor with PANTHER:PTN000071508 <newline> cytosolic small ribosomal subunit ; GO:0022627 | inferred from biological aspect of ancestor with PANTHER:PTN002241455 <newline> cytosolic small ribosomal subunit ; GO:0022627 | traceable author statement <newline> cytoplasm ; GO:0005737 | inferred from biological aspect of ancestor with PANTHER:PTN000071508	structural constituent of ribosome ; GO:0003735 | inferred from electronic annotation with InterPro:IPR002906 <newline> structural constituent of ribosome ; GO:0003735 | traceable author statement <newline> protein tag ; GO:0031386 | inferred from sequence or structural similarity with SGD:S000003962 <newline> protein tag ; GO:0031386 | inferred from biological aspect of ancestor with PANTHER:PTN000071508 <newline> ubiquitin protein ligase binding ; GO:0031625 | inferred from biological aspect of ancestor with PANTHER:PTN000071508	Hsap\UBA52 <newline> Hsap\EPS8 <newline> Hsap\ISG15 <newline> Hsap\NEDD8 <newline> Hsap\EPS8L1 <newline> Hsap\EPS8L2 <newline> Hsap\RPS27A <newline> Hsap\UBD <newline> Hsap\UBB <newline> Hsap\RXRB <newline> Hsap\EPS8L3 <newline> Hsap\UBC	2L
H3	CG5355	FBgn0032242	CG5355	-	CG5355	proteolysis ; GO:0006508 | inferred from electronic annotation with InterPro:IPR001375, InterPro:IPR002470, InterPro:IPR002471	cytoplasm ; GO:0005737 | inferred from biological aspect of ancestor with PANTHER:PTN000204594 <newline> cytosol ; GO:0005829 | inferred from biological aspect of ancestor with PANTHER:PTN000204594	serine-type endopeptidase activity ; GO:0004252 | inferred from electronic annotation with InterPro:IPR002470, InterPro:IPR002471, InterPro:IPR023302 <newline> serine-type exopeptidase activity ; GO:0070008 | inferred from electronic annotation with InterPro:IPR023302 <newline> oligopeptidase activity ; GO:0070012 | inferred from biological aspect of ancestor with PANTHER:PTN000204594 <newline> endopeptidase activity ; GO:0004175 | inferred from biological aspect of ancestor with PANTHER:PTN000204594 <newline> carboxylic ester hydrolase activity ; GO:0052689 | inferred from direct assay	Hsap\APEH <newline> Hsap\DPP4 <newline> Hsap\DPP10 <newline> Hsap\FAP <newline> Hsap\PREPL <newline> Hsap\PREP	2L

H3	Klp31E	FBgn0032243	CG5300	Kinesin-like protein at 31E	Klp31E	microtubule-based movement ; GO:0007018 | inferred from biological aspect of ancestor with PANTHER:PTN000648413	kinesin complex ; GO:0005871 | inferred from biological aspect of ancestor with PANTHER:PTN000648413 <newline> microtubule ; GO:0005874 | inferred from biological aspect of ancestor with PANTHER:PTN000648413	ATP binding ; GO:0005524 | inferred from electronic annotation with InterPro:IPR001752, InterPro:IPR019821 <newline> microtubule binding ; GO:0008017 | inferred from biological aspect of ancestor with PANTHER:PTN000648413 <newline> ATPase activity ; GO:0016887 | inferred from biological aspect of ancestor with PANTHER:PTN000648413 <newline> microtubule motor activity ; GO:0003777 | inferred from biological aspect of ancestor with PANTHER:PTN000648413	Hsap\CENPE <newline> Hsap\KIF7 <newline> Hsap\KIF3C <newline> Hsap\KIF3B <newline> Hsap\KIF27 <newline> Hsap\KIF21B <newline> Hsap\KIF21A <newline> Hsap\KIF17 <newline> Hsap\KIF4A <newline> Hsap\KIF5C <newline> Hsap\KIF15 <newline> Hsap\KIF5A <newline> Hsap\KIF5B <newline> Hsap\KIF3A <newline> Hsap\KIF4B	2L
H3	RfC3	FBgn0032244	CG5313	Replication factor C subunit 3	RfC3	leading strand elongation ; GO:0006272 | inferred from sequence or structural similarity with SGD:S000005234 <newline> sister chromatid cohesion ; GO:0007062 | inferred from sequence or structural similarity with SGD:S000005234 <newline> DNA-dependent DNA replication ; GO:0006261 | inferred from biological aspect of ancestor with PANTHER:PTN000186208	DNA replication factor C complex ; GO:0005663 | inferred from biological aspect of ancestor with PANTHER:PTN000186209 <newline> DNA replication factor C complex ; GO:0005663 | inferred from sequence or structural similarity with SGD:S000005234 <newline> Elg1 RFC-like complex ; GO:0031391 | inferred from direct assay <newline> nucleus ; GO:0005634 | inferred from biological aspect of ancestor with PANTHER:PTN000186209	ATP binding ; GO:0005524 | inferred from electronic annotation with InterPro:IPR003959 <newline> DNA clamp loader activity ; GO:0003689 | contributes_to inferred from biological aspect of ancestor with PANTHER:PTN000186209 <newline> DNA clamp loader activity ; GO:0003689 | contributes_to inferred from sequence or structural similarity with SGD:S000005234	Hsap\RFC5 <newline> Hsap\RFC4 <newline> Hsap\RFC2	2L
H3	pie	FBgn0005683	CG5354	pineapple eye	pie	germ-line stem cell division ; GO:0042078 | inferred from mutant phenotype <newline> compound eye development ; GO:0048749 | inferred from mutant phenotype <newline> intestinal stem cell homeostasis ; GO:0036335 | inferred from mutant phenotype <newline> somatic stem cell population maintenance ; GO:0035019 | inferred from mutant phenotype	-	-	Hsap\G2E3 <newline> Hsap\PHF6 <newline> Hsap\PHF11 <newline> Hsap\PHF7	2L

H3	CG5168	FBgn0032246	CG5168	WD repeat and FYVE domain containing 2	Wdfy2	-	endosome ; GO:0005768 | inferred from direct assay	metal ion binding ; GO:0046872 | inferred from electronic annotation with InterPro:IPR000306 <newline> protein kinase binding ; GO:0019901 | inferred from physical interaction with Lkb1	Hsap\WDFY2 <newline> Hsap\WDFY1	2L
H3	SmB	FBgn0262601	CG5352	Small ribonucleoprotein particle protein SmB	SmB	mRNA splicing, via spliceosome ; GO:0000398 | inferred from biological aspect of ancestor with PANTHER:PTN000075760 <newline> spermatogenesis ; GO:0007283 | inferred from mutant phenotype <newline> germ cell development ; GO:0007281 | inferred from mutant phenotype <newline> mRNA splicing, via spliceosome ; GO:0000398 | inferred by curator from GO:0071011,GO:0071013 <newline> gonad development ; GO:0008406 | inferred from mutant phenotype	U4/U6 x U5 tri-snRNP complex ; GO:0046540 | inferred from biological aspect of ancestor with PANTHER:PTN000075760 <newline> pole plasm ; GO:0045495 | inferred from direct assay <newline> catalytic step 2 spliceosome ; GO:0071013 | inferred from biological aspect of ancestor with PANTHER:PTN000075760 <newline> Cajal body ; GO:0015030 | inferred from direct assay <newline> U4 snRNP ; GO:0005687 | inferred from biological aspect of ancestor with PANTHER:PTN000075760 <newline> U5 snRNP ; GO:0005682 | inferred from biological aspect of ancestor with PANTHER:PTN000075760 <newline> U1 snRNP ; GO:0005685 | inferred from biological aspect of ancestor with PANTHER:PTN000075760 <newline> small nuclear ribonucleoprotein complex ; [illegible]	RNA binding ; GO:0003723 | inferred from sequence or structural similarity with HGNC:11153	Hsap\TAF11 <newline> Hsap\SNURF <newline> Hsap\SNRPN <newline> Hsap\SNRPB	2L

H3	KdelR	FBgn0267330	CG5183	KDEL receptor	KdelR	protein retention in ER lumen ; GO:0006621 | inferred from mutant phenotype <newline> protein retention in ER lumen ; GO:0006621 | inferred from biological aspect of ancestor with PANTHER:PTN000062353 <newline> endoplasmic reticulum to Golgi vesicle-mediated transport ; GO:0006888 | inferred from biological aspect of ancestor with PANTHER:PTN000062353	integral component of membrane ; GO:0016021 | inferred from electronic annotation with InterPro:IPR000133 <newline> cis-Golgi network ; GO:0005801 | inferred from biological aspect of ancestor with PANTHER:PTN000062353 <newline> endoplasmic reticulum ; GO:0005783 | inferred from biological aspect of ancestor with PANTHER:PTN000062353 <newline> Golgi apparatus ; GO:0005794 | inferred from biological aspect of ancestor with PANTHER:PTN000062353 <newline> endoplasmic reticulum ; GO:0005783 | inferred from sequence or structural similarity with SGD:S000000136 <newline> endoplasmic reticulum-Golgi intermediate compartment ; GO:0005793	KDEL sequence binding ; GO:0005046 | inferred from sequence or structural similarity with SGD:S000000136	Hsap\KDELR3 <newline> Hsap\KDELR1 <newline> Hsap\KDELR2	2L
H3	CG5188	FBgn0032247	CG5188	-	CG5188	proteolysis ; GO:0006508 | inferred from electronic annotation with InterPro:IPR002467	-	aminopeptidase activity ; GO:0004177 | inferred from electronic annotation with InterPro:IPR002467 <newline> metalloexopeptidase activity ; GO:0008235 | inferred from electronic annotation with InterPro:IPR002467	Hsap\METAP1 <newline> Hsap\METAP1D	2L

H3	Bug22	FBgn0032248	CG5343	Basal body up regulated gene 22	Bug22	cilium organization ; GO:0044782 | inferred from mutant phenotype <newline> mRNA splicing, via spliceosome ; GO:0000398 | inferred by curator from GO:0071011,GO:0071013 <newline> positive regulation of cell motility ; GO:2000147 | inferred from biological aspect of ancestor with PANTHER:PTN000279032 <newline> locomotion ; GO:0040011 | inferred from mutant phenotype <newline> protein polyglycylation ; GO:0018094 | inferred from mutant phenotype <newline> sperm individualization ; GO:0007291 | inferred from mutant phenotype <newline> cilium assembly ; GO:0060271 | inferred from biological aspect of ancestor with PANTHER:PTN000279032 <newline> sperm axoneme assembly ; GO:0007288 | inferred from mutant phenotype <newline> regulation of cilium beat frequency involved in ciliary motility ; GO:0060296 | inferred from [illegible]	cilium ; GO:0005929 | inferred from biological aspect of ancestor with PANTHER:PTN000279032 <newline> motile cilium ; GO:0031514 | inferred from biological aspect of ancestor with PANTHER:PTN000279032 <newline> catalytic step 2 spliceosome ; GO:0071013 | inferred from high throughput direct assay <newline> nucleolus ; GO:0005730 | inferred from direct assay <newline> nucleus ; GO:0005634 | inferred from direct assay <newline> sperm flagellum ; GO:0036126 | inferred from direct assay <newline> ciliary basal body ; GO:0036064 | inferred from biological aspect of ancestor with PANTHER:PTN000279032 <newline> cilium ; GO:0005929 | inferred from direct assay <newline> precatalytic spliceosome ; GO:0071011 | inferred from high throughput direct assay	-	Hsap\CFAP20 <newline> Hsap\C3orf67 <newline> Hsap\WDR90	2L

H3	TfIIB	FBgn0004915	CG5193	Transcription factor IIB	TfIIB	transcriptional start site selection at RNA polymerase II promoter ; GO:0001174 | inferred from biological aspect of ancestor with PANTHER:PTN000178620 <newline> DNA-templated transcription, initiation ; GO:0006352 | inferred from biological aspect of ancestor with PANTHER:PTN000178517 <newline> regulation of DNA binding ; GO:0051101 | inferred from direct assay <newline> transcription initiation from RNA polymerase II promoter ; GO:0006367 | inferred from sequence or structural similarity with HGNC:4648 inferred from sequence or structural similarity with SGD:S000006290 <newline> transcriptional start site selection at RNA polymerase II promoter ; GO:0001174 | inferred from direct assay <newline> RNA polymerase II preinitiation complex assembly ; GO:0051123 | inferred from biological aspect of ancestor with PANTHER:PTN000178621 <newline> [illegible]	nucleus ; GO:0005634 | inferred from direct assay <newline> nucleus ; GO:0005634 | inferred from biological aspect of ancestor with PANTHER:PTN000178518 <newline> transcriptional preinitiation complex ; GO:0097550 | inferred from biological aspect of ancestor with PANTHER:PTN000178620	TBP-class protein binding ; GO:0017025 | inferred from biological aspect of ancestor with PANTHER:PTN000178620 <newline> transcription factor binding ; GO:0008134 | inferred from physical interaction with Trf <newline> transcription factor binding ; GO:0008134 | inferred from biological aspect of ancestor with PANTHER:PTN000178517 <newline> RNA polymerase II complex recruiting activity ; GO:0001139 | inferred from biological aspect of ancestor with PANTHER:PTN000178620 <newline> RNA polymerase II core promoter sequence-specific DNA binding ; GO:0000979 | inferred from biological aspect of ancestor with PANTHER:PTN000178620	Hsap\GTF2B	2L
H3	TBC1D16	FBgn0032249	CG5337	TBC1 domain family member 16	TBC1D16	regulation of GTPase activity ; GO:0043087 | inferred from sequence or structural similarity with Evi5 <newline> activation of GTPase activity ; GO:0090630 | inferred from biological aspect of ancestor with PANTHER:PTN000537561 <newline> intracellular protein transport ; GO:0006886 | inferred from biological aspect of ancestor with PANTHER:PTN000537561	early endosome ; GO:0005769 | inferred from biological aspect of ancestor with PANTHER:PTN001120788	GTPase activator activity ; GO:0005096 | inferred from sequence or structural similarity with Evi5 <newline> GTPase activator activity ; GO:0005096 | inferred from biological aspect of ancestor with PANTHER:PTN000537561 <newline> Rab GTPase binding ; GO:0017137 | inferred from biological aspect of ancestor with PANTHER:PTN000537561	Hsap\TBC1D16	2L

H3	holn1	FBgn0032250	CG5198	hole-in-one	holn1	compound eye development ; GO:0048749 | inferred from mutant phenotype <newline> positive regulation of wound healing ; GO:0090303 | inferred from mutant phenotype <newline> wound healing ; GO:0042060 | inferred from mutant phenotype <newline> chaeta development ; GO:0022416 | inferred from mutant phenotype <newline> wing disc development ; GO:0035220 | inferred from mutant phenotype	U5 snRNP ; GO:0005682 | inferred from sequence or structural similarity with HGNC:1656 <newline> U5 snRNP ; GO:0005682 | inferred from biological aspect of ancestor with PANTHER:PTN000320122 <newline> nucleus ; GO:0005634 | inferred from direct assay	molecular_function ; GO:0003674 | no biological data available	Hsap\CD2BP2	2L
H3	STUB1	FBgn0027052	CG5203	STIP1 homology and U-box containing protein 1	STUB1	protein ubiquitination ; GO:0016567 | inferred from direct assay <newline> positive regulation of proteolysis ; GO:0045862 | inferred from direct assay <newline> negative regulation of insulin receptor signaling pathway ; GO:0046627 | inferred from direct assay	Z disc ; GO:0030018 | colocalizes_with inferred from direct assay	Hsp90 protein binding ; GO:0051879 | inferred from sequence or structural similarity with UniProtKB:Q9UNE7 <newline> ubiquitin protein ligase activity ; GO:0061630 | inferred from direct assay	Hsap\STUB1	2L
H3	Nse4	FBgn0032251	CG13142	Non-SMC element 4	Nse4	DNA repair ; GO:0006281 | inferred from sequence or structural similarity with SGD:S000002263 <newline> positive regulation of response to DNA damage stimulus ; GO:2001022 | inferred from biological aspect of ancestor with PANTHER:PTN000414206 <newline> DNA repair ; GO:0006281 | inferred from biological aspect of ancestor with PANTHER:PTN000414206	Smc5-Smc6 complex ; GO:0030915 | inferred from biological aspect of ancestor with PANTHER:PTN000414206 <newline> Smc5-Smc6 complex ; GO:0030915 | inferred from sequence or structural similarity with SGD:S000002263 <newline> nucleus ; GO:0005634 | inferred from sequence or structural similarity with SGD:S000002263 <newline> nucleus ; GO:0005634 | inferred from biological aspect of ancestor with PANTHER:PTN000414206	-	Hsap\NSMCE4A <newline> Hsap\EID3	2L
H3	CR45375	FBgn0266915	CR45375	long non-coding RNA:CR45375	lncRNA:CR45375	-	-	-	-	2L
H3	loh	FBgn0032252	CG6232	lonely heart	loh	positive regulation of extracellular matrix assembly ; GO:1901203 | inferred from mutant phenotype <newline> larval heart development ; GO:0007508 | inferred from mutant phenotype	collagen-containing extracellular matrix ; GO:0062023 | inferred from direct assay	peptidase activity ; GO:0008233 | inferred from electronic annotation with InterPro:IPR010909	Hsap\ADAMTSL2 <newline> Hsap\THSD4 <newline> Hsap\ADAMTSL4	2L

H3	ppk10	FBgn0065110	CG34042	pickpocket 10	ppk10	sodium ion transport ; GO:0006814 | inferred from sequence or structural similarity with MGI:MGI:101782	integral component of membrane ; GO:0016021 | inferred from sequence or structural similarity with MGI:MGI:101782	sodium channel activity ; GO:0005272 | inferred from sequence or structural similarity with MGI:MGI:101782	Hsap\ASIC1 <newline> Hsap\SCNN1A <newline> Hsap\SCNN1G <newline> Hsap\ASIC4 <newline> Hsap\ASIC3 <newline> Hsap\SCNN1D <newline> Hsap\ASIC2 <newline> Hsap\ASIC5 <newline> Hsap\SCNN1B	2L
H3	LManI	FBgn0032253	CG5322	Lysosomal alpha-mannosidase I	LManI	protein deglycosylation ; GO:0006517 | inferred from biological aspect of ancestor with PANTHER:PTN002480870 <newline> mannose metabolic process ; GO:0006013 | inferred from biological aspect of ancestor with PANTHER:PTN002480870	vacuolar membrane ; GO:0005774 | inferred from biological aspect of ancestor with PANTHER:PTN000176485	carbohydrate binding ; GO:0030246 | inferred from electronic annotation with InterPro:IPR011013 <newline> alpha-mannosidase activity ; GO:0004559 | inferred from biological aspect of ancestor with PANTHER:PTN002480870 <newline> mannosyl-oligosaccharide 1,2-alpha-mannosidase activity ; GO:0004571 | inferred from direct assay	Hsap\MAN2A1 <newline> Hsap\MAN2A2 <newline> Hsap\MAN2B1 <newline> Hsap\MAN2B2	2L
H3	LManII	FBgn0027611	CG6206	Lysosomal alpha-mannosidase II	LManII	mannose metabolic process ; GO:0006013 | inferred from biological aspect of ancestor with PANTHER:PTN002480870 <newline> protein deglycosylation ; GO:0006517 | inferred from biological aspect of ancestor with PANTHER:PTN002480870	vacuolar membrane ; GO:0005774 | inferred from biological aspect of ancestor with PANTHER:PTN000176485 <newline> extracellular region ; GO:0005576 | inferred from high throughput direct assay	carbohydrate binding ; GO:0030246 | inferred from electronic annotation with InterPro:IPR011013 <newline> alpha-mannosidase activity ; GO:0004559 | inferred from biological aspect of ancestor with PANTHER:PTN002480870 <newline> alpha-mannosidase activity ; GO:0004559 | inferred from direct assay <newline> mannosyl-oligosaccharide 1,2-alpha-mannosidase activity ; GO:0004571 | inferred from direct assay	Hsap\MAN2A2 <newline> Hsap\MAN2C1 <newline> Hsap\MAN2B2 <newline> Hsap\MAN2B1 <newline> Hsap\MAN2A1	2L
H3	RluA-1	FBgn0051719	CG31719	RluA pseudouridine synthase 1	RluA-1	enzyme-directed rRNA pseudouridine synthesis ; GO:0000455 | inferred from biological aspect of ancestor with PANTHER:PTN001311753	-	RNA binding ; GO:0003723 | inferred from electronic annotation with InterPro:IPR006145, InterPro:IPR006224, InterPro:IPR006225, InterPro:IPR020103 <newline> pseudouridine synthase activity ; GO:0009982 | inferred from biological aspect of ancestor with PANTHER:PTN001311753	Hsap\RPUSD3 <newline> Hsap\RPUSD2 <newline> Hsap\RPUSD4 <newline> Hsap\RPUSD1	2L

H3	RluA-2	FBgn0032256	CG6187	RluA pseudouridine synthase 2	RluA-2	defense response to Gram-negative bacterium ; GO:0050829 | inferred from high throughput mutant phenotype <newline> positive regulation of innate immune response ; GO:0045089 | inferred from high throughput mutant phenotype <newline> enzyme-directed rRNA pseudouridine synthesis ; GO:0000455 | inferred from biological aspect of ancestor with PANTHER:PTN001311753	-	RNA binding ; GO:0003723 | inferred from electronic annotation with InterPro:IPR002942, InterPro:IPR006145, InterPro:IPR006224, InterPro:IPR006225, InterPro:IPR020103 <newline> pseudouridine synthase activity ; GO:0009982 | inferred from biological aspect of ancestor with PANTHER:PTN001311753	Hsap\RPUSD3 <newline> Hsap\RPUSD2 <newline> Hsap\RPUSD4 <newline> Hsap\RPUSD1	2L
H3	Grip75	FBgn0026431	CG6176	Grip75	Grip75	cytoplasmic microtubule organization ; GO:0031122 | inferred from biological aspect of ancestor with PANTHER:PTN000447085 <newline> male meiosis cytokinesis ; GO:0007112 | inferred from mutant phenotype <newline> microtubule nucleation by interphase microtubule organizing center ; GO:0051415 | inferred from biological aspect of ancestor with PANTHER:PTN000447085 <newline> spindle assembly ; GO:0051225 | inferred from biological aspect of ancestor with PANTHER:PTN000447085 <newline> mitotic cell cycle ; GO:0000278 | inferred from biological aspect of ancestor with PANTHER:PTN000447085 <newline> meiotic cell cycle ; GO:0051321 | inferred from biological aspect of ancestor with PANTHER:PTN000447085 <newline> microtubule nucleation ; GO:0007020 | non-traceable author [illegible]	spindle pole ; GO:0000922 | inferred from electronic annotation with InterPro:IPR007259 <newline> centrosome ; GO:0005813 | inferred from biological aspect of ancestor with PANTHER:PTN000447085 <newline> equatorial microtubule organizing center ; GO:0000923 | inferred from biological aspect of ancestor with PANTHER:PTN000447085 <newline> gamma-tubulin ring complex ; GO:0008274 | non-traceable author statement <newline> gamma-tubulin complex ; GO:0000930 | inferred from biological aspect of ancestor with PANTHER:PTN000447085	gamma-tubulin binding ; GO:0043015 | inferred from biological aspect of ancestor with PANTHER:PTN000447085 <newline> structural constituent of cytoskeleton ; GO:0005200 | non-traceable author statement <newline> microtubule minus-end binding ; GO:0051011 | contributes_to inferred from biological aspect of ancestor with PANTHER:PTN000447085	Hsap\TUBGCP6 <newline> Hsap\TUBGCP3 <newline> Hsap\TUBGCP2 <newline> Hsap\TUBGCP5 <newline> Hsap\TUBGCP4	2L

H3	CG7456	FBgn0032258	CG7456	Component of oligomeric golgi complex 4	Cog4	Golgi organization ; GO:0007030 | inferred from biological aspect of ancestor with PANTHER:PTN000613642 <newline> Golgi organization ; GO:0007030 | inferred from sequence or structural similarity with UniProtKB:Q9H9E3 <newline> retrograde vesicle-mediated transport, Golgi to endoplasmic reticulum ; GO:0006890 | inferred from biological aspect of ancestor with PANTHER:PTN000613642 <newline> intra-Golgi vesicle-mediated transport ; GO:0006891 | inferred from sequence or structural similarity with SGD:S000006309 <newline> Golgi vesicle prefusion complex stabilization ; GO:0048213 | inferred from biological aspect of ancestor with PANTHER:PTN000613642	Golgi transport complex ; GO:0017119 | inferred from biological aspect of ancestor with PANTHER:PTN000613642 <newline> Golgi transport complex ; GO:0017119 | inferred from direct assay <newline> Golgi transport complex ; GO:0017119 | inferred from sequence or structural similarity with HGNC:18620	molecular_function ; GO:0003674 | no biological data available	Hsap\COG4	2L
H3	CG6144	FBgn0032259	CG6144	-	CG6144	oxidation-reduction process ; GO:0055114 | inferred from electronic annotation with InterPro:IPR005123	-	oxidoreductase activity ; GO:0016491 | inferred from electronic annotation with InterPro:IPR005123	Hsap\ALKBH6	2L
H3	CG13144	FBgn0032260	CG13144	-	CG13144	-	-	-	-	2L

H3	Myo31DF	FBgn0086347	CG7438	Myosin 31DF	Myo31DF	determination of left/right symmetry ; GO:0007368 | inferred from mutant phenotype <newline> determination of digestive tract left/right asymmetry ; GO:0071907 | inferred from mutant phenotype <newline> mesoderm development ; GO:0007498 | inferred from expression pattern <newline> imaginal disc-derived male genitalia morphogenesis ; GO:0048803 | inferred from mutant phenotype	myosin complex ; GO:0016459 | inferred from electronic annotation with InterPro:IPR001609, InterPro:IPR010926 <newline> cell cortex ; GO:0005938 | inferred from direct assay	ATP binding ; GO:0005524 | inferred from electronic annotation with InterPro:IPR001609 <newline> actin-dependent ATPase activity ; GO:0030898 | inferred from mutant phenotype <newline> phosphatidylinositol-4,5-bisphosphate binding ; GO:0005546 | inferred from direct assay <newline> microfilament motor activity ; GO:0000146 | inferred from direct assay	Hsap\MYO7B <newline> Hsap\MYH6 <newline> Hsap\MYO1A <newline> Hsap\MYH3 <newline> Hsap\MYH8 <newline> Hsap\MYO1C <newline> Hsap\MYH15 <newline> Hsap\MYO5B <newline> Hsap\MYH14 <newline> Hsap\MYH9 <newline> Hsap\MYO1H <newline> Hsap\MYH13 <newline> Hsap\MYH2 <newline> Hsap\MYO1E <newline> Hsap\MYH7 <newline> Hsap\MYH10 <newline> Hsap\KRTAP4-5 <newline> Hsap\OR2T33 <newline> Hsap\MYO5C <newline> Hsap\MYH4 <newline> Hsap\MYH7B <newline> Hsap\MYO1B <newline> Hsap\MYO1F <newline> Hsap\MYH1 <newline> Hsap\MYO1G <newline> Hsap\MYO1D <newline> Hsap\MYO7A	2L
H3	CR44224	FBgn0265155	CR44224	antisense RNA:CR44224	asRNA:CR44224	-	-	-	-	2L
H3	CG6094	FBgn0032261	CG6094	-	CG6094	mitochondrial translational termination ; GO:0070126 | inferred from sequence or structural similarity with UniProtKB:Q14197 <newline> mitochondrial translational termination ; GO:0070126 | inferred from biological aspect of ancestor with PANTHER:PTN000117598	mitochondrial large ribosomal subunit ; GO:0005762 | inferred from biological aspect of ancestor with PANTHER:PTN000117598 <newline> mitochondrion ; GO:0005739 | inferred from sequence or structural similarity with UniProtKB:Q14197	translation release factor activity, codon nonspecific ; GO:0016150 | inferred from sequence or structural similarity with UniProtKB:Q14197	Hsap\MRPL58	2L
H3	CG7384	FBgn0032262	CG7384	-	CG7384	-	-	-	-	2L

H3	Fatp1	FBgn0267828	CG46149	Fatty acid transport protein 1	Fatp1	triglyceride homeostasis ; GO:0070328 | inferred from mutant phenotype <newline> fatty acid biosynthetic process ; GO:0006633 | traceable author statement <newline> triglyceride homeostasis ; GO:0070328 | inferred from biological aspect of ancestor with PANTHER:PTN002875555	endoplasmic reticulum ; GO:0005783 | inferred from biological aspect of ancestor with PANTHER:PTN002875555 <newline> endomembrane system ; GO:0012505 | inferred from high throughput direct assay	long-chain fatty acid transporter activity ; GO:0005324 | inferred from biological aspect of ancestor with PANTHER:PTN001945407 <newline> long-chain fatty acid-CoA ligase activity ; GO:0004467 | inferred from biological aspect of ancestor with PANTHER:PTN001945407 <newline> very long-chain fatty acid-CoA ligase activity ; GO:0031957 | traceable author statement	Hsap\SLC27A6 <newline> Hsap\SLC27A1 <newline> Hsap\SLC27A4 <newline> Hsap\SLC27A5 <newline> Hsap\SLC27A3 <newline> Hsap\SLC27A2	2L
H3	Lrr47	FBgn0010398	CG6098	Leucine-rich repeat 47	Lrr47	Ras protein signal transduction ; GO:0007265 | inferred from sequence or structural similarity with MGI:MGI:103040	extracellular region ; GO:0005576 | inferred from sequence or structural similarity <newline> extrinsic component of membrane ; GO:0019898 | inferred from direct assay <newline> nucleus ; GO:0005634 | inferred from direct assay <newline> cytoplasm ; GO:0005737 | inferred from direct assay	-	Hsap\LRR1	2L
H3	Lip4	FBgn0032264	CG6113	Lipase 4	Lip4	cellular lipid metabolic process ; GO:0044255 | inferred from biological aspect of ancestor with PANTHER:PTN002591693	-	lipase activity ; GO:0016298 | inferred from biological aspect of ancestor with PANTHER:PTN002591693	Hsap\LIPM <newline> Hsap\LIPA <newline> Hsap\LIPJ <newline> Hsap\LIPN <newline> Hsap\LIPF <newline> Hsap\LIPK	2L
H3	CG18301	FBgn0032265	CG18301	-	CG18301	cellular lipid metabolic process ; GO:0044255 | inferred from biological aspect of ancestor with PANTHER:PTN002591693	-	lipase activity ; GO:0016298 | inferred from biological aspect of ancestor with PANTHER:PTN002591693	Hsap\LIPA <newline> Hsap\LIPN <newline> Hsap\LIPM <newline> Hsap\LIPF <newline> Hsap\LIPJ <newline> Hsap\LIPK	2L
H3	CG18302	FBgn0032266	CG18302	-	CG18302	cellular lipid metabolic process ; GO:0044255 | inferred from biological aspect of ancestor with PANTHER:PTN002591693	-	lipase activity ; GO:0016298 | inferred from biological aspect of ancestor with PANTHER:PTN002591693	Hsap\LIPK <newline> Hsap\LIPN <newline> Hsap\LIPJ <newline> Hsap\LIPA <newline> Hsap\LIPM <newline> Hsap\LIPF	2L
H3	CR44618	FBgn0265829	CR44618	long non-coding RNA:CR44618	lncRNA:CR44618	-	-	-	-	2L

H3	Trim9	FBgn0051721	CG31721	Tripartite motif containing 9	Trim9	netrin-activated signaling pathway ; GO:0038007 | inferred from mutant phenotype <newline> peripheral nervous system neuron axonogenesis ; GO:0048936 | inferred from mutant phenotype <newline> axonogenesis ; GO:0007409 | inferred from mutant phenotype <newline> axon guidance ; GO:0007411 | inferred from mutant phenotype <newline> compound eye development ; GO:0048749 | inferred from mutant phenotype inferred from genetic interaction with UniProtKB:A1Z920 <newline> axon midline choice point recognition ; GO:0016199 | inferred from mutant phenotype <newline> regulation of axonogenesis ; GO:0050770 | inferred from mutant phenotype inferred from genetic interaction with UniProtKB:A1Z920 <newline> proteasome-mediated ubiquitin-dependent protein catabolic process ; GO:0043161 | inferred from sequence or structural [illegible]	intracellular ; GO:0005622 | inferred from electronic annotation with InterPro:IPR000315, InterPro:IPR003649 <newline> axonal growth cone ; GO:0044295 | inferred from mutant phenotype inferred from genetic interaction with UniProtKB:A1Z920	zinc ion binding ; GO:0008270 | inferred from electronic annotation with InterPro:IPR000315, InterPro:IPR001841 <newline> protein binding ; GO:0005515 | inferred from physical interaction with UniProtKB:A1Z920 inferred from physical interaction with UniProtKB:Q9VWB7 <newline> ubiquitin-protein transferase activity ; GO:0004842 | inferred from sequence or structural similarity with HGNC:16288 <newline> zinc ion binding ; GO:0008270 | inferred from sequence model	Hsap\CMYA5 <newline> Hsap\TRIM36 <newline> Hsap\FSD1 <newline> Hsap\TRIM62 <newline> Hsap\TRIM10 <newline> Hsap\TRIM2 <newline> Hsap\TRIM42 <newline> Hsap\TRIM17 <newline> Hsap\TRIM59 <newline> Hsap\TRIM14 <newline> Hsap\TRIM69 <newline> Hsap\TRIM22 <newline> Hsap\TRIM5 <newline> Hsap\TRIM50 <newline> Hsap\FSD2 <newline> Hsap\TRIM60 <newline> Hsap\TRIM63 <newline> Hsap\TRIM46 <newline> Hsap\TRIM55 <newline> Hsap\TRIM67 <newline> Hsap\TRIM31 <newline> Hsap\TRIM38 <newline> Hsap\TRIM3 <newline> Hsap\SPRYD4 <newline> Hsap\TRIM25 <newline> Hsap\TRIM9 <newline> Hsap\FSD1L <newline> [illegible]	2L
H3	CG6138	FBgn0032268	CG6138	-	CG6138	-	-	-	-	2L
H3	w-cup	FBgn0032269	CG7363	world cup	w-cup	-	-	-	-	2L
H3	CG34160	FBgn0250826	CG34160	-	CG34160	biological_process ; GO:0008150 | no biological data available	cellular_component ; GO:0005575 | no biological data available	molecular_function ; GO:0003674 | no biological data available	-	2L
H3	CG34161	FBgn0085190	CG34161	-	CG34161	-	-	acylphosphatase activity ; GO:0003998 | inferred from biological aspect of ancestor with PANTHER:PTN000003030	Hsap\ACYP1 <newline> Hsap\ACYP2	2L
H3	CG7329	FBgn0032271	CG7329	-	CG7329	cellular lipid metabolic process ; GO:0044255 | inferred from biological aspect of ancestor with PANTHER:PTN002591693	-	lipase activity ; GO:0016298 | inferred from biological aspect of ancestor with PANTHER:PTN002591693	Hsap\LIPK <newline> Hsap\LIPN <newline> Hsap\LIPM <newline> Hsap\LIPJ <newline> Hsap\LIPA <newline> Hsap\LIPF	2L

H3	CG31872	FBgn0051872	CG31872	-	CG31872	lipid metabolic process ; GO:0006629 | inferred from electronic annotation with InterPro:IPR006693 <newline> multicellular organism reproduction ; GO:0032504 | inferred from high throughput expression pattern <newline> multicellular organism reproduction ; GO:0032504 | inferred from expression pattern	extracellular space ; GO:0005615 | inferred from high throughput direct assay <newline> extracellular space ; GO:0005615 | inferred from sequence model <newline> extracellular space ; GO:0005615 | inferred from direct assay	-	Hsap\LIPM <newline> Hsap\LIPF <newline> Hsap\LIPA <newline> Hsap\LIPK <newline> Hsap\LIPN <newline> Hsap\LIPJ	2L
H3	CG18284	FBgn0043825	CG18284	-	CG18284	lipid metabolic process ; GO:0006629 | inferred from electronic annotation with InterPro:IPR006693 <newline> multicellular organism reproduction ; GO:0032504 | inferred from high throughput expression pattern <newline> lipid metabolic process ; GO:0006629 | inferred from sequence or structural similarity	extracellular space ; GO:0005615 | inferred from sequence model <newline> extracellular space ; GO:0005615 | inferred from high throughput direct assay	hydrolase activity, acting on ester bonds ; GO:0016788 | inferred from electronic annotation with InterPro:IPR025483 <newline> lipase activity ; GO:0016298 | inferred from sequence or structural similarity	Hsap\LIPN <newline> Hsap\LIPK <newline> Hsap\LIPF <newline> Hsap\LIPM <newline> Hsap\LIPJ <newline> Hsap\LIPA	2L
H3	CG17097	FBgn0265264	CG17097	-	CG17097	multicellular organism reproduction ; GO:0032504 | inferred from expression pattern <newline> multicellular organism reproduction ; GO:0032504 | inferred from high throughput expression pattern <newline> lipid metabolic process ; GO:0006629 | inferred from sequence or structural similarity	extracellular space ; GO:0005615 | inferred from high throughput direct assay <newline> extracellular space ; GO:0005615 | inferred from sequence model	lipase activity ; GO:0016298 | inferred from sequence or structural similarity	Hsap\LIPJ <newline> Hsap\LIPA <newline> Hsap\LIPN <newline> Hsap\LIPK <newline> Hsap\LIPF <newline> Hsap\LIPM	2L
H3	CG17098	FBgn0032276	CG17098	-	CG17098	biological_process ; GO:0008150 | no biological data available	cellular_component ; GO:0005575 | no biological data available	zinc ion binding ; GO:0008270 | inferred from electronic annotation with InterPro:IPR003604 <newline> nucleic acid binding ; GO:0003676 | inferred from electronic annotation with InterPro:IPR003604, InterPro:IPR013087	Hsap\ZNF346 <newline> Hsap\ZNF385D <newline> Hsap\ZNF385B <newline> Hsap\ZMAT3 <newline> Hsap\ZMAT4 <newline> Hsap\ZNF385A <newline> Hsap\ZMAT1 <newline> Hsap\ZNF385C	2L

H4	polybromo	FBgn0039227	CG11375	polybromo	polybromo	vitelline membrane formation involved in chorion-containing eggshell formation ; GO:0007305 | inferred from mutant phenotype <newline> nucleosome disassembly ; GO:0006337 | inferred from biological aspect of ancestor with PANTHER:PTN000412155 <newline> ATP-dependent chromatin remodeling ; GO:0043044 | inferred from biological aspect of ancestor with PANTHER:PTN000412155 <newline> negative regulation of chromatin silencing ; GO:0031936 | inferred from genetic interaction with Pc, Bap170 <newline> negative regulation of transcription by RNA polymerase II ; GO:0000122 | inferred from mutant phenotype <newline> imaginal disc-derived leg morphogenesis ; GO:0007480 | inferred from genetic interaction with Bap170 inferred from genetic interaction with Bap170, e(y)3 inferred from genetic interaction with Bap170, e(y)3 <newline> transcription elongation from RNA	RSC-type complex ; GO:0016586 | inferred from biological aspect of ancestor with PANTHER:PTN000412155 <newline> RSC-type complex ; GO:0016586 | inferred from direct assay <newline> polytene chromosome ; GO:0005700 | inferred from direct assay <newline> brahma complex ; GO:0035060 | inferred from direct assay	chromatin binding ; GO:0003682 | inferred from biological aspect of ancestor with PANTHER:PTN000412155 <newline> DNA translocase activity ; GO:0015616 | contributes_to inferred from biological aspect of ancestor with PANTHER:PTN000412155	Hsap\PBRM1	3R
H4	CG5807	FBgn0027539	CG5807	lilipod	lili	female germ-line stem cell population maintenance ; GO:0036099 | inferred from mutant phenotype <newline> positive regulation of BMP signaling pathway ; GO:0030513 | inferred from genetic interaction with tkv inferred from genetic interaction with Dad	integral component of plasma membrane ; GO:0005887 | inferred from sequence or structural similarity with UniProtKB:Q6UX01	molecular_function ; GO:0003674 | no biological data available	Hsap\LMBR1L <newline> Hsap\LMBR1	3R
H4	CG6980	FBgn0039228	CG6980	-	CG6980	biological_process ; GO:0008150 | no biological data available	cellular_component ; GO:0005575 | no biological data available	molecular_function ; GO:0003674 | no biological data available	Hsap\TTC4 <newline> Hsap\STIP1 <newline> Hsap\SUGT1 <newline> Hsap\TTC9 <newline> Hsap\TTC28 <newline> Hsap\TTC31 <newline> Hsap\TTC9C <newline> Hsap\TTC12 <newline> Hsap\TTC9B	3R

H4	CG34150	FBgn0083986	CG34150	-	CG34150	biological_process ; GO:0008150 | no biological data available	cellular_component ; GO:0005575 | no biological data available	molecular_function ; GO:0003674 | no biological data available	Hsap\LYRM1 <newline> Hsap\SDHAF3 <newline> Hsap\HIST1H3F <newline> Hsap\C7orf55-LUC7L2 <newline> Hsap\LYRM7 <newline> Hsap\SDHAF1	3R
H4	CG5808	FBgn0027617	CG5808	-	CG5808	protein peptidyl-prolyl isomerization ; GO:0000413 | inferred from electronic annotation with InterPro:IPR002130 <newline> mRNA splicing, via spliceosome ; GO:0000398 | inferred by curator from GO:0071011,GO:0071013 <newline> regulation of phosphorylation of RNA polymerase II C-terminal domain ; GO:1901407 | inferred from biological aspect of ancestor with PANTHER:PTN000116816	catalytic step 2 spliceosome ; GO:0071013 | inferred from high throughput direct assay <newline> precatalytic spliceosome ; GO:0071011 | inferred from high throughput direct assay <newline> nucleus ; GO:0005634 | inferred from biological aspect of ancestor with PANTHER:PTN000116816	nucleic acid binding ; GO:0003676 | inferred from electronic annotation with InterPro:IPR000504 <newline> peptidyl-prolyl cis-trans isomerase activity ; GO:0003755 | inferred from electronic annotation with InterPro:IPR002130, InterPro:IPR024936 <newline> mRNA binding ; GO:0003729 | inferred from sequence or structural similarity	Hsap\RBMY1B <newline> Hsap\PPIL4 <newline> Hsap\SNRNP35 <newline> Hsap\RBMY1A1 <newline> Hsap\RBMY1E <newline> Hsap\RBMY1D	3R
H4	Saf-B	FBgn0039229	CG6995	Scaffold attachment factor B	Saf-B	regulation of transcription by RNA polymerase II ; GO:0006357 | inferred from biological aspect of ancestor with PANTHER:PTN001038533 <newline> regulation of alternative mRNA splicing, via spliceosome ; GO:0000381 | inferred from high throughput mutant phenotype <newline> regulation of mRNA processing ; GO:0050684 | inferred from biological aspect of ancestor with PANTHER:PTN001038533	nucleoplasm ; GO:0005654 | inferred from direct assay <newline> nucleus ; GO:0005634 | inferred by curator from GO:0000381 <newline> nucleus ; GO:0005634 | inferred from biological aspect of ancestor with PANTHER:PTN001038533 <newline> nuclear matrix ; GO:0016363 | inferred from direct assay	mRNA binding ; GO:0003729 | inferred from sequence or structural similarity <newline> sequence-specific DNA binding ; GO:0043565 | inferred from biological aspect of ancestor with PANTHER:PTN001038533	Hsap\TRA2A <newline> Hsap\SAFB2 <newline> Hsap\TRA2B <newline> Hsap\SAFB <newline> Hsap\SRSF2 <newline> Hsap\RBFOX2 <newline> Hsap\CSTF2 <newline> Hsap\SLTM <newline> Hsap\SRSF8 <newline> Hsap\RBMX2 <newline> Hsap\CSTF2T <newline> Hsap\RBMX	3R

H4	niki	FBgn0045980	CG10951	nimA-like kinase	niki	chromosome segregation ; GO:0007059 | inferred from biological aspect of ancestor with PANTHER:PTN000686715 <newline> protein phosphorylation ; GO:0006468 | inferred from sequence or structural similarity with Nek2	cytoplasm ; GO:0005737 | inferred from biological aspect of ancestor with PANTHER:PTN000686715 <newline> cilium ; GO:0005929 | inferred from sequence or structural similarity with HGNC:13387 <newline> centrosome ; GO:0005813 | inferred from biological aspect of ancestor with PANTHER:PTN000686715	ATP binding ; GO:0005524 | inferred from electronic annotation with InterPro:IPR000719, InterPro:IPR002290, InterPro:IPR017441 <newline> protein serine/threonine kinase activity ; GO:0004674 | inferred from sequence or structural similarity with Nek2 <newline> protein serine/threonine kinase activity ; GO:0004674 | inferred from biological aspect of ancestor with PANTHER:PTN002462863	Hsap\NEK9 <newline> Hsap\NEK1 <newline> Hsap\STKLD1 <newline> Hsap\NEK3 <newline> Hsap\NEK7 <newline> Hsap\NEK11 <newline> Hsap\NEK8 <newline> Hsap\NEK6 <newline> Hsap\NEK5 <newline> Hsap\NEK10 <newline> Hsap\NEK4 <newline> Hsap\NEK2 <newline> Hsap\MAPK15	3R
H4	CG43166	FBgn0262722	CR43166	long non-coding RNA:CR43166	lncRNA:CR43166	biological_process ; GO:0008150 | no biological data available	cellular_component ; GO:0005575 | no biological data available	molecular_function ; GO:0003674 | no biological data available	-	3R
H4	RabX4	FBgn0051118	CG31118	RabX4	RabX4	Rab protein signal transduction ; GO:0032482 | inferred from biological aspect of ancestor with PANTHER:PTN001292747 <newline> protein secretion ; GO:0009306 | inferred from biological aspect of ancestor with PANTHER:PTN000635132 <newline> regulation of exocytosis ; GO:0017157 | inferred from biological aspect of ancestor with PANTHER:PTN000635132 <newline> protein localization to plasma membrane ; GO:0072659 | inferred from biological aspect of ancestor with PANTHER:PTN000635134 <newline> cellular response to insulin stimulus ; GO:0032869 | inferred from biological aspect of ancestor with PANTHER:PTN000635544 <newline> Rab protein signal transduction ; GO:0032482 | inferred from sequence or structural similarity with Rab11 <newline> Golgi vesicle fusion to target membrane ; GO:0048210 | inferred from	trans-Golgi network transport vesicle ; GO:0030140 | inferred from biological aspect of ancestor with PANTHER:PTN000635544 <newline> recycling endosome ; GO:0055037 | inferred from high throughput direct assay <newline> plasma membrane ; GO:0005886 | inferred from biological aspect of ancestor with PANTHER:PTN000635134 <newline> endosome ; GO:0005768 | inferred from biological aspect of ancestor with PANTHER:PTN000635134 <newline> synapse ; GO:0045202 | inferred from high throughput direct assay <newline> synaptic vesicle ; GO:0008021 | inferred from biological aspect of ancestor with PANTHER:PTN001530779 <newline> neuronal cell body ; GO:0043025 | inferred from direct assay <newline> vesicle ; GO:0031982 | inferred from sequence or structural similarity with Rab11 <newline> synapse ;	GTP binding ; GO:0005525 | inferred from electronic annotation with InterPro:IPR001806, InterPro:IPR003579, InterPro:IPR005225 <newline> GTPase activity ; GO:0003924 | inferred from sequence or structural similarity with Rab11 <newline> GTPase activity ; GO:0003924 | inferred from biological aspect of ancestor with PANTHER:PTN001292747	Hsap\RAB43 <newline> Hsap\RAB30 <newline> Hsap\RAB35 <newline> Hsap\RAB8A <newline> Hsap\RAB10 <newline> Hsap\RAB19 <newline> Hsap\RAB13 <newline> Hsap\RAB1A <newline> Hsap\RAB40C <newline> Hsap\RAB40AL <newline> Hsap\RAB15 <newline> Hsap\RAB28 <newline> Hsap\RAB8B <newline> Hsap\RAB40A <newline> Hsap\RASEF <newline> Hsap\RAB40B <newline> Hsap\RAB1B <newline> Hsap\RAB12 <newline> Hsap\RAB33B	3R

H4	CG31357	FBgn0051357	CG31357	-	CG31357	negative regulation of gluconeogenesis ; GO:0045721 | inferred from electronic annotation with InterPro:IPR027714 <newline> negative regulation of apoptotic process ; GO:0043066 | inferred from biological aspect of ancestor with PANTHER:PTN000255802 <newline> ubiquitin-dependent protein catabolic process ; GO:0006511 | inferred from biological aspect of ancestor with PANTHER:PTN000255799 <newline> proteasome-mediated ubiquitin-dependent protein catabolic process ; GO:0043161 | inferred from biological aspect of ancestor with PANTHER:PTN000255799	cytoplasm ; GO:0005737 | inferred from biological aspect of ancestor with PANTHER:PTN000255799 <newline> nucleus ; GO:0005634 | inferred from biological aspect of ancestor with PANTHER:PTN000255799 <newline> GID complex ; GO:0034657 | inferred from biological aspect of ancestor with PANTHER:PTN000255799	ubiquitin-protein transferase activity ; GO:0004842 | contributes_to inferred from biological aspect of ancestor with PANTHER:PTN000255799	Hsap\MAEA	3R
H4	CG43273	FBgn0262962	CG43273	-	CG43273	biological_process ; GO:0008150 | no biological data available	cellular_component ; GO:0005575 | no biological data available	molecular_function ; GO:0003674 | no biological data available	-	3R
H4	CG13636	FBgn0039232	CG13636	sosie	sosie	ovarian follicle cell migration ; GO:0007297 | inferred from mutant phenotype <newline> germarium-derived egg chamber formation ; GO:0007293 | inferred from mutant phenotype <newline> sensory perception of sound ; GO:0007605 | inferred from mutant phenotype	apical plasma membrane ; GO:0016324 | inferred from direct assay <newline> female germline ring canal ; GO:0035324 | inferred from direct assay <newline> plasma membrane ; GO:0005886 | inferred from direct assay	-	-	3R
H4	CG33658	FBgn0053658	CG33658	-	CG33658	biological_process ; GO:0008150 | no biological data available	cellular_component ; GO:0005575 | no biological data available	molecular_function ; GO:0003674 | no biological data available	-	3R

H4	Esp	FBgn0013953	CG7005	Epidermal stripes and patches	Esp	sulfate transport ; GO:0008272 | inferred from electronic annotation with InterPro:IPR001902, InterPro:IPR011547 <newline> transmembrane transport ; GO:0055085 | inferred from electronic annotation with InterPro:IPR001902 <newline> negative regulation of female receptivity, post-mating ; GO:0045434 | inferred from mutant phenotype	integral component of plasma membrane ; GO:0005887 | inferred from biological aspect of ancestor with PANTHER:PTN000212031	secondary active sulfate transmembrane transporter activity ; GO:0008271 | inferred from electronic annotation with InterPro:IPR001902 <newline> sulfate transmembrane transporter activity ; GO:0015116 | inferred from biological aspect of ancestor with PANTHER:PTN000929279 <newline> anion:anion antiporter activity ; GO:0015301 | inferred from biological aspect of ancestor with PANTHER:PTN000212031	Hsap\SLC26A5 <newline> Hsap\SLC26A11 <newline> Hsap\SLC26A2 <newline> Hsap\SLC26A10 <newline> Hsap\SLC26A7 <newline> Hsap\SLC26A3 <newline> Hsap\SLC26A6 <newline> Hsap\SLC26A1 <newline> Hsap\SLC26A4 <newline> Hsap\SLC26A8 <newline> Hsap\SLC26A9	3R
H4	CG7006	FBgn0039233	CG7006	-	CG7006	ribosome assembly ; GO:0042255 | inferred from electronic annotation with InterPro:IPR016686 <newline> ribosomal large subunit biogenesis ; GO:0042273 | inferred from biological aspect of ancestor with PANTHER:PTN000602113	preribosome, large subunit precursor ; GO:0030687 | inferred from biological aspect of ancestor with PANTHER:PTN000602113 <newline> nucleolus ; GO:0005730 | inferred from biological aspect of ancestor with PANTHER:PTN000602113	RNA binding ; GO:0003723 | inferred from electronic annotation with InterPro:IPR002478	Hsap\NIP7	3R
H4	CG7012	FBgn0039234	CG7012	Nicastrin	Nct	Notch receptor processing ; GO:0007220 | inferred from biological aspect of ancestor with PANTHER:PTN000473192 <newline> amyloid-beta formation ; GO:0034205 | inferred from mutant phenotype <newline> Notch signaling pathway ; GO:0007219 | inferred from mutant phenotype <newline> membrane protein ectodomain proteolysis ; GO:0006509 | inferred from mutant phenotype <newline> Notch receptor processing, ligand-dependent ; GO:0035333 | inferred from genetic interaction with aph-1, pen-2, Psn <newline> amyloid-beta formation ; GO:0034205 | inferred from direct assay <newline> lateral inhibition ; GO:0046331 | inferred from mutant phenotype <newline> protein processing ; GO:0016485 | inferred from biological aspect of ancestor with PANTHER:PTN000473190 <newline> Notch receptor processing ; GO:0007220 | inferred from mutant	gamma-secretase complex ; GO:0070765 | inferred from physical interaction with aph-1, pen-2, Psn <newline> late endosome ; GO:0005770 | inferred from direct assay <newline> plasma membrane ; GO:0005886 | inferred from high throughput direct assay <newline> integral component of plasma membrane ; GO:0005887 | inferred from biological aspect of ancestor with PANTHER:PTN000473190 <newline> recycling endosome ; GO:0055037 | inferred from direct assay	-	Hsap\NCSTN	3R

H4	HDAC11	FBgn0051119	CG31119	Histone deacetylase 11	HDAC11	-	-	histone deacetylase activity ; GO:0004407 | inferred from sequence or structural similarity with UniProtKB:Q96DB2	Hsap\HDAC11 <newline> Hsap\MOCS3	3R
H4	CG10899	FBgn0039235	CG10899	Carbonic anhydrase 4	CAH4	-	-	carbonate dehydratase activity ; GO:0004089 | inferred from electronic annotation with InterPro:IPR018338 <newline> zinc ion binding ; GO:0008270 | inferred from electronic annotation with InterPro:IPR018338	Hsap\CA1 <newline> Hsap\CA9 <newline> Hsap\CA7 <newline> Hsap\CA12 <newline> Hsap\CA6 <newline> Hsap\PTPRG <newline> Hsap\CA4 <newline> Hsap\CA14 <newline> Hsap\CA3 <newline> Hsap\CA5A <newline> Hsap\CA8 <newline> Hsap\CA10 <newline> Hsap\CA13 <newline> Hsap\CA5B <newline> Hsap\CA2 <newline> Hsap\CA11	3R
H4	CG31105	FBgn0051105	CG31105	pickpocket 22	ppk22	sodium ion transport ; GO:0006814 | inferred from sequence or structural similarity with MGI:MGI:101782	integral component of membrane ; GO:0016021 | inferred from sequence or structural similarity with MGI:MGI:101782	sodium channel activity ; GO:0005272 | inferred from sequence or structural similarity with MGI:MGI:101782	Hsap\ASIC3 <newline> Hsap\ASIC4 <newline> Hsap\SCNN1B <newline> Hsap\SCNN1D <newline> Hsap\ASIC5 <newline> Hsap\ASIC1 <newline> Hsap\ASIC2 <newline> Hsap\SCNN1G <newline> Hsap\SCNN1A	3R
H4	CG13639	FBgn0265266	CG13639	-	CG13639	biological_process ; GO:0008150 | no biological data available	cellular_component ; GO:0005575 | no biological data available	molecular_function ; GO:0003674 | no biological data available	-	3R
H4	CG13640	FBgn0039237	CG13640	-	CG13640	-	-	-	-	3R
H4	CG7016	FBgn0039238	CG7016	-	CG7016	biological_process ; GO:0008150 | no biological data available	cellular_component ; GO:0005575 | no biological data available	molecular_function ; GO:0003674 | no biological data available	-	3R
H4	CG13641	FBgn0039239	CG13641	-	CG13641	-	-	-	-	3R
H4	Elal	FBgn0013949	CG7021	Elastin-like	Elal	response to nicotine ; GO:0035094 | inferred from expression pattern	cellular_component ; GO:0005575 | no biological data available	molecular_function ; GO:0003674 | no biological data available	-	3R

H4	CycB3	FBgn0015625	CG5814	Cyclin B3	CycB3	regulation of G2/M transition of mitotic cell cycle ; GO:0010389 | inferred from electronic annotation with InterPro:IPR015452 <newline> regulation of chromatin binding ; GO:0035561 | inferred from genetic interaction with Orc2 <newline> syncytial blastoderm mitotic cell cycle ; GO:0035186 | inferred from genetic interaction with CycA, CycB <newline> mitotic cell cycle ; GO:0000278 | inferred from direct assay <newline> mitotic cytokinesis ; GO:0000281 | inferred from direct assay <newline> mitotic cytokinesis ; GO:0000281 | inferred from direct assay inferred from mutant phenotype <newline> regulation of cyclin-dependent protein serine/threonine kinase activity ; GO:0000079 | inferred from biological aspect of ancestor with PANTHER:PTN000019791 <newline> mitotic cell cycle phase transition ; GO:0044772 | inferred from biological aspect of ancestor with PANTHER:PTN000019791	nucleus ; GO:0005634 | inferred from direct assay <newline> cyclin-dependent protein kinase holoenzyme complex ; GO:0000307 | inferred from biological aspect of ancestor with PANTHER:PTN000019791 <newline> nucleus ; GO:0005634 | inferred from biological aspect of ancestor with PANTHER:PTN000019791 <newline> cytoplasm ; GO:0005737 | inferred from biological aspect of ancestor with PANTHER:PTN000019791	protein kinase activity ; GO:0004672 | contributes_to inferred from biological aspect of ancestor with PANTHER:PTN000019791 <newline> protein kinase binding ; GO:0019901 | inferred from biological aspect of ancestor with PANTHER:PTN000019791 <newline> cyclin-dependent protein serine/threonine kinase regulator activity ; GO:0016538 | inferred from biological aspect of ancestor with PANTHER:PTN000019791 <newline> protein binding ; GO:0005515 | inferred from physical interaction with UniProtKB:P23572	Hsap\CNTD2 <newline> Hsap\CCNI2 <newline> Hsap\CCNQ <newline> Hsap\CCNB3 <newline> Hsap\CCNG1 <newline> Hsap\CCNB2 <newline> Hsap\CCNA1 <newline> Hsap\CCNA2 <newline> Hsap\CCNO <newline> Hsap\CCNB1 <newline> Hsap\CCNG2 <newline> Hsap\CCNI	3R
H4	CG3744	FBgn0039240	CG3744	-	CG3744	proteolysis ; GO:0006508 | inferred from electronic annotation with InterPro:IPR001375, InterPro:IPR002469	-	serine-type peptidase activity ; GO:0008236 | inferred from electronic annotation with InterPro:IPR001375	Hsap\DPP8 <newline> Hsap\DPP9	3R
H4	CG11089	FBgn0039241	CG11089	-	CG11089	wound healing ; GO:0042060 | inferred from mutant phenotype <newline> 'de novo' IMP biosynthetic process ; GO:0006189 | inferred from biological aspect of ancestor with PANTHER:PTN000190296	cytosol ; GO:0005829 | inferred from biological aspect of ancestor with PANTHER:PTN000190296	IMP cyclohydrolase activity ; GO:0003937 | inferred from biological aspect of ancestor with PANTHER:PTN000190296 <newline> phosphoribosylaminoimidazolecarboxamide formyltransferase activity ; GO:0004643 | inferred from biological aspect of ancestor with PANTHER:PTN000190296	Hsap\ATIC	3R
H4	CG31381	FBgn0043799	CG31381	-	CG31381	tRNA modification ; GO:0006400 | inferred from biological aspect of ancestor with PANTHER:PTN000119608	mitochondrion ; GO:0005739 | inferred from biological aspect of ancestor with PANTHER:PTN000119617	nucleic acid binding ; GO:0003676 | inferred from electronic annotation with InterPro:IPR013087 <newline> tRNA dimethylallyltransferase activity ; GO:0052381 | inferred from biological aspect of ancestor with PANTHER:PTN000119608	Hsap\TRIT1	3R

H4	CG31121	FBgn0051121	CG31121	-	CG31121	transmembrane transport ; GO:0055085 | inferred from biological aspect of ancestor with PANTHER:PTN000443718	plasma membrane ; GO:0005886 | inferred from biological aspect of ancestor with PANTHER:PTN000443718	ATP binding ; GO:0005524 | inferred from electronic annotation with InterPro:IPR003439 <newline> ATPase activity ; GO:0016887 | inferred from electronic annotation with InterPro:IPR003439 <newline> ATPase-coupled transmembrane transporter activity ; GO:0042626 | inferred from biological aspect of ancestor with PANTHER:PTN000443718	Hsap\ABCG8 <newline> Hsap\ABCG5 <newline> Hsap\ABCG2 <newline> Hsap\ABCG1 <newline> Hsap\ABCG4	3R
H4	CG11069	FBgn0039244	CG11069	-	CG11069	transmembrane transport ; GO:0055085 | inferred from biological aspect of ancestor with PANTHER:PTN000443718	plasma membrane ; GO:0005886 | inferred from biological aspect of ancestor with PANTHER:PTN000443718	ATP binding ; GO:0005524 | inferred from electronic annotation with InterPro:IPR003439 <newline> ATPase activity ; GO:0016887 | inferred from electronic annotation with InterPro:IPR003439 <newline> ATPase-coupled transmembrane transporter activity ; GO:0042626 | inferred from biological aspect of ancestor with PANTHER:PTN000443718	Hsap\ABCG1 <newline> Hsap\ABCG8 <newline> Hsap\ABCG5 <newline> Hsap\ABCG2 <newline> Hsap\ABCG4	3R
H4	CG13643	FBgn0040601	CG13643	-	CG13643	chitin metabolic process ; GO:0006030 | inferred from electronic annotation with InterPro:IPR002557	extracellular region ; GO:0005576 | inferred from electronic annotation with InterPro:IPR002557	chitin binding ; GO:0008061 | inferred from electronic annotation with InterPro:IPR002557	-	3R
H4	CG10845	FBgn0039246	CG10845	-	CG10845	biological_process ; GO:0008150 | no biological data available <newline> microtubule-based movement ; GO:0007018 | NOT inferred from sequence or structural similarity with MGI:MGI:2447072	kinesin complex ; GO:0005871 | inferred from sequence or structural similarity with Khc	ATPase activity ; GO:0016887 | NOT inferred from sequence or structural similarity with MGI:MGI:2447072 <newline> microtubule binding ; GO:0008017 | inferred from sequence or structural similarity with MGI:MGI:2447072 <newline> microtubule motor activity ; GO:0003777 | NOT inferred from sequence or structural similarity with MGI:MGI:2447072	Hsap\KIF3C <newline> Hsap\KIF5C <newline> Hsap\LZTFL1 <newline> Hsap\KIF3B <newline> Hsap\KIF5A <newline> Hsap\KIF5B	3R
H4	CR31382	FBgn0051382	CR31382	transfer RNA:Aspartic acid-GTC 1-12	tRNA:Asp-GTC-1-12	translation ; GO:0006412 | inferred by curator from GO:0033458	cytosol ; GO:0005829 | inferred by curator from GO:0033458	GAC codon-amino acid adaptor activity ; GO:0033458 | inferred from sequence model	-	3R

H4	CR31416	FBgn0051416	CR31416	transfer RNA:Aspartic acid-GTC 3-1	tRNA:Asp-GTC-3-1	translation ; GO:0006412 | inferred by curator from GO:0033458	cytosol ; GO:0005829 | inferred by curator from GO:0033458	GAC codon-amino acid adaptor activity ; GO:0033458 | inferred from sequence model	-	3R
H4	CG31120	FBgn0265190	CG44255	Phosphatidylinositol glycan anchor biosynthesis class S	PIG-S	attachment of GPI anchor to protein ; GO:0016255 | inferred from electronic annotation with InterPro:IPR019540 <newline> rhabdomere membrane biogenesis ; GO:0045313 | inferred from mutant phenotype <newline> GPI anchor biosynthetic process ; GO:0006506 | inferred from mutant phenotype	GPI-anchor transamidase complex ; GO:0042765 | inferred from electronic annotation with InterPro:IPR019540 <newline> nucleoplasm ; GO:0005654 | inferred from high throughput direct assay <newline> endomembrane system ; GO:0012505 | inferred from high throughput direct assay	GPI-anchor transamidase activity ; GO:0003923 | contributes_to inferred from sequence or structural similarity with MGI:MGI:2687325 <newline> peptidyl-proline 3-dioxygenase activity ; GO:0031544 | inferred from sequence or structural similarity with UniProtKB:Q8N543	Hsap\OGFOD1 <newline> Hsap\PIGS	3R
H4	CG11168	FBgn0039249	CG11168	-	CG11168	regulation of synaptic plasticity by receptor localization to synapse ; GO:1900383 | inferred from electronic annotation with InterPro:IPR033636	-	-	Hsap\CASKIN2 <newline> Hsap\ANKS1A <newline> Hsap\ANKS1B <newline> Hsap\CASKIN1	3R
H4	CG11120	FBgn0039250	CG11120	Mitotic spindle and nuclear protein	Mink	mitotic cytokinesis ; GO:0000281 | inferred from biological aspect of ancestor with PANTHER:PTN001286938 <newline> mitotic chromosome condensation ; GO:0007076 | inferred from biological aspect of ancestor with PANTHER:PTN001286938 <newline> establishment of mitotic spindle localization ; GO:0040001 | inferred from biological aspect of ancestor with PANTHER:PTN001286938	nucleus ; GO:0005634 | inferred from direct assay <newline> spindle microtubule ; GO:0005876 | colocalizes_with inferred from biological aspect of ancestor with PANTHER:PTN001286938 <newline> nucleolus ; GO:0005730 | inferred from biological aspect of ancestor with PANTHER:PTN001286938 <newline> mitotic spindle ; GO:0072686 | inferred from biological aspect of ancestor with PANTHER:PTN001286938 <newline> mitotic spindle ; GO:0072686 | inferred from direct assay	microtubule binding ; GO:0008017 | inferred from biological aspect of ancestor with PANTHER:PTN001286938 <newline> microtubule binding ; GO:0008017 | inferred from direct assay	Hsap\HMGN1 <newline> Hsap\NUSAP1	3R

H4	Trf4-2	FBgn0039251	CG17462	Topoisomerase related function 4-2	Trf4-2	histone mRNA catabolic process ; GO:0071044 | inferred from biological aspect of ancestor with PANTHER:PTN000558564 <newline> nuclear polyadenylation-dependent ncRNA catabolic process ; GO:0071046 | inferred from sequence or structural similarity with SGD:S000005243 <newline> snoRNA polyadenylation ; GO:0071050 | inferred from biological aspect of ancestor with PANTHER:PTN000558564	nucleolus ; GO:0005730 | inferred from sequence or structural similarity with SGD:S000005243 <newline> TRAMP complex ; GO:0031499 | inferred from biological aspect of ancestor with PANTHER:PTN000558564 <newline> nucleolus ; GO:0005730 | inferred from biological aspect of ancestor with PANTHER:PTN000558564	polynucleotide adenylyltransferase activity ; GO:0004652 | inferred from sequence or structural similarity with SGD:S000005243 <newline> polynucleotide adenylyltransferase activity ; GO:0004652 | inferred from biological aspect of ancestor with PANTHER:PTN000558564	Hsap\PAPD5 <newline> Hsap\PAPD4 <newline> Hsap\PAPD7	3R
H4	CG11771	FBgn0039252	CG11771	-	CG11771	proteolysis ; GO:0006508 | inferred from biological aspect of ancestor with PANTHER:PTN000211184 <newline> peptide metabolic process ; GO:0006518 | inferred from biological aspect of ancestor with PANTHER:PTN000211184	-	metalloendopeptidase activity ; GO:0004222 | inferred from biological aspect of ancestor with PANTHER:PTN000211184	Hsap\NLN <newline> Hsap\THOP1	3R
H4	Osbp	FBgn0020626	CG6708	Oxysterol binding protein	Osbp	sperm individualization ; GO:0007291 | inferred from mutant phenotype <newline> Golgi organization ; GO:0007030 | inferred from mutant phenotype <newline> intracellular protein transport ; GO:0006886 | inferred from mutant phenotype	membrane ; GO:0016020 | inferred from biological aspect of ancestor with PANTHER:PTN000104007 <newline> Golgi apparatus ; GO:0005794 | inferred from direct assay <newline> cytosol ; GO:0005829 | inferred from biological aspect of ancestor with PANTHER:PTN000104007 <newline> intracellular membrane-bounded organelle ; GO:0043231 | inferred from biological aspect of ancestor with PANTHER:PTN000104007 <newline> endoplasmic reticulum ; GO:0005783 | inferred from direct assay	lipid binding ; GO:0008289 | inferred from biological aspect of ancestor with PANTHER:PTN000104007 <newline> sterol binding ; GO:0032934 | inferred from biological aspect of ancestor with PANTHER:PTN000104007	Hsap\OSBPL3 <newline> Hsap\OSBPL7 <newline> Hsap\OSBP2 <newline> Hsap\OSBPL1A <newline> Hsap\OSBPL6 <newline> Hsap\OSBP <newline> Hsap\OSBPL2	3R

H4	ssh	FBgn0029157	CG6238	slingshot	ssh	regulation of axonogenesis ; GO:0050770 | inferred from mutant phenotype <newline> negative regulation of actin filament polymerization ; GO:0030837 | inferred from direct assay <newline> protein dephosphorylation ; GO:0006470 | non-traceable author statement <newline> regulation of axonogenesis ; GO:0050770 | inferred from genetic interaction with sick <newline> protein dephosphorylation ; GO:0006470 | inferred from mutant phenotype <newline> mitotic cell cycle ; GO:0000278 | inferred from mutant phenotype <newline> compound eye development ; GO:0048749 | inferred from genetic interaction with mbt <newline> regulation of actin polymerization or depolymerization ; GO:0008064 | inferred from mutant phenotype <newline> axonogenesis ; GO:0007409 | inferred from mutant phenotype <newline> regulation of [illegible]	apical part of cell ; GO:0045177 | inferred from direct assay	protein tyrosine phosphatase activity ; GO:0004725 | inferred from electronic annotation with InterPro:IPR016130 <newline> actin binding ; GO:0003779 | inferred from biological aspect of ancestor with PANTHER:PTN000016922 <newline> phosphoprotein phosphatase activity ; GO:0004721 | inferred from direct assay <newline> phosphoprotein phosphatase activity ; GO:0004721 | inferred from biological aspect of ancestor with PANTHER:PTN000016922 <newline> MAP kinase tyrosine/serine/threonine phosphatase activity ; GO:0017017 | NOT inferred from direct assay <newline> actin binding ; GO:0003779 | inferred from direct assay	Hsap\DUSP26 <newline> Hsap\DUSP19 <newline> Hsap\DUSP2 <newline> Hsap\SSH3 <newline> Hsap\DUSP10 <newline> Hsap\SSH2 <newline> Hsap\STYX <newline> Hsap\DUSP15 <newline> Hsap\DUSP6 <newline> Hsap\DUSP13 <newline> Hsap\DUSP18 <newline> Hsap\DUSP22 <newline> Hsap\DUSP27 <newline> Hsap\DUSP9 <newline> Hsap\DUSP28 <newline> Hsap\SSH1 <newline> Hsap\EPM2A <newline> Hsap\DUSP5 <newline> Hsap\DUSP1 <newline> Hsap\DUSP21 <newline> Hsap\DUSP14 <newline> Hsap\DUSP7 <newline> Hsap\STYXL1 <newline> Hsap\DUSP12 <newline> Hsap\DUSP8 <newline> Hsap\DUSP4 <newline> Hsap\DUPD1 <newline> [illegible]	3R
H4	Nmnat	FBgn0039254	CG13645	Nicotinamide mononucleotide adenylyltransferase	Nmnat	photoreceptor cell maintenance ; GO:0045494 | inferred from mutant phenotype <newline> dendritic spine maintenance ; GO:0097062 | inferred from mutant phenotype <newline> NAD biosynthetic process ; GO:0009435 | inferred from biological aspect of ancestor with PANTHER:PTN000247701	cytoplasm ; GO:0005737 | inferred from direct assay <newline> neuronal cell body ; GO:0043025 | inferred from direct assay	nicotinamide-nucleotide adenylyltransferase activity ; GO:0000309 | inferred from direct assay <newline> nicotinamide-nucleotide adenylyltransferase activity ; GO:0000309 | inferred from biological aspect of ancestor with PANTHER:PTN000247701 <newline> unfolded protein binding ; GO:0051082 | inferred from direct assay <newline> nicotinate-nucleotide adenylyltransferase activity ; GO:0004515 | inferred from biological aspect of ancestor with PANTHER:PTN000247701	Hsap\NMNAT3 <newline> Hsap\NMNAT2 <newline> Hsap\NMNAT1	3R

Supplementary Table 2. GO categories and their distribution in different intervals

go	name	namespace	n	HA	HB	HC	HD	LA	LB	LC	LD	LE	-Log10 (pval-H)	-Log10 (pval-L)	-Log10 (pval-H&L)
GO:0006412	translation	biological_process	521	1	1	1	1	1	1	1	1	1	6.83	8.56	15.37
GO:0003729	mRNA binding	molecular_function	179	1	1	1	0	1	1	1	1	1	5.35	10.04	14.92
GO:0016887	ATPase activity	molecular_function	152	1	1	1	1	1	1	0	0	1	8.49	5.35	13.27
GO:0005524	ATP binding	molecular_function	643	1	1	1	1	1	1	1	1	1	5.83	7.33	13.12
GO:0007476	imaginal disc-derived wing morphogenesis	biological_process	129	1	1	1	0	1	1	1	1	0	5.61	8	12.95
GO:0006355	regulation of transcription, DNA-templated	biological_process	309	1	1	1	0	1	1	1	1	1	4.37	8.96	12.3
GO:0008270	zinc ion binding	molecular_function	503	0	1	1	1	1	1	1	1	1	4.26	8.04	11.92
GO:0006470	protein dephosphorylation	biological_process	90	1	1	0	0	1	0	1	1	1	3.83	8.67	11.91
GO:0004407	histone deacetylase activity	molecular_function	14	0	1	0	1	1	1	0	0	0	6.35	5.87	11.64
GO:0006914	autophagy	biological_process	51	1	0	1	0	1	1	0	1	0	4.63	7.34	11.43
GO:0055114	oxidation-reduction process	biological_process	373	1	0	1	1	1	1	0	1	1	4.93	6.69	11.36
GO:0003723	RNA binding	molecular_function	361	0	0	1	1	1	1	1	1	1	2.67	8.54	10.41
GO:0005515	protein binding	molecular_function	858	1	1	1	1	1	1	1	1	0	5.5	4.89	10.05
GO:0030307	positive regulation of cell growth	biological_process	30	1	0	0	0	1	0	1	1	0	2.38	8.73	9.83
GO:0006508	proteolysis	biological_process	465	0	1	1	1	1	0	1	1	1	4.25	5.78	9.77
GO:0007186	G-protein coupled receptor signaling pathway	biological_process	134	1	0	0	0	1	1	1	1	0	1.75	8.88	9.67
GO:0055085	transmembrane transport	biological_process	254	1	0	0	1	1	1	0	1	1	3.12	7	9.63
GO:0004252	serine-type endopeptidase activity	molecular_function	276	0	1	1	0	1	0	1	1	1	3.04	6.87	9.44
GO:0007411	axon guidance	biological_process	183	0	1	1	0	1	1	0	1	1	2.98	7.28	9.35
GO:0005506	iron ion binding	molecular_function	149	0	0	0	1	1	1	0	1	1	1.69	8.56	9.35
GO:0000398	mRNA splicing, via spliceosome	biological_process	220	1	0	1	0	1	1	1	1	0	2.98	6.67	9.14
GO:0006506	GPI anchor biosynthetic process	biological_process	33	0	0	0	1	1	0	1	1	0	2.21	7.4	9.1
GO:0008150	biological_process	biological_process	1880	1	1	1	1	1	1	1	1	1	3.96	4.92	8.85
GO:0003676	nucleic acid binding	molecular_function	248	0	0	1	1	1	0	1	1	1	2.79	6.54	8.76
GO:0035160	maintenance of epithelial integrity, open tracheal system	biological_process	12	1	1	0	0	0	1	0	0	0	6.39	2.86	8.75
GO:0016491	oxidoreductase activity	molecular_function	97	1	0	1	0	1	1	0	1	0	3.6	5.43	8.62
GO:0007030	Golgi organization	biological_process	73	0	0	1	0	1	1	1	0	0	2.14	7.01	8.58
GO:0004930	G-protein coupled receptor activity	molecular_function	82	1	0	0	0	0	1	1	1	0	2.06	6.99	8.55
GO:0022416	chaeta development	biological_process	51	1	0	1	0	1	0	0	1	0	4.43	4.27	8.32
GO:0040011	locomotion	biological_process	31	1	0	1	0	1	1	0	0	0	4.39	4.49	8.24
GO:0045944	positive regulation of transcription from RNA polymerase II promoter	biological_process	319	1	0	0	0	1	1	1	1	1	1.09	8.97	8.2
GO:0061630	ubiquitin protein ligase activity	molecular_function	152	0	1	1	0	1	0	0	1	1	3.35	5.13	8.13
GO:0055059	asymmetric neuroblast division	biological_process	29	1	0	1	0	1	0	1	0	0	4.31	4.21	8.09
GO:0003674	molecular_function	molecular_function	3284	1	1	1	1	1	1	1	1	1	3.59	4.47	8.05
GO:0007616	long-term memory	biological_process	75	0	0	0	0	1	1	1	1	0	0	9.82	7.97
GO:0048190	wing disc dorsal/ventral pattern formation	biological_process	56	1	0	0	0	1	1	0	1	0	1.9	6.58	7.9
GO:0003735	structural constituent of ribosome	molecular_function	272	1	1	1	0	0	1	1	0	0	5.38	2.99	7.86
GO:0000976	transcription regulatory region sequence-specific DNA binding	molecular_function	76	0	0	0	0	1	1	1	1	0	0	9.91	7.84
GO:0032543	mitochondrial translation	biological_process	86	1	1	1	0	0	0	0	1	0	6.95	1.79	7.83
GO:0006814	sodium ion transport	biological_process	42	0	1	1	1	1	0	0	0	0	6.82	1.82	7.8
GO:0006099	tricarboxylic acid cycle	biological_process	38	0	0	1	0	1	0	0	1	0	2.65	5.45	7.76
GO:0042067	establishment of ommatidial planar polarity	biological_process	26	1	0	0	0	0	0	1	1	0	2.7	5.44	7.73
GO:0043565	sequence-specific DNA binding	molecular_function	241	1	0	1	0	1	0	0	1	1	3.1	4.99	7.6
GO:0007166	cell surface receptor signaling pathway	biological_process	33	0	0	0	0	0	1	1	1	0	0	8.39	7.47
GO:0016477	cell migration	biological_process	55	0	1	0	0	1	1	0	1	0	1.78	6.52	7.43

GO:0004672	protein kinase activity	molecular_function	85	0	0	1	1	1	0	1	0	0	3.98	3.76	7.42
GO:0008236	serine-type peptidase activity	molecular_function	19	0	1	0	1	1	0	0	0	0	5.44	2.41	7.41
GO:0006963	positive regulation of antibacterial peptide biosynthetic process	biological_process	26	0	1	0	0	1	0	0	1	0	2.5	5.3	7.39
GO:0000381	regulation of alternative mRNA splicing, via spliceosome	biological_process	89	0	0	1	0	0	1	1	1	0	1.76	6.18	7.36
GO:0007472	wing disc morphogenesis	biological_process	26	0	0	0	0	1	0	1	1	0	0	8.34	7.35
GO:0051015	actin filament binding	molecular_function	67	0	0	1	0	1	0	1	1	0	1.75	6.3	7.31
GO:0007283	spermatogenesis	biological_process	181	1	0	1	0	1	1	0	0	1	2.97	4.64	7.27
GO:0035220	wing disc development	biological_process	65	1	0	1	0	0	1	1	0	0	3.89	3.66	7.23
GO:0017056	structural constituent of nuclear pore	molecular_function	22	0	0	0	0	1	0	1	1	0	0	8.14	7.22
GO:0016485	protein processing	biological_process	23	0	1	0	1	0	0	1	0	0	5.29	2.36	7.18
GO:0000122	negative regulation of transcription from RNA polymerase II promoter	biological_process	222	1	1	0	0	1	0	1	0	1	2.93	4.84	7.18
GO:0007528	neuromuscular junction development	biological_process	49	0	0	0	0	1	1	0	1	0	0	8.09	7.16
GO:0008587	imaginal disc-derived wing margin morphogenesis	biological_process	43	0	0	0	0	1	1	0	1	0	0	8.35	7.15
GO:0005096	GTPase activator activity	molecular_function	82	0	1	1	0	1	0	0	1	0	3.82	3.91	7.1
GO:0006357	regulation of transcription from RNA polymerase II promoter	biological_process	290	1	0	1	0	1	1	0	1	0	2.89	4.68	7.06
GO:0003700	transcription factor activity, sequence-specific DNA binding	molecular_function	256	1	0	0	0	1	1	0	1	1	1.23	7.18	7.04
GO:0007165	signal transduction	biological_process	114	0	1	0	0	1	1	0	1	0	1.68	6.17	7.03
GO:0046872	metal ion binding	molecular_function	109	0	0	1	0	1	1	1	0	0	1.71	5.84	7.02
GO:0008188	neuropeptide receptor activity	molecular_function	44	1	0	0	0	0	1	0	1	0	2.46	4.95	7
GO:0070050	neuron cellular homeostasis	biological_process	25	1	0	0	0	0	1	0	1	0	2.37	5.13	6.99
GO:0007455	eye-antennal disc morphogenesis	biological_process	26	1	0	0	0	1	0	0	1	0	2.37	5.06	6.99
GO:0043161	proteasome-mediated ubiquitin-dependent protein catabolic process	biological_process	97	0	0	1	0	0	0	1	1	1	1.65	5.72	6.86
GO:0004842	ubiquitin-protein transferase activity	molecular_function	115	0	1	1	0	1	0	0	1	0	3.86	3.45	6.8
GO:0007293	germarium-derived egg chamber formation	biological_process	29	0	1	0	1	0	0	0	1	0	5.01	2.19	6.75
GO:0007015	actin filament organization	biological_process	73	0	0	1	0	1	1	1	0	0	1.6	5.79	6.7
GO:0007298	border follicle cell migration	biological_process	106	0	1	0	0	1	0	1	1	0	1.6	5.8	6.68
GO:0045893	positive regulation of transcription, DNA-templated	biological_process	129	0	0	0	0	1	1	0	1	1	0	8.11	6.66
GO:0003743	translation initiation factor activity	molecular_function	51	0	1	0	0	1	0	1	0	0	2.24	4.63	6.52
GO:0051321	meiotic cell cycle	biological_process	29	0	0	1	0	1	0	1	0	0	2.23	4.62	6.5
GO:0002181	cytoplasmic translation	biological_process	102	0	0	1	0	1	1	1	0	0	1.55	5.44	6.5
GO:0007391	dorsal closure	biological_process	106	1	0	0	0	0	0	1	1	1	1.55	5.66	6.5
GO:0007475	apposition of dorsal and ventral imaginal disc-derived wing surfaces	biological_process	34	0	1	0	0	1	1	0	0	0	2.19	5.36	6.47
GO:0004674	protein serine/threonine kinase activity	molecular_function	149	0	0	1	0	1	0	1	1	0	1.53	5.66	6.42
GO:0045887	positive regulation of synaptic growth at neuromuscular junction	biological_process	39	0	0	0	0	1	0	1	1	0	0	7.44	6.18
GO:0032956	regulation of actin cytoskeleton organization	biological_process	23	0	0	0	0	1	0	1	1	0	0	7.94	6.11
GO:0001700	embryonic development via the syncytial blastoderm	biological_process	49	0	0	1	0	0	0	1	1	0	2.07	4.66	6.1
GO:0007219	Notch signaling pathway	biological_process	20	0	0	0	1	1	1	0	0	0	2.07	4.54	6.08
GO:0006820	anion transport	biological_process	27	1	0	0	0	1	1	0	0	0	2.11	4.32	6.06
GO:0045089	positive regulation of innate immune response	biological_process	41	0	0	1	0	1	0	0	1	0	2.06	4.48	6.06
GO:0006030	chitin metabolic process	biological_process	98	0	0	0	1	0	1	0	1	0	2.05	4.44	6.04
GO:0006891	intra-Golgi vesicle-mediated transport	biological_process	26	0	0	1	0	0	1	0	1	0	2.09	4.3	6.03
GO:0008340	determination of adult lifespan	biological_process	169	0	0	0	0	1	1	1	0	1	0	7.7	5.98
GO:0008407	chaeta morphogenesis	biological_process	41	1	0	0	0	1	0	0	1	0	2.04	4.27	5.98
GO:0008284	positive regulation of cell proliferation	biological_process	50	0	1	0	0	1	0	0	1	0	2.03	4.32	5.98
GO:0042803	protein homodimerization activity	molecular_function	127	1	0	0	0	1	0	1	1	0	1.41	5.4	5.94
GO:0009267	cellular response to starvation	biological_process	60	0	0	0	0	1	1	0	1	0	0	7.06	5.91
GO:0035099	hemocyte migration	biological_process	20	0	1	0	0	1	0	0	1	0	2.01	4.89	5.91

GO:0017137	Rab GTPase binding	molecular_function	74	0	0	1	0	1	0	0	1	0	2.02	4.17	5.84
GO:1902093	positive regulation of flagellated sperm motility	biological_process	3	0	0	1	0	0	0	1	0	0	3.18	3	5.83
GO:0044782	cilium organization	biological_process	8	1	0	1	0	0	0	0	0	0	6.79	0	5.83
GO:0031453	positive regulation of heterochromatin assembly	biological_process	8	0	0	0	0	1	1	0	0	0	0	6.39	5.83
GO:0031032	actomyosin structure organization	biological_process	11	0	0	0	0	1	0	1	0	0	0	6.39	5.83
GO:0004176	ATP-dependent peptidase activity	molecular_function	3	0	0	1	0	1	0	0	0	0	3.2	3	5.83
GO:0008347	glial cell migration	biological_process	34	1	0	0	0	1	0	0	1	0	1.98	4.64	5.82
GO:0005272	sodium channel activity	molecular_function	33	0	1	1	1	0	0	0	0	0	7.1	0	5.78
GO:0007474	imaginal disc-derived wing vein specification	biological_process	55	0	0	0	0	1	0	0	1	1	0	7.44	5.76
GO:0000281	mitotic cytokinesis	biological_process	62	0	0	0	1	1	0	1	0	0	2	4.12	5.75
GO:0043087	regulation of GTPase activity	biological_process	53	0	1	1	0	0	0	0	1	0	4.34	1.86	5.75
GO:0043547	positive regulation of GTPase activity	biological_process	36	0	1	0	0	1	0	0	1	0	1.95	4.29	5.74
GO:0007480	imaginal disc-derived leg morphogenesis	biological_process	59	0	1	0	0	1	0	0	1	0	1.95	4.28	5.74
GO:0036335	intestinal stem cell homeostasis	biological_process	29	0	0	1	0	1	0	0	1	0	1.95	4.35	5.73
GO:0007602	phototransduction	biological_process	49	0	0	0	0	1	0	0	1	1	0	6.65	5.73
GO:0007281	germ cell development	biological_process	55	0	0	1	0	0	1	0	1	0	1.94	4.11	5.69
GO:0048592	eye morphogenesis	biological_process	8	0	0	0	0	1	0	0	0	1	0	6.36	5.68
GO:0042273	ribosomal large subunit biogenesis	biological_process	16	0	0	0	1	1	0	0	0	0	3.01	2.96	5.66
GO:0016597	amino acid binding	molecular_function	5	0	0	0	0	0	1	1	0	0	0	6.2	5.64
GO:2000737	negative regulation of stem cell differentiation	biological_process	6	0	1	0	0	1	0	0	0	0	3.07	2.89	5.62
GO:0030431	sleep	biological_process	47	0	0	0	0	1	0	1	1	0	0	6.53	5.61
GO:0004722	protein serine/threonine phosphatase activity	molecular_function	45	1	0	0	0	0	0	1	0	1	1.91	4.07	5.61
GO:0019827	stem cell population maintenance	biological_process	7	0	0	0	0	1	0	0	1	0	0	6.15	5.59
GO:0007435	salivary gland morphogenesis	biological_process	48	0	1	0	0	0	1	0	1	0	1.89	4.2	5.55
GO:0006607	NLS-bearing protein import into nucleus	biological_process	14	0	0	0	0	0	0	1	1	0	0	6.1	5.54
GO:0009060	aerobic respiration	biological_process	19	0	0	0	0	0	0	1	1	0	0	6.09	5.53
GO:0042059	negative regulation of epidermal growth factor receptor signaling pathway	biological_process	36	0	0	1	0	0	0	1	1	0	1.88	4.23	5.52
GO:0017048	Rho GTPase binding	molecular_function	11	0	0	0	0	0	0	1	1	0	0	6.08	5.52
GO:0006281	DNA repair	biological_process	67	0	0	1	0	1	0	0	1	0	1.89	3.96	5.52
GO:0050770	regulation of axonogenesis	biological_process	19	0	0	1	0	0	0	1	0	0	2.91	2.98	5.47
GO:0048477	oogenesis	biological_process	250	1	1	0	0	1	0	0	1	0	2.99	2.83	5.44
GO:0007630	jump response	biological_process	5	0	0	0	0	0	0	0	1	1	0	5.99	5.44
GO:0002168	instar larval development	biological_process	21	0	0	0	0	1	1	0	0	0	0	5.98	5.43
GO:0007414	axonal defasciculation	biological_process	5	0	1	0	0	0	1	0	0	0	2.85	2.75	5.35
GO:0010883	regulation of lipid storage	biological_process	21	1	0	0	0	0	0	0	1	0	3.07	2.76	5.35
GO:0010389	regulation of G2/M transition of mitotic cell cycle	biological_process	9	0	1	0	1	0	0	0	0	0	6.27	0	5.32
GO:0016321	female meiosis chromosome segregation	biological_process	52	0	1	0	0	0	0	1	0	0	2.82	2.74	5.28
GO:0006397	mRNA processing	biological_process	26	0	0	0	0	1	0	0	0	1	0	6.2	5.27
GO:1903688	positive regulation of border follicle cell migration	biological_process	40	1	0	0	0	1	0	0	1	0	1.79	3.93	5.24
GO:0008061	chitin binding	molecular_function	125	0	0	0	1	0	1	0	1	0	1.84	3.76	5.22
GO:0006402	mRNA catabolic process	biological_process	19	0	1	0	0	1	0	0	0	0	2.82	2.69	5.22
GO:0022857	transmembrane transporter activity	molecular_function	111	1	0	0	0	1	1	0	0	0	1.8	3.75	5.2
GO:0050829	defense response to Gram-negative bacterium	biological_process	118	0	0	1	0	1	0	0	1	0	1.79	3.73	5.18
GO:0018279	protein N-linked glycosylation via asparagine	biological_process	8	0	0	1	0	0	0	0	0	1	3.05	2.66	5.17
GO:0004579	dolichyl-diphosphooligosaccharide-protein glycotransferase activity	molecular_function	5	0	0	1	0	0	0	0	0	1	2.95	2.65	5.14
GO:0009982	pseudouridine synthase activity	molecular_function	9	0	0	1	0	1	0	0	0	0	2.73	2.66	5.11

GO:0008361	regulation of cell size	biological_process	14	0	0	0	0	1	0	0	1	0	0	5.79	5.08
GO:0042626	ATPase activity, coupled to transmembrane movement of substances	molecular_function	49	0	0	0	1	1	0	0	0	1	1.73	3.67	5.08
GO:0072321	chaperone-mediated protein transport	biological_process	5	0	0	0	0	1	0	0	1	0	0	5.84	5.03
GO:0030707	ovarian follicle cell development	biological_process	76	0	0	1	0	1	0	0	1	0	1.71	3.76	5.03
GO:0007399	nervous system development	biological_process	94	0	1	1	0	1	0	0	0	0	3.83	1.69	4.99
GO:1904801	positive regulation of neuron remodeling	biological_process	13	1	0	0	0	1	0	0	0	0	2.66	2.6	4.97
GO:0042325	regulation of phosphorylation	biological_process	7	0	0	0	0	0	0	1	1	0	0	5.51	4.95
GO:0019901	protein kinase binding	molecular_function	59	0	0	1	1	0	0	0	1	0	3.79	1.62	4.94
GO:0006644	phospholipid metabolic process	biological_process	21	1	0	0	0	0	1	0	0	0	2.66	2.55	4.94
GO:0007032	endosome organization	biological_process	12	0	0	0	0	0	0	1	1	0	0	5.48	4.93
GO:0008139	nuclear localization sequence binding	molecular_function	19	0	0	0	0	1	0	1	0	0	0	5.48	4.93
GO:0046928	regulation of neurotransmitter secretion	biological_process	14	0	0	0	0	1	1	0	0	0	0	5.48	4.93
GO:0048172	regulation of short-term neuronal synaptic plasticity	biological_process	7	0	0	0	0	1	1	0	0	0	0	5.89	4.92
GO:0048015	phosphatidylinositol-mediated signaling	biological_process	8	0	0	0	0	1	0	1	0	0	0	5.98	4.91
GO:0003824	catalytic activity	molecular_function	60	0	1	0	0	1	0	1	0	0	1.67	3.56	4.9
GO:0016575	histone deacetylation	biological_process	16	0	0	0	0	1	1	0	0	0	0	5.5	4.89
GO:0001745	compound eye morphogenesis	biological_process	119	1	0	0	0	1	0	0	1	0	1.67	3.73	4.88
GO:0017025	TBP-class protein binding	molecular_function	16	0	0	1	0	0	0	0	0	1	2.63	2.51	4.87
GO:0040003	chitin-based cuticle development	biological_process	165	0	1	1	0	1	0	0	0	0	3.89	1.56	4.86
GO:0015914	phospholipid transport	biological_process	8	0	0	0	0	1	0	1	0	0	0	5.42	4.85
GO:0006979	response to oxidative stress	biological_process	93	0	0	0	0	1	1	1	0	0	0	5.99	4.81
GO:0045752	positive regulation of Toll signaling pathway	biological_process	34	0	1	0	0	0	0	0	1	0	2.58	2.49	4.81
GO:0007431	salivary gland development	biological_process	23	0	1	0	0	0	1	0	0	0	2.57	2.61	4.79
GO:0030968	endoplasmic reticulum unfolded protein response	biological_process	18	0	0	0	0	1	0	1	0	0	0	5.35	4.78
GO:0046854	phosphatidylinositol phosphorylation	biological_process	23	0	0	0	0	1	1	0	0	0	0	5.4	4.74
GO:0034446	substrate adhesion-dependent cell spreading	biological_process	9	0	1	0	0	0	1	0	0	0	2.54	2.46	4.72
GO:0000028	ribosomal small subunit assembly	biological_process	19	0	0	1	0	0	0	1	0	0	2.65	2.43	4.71
GO:0007419	ventral cord development	biological_process	74	0	1	0	0	1	0	0	0	1	1.61	3.78	4.71
GO:0030162	regulation of proteolysis	biological_process	12	0	0	0	0	1	0	0	1	0	0	5.25	4.7
GO:0007494	midgut development	biological_process	32	0	1	0	0	0	1	0	0	0	2.51	2.48	4.68
GO:0007632	visual behavior	biological_process	18	0	0	0	0	0	0	1	1	0	0	5.25	4.68
GO:0008017	microtubule binding	molecular_function	101	0	0	1	1	0	0	1	0	0	3.62	1.56	4.68
GO:0046331	lateral inhibition	biological_process	12	0	0	0	1	1	0	0	0	0	2.52	2.41	4.67
GO:0090630	activation of GTPase activity	biological_process	29	0	0	1	0	0	0	0	1	0	2.5	2.47	4.64
GO:0000278	mitotic cell cycle	biological_process	150	0	0	1	1	0	0	1	0	0	3.59	1.49	4.63
GO:0007424	open tracheal system development	biological_process	90	1	0	0	0	1	0	0	1	0	1.58	3.57	4.63
GO:0005543	phospholipid binding	molecular_function	26	0	0	0	0	1	0	0	1	0	0	5.15	4.59
GO:0010508	positive regulation of autophagy	biological_process	29	0	1	0	0	0	0	0	1	0	2.47	2.38	4.58
GO:0051897	positive regulation of protein kinase B signaling	biological_process	9	0	0	0	0	1	0	0	1	0	0	5.4	4.58
GO:0007601	visual perception	biological_process	14	0	0	0	0	1	0	1	0	0	0	5.13	4.57
GO:0048749	compound eye development	biological_process	117	1	0	1	0	0	0	1	0	0	3.55	1.59	4.57
GO:0019904	protein domain specific binding	molecular_function	25	0	0	0	0	0	1	0	1	0	0	5.13	4.57
GO:0006869	lipid transport	biological_process	18	0	0	0	0	1	0	0	0	1	0	5.1	4.55
GO:0004185	serine-type carboxypeptidase activity	molecular_function	7	0	1	0	0	1	0	0	0	0	2.5	2.35	4.55
GO:0015450	P-P-bond-hydrolysis-driven protein transmembrane transporter activity	molecular_function	19	0	0	0	0	0	1	0	0	1	0	5.19	4.53

GO:0008594	photoreceptor cell morphogenesis	biological_process	12	1	0	0	0	1	0	0	0	0	2.43	2.36	4.52
GO:0050773	regulation of dendrite development	biological_process	16	0	0	0	0	1	0	0	1	0	0	5.21	4.51
GO:0005229	intracellular calcium activated chloride channel activity	molecular_function	8	0	0	0	0	1	0	0	0	1	0	5.53	4.51
GO:0006821	chloride transport	biological_process	17	0	0	0	0	1	0	0	0	1	0	5.53	4.51
GO:0007346	regulation of mitotic cell cycle	biological_process	48	0	0	0	0	1	0	1	0	0	0	5.25	4.5
GO:0016567	protein ubiquitination	biological_process	109	0	1	1	0	1	0	0	0	0	3.57	1.44	4.49
GO:0006886	intracellular protein transport	biological_process	152	0	1	1	0	0	0	0	1	0	3.5	1.44	4.49
GO:0016192	vesicle-mediated transport	biological_process	131	0	1	0	0	0	1	1	0	0	1.53	3.33	4.49
GO:0050768	negative regulation of neurogenesis	biological_process	7	0	0	0	0	1	0	0	1	0	0	5.59	4.49
GO:0050877	neurological system process	biological_process	25	0	0	0	0	1	0	0	1	0	0	5.01	4.45
GO:0034220	ion transmembrane transport	biological_process	26	0	0	0	0	1	0	0	1	0	0	5.01	4.45
GO:0005544	calcium-dependent phospholipid binding	molecular_function	13	0	0	0	0	0	1	1	0	0	0	4.98	4.41
GO:0046426	negative regulation of JAK-STAT cascade	biological_process	18	1	0	0	0	1	0	0	0	0	2.38	2.45	4.41
GO:0048813	dendrite morphogenesis	biological_process	132	0	0	0	0	1	0	0	1	1	0	5.64	4.4
GO:0001228	transcriptional activator activity, RNA polymerase II transcription regulatory region sequence-specific binding	molecular_function	88	1	0	0	0	1	1	0	0	0	1.5	3.41	4.39
GO:0008380	RNA splicing	biological_process	22	0	0	0	0	0	1	1	0	0	0	4.95	4.39
GO:0090303	positive regulation of wound healing	biological_process	14	0	0	1	0	0	0	1	0	0	2.37	2.36	4.39
GO:0006302	double-strand break repair	biological_process	35	0	0	0	0	1	0	1	0	0	0	4.94	4.39
GO:0006913	nucleocytoplasmic transport	biological_process	9	0	0	0	0	1	0	0	0	1	0	5.07	4.37
GO:0000387	spliceosomal snRNP assembly	biological_process	14	0	0	1	0	0	0	1	0	0	2.35	2.31	4.36
GO:0000978	RNA polymerase II core promoter proximal region sequence-specific DNA binding	molecular_function	119	1	0	0	0	1	0	0	0	1	1.48	3.39	4.32
GO:0008233	peptidase activity	molecular_function	18	0	0	1	0	0	0	0	1	0	2.33	2.57	4.32
GO:0017022	myosin binding	molecular_function	33	0	0	0	0	1	1	0	0	0	0	4.95	4.29
GO:0031532	actin cytoskeleton reorganization	biological_process	24	0	0	0	0	0	0	1	1	0	0	5.02	4.28
GO:0045792	negative regulation of cell size	biological_process	22	0	1	0	0	0	0	1	0	0	2.31	2.34	4.27
GO:0034613	cellular protein localization	biological_process	20	0	1	0	0	1	0	0	0	0	2.37	2.21	4.26
GO:0006413	translational initiation	biological_process	45	0	0	0	0	1	0	1	0	0	0	4.81	4.25
GO:0042052	rhabdomere development	biological_process	35	0	0	0	0	1	0	0	1	0	0	4.8	4.25
GO:0005319	lipid transporter activity	molecular_function	16	0	0	0	0	1	0	0	0	1	0	5.18	4.25
GO:0007173	epidermal growth factor receptor signaling pathway	biological_process	26	0	0	0	0	1	0	0	1	0	0	4.8	4.24
GO:0007422	peripheral nervous system development	biological_process	54	0	0	0	0	1	0	0	0	1	0	4.85	4.23
GO:0051603	proteolysis involved in cellular protein catabolic process	biological_process	21	0	0	1	0	1	0	0	0	0	2.35	2.2	4.23
GO:0008344	adult locomotory behavior	biological_process	61	0	0	0	0	1	0	0	1	0	0	4.73	4.17
GO:0009636	response to toxic substance	biological_process	14	0	0	0	0	1	0	0	1	0	0	4.71	4.13
GO:0051276	chromosome organization	biological_process	50	0	0	0	0	0	0	1	1	0	0	4.65	4.08
GO:0045292	mRNA cis splicing, via spliceosome	biological_process	19	1	0	0	0	0	1	0	0	0	2.26	2.12	4.08
GO:0003682	chromatin binding	molecular_function	163	0	0	0	0	1	0	1	1	0	0	4.99	4.08
GO:0072499	photoreceptor cell axon guidance	biological_process	27	1	0	0	0	0	0	0	1	0	2.24	2.12	4.07
GO:0033627	cell adhesion mediated by integrin	biological_process	21	0	1	0	0	0	1	0	0	0	2.2	2.11	4.05
GO:0003777	microtubule motor activity	molecular_function	34	0	0	1	1	0	0	0	0	0	4.87	0	4.05
GO:0034976	response to endoplasmic reticulum stress	biological_process	34	0	0	0	0	1	0	0	0	1	0	4.6	4.04
GO:0030514	negative regulation of BMP signaling pathway	biological_process	24	0	0	0	0	0	0	0	1	1	0	4.65	4.01
GO:0007520	myoblast fusion	biological_process	32	0	1	0	0	0	0	0	1	0	2.24	2.08	4.01

GO:0050830	defense response to Gram-positive bacterium	biological_process	48	0	1	0	0	0	0	0	1	0	2.17	2.07	3.98
GO:0006626	protein targeting to mitochondrion	biological_process	27	0	0	0	0	0	1	0	0	1	0	4.54	3.97
GO:0046628	positive regulation of insulin receptor signaling pathway	biological_process	17	0	1	0	0	0	0	0	1	0	2.16	2.39	3.96
GO:0007400	neuroblast fate determination	biological_process	23	0	0	0	0	1	0	0	0	1	0	4.88	3.95
GO:0007218	neuropeptide signaling pathway	biological_process	57	1	0	0	0	0	0	0	1	0	2.18	2.05	3.95
GO:0003677	DNA binding	molecular_function	435	0	0	0	0	1	1	0	1	1	0	5.29	3.94
GO:0005509	calcium ion binding	molecular_function	205	0	0	0	0	1	1	1	0	0	0	4.94	3.93
GO:0007442	hindgut morphogenesis	biological_process	17	0	0	0	0	1	0	0	1	0	0	4.6	3.91
GO:0005200	structural constituent of cytoskeleton	molecular_function	30	0	0	1	0	0	0	1	0	0	2.11	2.01	3.86
GO:0008286	insulin receptor signaling pathway	biological_process	33	0	0	0	0	1	1	0	0	0	0	4.74	3.84
GO:0042391	regulation of membrane potential	biological_process	41	0	0	0	0	1	0	0	1	0	0	4.42	3.83
GO:0044212	transcription regulatory region DNA binding	molecular_function	41	0	0	0	0	1	0	0	1	0	0	4.6	3.83
GO:0006468	protein phosphorylation	biological_process	190	0	0	1	0	1	0	1	0	0	1.31	2.97	3.82
GO:0003697	single-stranded DNA binding	molecular_function	51	0	0	0	0	1	0	0	1	0	0	4.38	3.82
GO:0045454	cell redox homeostasis	biological_process	49	0	0	0	0	0	1	1	0	0	0	4.36	3.81
GO:0030182	neuron differentiation	biological_process	34	0	0	0	0	1	0	0	1	0	0	4.35	3.79
GO:0007350	blastoderm segmentation	biological_process	23	0	0	0	0	1	0	0	1	0	0	4.41	3.79
GO:0007552	metamorphosis	biological_process	37	0	0	0	0	1	0	0	1	0	0	4.48	3.78
GO:0043022	ribosome binding	molecular_function	28	0	0	0	0	1	0	1	0	0	0	4.42	3.78
GO:0004970	ionotropic glutamate receptor activity	molecular_function	22	0	0	1	0	1	0	0	0	0	2.05	1.95	3.75
GO:0030036	actin cytoskeleton organization	biological_process	65	0	0	0	0	0	0	1	1	0	0	4.37	3.74
GO:0008157	protein phosphatase 1 binding	molecular_function	26	0	0	0	0	1	0	0	0	1	0	4.29	3.74
GO:0060090	binding, bridging	molecular_function	22	0	1	0	0	1	0	0	0	0	2.05	1.94	3.72
GO:0006338	chromatin remodeling	biological_process	55	0	0	0	0	1	0	1	0	0	0	4.27	3.72
GO:0035556	intracellular signal transduction	biological_process	92	0	1	0	0	0	0	0	1	0	2.07	1.93	3.69
GO:0030170	pyridoxal phosphate binding	molecular_function	34	0	0	0	0	0	1	0	0	1	0	4.22	3.67
GO:0048018	receptor agonist activity	molecular_function	28	0	0	0	0	1	0	0	1	0	0	4.46	3.67
GO:0015276	ligand-gated ion channel activity	molecular_function	75	0	0	1	0	1	0	0	0	0	2.01	1.92	3.66
GO:0000977	RNA polymerase II regulatory region sequence-specific DNA binding	molecular_function	166	1	0	0	0	1	1	0	0	0	1.26	2.87	3.66
GO:0031625	ubiquitin protein ligase binding	molecular_function	50	0	0	1	0	0	0	0	1	0	2	1.94	3.64
GO:0030163	protein catabolic process	biological_process	23	1	0	0	0	1	0	0	0	0	2.08	1.9	3.64
GO:0046330	positive regulation of JNK cascade	biological_process	28	0	0	0	0	1	0	0	1	0	0	4.35	3.62
GO:0006606	protein import into nucleus	biological_process	47	0	0	0	0	0	0	1	0	1	0	4.12	3.57
GO:0007443	Malpighian tubule morphogenesis	biological_process	35	0	0	1	0	0	0	0	1	0	1.96	1.88	3.57
GO:0050839	cell adhesion molecule binding	molecular_function	20	0	0	0	0	1	1	0	0	0	0	4.6	3.56
GO:0003713	transcription coactivator activity	molecular_function	63	0	0	0	0	0	1	0	1	0	0	4.11	3.56
GO:0035019	somatic stem cell population maintenance	biological_process	19	0	0	1	0	1	0	0	0	0	1.95	2.01	3.54
GO:0009416	response to light stimulus	biological_process	21	0	0	0	0	0	0	0	1	1	0	4.49	3.52
GO:0042594	response to starvation	biological_process	69	0	0	0	0	0	1	1	0	0	0	4.18	3.49
GO:0050808	synapse organization	biological_process	60	0	0	1	0	1	0	0	0	0	1.92	1.83	3.49
GO:0018105	peptidyl-serine phosphorylation	biological_process	49	0	0	0	0	1	0	1	0	0	0	4.35	3.49
GO:0006457	protein folding	biological_process	62	0	0	0	0	1	0	1	0	0	0	4.06	3.48
GO:0050832	defense response to fungus	biological_process	47	0	1	0	0	0	0	0	1	0	1.92	1.87	3.48
GO:0007349	cellularization	biological_process	41	0	0	0	0	1	0	1	0	0	0	4.01	3.46
GO:0016203	muscle attachment	biological_process	36	0	1	0	0	0	1	0	0	0	1.89	1.98	3.44
GO:0007606	sensory perception of chemical stimulus	biological_process	100	0	0	1	0	0	0	0	1	0	1.89	1.98	3.44

GO:0007274	neuromuscular synaptic transmission	biological_process	57	0	0	0	0	1	1	0	0	0	0	4.16	3.44
GO:0007444	imaginal disc development	biological_process	34	0	0	0	0	1	0	1	0	0	0	4.03	3.42
GO:0016573	histone acetylation	biological_process	54	0	0	0	0	0	1	0	1	0	0	3.94	3.39
GO:0007498	mesoderm development	biological_process	62	0	0	1	0	1	0	0	0	0	1.87	1.87	3.39
GO:0045747	positive regulation of Notch signaling pathway	biological_process	38	0	0	0	0	0	1	0	1	0	0	3.94	3.39
GO:0050804	modulation of synaptic transmission	biological_process	26	0	0	0	0	1	0	0	0	1	0	4.2	3.37
GO:0007291	sperm individualization	biological_process	54	1	0	1	0	0	0	0	0	0	4.21	0	3.37
GO:0004725	protein tyrosine phosphatase activity	molecular_function	44	0	0	0	0	0	0	1	1	0	0	4	3.37
GO:0007409	axonogenesis	biological_process	64	0	0	1	0	0	0	0	1	0	1.85	1.78	3.36
GO:0003712	transcription cofactor activity	molecular_function	60	0	1	0	0	1	0	0	0	0	1.85	1.81	3.35
GO:0045793	positive regulation of cell size	biological_process	41	0	0	0	0	1	0	0	1	0	0	4.21	3.35
GO:0004888	transmembrane signaling receptor activity	molecular_function	33	0	0	0	0	1	0	0	1	0	0	3.85	3.28
GO:0004175	endopeptidase activity	molecular_function	50	0	0	1	0	0	0	1	0	0	1.82	1.71	3.27
GO:0006511	ubiquitin-dependent protein catabolic process	biological_process	137	0	0	0	0	0	0	0	1	1	0	3.81	3.26
GO:0051082	unfolded protein binding	molecular_function	90	0	0	0	0	1	0	1	0	0	0	3.82	3.26
GO:0001751	compound eye photoreceptor cell differentiation	biological_process	31	0	0	0	0	1	0	1	0	0	0	4.48	3.25
GO:0007018	microtubule-based movement	biological_process	77	0	0	1	1	0	0	0	0	0	4.09	0	3.23
GO:0098609	cell-cell adhesion	biological_process	31	0	1	0	0	0	1	0	0	0	1.79	1.9	3.23
GO:0048010	vascular endothelial growth factor receptor signaling pathway	biological_process	30	0	0	0	0	1	0	0	1	0	0	4.13	3.23
GO:0003714	transcription corepressor activity	molecular_function	48	0	1	0	0	1	0	0	0	0	1.78	1.71	3.22
GO:0006888	ER to Golgi vesicle-mediated transport	biological_process	59	0	0	1	0	0	0	0	1	0	1.8	1.68	3.21
GO:0003779	actin binding	molecular_function	65	0	0	0	0	1	0	0	1	0	0	3.78	3.2
GO:0010628	positive regulation of gene expression	biological_process	64	0	0	0	0	1	1	0	0	0	0	3.8	3.2
GO:0005549	odorant binding	molecular_function	111	0	0	0	0	1	0	0	1	0	0	3.86	3.15
GO:0046983	protein dimerization activity	molecular_function	51	1	0	0	0	1	0	0	0	0	1.75	1.72	3.14
GO:0008083	growth factor activity	molecular_function	21	0	0	0	0	1	0	0	1	0	0	4.28	3.13
GO:0008234	cysteine-type peptidase activity	molecular_function	6	0	0	0	0	0	0	0	0	1	0	4.13	3.13
GO:0006955	immune response	biological_process	46	0	0	1	0	1	0	0	0	0	1.76	1.62	3.09
GO:0005525	GTP binding	molecular_function	155	0	1	0	0	1	0	0	0	0	1.72	1.63	3.09
GO:0000902	cell morphogenesis	biological_process	53	0	0	0	0	0	0	1	1	0	0	3.72	3.08
GO:0016705	oxidoreductase activity, acting on paired donors, with incorporation or reduction of molecular oxygen	molecular_function	85	0	0	0	0	0	0	0	1	1	0	3.68	3.07
GO:0007157	heterophilic cell-cell adhesion via plasma membrane cell adhesion molecules	biological_process	27	0	1	0	0	0	1	0	0	0	1.7	1.75	3.05
GO:0007268	chemical synaptic transmission	biological_process	95	0	0	0	0	1	0	0	1	0	0	3.72	3.03
GO:0045087	innate immune response	biological_process	59	0	1	0	0	0	0	0	1	0	1.68	1.59	3.02
GO:0007605	sensory perception of sound	biological_process	70	0	0	0	1	0	0	0	0	1	1.68	1.69	3.02
GO:0046843	dorsal appendage formation	biological_process	58	0	0	0	0	1	0	1	0	0	0	3.65	3.01
GO:0042060	wound healing	biological_process	64	0	0	1	1	0	0	0	0	0	3.76	0	2.98
GO:0007608	sensory perception of smell	biological_process	81	0	1	0	0	0	1	0	0	0	1.63	1.73	2.92
GO:0003924	GTPase activity	molecular_function	147	0	1	0	0	1	0	0	0	0	1.63	1.54	2.92
GO:0001227	transcriptional repressor activity, RNA polymerase II transcription regulatory region sequence-specific binding	molecular_function	69	0	0	0	0	1	0	0	0	1	0	3.62	2.92
GO:0006325	chromatin organization	biological_process	64	0	0	0	0	1	0	1	0	0	0	3.45	2.9
GO:0008134	transcription factor binding	molecular_function	116	0	0	1	0	0	1	0	0	0	1.6	1.54	2.86
GO:0004222	metalloendopeptidase activity	molecular_function	76	0	1	0	0	0	0	0	1	0	1.59	1.51	2.84
GO:0008586	imaginal disc-derived wing vein morphogenesis	biological_process	48	0	0	0	0	1	0	0	1	0	0	3.82	2.83

GO:1904580	regulation of intracellular mRNA localization	biological_process	3	0	0	0	0	1	0	0	0	0	0	3.83	2.82
GO:0000132	establishment of mitotic spindle orientation	biological_process	22	1	0	0	0	0	0	0	0	0	3.18	0	2.82
GO:0060271	cilium assembly	biological_process	69	0	0	1	0	1	0	0	0	0	1.59	1.49	2.82
GO:0007417	central nervous system development	biological_process	84	0	0	0	0	0	0	0	1	1	0	3.49	2.79
GO:0046982	protein heterodimerization activity	molecular_function	217	0	1	0	0	0	1	1	0	0	1.14	2.13	2.79
GO:0046856	phosphatidylinositol dephosphorylation	biological_process	13	0	0	0	0	0	0	1	0	0	0	3.03	2.77
GO:0002164	larval development	biological_process	10	0	0	0	0	1	0	0	0	0	0	3.03	2.77
GO:0035069	larval midgut histolysis	biological_process	9	0	0	0	0	0	0	0	1	0	0	3.03	2.77
GO:0046855	inositol phosphate dephosphorylation	biological_process	13	0	0	0	0	0	0	1	0	0	0	3.03	2.77
GO:0030308	negative regulation of cell growth	biological_process	13	0	0	0	0	1	0	0	0	0	0	3.03	2.77
GO:0048167	regulation of synaptic plasticity	biological_process	20	0	0	0	0	1	0	0	0	0	0	3.03	2.77
GO:0071902	positive regulation of protein serine/threonine kinase activity	biological_process	6	0	0	0	0	1	0	0	0	0	0	3.01	2.76
GO:0031098	stress-activated protein kinase signaling cascade	biological_process	17	0	0	0	0	0	0	1	0	0	0	3.01	2.76
GO:0031490	chromatin DNA binding	molecular_function	46	0	0	0	0	0	1	1	0	0	0	3.31	2.76
GO:0006490	oligosaccharide-lipid intermediate biosynthetic process	biological_process	4	0	0	1	0	0	0	0	0	0	3.22	0	2.75
GO:0007058	spindle assembly involved in female meiosis II	biological_process	4	0	0	1	0	0	0	0	0	0	3.24	0	2.75
GO:0034063	stress granule assembly	biological_process	7	0	0	0	0	0	0	0	1	0	0	3.02	2.75
GO:0007368	determination of left/right symmetry	biological_process	6	0	0	1	0	0	0	0	0	0	3.24	0	2.75
GO:0030060	L-malate dehydrogenase activity	molecular_function	4	0	0	1	0	0	0	0	0	0	3.22	0	2.75
GO:0030174	regulation of DNA-dependent DNA replication initiation	biological_process	5	0	0	1	0	0	0	0	0	0	3.22	0	2.75
GO:0007284	spermatogonial cell division	biological_process	4	0	0	1	0	0	0	0	0	0	3.22	0	2.75
GO:0017070	U6 snRNA binding	molecular_function	4	0	0	1	0	0	0	0	0	0	3.22	0	2.75
GO:0060291	long-term synaptic potentiation	biological_process	3	0	0	0	0	0	1	0	0	0	0	3.02	2.75
GO:0004177	aminopeptidase activity	molecular_function	16	0	0	1	0	0	0	0	0	0	3.18	0	2.74
GO:2000435	negative regulation of protein neddylation	biological_process	3	0	0	1	0	0	0	0	0	0	3.2	0	2.74
GO:0051101	regulation of DNA binding	biological_process	3	0	0	1	0	0	0	0	0	0	3.2	0	2.74
GO:0010592	positive regulation of lamellipodium assembly	biological_process	9	0	0	0	0	1	0	0	0	0	0	3	2.74
GO:0010468	regulation of gene expression	biological_process	116	0	0	0	0	1	1	0	0	0	0	3.29	2.74
GO:0006621	protein retention in ER lumen	biological_process	3	0	0	1	0	0	0	0	0	0	3.18	0	2.74
GO:0006515	misfolded or incompletely synthesized protein catabolic process	biological_process	4	0	0	1	0	0	0	0	0	0	3.2	0	2.74
GO:0031119	tRNA pseudouridine synthesis	biological_process	4	0	0	0	0	1	0	0	0	0	0	3.24	2.72
GO:0030720	oocyte localization involved in germarium-derived egg chamber formation	biological_process	15	0	0	0	0	1	0	0	0	0	0	2.99	2.71
GO:0042393	histone binding	molecular_function	73	0	0	0	0	1	1	0	0	0	0	3.25	2.7
GO:0097206	nephrocyte filtration	biological_process	7	0	0	0	0	0	0	0	1	0	0	2.98	2.68
GO:0006614	SRP-dependent cotranslational protein targeting to membrane	biological_process	8	0	0	0	0	1	0	0	0	0	0	2.93	2.68
GO:0020037	heme binding	molecular_function	138	0	0	0	0	0	0	0	1	1	0	3.24	2.68
GO:0046777	protein autophosphorylation	biological_process	28	0	0	0	0	0	0	0	1	0	0	2.96	2.67
GO:0001510	RNA methylation	biological_process	12	0	0	0	0	1	0	0	0	0	0	2.93	2.67
GO:0080009	mRNA methylation	biological_process	7	0	0	0	0	0	0	0	1	0	0	2.93	2.67
GO:0007509	mesoderm migration involved in gastrulation	biological_process	11	0	0	0	0	0	0	1	0	0	0	2.93	2.67
GO:0007160	cell-matrix adhesion	biological_process	14	0	1	0	0	0	0	0	0	0	3.01	0	2.66
GO:0042274	ribosomal small subunit biogenesis	biological_process	7	0	0	0	0	1	0	0	0	0	0	2.98	2.66
GO:0042562	hormone binding	molecular_function	5	0	1	0	0	0	0	0	0	0	3.43	0	2.65
GO:0006796	phosphate-containing compound metabolic process	biological_process	3	0	0	0	0	0	1	0	0	0	0	2.9	2.65
GO:0032934	sterol binding	molecular_function	13	0	0	0	0	1	0	0	0	0	0	2.89	2.64
GO:0061668	mitochondrial ribosome assembly	biological_process	6	0	0	0	0	0	1	0	0	0	0	2.89	2.64
GO:0051168	nuclear export	biological_process	7	0	0	0	0	0	0	1	0	0	0	2.9	2.64

GO:0006952	defense response	biological_process	21	0	0	0	0	1	0	0	0	0	0	2.9	2.64
GO:0051020	GTPase binding	molecular_function	7	0	0	0	0	0	0	1	0	0	0	2.88	2.63
GO:0000045	autophagosome assembly	biological_process	29	0	0	0	0	0	0	0	1	0	0	2.97	2.63
GO:0006497	protein lipidation	biological_process	6	0	0	0	0	0	0	0	1	0	0	2.97	2.63
GO:0015746	citrate transport	biological_process	5	0	0	0	0	1	0	0	0	0	0	2.99	2.62
GO:0008306	associative learning	biological_process	17	0	0	0	0	0	0	1	0	0	0	2.88	2.62
GO:0015141	succinate transmembrane transporter activity	molecular_function	10	0	0	0	0	1	0	0	0	0	0	2.99	2.62
GO:0015137	citrate transmembrane transporter activity	molecular_function	4	0	0	0	0	1	0	0	0	0	0	2.99	2.62
GO:0032515	negative regulation of phosphoprotein phosphatase activity	biological_process	5	0	0	0	0	0	0	0	0	1	0	2.88	2.62
GO:0035308	negative regulation of protein dephosphorylation	biological_process	7	0	0	0	0	0	0	0	0	1	0	2.88	2.62
GO:0016500	protein-hormone receptor activity	molecular_function	5	0	0	0	0	0	1	0	0	0	0	2.86	2.61
GO:0006665	sphingolipid metabolic process	biological_process	12	0	0	0	0	0	0	0	0	1	0	2.86	2.61
GO:0007190	activation of adenylate cyclase activity	biological_process	5	0	0	0	0	0	1	0	0	0	0	2.86	2.61
GO:0003383	apical constriction	biological_process	14	0	0	0	0	0	0	0	1	0	0	2.84	2.59
GO:0060997	dendritic spine morphogenesis	biological_process	6	0	0	0	0	0	0	0	1	0	0	2.84	2.59
GO:0006851	mitochondrial calcium ion transport	biological_process	7	0	0	0	0	1	0	0	0	0	0	2.84	2.59
GO:0051252	regulation of RNA metabolic process	biological_process	14	0	0	0	0	0	0	0	0	1	0	2.84	2.59
GO:0007019	microtubule depolymerization	biological_process	6	0	0	0	0	0	0	1	0	0	0	2.87	2.59
GO:0048027	mRNA 5'-UTR binding	molecular_function	8	0	1	0	0	0	0	0	0	0	3.05	0	2.59
GO:0043484	regulation of RNA splicing	biological_process	11	0	0	0	0	1	0	0	0	0	0	2.84	2.59
GO:0007377	germ-band extension	biological_process	11	0	0	0	0	0	0	0	0	1	0	2.84	2.59
GO:0021952	central nervous system projection neuron axonogenesis	biological_process	4	0	0	0	0	0	0	0	1	0	0	2.84	2.59
GO:0008088	axo-dendritic transport	biological_process	17	0	0	0	0	1	0	0	0	0	0	2.87	2.59
GO:0045785	positive regulation of cell adhesion	biological_process	4	0	0	0	0	0	0	1	0	0	0	2.83	2.58
GO:0030534	adult behavior	biological_process	4	0	0	0	0	1	0	0	0	0	0	2.84	2.58
GO:0035234	ectopic germ cell programmed cell death	biological_process	12	0	0	0	0	0	0	0	1	0	0	2.83	2.57
GO:0060729	intestinal epithelial structure maintenance	biological_process	8	0	0	0	0	0	0	0	1	0	0	2.83	2.57
GO:0070628	proteasome binding	molecular_function	10	0	0	0	0	0	0	1	0	0	0	2.82	2.57
GO:0004089	carbonate dehydratase activity	molecular_function	16	0	0	0	1	0	0	0	0	0	3.01	0	2.57
GO:0019752	carboxylic acid metabolic process	biological_process	7	0	0	0	0	0	0	0	0	1	0	2.83	2.57
GO:0031418	L-ascorbic acid binding	molecular_function	26	0	0	0	1	0	0	0	0	0	2.92	0	2.56
GO:1990146	protein localization to rhabdomere	biological_process	4	0	0	0	0	1	0	0	0	0	0	2.82	2.56
GO:0038001	paracrine signaling	biological_process	7	0	0	0	0	0	0	0	1	0	0	2.81	2.56
GO:0009620	response to fungus	biological_process	14	0	0	0	0	0	0	0	1	0	0	2.82	2.56
GO:0008020	G-protein coupled photoreceptor activity	molecular_function	8	0	0	0	0	1	0	0	0	0	0	2.81	2.56
GO:0008047	enzyme activator activity	molecular_function	3	0	0	0	0	0	0	0	1	0	0	2.81	2.55
GO:0016571	histone methylation	biological_process	17	0	0	0	0	0	1	0	0	0	0	2.81	2.55
GO:0050708	regulation of protein secretion	biological_process	5	0	0	1	0	0	0	0	0	0	2.9	0	2.55
GO:0043086	negative regulation of catalytic activity	biological_process	4	0	0	0	0	0	0	0	1	0	0	2.81	2.55
GO:0046833	positive regulation of RNA export from nucleus	biological_process	5	0	0	0	0	0	0	1	0	0	0	2.8	2.54
GO:0043524	negative regulation of neuron apoptotic process	biological_process	18	0	0	0	0	1	0	0	0	0	0	2.81	2.54
GO:0071038	nuclear polyadenylation-dependent tRNA catabolic process	biological_process	5	0	1	0	0	0	0	0	0	0	3.07	0	2.54
GO:0000467	exonucleolytic trimming to generate mature 3'-end of 5.8S rRNA from tricistronic rRNA transcript (SSU-rRNA, 5.8S rRNA, LSU-rRNA)	biological_process	6	0	1	0	0	0	0	0	0	0	3.07	0	2.54
GO:0071035	nuclear polyadenylation-dependent rRNA catabolic process	biological_process	5	0	1	0	0	0	0	0	0	0	3.07	0	2.54
GO:0035212	cell competition in a multicellular organism	biological_process	19	0	0	0	0	1	0	0	0	0	0	3.01	2.54
GO:0071632	optomotor response	biological_process	5	0	0	0	0	1	0	0	0	0	0	2.8	2.54

GO:0043928	exonucleolytic nuclear-transcribed mRNA catabolic process involved in deadenylation-dependent decay	biological_process	5	0	1	0	0	0	0	0	0	0	3.07	0	2.54
GO:0048085	adult chitin-containing cuticle pigmentation	biological_process	5	1	0	0	0	0	0	0	0	0	3.08	0	2.53
GO:0016831	carboxy-lyase activity	molecular_function	5	0	0	0	0	0	0	0	0	1	0	2.79	2.53
GO:1900242	regulation of synaptic vesicle endocytosis	biological_process	3	0	0	0	0	1	0	0	0	0	0	2.79	2.53
GO:0061760	antifungal innate immune response	biological_process	5	0	0	0	0	0	0	0	1	0	0	2.78	2.53
GO:0015012	heparan sulfate proteoglycan biosynthetic process	biological_process	12	0	0	0	0	1	0	0	0	0	0	2.79	2.53
GO:0010884	positive regulation of lipid storage	biological_process	11	0	0	0	0	1	0	0	0	0	0	2.91	2.53
GO:1901739	regulation of myoblast fusion	biological_process	9	0	0	0	0	0	0	1	0	0	0	2.79	2.53
GO:0014065	phosphatidylinositol 3-kinase signaling	biological_process	5	0	0	0	0	1	0	0	0	0	0	2.91	2.53
GO:0016255	attachment of GPI anchor to protein	biological_process	5	0	0	0	1	0	0	0	0	0	2.84	0	2.49
GO:0016075	rRNA catabolic process	biological_process	7	0	1	0	0	0	0	0	0	0	3.07	0	2.49
GO:0080019	fatty-acyl-CoA reductase (alcohol-forming) activity	molecular_function	17	0	0	0	0	1	0	0	0	0	0	2.87	2.49
GO:0003923	GPI-anchor transamidase activity	molecular_function	4	0	0	0	1	0	0	0	0	0	2.84	0	2.49
GO:0035336	long-chain fatty-acyl-CoA metabolic process	biological_process	17	0	0	0	0	1	0	0	0	0	0	2.87	2.49
GO:0071028	nuclear mRNA surveillance	biological_process	5	0	1	0	0	0	0	0	0	0	3.07	0	2.49
GO:2000253	positive regulation of feeding behavior	biological_process	8	0	0	0	0	0	0	0	1	0	0	2.74	2.48
GO:0006476	protein deacetylation	biological_process	3	0	0	0	0	1	0	0	0	0	0	2.74	2.48
GO:0070403	NAD+ binding	molecular_function	8	0	0	0	0	1	0	0	0	0	0	2.74	2.48
GO:2000582	positive regulation of ATP-dependent microtubule motor activity, plus-end-directed	biological_process	9	1	0	0	0	0	0	0	0	0	2.88	0	2.47
GO:0045892	negative regulation of transcription, DNA-templated	biological_process	127	0	0	0	0	1	0	0	0	1	0	3.1	2.47
GO:0043169	cation binding	molecular_function	10	1	0	0	0	0	0	0	0	0	2.81	0	2.46
GO:0008052	sensory organ boundary specification	biological_process	7	0	0	0	0	1	0	0	0	0	0	2.72	2.46
GO:0006108	malate metabolic process	biological_process	11	0	0	1	0	0	0	0	0	0	2.84	0	2.46
GO:0033314	mitotic DNA replication checkpoint	biological_process	10	0	0	0	0	0	0	1	0	0	0	2.72	2.46
GO:0009314	response to radiation	biological_process	7	0	0	0	0	0	0	1	0	0	0	2.72	2.46
GO:0033962	cytoplasmic mRNA processing body assembly	biological_process	8	0	0	1	0	0	0	0	0	0	2.88	0	2.46
GO:0018024	histone-lysine N-methyltransferase activity	molecular_function	5	0	0	0	0	0	1	0	0	0	0	2.82	2.45
GO:0016018	cyclosporin A binding	molecular_function	10	0	0	0	0	0	0	1	0	0	0	2.7	2.45
GO:0007508	larval heart development	biological_process	5	0	0	1	0	0	0	0	0	0	2.88	0	2.45
GO:0071907	determination of digestive tract left/right asymmetry	biological_process	6	0	0	1	0	0	0	0	0	0	2.89	0	2.45
GO:0005997	xylulose metabolic process	biological_process	3	0	0	0	0	0	0	0	1	0	0	2.71	2.45
GO:0019843	rRNA binding	molecular_function	17	0	0	1	0	0	0	0	0	0	2.87	0	2.45
GO:0030983	mismatched DNA binding	molecular_function	5	0	0	0	0	1	0	0	0	0	0	2.72	2.44
GO:0000245	spliceosomal complex assembly	biological_process	10	0	0	0	0	0	0	0	1	0	0	2.72	2.44
GO:0007440	foregut morphogenesis	biological_process	8	0	0	0	0	0	0	0	1	0	0	2.7	2.44
GO:0000400	four-way junction DNA binding	molecular_function	8	0	0	0	0	0	0	0	1	0	0	2.72	2.44
GO:0043539	protein serine/threonine kinase activator activity	molecular_function	8	0	0	0	0	0	0	0	1	0	0	2.68	2.43
GO:0008568	microtubule-severing ATPase activity	molecular_function	6	0	0	0	0	0	0	1	0	0	0	2.71	2.42
GO:0007095	mitotic G2 DNA damage checkpoint	biological_process	8	0	0	1	0	0	0	0	0	0	2.81	0	2.42
GO:0008187	poly-pyrimidine tract binding	molecular_function	7	0	0	0	0	0	0	1	0	0	0	2.68	2.42
GO:0007131	reciprocal meiotic recombination	biological_process	27	0	0	0	0	0	0	1	0	0	0	2.67	2.42
GO:0009058	biosynthetic process	biological_process	14	0	0	0	0	0	1	0	0	0	0	2.94	2.42
GO:0045003	double-strand break repair via synthesis-dependent strand annealing	biological_process	7	0	0	0	0	0	0	1	0	0	0	2.67	2.42
GO:0008028	monocarboxylic acid transmembrane transporter activity	molecular_function	15	0	0	0	0	0	0	0	1	0	0	2.68	2.42
GO:0051013	microtubule severing	biological_process	5	0	0	0	0	0	0	1	0	0	0	2.71	2.42

GO:0007224	smoothened signaling pathway	biological_process	14	0	0	0	0	0	0	0	1	0	0	2.68	2.41
GO:0006488	dolichol-linked oligosaccharide biosynthetic process	biological_process	6	0	0	1	0	0	0	0	0	0	3.07	0	2.41
GO:0034454	microtubule anchoring at centrosome	biological_process	5	1	0	0	0	0	0	0	0	0	2.76	0	2.41
GO:0016405	CoA-ligase activity	molecular_function	14	0	0	0	0	1	0	0	0	0	0	2.71	2.41
GO:0045873	negative regulation of sevenless signaling pathway	biological_process	5	0	0	0	0	0	0	1	0	0	0	2.67	2.41
GO:0030713	ovarian follicle cell stalk formation	biological_process	12	0	0	0	0	1	0	0	0	0	0	2.67	2.41
GO:0005126	cytokine receptor binding	molecular_function	3	0	0	0	0	1	0	0	0	0	0	2.67	2.41
GO:0007436	larval salivary gland morphogenesis	biological_process	13	0	0	0	0	1	0	0	0	0	0	2.66	2.4
GO:0034205	beta-amyloid formation	biological_process	4	0	0	0	1	0	0	0	0	0	2.76	0	2.4
GO:0034198	cellular response to amino acid starvation	biological_process	13	0	1	0	0	0	0	0	0	0	2.82	0	2.4
GO:0035313	wound healing, spreading of epidermal cells	biological_process	3	0	0	0	0	0	0	0	1	0	0	2.65	2.4
GO:0007040	lysosome organization	biological_process	11	0	0	0	0	0	0	0	1	0	0	2.69	2.4
GO:0035333	Notch receptor processing, ligand-dependent	biological_process	4	0	0	0	1	0	0	0	0	0	2.76	0	2.4
GO:0016082	synaptic vesicle priming	biological_process	16	0	0	0	0	0	1	0	0	0	0	2.69	2.4
GO:0031398	positive regulation of protein ubiquitination	biological_process	10	0	0	0	0	0	0	0	1	0	0	2.68	2.4
GO:0010841	positive regulation of circadian sleep/wake cycle, wakefulness	biological_process	5	0	0	1	0	0	0	0	0	0	2.83	0	2.39
GO:0021551	central nervous system morphogenesis	biological_process	5	0	1	0	0	0	0	0	0	0	2.8	0	2.39
GO:0005546	phosphatidylinositol-4,5-bisphosphate binding	molecular_function	15	0	0	1	0	0	0	0	0	0	2.83	0	2.39
GO:0043652	engulfment of apoptotic cell	biological_process	10	0	0	0	0	0	0	1	0	0	0	2.64	2.39
GO:0040014	regulation of multicellular organism growth	biological_process	17	0	0	0	0	1	0	0	0	0	0	2.64	2.39
GO:0007010	cytoskeleton organization	biological_process	20	0	0	0	0	0	0	1	0	0	0	2.63	2.38
GO:0050833	pyruvate transmembrane transporter activity	molecular_function	6	0	0	0	0	1	0	0	0	0	0	2.64	2.38
GO:0070373	negative regulation of ERK1 and ERK2 cascade	biological_process	17	0	0	0	0	0	0	1	0	0	0	2.63	2.38
GO:0002230	positive regulation of defense response to virus by host	biological_process	12	0	0	0	0	1	0	0	0	0	0	2.7	2.38
GO:0004865	protein serine/threonine phosphatase inhibitor activity	molecular_function	4	0	0	0	0	0	0	0	0	1	0	2.64	2.38
GO:0072697	protein localization to cell cortex	biological_process	6	0	0	0	0	1	0	0	0	0	0	2.69	2.37
GO:0006744	ubiquinone biosynthetic process	biological_process	10	0	0	0	0	0	1	0	0	0	0	2.62	2.37
GO:0061643	chemorepulsion of axon	biological_process	3	0	0	0	0	0	0	0	1	0	0	2.79	2.37
GO:0000184	nuclear-transcribed mRNA catabolic process, nonsense-mediated decay	biological_process	20	1	0	0	0	0	0	0	0	0	2.87	0	2.36
GO:0042058	regulation of epidermal growth factor receptor signaling pathway	biological_process	6	0	0	1	0	0	0	0	0	0	2.72	0	2.36
GO:0006405	RNA export from nucleus	biological_process	15	0	0	0	0	0	0	1	0	0	0	2.6	2.35
GO:0007612	learning	biological_process	14	0	0	0	0	0	0	0	1	0	0	2.66	2.35
GO:0000166	nucleotide binding	molecular_function	16	0	1	0	0	0	0	0	0	0	2.79	0	2.35
GO:0032968	positive regulation of transcription elongation from RNA polymerase II promoter	biological_process	19	0	0	0	0	1	0	0	0	0	0	2.61	2.35
GO:0045039	protein import into mitochondrial inner membrane	biological_process	8	0	0	0	0	0	0	0	1	0	0	2.72	2.34
GO:0040034	regulation of development, heterochronic	biological_process	13	0	0	0	0	1	0	0	0	0	0	2.64	2.34
GO:0000012	single strand break repair	biological_process	4	0	0	0	0	1	0	0	0	0	0	2.67	2.34
GO:0003684	damaged DNA binding	molecular_function	26	0	0	0	0	1	0	0	0	0	0	2.63	2.33
GO:0007265	Ras protein signal transduction	biological_process	13	0	0	1	0	0	0	0	0	0	2.77	0	2.33
GO:0008528	G-protein coupled peptide receptor activity	molecular_function	15	0	0	0	0	0	1	0	0	0	0	2.58	2.33
GO:0015301	anion:anion antiporter activity	molecular_function	10	0	0	0	1	0	0	0	0	0	2.68	0	2.33
GO:0051028	mRNA transport	biological_process	12	1	0	0	0	0	0	0	0	0	2.73	0	2.32
GO:0007309	oocyte axis specification	biological_process	9	0	0	0	0	0	0	1	0	0	0	2.58	2.32
GO:0051604	protein maturation	biological_process	5	0	0	0	0	0	0	0	1	0	0	2.57	2.31
GO:0002807	positive regulation of antimicrobial peptide biosynthetic process	biological_process	6	0	0	0	0	0	0	0	0	1	0	2.57	2.31
GO:0006189	'de novo' IMP biosynthetic process	biological_process	4	0	0	0	1	0	0	0	0	0	2.75	0	2.3

GO:0001932	regulation of protein phosphorylation	biological_process	7	0	0	0	0	0	0	1	0	0	0	2.63	2.3
GO:0000079	regulation of cyclin-dependent protein serine/threonine kinase activity	biological_process	14	0	0	0	1	0	0	0	0	0	2.65	0	2.3
GO:0000956	nuclear-transcribed mRNA catabolic process	biological_process	7	0	0	1	0	0	0	0	0	0	2.74	0	2.3
GO:0044772	mitotic cell cycle phase transition	biological_process	7	0	0	0	1	0	0	0	0	0	2.65	0	2.3
GO:0008543	fibroblast growth factor receptor signaling pathway	biological_process	22	0	0	0	0	1	0	0	0	0	0	2.55	2.3
GO:0008595	anterior/posterior axis specification, embryo	biological_process	18	0	0	0	0	1	0	0	0	0	0	2.67	2.3
GO:0008121	ubiquinol-cytochrome-c reductase activity	molecular_function	12	0	0	0	0	0	0	1	0	0	0	2.76	2.29
GO:0043248	proteasome assembly	biological_process	12	0	0	0	0	0	1	0	0	0	0	2.55	2.29
GO:0045727	positive regulation of translation	biological_process	19	0	0	0	0	1	0	0	0	0	0	2.55	2.29
GO:0008353	RNA polymerase II carboxy-terminal domain kinase activity	molecular_function	11	0	0	1	0	0	0	0	0	0	2.71	0	2.29
GO:0060079	excitatory postsynaptic potential	biological_process	3	0	0	0	0	0	0	0	1	0	0	2.75	2.29
GO:0046667	compound eye retinal cell programmed cell death	biological_process	9	0	0	0	0	1	0	0	0	0	0	2.66	2.29
GO:0060074	synapse maturation	biological_process	6	0	0	0	0	1	0	0	0	0	0	2.54	2.29
GO:0033540	fatty acid beta-oxidation using acyl-CoA oxidase	biological_process	7	0	0	0	0	0	0	1	0	0	0	2.53	2.28
GO:0001085	RNA polymerase II transcription factor binding	molecular_function	12	0	0	0	0	0	1	0	0	0	0	2.71	2.28
GO:0051209	release of sequestered calcium ion into cytosol	biological_process	5	0	0	0	0	0	0	1	0	0	0	2.79	2.28
GO:0032418	lysosome localization	biological_process	8	0	0	0	0	1	0	0	0	0	0	2.56	2.28
GO:0004197	cysteine-type endopeptidase activity	molecular_function	32	0	0	1	0	0	0	0	0	0	2.63	0	2.28
GO:0002804	positive regulation of antifungal peptide production	biological_process	9	0	0	0	0	0	0	0	1	0	0	2.56	2.28
GO:0048312	intracellular distribution of mitochondria	biological_process	5	0	0	0	0	1	0	0	0	0	0	2.7	2.28
GO:0007432	salivary gland boundary specification	biological_process	11	0	0	0	0	0	1	0	0	0	0	2.7	2.28
GO:0033403	UUA codon-amino acid adaptor activity	molecular_function	4	0	0	0	0	1	0	0	0	0	0	2.58	2.28
GO:0004435	phosphatidylinositol phospholipase C activity	molecular_function	3	0	0	0	0	0	0	1	0	0	0	2.79	2.28
GO:0070059	intrinsic apoptotic signaling pathway in response to endoplasmic reticulum stress	biological_process	5	0	0	0	0	1	0	0	0	0	0	2.53	2.28
GO:0032959	inositol trisphosphate biosynthetic process	biological_process	3	0	0	0	0	0	0	1	0	0	0	2.79	2.28
GO:0046622	positive regulation of organ growth	biological_process	11	0	0	0	0	1	0	0	0	0	0	2.59	2.27
GO:0007362	terminal region determination	biological_process	17	0	0	0	0	1	0	0	0	0	0	2.52	2.27
GO:0042766	nucleosome mobilization	biological_process	11	0	0	0	0	1	0	0	0	0	0	2.52	2.27
GO:0016015	morphogen activity	molecular_function	4	0	0	0	0	1	0	0	0	0	0	2.83	2.27
GO:0048599	oocyte development	biological_process	7	0	0	0	0	0	0	1	0	0	0	2.52	2.27
GO:0007313	maternal specification of dorsal/ventral axis, oocyte, soma encoded	biological_process	6	0	0	0	0	1	0	0	0	0	0	2.83	2.27
GO:0006911	phagocytosis, engulfment	biological_process	7	0	0	0	0	1	0	0	0	0	0	2.51	2.26
GO:0016570	histone modification	biological_process	7	0	0	0	0	1	0	0	0	0	0	2.89	2.26
GO:0045314	regulation of compound eye photoreceptor development	biological_process	7	0	0	0	1	0	0	0	0	0	2.61	0	2.26
GO:0019888	protein phosphatase regulator activity	molecular_function	8	0	0	0	0	1	0	0	0	0	0	2.52	2.26
GO:0007402	ganglion mother cell fate determination	biological_process	7	0	0	0	0	1	0	0	0	0	0	2.51	2.26
GO:1901987	regulation of cell cycle phase transition	biological_process	3	0	0	1	0	0	0	0	0	0	2.69	0	2.26
GO:0005198	structural molecule activity	molecular_function	28	0	0	0	0	0	0	0	1	0	0	2.52	2.26
GO:0046959	habituation	biological_process	12	0	0	0	0	0	0	1	0	0	0	2.52	2.26
GO:0016279	protein-lysine N-methyltransferase activity	molecular_function	9	0	0	0	0	0	0	0	1	0	0	2.51	2.25
GO:0007530	sex determination	biological_process	16	0	0	0	0	0	0	0	1	0	0	2.51	2.25
GO:0017147	Wnt-protein binding	molecular_function	16	0	0	0	0	0	0	0	1	0	0	2.6	2.25
GO:0008409	5'-3' exonuclease activity	molecular_function	7	0	1	0	0	0	0	0	0	0	2.62	0	2.25
GO:0000413	protein peptidyl-prolyl isomerization	biological_process	21	0	0	0	0	0	0	1	0	0	0	2.51	2.25
GO:0030716	oocyte fate determination	biological_process	11	0	0	0	0	0	0	1	0	0	0	2.5	2.24
GO:0019933	cAMP-mediated signaling	biological_process	9	0	0	0	0	0	0	1	0	0	0	2.83	2.24
GO:0008307	structural constituent of muscle	molecular_function	16	0	0	0	0	1	0	0	0	0	0	2.5	2.24
GO:0046527	glucosyltransferase activity	molecular_function	6	0	0	1	0	0	0	0	0	0	2.59	0	2.24
GO:0046427	positive regulation of JAK-STAT cascade	biological_process	11	0	0	1	0	0	0	0	0	0	2.59	0	2.24

GO:0033437	ACU codon-amino acid adaptor activity	molecular_function	8	0	0	0	0	1	0	0	0	0	0	2.75	2.24
GO:0000062	fatty-acyl-CoA binding	molecular_function	11	0	0	0	0	0	0	0	0	1	0	2.5	2.24
GO:0006790	sulfur compound metabolic process	biological_process	4	0	0	0	0	0	0	0	1	0	0	2.57	2.24
GO:0006633	fatty acid biosynthetic process	biological_process	32	0	0	0	0	1	0	0	0	0	0	2.51	2.24
GO:0015645	fatty acid ligase activity	molecular_function	16	0	0	0	0	1	0	0	0	0	0	2.51	2.24
GO:0030628	pre-mRNA 3'-splice site binding	molecular_function	5	0	0	0	0	0	0	1	0	0	0	2.6	2.24
GO:0030422	production of siRNA involved in RNA interference	biological_process	12	1	0	0	0	0	0	0	0	0	2.59	0	2.24
GO:0030676	Rac guanyl-nucleotide exchange factor activity	molecular_function	9	0	0	0	0	0	0	0	1	0	0	2.48	2.23
GO:2000252	negative regulation of feeding behavior	biological_process	8	0	0	0	0	1	0	0	0	0	0	2.49	2.23
GO:0043113	receptor clustering	biological_process	5	0	0	0	0	0	1	0	0	0	0	2.49	2.23
GO:0010997	anaphase-promoting complex binding	molecular_function	5	1	0	0	0	0	0	0	0	0	2.63	0	2.22
GO:0034427	nuclear-transcribed mRNA catabolic process, exonucleolytic, 3'-5'	biological_process	7	0	1	0	0	0	0	0	0	0	2.77	0	2.22
GO:0007367	segment polarity determination	biological_process	40	0	0	0	0	0	0	0	1	0	0	2.47	2.22
GO:0070983	dendrite guidance	biological_process	24	0	0	0	0	0	0	0	1	0	0	2.48	2.22
GO:0045450	bicoid mRNA localization	biological_process	22	0	0	1	0	0	0	0	0	0	2.57	0	2.22
GO:1904668	positive regulation of ubiquitin protein ligase activity	biological_process	4	1	0	0	0	0	0	0	0	0	2.63	0	2.22
GO:0007062	sister chromatid cohesion	biological_process	15	0	0	1	0	0	0	0	0	0	2.65	0	2.22
GO:0097027	ubiquitin-protein transferase activator activity	molecular_function	4	1	0	0	0	0	0	0	0	0	2.63	0	2.22
GO:0007626	locomotory behavior	biological_process	16	0	0	0	0	0	0	0	0	1	0	2.47	2.22
GO:0034475	U4 snRNA 3'-end processing	biological_process	7	0	1	0	0	0	0	0	0	0	2.77	0	2.22
GO:0051117	ATPase binding	molecular_function	8	0	0	1	0	0	0	0	0	0	2.56	0	2.21
GO:0007527	adult somatic muscle development	biological_process	30	0	1	0	0	0	0	0	0	0	2.57	0	2.21
GO:0006090	pyruvate metabolic process	biological_process	19	1	0	0	0	0	0	0	0	0	2.56	0	2.21
GO:0051415	interphase microtubule nucleation by interphase microtubule organizing center	biological_process	9	0	0	1	0	0	0	0	0	0	2.56	0	2.21
GO:0004596	peptide alpha-N-acetyltransferase activity	molecular_function	12	0	0	0	0	1	0	0	0	0	0	2.45	2.2
GO:0007271	synaptic transmission, cholinergic	biological_process	10	0	0	0	0	0	0	0	1	0	0	2.45	2.2
GO:0017196	N-terminal peptidyl-methionine acetylation	biological_process	9	0	0	0	0	1	0	0	0	0	0	2.45	2.2
GO:0007604	phototransduction, UV	biological_process	5	0	0	0	0	1	0	0	0	0	0	2.45	2.2
GO:0043001	Golgi to plasma membrane protein transport	biological_process	14	0	0	0	0	0	1	0	0	0	0	2.45	2.2
GO:0043697	cell dedifferentiation	biological_process	10	0	0	0	0	1	0	0	0	0	0	2.46	2.2
GO:0019730	antimicrobial humoral response	biological_process	7	0	0	0	0	0	0	0	1	0	0	2.44	2.19
GO:0031647	regulation of protein stability	biological_process	17	0	0	1	0	0	0	0	0	0	2.59	0	2.19
GO:0006998	nuclear envelope organization	biological_process	5	0	0	0	0	0	0	0	0	1	0	2.5	2.18
GO:0006006	glucose metabolic process	biological_process	15	0	0	0	0	0	0	0	1	0	0	2.66	2.18
GO:0045677	negative regulation of R7 cell differentiation	biological_process	6	0	0	0	0	0	0	1	0	0	0	2.46	2.18
GO:0007613	memory	biological_process	28	0	0	0	0	0	1	0	0	0	0	2.47	2.18
GO:0022904	respiratory electron transport chain	biological_process	9	0	0	0	0	0	0	0	1	0	0	2.77	2.18
GO:0051085	chaperone mediated protein folding requiring cofactor	biological_process	28	1	0	0	0	0	0	0	0	0	2.6	0	2.18
GO:0097254	renal tubular secretion	biological_process	8	0	0	0	0	0	0	0	1	0	0	2.45	2.18
GO:0071481	cellular response to X-ray	biological_process	6	0	0	0	0	1	0	0	0	0	0	2.43	2.18
GO:0097104	postsynaptic membrane assembly	biological_process	6	0	0	0	0	1	0	0	0	0	0	2.42	2.17
GO:0034551	mitochondrial respiratory chain complex III assembly	biological_process	7	0	0	0	0	0	0	1	0	0	0	2.43	2.17
GO:0003998	acylphosphatase activity	molecular_function	6	0	0	1	0	0	0	0	0	0	2.53	0	2.17
GO:0097105	presynaptic membrane assembly	biological_process	8	0	0	0	0	1	0	0	0	0	0	2.42	2.17
GO:0042043	neurexin family protein binding	molecular_function	10	0	0	0	0	1	0	0	0	0	0	2.42	2.17
GO:0045433	male courtship behavior, veined wing generated song production	biological_process	15	0	0	0	0	0	0	1	0	0	0	2.42	2.17

GO:0004571	mannosyl-oligosaccharide 1,2-alpha-mannosidase activity	molecular_function	8	0	0	1	0	0	0	0	0	0	2.61	0	2.16
GO:0008293	torso signaling pathway	biological_process	22	0	0	0	0	1	0	0	0	0	0	2.42	2.16
GO:0050919	negative chemotaxis	biological_process	5	0	0	0	0	0	0	0	1	0	0	2.64	2.16
GO:0035074	pupation	biological_process	7	0	0	0	0	0	0	0	1	0	0	2.94	2.16
GO:0046665	amnioserosa maintenance	biological_process	6	1	0	0	0	0	0	0	0	0	2.5	0	2.15
GO:0030866	cortical actin cytoskeleton organization	biological_process	30	0	0	0	0	0	0	1	0	0	0	2.44	2.15
GO:0009755	hormone-mediated signaling pathway	biological_process	15	0	0	0	0	0	1	0	0	0	0	2.61	2.15
GO:0070252	actin-mediated cell contraction	biological_process	5	0	0	0	0	0	0	1	0	0	0	2.41	2.15
GO:0045167	asymmetric protein localization involved in cell fate determination	biological_process	17	0	0	0	0	1	0	0	0	0	0	2.5	2.15
GO:0048567	ectodermal digestive tract morphogenesis	biological_process	4	0	0	0	0	0	1	0	0	0	0	2.43	2.15
GO:0004724	magnesium-dependent protein serine/threonine phosphatase activity	molecular_function	10	1	0	0	0	0	0	0	0	0	2.51	0	2.15
GO:0003689	DNA clamp loader activity	molecular_function	7	0	0	1	0	0	0	0	0	0	2.65	0	2.14
GO:0007348	regulation of syncytial blastoderm mitotic cell cycle	biological_process	8	0	0	0	0	0	0	1	0	0	0	2.4	2.14
GO:0000076	DNA replication checkpoint	biological_process	8	0	0	0	0	0	0	1	0	0	0	2.43	2.14
GO:0006805	xenobiotic metabolic process	biological_process	8	0	0	0	0	0	0	0	1	0	0	2.44	2.13
GO:2000377	regulation of reactive oxygen species metabolic process	biological_process	8	0	0	0	0	1	0	0	0	0	0	2.43	2.13
GO:0051729	germline cell cycle switching, mitotic to meiotic cell cycle	biological_process	4	0	1	0	0	0	0	0	0	0	2.48	0	2.13
GO:0007020	microtubule nucleation	biological_process	11	0	0	1	0	0	0	0	0	0	2.55	0	2.13
GO:0004693	cyclin-dependent protein serine/threonine kinase activity	molecular_function	14	0	0	1	0	0	0	0	0	0	2.6	0	2.13
GO:1902476	chloride transmembrane transport	biological_process	17	0	0	0	0	1	0	0	0	0	0	2.41	2.12
GO:0008168	methyltransferase activity	molecular_function	17	0	0	0	0	0	1	0	0	0	0	2.37	2.12
GO:0048082	regulation of adult chitin-containing cuticle pigmentation	biological_process	12	0	0	0	0	1	0	0	0	0	0	2.38	2.12
GO:0051123	RNA polymerase II transcriptional preinitiation complex assembly	biological_process	14	0	0	1	0	0	0	0	0	0	2.47	0	2.12
GO:0071482	cellular response to light stimulus	biological_process	17	0	0	0	0	1	0	0	0	0	0	2.37	2.12
GO:0042461	photoreceptor cell development	biological_process	12	0	0	0	0	0	1	0	0	0	0	2.39	2.12
GO:0043044	ATP-dependent chromatin remodeling	biological_process	18	0	0	0	0	1	0	0	0	0	0	2.38	2.12
GO:0051091	positive regulation of sequence-specific DNA binding transcription factor activity	biological_process	13	0	0	0	0	0	1	0	0	0	0	2.37	2.12
GO:0001737	establishment of imaginal disc-derived wing hair orientation	biological_process	23	0	0	0	0	0	0	1	0	0	0	2.37	2.11
GO:0010001	glial cell differentiation	biological_process	11	1	0	0	0	0	0	0	0	0	2.46	0	2.11
GO:0042063	gliogenesis	biological_process	4	0	0	0	0	0	0	0	1	0	0	2.54	2.11
GO:0044255	cellular lipid metabolic process	biological_process	6	1	0	0	0	0	0	0	0	0	2.46	0	2.11
GO:0000165	MAPK cascade	biological_process	11	0	0	0	0	1	0	0	0	0	0	2.53	2.1
GO:0006400	tRNA modification	biological_process	10	0	0	0	1	0	0	0	0	0	2.56	0	2.1
GO:0072375	medium-term memory	biological_process	18	0	0	0	0	0	0	0	1	0	0	2.36	2.1
GO:0019941	modification-dependent protein catabolic process	biological_process	6	0	0	1	0	0	0	0	0	0	2.54	0	2.1
GO:0006122	mitochondrial electron transport, ubiquinol to cytochrome c	biological_process	16	0	0	0	0	0	0	1	0	0	0	2.45	2.1
GO:0045824	negative regulation of innate immune response	biological_process	25	0	0	0	0	0	0	0	1	0	0	2.36	2.1
GO:0051489	regulation of filopodium assembly	biological_process	12	0	0	0	0	0	0	1	0	0	0	2.36	2.1
GO:0042744	hydrogen peroxide catabolic process	biological_process	10	0	0	0	0	0	1	0	0	0	0	2.38	2.1
GO:0045931	positive regulation of mitotic cell cycle	biological_process	10	0	0	0	0	1	0	0	0	0	0	2.34	2.09
GO:0043052	thermotaxis	biological_process	20	0	0	0	0	1	0	0	0	0	0	2.35	2.09
GO:0007093	mitotic cell cycle checkpoint	biological_process	10	0	0	0	0	0	0	1	0	0	0	2.34	2.09
GO:0051306	mitotic sister chromatid separation	biological_process	5	0	0	1	0	0	0	0	0	0	2.5	0	2.08
GO:0004300	enoyl-CoA hydratase activity	molecular_function	8	0	0	0	0	0	0	1	0	0	0	2.33	2.08

GO:0045332	phospholipid translocation	biological_process	7	0	0	0	0	0	0	1	0	0	0	2.33	2.08
GO:0050774	negative regulation of dendrite morphogenesis	biological_process	3	0	0	0	0	1	0	0	0	0	0	2.46	2.08
GO:0006272	leading strand elongation	biological_process	8	0	0	1	0	0	0	0	0	0	2.6	0	2.08
GO:0005507	copper ion binding	molecular_function	23	0	0	0	0	1	0	0	0	0	0	2.33	2.08
GO:0005547	phosphatidylinositol-3,4,5-trisphosphate binding	molecular_function	5	0	1	0	0	0	0	0	0	0	2.43	0	2.08
GO:0008235	metalloexopeptidase activity	molecular_function	14	0	0	1	0	0	0	0	0	0	2.43	0	2.08
GO:0006384	transcription initiation from RNA polymerase III promoter	biological_process	6	0	1	0	0	0	0	0	0	0	2.6	0	2.08
GO:0007295	growth of a germarium-derived egg chamber	biological_process	10	0	0	0	0	1	0	0	0	0	0	2.47	2.07
GO:0030833	regulation of actin filament polymerization	biological_process	19	0	0	0	0	0	0	0	1	0	0	2.33	2.07
GO:0003755	peptidyl-prolyl cis-trans isomerase activity	molecular_function	27	0	0	0	0	0	0	1	0	0	0	2.32	2.07
GO:0005088	Ras guanyl-nucleotide exchange factor activity	molecular_function	8	0	0	0	0	0	0	0	1	0	0	2.33	2.07
GO:0005109	frizzled binding	molecular_function	10	0	0	0	0	0	0	0	1	0	0	2.32	2.07
GO:0003725	double-stranded RNA binding	molecular_function	21	0	0	0	0	1	0	0	0	0	0	2.32	2.07
GO:0007301	female germline ring canal formation	biological_process	10	0	0	0	0	0	0	1	0	0	0	2.32	2.07
GO:0042051	compound eye photoreceptor development	biological_process	26	0	0	0	0	1	0	0	0	0	0	2.33	2.07
GO:0033500	carbohydrate homeostasis	biological_process	12	0	0	0	0	1	0	0	0	0	0	2.31	2.06
GO:0030031	cell projection assembly	biological_process	15	0	0	0	0	0	0	0	1	0	0	2.32	2.06
GO:0051537	2 iron, 2 sulfur cluster binding	molecular_function	20	0	0	0	0	0	0	0	1	0	0	2.35	2.05
GO:0045333	cellular respiration	biological_process	8	0	0	0	0	0	0	1	0	0	0	2.31	2.05
GO:0000470	maturation of LSU-rRNA	biological_process	16	0	0	1	0	0	0	0	0	0	2.44	0	2.05
GO:0022848	acetylcholine-gated cation-selective channel activity	molecular_function	10	0	0	0	0	0	0	0	1	0	0	2.3	2.05
GO:0030536	larval feeding behavior	biological_process	21	0	0	0	0	1	0	0	0	0	0	2.29	2.04
GO:0038202	TORC1 signaling	biological_process	8	0	1	0	0	0	0	0	0	0	2.4	0	2.04
GO:0007288	sperm axoneme assembly	biological_process	25	0	0	1	0	0	0	0	0	0	2.43	0	2.04
GO:0042254	ribosome biogenesis	biological_process	16	0	0	0	0	1	0	0	0	0	0	2.35	2.04
GO:0048142	germarium-derived cystoblast division	biological_process	10	0	0	1	0	0	0	0	0	0	2.39	0	2.04
GO:0006509	membrane protein ectodomain proteolysis	biological_process	9	0	0	0	1	0	0	0	0	0	2.39	0	2.04
GO:0017156	calcium ion regulated exocytosis	biological_process	9	0	0	0	0	0	1	0	0	0	0	2.29	2.03
GO:0048791	calcium ion-regulated exocytosis of neurotransmitter	biological_process	6	0	0	0	0	0	1	0	0	0	0	2.29	2.03
GO:0071277	cellular response to calcium ion	biological_process	7	0	0	0	0	0	1	0	0	0	0	2.29	2.03
GO:0014059	regulation of dopamine secretion	biological_process	8	0	0	0	0	0	1	0	0	0	0	2.29	2.03
GO:0030276	clathrin binding	molecular_function	15	0	0	0	0	0	1	0	0	0	0	2.29	2.03
GO:0017158	regulation of calcium ion-dependent exocytosis	biological_process	7	0	0	0	0	0	1	0	0	0	0	2.29	2.03
GO:0001752	compound eye photoreceptor fate commitment	biological_process	11	0	0	0	0	1	0	0	0	0	0	2.28	2.03
GO:0007611	learning or memory	biological_process	16	0	0	0	0	0	0	0	1	0	0	2.46	2.03
GO:0001666	response to hypoxia	biological_process	34	0	0	0	0	0	0	1	0	0	0	2.42	2.03
GO:0035335	peptidyl-tyrosine dephosphorylation	biological_process	9	0	0	0	0	0	0	1	0	0	0	2.29	2.03
GO:0016204	determination of muscle attachment site	biological_process	9	0	0	0	0	0	0	0	1	0	0	2.49	2.03
GO:0071456	cellular response to hypoxia	biological_process	29	0	0	0	0	0	0	0	1	0	0	2.46	2.02
GO:0007603	phototransduction, visible light	biological_process	12	0	0	0	0	1	0	0	0	0	0	2.28	2.02
GO:0032869	cellular response to insulin stimulus	biological_process	23	0	0	0	0	0	0	0	1	0	0	2.28	2.02
GO:0031440	regulation of mRNA 3'-end processing	biological_process	8	0	0	0	0	0	1	0	0	0	0	2.28	2.02
GO:0051011	microtubule minus-end binding	molecular_function	10	0	0	1	0	0	0	0	0	0	2.37	0	2.02

GO:0001178	regulation of transcriptional start site selection at RNA polymerase II promoter	biological_process	8	0	0	0	0	0	1	0	0	0	0	2.28	2.02
GO:0000077	DNA damage checkpoint	biological_process	15	0	0	0	0	0	0	1	0	0	0	2.31	2.02
GO:0045572	positive regulation of imaginal disc growth	biological_process	19	0	0	0	0	1	0	0	0	0	0	2.41	2.02
GO:0007405	neuroblast proliferation	biological_process	19	0	0	0	0	1	0	0	0	0	0	2.26	2.01
GO:0043519	regulation of myosin II filament organization	biological_process	7	0	0	0	0	0	0	1	0	0	0	2.26	2.01
GO:0048383	mesectoderm development	biological_process	6	0	0	0	0	0	0	1	0	0	0	2.26	2.01
GO:0045434	negative regulation of female receptivity, post-mating	biological_process	16	0	0	0	1	0	0	0	0	0	2.36	0	2.01
GO:0007158	neuron cell-cell adhesion	biological_process	8	0	0	0	0	1	0	0	0	0	0	2.27	2.01
GO:0006417	regulation of translation	biological_process	19	0	0	0	1	0	0	0	0	0	2.36	0	2.01
GO:0032880	regulation of protein localization	biological_process	16	0	0	1	0	0	0	0	0	0	2.46	0	2.01
GO:0016311	dephosphorylation	biological_process	44	0	0	0	0	0	1	0	0	0	0	2.27	2.01
GO:0035002	liquid clearance, open tracheal system	biological_process	23	0	1	0	0	0	0	0	0	0	2.36	0	2.01
GO:1900073	regulation of neuromuscular synaptic transmission	biological_process	12	0	0	0	0	0	1	0	0	0	0	2.41	2.01
GO:0007290	spermatid nucleus elongation	biological_process	4	1	0	0	0	0	0	0	0	0	2.47	0	2
GO:0005214	structural constituent of chitin-based cuticle	molecular_function	37	0	1	0	0	0	0	0	0	0	2.4	0	2
GO:0007369	gastrulation	biological_process	10	0	0	0	0	0	0	1	0	0	0	2.68	2
GO:0007549	dosage compensation	biological_process	18	0	0	0	0	0	0	1	0	0	0	2.32	2
GO:0035094	response to nicotine	biological_process	8	0	0	0	1	0	0	0	0	0	2.36	0	2
GO:0010043	response to zinc ion	biological_process	9	0	0	0	0	1	0	0	0	0	0	2.3	1.99
GO:1902669	positive regulation of axon guidance	biological_process	13	0	0	0	0	0	0	0	1	0	0	2.42	1.99
GO:0031386	protein tag	molecular_function	11	0	0	1	0	0	0	0	0	0	2.42	0	1.99
GO:0006915	apoptotic process	biological_process	49	0	0	0	0	0	0	0	1	0	0	2.25	1.99
GO:0048665	neuron fate specification	biological_process	8	0	0	0	0	1	0	0	0	0	0	2.24	1.99
GO:0000086	G2/M transition of mitotic cell cycle	biological_process	13	0	0	1	0	0	0	0	0	0	2.39	0	1.99
GO:1990269	RNA polymerase II C-terminal domain phosphoserine binding	molecular_function	5	0	0	0	0	1	0	0	0	0	0	2.23	1.98
GO:0007406	negative regulation of neuroblast proliferation	biological_process	19	0	0	0	0	0	1	0	0	0	0	2.24	1.98
GO:0070374	positive regulation of ERK1 and ERK2 cascade	biological_process	23	0	0	0	0	0	0	0	1	0	0	2.26	1.98
GO:0008138	protein tyrosine/serine/threonine phosphatase activity	molecular_function	13	0	0	0	0	0	0	0	1	0	0	2.24	1.98
GO:0043486	histone exchange	biological_process	19	0	0	0	0	0	0	0	1	0	0	2.24	1.98
GO:0007266	Rho protein signal transduction	biological_process	24	0	0	0	0	0	0	1	0	0	0	2.24	1.98
GO:0008592	regulation of Toll signaling pathway	biological_process	9	0	0	0	0	0	0	0	1	0	0	2.43	1.98
GO:0001736	establishment of planar polarity	biological_process	30	0	0	0	0	0	0	1	0	0	0	2.24	1.98
GO:0048800	antennal morphogenesis	biological_process	16	0	0	0	0	1	0	0	0	0	0	2.38	1.98
GO:0007430	terminal branching, open tracheal system	biological_process	38	0	0	0	0	1	0	0	0	0	0	2.27	1.97
GO:0033410	UAC codon-amino acid adaptor activity	molecular_function	9	0	0	0	0	0	0	0	0	1	0	2.53	1.97
GO:0050907	detection of chemical stimulus involved in sensory perception	biological_process	12	0	0	1	0	0	0	0	0	0	2.37	0	1.97
GO:0035011	melanotic encapsulation of foreign target	biological_process	30	0	0	0	0	0	0	0	1	0	0	2.23	1.97
GO:0035096	larval midgut cell programmed cell death	biological_process	27	0	0	0	0	0	0	0	1	0	0	2.39	1.97
GO:0071805	potassium ion transmembrane transport	biological_process	27	1	0	0	0	0	0	0	0	0	2.38	0	1.97
GO:0035264	multicellular organism growth	biological_process	11	0	0	0	0	1	0	0	0	0	0	2.33	1.97
GO:0070328	triglyceride homeostasis	biological_process	30	1	0	0	0	0	0	0	0	0	2.31	0	1.96
GO:0004721	phosphoprotein phosphatase activity	molecular_function	26	0	0	0	0	0	0	0	0	1	0	2.22	1.96
GO:0048803	imaginal disc-derived male genitalia morphogenesis	biological_process	18	0	0	1	0	0	0	0	0	0	2.44	0	1.96
GO:0007502	digestive tract mesoderm development	biological_process	7	0	0	1	0	0	0	0	0	0	2.31	0	1.96

GO:0045187	regulation of circadian sleep/wake cycle, sleep	biological_process	17	0	0	0	0	0	0	0	1	0	0	2.26	1.96
GO:0045751	negative regulation of Toll signaling pathway	biological_process	14	0	0	0	0	0	0	0	1	0	0	2.21	1.95
GO:0001786	phosphatidylserine binding	molecular_function	11	0	0	0	0	0	1	0	0	0	0	2.2	1.95
GO:0004521	endoribonuclease activity	molecular_function	29	0	0	0	0	1	0	0	0	0	0	2.21	1.95
GO:0007638	mechanosensory behavior	biological_process	19	0	0	0	0	0	0	1	0	0	0	2.21	1.95
GO:0034314	Arp2/3 complex-mediated actin nucleation	biological_process	11	0	0	0	0	0	0	1	0	0	0	2.26	1.95
GO:0016579	protein deubiquitination	biological_process	38	0	0	0	0	0	0	0	1	0	0	2.22	1.94
GO:0007615	anesthesia-resistant memory	biological_process	17	0	0	0	0	0	0	0	1	0	0	2.23	1.94
GO:0042127	regulation of cell proliferation	biological_process	31	0	0	0	0	1	0	0	0	0	0	2.26	1.94
GO:0030239	myofibril assembly	biological_process	15	0	1	0	0	0	0	0	0	0	2.3	0	1.94
GO:0043015	gamma-tubulin binding	molecular_function	11	0	0	1	0	0	0	0	0	0	2.29	0	1.94
GO:0008356	asymmetric cell division	biological_process	22	0	0	0	0	1	0	0	0	0	0	2.22	1.94
GO:0048100	wing disc anterior/posterior pattern formation	biological_process	16	0	0	0	0	1	0	0	0	0	0	2.26	1.93
GO:0006368	transcription elongation from RNA polymerase II promoter	biological_process	18	0	0	0	0	1	0	0	0	0	0	2.42	1.93
GO:0042026	protein refolding	biological_process	34	0	0	0	0	0	0	1	0	0	0	2.19	1.93
GO:0007622	rhythmic behavior	biological_process	11	0	0	0	0	0	0	0	1	0	0	2.31	1.93
GO:0045184	establishment of protein localization	biological_process	6	0	0	0	0	0	0	1	0	0	0	2.46	1.93
GO:0003384	apical constriction involved in gastrulation	biological_process	9	0	0	0	0	0	0	1	0	0	0	2.17	1.92
GO:0022849	glutamate-gated calcium ion channel activity	molecular_function	6	0	0	0	0	1	0	0	0	0	0	2.29	1.92
GO:0045570	regulation of imaginal disc growth	biological_process	11	0	0	0	0	1	0	0	0	0	0	2.29	1.92
GO:0030150	protein import into mitochondrial matrix	biological_process	27	0	0	0	0	0	0	0	0	1	0	2.17	1.92
GO:0035317	imaginal disc-derived wing hair organization	biological_process	25	0	0	0	0	0	0	1	0	0	0	2.18	1.92
GO:0016787	hydrolase activity	molecular_function	27	0	0	0	0	0	0	0	0	1	0	2.17	1.92
GO:0061327	anterior Malpighian tubule development	biological_process	12	0	0	0	0	0	0	0	1	0	0	2.25	1.91
GO:0007140	male meiosis	biological_process	34	0	0	1	0	0	0	0	0	0	2.34	0	1.91
GO:0018094	protein polyglycylation	biological_process	12	0	0	1	0	0	0	0	0	0	2.27	0	1.91
GO:0008094	DNA-dependent ATPase activity	molecular_function	20	0	0	0	0	1	0	0	0	0	0	2.16	1.91
GO:0016538	cyclin-dependent protein serine/threonine kinase regulator activity	molecular_function	14	0	0	0	1	0	0	0	0	0	2.33	0	1.91
GO:0009055	electron carrier activity	molecular_function	32	0	0	0	0	0	0	0	1	0	0	2.22	1.91
GO:0000460	maturation of 5.8S rRNA	biological_process	11	0	0	1	0	0	0	0	0	0	2.31	0	1.91
GO:0061320	pericardial nephrocyte differentiation	biological_process	10	1	0	0	0	0	0	0	0	0	2.26	0	1.91
GO:0040040	thermosensory behavior	biological_process	18	0	0	0	0	0	0	0	1	0	0	2.35	1.91
GO:0004843	thiol-dependent ubiquitin-specific protease activity	molecular_function	39	0	0	0	0	0	0	0	1	0	0	2.15	1.9
GO:1905515	non-motile cilium assembly	biological_process	23	0	0	0	0	0	0	0	0	1	0	2.16	1.9
GO:0000979	RNA polymerase II core promoter sequence-specific DNA binding	molecular_function	9	0	0	1	0	0	0	0	0	0	2.25	0	1.9
GO:0045466	R7 cell differentiation	biological_process	11	0	0	0	0	1	0	0	0	0	0	2.65	1.89
GO:0045165	cell fate commitment	biological_process	26	0	0	0	0	0	0	0	1	0	0	2.15	1.89
GO:0008289	lipid binding	molecular_function	33	0	0	0	0	1	0	0	0	0	0	2.14	1.89
GO:0043130	ubiquitin binding	molecular_function	29	0	0	0	0	0	0	1	0	0	0	2.14	1.89
GO:0035159	regulation of tube length, open tracheal system	biological_process	29	0	1	0	0	0	0	0	0	0	2.3	0	1.89
GO:0035218	leg disc development	biological_process	10	0	0	0	0	0	0	0	1	0	0	2.15	1.89
GO:0000146	microfilament motor activity	molecular_function	12	0	0	1	0	0	0	0	0	0	2.23	0	1.88
GO:0060548	negative regulation of cell death	biological_process	20	0	0	0	0	0	0	0	1	0	0	2.14	1.88
GO:0016055	Wnt signaling pathway	biological_process	21	0	0	0	0	0	0	0	1	0	0	2.14	1.88

GO:0007390	germ-band shortening	biological_process	21	1	0	0	0	0	0	0	0	0	2.23	0	1.88
GO:0035222	wing disc pattern formation	biological_process	21	0	0	0	0	0	0	0	1	0	0	2.27	1.88
GO:0016063	rhodopsin biosynthetic process	biological_process	15	0	0	0	0	1	0	0	0	0	0	2.13	1.88
GO:2000331	regulation of terminal button organization	biological_process	25	0	0	0	0	1	0	0	0	0	0	2.18	1.88
GO:0016476	regulation of embryonic cell shape	biological_process	21	0	0	0	0	0	0	0	1	0	0	2.18	1.88
GO:0000082	G1/S transition of mitotic cell cycle	biological_process	11	0	0	1	0	0	0	0	0	0	2.33	0	1.88
GO:0008335	female germline ring canal stabilization	biological_process	5	0	0	0	0	0	0	1	0	0	0	2.37	1.88
GO:0030050	vesicle transport along actin filament	biological_process	10	0	0	1	0	0	0	0	0	0	2.23	0	1.88
GO:0007317	regulation of pole plasm oskar mRNA localization	biological_process	22	0	0	0	0	1	0	0	0	0	0	2.25	1.87
GO:0007366	periodic partitioning by pair rule gene	biological_process	8	0	0	0	0	0	0	0	0	1	0	2.14	1.87
GO:0018345	protein palmitoylation	biological_process	24	0	0	0	0	0	0	0	1	0	0	2.15	1.87
GO:0055088	lipid homeostasis	biological_process	39	0	0	0	0	1	0	0	0	0	0	2.12	1.87
GO:0010507	negative regulation of autophagy	biological_process	19	0	0	0	0	1	0	0	0	0	0	2.15	1.86
GO:0007379	segment specification	biological_process	20	0	0	0	0	1	0	0	0	0	0	2.16	1.86
GO:0051879	Hsp90 protein binding	molecular_function	9	0	0	1	0	0	0	0	0	0	2.2	0	1.85
GO:0007143	female meiotic division	biological_process	43	0	0	0	0	0	0	1	0	0	0	2.13	1.85
GO:0035186	syncytial blastoderm mitotic cell cycle	biological_process	17	0	0	0	1	0	0	0	0	0	2.2	0	1.85
GO:0042593	glucose homeostasis	biological_process	41	0	0	0	0	1	0	0	0	0	0	2.18	1.85
GO:0005516	calmodulin binding	molecular_function	36	0	0	0	0	0	0	1	0	0	0	2.11	1.85
GO:0007517	muscle organ development	biological_process	37	0	0	0	0	1	0	0	0	0	0	2.27	1.84
GO:0004866	endopeptidase inhibitor activity	molecular_function	8	0	0	0	0	0	1	0	0	0	0	2.1	1.84
GO:0008066	glutamate receptor activity	molecular_function	13	0	0	0	0	1	0	0	0	0	0	2.23	1.84
GO:0006310	DNA recombination	biological_process	21	0	0	0	0	0	0	0	1	0	0	2.1	1.84
GO:0007526	larval somatic muscle development	biological_process	35	0	0	0	0	1	0	0	0	0	0	2.09	1.84
GO:0060025	regulation of synaptic activity	biological_process	16	0	0	0	0	1	0	0	0	0	0	2.09	1.84
GO:0046329	negative regulation of JNK cascade	biological_process	21	1	0	0	0	0	0	0	0	0	2.19	0	1.84
GO:0060361	flight	biological_process	16	0	0	0	0	0	1	0	0	0	0	2.39	1.83
GO:0004181	metallocarboxypeptidase activity	molecular_function	23	0	1	0	0	0	0	0	0	0	2.18	0	1.83
GO:0000030	mannosyltransferase activity	molecular_function	12	0	0	0	0	0	0	0	1	0	0	2.36	1.83
GO:0000723	telomere maintenance	biological_process	24	0	0	0	0	0	0	1	0	0	0	2.08	1.83
GO:0051959	dynein light intermediate chain binding	molecular_function	19	1	0	0	0	0	0	0	0	0	2.25	0	1.83
GO:0000212	meiotic spindle organization	biological_process	22	0	1	0	0	0	0	0	0	0	2.19	0	1.82
GO:0007300	ovarian nurse cell to oocyte transport	biological_process	11	0	0	0	0	0	0	1	0	0	0	2.07	1.82
GO:0016360	sensory organ precursor cell fate determination	biological_process	22	0	0	0	0	1	0	0	0	0	0	2.09	1.82
GO:0005102	receptor binding	molecular_function	36	0	0	0	0	0	0	0	1	0	0	2.07	1.82
GO:0045879	negative regulation of smoothened signaling pathway	biological_process	50	0	0	0	0	0	0	0	1	0	0	2.06	1.81
GO:0016246	RNA interference	biological_process	33	1	0	0	0	0	0	0	0	0	2.17	0	1.81
GO:1904315	transmitter-gated ion channel activity involved in regulation of postsynaptic membrane potential	molecular_function	11	0	0	0	0	1	0	0	0	0	0	2.14	1.8
GO:0046627	negative regulation of insulin receptor signaling pathway	biological_process	26	0	0	1	0	0	0	0	0	0	2.16	0	1.8
GO:0030898	actin-dependent ATPase activity	molecular_function	11	0	0	1	0	0	0	0	0	0	2.15	0	1.8

GO:0045471	response to ethanol	biological_process	13	0	0	0	0	0	0	1	0	0	0	2.05	1.8
GO:0007155	cell adhesion	biological_process	51	0	0	0	0	0	1	0	0	0	0	2.3	1.8
GO:0008354	germ cell migration	biological_process	38	0	0	0	0	0	1	0	0	0	0	2.09	1.79
GO:0006261	DNA-dependent DNA replication	biological_process	26	0	0	1	0	0	0	0	0	0	2.27	0	1.79
GO:0005172	vascular endothelial growth factor receptor binding	molecular_function	4	0	0	0	0	0	0	0	1	0	0	2.5	1.79
GO:0035167	larval lymph gland hemopoiesis	biological_process	33	0	0	0	0	1	0	0	0	0	0	2.04	1.79
GO:0007076	mitotic chromosome condensation	biological_process	23	0	0	0	1	0	0	0	0	0	2.2	0	1.79
GO:0018107	peptidyl-threonine phosphorylation	biological_process	16	0	0	0	0	0	0	1	0	0	0	2.5	1.79
GO:0005112	Notch binding	molecular_function	21	0	0	0	0	1	0	0	0	0	0	2.04	1.79
GO:0004653	polypeptide N-acetylgalactosaminyltransferase activity	molecular_function	14	0	0	0	0	0	0	0	1	0	0	2.06	1.78
GO:0008201	heparin binding	molecular_function	19	0	0	0	0	0	0	0	1	0	0	2.23	1.78
GO:0008345	larval locomotory behavior	biological_process	31	0	0	0	0	0	0	0	1	0	0	2.04	1.78
GO:0019905	syntaxin binding	molecular_function	25	0	0	0	0	0	1	0	0	0	0	2.03	1.78
GO:0008010	structural constituent of chitin-based larval cuticle	molecular_function	105	0	0	0	0	1	0	0	0	0	0	2.03	1.78
GO:0032482	Rab protein signal transduction	biological_process	36	0	1	0	0	0	0	0	0	0	2.13	0	1.78
GO:0016348	imaginal disc-derived leg joint morphogenesis	biological_process	14	0	0	0	0	0	0	0	1	0	0	2.03	1.78
GO:0036098	male germ-line stem cell population maintenance	biological_process	16	0	0	0	0	0	0	0	1	0	0	2.22	1.77
GO:0097352	autophagosome maturation	biological_process	23	0	0	1	0	0	0	0	0	0	2.12	0	1.77
GO:0006351	transcription, DNA-templated	biological_process	35	0	0	0	0	0	0	0	1	0	0	2.05	1.77
GO:0016197	endosomal transport	biological_process	25	0	0	0	0	0	0	1	0	0	0	2.09	1.77
GO:0045475	locomotor rhythm	biological_process	62	0	0	0	0	0	0	0	1	0	0	2.02	1.76
GO:0019991	septate junction assembly	biological_process	34	0	0	0	0	0	0	0	0	1	0	2.01	1.76
GO:0008258	head involution	biological_process	50	1	0	0	0	0	0	0	0	0	2.12	0	1.76
GO:0098656	anion transmembrane transport	biological_process	9	0	0	0	0	1	0	0	0	0	0	2.13	1.76
GO:0016199	axon midline choice point recognition	biological_process	25	0	0	1	0	0	0	0	0	0	2.1	0	1.75
GO:0005261	cation channel activity	molecular_function	23	0	0	0	0	0	0	1	0	0	0	2.07	1.75
GO:0004298	threonine-type endopeptidase activity	molecular_function	27	0	0	0	0	0	0	1	0	0	0	2.01	1.75
GO:0010499	proteasomal ubiquitin-independent protein catabolic process	biological_process	25	0	0	0	0	0	0	1	0	0	0	2.01	1.75
GO:0042073	intraciliary transport	biological_process	15	0	0	0	0	0	0	0	1	0	0	2	1.75
GO:0010498	proteasomal protein catabolic process	biological_process	27	0	0	0	0	0	0	1	0	0	0	2.01	1.75
GO:0042302	structural constituent of cuticle	molecular_function	102	0	0	0	0	1	0	0	0	0	0	2.02	1.75
GO:0035195	gene silencing by miRNA	biological_process	44	1	0	0	0	0	0	0	0	0	2.14	0	1.75
GO:0008239	dipeptidyl-peptidase activity	molecular_function	17	0	0	0	0	1	0	0	0	0	0	2.05	1.74
GO:0035249	synaptic transmission, glutamatergic	biological_process	13	0	0	0	0	1	0	0	0	0	0	2.15	1.74
GO:0035050	embryonic heart tube development	biological_process	21	1	0	0	0	0	0	0	0	0	2.08	0	1.73
GO:0045214	sarcomere organization	biological_process	34	0	1	0	0	0	0	0	0	0	2.08	0	1.73
GO:0001708	cell fate specification	biological_process	31	0	0	0	0	1	0	0	0	0	0	2.12	1.73
GO:0009306	protein secretion	biological_process	50	0	0	0	0	0	0	0	1	0	0	1.98	1.73
GO:0045464	R8 cell fate specification	biological_process	17	0	0	0	0	0	0	1	0	0	0	2.09	1.73
GO:0007420	brain development	biological_process	40	0	0	0	0	0	0	1	0	0	0	1.99	1.73
GO:0004190	aspartic-type endopeptidase activity	molecular_function	15	0	0	0	0	0	0	1	0	0	0	1.98	1.72
GO:0006487	protein N-linked glycosylation	biological_process	29	0	0	1	0	0	0	0	0	0	2.13	0	1.72
GO:0042078	germ-line stem cell division	biological_process	18	0	0	1	0	0	0	0	0	0	2.07	0	1.72
GO:0004402	histone acetyltransferase activity	molecular_function	30	0	0	0	0	0	0	0	1	0	0	1.96	1.71
GO:0031122	cytoplasmic microtubule organization	biological_process	27	0	0	1	0	0	0	0	0	0	2.11	0	1.71
GO:0031145	anaphase-promoting complex-dependent catabolic process	biological_process	20	1	0	0	0	0	0	0	0	0	2.05	0	1.7

GO:0007297	ovarian follicle cell migration	biological_process	13	0	0	0	1	0	0	0	0	0	2.05	0	1.7
GO:0070491	repressing transcription factor binding	molecular_function	19	0	0	0	0	1	0	0	0	0	0	1.95	1.7
GO:0006013	mannose metabolic process	biological_process	10	0	0	1	0	0	0	0	0	0	2.05	0	1.7
GO:0006890	retrograde vesicle-mediated transport, Golgi to ER	biological_process	22	0	0	1	0	0	0	0	0	0	2.06	0	1.69
GO:0016267	O-glycan processing, core 1	biological_process	11	0	0	0	0	0	0	0	1	0	0	1.94	1.69
GO:0016263	glycoprotein-N-acetylgalactosamine 3-beta-galactosyltransferase activity	molecular_function	11	0	0	0	0	0	0	0	1	0	0	1.94	1.69
GO:0007623	circadian rhythm	biological_process	44	0	0	0	0	0	0	0	1	0	0	1.99	1.69
GO:0035147	branch fusion, open tracheal system	biological_process	21	0	0	0	0	1	0	0	0	0	0	2.14	1.68
GO:0050660	flavin adenine dinucleotide binding	molecular_function	53	0	0	0	0	1	0	0	0	0	0	1.93	1.68
GO:0004559	alpha-mannosidase activity	molecular_function	9	0	0	1	0	0	0	0	0	0	2.01	0	1.66
GO:0045505	dynein intermediate chain binding	molecular_function	29	1	0	0	0	0	0	0	0	0	2.07	0	1.66
GO:0009408	response to heat	biological_process	65	0	0	0	0	0	0	1	0	0	0	2.01	1.66
GO:0005125	cytokine activity	molecular_function	18	0	0	0	0	0	0	0	1	0	0	2.09	1.65
GO:0006517	protein deglycosylation	biological_process	17	0	0	1	0	0	0	0	0	0	2	0	1.65
GO:0035215	genital disc development	biological_process	13	0	0	0	0	1	0	0	0	0	0	2.03	1.65
GO:0009312	oligosaccharide biosynthetic process	biological_process	21	0	0	0	0	0	0	0	1	0	0	1.9	1.64
GO:0007220	Notch receptor processing	biological_process	15	0	0	0	1	0	0	0	0	0	1.99	0	1.64
GO:0000070	mitotic sister chromatid segregation	biological_process	32	0	0	1	0	0	0	0	0	0	2.01	0	1.64
GO:0033458	GAC codon-amino acid adaptor activity	molecular_function	11	0	0	0	1	0	0	0	0	0	1.99	0	1.64
GO:0000149	SNARE binding	molecular_function	36	0	0	0	0	0	1	0	0	0	0	1.93	1.64
GO:0033227	dsRNA transport	biological_process	26	0	0	0	0	1	0	0	0	0	0	1.98	1.64
GO:0030261	chromosome condensation	biological_process	35	0	0	0	0	0	0	1	0	0	0	1.88	1.63
GO:0007269	neurotransmitter secretion	biological_process	64	0	0	0	0	0	1	0	0	0	0	1.89	1.63
GO:0007427	epithelial cell migration, open tracheal system	biological_process	36	0	0	0	0	1	0	0	0	0	0	2.03	1.63
GO:0048675	axon extension	biological_process	29	0	0	0	0	0	0	0	1	0	0	1.99	1.61
GO:0036099	female germ-line stem cell population maintenance	biological_process	32	0	0	0	0	1	0	0	0	0	0	1.9	1.61
GO:0035023	regulation of Rho protein signal transduction	biological_process	16	0	0	0	0	0	0	0	1	0	0	2.17	1.61
GO:0009953	dorsal/ventral pattern formation	biological_process	35	0	0	0	0	1	0	0	0	0	0	1.86	1.6
GO:0048149	behavioral response to ethanol	biological_process	68	0	0	0	0	0	0	1	0	0	0	1.88	1.6
GO:0030198	extracellular matrix organization	biological_process	26	0	1	0	0	0	0	0	0	0	1.95	0	1.6
GO:0007112	male meiosis cytokinesis	biological_process	34	0	0	1	0	0	0	0	0	0	2.01	0	1.59
GO:0048488	synaptic vesicle endocytosis	biological_process	37	0	0	0	0	1	0	0	0	0	0	1.85	1.59
GO:0008360	regulation of cell shape	biological_process	43	0	0	0	0	0	0	1	0	0	0	1.92	1.59
GO:0042048	olfactory behavior	biological_process	47	0	0	0	0	0	0	0	1	0	0	2.02	1.58
GO:0031146	SCF-dependent proteasomal ubiquitin-dependent protein catabolic process	biological_process	65	0	0	0	0	0	0	0	1	0	0	1.83	1.58
GO:0051225	spindle assembly	biological_process	39	0	0	1	0	0	0	0	0	0	1.98	0	1.58
GO:0051726	regulation of cell cycle	biological_process	45	0	0	0	0	1	0	0	0	0	0	1.83	1.57
GO:0005201	extracellular matrix structural constituent	molecular_function	33	0	0	0	0	0	0	0	1	0	0	1.97	1.57
GO:0007189	adenylate cyclase-activating G-protein coupled receptor signaling pathway	biological_process	29	0	0	0	0	0	1	0	0	0	0	1.83	1.56
GO:0045746	negative regulation of Notch signaling pathway	biological_process	41	0	0	0	0	1	0	0	0	0	0	1.81	1.56
GO:0016747	transferase activity, transferring acyl groups other than amino-acyl groups	molecular_function	32	0	0	0	0	0	1	0	0	0	0	1.81	1.56
GO:0030335	positive regulation of cell migration	biological_process	16	0	0	0	0	0	0	0	1	0	0	2.09	1.55
GO:0046716	muscle cell cellular homeostasis	biological_process	45	0	0	0	0	1	0	0	0	0	0	1.8	1.55
GO:0000724	double-strand break repair via homologous recombination	biological_process	40	0	0	0	0	0	0	0	1	0	0	1.81	1.55

GO:0006367	transcription initiation from RNA polymerase II promoter	biological_process	51	0	0	1	0	0	0	0	0	0	1.89	0	1.54
GO:0033041	sweet taste receptor activity	molecular_function	42	0	0	0	0	1	0	0	0	0	0	1.79	1.53
GO:0016788	hydrolase activity, acting on ester bonds	molecular_function	30	0	0	1	0	0	0	0	0	0	1.92	0	1.52
GO:0006816	calcium ion transport	biological_process	32	0	0	0	0	1	0	0	0	0	0	1.94	1.51
GO:0048542	lymph gland development	biological_process	31	0	0	0	0	1	0	0	0	0	0	1.94	1.51
GO:0006812	cation transport	biological_process	31	0	0	0	0	0	0	0	1	0	0	1.85	1.51
GO:0050911	detection of chemical stimulus involved in sensory perception of smell	biological_process	72	0	0	0	0	1	0	0	0	0	0	1.76	1.51
GO:0042147	retrograde transport, endosome to Golgi	biological_process	29	0	0	0	0	1	0	0	0	0	0	1.75	1.5
GO:0004984	olfactory receptor activity	molecular_function	70	0	0	0	0	1	0	0	0	0	0	1.72	1.47
GO:0052689	carboxylic ester hydrolase activity	molecular_function	49	0	0	1	0	0	0	0	0	0	1.83	0	1.46
GO:0006897	endocytosis	biological_process	60	0	1	0	0	0	0	0	0	0	1.8	0	1.45
GO:0000287	magnesium ion binding	molecular_function	50	0	0	0	0	0	0	1	0	0	0	1.68	1.43
GO:0005975	carbohydrate metabolic process	biological_process	85	0	0	1	0	0	0	0	0	0	1.81	0	1.43
GO:0007304	chorion-containing eggshell formation	biological_process	31	0	0	0	0	0	0	0	1	0	0	1.87	1.43
GO:0016318	ommatidial rotation	biological_process	32	1	0	0	0	0	0	0	0	0	1.76	0	1.42
GO:0038023	signaling receptor activity	molecular_function	54	0	0	0	0	1	0	0	0	0	0	1.75	1.41
GO:0008104	protein localization	biological_process	59	0	0	0	0	0	0	0	1	0	0	1.69	1.39
GO:0030718	germ-line stem cell population maintenance	biological_process	42	0	0	0	0	1	0	0	0	0	0	1.74	1.37
GO:0008406	gonad development	biological_process	37	0	0	1	0	0	0	0	0	0	1.68	0	1.34
GO:0007275	multicellular organism development	biological_process	60	0	0	0	0	0	0	0	1	0	0	1.7	1.34
GO:0006629	lipid metabolic process	biological_process	44	0	0	1	0	0	0	0	0	0	1.68	0	1.33
GO:0006352	DNA-templated transcription, initiation	biological_process	31	0	0	1	0	0	0	0	0	0	1.93	0	1.32
GO:0030154	cell differentiation	biological_process	88	0	0	0	0	0	1	0	0	0	0	1.66	1.31
GO:0008039	synaptic target recognition	biological_process	33	0	0	0	0	0	0	0	1	0	0	1.71	1.31
GO:0050909	sensory perception of taste	biological_process	63	0	0	0	0	1	0	0	0	0	0	1.6	1.28
GO:0016319	mushroom body development	biological_process	86	0	0	0	0	0	0	1	0	0	0	1.67	1.27
GO:0008527	taste receptor activity	molecular_function	55	0	0	0	0	1	0	0	0	0	0	1.56	1.27
GO:0007423	sensory organ development	biological_process	55	0	0	0	0	1	0	0	0	0	0	1.64	1.23
GO:0030246	carbohydrate binding	molecular_function	68	0	0	1	0	0	0	0	0	0	1.47	0	1.12
GO:0000981	RNA polymerase II transcription factor activity, sequence-specific DNA binding	molecular_function	172	1	0	0	0	0	0	0	0	0	1.44	0	1.1
GO:0032504	multicellular organism reproduction	biological_process	247	0	0	0	0	0	0	0	1	0	0	1.03	0.8
GO:0090062	regulation of trehalose metabolic process	biological_process	3	0	0	0	0	0	0	0	0	0	0	0	0
GO:0090110	cargo loading into COPII-coated vesicle	biological_process	4	0	0	0	0	0	0	0	0	0	0	0	0
GO:0042659	regulation of cell fate specification	biological_process	10	0	0	0	0	0	0	0	0	0	0	0	0
GO:0090141	positive regulation of mitochondrial fission	biological_process	7	0	0	0	0	0	0	0	0	0	0	0	0
GO:0090328	regulation of olfactory learning	biological_process	10	0	0	0	0	0	0	0	0	0	0	0	0
GO:2000274	regulation of epithelial cell migration, open tracheal system	biological_process	10	0	0	0	0	0	0	0	0	0	0	0	0
GO:0042176	regulation of protein catabolic process	biological_process	5	0	0	0	0	0	0	0	0	0	0	0	0
GO:0042407	cristae formation	biological_process	10	0	0	0	0	0	0	0	0	0	0	0	0
GO:0035072	ecdysone-mediated induction of salivary gland cell autophagic cell death	biological_process	14	0	0	0	0	0	0	0	0	0	0	0	0
GO:0090382	phagosome maturation	biological_process	3	0	0	0	0	0	0	0	0	0	0	0	0
GO:0042169	SH2 domain binding	molecular_function	8	0	0	0	0	0	0	0	0	0	0	0	0
GO:0090070	positive regulation of ribosome biogenesis	biological_process	4	0	0	0	0	0	0	0	0	0	0	0	0
GO:2000177	regulation of neural precursor cell proliferation	biological_process	10	0	0	0	0	0	0	0	0	0	0	0	0
GO:0042542	response to hydrogen peroxide	biological_process	10	0	0	0	0	0	0	0	0	0	0	0	0

GO:0035066	positive regulation of histone acetylation	biological_process	3	0	0	0	0	0	0	0	0	0	0	0	0
GO:0090482	vitamin transmembrane transporter activity	molecular_function	3	0	0	0	0	0	0	0	0	0	0	0	0
GO:2000370	positive regulation of clathrin-dependent endocytosis	biological_process	6	0	0	0	0	0	0	0	0	0	0	0	0
GO:0035070	salivary gland histolysis	biological_process	11	0	0	0	0	0	0	0	0	0	0	0	0
GO:0090090	negative regulation of canonical Wnt signaling pathway	biological_process	44	0	0	0	0	0	0	0	0	0	0	0	0
GO:0035022	positive regulation of Rac protein signal transduction	biological_process	3	0	0	0	0	0	0	0	0	0	0	0	0
GO:0090309	positive regulation of methylation-dependent chromatin silencing	biological_process	7	0	0	0	0	0	0	0	0	0	0	0	0
GO:0042675	compound eye cone cell differentiation	biological_process	10	0	0	0	0	0	0	0	0	0	0	0	0
GO:0090158	endoplasmic reticulum membrane organization	biological_process	5	0	0	0	0	0	0	0	0	0	0	0	0
GO:0035064	methylated histone binding	molecular_function	18	0	0	0	0	0	0	0	0	0	0	0	0
GO:0090175	regulation of establishment of planar polarity	biological_process	18	0	0	0	0	0	0	0	0	0	0	0	0
GO:0035042	fertilization, exchange of chromosomal proteins	biological_process	7	0	0	0	0	0	0	0	0	0	0	0	0
GO:0035035	histone acetyltransferase binding	molecular_function	5	0	0	0	0	0	0	0	0	0	0	0	0
GO:0035051	cardiocyte differentiation	biological_process	6	0	0	0	0	0	0	0	0	0	0	0	0
GO:0090176	microtubule cytoskeleton organization involved in establishment of planar polarity	biological_process	3	0	0	0	0	0	0	0	0	0	0	0	0
GO:0090210	regulation of establishment of blood-brain barrier	biological_process	3	0	0	0	0	0	0	0	0	0	0	0	0
GO:0090251	protein localization involved in establishment of planar polarity	biological_process	7	0	0	0	0	0	0	0	0	0	0	0	0
GO:0035025	positive regulation of Rho protein signal transduction	biological_process	15	0	0	0	0	0	0	0	0	0	0	0	0
GO:0090163	establishment of epithelial cell planar polarity	biological_process	5	0	0	0	0	0	0	0	0	0	0	0	0
GO:0090254	cell elongation involved in imaginal disc-derived wing morphogenesis	biological_process	11	0	0	0	0	0	0	0	0	0	0	0	0
GO:0090263	positive regulation of canonical Wnt signaling pathway	biological_process	48	0	0	0	0	0	0	0	0	0	0	0	0
GO:0042277	peptide binding	molecular_function	29	0	0	0	0	0	0	0	0	0	0	0	0
GO:0042307	positive regulation of protein import into nucleus	biological_process	4	0	0	0	0	0	0	0	0	0	0	0	0
GO:0090162	establishment of epithelial cell polarity	biological_process	4	0	0	0	0	0	0	0	0	0	0	0	0
GO:0035065	regulation of histone acetylation	biological_process	6	0	0	0	0	0	0	0	0	0	0	0	0
GO:2000300	regulation of synaptic vesicle exocytosis	biological_process	3	0	0	0	0	0	0	0	0	0	0	0	0
GO:0090307	mitotic spindle assembly	biological_process	12	0	0	0	0	0	0	0	0	0	0	0	0
GO:0042332	gravitaxis	biological_process	28	0	0	0	0	0	0	0	0	0	0	0	0
GO:0042335	cuticle development	biological_process	10	0	0	0	0	0	0	0	0	0	0	0	0
GO:0042246	tissue regeneration	biological_process	11	0	0	0	0	0	0	0	0	0	0	0	0
GO:0042381	hemolymph coagulation	biological_process	6	0	0	0	0	0	0	0	0	0	0	0	0
GO:0042386	hemocyte differentiation	biological_process	7	0	0	0	0	0	0	0	0	0	0	0	0
GO:2000289	regulation of photoreceptor cell axon guidance	biological_process	3	0	0	0	0	0	0	0	0	0	0	0	0
GO:0035289	posterior head segmentation	biological_process	11	0	0	0	0	0	0	0	0	0	0	0	0
GO:0080182	histone H3-K4 trimethylation	biological_process	3	0	0	0	0	0	0	0	0	0	0	0	0
GO:2000766	negative regulation of cytoplasmic translation	biological_process	3	0	0	0	0	0	0	0	0	0	0	0	0
GO:0043014	alpha-tubulin binding	molecular_function	7	0	0	0	0	0	0	0	0	0	0	0	0
GO:0034599	cellular response to oxidative stress	biological_process	20	0	0	0	0	0	0	0	0	0	0	0	0
GO:0042981	regulation of apoptotic process	biological_process	31	0	0	0	0	0	0	0	0	0	0	0	0
GO:0042834	peptidoglycan binding	molecular_function	18	0	0	0	0	0	0	0	0	0	0	0	0
GO:0042832	defense response to protozoan	biological_process	3	0	0	0	0	0	0	0	0	0	0	0	0
GO:0034605	cellular response to heat	biological_process	21	0	0	0	0	0	0	0	0	0	0	0	0
GO:2000738	positive regulation of stem cell differentiation	biological_process	5	0	0	0	0	0	0	0	0	0	0	0	0
GO:0034584	piRNA binding	molecular_function	4	0	0	0	0	0	0	0	0	0	0	0	0
GO:0042826	histone deacetylase binding	molecular_function	21	0	0	0	0	0	0	0	0	0	0	0	0
GO:0034614	cellular response to reactive oxygen species	biological_process	6	0	0	0	0	0	0	0	0	0	0	0	0

GO:0042813	Wnt-activated receptor activity	molecular_function	6	0	0	0	0	0	0	0	0	0	0	0	0
GO:0034620	cellular response to unfolded protein	biological_process	17	0	0	0	0	0	0	0	0	0	0	0	0
GO:0071855	neuropeptide receptor binding	molecular_function	11	0	0	0	0	0	0	0	0	0	0	0	0
GO:0042802	identical protein binding	molecular_function	27	0	0	0	0	0	0	0	0	0	0	0	0
GO:0034587	piRNA metabolic process	biological_process	11	0	0	0	0	0	0	0	0	0	0	0	0
GO:0034553	mitochondrial respiratory chain complex II assembly	biological_process	5	0	0	0	0	0	0	0	0	0	0	0	0
GO:0034644	cellular response to UV	biological_process	7	0	0	0	0	0	0	0	0	0	0	0	0
GO:0043065	positive regulation of apoptotic process	biological_process	34	0	0	0	0	0	0	0	0	0	0	0	0
GO:0043069	negative regulation of programmed cell death	biological_process	7	0	0	0	0	0	0	0	0	0	0	0	0
GO:2001046	positive regulation of integrin-mediated signaling pathway	biological_process	3	0	0	0	0	0	0	0	0	0	0	0	0
GO:0043066	negative regulation of apoptotic process	biological_process	72	0	0	0	0	0	0	0	0	0	0	0	0
GO:2001023	regulation of response to drug	biological_process	3	0	0	0	0	0	0	0	0	0	0	0	0
GO:0034472	snRNA 3'-end processing	biological_process	14	0	0	0	0	0	0	0	0	0	0	0	0
GO:0034497	protein localization to pre-autophagosomal structure	biological_process	6	0	0	0	0	0	0	0	0	0	0	0	0
GO:0071689	muscle thin filament assembly	biological_process	5	0	0	0	0	0	0	0	0	0	0	0	0
GO:0043021	ribonucleoprotein complex binding	molecular_function	7	0	0	0	0	0	0	0	0	0	0	0	0
GO:0071694	maintenance of protein location in extracellular region	biological_process	3	0	0	0	0	0	0	0	0	0	0	0	0
GO:0071702	organic substance transport	biological_process	8	0	0	0	0	0	0	0	0	0	0	0	0
GO:2001020	regulation of response to DNA damage stimulus	biological_process	5	0	0	0	0	0	0	0	0	0	0	0	0
GO:0043035	chromatin insulator sequence binding	molecular_function	15	0	0	0	0	0	0	0	0	0	0	0	0
GO:0071711	basement membrane organization	biological_process	16	0	0	0	0	0	0	0	0	0	0	0	0
GO:0071763	nuclear membrane organization	biological_process	3	0	0	0	0	0	0	0	0	0	0	0	0
GO:0042800	histone methyltransferase activity (H3-K4 specific)	molecular_function	15	0	0	0	0	0	0	0	0	0	0	0	0
GO:0034720	histone H3-K4 demethylation	biological_process	3	0	0	0	0	0	0	0	0	0	0	0	0
GO:0042683	negative regulation of compound eye cone cell fate specification	biological_process	3	0	0	0	0	0	0	0	0	0	0	0	0
GO:0072659	protein localization to plasma membrane	biological_process	36	0	0	0	0	0	0	0	0	0	0	0	0
GO:0072582	17-beta-hydroxysteroid dehydrogenase (NADP+) activity	molecular_function	8	0	0	0	0	0	0	0	0	0	0	0	0
GO:0042749	regulation of circadian sleep/wake cycle	biological_process	7	0	0	0	0	0	0	0	0	0	0	0	0
GO:0072583	clathrin-dependent endocytosis	biological_process	9	0	0	0	0	0	0	0	0	0	0	0	0
GO:0042742	defense response to bacterium	biological_process	60	0	0	0	0	0	0	0	0	0	0	0	0
GO:0035010	encapsulation of foreign target	biological_process	13	0	0	0	0	0	0	0	0	0	0	0	0
GO:0072657	protein localization to membrane	biological_process	9	0	0	0	0	0	0	0	0	0	0	0	0
GO:0042694	muscle cell fate specification	biological_process	7	0	0	0	0	0	0	0	0	0	0	0	0
GO:0042752	regulation of circadian rhythm	biological_process	23	0	0	0	0	0	0	0	0	0	0	0	0
GO:2000647	negative regulation of stem cell proliferation	biological_process	6	0	0	0	0	0	0	0	0	0	0	0	0
GO:0042690	negative regulation of crystal cell differentiation	biological_process	4	0	0	0	0	0	0	0	0	0	0	0	0
GO:0080025	phosphatidylinositol-3,5-bisphosphate binding	molecular_function	6	0	0	0	0	0	0	0	0	0	0	0	0
GO:0035017	cuticle pattern formation	biological_process	16	0	0	0	0	0	0	0	0	0	0	0	0
GO:0042688	crystal cell differentiation	biological_process	5	0	0	0	0	0	0	0	0	0	0	0	0
GO:0042685	cardioblast cell fate specification	biological_process	3	0	0	0	0	0	0	0	0	0	0	0	0
GO:0072555	17-beta-ketosteroid reductase activity	molecular_function	8	0	0	0	0	0	0	0	0	0	0	0	0
GO:0042761	very long-chain fatty acid biosynthetic process	biological_process	23	0	0	0	0	0	0	0	0	0	0	0	0
GO:0071896	protein localization to adherens junction	biological_process	4	0	0	0	0	0	0	0	0	0	0	0	0
GO:0035001	dorsal trunk growth, open tracheal system	biological_process	10	0	0	0	0	0	0	0	0	0	0	0	0
GO:0034765	regulation of ion transmembrane transport	biological_process	3	0	0	0	0	0	0	0	0	0	0	0	0
GO:0034982	mitochondrial protein processing	biological_process	3	0	0	0	0	0	0	0	0	0	0	0	0
GO:0042797	tRNA transcription from RNA polymerase III promoter	biological_process	3	0	0	0	0	0	0	0	0	0	0	0	0

GO:0042790	transcription of nuclear large rRNA transcript from RNA polymerase I promoter	biological_process	3	0	0	0	0	0	0	0	0	0	0	0	0
GO:0042776	mitochondrial ATP synthesis coupled proton transport	biological_process	8	0	0	0	0	0	0	0	0	0	0	0	0
GO:0042775	mitochondrial ATP synthesis coupled electron transport	biological_process	3	0	0	0	0	0	0	0	0	0	0	0	0
GO:2000736	regulation of stem cell differentiation	biological_process	12	0	0	0	0	0	0	0	0	0	0	0	0
GO:0072344	rescue of stalled ribosome	biological_process	3	0	0	0	0	0	0	0	0	0	0	0	0
GO:0071932	replication fork reversal	biological_process	3	0	0	0	0	0	0	0	0	0	0	0	0
GO:0035006	melanization defense response	biological_process	15	0	0	0	0	0	0	0	0	0	0	0	0
GO:0071949	FAD binding	molecular_function	24	0	0	0	0	0	0	0	0	0	0	0	0
GO:0042771	intrinsic apoptotic signaling pathway in response to DNA damage by p53 class mediator	biological_process	4	0	0	0	0	0	0	0	0	0	0	0	0
GO:0072334	UDP-galactose transmembrane transport	biological_process	5	0	0	0	0	0	0	0	0	0	0	0	0
GO:0042770	signal transduction in response to DNA damage	biological_process	4	0	0	0	0	0	0	0	0	0	0	0	0
GO:0035009	negative regulation of melanization defense response	biological_process	3	0	0	0	0	0	0	0	0	0	0	0	0
GO:0097156	fasciculation of motor neuron axon	biological_process	4	0	0	0	0	0	0	0	0	0	0	0	0
GO:0090502	RNA phosphodiester bond hydrolysis, endonucleolytic	biological_process	8	0	0	0	0	0	0	0	0	0	0	0	0
GO:1990399	epithelium regeneration	biological_process	5	0	0	0	0	0	0	0	0	0	0	0	0
GO:1990511	piRNA biosynthetic process	biological_process	8	0	0	0	0	0	0	0	0	0	0	0	0
GO:0035971	peptidyl-histidine dephosphorylation	biological_process	4	0	0	0	0	0	0	0	0	0	0	0	0
GO:0035883	enteroendocrine cell differentiation	biological_process	9	0	0	0	0	0	0	0	0	0	0	0	0
GO:0035725	sodium ion transmembrane transport	biological_process	5	0	0	0	0	0	0	0	0	0	0	0	0
GO:1990472	piRNA dual-strand cluster binding	molecular_function	3	0	0	0	0	0	0	0	0	0	0	0	0
GO:0071617	lysophospholipid acyltransferase activity	molecular_function	4	0	0	0	0	0	0	0	0	0	0	0	0
GO:0035615	clathrin adaptor activity	molecular_function	10	0	0	0	0	0	0	0	0	0	0	0	0
GO:0036089	cleavage furrow formation	biological_process	3	0	0	0	0	0	0	0	0	0	0	0	0
GO:0035591	signaling adaptor activity	molecular_function	5	0	0	0	0	0	0	0	0	0	0	0	0
GO:1903078	positive regulation of protein localization to plasma membrane	biological_process	4	0	0	0	0	0	0	0	0	0	0	0	0
GO:1903146	regulation of mitophagy	biological_process	6	0	0	0	0	0	0	0	0	0	0	0	0
GO:0035542	regulation of SNARE complex assembly	biological_process	8	0	0	0	0	0	0	0	0	0	0	0	0
GO:0035372	protein localization to microtubule	biological_process	6	0	0	0	0	0	0	0	0	0	0	0	0
GO:1903231	mRNA binding involved in posttranscriptional gene silencing	molecular_function	5	0	0	0	0	0	0	0	0	0	0	0	0
GO:0035217	labial disc development	biological_process	4	0	0	0	0	0	0	0	0	0	0	0	0
GO:0036090	cleavage furrow ingression	biological_process	4	0	0	0	0	0	0	0	0	0	0	0	0
GO:0035206	regulation of hemocyte proliferation	biological_process	28	0	0	0	0	0	0	0	0	0	0	0	0
GO:0036159	inner dynein arm assembly	biological_process	6	0	0	0	0	0	0	0	0	0	0	0	0
GO:1902626	assembly of large subunit precursor of preribosome	biological_process	3	0	0	0	0	0	0	0	0	0	0	0	0
GO:0036376	sodium ion export from cell	biological_process	8	0	0	0	0	0	0	0	0	0	0	0	0
GO:0035207	negative regulation of hemocyte proliferation	biological_process	7	0	0	0	0	0	0	0	0	0	0	0	0
GO:0036297	interstrand cross-link repair	biological_process	14	0	0	0	0	0	0	0	0	0	0	0	0
GO:0035208	positive regulation of hemocyte proliferation	biological_process	7	0	0	0	0	0	0	0	0	0	0	0	0
GO:0036228	protein targeting to nuclear inner membrane	biological_process	3	0	0	0	0	0	0	0	0	0	0	0	0
GO:0036158	outer dynein arm assembly	biological_process	12	0	0	0	0	0	0	0	0	0	0	0	0
GO:0035214	eye-antennal disc development	biological_process	18	0	0	0	0	0	0	0	0	0	0	0	0
GO:0036122	BMP binding	molecular_function	10	0	0	0	0	0	0	0	0	0	0	0	0
GO:0035209	pupal development	biological_process	9	0	0	0	0	0	0	0	0	0	0	0	0

GO:1902692	regulation of neuroblast proliferation	biological_process	15	0	0	0	0	0	0	0	0	0	0	0	0
GO:1902936	phosphatidylinositol bisphosphate binding	molecular_function	23	0	0	0	0	0	0	0	0	0	0	0	0
GO:0035211	spermathecum morphogenesis	biological_process	3	0	0	0	0	0	0	0	0	0	0	0	0
GO:1990573	potassium ion import across plasma membrane	biological_process	16	0	0	0	0	0	0	0	0	0	0	0	0
GO:1903723	negative regulation of centriole elongation	biological_process	3	0	0	0	0	0	0	0	0	0	0	0	0
GO:1903801	L-leucine import into cell	biological_process	3	0	0	0	0	0	0	0	0	0	0	0	0
GO:0035223	leg disc pattern formation	biological_process	4	0	0	0	0	0	0	0	0	0	0	0	0
GO:0035310	notum cell fate specification	biological_process	5	0	0	0	0	0	0	0	0	0	0	0	0
GO:0035277	spiracle morphogenesis, open tracheal system	biological_process	23	0	0	0	0	0	0	0	0	0	0	0	0
GO:1904398	positive regulation of neuromuscular junction development	biological_process	3	0	0	0	0	0	0	0	0	0	0	0	0
GO:1904825	protein localization to microtubule plus-end	biological_process	6	0	0	0	0	0	0	0	0	0	0	0	0
GO:1905349	ciliary transition zone assembly	biological_process	5	0	0	0	0	0	0	0	0	0	0	0	0
GO:1905475	regulation of protein localization to membrane	biological_process	3	0	0	0	0	0	0	0	0	0	0	0	0
GO:0035312	5'-3' exodeoxyribonuclease activity	molecular_function	3	0	0	0	0	0	0	0	0	0	0	0	0
GO:0035309	wing and notum subfield formation	biological_process	6	0	0	0	0	0	0	0	0	0	0	0	0
GO:0035224	genital disc anterior/posterior pattern formation	biological_process	8	0	0	0	0	0	0	0	0	0	0	0	0
GO:0035278	miRNA mediated inhibition of translation	biological_process	5	0	0	0	0	0	0	0	0	0	0	0	0
GO:0035282	segmentation	biological_process	15	0	0	0	0	0	0	0	0	0	0	0	0
GO:0035297	regulation of Malpighian tubule diameter	biological_process	3	0	0	0	0	0	0	0	0	0	0	0	0
GO:1905536	negative regulation of eukaryotic translation initiation factor 4F complex assembly	biological_process	3	0	0	0	0	0	0	0	0	0	0	0	0
GO:0035293	chitin-based larval cuticle pattern formation	biological_process	20	0	0	0	0	0	0	0	0	0	0	0	0
GO:1990050	phosphatidic acid transporter activity	molecular_function	3	0	0	0	0	0	0	0	0	0	0	0	0
GO:0035269	protein O-linked mannosylation	biological_process	12	0	0	0	0	0	0	0	0	0	0	0	0
GO:0035263	genital disc sexually dimorphic development	biological_process	5	0	0	0	0	0	0	0	0	0	0	0	0
GO:0035262	gonad morphogenesis	biological_process	6	0	0	0	0	0	0	0	0	0	0	0	0
GO:0035320	imaginal disc-derived wing hair site selection	biological_process	4	0	0	0	0	0	0	0	0	0	0	0	0
GO:0035257	nuclear hormone receptor binding	molecular_function	4	0	0	0	0	0	0	0	0	0	0	0	0
GO:0035250	UDP-galactosyltransferase activity	molecular_function	5	0	0	0	0	0	0	0	0	0	0	0	0
GO:1990182	exosomal secretion	biological_process	6	0	0	0	0	0	0	0	0	0	0	0	0
GO:0035321	maintenance of imaginal disc-derived wing hair orientation	biological_process	3	0	0	0	0	0	0	0	0	0	0	0	0
GO:1990247	N6-methyladenosine-containing RNA binding	molecular_function	5	0	0	0	0	0	0	0	0	0	0	0	0
GO:0035241	protein-arginine omega-N monomethyltransferase activity	molecular_function	7	0	0	0	0	0	0	0	0	0	0	0	0
GO:0035225	determination of genital disc primordium	biological_process	10	0	0	0	0	0	0	0	0	0	0	0	0
GO:1990386	mitotic cleavage furrow ingression	biological_process	3	0	0	0	0	0	0	0	0	0	0	0	0
GO:0035331	negative regulation of hippo signaling	biological_process	23	0	0	0	0	0	0	0	0	0	0	0	0
GO:0035332	positive regulation of hippo signaling	biological_process	20	0	0	0	0	0	0	0	0	0	0	0	0
GO:1904263	positive regulation of TORC1 signaling	biological_process	22	0	0	0	0	0	0	0	0	0	0	0	0
GO:1903862	positive regulation of oxidative phosphorylation	biological_process	6	0	0	0	0	0	0	0	0	0	0	0	0
GO:1903818	positive regulation of voltage-gated potassium channel activity	biological_process	6	0	0	0	0	0	0	0	0	0	0	0	0
GO:0036444	mitochondrial calcium uptake	biological_process	4	0	0	0	0	0	0	0	0	0	0	0	0
GO:0036466	synaptic vesicle recycling via endosome	biological_process	4	0	0	0	0	0	0	0	0	0	0	0	0
GO:0042162	telomeric DNA binding	molecular_function	5	0	0	0	0	0	0	0	0	0	0	0	0
GO:0098655	cation transmembrane transport	biological_process	6	0	0	0	0	0	0	0	0	0	0	0	0
GO:0097479	synaptic vesicle localization	biological_process	3	0	0	0	0	0	0	0	0	0	0	0	0
GO:0035093	spermatogenesis, exchange of chromosomal proteins	biological_process	10	0	0	0	0	0	0	0	0	0	0	0	0

GO:0097602	cullin family protein binding	molecular_function	17	0	0	0	0	0	0	0	0	0	0	0	0
GO:0098542	defense response to other organism	biological_process	6	0	0	0	0	0	0	0	0	0	0	0	0
GO:0098586	cellular response to virus	biological_process	10	0	0	0	0	0	0	0	0	0	0	0	0
GO:0098632	protein binding involved in cell-cell adhesion	molecular_function	10	0	0	0	0	0	0	0	0	0	0	0	0
GO:0042054	histone methyltransferase activity	molecular_function	12	0	0	0	0	0	0	0	0	0	0	0	0
GO:0035092	sperm chromatin condensation	biological_process	11	0	0	0	0	0	0	0	0	0	0	0	0
GO:0035149	lumen formation, open tracheal system	biological_process	35	0	0	0	0	0	0	0	0	0	0	0	0
GO:0098789	pre-mRNA cleavage required for polyadenylation	biological_process	4	0	0	0	0	0	0	0	0	0	0	0	0
GO:0042045	epithelial fluid transport	biological_process	4	0	0	0	0	0	0	0	0	0	0	0	0
GO:0035151	regulation of tube size, open tracheal system	biological_process	19	0	0	0	0	0	0	0	0	0	0	0	0
GO:0035152	regulation of tube architecture, open tracheal system	biological_process	8	0	0	0	0	0	0	0	0	0	0	0	0
GO:0098821	BMP receptor activity	molecular_function	4	0	0	0	0	0	0	0	0	0	0	0	0
GO:1990841	promoter-specific chromatin binding	molecular_function	7	0	0	0	0	0	0	0	0	0	0	0	0
GO:0042065	glial cell growth	biological_process	6	0	0	0	0	0	0	0	0	0	0	0	0
GO:1902600	hydrogen ion transmembrane transport	biological_process	57	0	0	0	0	0	0	0	0	0	0	0	0
GO:0035087	siRNA loading onto RISC involved in RNA interference	biological_process	4	0	0	0	0	0	0	0	0	0	0	0	0
GO:0097039	protein linear polyubiquitination	biological_process	3	0	0	0	0	0	0	0	0	0	0	0	0
GO:0035073	pupariation	biological_process	14	0	0	0	0	0	0	0	0	0	0	0	0
GO:2000134	negative regulation of G1/S transition of mitotic cell cycle	biological_process	11	0	0	0	0	0	0	0	0	0	0	0	0
GO:0035075	response to ecdysone	biological_process	19	0	0	0	0	0	0	0	0	0	0	0	0
GO:0097108	hedgehog family protein binding	molecular_function	4	0	0	0	0	0	0	0	0	0	0	0	0
GO:0035082	axoneme assembly	biological_process	11	0	0	0	0	0	0	0	0	0	0	0	0
GO:0035088	establishment or maintenance of apical/basal cell polarity	biological_process	10	0	0	0	0	0	0	0	0	0	0	0	0
GO:0042066	perineurial glial growth	biological_process	5	0	0	0	0	0	0	0	0	0	0	0	0
GO:0035091	phosphatidylinositol binding	molecular_function	23	0	0	0	0	0	0	0	0	0	0	0	0
GO:0097150	neuronal stem cell population maintenance	biological_process	10	0	0	0	0	0	0	0	0	0	0	0	0
GO:2000035	regulation of stem cell division	biological_process	8	0	0	0	0	0	0	0	0	0	0	0	0
GO:0035290	trunk segmentation	biological_process	8	0	0	0	0	0	0	0	0	0	0	0	0
GO:0097250	mitochondrial respiratory chain supercomplex assembly	biological_process	4	0	0	0	0	0	0	0	0	0	0	0	0
GO:0097320	plasma membrane tubulation	biological_process	7	0	0	0	0	0	0	0	0	0	0	0	0
GO:0101006	protein histidine phosphatase activity	molecular_function	4	0	0	0	0	0	0	0	0	0	0	0	0
GO:0101030	tRNA-guanine transglycosylation	biological_process	3	0	0	0	0	0	0	0	0	0	0	0	0
GO:0035155	negative regulation of terminal cell fate specification, open tracheal system	biological_process	3	0	0	0	0	0	0	0	0	0	0	0	0
GO:0040005	chitin-based cuticle attachment to epithelium	biological_process	3	0	0	0	0	0	0	0	0	0	0	0	0
GO:0035179	larval turning behavior	biological_process	3	0	0	0	0	0	0	0	0	0	0	0	0
GO:0035193	larval central nervous system remodeling	biological_process	6	0	0	0	0	0	0	0	0	0	0	0	0
GO:1900364	negative regulation of mRNA polyadenylation	biological_process	3	0	0	0	0	0	0	0	0	0	0	0	0
GO:0040008	regulation of growth	biological_process	22	0	0	0	0	0	0	0	0	0	0	0	0
GO:1900407	regulation of cellular response to oxidative stress	biological_process	3	0	0	0	0	0	0	0	0	0	0	0	0
GO:1901642	nucleoside transmembrane transport	biological_process	5	0	0	0	0	0	0	0	0	0	0	0	0
GO:0035199	salt aversion	biological_process	4	0	0	0	0	0	0	0	0	0	0	0	0
GO:0035158	regulation of tube diameter, open tracheal system	biological_process	11	0	0	0	0	0	0	0	0	0	0	0	0
GO:0035202	tracheal pit formation in open tracheal system	biological_process	10	0	0	0	0	0	0	0	0	0	0	0	0
GO:0038083	peptidyl-tyrosine autophosphorylation	biological_process	8	0	0	0	0	0	0	0	0	0	0	0	0
GO:0038061	NIK/NF-kappaB signaling	biological_process	3	0	0	0	0	0	0	0	0	0	0	0	0
GO:0038024	cargo receptor activity	molecular_function	11	0	0	0	0	0	0	0	0	0	0	0	0
GO:0035204	negative regulation of lamellocyte differentiation	biological_process	9	0	0	0	0	0	0	0	0	0	0	0	0

GO:0038004	epidermal growth factor receptor ligand maturation	biological_process	5	0	0	0	0	0	0	0	0	0	0	0	0
GO:0040012	regulation of locomotion	biological_process	4	0	0	0	0	0	0	0	0	0	0	0	0
GO:0035176	social behavior	biological_process	6	0	0	0	0	0	0	0	0	0	0	0	0
GO:1900264	positive regulation of DNA-directed DNA polymerase activity	biological_process	4	0	0	0	0	0	0	0	0	0	0	0	0
GO:0040015	negative regulation of multicellular organism growth	biological_process	11	0	0	0	0	0	0	0	0	0	0	0	0
GO:0040016	embryonic cleavage	biological_process	10	0	0	0	0	0	0	0	0	0	0	0	0
GO:0035172	hemocyte proliferation	biological_process	6	0	0	0	0	0	0	0	0	0	0	0	0
GO:0035171	lamellocyte differentiation	biological_process	5	0	0	0	0	0	0	0	0	0	0	0	0
GO:0040018	positive regulation of multicellular organism growth	biological_process	28	0	0	0	0	0	0	0	0	0	0	0	0
GO:0040020	regulation of meiotic nuclear division	biological_process	3	0	0	0	0	0	0	0	0	0	0	0	0
GO:1900182	positive regulation of protein localization to nucleus	biological_process	5	0	0	0	0	0	0	0	0	0	0	0	0
GO:1900087	positive regulation of G1/S transition of mitotic cell cycle	biological_process	10	0	0	0	0	0	0	0	0	0	0	0	0
GO:1990709	presynaptic active zone organization	biological_process	7	0	0	0	0	0	0	0	0	0	0	0	0
GO:0035170	lymph gland crystal cell differentiation	biological_process	8	0	0	0	0	0	0	0	0	0	0	0	0
GO:0042023	DNA endoreduplication	biological_process	18	0	0	0	0	0	0	0	0	0	0	0	0
GO:0035169	lymph gland plasmatocyte differentiation	biological_process	7	0	0	0	0	0	0	0	0	0	0	0	0
GO:1900075	positive regulation of neuromuscular synaptic transmission	biological_process	5	0	0	0	0	0	0	0	0	0	0	0	0
GO:0035165	embryonic crystal cell differentiation	biological_process	3	0	0	0	0	0	0	0	0	0	0	0	0
GO:0035721	intraciliary retrograde transport	biological_process	7	0	0	0	0	0	0	0	0	0	0	0	0
GO:0070734	histone H3-K27 methylation	biological_process	3	0	0	0	0	0	0	0	0	0	0	0	0
GO:0071577	zinc II ion transmembrane transport	biological_process	12	0	0	0	0	0	0	0	0	0	0	0	0
GO:0051480	regulation of cytosolic calcium ion concentration	biological_process	6	0	0	0	0	0	0	0	0	0	0	0	0
GO:0047617	acyl-CoA hydrolase activity	molecular_function	6	0	0	0	0	0	0	0	0	0	0	0	0
GO:0047555	3',5'-cyclic-GMP phosphodiesterase activity	molecular_function	6	0	0	0	0	0	0	0	0	0	0	0	0
GO:0047496	vesicle transport along microtubule	biological_process	4	0	0	0	0	0	0	0	0	0	0	0	0
GO:0047389	glycerophosphocholine phosphodiesterase activity	molecular_function	5	0	0	0	0	0	0	0	0	0	0	0	0
GO:0051491	positive regulation of filopodium assembly	biological_process	26	0	0	0	0	0	0	0	0	0	0	0	0
GO:0047372	acylglycerol lipase activity	molecular_function	3	0	0	0	0	0	0	0	0	0	0	0	0
GO:0047134	protein-disulfide reductase activity	molecular_function	3	0	0	0	0	0	0	0	0	0	0	0	0
GO:0051493	regulation of cytoskeleton organization	biological_process	9	0	0	0	0	0	0	0	0	0	0	0	0
GO:0051536	iron-sulfur cluster binding	molecular_function	9	0	0	0	0	0	0	0	0	0	0	0	0
GO:0051539	4 iron, 4 sulfur cluster binding	molecular_function	22	0	0	0	0	0	0	0	0	0	0	0	0
GO:0051560	mitochondrial calcium ion homeostasis	biological_process	5	0	0	0	0	0	0	0	0	0	0	0	0
GO:0046976	histone methyltransferase activity (H3-K27 specific)	molecular_function	5	0	0	0	0	0	0	0	0	0	0	0	0
GO:0051568	histone H3-K4 methylation	biological_process	24	0	0	0	0	0	0	0	0	0	0	0	0
GO:0046974	histone methyltransferase activity (H3-K9 specific)	molecular_function	7	0	0	0	0	0	0	0	0	0	0	0	0
GO:0051571	positive regulation of histone H3-K4 methylation	biological_process	5	0	0	0	0	0	0	0	0	0	0	0	0
GO:0046961	proton-transporting ATPase activity, rotational mechanism	molecular_function	35	0	0	0	0	0	0	0	0	0	0	0	0
GO:0046957	negative phototaxis	biological_process	5	0	0	0	0	0	0	0	0	0	0	0	0
GO:0046956	positive phototaxis	biological_process	4	0	0	0	0	0	0	0	0	0	0	0	0
GO:0051607	defense response to virus	biological_process	35	0	0	0	0	0	0	0	0	0	0	0	0
GO:0051490	negative regulation of filopodium assembly	biological_process	3	0	0	0	0	0	0	0	0	0	0	0	0
GO:0051453	regulation of intracellular pH	biological_process	6	0	0	0	0	0	0	0	0	0	0	0	0
GO:0051642	centrosome localization	biological_process	16	0	0	0	0	0	0	0	0	0	0	0	0
GO:0051445	regulation of meiotic cell cycle	biological_process	5	0	0	0	0	0	0	0	0	0	0	0	0
GO:0048065	male courtship behavior, veined wing extension	biological_process	3	0	0	0	0	0	0	0	0	0	0	0	0
GO:0048047	mating behavior, sex discrimination	biological_process	9	0	0	0	0	0	0	0	0	0	0	0	0
GO:0048036	central complex development	biological_process	10	0	0	0	0	0	0	0	0	0	0	0	0
GO:0051127	positive regulation of actin nucleation	biological_process	5	0	0	0	0	0	0	0	0	0	0	0	0

GO:0051156	glucose 6-phosphate metabolic process	biological_process	3	0	0	0	0	0	0	0	0	0	0	0	0
GO:0051180	vitamin transport	biological_process	3	0	0	0	0	0	0	0	0	0	0	0	0
GO:0048026	positive regulation of mRNA splicing, via spliceosome	biological_process	4	0	0	0	0	0	0	0	0	0	0	0	0
GO:0051260	protein homooligomerization	biological_process	12	0	0	0	0	0	0	0	0	0	0	0	0
GO:0048025	negative regulation of mRNA splicing, via spliceosome	biological_process	3	0	0	0	0	0	0	0	0	0	0	0	0
GO:0048024	regulation of mRNA splicing, via spliceosome	biological_process	19	0	0	0	0	0	0	0	0	0	0	0	0
GO:0048019	receptor antagonist activity	molecular_function	3	0	0	0	0	0	0	0	0	0	0	0	0
GO:0051287	NAD binding	molecular_function	31	0	0	0	0	0	0	0	0	0	0	0	0
GO:0051292	nuclear pore complex assembly	biological_process	4	0	0	0	0	0	0	0	0	0	0	0	0
GO:0048013	ephrin receptor signaling pathway	biological_process	4	0	0	0	0	0	0	0	0	0	0	0	0
GO:0051294	establishment of spindle orientation	biological_process	6	0	0	0	0	0	0	0	0	0	0	0	0
GO:0051299	centrosome separation	biological_process	14	0	0	0	0	0	0	0	0	0	0	0	0
GO:0051301	cell division	biological_process	11	0	0	0	0	0	0	0	0	0	0	0	0
GO:0051304	chromosome separation	biological_process	5	0	0	0	0	0	0	0	0	0	0	0	0
GO:0051438	regulation of ubiquitin-protein transferase activity	biological_process	3	0	0	0	0	0	0	0	0	0	0	0	0
GO:0046935	1-phosphatidylinositol-3-kinase regulator activity	molecular_function	4	0	0	0	0	0	0	0	0	0	0	0	0
GO:0046933	proton-transporting ATP synthase activity, rotational mechanism	molecular_function	18	0	0	0	0	0	0	0	0	0	0	0	0
GO:0071539	protein localization to centrosome	biological_process	3	0	0	0	0	0	0	0	0	0	0	0	0
GO:0046664	dorsal closure, amnioserosa morphology change	biological_process	14	0	0	0	0	0	0	0	0	0	0	0	0
GO:0046621	negative regulation of organ growth	biological_process	10	0	0	0	0	0	0	0	0	0	0	0	0
GO:0046620	regulation of organ growth	biological_process	11	0	0	0	0	0	0	0	0	0	0	0	0
GO:0046594	maintenance of pole plasm mRNA location	biological_process	3	0	0	0	0	0	0	0	0	0	0	0	0
GO:0060027	convergent extension involved in gastrulation	biological_process	4	0	0	0	0	0	0	0	0	0	0	0	0
GO:0046592	polyamine oxidase activity	molecular_function	6	0	0	0	0	0	0	0	0	0	0	0	0
GO:0060050	positive regulation of protein glycosylation	biological_process	3	0	0	0	0	0	0	0	0	0	0	0	0
GO:0060070	canonical Wnt signaling pathway	biological_process	13	0	0	0	0	0	0	0	0	0	0	0	0
GO:0046579	positive regulation of Ras protein signal transduction	biological_process	22	0	0	0	0	0	0	0	0	0	0	0	0
GO:0046530	photoreceptor cell differentiation	biological_process	9	0	0	0	0	0	0	0	0	0	0	0	0
GO:0060078	regulation of postsynaptic membrane potential	biological_process	3	0	0	0	0	0	0	0	0	0	0	0	0
GO:0060086	circadian temperature homeostasis	biological_process	4	0	0	0	0	0	0	0	0	0	0	0	0
GO:0046529	imaginal disc fusion, thorax closure	biological_process	21	0	0	0	0	0	0	0	0	0	0	0	0
GO:0060179	male mating behavior	biological_process	11	0	0	0	0	0	0	0	0	0	0	0	0
GO:0046513	ceramide biosynthetic process	biological_process	7	0	0	0	0	0	0	0	0	0	0	0	0
GO:0046475	glycerophospholipid catabolic process	biological_process	6	0	0	0	0	0	0	0	0	0	0	0	0
GO:0060213	positive regulation of nuclear-transcribed mRNA poly(A) tail shortening	biological_process	13	0	0	0	0	0	0	0	0	0	0	0	0
GO:0060250	germ-line stem-cell niche homeostasis	biological_process	19	0	0	0	0	0	0	0	0	0	0	0	0
GO:0046332	SMAD binding	molecular_function	3	0	0	0	0	0	0	0	0	0	0	0	0
GO:0060252	positive regulation of glial cell proliferation	biological_process	5	0	0	0	0	0	0	0	0	0	0	0	0
GO:0055093	response to hyperoxia	biological_process	14	0	0	0	0	0	0	0	0	0	0	0	0
GO:0055075	potassium ion homeostasis	biological_process	5	0	0	0	0	0	0	0	0	0	0	0	0
GO:0051646	mitochondrion localization	biological_process	7	0	0	0	0	0	0	0	0	0	0	0	0
GO:0055070	copper ion homeostasis	biological_process	14	0	0	0	0	0	0	0	0	0	0	0	0
GO:0046907	intracellular transport	biological_process	17	0	0	0	0	0	0	0	0	0	0	0	0
GO:0051647	nucleus localization	biological_process	8	0	0	0	0	0	0	0	0	0	0	0	0
GO:0051721	protein phosphatase 2A binding	molecular_function	3	0	0	0	0	0	0	0	0	0	0	0	0
GO:0051754	meiotic sister chromatid cohesion, centromeric	biological_process	4	0	0	0	0	0	0	0	0	0	0	0	0

GO:0051865	protein autoubiquitination	biological_process	16	0	0	0	0	0	0	0	0	0	0	0	0
GO:0046844	micropyle formation	biological_process	4	0	0	0	0	0	0	0	0	0	0	0	0
GO:0046835	carbohydrate phosphorylation	biological_process	10	0	0	0	0	0	0	0	0	0	0	0	0
GO:0046834	lipid phosphorylation	biological_process	6	0	0	0	0	0	0	0	0	0	0	0	0
GO:0051898	negative regulation of protein kinase B signaling	biological_process	7	0	0	0	0	0	0	0	0	0	0	0	0
GO:0051920	peroxiredoxin activity	molecular_function	4	0	0	0	0	0	0	0	0	0	0	0	0
GO:0051960	regulation of nervous system development	biological_process	4	0	0	0	0	0	0	0	0	0	0	0	0
GO:0046823	negative regulation of nucleocytoplasmic transport	biological_process	3	0	0	0	0	0	0	0	0	0	0	0	0
GO:0051998	protein carboxyl O-methyltransferase activity	molecular_function	4	0	0	0	0	0	0	0	0	0	0	0	0
GO:0046822	regulation of nucleocytoplasmic transport	biological_process	6	0	0	0	0	0	0	0	0	0	0	0	0
GO:0052720	apurinic/apyrimidinic endodeoxyribonuclease activity	molecular_function	3	0	0	0	0	0	0	0	0	0	0	0	0
GO:0055013	cardiac muscle cell development	biological_process	3	0	0	0	0	0	0	0	0	0	0	0	0
GO:0055064	chloride ion homeostasis	biological_process	5	0	0	0	0	0	0	0	0	0	0	0	0
GO:0046693	sperm storage	biological_process	14	0	0	0	0	0	0	0	0	0	0	0	0
GO:0046692	sperm competition	biological_process	18	0	0	0	0	0	0	0	0	0	0	0	0
GO:0051124	synaptic growth at neuromuscular junction	biological_process	35	0	0	0	0	0	0	0	0	0	0	0	0
GO:0048066	developmental pigmentation	biological_process	12	0	0	0	0	0	0	0	0	0	0	0	0
GO:0048072	compound eye pigmentation	biological_process	13	0	0	0	0	0	0	0	0	0	0	0	0
GO:0048617	embryonic foregut morphogenesis	biological_process	3	0	0	0	0	0	0	0	0	0	0	0	0
GO:0048856	anatomical structure development	biological_process	19	0	0	0	0	0	0	0	0	0	0	0	0
GO:0048589	developmental growth	biological_process	5	0	0	0	0	0	0	0	0	0	0	0	0
GO:0048865	stem cell fate commitment	biological_process	5	0	0	0	0	0	0	0	0	0	0	0	0
GO:0048935	peripheral nervous system neuron development	biological_process	8	0	0	0	0	0	0	0	0	0	0	0	0
GO:0048565	digestive tract development	biological_process	3	0	0	0	0	0	0	0	0	0	0	0	0
GO:0048531	beta-1,3-galactosyltransferase activity	molecular_function	5	0	0	0	0	0	0	0	0	0	0	0	0
GO:0048515	spermatid differentiation	biological_process	15	0	0	0	0	0	0	0	0	0	0	0	0
GO:0048513	animal organ development	biological_process	5	0	0	0	0	0	0	0	0	0	0	0	0
GO:0048512	circadian behavior	biological_process	9	0	0	0	0	0	0	0	0	0	0	0	0
GO:0050321	tau-protein kinase activity	molecular_function	5	0	0	0	0	0	0	0	0	0	0	0	0
GO:0050567	glutaminyl-tRNA synthase (glutamine-hydrolyzing) activity	molecular_function	3	0	0	0	0	0	0	0	0	0	0	0	0
GO:0050650	chondroitin sulfate proteoglycan biosynthetic process	biological_process	4	0	0	0	0	0	0	0	0	0	0	0	0
GO:0050661	NADP binding	molecular_function	14	0	0	0	0	0	0	0	0	0	0	0	0
GO:0050688	regulation of defense response to virus	biological_process	8	0	0	0	0	0	0	0	0	0	0	0	0
GO:0048499	synaptic vesicle membrane organization	biological_process	5	0	0	0	0	0	0	0	0	0	0	0	0
GO:0050714	positive regulation of protein secretion	biological_process	6	0	0	0	0	0	0	0	0	0	0	0	0
GO:0048490	anterograde synaptic vesicle transport	biological_process	4	0	0	0	0	0	0	0	0	0	0	0	0
GO:0048489	synaptic vesicle transport	biological_process	18	0	0	0	0	0	0	0	0	0	0	0	0
GO:0048487	beta-tubulin binding	molecular_function	6	0	0	0	0	0	0	0	0	0	0	0	0
GO:0048615	embryonic anterior midgut (ectodermal) morphogenesis	biological_process	4	0	0	0	0	0	0	0	0	0	0	0	0
GO:0048619	embryonic hindgut morphogenesis	biological_process	8	0	0	0	0	0	0	0	0	0	0	0	0
GO:0048076	regulation of compound eye pigmentation	biological_process	5	0	0	0	0	0	0	0	0	0	0	0	0
GO:0048846	axon extension involved in axon guidance	biological_process	7	0	0	0	0	0	0	0	0	0	0	0	0
GO:0048747	muscle fiber development	biological_process	10	0	0	0	0	0	0	0	0	0	0	0	0
GO:0048730	epidermis morphogenesis	biological_process	5	0	0	0	0	0	0	0	0	0	0	0	0
GO:0048728	proboscis development	biological_process	5	0	0	0	0	0	0	0	0	0	0	0	0
GO:0048699	generation of neurons	biological_process	4	0	0	0	0	0	0	0	0	0	0	0	0

GO:0048790	maintenance of presynaptic active zone structure	biological_process	9	0	0	0	0	0	0	0	0	0	0	0	0
GO:0048691	positive regulation of axon extension involved in regeneration	biological_process	4	0	0	0	0	0	0	0	0	0	0	0	0
GO:0048680	positive regulation of axon regeneration	biological_process	4	0	0	0	0	0	0	0	0	0	0	0	0
GO:0048674	collateral sprouting of injured axon	biological_process	3	0	0	0	0	0	0	0	0	0	0	0	0
GO:0048666	neuron development	biological_process	14	0	0	0	0	0	0	0	0	0	0	0	0
GO:0048802	notum morphogenesis	biological_process	5	0	0	0	0	0	0	0	0	0	0	0	0
GO:0048812	neuron projection morphogenesis	biological_process	17	0	0	0	0	0	0	0	0	0	0	0	0
GO:0048663	neuron fate commitment	biological_process	5	0	0	0	0	0	0	0	0	0	0	0	0
GO:0048644	muscle organ morphogenesis	biological_process	5	0	0	0	0	0	0	0	0	0	0	0	0
GO:0048814	regulation of dendrite morphogenesis	biological_process	20	0	0	0	0	0	0	0	0	0	0	0	0
GO:0048639	positive regulation of developmental growth	biological_process	3	0	0	0	0	0	0	0	0	0	0	0	0
GO:0048841	regulation of axon extension involved in axon guidance	biological_process	3	0	0	0	0	0	0	0	0	0	0	0	0
GO:0048636	positive regulation of muscle organ development	biological_process	6	0	0	0	0	0	0	0	0	0	0	0	0
GO:0048621	post-embryonic digestive tract morphogenesis	biological_process	3	0	0	0	0	0	0	0	0	0	0	0	0
GO:0048843	negative regulation of axon extension involved in axon guidance	biological_process	5	0	0	0	0	0	0	0	0	0	0	0	0
GO:0050764	regulation of phagocytosis	biological_process	4	0	0	0	0	0	0	0	0	0	0	0	0
GO:0048478	replication fork protection	biological_process	6	0	0	0	0	0	0	0	0	0	0	0	0
GO:0048388	endosomal lumen acidification	biological_process	3	0	0	0	0	0	0	0	0	0	0	0	0
GO:0050766	positive regulation of phagocytosis	biological_process	7	0	0	0	0	0	0	0	0	0	0	0	0
GO:0050974	detection of mechanical stimulus involved in sensory perception	biological_process	3	0	0	0	0	0	0	0	0	0	0	0	0
GO:0050975	sensory perception of touch	biological_process	10	0	0	0	0	0	0	0	0	0	0	0	0
GO:0050976	detection of mechanical stimulus involved in sensory perception of touch	biological_process	4	0	0	0	0	0	0	0	0	0	0	0	0
GO:0034453	microtubule anchoring	biological_process	6	0	0	0	0	0	0	0	0	0	0	0	0
GO:0048140	male germ-line cyst encapsulation	biological_process	8	0	0	0	0	0	0	0	0	0	0	0	0
GO:0048137	spermatocyte division	biological_process	15	0	0	0	0	0	0	0	0	0	0	0	0
GO:0048133	male germ-line stem cell asymmetric division	biological_process	7	0	0	0	0	0	0	0	0	0	0	0	0
GO:0050982	detection of mechanical stimulus	biological_process	6	0	0	0	0	0	0	0	0	0	0	0	0
GO:0048132	female germ-line stem cell asymmetric division	biological_process	18	0	0	0	0	0	0	0	0	0	0	0	0
GO:0051010	microtubule plus-end binding	molecular_function	10	0	0	0	0	0	0	0	0	0	0	0	0
GO:0051016	barbed-end actin filament capping	biological_process	4	0	0	0	0	0	0	0	0	0	0	0	0
GO:0048099	anterior/posterior lineage restriction, imaginal disc	biological_process	4	0	0	0	0	0	0	0	0	0	0	0	0
GO:0048098	antennal joint development	biological_process	4	0	0	0	0	0	0	0	0	0	0	0	0
GO:0048096	chromatin-mediated maintenance of transcription	biological_process	10	0	0	0	0	0	0	0	0	0	0	0	0
GO:0051018	protein kinase A binding	molecular_function	4	0	0	0	0	0	0	0	0	0	0	0	0
GO:0051039	positive regulation of transcription involved in meiotic cell cycle	biological_process	3	0	0	0	0	0	0	0	0	0	0	0	0
GO:0051087	chaperone binding	molecular_function	34	0	0	0	0	0	0	0	0	0	0	0	0
GO:0051092	positive regulation of NF-kappaB transcription factor activity	biological_process	5	0	0	0	0	0	0	0	0	0	0	0	0
GO:0048081	positive regulation of cuticle pigmentation	biological_process	3	0	0	0	0	0	0	0	0	0	0	0	0
GO:0048193	Golgi vesicle transport	biological_process	36	0	0	0	0	0	0	0	0	0	0	0	0
GO:0048227	plasma membrane to endosome transport	biological_process	5	0	0	0	0	0	0	0	0	0	0	0	0
GO:0050966	detection of mechanical stimulus involved in sensory perception of pain	biological_process	4	0	0	0	0	0	0	0	0	0	0	0	0
GO:0048278	vesicle docking	biological_process	10	0	0	0	0	0	0	0	0	0	0	0	0
GO:0050767	regulation of neurogenesis	biological_process	34	0	0	0	0	0	0	0	0	0	0	0	0
GO:0050769	positive regulation of neurogenesis	biological_process	4	0	0	0	0	0	0	0	0	0	0	0	0

GO:0050777	negative regulation of immune response	biological_process	5	0	0	0	0	0	0	0	0	0	0	0	0
GO:0050803	regulation of synapse structure or activity	biological_process	19	0	0	0	0	0	0	0	0	0	0	0	0
GO:0050805	negative regulation of synaptic transmission	biological_process	4	0	0	0	0	0	0	0	0	0	0	0	0
GO:0048311	mitochondrion distribution	biological_process	5	0	0	0	0	0	0	0	0	0	0	0	0
GO:0050807	regulation of synapse organization	biological_process	10	0	0	0	0	0	0	0	0	0	0	0	0
GO:0048285	organelle fission	biological_process	3	0	0	0	0	0	0	0	0	0	0	0	0
GO:0050821	protein stabilization	biological_process	39	0	0	0	0	0	0	0	0	0	0	0	0
GO:0050954	sensory perception of mechanical stimulus	biological_process	4	0	0	0	0	0	0	0	0	0	0	0	0
GO:0050884	neuromuscular process controlling posture	biological_process	4	0	0	0	0	0	0	0	0	0	0	0	0
GO:0050908	detection of light stimulus involved in visual perception	biological_process	7	0	0	0	0	0	0	0	0	0	0	0	0
GO:0048266	behavioral response to pain	biological_process	3	0	0	0	0	0	0	0	0	0	0	0	0
GO:0048260	positive regulation of receptor-mediated endocytosis	biological_process	10	0	0	0	0	0	0	0	0	0	0	0	0
GO:0050912	detection of chemical stimulus involved in sensory perception of taste	biological_process	20	0	0	0	0	0	0	0	0	0	0	0	0
GO:0050913	sensory perception of bitter taste	biological_process	5	0	0	0	0	0	0	0	0	0	0	0	0
GO:0048256	flap endonuclease activity	molecular_function	4	0	0	0	0	0	0	0	0	0	0	0	0
GO:0050914	sensory perception of salty taste	biological_process	3	0	0	0	0	0	0	0	0	0	0	0	0
GO:0060259	regulation of feeding behavior	biological_process	8	0	0	0	0	0	0	0	0	0	0	0	0
GO:0060261	positive regulation of transcription initiation from RNA polymerase II promoter	biological_process	7	0	0	0	0	0	0	0	0	0	0	0	0
GO:0060269	centripetally migrating follicle cell migration	biological_process	5	0	0	0	0	0	0	0	0	0	0	0	0
GO:0070584	mitochondrion morphogenesis	biological_process	9	0	0	0	0	0	0	0	0	0	0	0	0
GO:0070588	calcium ion transmembrane transport	biological_process	23	0	0	0	0	0	0	0	0	0	0	0	0
GO:0044877	macromolecular complex binding	molecular_function	11	0	0	0	0	0	0	0	0	0	0	0	0
GO:0044804	nucleophagy	biological_process	8	0	0	0	0	0	0	0	0	0	0	0	0
GO:0070593	dendrite self-avoidance	biological_process	18	0	0	0	0	0	0	0	0	0	0	0	0
GO:0070679	inositol 1,4,5 trisphosphate binding	molecular_function	4	0	0	0	0	0	0	0	0	0	0	0	0
GO:0044773	mitotic DNA damage checkpoint	biological_process	5	0	0	0	0	0	0	0	0	0	0	0	0
GO:0070681	glutaminyl-tRNAGln biosynthesis via transamidation	biological_process	3	0	0	0	0	0	0	0	0	0	0	0	0
GO:0070700	BMP receptor binding	molecular_function	3	0	0	0	0	0	0	0	0	0	0	0	0
GO:0044719	regulation of imaginal disc-derived wing size	biological_process	14	0	0	0	0	0	0	0	0	0	0	0	0
GO:0044571	[2Fe-2S] cluster assembly	biological_process	3	0	0	0	0	0	0	0	0	0	0	0	0
GO:0044458	motile cilium assembly	biological_process	4	0	0	0	0	0	0	0	0	0	0	0	0
GO:0048789	cytoskeletal matrix organization at active zone	biological_process	7	0	0	0	0	0	0	0	0	0	0	0	0
GO:0044331	cell-cell adhesion mediated by cadherin	biological_process	14	0	0	0	0	0	0	0	0	0	0	0	0
GO:0070735	protein-glycine ligase activity	molecular_function	11	0	0	0	0	0	0	0	0	0	0	0	0
GO:0044325	ion channel binding	molecular_function	7	0	0	0	0	0	0	0	0	0	0	0	0
GO:0070816	phosphorylation of RNA polymerase II C-terminal domain	biological_process	7	0	0	0	0	0	0	0	0	0	0	0	0
GO:0070828	heterochromatin organization	biological_process	7	0	0	0	0	0	0	0	0	0	0	0	0
GO:0043994	histone acetyltransferase activity (H3-K23 specific)	molecular_function	4	0	0	0	0	0	0	0	0	0	0	0	0
GO:0070831	basement membrane assembly	biological_process	6	0	0	0	0	0	0	0	0	0	0	0	0
GO:0045004	DNA replication proofreading	biological_process	5	0	0	0	0	0	0	0	0	0	0	0	0
GO:0045010	actin nucleation	biological_process	3	0	0	0	0	0	0	0	0	0	0	0	0
GO:0045316	negative regulation of compound eye photoreceptor development	biological_process	3	0	0	0	0	0	0	0	0	0	0	0	0
GO:0045022	early endosome to late endosome transport	biological_process	6	0	0	0	0	0	0	0	0	0	0	0	0
GO:0045216	cell-cell junction organization	biological_process	6	0	0	0	0	0	0	0	0	0	0	0	0
GO:0045213	neurotransmitter receptor metabolic process	biological_process	3	0	0	0	0	0	0	0	0	0	0	0	0

GO:0070481	nuclear-transcribed mRNA catabolic process, non-stop decay	biological_process	3	0	0	0	0	0	0	0	0	0	0	0	0
GO:0045198	establishment of epithelial cell apical/basal polarity	biological_process	11	0	0	0	0	0	0	0	0	0	0	0	0
GO:0045197	establishment or maintenance of epithelial cell apical/basal polarity	biological_process	17	0	0	0	0	0	0	0	0	0	0	0	0
GO:0045196	establishment or maintenance of neuroblast polarity	biological_process	11	0	0	0	0	0	0	0	0	0	0	0	0
GO:0045182	translation regulator activity	molecular_function	6	0	0	0	0	0	0	0	0	0	0	0	0
GO:0045176	apical protein localization	biological_process	17	0	0	0	0	0	0	0	0	0	0	0	0
GO:0045175	basal protein localization	biological_process	10	0	0	0	0	0	0	0	0	0	0	0	0
GO:0070509	calcium ion import	biological_process	3	0	0	0	0	0	0	0	0	0	0	0	0
GO:0045143	homologous chromosome segregation	biological_process	4	0	0	0	0	0	0	0	0	0	0	0	0
GO:0070530	K63-linked polyubiquitin binding	molecular_function	5	0	0	0	0	0	0	0	0	0	0	0	0
GO:0045132	meiotic chromosome segregation	biological_process	9	0	0	0	0	0	0	0	0	0	0	0	0
GO:0045116	protein neddylation	biological_process	11	0	0	0	0	0	0	0	0	0	0	0	0
GO:0070534	protein K63-linked ubiquitination	biological_process	7	0	0	0	0	0	0	0	0	0	0	0	0
GO:0045088	regulation of innate immune response	biological_process	13	0	0	0	0	0	0	0	0	0	0	0	0
GO:0070536	protein K63-linked deubiquitination	biological_process	4	0	0	0	0	0	0	0	0	0	0	0	0
GO:0045041	protein import into mitochondrial intermembrane space	biological_process	6	0	0	0	0	0	0	0	0	0	0	0	0
GO:0045035	sensory organ precursor cell division	biological_process	10	0	0	0	0	0	0	0	0	0	0	0	0
GO:0043984	histone H4-K16 acetylation	biological_process	7	0	0	0	0	0	0	0	0	0	0	0	0
GO:0043970	histone H3-K9 acetylation	biological_process	3	0	0	0	0	0	0	0	0	0	0	0	0
GO:0043968	histone H2A acetylation	biological_process	5	0	0	0	0	0	0	0	0	0	0	0	0
GO:0070840	dynein complex binding	molecular_function	5	0	0	0	0	0	0	0	0	0	0	0	0
GO:0043433	negative regulation of sequence-specific DNA binding transcription factor activity	biological_process	7	0	0	0	0	0	0	0	0	0	0	0	0
GO:0071276	cellular response to cadmium ion	biological_process	6	0	0	0	0	0	0	0	0	0	0	0	0
GO:0043410	positive regulation of MAPK cascade	biological_process	10	0	0	0	0	0	0	0	0	0	0	0	0
GO:0043280	positive regulation of cysteine-type endopeptidase activity involved in apoptotic process	biological_process	4	0	0	0	0	0	0	0	0	0	0	0	0
GO:0043277	apoptotic cell clearance	biological_process	9	0	0	0	0	0	0	0	0	0	0	0	0
GO:0043252	sodium-independent organic anion transport	biological_process	8	0	0	0	0	0	0	0	0	0	0	0	0
GO:0043171	peptide catabolic process	biological_process	24	0	0	0	0	0	0	0	0	0	0	0	0
GO:0071329	cellular response to sucrose stimulus	biological_process	7	0	0	0	0	0	0	0	0	0	0	0	0
GO:0071333	cellular response to glucose stimulus	biological_process	7	0	0	0	0	0	0	0	0	0	0	0	0
GO:0043153	entrainment of circadian clock by photoperiod	biological_process	11	0	0	0	0	0	0	0	0	0	0	0	0
GO:0043150	DNA synthesis involved in double-strand break repair via homologous recombination	biological_process	5	0	0	0	0	0	0	0	0	0	0	0	0
GO:0043138	3'-5' DNA helicase activity	molecular_function	20	0	0	0	0	0	0	0	0	0	0	0	0
GO:0071361	cellular response to ethanol	biological_process	9	0	0	0	0	0	0	0	0	0	0	0	0
GO:0071363	cellular response to growth factor stimulus	biological_process	5	0	0	0	0	0	0	0	0	0	0	0	0
GO:0071390	cellular response to ecdysone	biological_process	11	0	0	0	0	0	0	0	0	0	0	0	0
GO:0043122	regulation of I-kappaB kinase/NF-kappaB signaling	biological_process	3	0	0	0	0	0	0	0	0	0	0	0	0
GO:0043111	replication fork arrest	biological_process	3	0	0	0	0	0	0	0	0	0	0	0	0
GO:0071480	cellular response to gamma radiation	biological_process	12	0	0	0	0	0	0	0	0	0	0	0	0
GO:0071526	semaphorin-plexin signaling pathway	biological_process	4	0	0	0	0	0	0	0	0	0	0	0	0
GO:0071260	cellular response to mechanical stimulus	biological_process	7	0	0	0	0	0	0	0	0	0	0	0	0
GO:0071243	cellular response to arsenic-containing substance	biological_process	6	0	0	0	0	0	0	0	0	0	0	0	0
GO:0071230	cellular response to amino acid stimulus	biological_process	12	0	0	0	0	0	0	0	0	0	0	0	0

GO:0043621	protein self-association	molecular_function	3	0	0	0	0	0	0	0	0	0	0	0	0
GO:0043967	histone H4 acetylation	biological_process	14	0	0	0	0	0	0	0	0	0	0	0	0
GO:0043966	histone H3 acetylation	biological_process	28	0	0	0	0	0	0	0	0	0	0	0	0
GO:0070868	heterochromatin organization involved in chromatin silencing	biological_process	27	0	0	0	0	0	0	0	0	0	0	0	0
GO:0043703	photoreceptor cell fate determination	biological_process	6	0	0	0	0	0	0	0	0	0	0	0	0
GO:0070891	lipoteichoic acid binding	molecular_function	3	0	0	0	0	0	0	0	0	0	0	0	0
GO:0043695	detection of pheromone	biological_process	14	0	0	0	0	0	0	0	0	0	0	0	0
GO:0043666	regulation of phosphoprotein phosphatase activity	biological_process	6	0	0	0	0	0	0	0	0	0	0	0	0
GO:0070898	RNA polymerase III transcriptional preinitiation complex assembly	biological_process	6	0	0	0	0	0	0	0	0	0	0	0	0
GO:0070914	UV-damage excision repair	biological_process	3	0	0	0	0	0	0	0	0	0	0	0	0
GO:0043491	protein kinase B signaling	biological_process	3	0	0	0	0	0	0	0	0	0	0	0	0
GO:0070932	histone H3 deacetylation	biological_process	5	0	0	0	0	0	0	0	0	0	0	0	0
GO:0070936	protein K48-linked ubiquitination	biological_process	10	0	0	0	0	0	0	0	0	0	0	0	0
GO:0070940	dephosphorylation of RNA polymerase II C-terminal domain	biological_process	3	0	0	0	0	0	0	0	0	0	0	0	0
GO:0043531	ADP binding	molecular_function	4	0	0	0	0	0	0	0	0	0	0	0	0
GO:0071051	polyadenylation-dependent snoRNA 3'-end processing	biological_process	6	0	0	0	0	0	0	0	0	0	0	0	0
GO:0071108	protein K48-linked deubiquitination	biological_process	3	0	0	0	0	0	0	0	0	0	0	0	0
GO:0043508	negative regulation of JUN kinase activity	biological_process	3	0	0	0	0	0	0	0	0	0	0	0	0
GO:0043495	protein anchor	molecular_function	3	0	0	0	0	0	0	0	0	0	0	0	0
GO:0045296	cadherin binding	molecular_function	12	0	0	0	0	0	0	0	0	0	0	0	0
GO:0070273	phosphatidylinositol-4-phosphate binding	molecular_function	6	0	0	0	0	0	0	0	0	0	0	0	0
GO:0060285	cilium-dependent cell motility	biological_process	7	0	0	0	0	0	0	0	0	0	0	0	0
GO:0045926	negative regulation of growth	biological_process	15	0	0	0	0	0	0	0	0	0	0	0	0
GO:0045910	negative regulation of DNA recombination	biological_process	7	0	0	0	0	0	0	0	0	0	0	0	0
GO:0060914	heart formation	biological_process	6	0	0	0	0	0	0	0	0	0	0	0	0
GO:0060968	regulation of gene silencing	biological_process	9	0	0	0	0	0	0	0	0	0	0	0	0
GO:0061024	membrane organization	biological_process	15	0	0	0	0	0	0	0	0	0	0	0	0
GO:0061057	peptidoglycan recognition protein signaling pathway	biological_process	17	0	0	0	0	0	0	0	0	0	0	0	0
GO:0061059	positive regulation of peptidoglycan recognition protein signaling pathway	biological_process	10	0	0	0	0	0	0	0	0	0	0	0	0
GO:0061060	negative regulation of peptidoglycan recognition protein signaling pathway	biological_process	19	0	0	0	0	0	0	0	0	0	0	0	0
GO:0045886	negative regulation of synaptic growth at neuromuscular junction	biological_process	42	0	0	0	0	0	0	0	0	0	0	0	0
GO:0045880	positive regulation of smoothened signaling pathway	biological_process	46	0	0	0	0	0	0	0	0	0	0	0	0
GO:0045874	positive regulation of sevenless signaling pathway	biological_process	8	0	0	0	0	0	0	0	0	0	0	0	0
GO:0061077	chaperone-mediated protein folding	biological_process	23	0	0	0	0	0	0	0	0	0	0	0	0
GO:0045861	negative regulation of proteolysis	biological_process	21	0	0	0	0	0	0	0	0	0	0	0	0
GO:0045860	positive regulation of protein kinase activity	biological_process	6	0	0	0	0	0	0	0	0	0	0	0	0
GO:0045842	positive regulation of mitotic metaphase/anaphase transition	biological_process	12	0	0	0	0	0	0	0	0	0	0	0	0
GO:0061086	negative regulation of histone H3-K27 methylation	biological_process	4	0	0	0	0	0	0	0	0	0	0	0	0
GO:0061088	regulation of sequestering of zinc ion	biological_process	3	0	0	0	0	0	0	0	0	0	0	0	0
GO:0061101	neuroendocrine cell differentiation	biological_process	6	0	0	0	0	0	0	0	0	0	0	0	0
GO:0045823	positive regulation of heart contraction	biological_process	5	0	0	0	0	0	0	0	0	0	0	0	0
GO:0045819	positive regulation of glycogen catabolic process	biological_process	3	0	0	0	0	0	0	0	0	0	0	0	0
GO:0045924	regulation of female receptivity	biological_process	10	0	0	0	0	0	0	0	0	0	0	0	0
GO:0060857	establishment of glial blood-brain barrier	biological_process	19	0	0	0	0	0	0	0	0	0	0	0	0

GO:0045317	equator specification	biological_process	6	0	0	0	0	0	0	0	0	0	0	0	0
GO:0060628	regulation of ER to Golgi vesicle-mediated transport	biological_process	3	0	0	0	0	0	0	0	0	0	0	0	0
GO:0060294	cilium movement involved in cell motility	biological_process	5	0	0	0	0	0	0	0	0	0	0	0	0
GO:0046323	glucose import	biological_process	20	0	0	0	0	0	0	0	0	0	0	0	0
GO:0046168	glycerol-3-phosphate catabolic process	biological_process	3	0	0	0	0	0	0	0	0	0	0	0	0
GO:0046068	cGMP metabolic process	biological_process	3	0	0	0	0	0	0	0	0	0	0	0	0
GO:0046058	cAMP metabolic process	biological_process	3	0	0	0	0	0	0	0	0	0	0	0	0
GO:0046034	ATP metabolic process	biological_process	9	0	0	0	0	0	0	0	0	0	0	0	0
GO:0046011	regulation of oskar mRNA translation	biological_process	7	0	0	0	0	0	0	0	0	0	0	0	0
GO:0060323	head morphogenesis	biological_process	5	0	0	0	0	0	0	0	0	0	0	0	0
GO:0045998	positive regulation of ecdysteroid biosynthetic process	biological_process	6	0	0	0	0	0	0	0	0	0	0	0	0
GO:0045968	negative regulation of juvenile hormone biosynthetic process	biological_process	3	0	0	0	0	0	0	0	0	0	0	0	0
GO:0060391	positive regulation of SMAD protein import into nucleus	biological_process	3	0	0	0	0	0	0	0	0	0	0	0	0
GO:0045950	negative regulation of mitotic recombination	biological_process	3	0	0	0	0	0	0	0	0	0	0	0	0
GO:0045947	negative regulation of translational initiation	biological_process	8	0	0	0	0	0	0	0	0	0	0	0	0
GO:0045945	positive regulation of transcription from RNA polymerase III promoter	biological_process	3	0	0	0	0	0	0	0	0	0	0	0	0
GO:0060395	SMAD protein signal transduction	biological_process	13	0	0	0	0	0	0	0	0	0	0	0	0
GO:0045943	positive regulation of transcription from RNA polymerase I promoter	biological_process	3	0	0	0	0	0	0	0	0	0	0	0	0
GO:0045938	positive regulation of circadian sleep/wake cycle, sleep	biological_process	17	0	0	0	0	0	0	0	0	0	0	0	0
GO:0060537	muscle tissue development	biological_process	5	0	0	0	0	0	0	0	0	0	0	0	0
GO:0045927	positive regulation of growth	biological_process	13	0	0	0	0	0	0	0	0	0	0	0	0
GO:0045807	positive regulation of endocytosis	biological_process	13	0	0	0	0	0	0	0	0	0	0	0	0
GO:0045786	negative regulation of cell cycle	biological_process	4	0	0	0	0	0	0	0	0	0	0	0	0
GO:0061330	Malpighian tubule stellate cell differentiation	biological_process	3	0	0	0	0	0	0	0	0	0	0	0	0
GO:0061331	epithelial cell proliferation involved in Malpighian tubule morphogenesis	biological_process	11	0	0	0	0	0	0	0	0	0	0	0	0
GO:0045500	sevenless signaling pathway	biological_process	17	0	0	0	0	0	0	0	0	0	0	0	0
GO:0045499	chemorepellent activity	molecular_function	4	0	0	0	0	0	0	0	0	0	0	0	0
GO:0045498	sex comb development	biological_process	11	0	0	0	0	0	0	0	0	0	0	0	0
GO:0061817	endoplasmic reticulum-plasma membrane tethering	biological_process	5	0	0	0	0	0	0	0	0	0	0	0	0
GO:0045494	photoreceptor cell maintenance	biological_process	16	0	0	0	0	0	0	0	0	0	0	0	0
GO:0045478	fusome organization	biological_process	8	0	0	0	0	0	0	0	0	0	0	0	0
GO:0045477	regulation of nurse cell apoptotic process	biological_process	3	0	0	0	0	0	0	0	0	0	0	0	0
GO:0045467	R7 cell development	biological_process	15	0	0	0	0	0	0	0	0	0	0	0	0
GO:0045463	R8 cell development	biological_process	4	0	0	0	0	0	0	0	0	0	0	0	0
GO:0070006	metalloaminopeptidase activity	molecular_function	23	0	0	0	0	0	0	0	0	0	0	0	0
GO:0045456	ecdysteroid biosynthetic process	biological_process	6	0	0	0	0	0	0	0	0	0	0	0	0
GO:0070034	telomerase RNA binding	molecular_function	3	0	0	0	0	0	0	0	0	0	0	0	0
GO:0070125	mitochondrial translational elongation	biological_process	4	0	0	0	0	0	0	0	0	0	0	0	0
GO:0045451	pole plasm oskar mRNA localization	biological_process	43	0	0	0	0	0	0	0	0	0	0	0	0
GO:0045448	mitotic cell cycle, embryonic	biological_process	10	0	0	0	0	0	0	0	0	0	0	0	0
GO:0070129	regulation of mitochondrial translation	biological_process	3	0	0	0	0	0	0	0	0	0	0	0	0
GO:0070181	small ribosomal subunit rRNA binding	molecular_function	8	0	0	0	0	0	0	0	0	0	0	0	0
GO:0070182	DNA polymerase binding	molecular_function	8	0	0	0	0	0	0	0	0	0	0	0	0
GO:0070193	synaptonemal complex organization	biological_process	5	0	0	0	0	0	0	0	0	0	0	0	0
GO:0061631	ubiquitin conjugating enzyme activity	molecular_function	31	0	0	0	0	0	0	0	0	0	0	0	0
GO:0045503	dynein light chain binding	molecular_function	14	0	0	0	0	0	0	0	0	0	0	0	0
GO:0045504	dynein heavy chain binding	molecular_function	19	0	0	0	0	0	0	0	0	0	0	0	0
GO:0045736	negative regulation of cyclin-dependent protein serine/threonine kinase activity	biological_process	3	0	0	0	0	0	0	0	0	0	0	0	0

GO:0061343	cell adhesion involved in heart morphogenesis	biological_process	12	0	0	0	0	0	0	0	0	0	0	0	0
GO:0061357	positive regulation of Wnt protein secretion	biological_process	10	0	0	0	0	0	0	0	0	0	0	0	0
GO:0061382	Malpighian tubule tip cell differentiation	biological_process	9	0	0	0	0	0	0	0	0	0	0	0	0
GO:0045743	positive regulation of fibroblast growth factor receptor signaling pathway	biological_process	7	0	0	0	0	0	0	0	0	0	0	0	0
GO:0045742	positive regulation of epidermal growth factor receptor signaling pathway	biological_process	12	0	0	0	0	0	0	0	0	0	0	0	0
GO:0045740	positive regulation of DNA replication	biological_process	3	0	0	0	0	0	0	0	0	0	0	0	0
GO:0061572	actin filament bundle organization	biological_process	4	0	0	0	0	0	0	0	0	0	0	0	0
GO:0045737	positive regulation of cyclin-dependent protein serine/threonine kinase activity	biological_process	4	0	0	0	0	0	0	0	0	0	0	0	0
GO:0045732	positive regulation of protein catabolic process	biological_process	18	0	0	0	0	0	0	0	0	0	0	0	0
GO:0045571	negative regulation of imaginal disc growth	biological_process	17	0	0	0	0	0	0	0	0	0	0	0	0
GO:0061575	cyclin-dependent protein serine/threonine kinase activator activity	molecular_function	6	0	0	0	0	0	0	0	0	0	0	0	0
GO:0045705	negative regulation of salivary gland boundary specification	biological_process	8	0	0	0	0	0	0	0	0	0	0	0	0
GO:0061578	Lys63-specific deubiquitinase activity	molecular_function	8	0	0	0	0	0	0	0	0	0	0	0	0
GO:0045678	positive regulation of R7 cell differentiation	biological_process	3	0	0	0	0	0	0	0	0	0	0	0	0
GO:0061608	nuclear import signal receptor activity	molecular_function	11	0	0	0	0	0	0	0	0	0	0	0	0
GO:0045666	positive regulation of neuron differentiation	biological_process	9	0	0	0	0	0	0	0	0	0	0	0	0
GO:0045611	negative regulation of hemocyte differentiation	biological_process	5	0	0	0	0	0	0	0	0	0	0	0	0
GO:0045595	regulation of cell differentiation	biological_process	9	0	0	0	0	0	0	0	0	0	0	0	0
GO:0000001	mitochondrion inheritance	biological_process	3	0	0	0	0	0	0	0	0	0	0	0	0
GO:0018216	peptidyl-arginine methylation	biological_process	6	0	0	0	0	0	0	0	0	0	0	0	0
GO:0034450	ubiquitin-ubiquitin ligase activity	molecular_function	5	0	0	0	0	0	0	0	0	0	0	0	0
GO:0006623	protein targeting to vacuole	biological_process	11	0	0	0	0	0	0	0	0	0	0	0	0
GO:0006726	eye pigment biosynthetic process	biological_process	8	0	0	0	0	0	0	0	0	0	0	0	0
GO:0006723	cuticle hydrocarbon biosynthetic process	biological_process	4	0	0	0	0	0	0	0	0	0	0	0	0
GO:0006688	glycosphingolipid biosynthetic process	biological_process	8	0	0	0	0	0	0	0	0	0	0	0	0
GO:0006672	ceramide metabolic process	biological_process	3	0	0	0	0	0	0	0	0	0	0	0	0
GO:0006662	glycerol ether metabolic process	biological_process	6	0	0	0	0	0	0	0	0	0	0	0	0
GO:0006654	phosphatidic acid biosynthetic process	biological_process	4	0	0	0	0	0	0	0	0	0	0	0	0
GO:0006650	glycerophospholipid metabolic process	biological_process	3	0	0	0	0	0	0	0	0	0	0	0	0
GO:0006646	phosphatidylethanolamine biosynthetic process	biological_process	6	0	0	0	0	0	0	0	0	0	0	0	0
GO:0006641	triglyceride metabolic process	biological_process	9	0	0	0	0	0	0	0	0	0	0	0	0
GO:0006637	acyl-CoA metabolic process	biological_process	6	0	0	0	0	0	0	0	0	0	0	0	0
GO:0006635	fatty acid beta-oxidation	biological_process	28	0	0	0	0	0	0	0	0	0	0	0	0
GO:0006631	fatty acid metabolic process	biological_process	13	0	0	0	0	0	0	0	0	0	0	0	0
GO:0006620	posttranslational protein targeting to membrane	biological_process	3	0	0	0	0	0	0	0	0	0	0	0	0
GO:0006728	pteridine biosynthetic process	biological_process	4	0	0	0	0	0	0	0	0	0	0	0	0
GO:0006616	SRP-dependent cotranslational protein targeting to membrane, translocation	biological_process	7	0	0	0	0	0	0	0	0	0	0	0	0
GO:0006612	protein targeting to membrane	biological_process	25	0	0	0	0	0	0	0	0	0	0	0	0
GO:0006611	protein export from nucleus	biological_process	12	0	0	0	0	0	0	0	0	0	0	0	0
GO:0006605	protein targeting	biological_process	4	0	0	0	0	0	0	0	0	0	0	0	0
GO:0006584	catecholamine metabolic process	biological_process	4	0	0	0	0	0	0	0	0	0	0	0	0
GO:0006559	L-phenylalanine catabolic process	biological_process	6	0	0	0	0	0	0	0	0	0	0	0	0
GO:0006552	leucine catabolic process	biological_process	3	0	0	0	0	0	0	0	0	0	0	0	0
GO:0006551	leucine metabolic process	biological_process	3	0	0	0	0	0	0	0	0	0	0	0	0
GO:0006546	glycine catabolic process	biological_process	3	0	0	0	0	0	0	0	0	0	0	0	0

GO:0006537	glutamate biosynthetic process	biological_process	5	0	0	0	0	0	0	0	0	0	0	0	0
GO:0006520	cellular amino acid metabolic process	biological_process	15	0	0	0	0	0	0	0	0	0	0	0	0
GO:0006513	protein monoubiquitination	biological_process	8	0	0	0	0	0	0	0	0	0	0	0	0
GO:0006727	ommochrome biosynthetic process	biological_process	20	0	0	0	0	0	0	0	0	0	0	0	0
GO:0006729	tetrahydrobiopterin biosynthetic process	biological_process	6	0	0	0	0	0	0	0	0	0	0	0	0
GO:0034394	protein localization to cell surface	biological_process	5	0	0	0	0	0	0	0	0	0	0	0	0
GO:0006874	cellular calcium ion homeostasis	biological_process	18	0	0	0	0	0	0	0	0	0	0	0	0
GO:0006906	vesicle fusion	biological_process	21	0	0	0	0	0	0	0	0	0	0	0	0
GO:0006904	vesicle docking involved in exocytosis	biological_process	20	0	0	0	0	0	0	0	0	0	0	0	0
GO:0006896	Golgi to vacuole transport	biological_process	6	0	0	0	0	0	0	0	0	0	0	0	0
GO:0006895	Golgi to endosome transport	biological_process	4	0	0	0	0	0	0	0	0	0	0	0	0
GO:0006893	Golgi to plasma membrane transport	biological_process	10	0	0	0	0	0	0	0	0	0	0	0	0
GO:0006892	post-Golgi vesicle-mediated transport	biological_process	5	0	0	0	0	0	0	0	0	0	0	0	0
GO:0006887	exocytosis	biological_process	31	0	0	0	0	0	0	0	0	0	0	0	0
GO:0006884	cell volume homeostasis	biological_process	7	0	0	0	0	0	0	0	0	0	0	0	0
GO:0006883	cellular sodium ion homeostasis	biological_process	8	0	0	0	0	0	0	0	0	0	0	0	0
GO:0006882	cellular zinc ion homeostasis	biological_process	7	0	0	0	0	0	0	0	0	0	0	0	0
GO:0006879	cellular iron ion homeostasis	biological_process	9	0	0	0	0	0	0	0	0	0	0	0	0
GO:0006878	cellular copper ion homeostasis	biological_process	7	0	0	0	0	0	0	0	0	0	0	0	0
GO:0006865	amino acid transport	biological_process	3	0	0	0	0	0	0	0	0	0	0	0	0
GO:0006730	one-carbon metabolic process	biological_process	8	0	0	0	0	0	0	0	0	0	0	0	0
GO:0006850	mitochondrial pyruvate transport	biological_process	4	0	0	0	0	0	0	0	0	0	0	0	0
GO:0006844	acyl carnitine transport	biological_process	4	0	0	0	0	0	0	0	0	0	0	0	0
GO:0006839	mitochondrial transport	biological_process	15	0	0	0	0	0	0	0	0	0	0	0	0
GO:0006836	neurotransmitter transport	biological_process	21	0	0	0	0	0	0	0	0	0	0	0	0
GO:0006829	zinc II ion transport	biological_process	3	0	0	0	0	0	0	0	0	0	0	0	0
GO:0006828	manganese ion transport	biological_process	3	0	0	0	0	0	0	0	0	0	0	0	0
GO:0006825	copper ion transport	biological_process	5	0	0	0	0	0	0	0	0	0	0	0	0
GO:0006817	phosphate ion transport	biological_process	6	0	0	0	0	0	0	0	0	0	0	0	0
GO:0006813	potassium ion transport	biological_process	19	0	0	0	0	0	0	0	0	0	0	0	0
GO:0006811	ion transport	biological_process	14	0	0	0	0	0	0	0	0	0	0	0	0
GO:0006783	heme biosynthetic process	biological_process	12	0	0	0	0	0	0	0	0	0	0	0	0
GO:0006777	Mo-molybdopterin cofactor biosynthetic process	biological_process	6	0	0	0	0	0	0	0	0	0	0	0	0
GO:0006493	protein O-linked glycosylation	biological_process	21	0	0	0	0	0	0	0	0	0	0	0	0
GO:0006491	N-glycan processing	biological_process	16	0	0	0	0	0	0	0	0	0	0	0	0
GO:0006486	protein glycosylation	biological_process	35	0	0	0	0	0	0	0	0	0	0	0	0
GO:0006182	cGMP biosynthetic process	biological_process	12	0	0	0	0	0	0	0	0	0	0	0	0
GO:0006298	mismatch repair	biological_process	12	0	0	0	0	0	0	0	0	0	0	0	0
GO:0006296	nucleotide-excision repair, DNA incision, 5'-to lesion	biological_process	4	0	0	0	0	0	0	0	0	0	0	0	0
GO:0006289	nucleotide-excision repair	biological_process	22	0	0	0	0	0	0	0	0	0	0	0	0
GO:0006285	base-excision repair, AP site formation	biological_process	3	0	0	0	0	0	0	0	0	0	0	0	0
GO:0006284	base-excision repair	biological_process	8	0	0	0	0	0	0	0	0	0	0	0	0
GO:0006282	regulation of DNA repair	biological_process	3	0	0	0	0	0	0	0	0	0	0	0	0
GO:0006275	regulation of DNA replication	biological_process	5	0	0	0	0	0	0	0	0	0	0	0	0
GO:0006271	DNA strand elongation involved in DNA replication	biological_process	6	0	0	0	0	0	0	0	0	0	0	0	0
GO:0006270	DNA replication initiation	biological_process	15	0	0	0	0	0	0	0	0	0	0	0	0
GO:0006265	DNA topological change	biological_process	7	0	0	0	0	0	0	0	0	0	0	0	0
GO:0006264	mitochondrial DNA replication	biological_process	4	0	0	0	0	0	0	0	0	0	0	0	0

GO:0006260	DNA replication	biological_process	27	0	0	0	0	0	0	0	0	0	0	0	0
GO:0006171	cAMP biosynthetic process	biological_process	5	0	0	0	0	0	0	0	0	0	0	0	0
GO:0006479	protein methylation	biological_process	6	0	0	0	0	0	0	0	0	0	0	0	0
GO:0006164	purine nucleotide biosynthetic process	biological_process	6	0	0	0	0	0	0	0	0	0	0	0	0
GO:0006144	purine nucleobase metabolic process	biological_process	3	0	0	0	0	0	0	0	0	0	0	0	0
GO:0006139	nucleobase-containing compound metabolic process	biological_process	8	0	0	0	0	0	0	0	0	0	0	0	0
GO:0006123	mitochondrial electron transport, cytochrome c to oxygen	biological_process	17	0	0	0	0	0	0	0	0	0	0	0	0
GO:0006120	mitochondrial electron transport, NADH to ubiquinone	biological_process	37	0	0	0	0	0	0	0	0	0	0	0	0
GO:0006119	oxidative phosphorylation	biological_process	6	0	0	0	0	0	0	0	0	0	0	0	0
GO:0006117	acetaldehyde metabolic process	biological_process	5	0	0	0	0	0	0	0	0	0	0	0	0
GO:0006116	NADH oxidation	biological_process	6	0	0	0	0	0	0	0	0	0	0	0	0
GO:0006106	fumarate metabolic process	biological_process	4	0	0	0	0	0	0	0	0	0	0	0	0
GO:0006096	glycolytic process	biological_process	25	0	0	0	0	0	0	0	0	0	0	0	0
GO:0006094	gluconeogenesis	biological_process	9	0	0	0	0	0	0	0	0	0	0	0	0
GO:0006091	generation of precursor metabolites and energy	biological_process	4	0	0	0	0	0	0	0	0	0	0	0	0
GO:0006301	postreplication repair	biological_process	5	0	0	0	0	0	0	0	0	0	0	0	0
GO:0006303	double-strand break repair via nonhomologous end joining	biological_process	7	0	0	0	0	0	0	0	0	0	0	0	0
GO:0006309	apoptotic DNA fragmentation	biological_process	18	0	0	0	0	0	0	0	0	0	0	0	0
GO:0006333	chromatin assembly or disassembly	biological_process	28	0	0	0	0	0	0	0	0	0	0	0	0
GO:0006469	negative regulation of protein kinase activity	biological_process	6	0	0	0	0	0	0	0	0	0	0	0	0
GO:0006465	signal peptide processing	biological_process	7	0	0	0	0	0	0	0	0	0	0	0	0
GO:0006464	cellular protein modification process	biological_process	12	0	0	0	0	0	0	0	0	0	0	0	0
GO:0006458	'de novo' protein folding	biological_process	5	0	0	0	0	0	0	0	0	0	0	0	0
GO:0006450	regulation of translational fidelity	biological_process	4	0	0	0	0	0	0	0	0	0	0	0	0
GO:0006446	regulation of translational initiation	biological_process	11	0	0	0	0	0	0	0	0	0	0	0	0
GO:0006434	seryl-tRNA aminoacylation	biological_process	3	0	0	0	0	0	0	0	0	0	0	0	0
GO:0006419	alanyl-tRNA aminoacylation	biological_process	3	0	0	0	0	0	0	0	0	0	0	0	0
GO:0006418	tRNA aminoacylation for protein translation	biological_process	5	0	0	0	0	0	0	0	0	0	0	0	0
GO:0006415	translational termination	biological_process	5	0	0	0	0	0	0	0	0	0	0	0	0
GO:0006414	translational elongation	biological_process	15	0	0	0	0	0	0	0	0	0	0	0	0
GO:0006406	mRNA export from nucleus	biological_process	21	0	0	0	0	0	0	0	0	0	0	0	0
GO:0006401	RNA catabolic process	biological_process	8	0	0	0	0	0	0	0	0	0	0	0	0
GO:0006396	RNA processing	biological_process	22	0	0	0	0	0	0	0	0	0	0	0	0
GO:0006390	transcription from mitochondrial promoter	biological_process	8	0	0	0	0	0	0	0	0	0	0	0	0
GO:0006388	tRNA splicing, via endonucleolytic cleavage and ligation	biological_process	6	0	0	0	0	0	0	0	0	0	0	0	0
GO:0006383	transcription from RNA polymerase III promoter	biological_process	15	0	0	0	0	0	0	0	0	0	0	0	0
GO:0006382	adenosine to inosine editing	biological_process	5	0	0	0	0	0	0	0	0	0	0	0	0
GO:0006376	mRNA splice site selection	biological_process	7	0	0	0	0	0	0	0	0	0	0	0	0
GO:0006366	transcription from RNA polymerase II promoter	biological_process	60	0	0	0	0	0	0	0	0	0	0	0	0
GO:0006364	rRNA processing	biological_process	40	0	0	0	0	0	0	0	0	0	0	0	0
GO:0006360	transcription from RNA polymerase I promoter	biological_process	5	0	0	0	0	0	0	0	0	0	0	0	0
GO:0006342	chromatin silencing	biological_process	39	0	0	0	0	0	0	0	0	0	0	0	0
GO:0006336	DNA replication-independent nucleosome assembly	biological_process	4	0	0	0	0	0	0	0	0	0	0	0	0
GO:0006334	nucleosome assembly	biological_process	89	0	0	0	0	0	0	0	0	0	0	0	0

GO:0006909	phagocytosis	biological_process	23	0	0	0	0	0	0	0	0	0	0	0	0
GO:0006919	activation of cysteine-type endopeptidase activity involved in apoptotic process	biological_process	16	0	0	0	0	0	0	0	0	0	0	0	0
GO:0006930	substrate-dependent cell migration, cell extension	biological_process	3	0	0	0	0	0	0	0	0	0	0	0	0
GO:0007314	oocyte anterior/posterior axis specification	biological_process	21	0	0	0	0	0	0	0	0	0	0	0	0
GO:0007395	dorsal closure, spreading of leading edge cells	biological_process	9	0	0	0	0	0	0	0	0	0	0	0	0
GO:0007394	dorsal closure, elongation of leading edge cells	biological_process	10	0	0	0	0	0	0	0	0	0	0	0	0
GO:0007389	pattern specification process	biological_process	8	0	0	0	0	0	0	0	0	0	0	0	0
GO:0007384	specification of segmental identity, thorax	biological_process	3	0	0	0	0	0	0	0	0	0	0	0	0
GO:0007380	specification of segmental identity, head	biological_process	6	0	0	0	0	0	0	0	0	0	0	0	0
GO:0007378	amnioserosa formation	biological_process	8	0	0	0	0	0	0	0	0	0	0	0	0
GO:0007370	ventral furrow formation	biological_process	19	0	0	0	0	0	0	0	0	0	0	0	0
GO:0007343	egg activation	biological_process	15	0	0	0	0	0	0	0	0	0	0	0	0
GO:0007319	negative regulation of oskar mRNA translation	biological_process	8	0	0	0	0	0	0	0	0	0	0	0	0
GO:0007318	pole plasm protein localization	biological_process	12	0	0	0	0	0	0	0	0	0	0	0	0
GO:0007316	pole plasm RNA localization	biological_process	4	0	0	0	0	0	0	0	0	0	0	0	0
GO:0007315	pole plasm assembly	biological_process	17	0	0	0	0	0	0	0	0	0	0	0	0
GO:0007312	oocyte nucleus migration involved in oocyte dorsal/ventral axis specification	biological_process	7	0	0	0	0	0	0	0	0	0	0	0	0
GO:0007279	pole cell formation	biological_process	23	0	0	0	0	0	0	0	0	0	0	0	0
GO:0007311	maternal specification of dorsal/ventral axis, oocyte, germ-line encoded	biological_process	5	0	0	0	0	0	0	0	0	0	0	0	0
GO:0007310	oocyte dorsal/ventral axis specification	biological_process	19	0	0	0	0	0	0	0	0	0	0	0	0
GO:0007307	eggshell chorion gene amplification	biological_process	22	0	0	0	0	0	0	0	0	0	0	0	0
GO:0007306	eggshell chorion assembly	biological_process	17	0	0	0	0	0	0	0	0	0	0	0	0
GO:0007305	vitelline membrane formation involved in chorion-containing eggshell formation	biological_process	14	0	0	0	0	0	0	0	0	0	0	0	0
GO:0007303	cytoplasmic transport, nurse cell to oocyte	biological_process	13	0	0	0	0	0	0	0	0	0	0	0	0
GO:0007296	vitellogenesis	biological_process	3	0	0	0	0	0	0	0	0	0	0	0	0
GO:0007294	germarium-derived oocyte fate determination	biological_process	27	0	0	0	0	0	0	0	0	0	0	0	0
GO:0007289	spermatid nucleus differentiation	biological_process	5	0	0	0	0	0	0	0	0	0	0	0	0
GO:0007287	Nebenkern assembly	biological_process	5	0	0	0	0	0	0	0	0	0	0	0	0
GO:0007286	spermatid development	biological_process	26	0	0	0	0	0	0	0	0	0	0	0	0
GO:0007285	primary spermatocyte growth	biological_process	4	0	0	0	0	0	0	0	0	0	0	0	0
GO:0007398	ectoderm development	biological_process	7	0	0	0	0	0	0	0	0	0	0	0	0
GO:0007403	glial cell fate determination	biological_process	3	0	0	0	0	0	0	0	0	0	0	0	0
GO:0007412	axon target recognition	biological_process	13	0	0	0	0	0	0	0	0	0	0	0	0
GO:0007413	axonal fasciculation	biological_process	17	0	0	0	0	0	0	0	0	0	0	0	0
GO:0007507	heart development	biological_process	40	0	0	0	0	0	0	0	0	0	0	0	0
GO:0007500	mesodermal cell fate determination	biological_process	5	0	0	0	0	0	0	0	0	0	0	0	0
GO:0007499	ectoderm and mesoderm interaction	biological_process	3	0	0	0	0	0	0	0	0	0	0	0	0
GO:0007488	histoblast morphogenesis	biological_process	10	0	0	0	0	0	0	0	0	0	0	0	0
GO:0007487	analia development	biological_process	4	0	0	0	0	0	0	0	0	0	0	0	0
GO:0007485	imaginal disc-derived male genitalia development	biological_process	6	0	0	0	0	0	0	0	0	0	0	0	0
GO:0007483	genital disc morphogenesis	biological_process	3	0	0	0	0	0	0	0	0	0	0	0	0
GO:0007482	haltere development	biological_process	11	0	0	0	0	0	0	0	0	0	0	0	0
GO:0007479	leg disc proximal/distal pattern formation	biological_process	17	0	0	0	0	0	0	0	0	0	0	0	0
GO:0007473	wing disc proximal/distal pattern formation	biological_process	3	0	0	0	0	0	0	0	0	0	0	0	0

GO:0007469	antennal development	biological_process	11	0	0	0	0	0	0	0	0	0	0	0	0
GO:0007468	regulation of rhodopsin gene expression	biological_process	3	0	0	0	0	0	0	0	0	0	0	0	0
GO:0007465	R7 cell fate commitment	biological_process	22	0	0	0	0	0	0	0	0	0	0	0	0
GO:0007464	R3/R4 cell fate commitment	biological_process	13	0	0	0	0	0	0	0	0	0	0	0	0
GO:0007458	progression of morphogenetic furrow involved in compound eye	biological_process	8	0	0	0	0	0	0	0	0	0	0	0	0
GO:0007451	dorsal/ventral lineage restriction, imaginal disc	biological_process	3	0	0	0	0	0	0	0	0	0	0	0	0
GO:0007450	dorsal/ventral pattern formation, imaginal disc	biological_process	6	0	0	0	0	0	0	0	0	0	0	0	0
GO:0007448	anterior/posterior pattern specification, imaginal disc	biological_process	6	0	0	0	0	0	0	0	0	0	0	0	0
GO:0007447	imaginal disc pattern formation	biological_process	5	0	0	0	0	0	0	0	0	0	0	0	0
GO:0007446	imaginal disc growth	biological_process	17	0	0	0	0	0	0	0	0	0	0	0	0
GO:0007438	oenocyte development	biological_process	8	0	0	0	0	0	0	0	0	0	0	0	0
GO:0007426	tracheal outgrowth, open tracheal system	biological_process	18	0	0	0	0	0	0	0	0	0	0	0	0
GO:0007425	epithelial cell fate determination, open tracheal system	biological_process	3	0	0	0	0	0	0	0	0	0	0	0	0
GO:0007416	synapse assembly	biological_process	23	0	0	0	0	0	0	0	0	0	0	0	0
GO:0007415	defasciculation of motor neuron axon	biological_process	8	0	0	0	0	0	0	0	0	0	0	0	0
GO:0007280	pole cell migration	biological_process	22	0	0	0	0	0	0	0	0	0	0	0	0
GO:0007278	pole cell fate determination	biological_process	4	0	0	0	0	0	0	0	0	0	0	0	0
GO:0006936	muscle contraction	biological_process	11	0	0	0	0	0	0	0	0	0	0	0	0
GO:0007035	vacuolar acidification	biological_process	12	0	0	0	0	0	0	0	0	0	0	0	0
GO:0007080	mitotic metaphase plate congression	biological_process	14	0	0	0	0	0	0	0	0	0	0	0	0
GO:0007065	male meiosis sister chromatid cohesion	biological_process	5	0	0	0	0	0	0	0	0	0	0	0	0
GO:0007064	mitotic sister chromatid cohesion	biological_process	8	0	0	0	0	0	0	0	0	0	0	0	0
GO:0007060	male meiosis chromosome segregation	biological_process	20	0	0	0	0	0	0	0	0	0	0	0	0
GO:0007059	chromosome segregation	biological_process	38	0	0	0	0	0	0	0	0	0	0	0	0
GO:0007057	spindle assembly involved in female meiosis I	biological_process	5	0	0	0	0	0	0	0	0	0	0	0	0
GO:0007053	spindle assembly involved in male meiosis	biological_process	4	0	0	0	0	0	0	0	0	0	0	0	0
GO:0007052	mitotic spindle organization	biological_process	48	0	0	0	0	0	0	0	0	0	0	0	0
GO:0007051	spindle organization	biological_process	12	0	0	0	0	0	0	0	0	0	0	0	0
GO:0007050	cell cycle arrest	biological_process	4	0	0	0	0	0	0	0	0	0	0	0	0
GO:0007043	cell-cell junction assembly	biological_process	6	0	0	0	0	0	0	0	0	0	0	0	0
GO:0007041	lysosomal transport	biological_process	9	0	0	0	0	0	0	0	0	0	0	0	0
GO:0007031	peroxisome organization	biological_process	16	0	0	0	0	0	0	0	0	0	0	0	0
GO:0007277	pole cell development	biological_process	7	0	0	0	0	0	0	0	0	0	0	0	0
GO:0007029	endoplasmic reticulum organization	biological_process	19	0	0	0	0	0	0	0	0	0	0	0	0
GO:0007023	post-chaperonin tubulin folding pathway	biological_process	6	0	0	0	0	0	0	0	0	0	0	0	0
GO:0007021	tubulin complex assembly	biological_process	8	0	0	0	0	0	0	0	0	0	0	0	0
GO:0007017	microtubule-based process	biological_process	23	0	0	0	0	0	0	0	0	0	0	0	0
GO:0007016	cytoskeletal anchoring at plasma membrane	biological_process	11	0	0	0	0	0	0	0	0	0	0	0	0
GO:0007009	plasma membrane organization	biological_process	15	0	0	0	0	0	0	0	0	0	0	0	0
GO:0007006	mitochondrial membrane organization	biological_process	5	0	0	0	0	0	0	0	0	0	0	0	0
GO:0007005	mitochondrion organization	biological_process	39	0	0	0	0	0	0	0	0	0	0	0	0
GO:0007004	telomere maintenance via telomerase	biological_process	4	0	0	0	0	0	0	0	0	0	0	0	0
GO:0006997	nucleus organization	biological_process	10	0	0	0	0	0	0	0	0	0	0	0	0
GO:0006974	cellular response to DNA damage stimulus	biological_process	34	0	0	0	0	0	0	0	0	0	0	0	0
GO:0006967	positive regulation of antifungal peptide biosynthetic process	biological_process	3	0	0	0	0	0	0	0	0	0	0	0	0
GO:0007091	metaphase/anaphase transition of mitotic cell cycle	biological_process	12	0	0	0	0	0	0	0	0	0	0	0	0
GO:0007094	mitotic spindle assembly checkpoint	biological_process	15	0	0	0	0	0	0	0	0	0	0	0	0
GO:0007096	regulation of exit from mitosis	biological_process	10	0	0	0	0	0	0	0	0	0	0	0	0

GO:0007097	nuclear migration	biological_process	17	0	0	0	0	0	0	0	0	0	0	0	0
GO:0007270	neuron-neuron synaptic transmission	biological_process	15	0	0	0	0	0	0	0	0	0	0	0	0
GO:0007264	small GTPase mediated signal transduction	biological_process	17	0	0	0	0	0	0	0	0	0	0	0	0
GO:0007254	JNK cascade	biological_process	17	0	0	0	0	0	0	0	0	0	0	0	0
GO:0007249	I-kappaB kinase/NF-kappaB signaling	biological_process	5	0	0	0	0	0	0	0	0	0	0	0	0
GO:0007229	integrin-mediated signaling pathway	biological_process	7	0	0	0	0	0	0	0	0	0	0	0	0
GO:0007216	G-protein coupled glutamate receptor signaling pathway	biological_process	4	0	0	0	0	0	0	0	0	0	0	0	0
GO:0007214	gamma-aminobutyric acid signaling pathway	biological_process	3	0	0	0	0	0	0	0	0	0	0	0	0
GO:0007200	phospholipase C-activating G-protein coupled receptor signaling pathway	biological_process	4	0	0	0	0	0	0	0	0	0	0	0	0
GO:0007193	adenylate cyclase-inhibiting G-protein coupled receptor signaling pathway	biological_process	12	0	0	0	0	0	0	0	0	0	0	0	0
GO:0007188	adenylate cyclase-modulating G-protein coupled receptor signaling pathway	biological_process	19	0	0	0	0	0	0	0	0	0	0	0	0
GO:0007175	negative regulation of epidermal growth factor-activated receptor activity	biological_process	5	0	0	0	0	0	0	0	0	0	0	0	0
GO:0007169	transmembrane receptor protein tyrosine kinase signaling pathway	biological_process	30	0	0	0	0	0	0	0	0	0	0	0	0
GO:0007168	receptor guanylyl cyclase signaling pathway	biological_process	7	0	0	0	0	0	0	0	0	0	0	0	0
GO:0007164	establishment of tissue polarity	biological_process	3	0	0	0	0	0	0	0	0	0	0	0	0
GO:0007163	establishment or maintenance of cell polarity	biological_process	23	0	0	0	0	0	0	0	0	0	0	0	0
GO:0007156	homophilic cell adhesion via plasma membrane adhesion molecules	biological_process	45	0	0	0	0	0	0	0	0	0	0	0	0
GO:0007147	female meiosis II	biological_process	3	0	0	0	0	0	0	0	0	0	0	0	0
GO:0007144	female meiosis I	biological_process	4	0	0	0	0	0	0	0	0	0	0	0	0
GO:0007141	male meiosis I	biological_process	10	0	0	0	0	0	0	0	0	0	0	0	0
GO:0007130	synaptonemal complex assembly	biological_process	6	0	0	0	0	0	0	0	0	0	0	0	0
GO:0007113	endomitotic cell cycle	biological_process	11	0	0	0	0	0	0	0	0	0	0	0	0
GO:0007111	meiosis II cytokinesis	biological_process	18	0	0	0	0	0	0	0	0	0	0	0	0
GO:0007110	meiosis I cytokinesis	biological_process	22	0	0	0	0	0	0	0	0	0	0	0	0
GO:0007099	centriole replication	biological_process	25	0	0	0	0	0	0	0	0	0	0	0	0
GO:0007098	centrosome cycle	biological_process	36	0	0	0	0	0	0	0	0	0	0	0	0
GO:0006072	glycerol-3-phosphate metabolic process	biological_process	9	0	0	0	0	0	0	0	0	0	0	0	0
GO:0006066	alcohol metabolic process	biological_process	4	0	0	0	0	0	0	0	0	0	0	0	0
GO:0006048	UDP-N-acetylglucosamine biosynthetic process	biological_process	4	0	0	0	0	0	0	0	0	0	0	0	0
GO:0002092	positive regulation of receptor internalization	biological_process	5	0	0	0	0	0	0	0	0	0	0	0	0
GO:0002921	negative regulation of humoral immune response	biological_process	3	0	0	0	0	0	0	0	0	0	0	0	0
GO:0002433	immune response-regulating cell surface receptor signaling pathway involved in phagocytosis	biological_process	3	0	0	0	0	0	0	0	0	0	0	0	0
GO:0002385	mucosal immune response	biological_process	12	0	0	0	0	0	0	0	0	0	0	0	0
GO:0002347	response to tumor cell	biological_process	5	0	0	0	0	0	0	0	0	0	0	0	0
GO:0002225	positive regulation of antimicrobial peptide production	biological_process	21	0	0	0	0	0	0	0	0	0	0	0	0
GO:0002218	activation of innate immune response	biological_process	4	0	0	0	0	0	0	0	0	0	0	0	0
GO:0002183	cytoplasmic translational initiation	biological_process	3	0	0	0	0	0	0	0	0	0	0	0	0
GO:0002161	aminoacyl-tRNA editing activity	molecular_function	11	0	0	0	0	0	0	0	0	0	0	0	0
GO:0002143	tRNA wobble position uridine thiolation	biological_process	4	0	0	0	0	0	0	0	0	0	0	0	0
GO:0002121	inter-male aggressive behavior	biological_process	15	0	0	0	0	0	0	0	0	0	0	0	0
GO:0002115	store-operated calcium entry	biological_process	3	0	0	0	0	0	0	0	0	0	0	0	0
GO:0002098	tRNA wobble uridine modification	biological_process	14	0	0	0	0	0	0	0	0	0	0	0	0

GO:0002082	regulation of oxidative phosphorylation	biological_process	5	0	0	0	0	0	0	0	0	0	0	0	0
GO:0001746	Bolwig's organ morphogenesis	biological_process	14	0	0	0	0	0	0	0	0	0	0	0	0
GO:0002052	positive regulation of neuroblast proliferation	biological_process	24	0	0	0	0	0	0	0	0	0	0	0	0
GO:0002039	p53 binding	molecular_function	6	0	0	0	0	0	0	0	0	0	0	0	0
GO:0002031	G-protein coupled receptor internalization	biological_process	4	0	0	0	0	0	0	0	0	0	0	0	0
GO:0002027	regulation of heart rate	biological_process	7	0	0	0	0	0	0	0	0	0	0	0	0
GO:0002009	morphogenesis of an epithelium	biological_process	18	0	0	0	0	0	0	0	0	0	0	0	0
GO:0001964	startle response	biological_process	6	0	0	0	0	0	0	0	0	0	0	0	0
GO:0001934	positive regulation of protein phosphorylation	biological_process	11	0	0	0	0	0	0	0	0	0	0	0	0
GO:0001933	negative regulation of protein phosphorylation	biological_process	11	0	0	0	0	0	0	0	0	0	0	0	0
GO:0001881	receptor recycling	biological_process	4	0	0	0	0	0	0	0	0	0	0	0	0
GO:0001838	embryonic epithelial tube formation	biological_process	3	0	0	0	0	0	0	0	0	0	0	0	0
GO:0001784	phosphotyrosine binding	molecular_function	6	0	0	0	0	0	0	0	0	0	0	0	0
GO:0001764	neuron migration	biological_process	6	0	0	0	0	0	0	0	0	0	0	0	0
GO:0003007	heart morphogenesis	biological_process	12	0	0	0	0	0	0	0	0	0	0	0	0
GO:0003015	heart process	biological_process	11	0	0	0	0	0	0	0	0	0	0	0	0
GO:0003053	circadian regulation of heart rate	biological_process	3	0	0	0	0	0	0	0	0	0	0	0	0
GO:0003151	outflow tract morphogenesis	biological_process	4	0	0	0	0	0	0	0	0	0	0	0	0
GO:0003951	NAD+ kinase activity	molecular_function	12	0	0	0	0	0	0	0	0	0	0	0	0
GO:0003917	DNA topoisomerase type I activity	molecular_function	3	0	0	0	0	0	0	0	0	0	0	0	0
GO:0003916	DNA topoisomerase activity	molecular_function	3	0	0	0	0	0	0	0	0	0	0	0	0
GO:0003906	DNA-(apurinic or apyrimidinic site) lyase activity	molecular_function	5	0	0	0	0	0	0	0	0	0	0	0	0
GO:0003904	deoxyribodipyrimidine photo-lyase activity	molecular_function	3	0	0	0	0	0	0	0	0	0	0	0	0
GO:0003899	DNA-directed 5'-3' RNA polymerase activity	molecular_function	26	0	0	0	0	0	0	0	0	0	0	0	0
GO:0003887	DNA-directed DNA polymerase activity	molecular_function	12	0	0	0	0	0	0	0	0	0	0	0	0
GO:0003841	1-acylglycerol-3-phosphate O-acyltransferase activity	molecular_function	4	0	0	0	0	0	0	0	0	0	0	0	0
GO:0003785	actin monomer binding	molecular_function	4	0	0	0	0	0	0	0	0	0	0	0	0
GO:0003774	motor activity	molecular_function	16	0	0	0	0	0	0	0	0	0	0	0	0
GO:0003746	translation elongation factor activity	molecular_function	15	0	0	0	0	0	0	0	0	0	0	0	0
GO:0003730	mRNA 3'-UTR binding	molecular_function	39	0	0	0	0	0	0	0	0	0	0	0	0
GO:0003727	single-stranded RNA binding	molecular_function	14	0	0	0	0	0	0	0	0	0	0	0	0
GO:0003726	double-stranded RNA adenosine deaminase activity	molecular_function	5	0	0	0	0	0	0	0	0	0	0	0	0
GO:0003724	RNA helicase activity	molecular_function	44	0	0	0	0	0	0	0	0	0	0	0	0
GO:0003707	steroid hormone receptor activity	molecular_function	16	0	0	0	0	0	0	0	0	0	0	0	0
GO:0003696	satellite DNA binding	molecular_function	4	0	0	0	0	0	0	0	0	0	0	0	0
GO:0003690	double-stranded DNA binding	molecular_function	25	0	0	0	0	0	0	0	0	0	0	0	0
GO:0003688	DNA replication origin binding	molecular_function	16	0	0	0	0	0	0	0	0	0	0	0	0
GO:0003678	DNA helicase activity	molecular_function	22	0	0	0	0	0	0	0	0	0	0	0	0
GO:0003400	regulation of COPII vesicle coating	biological_process	4	0	0	0	0	0	0	0	0	0	0	0	0
GO:0003382	epithelial cell morphogenesis	biological_process	8	0	0	0	0	0	0	0	0	0	0	0	0
GO:0003380	establishment or maintenance of cytoskeleton polarity involved in gastrulation	biological_process	4	0	0	0	0	0	0	0	0	0	0	0	0
GO:0003341	cilium movement	biological_process	26	0	0	0	0	0	0	0	0	0	0	0	0
GO:0003333	amino acid transmembrane transport	biological_process	35	0	0	0	0	0	0	0	0	0	0	0	0

GO:0001755	neural crest cell migration	biological_process	4	0	0	0	0	0	0	0	0	0	0	0	0
GO:0001744	optic lobe placode formation	biological_process	4	0	0	0	0	0	0	0	0	0	0	0	0
GO:0003958	NADPH-hemoprotein reductase activity	molecular_function	3	0	0	0	0	0	0	0	0	0	0	0	0
GO:0000335	negative regulation of transposition, DNA-mediated	biological_process	6	0	0	0	0	0	0	0	0	0	0	0	0
GO:0000819	sister chromatid segregation	biological_process	4	0	0	0	0	0	0	0	0	0	0	0	0
GO:0000738	DNA catabolic process, exonucleolytic	biological_process	4	0	0	0	0	0	0	0	0	0	0	0	0
GO:0000737	DNA catabolic process, endonucleolytic	biological_process	17	0	0	0	0	0	0	0	0	0	0	0	0
GO:0000727	double-strand break repair via break-induced replication	biological_process	14	0	0	0	0	0	0	0	0	0	0	0	0
GO:0000712	resolution of meiotic recombination intermediates	biological_process	9	0	0	0	0	0	0	0	0	0	0	0	0
GO:0000463	maturation of LSU-rRNA from tricistronic rRNA transcript (SSU-rRNA, 5.8S rRNA, LSU-rRNA)	biological_process	12	0	0	0	0	0	0	0	0	0	0	0	0
GO:0000462	maturation of SSU-rRNA from tricistronic rRNA transcript (SSU-rRNA, 5.8S rRNA, LSU-rRNA)	biological_process	23	0	0	0	0	0	0	0	0	0	0	0	0
GO:0000447	endonucleolytic cleavage in ITS1 to separate SSU-rRNA from 5.8S rRNA and LSU-rRNA from tricistronic rRNA transcript (SSU-rRNA, 5.8S rRNA, LSU-rRNA)	biological_process	9	0	0	0	0	0	0	0	0	0	0	0	0
GO:0000423	macromitophagy	biological_process	4	0	0	0	0	0	0	0	0	0	0	0	0
GO:0000422	mitophagy	biological_process	21	0	0	0	0	0	0	0	0	0	0	0	0
GO:0000379	tRNA-type intron splice site recognition and cleavage	biological_process	3	0	0	0	0	0	0	0	0	0	0	0	0
GO:0000338	protein deneddylation	biological_process	10	0	0	0	0	0	0	0	0	0	0	0	0
GO:0000302	response to reactive oxygen species	biological_process	11	0	0	0	0	0	0	0	0	0	0	0	0
GO:0001709	cell fate determination	biological_process	13	0	0	0	0	0	0	0	0	0	0	0	0
GO:0000288	nuclear-transcribed mRNA catabolic process, deadenylation-dependent decay	biological_process	8	0	0	0	0	0	0	0	0	0	0	0	0
GO:0000266	mitochondrial fission	biological_process	9	0	0	0	0	0	0	0	0	0	0	0	0
GO:0000253	3-keto sterol reductase activity	molecular_function	9	0	0	0	0	0	0	0	0	0	0	0	0
GO:0000226	microtubule cytoskeleton organization	biological_process	59	0	0	0	0	0	0	0	0	0	0	0	0
GO:0000213	tRNA-intron endonuclease activity	molecular_function	3	0	0	0	0	0	0	0	0	0	0	0	0
GO:0000209	protein polyubiquitination	biological_process	75	0	0	0	0	0	0	0	0	0	0	0	0
GO:0000175	3'-5'-exoribonuclease activity	molecular_function	14	0	0	0	0	0	0	0	0	0	0	0	0
GO:0000056	ribosomal small subunit export from nucleus	biological_process	3	0	0	0	0	0	0	0	0	0	0	0	0
GO:0000055	ribosomal large subunit export from nucleus	biological_process	6	0	0	0	0	0	0	0	0	0	0	0	0
GO:0000049	tRNA binding	molecular_function	26	0	0	0	0	0	0	0	0	0	0	0	0
GO:0000027	ribosomal large subunit assembly	biological_process	19	0	0	0	0	0	0	0	0	0	0	0	0
GO:0000014	single-stranded DNA endodeoxyribonuclease activity	molecular_function	17	0	0	0	0	0	0	0	0	0	0	0	0
GO:0000822	inositol hexakisphosphate binding	molecular_function	5	0	0	0	0	0	0	0	0	0	0	0	0
GO:0000915	actomyosin contractile ring assembly	biological_process	15	0	0	0	0	0	0	0	0	0	0	0	0
GO:0000916	actomyosin contractile ring contraction	biological_process	13	0	0	0	0	0	0	0	0	0	0	0	0
GO:0000972	transcription-dependent tethering of RNA polymerase II gene DNA at nuclear periphery	biological_process	4	0	0	0	0	0	0	0	0	0	0	0	0
GO:0001707	mesoderm formation	biological_process	3	0	0	0	0	0	0	0	0	0	0	0	0
GO:0001676	long-chain fatty acid metabolic process	biological_process	8	0	0	0	0	0	0	0	0	0	0	0	0
GO:0001671	ATPase activator activity	molecular_function	14	0	0	0	0	0	0	0	0	0	0	0	0
GO:0001664	G-protein coupled receptor binding	molecular_function	31	0	0	0	0	0	0	0	0	0	0	0	0
GO:0001654	eye development	biological_process	19	0	0	0	0	0	0	0	0	0	0	0	0
GO:0001653	peptide receptor activity	molecular_function	10	0	0	0	0	0	0	0	0	0	0	0	0
GO:0001578	microtubule bundle formation	biological_process	11	0	0	0	0	0	0	0	0	0	0	0	0
GO:0001558	regulation of cell growth	biological_process	13	0	0	0	0	0	0	0	0	0	0	0	0

GO:0001556	oocyte maturation	biological_process	4	0	0	0	0	0	0	0	0	0	0	0	0
GO:0001555	oocyte growth	biological_process	6	0	0	0	0	0	0	0	0	0	0	0	0
GO:0001530	lipopolysaccharide binding	molecular_function	7	0	0	0	0	0	0	0	0	0	0	0	0
GO:0001525	angiogenesis	biological_process	3	0	0	0	0	0	0	0	0	0	0	0	0
GO:0001522	pseudouridine synthesis	biological_process	4	0	0	0	0	0	0	0	0	0	0	0	0
GO:0001113	transcriptional open complex formation at RNA polymerase II promoter	biological_process	8	0	0	0	0	0	0	0	0	0	0	0	0
GO:0001111	promoter clearance from RNA polymerase II promoter	biological_process	8	0	0	0	0	0	0	0	0	0	0	0	0
GO:0001102	RNA polymerase II activating transcription factor binding	molecular_function	17	0	0	0	0	0	0	0	0	0	0	0	0
GO:0001092	TFIIA-class transcription factor binding	molecular_function	4	0	0	0	0	0	0	0	0	0	0	0	0
GO:0001055	RNA polymerase II activity	molecular_function	12	0	0	0	0	0	0	0	0	0	0	0	0
GO:0001047	core promoter binding	molecular_function	7	0	0	0	0	0	0	0	0	0	0	0	0
GO:0001046	core promoter sequence-specific DNA binding	molecular_function	5	0	0	0	0	0	0	0	0	0	0	0	0
GO:0001016	RNA polymerase III regulatory region DNA binding	molecular_function	4	0	0	0	0	0	0	0	0	0	0	0	0
GO:0001012	RNA polymerase II regulatory region DNA binding	molecular_function	10	0	0	0	0	0	0	0	0	0	0	0	0
GO:0001006	RNA polymerase III type 3 promoter sequence-specific DNA binding	molecular_function	6	0	0	0	0	0	0	0	0	0	0	0	0
GO:0000993	RNA polymerase II core binding	molecular_function	15	0	0	0	0	0	0	0	0	0	0	0	0
GO:0000973	posttranscriptional tethering of RNA polymerase II gene DNA at nuclear periphery	biological_process	4	0	0	0	0	0	0	0	0	0	0	0	0
GO:0003954	NADH dehydrogenase activity	molecular_function	32	0	0	0	0	0	0	0	0	0	0	0	0
GO:0003985	acetyl-CoA C-acetyltransferase activity	molecular_function	3	0	0	0	0	0	0	0	0	0	0	0	0
GO:0006044	N-acetylglucosamine metabolic process	biological_process	9	0	0	0	0	0	0	0	0	0	0	0	0
GO:0005121	Toll binding	molecular_function	8	0	0	0	0	0	0	0	0	0	0	0	0
GO:0005249	voltage-gated potassium channel activity	molecular_function	12	0	0	0	0	0	0	0	0	0	0	0	0
GO:0005245	voltage-gated calcium channel activity	molecular_function	7	0	0	0	0	0	0	0	0	0	0	0	0
GO:0005242	inward rectifier potassium channel activity	molecular_function	3	0	0	0	0	0	0	0	0	0	0	0	0
GO:0005227	calcium activated cation channel activity	molecular_function	3	0	0	0	0	0	0	0	0	0	0	0	0
GO:0005216	ion channel activity	molecular_function	7	0	0	0	0	0	0	0	0	0	0	0	0
GO:0005212	structural constituent of eye lens	molecular_function	4	0	0	0	0	0	0	0	0	0	0	0	0
GO:0005184	neuropeptide hormone activity	molecular_function	33	0	0	0	0	0	0	0	0	0	0	0	0
GO:0005179	hormone activity	molecular_function	26	0	0	0	0	0	0	0	0	0	0	0	0
GO:0005178	integrin binding	molecular_function	11	0	0	0	0	0	0	0	0	0	0	0	0
GO:0005158	insulin receptor binding	molecular_function	14	0	0	0	0	0	0	0	0	0	0	0	0
GO:0005154	epidermal growth factor receptor binding	molecular_function	8	0	0	0	0	0	0	0	0	0	0	0	0
GO:0005122	torso binding	molecular_function	3	0	0	0	0	0	0	0	0	0	0	0	0
GO:0005118	sevenless binding	molecular_function	4	0	0	0	0	0	0	0	0	0	0	0	0
GO:0004864	protein phosphatase inhibitor activity	molecular_function	10	0	0	0	0	0	0	0	0	0	0	0	0
GO:0005113	patched binding	molecular_function	5	0	0	0	0	0	0	0	0	0	0	0	0
GO:0005092	GDP-dissociation inhibitor activity	molecular_function	6	0	0	0	0	0	0	0	0	0	0	0	0
GO:0005089	Rho guanyl-nucleotide exchange factor activity	molecular_function	24	0	0	0	0	0	0	0	0	0	0	0	0
GO:0005086	ARF guanyl-nucleotide exchange factor activity	molecular_function	7	0	0	0	0	0	0	0	0	0	0	0	0
GO:0005085	guanyl-nucleotide exchange factor activity	molecular_function	24	0	0	0	0	0	0	0	0	0	0	0	0
GO:0005080	protein kinase C binding	molecular_function	6	0	0	0	0	0	0	0	0	0	0	0	0
GO:0005078	MAP-kinase scaffold activity	molecular_function	6	0	0	0	0	0	0	0	0	0	0	0	0
GO:0005049	nuclear export signal receptor activity	molecular_function	4	0	0	0	0	0	0	0	0	0	0	0	0
GO:0005044	scavenger receptor activity	molecular_function	14	0	0	0	0	0	0	0	0	0	0	0	0

GO:0005001	transmembrane receptor protein tyrosine phosphatase activity	molecular_function	5	0	0	0	0	0	0	0	0	0	0	0	0
GO:0004965	G-protein coupled GABA receptor activity	molecular_function	3	0	0	0	0	0	0	0	0	0	0	0	0
GO:0004879	RNA polymerase II transcription factor activity, ligand-activated sequence-specific DNA binding	molecular_function	18	0	0	0	0	0	0	0	0	0	0	0	0
GO:0005251	delayed rectifier potassium channel activity	molecular_function	5	0	0	0	0	0	0	0	0	0	0	0	0
GO:0005262	calcium channel activity	molecular_function	24	0	0	0	0	0	0	0	0	0	0	0	0
GO:0005283	sodium:amino acid symporter activity	molecular_function	7	0	0	0	0	0	0	0	0	0	0	0	0
GO:0005313	L-glutamate transmembrane transporter activity	molecular_function	5	0	0	0	0	0	0	0	0	0	0	0	0
GO:0006035	cuticle chitin biosynthetic process	biological_process	4	0	0	0	0	0	0	0	0	0	0	0	0
GO:0006032	chitin catabolic process	biological_process	17	0	0	0	0	0	0	0	0	0	0	0	0
GO:0006012	galactose metabolic process	biological_process	3	0	0	0	0	0	0	0	0	0	0	0	0
GO:0006011	UDP-glucose metabolic process	biological_process	10	0	0	0	0	0	0	0	0	0	0	0	0
GO:0006002	fructose 6-phosphate metabolic process	biological_process	3	0	0	0	0	0	0	0	0	0	0	0	0
GO:0006000	fructose metabolic process	biological_process	7	0	0	0	0	0	0	0	0	0	0	0	0
GO:0005992	trehalose biosynthetic process	biological_process	3	0	0	0	0	0	0	0	0	0	0	0	0
GO:0005991	trehalose metabolic process	biological_process	3	0	0	0	0	0	0	0	0	0	0	0	0
GO:0005977	glycogen metabolic process	biological_process	6	0	0	0	0	0	0	0	0	0	0	0	0
GO:0005550	pheromone binding	molecular_function	5	0	0	0	0	0	0	0	0	0	0	0	0
GO:0005537	mannose binding	molecular_function	3	0	0	0	0	0	0	0	0	0	0	0	0
GO:0005534	galactose binding	molecular_function	19	0	0	0	0	0	0	0	0	0	0	0	0
GO:0005518	collagen binding	molecular_function	6	0	0	0	0	0	0	0	0	0	0	0	0
GO:0005504	fatty acid binding	molecular_function	7	0	0	0	0	0	0	0	0	0	0	0	0
GO:0005484	SNAP receptor activity	molecular_function	25	0	0	0	0	0	0	0	0	0	0	0	0
GO:0005459	UDP-galactose transmembrane transporter activity	molecular_function	5	0	0	0	0	0	0	0	0	0	0	0	0
GO:0005452	inorganic anion exchanger activity	molecular_function	3	0	0	0	0	0	0	0	0	0	0	0	0
GO:0005416	cation:amino acid symporter activity	molecular_function	6	0	0	0	0	0	0	0	0	0	0	0	0
GO:0005391	sodium:potassium-exchanging ATPase activity	molecular_function	8	0	0	0	0	0	0	0	0	0	0	0	0
GO:0005385	zinc ion transmembrane transporter activity	molecular_function	16	0	0	0	0	0	0	0	0	0	0	0	0
GO:0005351	sugar:proton symporter activity	molecular_function	20	0	0	0	0	0	0	0	0	0	0	0	0
GO:0005337	nucleoside transmembrane transporter activity	molecular_function	5	0	0	0	0	0	0	0	0	0	0	0	0
GO:0005328	neurotransmitter:sodium symporter activity	molecular_function	21	0	0	0	0	0	0	0	0	0	0	0	0
GO:0005326	neurotransmitter transporter activity	molecular_function	20	0	0	0	0	0	0	0	0	0	0	0	0
GO:0005314	high-affinity glutamate transmembrane transporter activity	molecular_function	3	0	0	0	0	0	0	0	0	0	0	0	0
GO:0004867	serine-type endopeptidase inhibitor activity	molecular_function	79	0	0	0	0	0	0	0	0	0	0	0	0
GO:0004860	protein kinase inhibitor activity	molecular_function	6	0	0	0	0	0	0	0	0	0	0	0	0
GO:0003988	acetyl-CoA C-acyltransferase activity	molecular_function	4	0	0	0	0	0	0	0	0	0	0	0	0
GO:0004165	dodecenoyl-CoA delta-isomerase activity	molecular_function	4	0	0	0	0	0	0	0	0	0	0	0	0
GO:0004438	phosphatidylinositol-3-phosphatase activity	molecular_function	7	0	0	0	0	0	0	0	0	0	0	0	0
GO:0004386	helicase activity	molecular_function	32	0	0	0	0	0	0	0	0	0	0	0	0
GO:0004383	guanylate cyclase activity	molecular_function	13	0	0	0	0	0	0	0	0	0	0	0	0
GO:0004368	glycerol-3-phosphate dehydrogenase activity	molecular_function	3	0	0	0	0	0	0	0	0	0	0	0	0
GO:0004367	glycerol-3-phosphate dehydrogenase [NAD+] activity	molecular_function	5	0	0	0	0	0	0	0	0	0	0	0	0
GO:0004348	glucosylceramidase activity	molecular_function	3	0	0	0	0	0	0	0	0	0	0	0	0
GO:0004333	fumarate hydratase activity	molecular_function	4	0	0	0	0	0	0	0	0	0	0	0	0
GO:0004312	fatty acid synthase activity	molecular_function	4	0	0	0	0	0	0	0	0	0	0	0	0
GO:0004307	ethanolaminephosphotransferase activity	molecular_function	3	0	0	0	0	0	0	0	0	0	0	0	0
GO:0004303	estradiol 17-beta-dehydrogenase activity	molecular_function	17	0	0	0	0	0	0	0	0	0	0	0	0
GO:0004301	epoxide hydrolase activity	molecular_function	3	0	0	0	0	0	0	0	0	0	0	0	0
GO:0004167	dopachrome isomerase activity	molecular_function	5	0	0	0	0	0	0	0	0	0	0	0	0
GO:0004129	cytochrome-c oxidase activity	molecular_function	22	0	0	0	0	0	0	0	0	0	0	0	0
GO:0004850	uridine phosphorylase activity	molecular_function	3	0	0	0	0	0	0	0	0	0	0	0	0
GO:0004126	cytidine deaminase activity	molecular_function	3	0	0	0	0	0	0	0	0	0	0	0	0

GO:0004115	3',5'-cyclic-AMP phosphodiesterase activity	molecular_function	4	0	0	0	0	0	0	0	0	0	0	0	0
GO:0004114	3',5'-cyclic-nucleotide phosphodiesterase activity	molecular_function	3	0	0	0	0	0	0	0	0	0	0	0	0
GO:0004096	catalase activity	molecular_function	3	0	0	0	0	0	0	0	0	0	0	0	0
GO:0004092	carnitine O-acetyltransferase activity	molecular_function	3	0	0	0	0	0	0	0	0	0	0	0	0
GO:0004035	alkaline phosphatase activity	molecular_function	14	0	0	0	0	0	0	0	0	0	0	0	0
GO:0004034	aldose 1-epimerase activity	molecular_function	5	0	0	0	0	0	0	0	0	0	0	0	0
GO:0004029	aldehyde dehydrogenase (NAD) activity	molecular_function	3	0	0	0	0	0	0	0	0	0	0	0	0
GO:0004022	alcohol dehydrogenase (NAD) activity	molecular_function	7	0	0	0	0	0	0	0	0	0	0	0	0
GO:0004016	adenylate cyclase activity	molecular_function	13	0	0	0	0	0	0	0	0	0	0	0	0
GO:0003997	acyl-CoA oxidase activity	molecular_function	6	0	0	0	0	0	0	0	0	0	0	0	0
GO:0003994	aconitate hydratase activity	molecular_function	4	0	0	0	0	0	0	0	0	0	0	0	0
GO:0004467	long-chain fatty acid-CoA ligase activity	molecular_function	9	0	0	0	0	0	0	0	0	0	0	0	0
GO:0004497	monooxygenase activity	molecular_function	14	0	0	0	0	0	0	0	0	0	0	0	0
GO:0004518	nuclease activity	molecular_function	19	0	0	0	0	0	0	0	0	0	0	0	0
GO:0004519	endonuclease activity	molecular_function	19	0	0	0	0	0	0	0	0	0	0	0	0
GO:0004828	serine-tRNA ligase activity	molecular_function	3	0	0	0	0	0	0	0	0	0	0	0	0
GO:0004813	alanine-tRNA ligase activity	molecular_function	3	0	0	0	0	0	0	0	0	0	0	0	0
GO:0004806	triglyceride lipase activity	molecular_function	3	0	0	0	0	0	0	0	0	0	0	0	0
GO:0004805	trehalose-phosphatase activity	molecular_function	3	0	0	0	0	0	0	0	0	0	0	0	0
GO:0004791	thioredoxin-disulfide reductase activity	molecular_function	3	0	0	0	0	0	0	0	0	0	0	0	0
GO:0004758	serine C-palmitoyltransferase activity	molecular_function	3	0	0	0	0	0	0	0	0	0	0	0	0
GO:0004745	retinol dehydrogenase activity	molecular_function	5	0	0	0	0	0	0	0	0	0	0	0	0
GO:0004743	pyruvate kinase activity	molecular_function	6	0	0	0	0	0	0	0	0	0	0	0	0
GO:0004715	non-membrane spanning protein tyrosine kinase activity	molecular_function	7	0	0	0	0	0	0	0	0	0	0	0	0
GO:0004714	transmembrane receptor protein tyrosine kinase activity	molecular_function	19	0	0	0	0	0	0	0	0	0	0	0	0
GO:0004713	protein tyrosine kinase activity	molecular_function	21	0	0	0	0	0	0	0	0	0	0	0	0
GO:0004712	protein serine/threonine/tyrosine kinase activity	molecular_function	6	0	0	0	0	0	0	0	0	0	0	0	0
GO:0004707	MAP kinase activity	molecular_function	7	0	0	0	0	0	0	0	0	0	0	0	0
GO:0004692	cGMP-dependent protein kinase activity	molecular_function	3	0	0	0	0	0	0	0	0	0	0	0	0
GO:0004691	cAMP-dependent protein kinase activity	molecular_function	4	0	0	0	0	0	0	0	0	0	0	0	0
GO:0004689	phosphorylase kinase activity	molecular_function	3	0	0	0	0	0	0	0	0	0	0	0	0
GO:0004663	Rab geranylgeranyltransferase activity	molecular_function	3	0	0	0	0	0	0	0	0	0	0	0	0
GO:0004656	procollagen-proline 4-dioxygenase activity	molecular_function	26	0	0	0	0	0	0	0	0	0	0	0	0
GO:0004620	phospholipase activity	molecular_function	5	0	0	0	0	0	0	0	0	0	0	0	0
GO:0004601	peroxidase activity	molecular_function	17	0	0	0	0	0	0	0	0	0	0	0	0
GO:0004582	dolichyl-phosphate beta-D-mannosyltransferase activity	molecular_function	3	0	0	0	0	0	0	0	0	0	0	0	0
GO:0004568	chitinase activity	molecular_function	17	0	0	0	0	0	0	0	0	0	0	0	0
GO:0004553	hydrolase activity, hydrolyzing O-glycosyl compounds	molecular_function	6	0	0	0	0	0	0	0	0	0	0	0	0
GO:0004536	deoxyribonuclease activity	molecular_function	4	0	0	0	0	0	0	0	0	0	0	0	0
GO:0004523	RNA-DNA hybrid ribonuclease activity	molecular_function	4	0	0	0	0	0	0	0	0	0	0	0	0
GO:0007516	hemocyte development	biological_process	7	0	0	0	0	0	0	0	0	0	0	0	0
GO:0007521	muscle cell fate determination	biological_process	5	0	0	0	0	0	0	0	0	0	0	0	0
GO:0007523	larval visceral muscle development	biological_process	9	0	0	0	0	0	0	0	0	0	0	0	0
GO:0030148	sphingolipid biosynthetic process	biological_process	26	0	0	0	0	0	0	0	0	0	0	0	0
GO:0030295	protein kinase activator activity	molecular_function	7	0	0	0	0	0	0	0	0	0	0	0	0
GO:0030267	glyoxylate reductase (NADP) activity	molecular_function	4	0	0	0	0	0	0	0	0	0	0	0	0
GO:0030258	lipid modification	biological_process	4	0	0	0	0	0	0	0	0	0	0	0	0
GO:0030247	polysaccharide binding	molecular_function	5	0	0	0	0	0	0	0	0	0	0	0	0
GO:0030241	skeletal muscle myosin thick filament assembly	biological_process	3	0	0	0	0	0	0	0	0	0	0	0	0
GO:0030237	female sex determination	biological_process	7	0	0	0	0	0	0	0	0	0	0	0	0
GO:0030234	enzyme regulator activity	molecular_function	30	0	0	0	0	0	0	0	0	0	0	0	0
GO:0030215	semaphorin receptor binding	molecular_function	8	0	0	0	0	0	0	0	0	0	0	0	0

GO:0030178	negative regulation of Wnt signaling pathway	biological_process	3	0	0	0	0	0	0	0	0	0	0	0	0
GO:0030177	positive regulation of Wnt signaling pathway	biological_process	7	0	0	0	0	0	0	0	0	0	0	0	0
GO:0030166	proteoglycan biosynthetic process	biological_process	5	0	0	0	0	0	0	0	0	0	0	0	0
GO:0030165	PDZ domain binding	molecular_function	7	0	0	0	0	0	0	0	0	0	0	0	0
GO:0030145	manganese ion binding	molecular_function	23	0	0	0	0	0	0	0	0	0	0	0	0
GO:0021782	glial cell development	biological_process	20	0	0	0	0	0	0	0	0	0	0	0	0
GO:0030100	regulation of endocytosis	biological_process	14	0	0	0	0	0	0	0	0	0	0	0	0
GO:0030097	hemopoiesis	biological_process	13	0	0	0	0	0	0	0	0	0	0	0	0
GO:0030071	regulation of mitotic metaphase/anaphase transition	biological_process	6	0	0	0	0	0	0	0	0	0	0	0	0
GO:0030048	actin filament-based movement	biological_process	11	0	0	0	0	0	0	0	0	0	0	0	0
GO:0030041	actin filament polymerization	biological_process	8	0	0	0	0	0	0	0	0	0	0	0	0
GO:0030037	actin filament reorganization involved in cell cycle	biological_process	5	0	0	0	0	0	0	0	0	0	0	0	0
GO:0030029	actin filament-based process	biological_process	5	0	0	0	0	0	0	0	0	0	0	0	0
GO:0030011	maintenance of cell polarity	biological_process	6	0	0	0	0	0	0	0	0	0	0	0	0
GO:0030007	cellular potassium ion homeostasis	biological_process	8	0	0	0	0	0	0	0	0	0	0	0	0
GO:0022883	zinc efflux transmembrane transporter activity	molecular_function	3	0	0	0	0	0	0	0	0	0	0	0	0
GO:0022843	voltage-gated cation channel activity	molecular_function	8	0	0	0	0	0	0	0	0	0	0	0	0
GO:0022804	active transmembrane transporter activity	molecular_function	8	0	0	0	0	0	0	0	0	0	0	0	0
GO:0030297	transmembrane receptor protein tyrosine kinase activator activity	molecular_function	5	0	0	0	0	0	0	0	0	0	0	0	0
GO:0030317	flagellated sperm motility	biological_process	15	0	0	0	0	0	0	0	0	0	0	0	0
GO:0030332	cyclin binding	molecular_function	8	0	0	0	0	0	0	0	0	0	0	0	0
GO:0030336	negative regulation of cell migration	biological_process	6	0	0	0	0	0	0	0	0	0	0	0	0
GO:0030674	protein binding, bridging	molecular_function	14	0	0	0	0	0	0	0	0	0	0	0	0
GO:0030641	regulation of cellular pH	biological_process	6	0	0	0	0	0	0	0	0	0	0	0	0
GO:0030619	U1 snRNA binding	molecular_function	5	0	0	0	0	0	0	0	0	0	0	0	0
GO:0030589	pseudocleavage involved in syncytial blastoderm formation	biological_process	10	0	0	0	0	0	0	0	0	0	0	0	0
GO:0030576	Cajal body organization	biological_process	5	0	0	0	0	0	0	0	0	0	0	0	0
GO:0030565	snRNA pseudouridylation guide activity	molecular_function	5	0	0	0	0	0	0	0	0	0	0	0	0
GO:0030544	Hsp70 protein binding	molecular_function	10	0	0	0	0	0	0	0	0	0	0	0	0
GO:0030539	male genitalia development	biological_process	4	0	0	0	0	0	0	0	0	0	0	0	0
GO:0030537	larval behavior	biological_process	3	0	0	0	0	0	0	0	0	0	0	0	0
GO:0030515	snoRNA binding	molecular_function	12	0	0	0	0	0	0	0	0	0	0	0	0
GO:0030513	positive regulation of BMP signaling pathway	biological_process	20	0	0	0	0	0	0	0	0	0	0	0	0
GO:0030510	regulation of BMP signaling pathway	biological_process	7	0	0	0	0	0	0	0	0	0	0	0	0
GO:0030509	BMP signaling pathway	biological_process	14	0	0	0	0	0	0	0	0	0	0	0	0
GO:0030497	fatty acid elongation	biological_process	22	0	0	0	0	0	0	0	0	0	0	0	0
GO:0030490	maturation of SSU-rRNA	biological_process	15	0	0	0	0	0	0	0	0	0	0	0	0
GO:0030488	tRNA methylation	biological_process	19	0	0	0	0	0	0	0	0	0	0	0	0
GO:0030433	ER-associated ubiquitin-dependent protein catabolic process	biological_process	25	0	0	0	0	0	0	0	0	0	0	0	0
GO:0030423	targeting of mRNA for destruction involved in RNA interference	biological_process	5	0	0	0	0	0	0	0	0	0	0	0	0
GO:0030414	peptidase inhibitor activity	molecular_function	4	0	0	0	0	0	0	0	0	0	0	0	0
GO:0030388	fructose 1,6-bisphosphate metabolic process	biological_process	4	0	0	0	0	0	0	0	0	0	0	0	0
GO:0030382	sperm mitochondrion organization	biological_process	9	0	0	0	0	0	0	0	0	0	0	0	0
GO:0030381	chorion-containing eggshell pattern formation	biological_process	12	0	0	0	0	0	0	0	0	0	0	0	0
GO:0030374	ligand-dependent nuclear receptor transcription coactivator activity	molecular_function	9	0	0	0	0	0	0	0	0	0	0	0	0

GO:0030371	translation repressor activity	molecular_function	8	0	0	0	0	0	0	0	0	0	0	0	0
GO:0030337	DNA polymerase processivity factor activity	molecular_function	4	0	0	0	0	0	0	0	0	0	0	0	0
GO:0022409	positive regulation of cell-cell adhesion	biological_process	7	0	0	0	0	0	0	0	0	0	0	0	0
GO:0021556	central nervous system formation	biological_process	9	0	0	0	0	0	0	0	0	0	0	0	0
GO:0030705	cytoskeleton-dependent intracellular transport	biological_process	5	0	0	0	0	0	0	0	0	0	0	0	0
GO:0016817	hydrolase activity, acting on acid anhydrides	molecular_function	3	0	0	0	0	0	0	0	0	0	0	0	0
GO:0017157	regulation of exocytosis	biological_process	14	0	0	0	0	0	0	0	0	0	0	0	0
GO:0017150	tRNA dihydrouridine synthase activity	molecular_function	4	0	0	0	0	0	0	0	0	0	0	0	0
GO:0017148	negative regulation of translation	biological_process	47	0	0	0	0	0	0	0	0	0	0	0	0
GO:0017112	Rab guanyl-nucleotide exchange factor activity	molecular_function	23	0	0	0	0	0	0	0	0	0	0	0	0
GO:0017108	5'-flap endonuclease activity	molecular_function	7	0	0	0	0	0	0	0	0	0	0	0	0
GO:0017085	response to insecticide	biological_process	17	0	0	0	0	0	0	0	0	0	0	0	0
GO:0017077	oxidative phosphorylation uncoupler activity	molecular_function	3	0	0	0	0	0	0	0	0	0	0	0	0
GO:0017069	snRNA binding	molecular_function	4	0	0	0	0	0	0	0	0	0	0	0	0
GO:0017016	Ras GTPase binding	molecular_function	6	0	0	0	0	0	0	0	0	0	0	0	0
GO:0016936	galactoside binding	molecular_function	3	0	0	0	0	0	0	0	0	0	0	0	0
GO:0016925	protein sumoylation	biological_process	11	0	0	0	0	0	0	0	0	0	0	0	0
GO:0016922	ligand-dependent nuclear receptor binding	molecular_function	4	0	0	0	0	0	0	0	0	0	0	0	0
GO:0016810	hydrolase activity, acting on carbon-nitrogen (but not peptide) bonds	molecular_function	4	0	0	0	0	0	0	0	0	0	0	0	0
GO:0019934	cGMP-mediated signaling	biological_process	14	0	0	0	0	0	0	0	0	0	0	0	0
GO:0016805	dipeptidase activity	molecular_function	6	0	0	0	0	0	0	0	0	0	0	0	0
GO:0016791	phosphatase activity	molecular_function	36	0	0	0	0	0	0	0	0	0	0	0	0
GO:0016790	thiolester hydrolase activity	molecular_function	4	0	0	0	0	0	0	0	0	0	0	0	0
GO:0016780	phosphotransferase activity, for other substituted phosphate groups	molecular_function	3	0	0	0	0	0	0	0	0	0	0	0	0
GO:0016779	nucleotidyltransferase activity	molecular_function	6	0	0	0	0	0	0	0	0	0	0	0	0
GO:0016773	phosphotransferase activity, alcohol group as acceptor	molecular_function	9	0	0	0	0	0	0	0	0	0	0	0	0
GO:0016758	transferase activity, transferring hexosyl groups	molecular_function	44	0	0	0	0	0	0	0	0	0	0	0	0
GO:0016757	transferase activity, transferring glycosyl groups	molecular_function	6	0	0	0	0	0	0	0	0	0	0	0	0
GO:0016709	oxidoreductase activity, acting on paired donors, with incorporation or reduction of molecular oxygen, NAD(P)H as one donor, and incorporation of one atom of oxygen	molecular_function	5	0	0	0	0	0	0	0	0	0	0	0	0
GO:0016706	oxidoreductase activity, acting on paired donors, with incorporation or reduction of molecular oxygen, 2-oxoglutarate as one donor, and incorporation of one atom each of oxygen into both donors	molecular_function	7	0	0	0	0	0	0	0	0	0	0	0	0
GO:0016702	oxidoreductase activity, acting on single donors with incorporation of molecular oxygen, incorporation of two atoms of oxygen	molecular_function	28	0	0	0	0	0	0	0	0	0	0	0	0
GO:0016620	oxidoreductase activity, acting on the aldehyde or oxo group of donors, NAD or NADP as acceptor	molecular_function	7	0	0	0	0	0	0	0	0	0	0	0	0
GO:0018026	peptidyl-lysine monomethylation	biological_process	3	0	0	0	0	0	0	0	0	0	0	0	0
GO:0018095	protein polyglutamylation	biological_process	6	0	0	0	0	0	0	0	0	0	0	0	0
GO:0018108	peptidyl-tyrosine phosphorylation	biological_process	12	0	0	0	0	0	0	0	0	0	0	0	0
GO:0000002	mitochondrial genome maintenance	biological_process	6	0	0	0	0	0	0	0	0	0	0	0	0
GO:0019915	lipid storage	biological_process	13	0	0	0	0	0	0	0	0	0	0	0	0
GO:0019900	kinase binding	molecular_function	17	0	0	0	0	0	0	0	0	0	0	0	0
GO:0019899	enzyme binding	molecular_function	12	0	0	0	0	0	0	0	0	0	0	0	0
GO:0019896	axonal transport of mitochondrion	biological_process	7	0	0	0	0	0	0	0	0	0	0	0	0

GO:0019894	kinesin binding	molecular_function	9	0	0	0	0	0	0	0	0	0	0	0	0
GO:0019784	NEDD8-specific protease activity	molecular_function	4	0	0	0	0	0	0	0	0	0	0	0	0
GO:0019731	antibacterial humoral response	biological_process	25	0	0	0	0	0	0	0	0	0	0	0	0
GO:0019722	calcium-mediated signaling	biological_process	28	0	0	0	0	0	0	0	0	0	0	0	0
GO:0019706	protein-cysteine S-palmitoyltransferase activity	molecular_function	23	0	0	0	0	0	0	0	0	0	0	0	0
GO:0019464	glycine decarboxylation via glycine cleavage system	biological_process	3	0	0	0	0	0	0	0	0	0	0	0	0
GO:0019432	triglyceride biosynthetic process	biological_process	6	0	0	0	0	0	0	0	0	0	0	0	0
GO:0019348	dolichol metabolic process	biological_process	3	0	0	0	0	0	0	0	0	0	0	0	0
GO:0019254	carnitine metabolic process, CoA-linked	biological_process	3	0	0	0	0	0	0	0	0	0	0	0	0
GO:0019239	deaminase activity	molecular_function	3	0	0	0	0	0	0	0	0	0	0	0	0
GO:0019226	transmission of nerve impulse	biological_process	8	0	0	0	0	0	0	0	0	0	0	0	0
GO:0019216	regulation of lipid metabolic process	biological_process	8	0	0	0	0	0	0	0	0	0	0	0	0
GO:0019101	female somatic sex determination	biological_process	5	0	0	0	0	0	0	0	0	0	0	0	0
GO:0019001	guanyl nucleotide binding	molecular_function	6	0	0	0	0	0	0	0	0	0	0	0	0
GO:0018993	somatic sex determination	biological_process	7	0	0	0	0	0	0	0	0	0	0	0	0
GO:0018991	oviposition	biological_process	12	0	0	0	0	0	0	0	0	0	0	0	0
GO:0018990	ecdysis, chitin-based cuticle	biological_process	21	0	0	0	0	0	0	0	0	0	0	0	0
GO:0018401	peptidyl-proline hydroxylation to 4-hydroxy-L-proline	biological_process	27	0	0	0	0	0	0	0	0	0	0	0	0
GO:0018344	protein geranylgeranylation	biological_process	6	0	0	0	0	0	0	0	0	0	0	0	0
GO:0018342	protein prenylation	biological_process	4	0	0	0	0	0	0	0	0	0	0	0	0
GO:0018230	peptidyl-L-cysteine S-palmitoylation	biological_process	20	0	0	0	0	0	0	0	0	0	0	0	0
GO:0030702	chromatin silencing at centromere	biological_process	5	0	0	0	0	0	0	0	0	0	0	0	0
GO:0030706	germarium-derived oocyte differentiation	biological_process	6	0	0	0	0	0	0	0	0	0	0	0	0
GO:0016616	oxidoreductase activity, acting on the CH-OH group of donors, NAD or NADP as acceptor	molecular_function	17	0	0	0	0	0	0	0	0	0	0	0	0
GO:0032786	positive regulation of DNA-templated transcription, elongation	biological_process	5	0	0	0	0	0	0	0	0	0	0	0	0
GO:0033134	ubiquitin activating enzyme binding	molecular_function	3	0	0	0	0	0	0	0	0	0	0	0	0
GO:0033108	mitochondrial respiratory chain complex assembly	biological_process	4	0	0	0	0	0	0	0	0	0	0	0	0
GO:0033038	bitter taste receptor activity	molecular_function	21	0	0	0	0	0	0	0	0	0	0	0	0
GO:0032981	mitochondrial respiratory chain complex I assembly	biological_process	15	0	0	0	0	0	0	0	0	0	0	0	0
GO:0032958	inositol phosphate biosynthetic process	biological_process	6	0	0	0	0	0	0	0	0	0	0	0	0
GO:0032927	positive regulation of activin receptor signaling pathway	biological_process	4	0	0	0	0	0	0	0	0	0	0	0	0
GO:0032924	activin receptor signaling pathway	biological_process	8	0	0	0	0	0	0	0	0	0	0	0	0
GO:0032922	circadian regulation of gene expression	biological_process	8	0	0	0	0	0	0	0	0	0	0	0	0
GO:0032879	regulation of localization	biological_process	8	0	0	0	0	0	0	0	0	0	0	0	0
GO:0032876	negative regulation of DNA endoreduplication	biological_process	3	0	0	0	0	0	0	0	0	0	0	0	0
GO:0032870	cellular response to hormone stimulus	biological_process	5	0	0	0	0	0	0	0	0	0	0	0	0
GO:0032790	ribosome disassembly	biological_process	5	0	0	0	0	0	0	0	0	0	0	0	0
GO:0032780	negative regulation of ATPase activity	biological_process	4	0	0	0	0	0	0	0	0	0	0	0	0
GO:0032453	histone demethylase activity (H3-K4 specific)	molecular_function	3	0	0	0	0	0	0	0	0	0	0	0	0
GO:0032549	ribonucleoside binding	molecular_function	3	0	0	0	0	0	0	0	0	0	0	0	0
GO:0032529	follicle cell microvillus organization	biological_process	3	0	0	0	0	0	0	0	0	0	0	0	0
GO:0032510	endosome to lysosome transport via multivesicular body sorting pathway	biological_process	4	0	0	0	0	0	0	0	0	0	0	0	0
GO:0032509	endosome transport via multivesicular body sorting pathway	biological_process	18	0	0	0	0	0	0	0	0	0	0	0	0
GO:0032508	DNA duplex unwinding	biological_process	13	0	0	0	0	0	0	0	0	0	0	0	0
GO:0032507	maintenance of protein location in cell	biological_process	7	0	0	0	0	0	0	0	0	0	0	0	0

GO:0032502	developmental process	biological_process	7	0	0	0	0	0	0	0	0	0	0	0	0
GO:0032496	response to lipopolysaccharide	biological_process	3	0	0	0	0	0	0	0	0	0	0	0	0
GO:0032483	regulation of Rab protein signal transduction	biological_process	12	0	0	0	0	0	0	0	0	0	0	0	0
GO:0032469	endoplasmic reticulum calcium ion homeostasis	biological_process	4	0	0	0	0	0	0	0	0	0	0	0	0
GO:0032467	positive regulation of cytokinesis	biological_process	4	0	0	0	0	0	0	0	0	0	0	0	0
GO:0032465	regulation of cytokinesis	biological_process	14	0	0	0	0	0	0	0	0	0	0	0	0
GO:0033149	FFAT motif binding	molecular_function	4	0	0	0	0	0	0	0	0	0	0	0	0
GO:0033209	tumor necrosis factor-mediated signaling pathway	biological_process	3	0	0	0	0	0	0	0	0	0	0	0	0
GO:0033363	secretory granule organization	biological_process	7	0	0	0	0	0	0	0	0	0	0	0	0
GO:0033402	UUC codon-amino acid adaptor activity	molecular_function	8	0	0	0	0	0	0	0	0	0	0	0	0
GO:0034389	lipid particle organization	biological_process	12	0	0	0	0	0	0	0	0	0	0	0	0
GO:0034338	short-chain carboxylesterase activity	molecular_function	3	0	0	0	0	0	0	0	0	0	0	0	0
GO:0034333	adherens junction assembly	biological_process	5	0	0	0	0	0	0	0	0	0	0	0	0
GO:0034332	adherens junction organization	biological_process	11	0	0	0	0	0	0	0	0	0	0	0	0
GO:0034315	regulation of Arp2/3 complex-mediated actin nucleation	biological_process	3	0	0	0	0	0	0	0	0	0	0	0	0
GO:0034237	protein kinase A regulatory subunit binding	molecular_function	5	0	0	0	0	0	0	0	0	0	0	0	0
GO:0034235	GPI anchor binding	molecular_function	4	0	0	0	0	0	0	0	0	0	0	0	0
GO:0034227	tRNA thio-modification	biological_process	4	0	0	0	0	0	0	0	0	0	0	0	0
GO:0034097	response to cytokine	biological_process	4	0	0	0	0	0	0	0	0	0	0	0	0
GO:0034080	CENP-A containing nucleosome assembly	biological_process	5	0	0	0	0	0	0	0	0	0	0	0	0
GO:0034059	response to anoxia	biological_process	4	0	0	0	0	0	0	0	0	0	0	0	0
GO:0034058	endosomal vesicle fusion	biological_process	12	0	0	0	0	0	0	0	0	0	0	0	0
GO:0034046	poly(G) binding	molecular_function	3	0	0	0	0	0	0	0	0	0	0	0	0
GO:0033674	positive regulation of kinase activity	biological_process	22	0	0	0	0	0	0	0	0	0	0	0	0
GO:0033617	mitochondrial respiratory chain complex IV assembly	biological_process	14	0	0	0	0	0	0	0	0	0	0	0	0
GO:0033615	mitochondrial proton-transporting ATP synthase complex assembly	biological_process	3	0	0	0	0	0	0	0	0	0	0	0	0
GO:0033613	activating transcription factor binding	molecular_function	9	0	0	0	0	0	0	0	0	0	0	0	0
GO:0033499	galactose catabolic process via UDP-galactose	biological_process	8	0	0	0	0	0	0	0	0	0	0	0	0
GO:0033462	GGC codon-amino acid adaptor activity	molecular_function	14	0	0	0	0	0	0	0	0	0	0	0	0
GO:0033448	AGG codon-amino acid adaptor activity	molecular_function	3	0	0	0	0	0	0	0	0	0	0	0	0
GO:0033447	AGA codon-amino acid adaptor activity	molecular_function	3	0	0	0	0	0	0	0	0	0	0	0	0
GO:0033428	CAG codon-amino acid adaptor activity	molecular_function	8	0	0	0	0	0	0	0	0	0	0	0	0
GO:0033421	CCU codon-amino acid adaptor activity	molecular_function	7	0	0	0	0	0	0	0	0	0	0	0	0
GO:0033416	UGG codon-amino acid adaptor activity	molecular_function	8	0	0	0	0	0	0	0	0	0	0	0	0
GO:0033405	UCU codon-amino acid adaptor activity	molecular_function	8	0	0	0	0	0	0	0	0	0	0	0	0
GO:0032456	endocytic recycling	biological_process	27	0	0	0	0	0	0	0	0	0	0	0	0
GO:0032452	histone demethylase activity	molecular_function	6	0	0	0	0	0	0	0	0	0	0	0	0
GO:0030709	border follicle cell delamination	biological_process	5	0	0	0	0	0	0	0	0	0	0	0	0
GO:0031104	dendrite regeneration	biological_process	3	0	0	0	0	0	0	0	0	0	0	0	0
GO:0031396	regulation of protein ubiquitination	biological_process	4	0	0	0	0	0	0	0	0	0	0	0	0
GO:0031340	positive regulation of vesicle fusion	biological_process	3	0	0	0	0	0	0	0	0	0	0	0	0
GO:0031297	replication fork processing	biological_process	5	0	0	0	0	0	0	0	0	0	0	0	0
GO:0031290	retinal ganglion cell axon guidance	biological_process	12	0	0	0	0	0	0	0	0	0	0	0	0
GO:0031267	small GTPase binding	molecular_function	3	0	0	0	0	0	0	0	0	0	0	0	0
GO:0031223	auditory behavior	biological_process	15	0	0	0	0	0	0	0	0	0	0	0	0
GO:0031204	posttranslational protein targeting to membrane, translocation	biological_process	4	0	0	0	0	0	0	0	0	0	0	0	0
GO:0031177	phosphopantetheine binding	molecular_function	5	0	0	0	0	0	0	0	0	0	0	0	0

GO:0031175	neuron projection development	biological_process	11	0	0	0	0	0	0	0	0	0	0	0	0
GO:0031124	mRNA 3'-end processing	biological_process	3	0	0	0	0	0	0	0	0	0	0	0	0
GO:0031114	regulation of microtubule depolymerization	biological_process	3	0	0	0	0	0	0	0	0	0	0	0	0
GO:0031110	regulation of microtubule polymerization or depolymerization	biological_process	8	0	0	0	0	0	0	0	0	0	0	0	0
GO:0031081	nuclear pore distribution	biological_process	3	0	0	0	0	0	0	0	0	0	0	0	0
GO:0032450	maltose alpha-glucosidase activity	molecular_function	10	0	0	0	0	0	0	0	0	0	0	0	0
GO:0031072	heat shock protein binding	molecular_function	26	0	0	0	0	0	0	0	0	0	0	0	0
GO:0031062	positive regulation of histone methylation	biological_process	3	0	0	0	0	0	0	0	0	0	0	0	0
GO:0031054	pre-miRNA processing	biological_process	6	0	0	0	0	0	0	0	0	0	0	0	0
GO:0031047	gene silencing by RNA	biological_process	10	0	0	0	0	0	0	0	0	0	0	0	0
GO:0030955	potassium ion binding	molecular_function	6	0	0	0	0	0	0	0	0	0	0	0	0
GO:0030882	lipid antigen binding	molecular_function	3	0	0	0	0	0	0	0	0	0	0	0	0
GO:0030838	positive regulation of actin filament polymerization	biological_process	14	0	0	0	0	0	0	0	0	0	0	0	0
GO:0030728	ovulation	biological_process	7	0	0	0	0	0	0	0	0	0	0	0	0
GO:0030723	ovarian fusome organization	biological_process	11	0	0	0	0	0	0	0	0	0	0	0	0
GO:0030721	spectrosome organization	biological_process	3	0	0	0	0	0	0	0	0	0	0	0	0
GO:0030717	karyosome formation	biological_process	34	0	0	0	0	0	0	0	0	0	0	0	0
GO:0030714	anterior/posterior axis specification, follicular epithelium	biological_process	3	0	0	0	0	0	0	0	0	0	0	0	0
GO:0031491	nucleosome binding	molecular_function	15	0	0	0	0	0	0	0	0	0	0	0	0
GO:0031492	nucleosomal DNA binding	molecular_function	28	0	0	0	0	0	0	0	0	0	0	0	0
GO:0031507	heterochromatin assembly	biological_process	6	0	0	0	0	0	0	0	0	0	0	0	0
GO:0031573	intra-S DNA damage checkpoint	biological_process	8	0	0	0	0	0	0	0	0	0	0	0	0
GO:0032446	protein modification by small protein conjugation	biological_process	8	0	0	0	0	0	0	0	0	0	0	0	0
GO:0032436	positive regulation of proteasomal ubiquitin-dependent protein catabolic process	biological_process	35	0	0	0	0	0	0	0	0	0	0	0	0
GO:0032367	intracellular cholesterol transport	biological_process	7	0	0	0	0	0	0	0	0	0	0	0	0
GO:0032357	oxidized purine DNA binding	molecular_function	3	0	0	0	0	0	0	0	0	0	0	0	0
GO:0032324	molybdopterin cofactor biosynthetic process	biological_process	4	0	0	0	0	0	0	0	0	0	0	0	0
GO:0032266	phosphatidylinositol-3-phosphate binding	molecular_function	14	0	0	0	0	0	0	0	0	0	0	0	0
GO:0032259	methylation	biological_process	5	0	0	0	0	0	0	0	0	0	0	0	0
GO:0032222	regulation of synaptic transmission, cholinergic	biological_process	4	0	0	0	0	0	0	0	0	0	0	0	0
GO:0032095	regulation of response to food	biological_process	3	0	0	0	0	0	0	0	0	0	0	0	0
GO:0032053	ciliary basal body organization	biological_process	7	0	0	0	0	0	0	0	0	0	0	0	0
GO:0032050	clathrin heavy chain binding	molecular_function	4	0	0	0	0	0	0	0	0	0	0	0	0
GO:0032027	myosin light chain binding	molecular_function	5	0	0	0	0	0	0	0	0	0	0	0	0
GO:0032024	positive regulation of insulin secretion	biological_process	6	0	0	0	0	0	0	0	0	0	0	0	0
GO:0032014	positive regulation of ARF protein signal transduction	biological_process	3	0	0	0	0	0	0	0	0	0	0	0	0
GO:0032012	regulation of ARF protein signal transduction	biological_process	4	0	0	0	0	0	0	0	0	0	0	0	0
GO:0032008	positive regulation of TOR signaling	biological_process	16	0	0	0	0	0	0	0	0	0	0	0	0
GO:0031990	mRNA export from nucleus in response to heat stress	biological_process	6	0	0	0	0	0	0	0	0	0	0	0	0
GO:0031937	positive regulation of chromatin silencing	biological_process	13	0	0	0	0	0	0	0	0	0	0	0	0
GO:0031936	negative regulation of chromatin silencing	biological_process	15	0	0	0	0	0	0	0	0	0	0	0	0
GO:0031935	regulation of chromatin silencing	biological_process	13	0	0	0	0	0	0	0	0	0	0	0	0
GO:0031887	lipid particle transport along microtubule	biological_process	4	0	0	0	0	0	0	0	0	0	0	0	0
GO:0031683	G-protein beta/gamma-subunit complex binding	molecular_function	8	0	0	0	0	0	0	0	0	0	0	0	0

GO:0031670	cellular response to nutrient	biological_process	5	0	0	0	0	0	0	0	0	0	0	0	0
GO:0031623	receptor internalization	biological_process	5	0	0	0	0	0	0	0	0	0	0	0	0
GO:0031589	cell-substrate adhesion	biological_process	3	0	0	0	0	0	0	0	0	0	0	0	0
GO:0016618	hydroxypyruvate reductase activity	molecular_function	4	0	0	0	0	0	0	0	0	0	0	0	0
GO:0016584	nucleosome positioning	biological_process	7	0	0	0	0	0	0	0	0	0	0	0	0
GO:0007525	somatic muscle development	biological_process	25	0	0	0	0	0	0	0	0	0	0	0	0
GO:0008420	CTD phosphatase activity	molecular_function	4	0	0	0	0	0	0	0	0	0	0	0	0
GO:0008585	female gonad development	biological_process	11	0	0	0	0	0	0	0	0	0	0	0	0
GO:0008584	male gonad development	biological_process	8	0	0	0	0	0	0	0	0	0	0	0	0
GO:0008582	regulation of synaptic growth at neuromuscular junction	biological_process	35	0	0	0	0	0	0	0	0	0	0	0	0
GO:0008574	ATP-dependent microtubule motor activity, plus-end-directed	molecular_function	14	0	0	0	0	0	0	0	0	0	0	0	0
GO:0008569	ATP-dependent microtubule motor activity, minus-end-directed	molecular_function	16	0	0	0	0	0	0	0	0	0	0	0	0
GO:0008536	Ran GTPase binding	molecular_function	20	0	0	0	0	0	0	0	0	0	0	0	0
GO:0008535	respiratory chain complex IV assembly	biological_process	7	0	0	0	0	0	0	0	0	0	0	0	0
GO:0008525	phosphatidylcholine transporter activity	molecular_function	3	0	0	0	0	0	0	0	0	0	0	0	0
GO:0008499	UDP-galactose:beta-N-acetylglucosamine beta-1,3-galactosyltransferase activity	molecular_function	6	0	0	0	0	0	0	0	0	0	0	0	0
GO:0008494	translation activator activity	molecular_function	5	0	0	0	0	0	0	0	0	0	0	0	0
GO:0008474	palmitoyl-(protein) hydrolase activity	molecular_function	4	0	0	0	0	0	0	0	0	0	0	0	0
GO:0008465	glycerate dehydrogenase activity	molecular_function	4	0	0	0	0	0	0	0	0	0	0	0	0
GO:0008408	3'-5' exonuclease activity	molecular_function	14	0	0	0	0	0	0	0	0	0	0	0	0
GO:0008316	structural constituent of vitelline membrane	molecular_function	3	0	0	0	0	0	0	0	0	0	0	0	0
GO:0008381	mechanically-gated ion channel activity	molecular_function	5	0	0	0	0	0	0	0	0	0	0	0	0
GO:0008376	acetylgalactosaminyltransferase activity	molecular_function	13	0	0	0	0	0	0	0	0	0	0	0	0
GO:0008375	acetylglucosaminyltransferase activity	molecular_function	19	0	0	0	0	0	0	0	0	0	0	0	0
GO:0008374	O-acyltransferase activity	molecular_function	5	0	0	0	0	0	0	0	0	0	0	0	0
GO:0008366	axon ensheathment	biological_process	8	0	0	0	0	0	0	0	0	0	0	0	0
GO:0008365	adult chitin-based cuticle development	biological_process	5	0	0	0	0	0	0	0	0	0	0	0	0
GO:0008363	larval chitin-based cuticle development	biological_process	20	0	0	0	0	0	0	0	0	0	0	0	0
GO:0008362	chitin-based embryonic cuticle biosynthetic process	biological_process	15	0	0	0	0	0	0	0	0	0	0	0	0
GO:0008355	olfactory learning	biological_process	40	0	0	0	0	0	0	0	0	0	0	0	0
GO:0008343	adult feeding behavior	biological_process	13	0	0	0	0	0	0	0	0	0	0	0	0
GO:0008333	endosome to lysosome transport	biological_process	11	0	0	0	0	0	0	0	0	0	0	0	0
GO:0008324	cation transmembrane transporter activity	molecular_function	6	0	0	0	0	0	0	0	0	0	0	0	0
GO:0008593	regulation of Notch signaling pathway	biological_process	8	0	0	0	0	0	0	0	0	0	0	0	0
GO:0008608	attachment of spindle microtubules to kinetochore	biological_process	3	0	0	0	0	0	0	0	0	0	0	0	0
GO:0008613	diuretic hormone activity	molecular_function	4	0	0	0	0	0	0	0	0	0	0	0	0
GO:0008630	intrinsic apoptotic signaling pathway in response to DNA damage	biological_process	11	0	0	0	0	0	0	0	0	0	0	0	0
GO:0009744	response to sucrose	biological_process	18	0	0	0	0	0	0	0	0	0	0	0	0
GO:0009725	response to hormone	biological_process	3	0	0	0	0	0	0	0	0	0	0	0	0
GO:0009653	anatomical structure morphogenesis	biological_process	19	0	0	0	0	0	0	0	0	0	0	0	0
GO:0009651	response to salt stress	biological_process	9	0	0	0	0	0	0	0	0	0	0	0	0
GO:0009649	entrainment of circadian clock	biological_process	13	0	0	0	0	0	0	0	0	0	0	0	0
GO:0009617	response to bacterium	biological_process	44	0	0	0	0	0	0	0	0	0	0	0	0
GO:0009611	response to wounding	biological_process	21	0	0	0	0	0	0	0	0	0	0	0	0
GO:0009435	NAD biosynthetic process	biological_process	4	0	0	0	0	0	0	0	0	0	0	0	0

GO:0009411	response to UV	biological_process	15	0	0	0	0	0	0	0	0	0	0	0	0
GO:0009409	response to cold	biological_process	6	0	0	0	0	0	0	0	0	0	0	0	0
GO:0009396	folic acid-containing compound biosynthetic process	biological_process	3	0	0	0	0	0	0	0	0	0	0	0	0
GO:0009378	four-way junction helicase activity	molecular_function	5	0	0	0	0	0	0	0	0	0	0	0	0
GO:0009311	oligosaccharide metabolic process	biological_process	4	0	0	0	0	0	0	0	0	0	0	0	0
GO:0009266	response to temperature stimulus	biological_process	6	0	0	0	0	0	0	0	0	0	0	0	0
GO:0009247	glycolipid biosynthetic process	biological_process	6	0	0	0	0	0	0	0	0	0	0	0	0
GO:0009190	cyclic nucleotide biosynthetic process	biological_process	10	0	0	0	0	0	0	0	0	0	0	0	0
GO:0009166	nucleotide catabolic process	biological_process	4	0	0	0	0	0	0	0	0	0	0	0	0
GO:0009116	nucleoside metabolic process	biological_process	16	0	0	0	0	0	0	0	0	0	0	0	0
GO:0009113	purine nucleobase biosynthetic process	biological_process	6	0	0	0	0	0	0	0	0	0	0	0	0
GO:0009086	methionine biosynthetic process	biological_process	5	0	0	0	0	0	0	0	0	0	0	0	0
GO:0009047	dosage compensation by hyperactivation of X chromosome	biological_process	15	0	0	0	0	0	0	0	0	0	0	0	0
GO:0008821	crossover junction endodeoxyribonuclease activity	molecular_function	5	0	0	0	0	0	0	0	0	0	0	0	0
GO:0008800	beta-lactamase activity	molecular_function	4	0	0	0	0	0	0	0	0	0	0	0	0
GO:0008757	S-adenosylmethionine-dependent methyltransferase activity	molecular_function	6	0	0	0	0	0	0	0	0	0	0	0	0
GO:0008637	apoptotic mitochondrial changes	biological_process	5	0	0	0	0	0	0	0	0	0	0	0	0
GO:0008320	protein transmembrane transporter activity	molecular_function	9	0	0	0	0	0	0	0	0	0	0	0	0
GO:0008315	meiotic G2/MI transition	biological_process	3	0	0	0	0	0	0	0	0	0	0	0	0
GO:0009792	embryo development ending in birth or egg hatching	biological_process	19	0	0	0	0	0	0	0	0	0	0	0	0
GO:0007628	adult walking behavior	biological_process	20	0	0	0	0	0	0	0	0	0	0	0	0
GO:0008063	Toll signaling pathway	biological_process	11	0	0	0	0	0	0	0	0	0	0	0	0
GO:0008062	eclosion rhythm	biological_process	9	0	0	0	0	0	0	0	0	0	0	0	0
GO:0008056	ocellus development	biological_process	4	0	0	0	0	0	0	0	0	0	0	0	0
GO:0008049	male courtship behavior	biological_process	64	0	0	0	0	0	0	0	0	0	0	0	0
GO:0008045	motor neuron axon guidance	biological_process	79	0	0	0	0	0	0	0	0	0	0	0	0
GO:0008038	neuron recognition	biological_process	6	0	0	0	0	0	0	0	0	0	0	0	0
GO:0008033	tRNA processing	biological_process	13	0	0	0	0	0	0	0	0	0	0	0	0
GO:0008022	protein C-terminus binding	molecular_function	3	0	0	0	0	0	0	0	0	0	0	0	0
GO:0008016	regulation of heart contraction	biological_process	7	0	0	0	0	0	0	0	0	0	0	0	0
GO:0008011	structural constituent of pupal chitin-based cuticle	molecular_function	5	0	0	0	0	0	0	0	0	0	0	0	0
GO:0007631	feeding behavior	biological_process	5	0	0	0	0	0	0	0	0	0	0	0	0
GO:0007629	flight behavior	biological_process	17	0	0	0	0	0	0	0	0	0	0	0	0
GO:0007620	copulation	biological_process	9	0	0	0	0	0	0	0	0	0	0	0	0
GO:0008311	double-stranded DNA 3'-5' exodeoxyribonuclease activity	molecular_function	4	0	0	0	0	0	0	0	0	0	0	0	0
GO:0007619	courtship behavior	biological_process	24	0	0	0	0	0	0	0	0	0	0	0	0
GO:0007618	mating	biological_process	3	0	0	0	0	0	0	0	0	0	0	0	0
GO:0007617	mating behavior	biological_process	14	0	0	0	0	0	0	0	0	0	0	0	0
GO:0007614	short-term memory	biological_process	29	0	0	0	0	0	0	0	0	0	0	0	0
GO:0007594	puparial adhesion	biological_process	9	0	0	0	0	0	0	0	0	0	0	0	0
GO:0007593	chitin-based cuticle sclerotization	biological_process	4	0	0	0	0	0	0	0	0	0	0	0	0
GO:0007591	molting cycle, chitin-based cuticle	biological_process	12	0	0	0	0	0	0	0	0	0	0	0	0
GO:0007589	body fluid secretion	biological_process	5	0	0	0	0	0	0	0	0	0	0	0	0
GO:0007568	aging	biological_process	4	0	0	0	0	0	0	0	0	0	0	0	0
GO:0007560	imaginal disc morphogenesis	biological_process	10	0	0	0	0	0	0	0	0	0	0	0	0
GO:0007548	sex differentiation	biological_process	13	0	0	0	0	0	0	0	0	0	0	0	0
GO:0007541	sex determination, primary response to X:A ratio	biological_process	3	0	0	0	0	0	0	0	0	0	0	0	0
GO:0008069	dorsal/ventral axis specification, ovarian follicular epithelium	biological_process	14	0	0	0	0	0	0	0	0	0	0	0	0

GO:0008080	N-acetyltransferase activity	molecular_function	16	0	0	0	0	0	0	0	0	0	0	0	0
GO:0008081	phosphoric diester hydrolase activity	molecular_function	9	0	0	0	0	0	0	0	0	0	0	0	0
GO:0008084	imaginal disc growth factor receptor binding	molecular_function	6	0	0	0	0	0	0	0	0	0	0	0	0
GO:0008308	voltage-gated anion channel activity	molecular_function	5	0	0	0	0	0	0	0	0	0	0	0	0
GO:0008298	intracellular mRNA localization	biological_process	21	0	0	0	0	0	0	0	0	0	0	0	0
GO:0008296	3'-5'-exodeoxyribonuclease activity	molecular_function	7	0	0	0	0	0	0	0	0	0	0	0	0
GO:0008285	negative regulation of cell proliferation	biological_process	40	0	0	0	0	0	0	0	0	0	0	0	0
GO:0008283	cell proliferation	biological_process	7	0	0	0	0	0	0	0	0	0	0	0	0
GO:0008276	protein methyltransferase activity	molecular_function	4	0	0	0	0	0	0	0	0	0	0	0	0
GO:0008273	calcium, potassium:sodium antiporter activity	molecular_function	5	0	0	0	0	0	0	0	0	0	0	0	0
GO:0008266	poly(U) RNA binding	molecular_function	10	0	0	0	0	0	0	0	0	0	0	0	0
GO:0008251	tRNA-specific adenosine deaminase activity	molecular_function	5	0	0	0	0	0	0	0	0	0	0	0	0
GO:0008241	peptidyl-dipeptidase activity	molecular_function	6	0	0	0	0	0	0	0	0	0	0	0	0
GO:0008238	exopeptidase activity	molecular_function	6	0	0	0	0	0	0	0	0	0	0	0	0
GO:0008237	metallopeptidase activity	molecular_function	21	0	0	0	0	0	0	0	0	0	0	0	0
GO:0008219	cell death	biological_process	20	0	0	0	0	0	0	0	0	0	0	0	0
GO:0008194	UDP-glycosyltransferase activity	molecular_function	35	0	0	0	0	0	0	0	0	0	0	0	0
GO:0008190	eukaryotic initiation factor 4E binding	molecular_function	6	0	0	0	0	0	0	0	0	0	0	0	0
GO:0008175	tRNA methyltransferase activity	molecular_function	5	0	0	0	0	0	0	0	0	0	0	0	0
GO:0008160	protein tyrosine phosphatase activator activity	molecular_function	3	0	0	0	0	0	0	0	0	0	0	0	0
GO:0008156	negative regulation of DNA replication	biological_process	7	0	0	0	0	0	0	0	0	0	0	0	0
GO:0008146	sulfotransferase activity	molecular_function	11	0	0	0	0	0	0	0	0	0	0	0	0
GO:0008137	NADH dehydrogenase (ubiquinone) activity	molecular_function	24	0	0	0	0	0	0	0	0	0	0	0	0
GO:0008103	oocyte microtubule cytoskeleton polarization	biological_process	19	0	0	0	0	0	0	0	0	0	0	0	0
GO:0008097	5S rRNA binding	molecular_function	3	0	0	0	0	0	0	0	0	0	0	0	0
GO:0008093	cytoskeletal adaptor activity	molecular_function	3	0	0	0	0	0	0	0	0	0	0	0	0
GO:0008092	cytoskeletal protein binding	molecular_function	7	0	0	0	0	0	0	0	0	0	0	0	0
GO:0008090	retrograde axonal transport	biological_process	9	0	0	0	0	0	0	0	0	0	0	0	0
GO:0009786	regulation of asymmetric cell division	biological_process	3	0	0	0	0	0	0	0	0	0	0	0	0
GO:0009880	embryonic pattern specification	biological_process	23	0	0	0	0	0	0	0	0	0	0	0	0
GO:0016578	histone deubiquitination	biological_process	6	0	0	0	0	0	0	0	0	0	0	0	0
GO:0015918	sterol transport	biological_process	9	0	0	0	0	0	0	0	0	0	0	0	0
GO:0016081	synaptic vesicle docking	biological_process	23	0	0	0	0	0	0	0	0	0	0	0	0
GO:0016080	synaptic vesicle targeting	biological_process	10	0	0	0	0	0	0	0	0	0	0	0	0
GO:0016079	synaptic vesicle exocytosis	biological_process	22	0	0	0	0	0	0	0	0	0	0	0	0
GO:0016062	adaptation of rhodopsin mediated signaling	biological_process	4	0	0	0	0	0	0	0	0	0	0	0	0
GO:0016060	metarhodopsin inactivation	biological_process	5	0	0	0	0	0	0	0	0	0	0	0	0
GO:0016059	deactivation of rhodopsin mediated signaling	biological_process	14	0	0	0	0	0	0	0	0	0	0	0	0
GO:0016057	regulation of membrane potential in photoreceptor cell	biological_process	7	0	0	0	0	0	0	0	0	0	0	0	0
GO:0016051	carbohydrate biosynthetic process	biological_process	4	0	0	0	0	0	0	0	0	0	0	0	0
GO:0016042	lipid catabolic process	biological_process	42	0	0	0	0	0	0	0	0	0	0	0	0
GO:0016036	cellular response to phosphate starvation	biological_process	4	0	0	0	0	0	0	0	0	0	0	0	0
GO:0015986	ATP synthesis coupled proton transport	biological_process	19	0	0	0	0	0	0	0	0	0	0	0	0
GO:0015937	coenzyme A biosynthetic process	biological_process	6	0	0	0	0	0	0	0	0	0	0	0	0
GO:0015858	nucleoside transport	biological_process	3	0	0	0	0	0	0	0	0	0	0	0	0
GO:0015171	amino acid transmembrane transporter activity	molecular_function	30	0	0	0	0	0	0	0	0	0	0	0	0
GO:0015813	L-glutamate transport	biological_process	5	0	0	0	0	0	0	0	0	0	0	0	0
GO:0015810	aspartate transport	biological_process	4	0	0	0	0	0	0	0	0	0	0	0	0

GO:0015701	bicarbonate transport	biological_process	3	0	0	0	0	0	0	0	0	0	0	0	0
GO:0015631	tubulin binding	molecular_function	12	0	0	0	0	0	0	0	0	0	0	0	0
GO:0015386	potassium:proton antiporter activity	molecular_function	4	0	0	0	0	0	0	0	0	0	0	0	0
GO:0015385	sodium:proton antiporter activity	molecular_function	5	0	0	0	0	0	0	0	0	0	0	0	0
GO:0015379	potassium:chloride symporter activity	molecular_function	5	0	0	0	0	0	0	0	0	0	0	0	0
GO:0015347	sodium-independent organic anion transmembrane transporter activity	molecular_function	8	0	0	0	0	0	0	0	0	0	0	0	0
GO:0015279	store-operated calcium channel activity	molecular_function	5	0	0	0	0	0	0	0	0	0	0	0	0
GO:0015277	kainate selective glutamate receptor activity	molecular_function	10	0	0	0	0	0	0	0	0	0	0	0	0
GO:0015267	channel activity	molecular_function	9	0	0	0	0	0	0	0	0	0	0	0	0
GO:0015183	L-aspartate transmembrane transporter activity	molecular_function	5	0	0	0	0	0	0	0	0	0	0	0	0
GO:0016139	glycoside catabolic process	biological_process	4	0	0	0	0	0	0	0	0	0	0	0	0
GO:0016174	NAD(P)H oxidase activity	molecular_function	3	0	0	0	0	0	0	0	0	0	0	0	0
GO:0016185	synaptic vesicle budding from presynaptic endocytic zone membrane	biological_process	3	0	0	0	0	0	0	0	0	0	0	0	0
GO:0016201	synaptic target inhibition	biological_process	15	0	0	0	0	0	0	0	0	0	0	0	0
GO:0016577	histone demethylation	biological_process	5	0	0	0	0	0	0	0	0	0	0	0	0
GO:0016572	histone phosphorylation	biological_process	7	0	0	0	0	0	0	0	0	0	0	0	0
GO:0016559	peroxisome fission	biological_process	5	0	0	0	0	0	0	0	0	0	0	0	0
GO:0016558	protein import into peroxisome matrix	biological_process	8	0	0	0	0	0	0	0	0	0	0	0	0
GO:0016540	protein autoprocessing	biological_process	7	0	0	0	0	0	0	0	0	0	0	0	0
GO:0016531	copper chaperone activity	molecular_function	3	0	0	0	0	0	0	0	0	0	0	0	0
GO:0016462	pyrophosphatase activity	molecular_function	3	0	0	0	0	0	0	0	0	0	0	0	0
GO:0016458	gene silencing	biological_process	23	0	0	0	0	0	0	0	0	0	0	0	0
GO:0016457	dosage compensation complex assembly involved in dosage compensation by hyperactivation of X chromosome	biological_process	5	0	0	0	0	0	0	0	0	0	0	0	0
GO:0016441	posttranscriptional gene silencing	biological_process	7	0	0	0	0	0	0	0	0	0	0	0	0
GO:0016407	acetyltransferase activity	molecular_function	4	0	0	0	0	0	0	0	0	0	0	0	0
GO:0016358	dendrite development	biological_process	13	0	0	0	0	0	0	0	0	0	0	0	0
GO:0016339	calcium-dependent cell-cell adhesion via plasma membrane cell adhesion molecules	biological_process	20	0	0	0	0	0	0	0	0	0	0	0	0
GO:0016336	establishment or maintenance of polarity of larval imaginal disc epithelium	biological_process	5	0	0	0	0	0	0	0	0	0	0	0	0
GO:0016334	establishment or maintenance of polarity of follicular epithelium	biological_process	10	0	0	0	0	0	0	0	0	0	0	0	0
GO:0016333	morphogenesis of follicular epithelium	biological_process	10	0	0	0	0	0	0	0	0	0	0	0	0
GO:0016325	oocyte microtubule cytoskeleton organization	biological_process	16	0	0	0	0	0	0	0	0	0	0	0	0
GO:0016322	neuron remodeling	biological_process	43	0	0	0	0	0	0	0	0	0	0	0	0
GO:0016310	phosphorylation	biological_process	16	0	0	0	0	0	0	0	0	0	0	0	0
GO:0016301	kinase activity	molecular_function	21	0	0	0	0	0	0	0	0	0	0	0	0
GO:0016298	lipase activity	molecular_function	40	0	0	0	0	0	0	0	0	0	0	0	0
GO:0016242	negative regulation of macroautophagy	biological_process	7	0	0	0	0	0	0	0	0	0	0	0	0
GO:0016236	macroautophagy	biological_process	16	0	0	0	0	0	0	0	0	0	0	0	0
GO:0016233	telomere capping	biological_process	13	0	0	0	0	0	0	0	0	0	0	0	0
GO:0016226	iron-sulfur cluster assembly	biological_process	20	0	0	0	0	0	0	0	0	0	0	0	0
GO:0015179	L-amino acid transmembrane transporter activity	molecular_function	12	0	0	0	0	0	0	0	0	0	0	0	0
GO:0015114	phosphate ion transmembrane transporter activity	molecular_function	8	0	0	0	0	0	0	0	0	0	0	0	0
GO:0009887	animal organ morphogenesis	biological_process	14	0	0	0	0	0	0	0	0	0	0	0	0
GO:0010037	response to carbon dioxide	biological_process	3	0	0	0	0	0	0	0	0	0	0	0	0
GO:0010529	negative regulation of transposition	biological_process	14	0	0	0	0	0	0	0	0	0	0	0	0
GO:0010526	negative regulation of transposition, RNA-mediated	biological_process	5	0	0	0	0	0	0	0	0	0	0	0	0
GO:0010506	regulation of autophagy	biological_process	37	0	0	0	0	0	0	0	0	0	0	0	0

GO:0010485	H4 histone acetyltransferase activity	molecular_function	8	0	0	0	0	0	0	0	0	0	0	0	0
GO:0010470	regulation of gastrulation	biological_process	5	0	0	0	0	0	0	0	0	0	0	0	0
GO:0010459	negative regulation of heart rate	biological_process	3	0	0	0	0	0	0	0	0	0	0	0	0
GO:0010457	centriole-centriole cohesion	biological_process	4	0	0	0	0	0	0	0	0	0	0	0	0
GO:0010378	temperature compensation of the circadian clock	biological_process	6	0	0	0	0	0	0	0	0	0	0	0	0
GO:0010312	detoxification of zinc ion	biological_process	12	0	0	0	0	0	0	0	0	0	0	0	0
GO:0010259	multicellular organism aging	biological_process	5	0	0	0	0	0	0	0	0	0	0	0	0
GO:0010212	response to ionizing radiation	biological_process	14	0	0	0	0	0	0	0	0	0	0	0	0
GO:0010181	FMN binding	molecular_function	11	0	0	0	0	0	0	0	0	0	0	0	0
GO:0010032	meiotic chromosome condensation	biological_process	7	0	0	0	0	0	0	0	0	0	0	0	0
GO:0015095	magnesium ion transmembrane transporter activity	molecular_function	3	0	0	0	0	0	0	0	0	0	0	0	0
GO:0010004	gastrulation involving germ band extension	biological_process	13	0	0	0	0	0	0	0	0	0	0	0	0
GO:0010002	cardioblast differentiation	biological_process	8	0	0	0	0	0	0	0	0	0	0	0	0
GO:0009996	negative regulation of cell fate specification	biological_process	4	0	0	0	0	0	0	0	0	0	0	0	0
GO:0009994	oocyte differentiation	biological_process	4	0	0	0	0	0	0	0	0	0	0	0	0
GO:0009987	cellular process	biological_process	4	0	0	0	0	0	0	0	0	0	0	0	0
GO:0009972	cytidine deamination	biological_process	3	0	0	0	0	0	0	0	0	0	0	0	0
GO:0009966	regulation of signal transduction	biological_process	9	0	0	0	0	0	0	0	0	0	0	0	0
GO:0009952	anterior/posterior pattern specification	biological_process	29	0	0	0	0	0	0	0	0	0	0	0	0
GO:0009950	dorsal/ventral axis specification	biological_process	25	0	0	0	0	0	0	0	0	0	0	0	0
GO:0009948	anterior/posterior axis specification	biological_process	5	0	0	0	0	0	0	0	0	0	0	0	0
GO:0009913	epidermal cell differentiation	biological_process	7	0	0	0	0	0	0	0	0	0	0	0	0
GO:0009888	tissue development	biological_process	17	0	0	0	0	0	0	0	0	0	0	0	0
GO:0010569	regulation of double-strand break repair via homologous recombination	biological_process	4	0	0	0	0	0	0	0	0	0	0	0	0
GO:0010586	miRNA metabolic process	biological_process	3	0	0	0	0	0	0	0	0	0	0	0	0
GO:0010591	regulation of lamellipodium assembly	biological_process	7	0	0	0	0	0	0	0	0	0	0	0	0
GO:0010623	programmed cell death involved in cell development	biological_process	9	0	0	0	0	0	0	0	0	0	0	0	0
GO:0015037	peptide disulfide oxidoreductase activity	molecular_function	5	0	0	0	0	0	0	0	0	0	0	0	0
GO:0015036	disulfide oxidoreductase activity	molecular_function	11	0	0	0	0	0	0	0	0	0	0	0	0
GO:0015035	protein disulfide oxidoreductase activity	molecular_function	18	0	0	0	0	0	0	0	0	0	0	0	0
GO:0015031	protein transport	biological_process	20	0	0	0	0	0	0	0	0	0	0	0	0
GO:0015026	coreceptor activity	molecular_function	7	0	0	0	0	0	0	0	0	0	0	0	0
GO:0015020	glucuronosyltransferase activity	molecular_function	6	0	0	0	0	0	0	0	0	0	0	0	0
GO:0015018	galactosylgalactosylxylosylprotein 3-beta-glucuronosyltransferase activity	molecular_function	3	0	0	0	0	0	0	0	0	0	0	0	0
GO:0015014	heparan sulfate proteoglycan biosynthetic process, polysaccharide chain biosynthetic process	biological_process	6	0	0	0	0	0	0	0	0	0	0	0	0
GO:0014068	positive regulation of phosphatidylinositol 3-kinase signaling	biological_process	9	0	0	0	0	0	0	0	0	0	0	0	0
GO:0014067	negative regulation of phosphatidylinositol 3-kinase signaling	biological_process	9	0	0	0	0	0	0	0	0	0	0	0	0
GO:0014019	neuroblast development	biological_process	21	0	0	0	0	0	0	0	0	0	0	0	0
GO:0014016	neuroblast differentiation	biological_process	3	0	0	0	0	0	0	0	0	0	0	0	0
GO:0010996	response to auditory stimulus	biological_process	6	0	0	0	0	0	0	0	0	0	0	0	0
GO:0010976	positive regulation of neuron projection development	biological_process	4	0	0	0	0	0	0	0	0	0	0	0	0
GO:0010971	positive regulation of G2/M transition of mitotic cell cycle	biological_process	4	0	0	0	0	0	0	0	0	0	0	0	0
GO:0010951	negative regulation of endopeptidase activity	biological_process	33	0	0	0	0	0	0	0	0	0	0	0	0
GO:0010942	positive regulation of cell death	biological_process	4	0	0	0	0	0	0	0	0	0	0	0	0

GO:0010897	negative regulation of triglyceride catabolic process	biological_process	4	0	0	0	0	0	0	0	0	0	0	0	0
GO:0010888	negative regulation of lipid storage	biological_process	9	0	0	0	0	0	0	0	0	0	0	0	0
GO:0010862	positive regulation of pathway-restricted SMAD protein phosphorylation	biological_process	7	0	0	0	0	0	0	0	0	0	0	0	0
GO:0010821	regulation of mitochondrion organization	biological_process	10	0	0	0	0	0	0	0	0	0	0	0	0
GO:0010737	protein kinase A signaling	biological_process	3	0	0	0	0	0	0	0	0	0	0	0	0
GO:0010669	epithelial structure maintenance	biological_process	6	0	0	0	0	0	0	0	0	0	0	0	0
GO:0010636	positive regulation of mitochondrial fusion	biological_process	4	0	0	0	0	0	0	0	0	0	0	0	0
GO:0010629	negative regulation of gene expression	biological_process	68	0	0	0	0	0	0	0	0	0	0	0	0
GO:2001070	starch binding	molecular_function	5	0	0	0	0	0	0	0	0	0	0	0	0

Note:
Statistical significance was calculated by exact binomial test.

Supplementary Table 3. Canonical Pathways from orthologs of human highlanders

Ingenuity Canonical Pathways	-log(p-value)	Molecules
Amyotrophic Lateral Sclerosis Signaling	14.1	GRIA1,GRIA4,GRID2,GRIK2,PDGFC,PGF,PIK3CA,PIK3CB,PIK3CG,VEGFA, VEGFB,VEGFC
Clathrin-mediated Endocytosis Signaling	10.5	FGF13,PDGFB,PDGFC,PGF,PIK3CA,PIK3CB,PIK3CG,PPP3CB,TF,VEGFA, VEGFB,VEGFC
Actin Cytoskeleton Signaling	9.86	ACTN1,FGF13,MYLK3,PAK3,PDGFB,PDGFC,PIK3CA,PIK3CB,PIK3CG,SSH2,VAV2 ,VAV3
Nitric Oxide Signaling in the Cardiovascular System	9.43	PDGFC,PGF,PIK3CA,PIK3CB,PIK3CG,PRKCE,VEGFA,VEGFB,VEGFC
VEGF Signaling	9.43	ACTN1,PDGFC,PGF,PIK3CA,PIK3CB,PIK3CG,VEGFA,VEGFB,VEGFC
Ovarian Cancer Signaling	9.42	FSHR,PDGFC,PGF,PIK3CA,PIK3CB,PIK3CG,VEGFA,VEGFB,VEGFC,WNT8B
Hepatic Fibrosis Signaling Pathway	9.31	MAP2K5,MYLK3,PDGFB,PDGFC,PGF,PIK3CA,PIK3CB,PIK3CG,PRKCE,TF, VEGFA,VEGFB,VEGFC,WNT8B
Pancreatic Adenocarcinoma Signaling	9.05	CYP2E1,PDGFC,PGF,PIK3CA,PIK3CB,PIK3CG,VEGFA,VEGFB,VEGFC
eNOS Signaling	8.84	CHRNA3,PDGFC,PGF,PIK3CA,PIK3CB,PIK3CG,PRKCE,VEGFA,VEGFB, VEGFC
VEGF Family Ligand-Receptor Interactions	8.6	PGF,PIK3CA,PIK3CB,PIK3CG,PRKCE,VEGFA,VEGFB,VEGFC
Role of Macrophages, Fibroblasts and Endothelial Cells in Rheumatoid Arthritis	8.09	PDGFB,PDGFC,PGF,PIK3CA,PIK3CB,PIK3CG,PPP3CB,PRKCE,VEGFA, VEGFB,VEGFC,WNT8B
Endocannabinoid Cancer Inhibition Pathway	8.01	MAP2K5,PDGFC,PGF,PIK3CA,PIK3CB,PIK3CG,VEGFA,VEGFB,VEGFC
Axonal Guidance Signaling	7.79	LRRC4C,PAK3,PDGFB,PDGFC,PGF,PIK3CA,PIK3CB,PIK3CG,PPP3CB, PRKCE,VEGFA,VEGFB,VEGFC,WNT8B
HIF1α Signaling	7.57	PDGFC,PGF,PIK3CA,PIK3CB,PIK3CG,VEGFA,VEGFB,VEGFC
Macropinocytosis Signaling	7.48	HGF,PDGFB,PDGFC,PIK3CA,PIK3CB,PIK3CG,PRKCE
IL-3 Signaling	7.36	INPP5D,PIK3CA,PIK3CB,PIK3CG,PPP3CB,PRKCE,STAT5B
Renal Cell Carcinoma Signaling	7.32	HGF,PAK3,PDGFB,PIK3CA,PIK3CB,PIK3CG,VEGFA
Hepatic Fibrosis / Hepatic Stellate Cell Activation	7.02	CYP2E1,ECE1,HGF,PDGFB,PDGFC,PGF,VEGFA,VEGFB,VEGFC
ILK Signaling	6.94	ACTN1,PDGFC,PGF,PIK3CA,PIK3CB,PIK3CG,VEGFA,VEGFB,VEGFC
Gap Junction Signaling	6.79	GRIA1,GRIA4,GRIK2,MAP2K5,PIK3CA,PIK3CB,PIK3CG,PPP3CB,PRKCE
IL-8 Signaling	6.75	PDGFC,PGF,PIK3CA,PIK3CB,PIK3CG,PRKCE,VEGFA,VEGFB,VEGFC
mTOR Signaling	6.57	PDGFC,PGF,PIK3CA,PIK3CB,PIK3CG,PRKCE,VEGFA,VEGFB,VEGFC
Tec Kinase Signaling	6.32	PAK3,PIK3CA,PIK3CB,PIK3CG,PRKCE,STAT5B,VAV2,VAV3
GM-CSF Signaling	6.29	PIK3CA,PIK3CB,PIK3CG,PPP3CB,RUNX1,STAT5B
Fc Epsilon RI Signaling	6.18	INPP5D,PIK3CA,PIK3CB,PIK3CG,PRKCE,VAV2,VAV3
Ephrin Receptor Signaling	6.01	PAK3,PDGFB,PDGFC,PGF,PIK3CG,VEGFA,VEGFB,VEGFC
Breast Cancer Regulation by Stathmin1	5.91	ARHGEF9,FSHR,HGF,PDGFB,PDGFC,PGF,PIK3CA,PIK3CB,PIK3CG,PRKCE, VEGFA,VEGFB,VEGFC
Colorectal Cancer Metastasis Signaling	5.89	PDGFC,PGF,PIK3CA,PIK3CB,PIK3CG,VEGFA,VEGFB,VEGFC,WNT8B
NRF2-mediated Oxidative Stress Response	5.85	ABCC4,DNAJA3,DNAJB5,MAP2K5,PIK3CA,PIK3CB,PIK3CG,PRKCE
Ephrin A Signaling	5.79	PIK3CA,PIK3CB,PIK3CG,VAV2,VAV3
PDGF Signaling	5.76	INPP5D,PDGFB,PDGFC,PIK3CA,PIK3CB,PIK3CG
PI3K Signaling in B Lymphocytes	5.7	INPP5D,PIK3CA,PIK3CB,PIK3CG,PPP3CB,VAV2,VAV3
Acute Myeloid Leukemia Signaling	5.67	MAP2K5,PIK3CA,PIK3CB,PIK3CG,RUNX1,STAT5B
CREB Signaling in Neurons	5.56	GRIA1,GRIA4,GRID2,GRIK2,PIK3CA,PIK3CB,PIK3CG,PRKCE
Bladder Cancer Signaling	5.45	FGF13,PDGFC,PGF,VEGFA,VEGFB,VEGFC
PAK Signaling	5.45	PAK3,PDGFB,PDGFC,PIK3CA,PIK3CB,PIK3CG
Glutamate Receptor Signaling	5.37	GRIA1,GRIA4,GRID2,GRIK2,SLC17A1
Neuropathic Pain Signaling In Dorsal Horn Neurons	5.35	GRIA1,GRIA4,PIK3CA,PIK3CB,PIK3CG,PRKCE
T Cell Receptor Signaling	5.25	PIK3CA,PIK3CB,PIK3CG,PPP3CB,VAV2,VAV3
Thrombopoietin Signaling	5.15	PIK3CA,PIK3CB,PIK3CG,PRKCE,STAT5B
Glioma Signaling	5.13	PDGFB,PDGFC,PIK3CA,PIK3CB,PIK3CG,PRKCE
Role of Tissue Factor in Cancer	4.98	PIK3CA,PIK3CB,PIK3CG,STAT5B,VEGFA,VEGFC
Estrogen Receptor Signaling	4.97	PDGFC,PGF,PIK3CA,PIK3CB,PIK3CG,PRKCE,VEGFA,VEGFB,VEGFC
IL-9 Signaling	4.95	PIK3CA,PIK3CB,PIK3CG,STAT5B
Growth Hormone Signaling	4.9	PIK3CA,PIK3CB,PIK3CG,PRKCE,STAT5B
B Cell Receptor Signaling	4.86	INPP5D,PIK3CA,PIK3CB,PIK3CG,PPP3CB,VAV2,VAV3
Angiopoietin Signaling	4.78	PAK3,PIK3CA,PIK3CB,PIK3CG,STAT5B
Regulation of the Epithelial-Mesenchymal Transition Pathway	4.76	FGF13,HGF,MAP2K5,PIK3CA,PIK3CB,PIK3CG,WNT8B
Erythropoietin Signaling	4.75	PIK3CA,PIK3CB,PIK3CG,PRKCE,STAT5B
RAR Activation	4.73	PIK3CA,PIK3CB,PIK3CG,PRKCE,SMARCD3,STAT5B,VEGFA
IL-7 Signaling Pathway	4.7	HGF,PIK3CA,PIK3CB,PIK3CG,STAT5B
Leukocyte Extravasation Signaling	4.69	ACTN1,PIK3CA,PIK3CB,PIK3CG,PRKCE,VAV2,VAV3
Superpathway of Inositol Phosphate Compounds	4.67	DUSP5,INPP5D,ITPKB,MTMR8,PIK3CA,PIK3CB,PIK3CG
BEX2 Signaling Pathway	4.67	PDGFC,PGF,VEGFA,VEGFB,VEGFC
FLT3 Signaling in Hematopoietic Progenitor Cells	4.64	INPP5D,PIK3CA,PIK3CB,PIK3CG,STAT5B
Human Embryonic Stem Cell Pluripotency	4.63	PDGFB,PDGFC,PIK3CA,PIK3CB,PIK3CG,WNT8B
Prolactin Signaling	4.62	PIK3CA,PIK3CB,PIK3CG,PRKCE,STAT5B
FGF Signaling	4.54	FGF13,HGF,PIK3CA,PIK3CB,PIK3CG
Osteoarthritis Pathway	4.49	ANXA5,PDGFC,PGF,VEGFA,VEGFB,VEGFC,WNT8B
Integrin Signaling	4.47	ACTN1,MYLK3,PAK3,PDGFB,PIK3CA,PIK3CB,PIK3CG
IL-23 Signaling Pathway	4.45	PIK3CA,PIK3CB,PIK3CG,RUNX1
Fcγ Receptor-mediated Phagocytosis in Macrophages and Monocytes	4.3	INPP5D,PIK3CG,PRKCE,VAV2,VAV3
ErbB Signaling	4.3	PAK3,PIK3CA,PIK3CB,PIK3CG,PRKCE
PKCθ Signaling in T Lymphocytes	4.29	PIK3CA,PIK3CB,PIK3CG,PPP3CB,VAV2,VAV3
UVB-Induced MAPK Signaling	4.16	PIK3CA,PIK3CB,PIK3CG,PRKCE
Glioblastoma Multiforme Signaling	4.14	PDGFB,PDGFC,PIK3CA,PIK3CB,PIK3CG,WNT8B
Signaling by Rho Family GTPases	4.1	ARHGEF9,PAK3,PIK3CA,PIK3CB,PIK3CG,SEPTIN3,SEPTIN7
Paxillin Signaling	4.02	ACTN1,PAK3,PIK3CA,PIK3CB,PIK3CG
iCOS-iCOSL Signaling in T Helper Cells	3.96	INPP5D,PIK3CA,PIK3CB,PIK3CG,PPP3CB
HGF Signaling	3.96	HGF,PIK3CA,PIK3CB,PIK3CG,PRKCE
IL-2 Signaling	3.89	PIK3CA,PIK3CB,PIK3CG,STAT5B
fMLP Signaling in Neutrophils	3.87	PIK3CA,PIK3CB,PIK3CG,PPP3CB,PRKCE
Sphingosine-1-phosphate Signaling	3.85	PDGFB,PDGFC,PIK3CA,PIK3CB,PIK3CG
Renin-Angiotensin Signaling	3.84	PAK3,PIK3CA,PIK3CB,PIK3CG,PRKCE
CD40 Signaling	3.78	MAP2K5,PIK3CA,PIK3CB,PIK3CG
ErbB2-ErbB3 Signaling	3.78	PIK3CA,PIK3CB,PIK3CG,STAT5B
CCR3 Signaling in Eosinophils	3.74	PAK3,PIK3CA,PIK3CB,PIK3CG,PRKCE

ErbB4 Signaling	3.73	PIK3CA,PIK3CB,PIK3CG,PRKCE
Natural Killer Cell Signaling	3.72	PAK3,PIK3CA,PIK3CB,PIK3CG,VAV2,VAV3
Role of JAK1 and JAK3 in γc Cytokine Signaling	3.68	PIK3CA,PIK3CB,PIK3CG,STAT5B
Reelin Signaling in Neurons	3.66	ARHGEF9,MAP2K5,PIK3CA,PIK3CB,PIK3CG
Gα12/13 Signaling	3.64	PIK3CA,PIK3CB,PIK3CG,VAV2,VAV3
Role of p14/p19ARF in Tumor Suppression	3.61	PIK3CA,PIK3CB,PIK3CG
Estrogen-Dependent Breast Cancer Signaling	3.56	PIK3CA,PIK3CB,PIK3CG,STAT5B
Th2 Pathway	3.55	PIK3CA,PIK3CB,PIK3CG,RUNX3,STAT5B
Neuroinflammation Signaling Pathway	3.54	GABRA3,GABRB1,GRIA1,PIK3CA,PIK3CB,PIK3CG,PPP3CB
FcγRIIB Signaling in B Lymphocytes	3.54	INPP5D,PIK3CA,PIK3CB,PIK3CG
IL-15 Signaling	3.54	PIK3CA,PIK3CB,PIK3CG,STAT5B
GDNF Family Ligand-Receptor Interactions	3.52	GFRA1,PIK3CA,PIK3CB,PIK3CG
Neurotrophin/TRK Signaling	3.52	MAP2K5,PIK3CA,PIK3CB,PIK3CG
Xenobiotic Metabolism General Signaling Pathway	3.45	MAP2K5,PIK3CA,PIK3CB,PIK3CG,PRKCE
Synaptogenesis Signaling Pathway	3.44	GRIA1,GRIA4,NLGN1,PIK3CA,PIK3CB,PIK3CG,PRKCE
JAK/Stat Signaling	3.43	PIK3CA,PIK3CB,PIK3CG,STAT5B
LPS-stimulated MAPK Signaling	3.39	PIK3CA,PIK3CB,PIK3CG,PRKCE
NF-κB Activation by Viruses	3.39	PIK3CA,PIK3CB,PIK3CG,PRKCE
HER-2 Signaling in Breast Cancer	3.35	PIK3CA,PIK3CB,PIK3CG,PRKCE
IL-4 Signaling	3.33	INPP5D,PIK3CA,PIK3CB,PIK3CG
RANK Signaling in Osteoclasts	3.27	PIK3CA,PIK3CB,PIK3CG,PPP3CB
Docosahexaenoic Acid (DHA) Signaling	3.26	PIK3CA,PIK3CB,PIK3CG
Aldosterone Signaling in Epithelial Cells	3.25	DNAJB5,PIK3CA,PIK3CB,PIK3CG,PRKCE
Gαq Signaling	3.25	PIK3CA,PIK3CB,PIK3CG,PPP3CB,PRKCE
3-phosphoinositide Biosynthesis	3.16	DUSP5,MTMR8,PIK3CA,PIK3CB,PIK3CG
FAK Signaling	3.15	PAK3,PIK3CA,PIK3CB,PIK3CG
CXCR4 Signaling	3.14	PAK3,PIK3CA,PIK3CB,PIK3CG,PRKCE
Germ Cell-Sertoli Cell Junction Signaling	3.1	ACTN1,PAK3,PIK3CA,PIK3CB,PIK3CG
Th1 and Th2 Activation Pathway	3.1	PIK3CA,PIK3CB,PIK3CG,RUNX3,STAT5B
Apelin Cardiomyocyte Signaling Pathway	3.08	PIK3CA,PIK3CB,PIK3CG,PRKCE
Apelin Pancreas Signaling Pathway	3.07	PIK3CA,PIK3CB,PIK3CG
Chronic Myeloid Leukemia Signaling	3.02	PIK3CA,PIK3CB,PIK3CG,STAT5B
PD-1, PD-L1 cancer immunotherapy pathway	2.97	PIK3CA,PIK3CB,PIK3CG,STAT5B
Cardiac Hypertrophy Signaling (Enhanced)	2.97	FGF13,MAP2K5,PIK3CA,PIK3CB,PIK3CG,PPP3CB,PRKCE,WNT8B
Virus Entry via Endocytic Pathways	2.95	PIK3CA,PIK3CB,PIK3CG,PRKCE
Systemic Lupus Erythematosus In B Cell Signaling Pathway	2.95	INPP5D,PIK3CA,PIK3CB,PIK3CG,PPP3CB,PRKCE
Endothelin-1 Signaling	2.91	ECE1,PIK3CA,PIK3CB,PIK3CG,PRKCE
Melanoma Signaling	2.91	PIK3CA,PIK3CB,PIK3CG
Rac Signaling	2.88	PAK3,PIK3CA,PIK3CB,PIK3CG
Molecular Mechanisms of Cancer	2.87	ARHGEF9,PAK3,PIK3CA,PIK3CB,PIK3CG,PRKCE,WNT8B
ERK/MAPK Signaling	2.86	PAK3,PIK3CA,PIK3CB,PIK3CG,PRKCE
Endocannabinoid Developing Neuron Pathway	2.84	MAP2K5,PIK3CA,PIK3CB,PIK3CG
Apelin Endothelial Signaling Pathway	2.84	PIK3CA,PIK3CB,PIK3CG,PRKCE
Lymphotoxin β Receptor Signaling	2.83	PIK3CA,PIK3CB,PIK3CG
Adrenomedullin signaling pathway	2.82	MAP2K5,MYLK3,PIK3CA,PIK3CB,PIK3CG
Role of NANOG in Mammalian Embryonic Stem Cell Pluripotency	2.79	PIK3CA,PIK3CB,PIK3CG,WNT8B
Role of IL-17A in Arthritis	2.79	PIK3CA,PIK3CB,PIK3CG
EGF Signaling	2.79	PIK3CA,PIK3CB,PIK3CG
GP6 Signaling Pathway	2.79	PIK3CA,PIK3CB,PIK3CG,PRKCE
CD28 Signaling in T Helper Cells	2.77	PIK3CA,PIK3CB,PIK3CG,PPP3CB
Th1 Pathway	2.76	PIK3CA,PIK3CB,PIK3CG,RUNX3
CNTF Signaling	2.74	PIK3CA,PIK3CB,PIK3CG
Thrombin Signaling	2.72	ARHGEF9,PIK3CA,PIK3CB,PIK3CG,PRKCE
MSP-RON Signaling Pathway	2.72	PIK3CA,PIK3CB,PIK3CG
Cancer Drug Resistance By Drug Efflux	2.72	PIK3CA,PIK3CB,PIK3CG
IL-6 Signaling	2.71	PIK3CA,PIK3CB,PIK3CG,VEGFA
Phagosome Formation	2.71	PIK3CA,PIK3CB,PIK3CG,PRKCE
PTEN Signaling	2.69	INPP5D,PIK3CA,PIK3CB,PIK3CG
14-3-3-mediated Signaling	2.68	PIK3CA,PIK3CB,PIK3CG,PRKCE
P2Y Purigenic Receptor Signaling Pathway	2.68	PIK3CA,PIK3CB,PIK3CG,PRKCE
Endometrial Cancer Signaling	2.68	PIK3CA,PIK3CB,PIK3CG
AMPK Signaling	2.67	CHRNA3,PIK3CA,PIK3CB,PIK3CG,SMARCD3
Role of NFAT in Cardiac Hypertrophy	2.67	PIK3CA,PIK3CB,PIK3CG,PPP3CB,PRKCE
Synaptic Long Term Potentiation	2.66	GRIA1,GRIA4,PPP3CB,PRKCE
p70S6K Signaling	2.66	PIK3CA,PIK3CB,PIK3CG,PRKCE
IL-12 Signaling and Production in Macrophages	2.62	PIK3CA,PIK3CB,PIK3CG,PRKCE
Role of Osteoblasts, Osteoclasts and Chondrocytes in Rheumatoid Arthritis	2.61	PIK3CA,PIK3CB,PIK3CG,PPP3CB,WNT8B
Role of PI3K/AKT Signaling in the Pathogenesis of Influenza	2.6	PIK3CA,PIK3CB,PIK3CG
1D-myo-inositol Hexakisphosphate Biosynthesis II (Mammalian)	2.59	INPP5D,ITPKB
D-myo-inositol (1,3,4)-trisphosphate Biosynthesis	2.59	INPP5D,ITPKB
IL-17A Signaling in Airway Cells	2.58	PIK3CA,PIK3CB,PIK3CG
Hereditary Breast Cancer Signaling	2.53	PIK3CA,PIK3CB,PIK3CG,SMARCD3
Insulin Receptor Signaling	2.53	INPP5D,PIK3CA,PIK3CB,PIK3CG
Glucocorticoid Receptor Signaling	2.52	PIK3CA,PIK3CB,PIK3CG,PPP3CB,SMARCD3,STAT5B
Type II Diabetes Mellitus Signaling	2.51	PIK3CA,PIK3CB,PIK3CG,PRKCE
SPINK1 General Cancer Pathway	2.5	PIK3CA,PIK3CB,PIK3CG
Huntington's Disease Signaling	2.47	PIK3CA,PIK3CB,PIK3CG,PRKCE,PSME2
Small Cell Lung Cancer Signaling	2.47	PIK3CA,PIK3CB,PIK3CG
Non-Small Cell Lung Cancer Signaling	2.43	PIK3CA,PIK3CB,PIK3CG
Relaxin Signaling	2.42	PIK3CA,PIK3CB,PIK3CG,VEGFA
Leptin Signaling in Obesity	2.42	PIK3CA,PIK3CB,PIK3CG
Glioma Invasiveness Signaling	2.42	PIK3CA,PIK3CB,PIK3CG
Role of Pattern Recognition Receptors in Recognition of Bacteria and Viruses	2.38	PIK3CA,PIK3CB,PIK3CG,PRKCE
Superpathway of D-myo-inositol (1,4,5)-trisphosphate Metabolism	2.37	INPP5D,ITPKB
Sorbitol Degradation I	2.37	SORD

Antiproliferative Role of Somatostatin Receptor 2	2.37	PIK3CA,PIK3CB,PIK3CG
Thyroid Cancer Signaling	2.34	PIK3CA,PIK3CB,PIK3CG
IL-17 Signaling	2.32	PIK3CA,PIK3CB,PIK3CG
HOTAIR Regulatory Pathway	2.32	PIK3CA,PIK3CB,PIK3CG,WNT8B
PEDF Signaling	2.29	PIK3CA,PIK3CB,PIK3CG
HMGB1 Signaling	2.27	MAP2K5,PIK3CA,PIK3CB,PIK3CG
TR/RXR Activation	2.26	PIK3CA,PIK3CB,PIK3CG
G-Protein Coupled Receptor Signaling	2.22	FSHR,PIK3CA,PIK3CB,PIK3CG,PRKCE
Ceramide Signaling	2.21	PIK3CA,PIK3CB,PIK3CG
Senescence Pathway	2.2	MAP2K5,PIK3CA,PIK3CB,PIK3CG,PPP3CB
CTLA4 Signaling in Cytotoxic T Lymphocytes	2.19	PIK3CA,PIK3CB,PIK3CG
Regulation of IL-2 Expression in Activated and Anergic T Lymphocytes	2.19	PPP3CB,VAV2,VAV3
PI3K/AKT Signaling	2.19	INPP5D,PIK3CA,PIK3CB,PIK3CG
T Cell Exhaustion Signaling Pathway	2.19	PIK3CA,PIK3CB,PIK3CG,VEGFA
Prostate Cancer Signaling	2.17	PIK3CA,PIK3CB,PIK3CG
Acute Phase Response Signaling	2.15	PIK3CA,PIK3CB,PIK3CG,TF
Role of NFAT in Regulation of the Immune Response	2.13	PIK3CA,PIK3CB,PIK3CG,PPP3CB
Melanocyte Development and Pigmentation Signaling	2.13	PIK3CA,PIK3CB,PIK3CG
Xenobiotic Metabolism Signaling	2.13	MAP2K5,PIK3CA,PIK3CB,PIK3CG,PRKCE
p53 Signaling	2.08	PIK3CA,PIK3CB,PIK3CG
UVA-Induced MAPK Signaling	2.08	PIK3CA,PIK3CB,PIK3CG
Production of Nitric Oxide and Reactive Oxygen Species in Macrophages	2.08	PIK3CA,PIK3CB,PIK3CG,PRKCE
Alanine Degradation III	2.07	GPT2
Alanine Biosynthesis II	2.07	GPT2
Synaptic Long Term Depression	2.07	GRIA1,GRIA4,GRID2,PRKCE
SAPK/JNK Signaling	2.03	PIK3CA,PIK3CB,PIK3CG
Mouse Embryonic Stem Cell Pluripotency	2.02	PIK3CA,PIK3CB,PIK3CG
PPAR Signaling	2.01	PDGFB,PDGFC,STAT5B
IGF-1 Signaling	2.01	PIK3CA,PIK3CB,PIK3CG
Telomerase Signaling	1.97	PIK3CA,PIK3CB,PIK3CG
Calcium Signaling	1.94	CHRNA3,GRIA1,GRIA4,PPP3CB
NGF Signaling	1.9	PIK3CA,PIK3CB,PIK3CG
D-glucuronate Degradation I	1.9	DCXR
Anandamide Degradation	1.9	FAAH2
Systemic Lupus Erythematosus In T Cell Signaling Pathway	1.87	MAP2K5,PIK3CA,PIK3CB,PIK3CG,PPP3CB
EIF2 Signaling	1.82	PIK3CA,PIK3CB,PIK3CG,VEGFA
RhoA Signaling	1.81	MYLK3,SEPTIN3,SEPTIN7
Systemic Lupus Erythematosus Signaling	1.79	INPP5D,PIK3CA,PIK3CB,PIK3CG
nNOS Signaling in Neurons	1.77	PPP3CB,PRKCE
Endocannabinoid Neuronal Synapse Pathway	1.77	GRIA1,GRIA4,PPP3CB
Cardiac Hypertrophy Signaling	1.72	PIK3CA,PIK3CB,PIK3CG,PPP3CB
STAT3 Pathway	1.71	HGF,PDGFB,VEGFA
Iron homeostasis signaling pathway	1.69	PDGFB,STAT5B,TF
Opioid Signaling Pathway	1.68	MAP2K5,PIK3CG,PPP3CB,PRKCE
Nur77 Signaling in T Lymphocytes	1.59	MAP2K5,PPP3CB
Epithelial Adherens Junction Signaling	1.57	ACTN1,HGF,VAV2
Protein Kinase A Signaling	1.57	DUSP22,DUSP5,MYLK3,PPP3CB,PRKCE
3-phosphoinositide Degradation	1.55	DUSP5,INPP5D,MTMR8
Regulation of eIF4 and p70S6K Signaling	1.54	PIK3CA,PIK3CB,PIK3CG
Pyridoxal 5'-phosphate Salvage Pathway	1.51	PAK3,PRKCE
Calcium-induced T Lymphocyte Apoptosis	1.5	PPP3CB,PRKCE
Remodeling of Epithelial Adherens Junctions	1.47	ACTN1,HGF
Melatonin Signaling	1.43	MAP2K5,PRKCE
Ephrin B Signaling	1.43	VAV2,VAV3
NF-κB Signaling	1.39	PIK3CA,PIK3CB,PIK3CG
Calcium Transport I	1.38	ANXA5
Dendritic Cell Maturation	1.37	PIK3CA,PIK3CB,PIK3CG
Agrin Interactions at Neuromuscular Junction	1.35	PAK3,UTRN
Pregnenolone Biosynthesis	1.27	CYP2E1
Glucocorticoid Biosynthesis	1.27	CYP17A1
GABA Receptor Signaling	1.21	GABRA3,GABRB1
Histidine Degradation VI	1.21	CYP2E1
Neuregulin Signaling	1.2	PRKCE,STAT5B
Salvage Pathways of Pyrimidine Ribonucleotides	1.2	PAK3,PRKCE
Androgen Biosynthesis	1.18	CYP17A1
RAN Signaling	1.16	RANBP2
Ubiquinol-10 Biosynthesis (Eukaryotic)	1.16	CYP2E1
D-myo-inositol (1,4,5)-trisphosphate Degradation	1.16	INPP5D
Sumoylation Pathway	1.15	RANBP2,SENP1
Methylglyoxal Degradation III	1.13	CYP2E1
FAT10 Signaling Pathway	1.13	PSME2
Role of PKR in Interferon Induction and Antiviral Response	1.06	PDGFB,PDGFC
Polyamine Regulation in Colon Cancer	1.05	PSME2
Cholecystokinin/Gastrin-mediated Signaling	1.04	MAP2K5,PRKCE
G Beta Gamma Signaling	1.03	PIK3CG,PRKCE
Phospholipase C Signaling	1.02	ARHGEF9,PPP3CB,PRKCE
IL-22 Signaling	1.01	STAT5B
Atherosclerosis Signaling	1	PDGFB,PDGFC
Role of JAK family kinases in IL-6-type Cytokine Signaling	0.996	STAT5B
Bupropion Degradation	0.996	CYP2E1
Acetone Degradation I (to Methylglyoxal)	0.924	CYP2E1
D-myo-inositol (1,4,5,6)-Tetrakisphosphate Biosynthesis	0.921	DUSP5,MTMR8
D-myo-inositol (3,4,5,6)-tetrakisphosphate Biosynthesis	0.921	DUSP5,MTMR8
Corticotropin Releasing Hormone Signaling	0.903	PRKCE,VEGFA
Factors Promoting Cardiogenesis in Vertebrates	0.876	PRKCE,WNT8B
Inhibition of Angiogenesis by TSP1	0.873	VEGFA
Role of JAK2 in Hormone-like Cytokine Signaling	0.873	STAT5B
D-myo-inositol-5-phosphate Metabolism	0.851	DUSP5,MTMR8
Dopamine-DARPP32 Feedback in cAMP Signaling	0.821	PPP3CB,PRKCE

Cdc42 Signaling	0.804	PAK3,VAV2
Mechanisms of Viral Exit from Host Cells	0.796	PRKCE
nNOS Signaling in Skeletal Muscle Cells	0.796	DMD
Estrogen Biosynthesis	0.796	CYP2E1
GNRH Signaling	0.78	PAK3,PRKCE
Oncostatin M Signaling	0.777	STAT5B
BAG2 Signaling Pathway	0.777	PSME2
RhoGDI Signaling	0.752	ARHGEF9,PAK3
PFKFB4 Signaling Pathway	0.752	MAP2K5
Stearate Biosynthesis I (Animals)	0.742	CYP2E1
Xenobiotic Metabolism CAR Signaling Pathway	0.721	MAP2K5,PRKCE
TNFR1 Signaling	0.719	PAK3
Amyloid Processing	0.719	PRKCE
UVC-Induced MAPK Signaling	0.71	PRKCE
CD27 Signaling in Lymphocytes	0.697	MAP2K5
Phototransduction Pathway	0.697	OPN5
Nicotine Degradation III	0.676	CYP2E1
Semaphorin Signaling in Neurons	0.648	PAK3
Melatonin Degradation I	0.648	CYP2E1
PCP pathway	0.648	WNT8B
Netrin Signaling	0.618	PPP3CB
Nicotine Degradation II	0.618	CYP2E1
Superpathway of Melatonin Degradation	0.618	CYP2E1
Regulation of Cellular Mechanics by Calpain Protease	0.618	ACTN1
Sperm Motility	0.614	MAP2K5,PRKCE
cAMP-mediated signaling	0.6	FSHR,PPP3CB
Basal Cell Carcinoma Signaling	0.58	WNT8B
ERK5 Signaling	0.58	MAP2K5
Hypoxia Signaling in the Cardiovascular System	0.57	VEGFA
TREM1 Signaling	0.564	STAT5B
VDR/RXR Activation	0.55	PRKCE
Role of Wnt/GSK-3β Signaling in the Pathogenesis of Influenza	0.55	WNT8B
Role of BRCA1 in DNA Damage Response	0.541	SMARCD3
Chemokine Signaling	0.541	PIK3CG
Th17 Activation Pathway	0.495	RUNX1
Protein Ubiquitination Pathway	0.493	DNAJB5,PSME2
Regulation of Actin-based Motility by Rho	0.483	PAK3
CCR5 Signaling in Macrophages	0.483	PRKCE
α-Adrenergic Signaling	0.475	PRKCE
TGF-β Signaling	0.475	RUNX3
Apoptosis Signaling	0.465	PRKCE
Gαs Signaling	0.438	FSHR
Antioxidant Action of Vitamin C	0.431	STAT5B
GPCR-Mediated Nutrient Sensing in Enteroendocrine Cells	0.421	PRKCE
Neuroprotective Role of THOP1 in Alzheimer's Disease	0.407	ECE1
LXR/RXR Activation	0.396	TF
IL-15 Production	0.396	MAP2K5
Inhibition of ARE-Mediated mRNA Degradation Pathway	0.394	PSME2
FXR/RXR Activation	0.383	TF
White Adipose Tissue Browning Pathway	0.375	VEGFA
Adipogenesis pathway	0.363	STAT5B
Androgen Signaling	0.358	PRKCE
Necroptosis Signaling Pathway	0.312	PPP3CB
Wnt/β-catenin Signaling	0.283	WNT8B
Hepatic Cholestasis	0.264	PRKCE
Sertoli Cell-Sertoli Cell Junction Signaling	0.264	ACTN1
PPARα/RXRα Activation	0.256	STAT5B
Xenobiotic Metabolism PXR Signaling Pathway	0.253	PRKCE
LPS/IL-1 Mediated Inhibition of RXR Function	0.211	ABCC4

Note:
Statistical significance was calculated by the right-tailed Fisher's Exact Test.

Supplementary Table 4. Molecular Function GO Term Enrichments

GO-ID	p-value	corr p-value	x	n	X	N	Description	Genes in test set	Fly ortho in L intervals
5230	1.32E-08	4.21E-06	8	72	95	15439	extracellular ligand-gated ion channel activity	GRIA1|GLRA1|CHRNA3|GABRB1|GRID2|GABRA3|GRIK2|GRIA4	
70851	3.74E-08	5.96E-06	8	82	95	15439	growth factor receptor binding	VAV3|HGF|PDGFC|PDGFB|VEGFB|VEGFC|VAV2|VEGFA	
5544	2.44E-07	2.60E-05	5	23	95	15439	calcium-dependent phospholipid binding	ANXA8L1|ANXA5|ANXA11|ANXA7|ANXA8	
5231	4.05E-07	3.23E-05	6	47	95	15439	excitatory extracellular ligand-gated ion channel activity	GRIA1|GLRA1|CHRNA3|GRID2|GRIK2|GRIA4	
22834	9.94E-07	5.29E-05	8	125	95	15439	ligand-gated channel activity	GRIA1|GLRA1|CHRNA3|GABRB1|GRID2|GABRA3|GRIK2|GRIA4	
15276	9.94E-07	5.29E-05	8	125	95	15439	ligand-gated ion channel activity	GRIA1|GLRA1|CHRNA3|GABRB1|GRID2|GABRA3|GRIK2|GRIA4	
46934	2.24E-06	9.23E-05	3	5	95	15439	phosphatidylinositol-4,5-bisphosphate 3-kinase activity	PIK3CA|PIK3CB|PIK3CG	Pi3K92E
4970	2.31E-06	9.23E-05	4	16	95	15439	ionotropic glutamate receptor activity	GRIA1|GRID2|GRIK2|GRIA4	GluRIIE, CG18039, Ir92a
5234	3.85E-06	1.37E-04	4	18	95	15439	extracellular-glutamate-gated ion channel activity	GRIA1|GRID2|GRIK2|GRIA4	GluRIIE, CG18039, Ir92a
5172	4.45E-06	1.42E-04	3	6	95	15439	vascular endothelial growth factor receptor binding	VEGFB|VEGFC|VEGFA	Pvf1
43498	4.99E-06	1.45E-04	5	41	95	15439	cell surface binding	TF|PDGFC|ANXA5|PDGFB|VEGFA	
5089	5.65E-06	1.48E-04	6	73	95	15439	Rho guanyl-nucleotide exchange factor activity	VAV3|PREX1|ARHGEF9|HGF|PAK3|VAV2	
8083	6.04E-06	1.48E-04	8	159	95	15439	growth factor activity	HGF|PDGFC|PDGFB|VEGFB|VEGFC|FGF13|PGF|VEGFA	CG7432, Pvf1, bnl
5102	1.10E-05	2.50E-04	18	921	95	15439	receptor binding	VAV3|STAT5B|STOML2|SMARCD3|ACTN1|HGF|ANXA5|PDGFB|VEGFB |VEGFC|GFRA1|PGF|LRP1B|VAV2|VEGFA|DNAJA3|PDGFC|FGF13	
5088	1.56E-05	3.33E-04	6	87	95	15439	Ras guanyl-nucleotide exchange factor activity	VAV3|PREX1|ARHGEF9|HGF|PAK3|VAV2	
8066	2.12E-05	4.22E-04	4	27	95	15439	glutamate receptor activity	GRIA1|GRID2|GRIK2|GRIA4	GluRIIE, CG18039, Ir92a
16303	2.63E-05	4.93E-04	3	10	95	15439	1-phosphatidylinositol-3-kinase activity	PIK3CA|PIK3CB|PIK3CG	Pi3K92E
8509	2.96E-05	5.24E-04	7	144	95	15439	anion transmembrane transporter activity	ABCC4|GLRA1|GABRB1|GABRA3|SLC17A1|SLC17A3|SLC17A4	
35004	3.59E-05	5.98E-04	3	11	95	15439	phosphoinositide 3-kinase activity	PIK3CA|PIK3CB|PIK3CG	Pi3K92E
43183	3.75E-05	5.98E-04	2	2	95	15439	vascular endothelial growth factor receptor 1 binding	VEGFB|VEGFA	Pvf1
5161	4.77E-05	7.25E-04	3	12	95	15439	platelet-derived growth factor receptor binding	PDGFC|PDGFB|VEGFA	Pvf1
15114	6.17E-05	8.95E-04	3	13	95	15439	phosphate transmembrane transporter activity	SLC17A1|SLC17A3|SLC17A4	
4428	6.81E-05	9.45E-04	4	36	95	15439	inositol or phosphatidylinositol kinase activity	ITPKB|PIK3CA|PIK3CB|PIK3CG	Pi3K92E, CG15745, IP3K2
5083	7.36E-05	9.78E-04	9	290	95	15439	small GTPase regulator activity	VAV3|PREX1|ARHGEF9|HGF|DNAJA3|ERC1|ARHGAP15|PAK3|VAV2	
42056	9.73E-05	1.24E-03	3	15	95	15439	chemoattractant activity	VEGFB|VEGFC|VEGFA	
15075	1.07E-04	1.24E-03	14	710	95	15439	ion transmembrane transporter activity	GRIA1|ABCC4|CHRNA3|GABRB1|GRID2|GABRA3|ATP11C|GRIK2|GLRA1|SCN 2A|SLC17A1|SLC17A3|GRIA4|SLC17A4	
22836	1.08E-04	1.24E-03	9	305	95	15439	gated channel activity	GRIA1|GLRA1|CHRNA3|GABRB1|GRID2|GABRA3|GRIK2|SCN2A|GRIA4	
5216	1.09E-04	1.24E-03	10	378	95	15439	ion channel activity	GRIA1|ABCC4|GLRA1|CHRNA3|GABRB1|GRID2|GABRA3|GRIK2|SCN2A |GRIA4	
22838	1.35E-04	1.49E-03	10	388	95	15439	substrate-specific channel activity	GRIA1|ABCC4|GLRA1|CHRNA3|GABRB1|GRID2|GABRA3|GRIK2|SCN2A |GRIA4	
5543	1.54E-04	1.64E-03	7	187	95	15439	phospholipid binding	PREX1|TF|ANXA8L1|ANXA5|ANXA11|ANXA7|ANXA8	
15267	1.84E-04	1.87E-03	10	403	95	15439	channel activity	GRIA1|ABCC4|GLRA1|CHRNA3|GABRB1|GRID2|GABRA3|GRIK2|SCN2A|GRIA 4	
22803	1.88E-04	1.87E-03	10	404	95	15439	passive transmembrane transporter activity	GRIA1|ABCC4|GLRA1|CHRNA3|GABRB1|GRID2|GABRA3|GRIK2|SCN2A |GRIA4	
5085	2.81E-04	2.72E-03	6	146	95	15439	guanyl-nucleotide exchange factor activity	VAV3|PREX1|ARHGEF9|HGF|PAK3|VAV2	
22857	3.54E-04	3.32E-03	15	895	95	15439	transmembrane transporter activity	GRIA1|ABCC4|CHRNA3|GABRB1|GRID2|GABRA3|ATP11C|GRIK2|ABCC12|GL RA1|SCN2A|SLC17A1|SLC17A3|GRIA4|SLC17A4	
22891	4.55E-04	4.14E-03	14	817	95	15439	substrate-specific transmembrane transporter activity	GRIA1|ABCC4|CHRNA3|GABRB1|GRID2|GABRA3|ATP11C|GRIK2|GLRA1|SCN 2A|SLC17A1|SLC17A3|GRIA4|SLC17A4	
15277	5.53E-04	4.90E-03	2	6	95	15439	kainate selective glutamate receptor activity	GRIA1|GRIK2	GluRIIE, CG18039
1727	6.61E-04	5.70E-03	3	28	95	15439	lipid kinase activity	PIK3CA|PIK3CB|PIK3CG	Pi3K92E
5100	7.34E-04	6.16E-03	3	29	95	15439	Rho GTPase activator activity	PREX1|HGF|ARHGAP15	
5254	8.11E-04	6.63E-03	4	68	95	15439	chloride channel activity	ABCC4|GLRA1|GABRB1|GABRA3	
5436	1.02E-03	8.12E-03	2	8	95	15439	sodium:phosphate symporter activity	SLC17A3|SLC17A4	
30695	1.04E-03	8.12E-03	9	416	95	15439	GTPase regulator activity	VAV3|PREX1|ARHGEF9|HGF|DNAJA3|ERC1|ARHGAP15|PAK3|VAV2	

5253	1.17E-03	8.89E-03	4	75	95	15439	anion channel activity	ABCC4|GLRA1|GABRB1|GABRA3	
60589	1.21E-03	8.99E-03	9	425	95	15439	nucleoside-triphosphatase regulator activity	VAV3|PREX1|ARHGEF9|HGF|DNAJA3|ERC1|ARHGAP15|PAK3|VAV2	
15103	1.39E-03	1.01E-02	3	36	95	15439	inorganic anion transmembrane transporter activity	SLC17A1|SLC17A3|SLC17A4	
5509	1.58E-03	1.12E-02	11	626	95	15439	calcium ion binding	STAT5B|ANXA8L1|ACTN1|ANXA5|ANXA11|ANXA7|ANXA8|DMD|UTRN|LRP1B|RUNX1	
1968	1.99E-03	1.38E-02	2	11	95	15439	fibronectin binding	C6ORF15|VEGFA	
22892	2.05E-03	1.39E-02	14	955	95	15439	substrate-specific transporter activity	GRIA1|ABCC4|CHRNA3|GABRB1|GRID2|GABRA3|ATP11C|GRIK2|GLRA1|SCN2A|SLC17A1|SLC17A3|GRIA4|SLC17A4	
46982	2.11E-03	1.40E-02	6	215	95	15439	protein heterodimerization activity	HGF|PDGFB|VEGFB|PGF|RUNX1|VEGFA	
42803	2.25E-03	1.46E-02	8	377	95	15439	protein homodimerization activity	PDGFC|PDGFB|VEGFB|ECE1|GRIK2|PGF|RUNX1|VEGFA	
30234	2.29E-03	1.46E-02	13	860	95	15439	enzyme regulator activity	VAV3|PRKCE|HGF|ANXA5|MTMR8|ARHGAP15|VAV2|PREX1|ARHGEF9|DNAJA3|ERC1|FGF13|PAK3	
43560	2.38E-03	1.49E-02	2	12	95	15439	insulin receptor substrate binding	PIK3CA|PIK3CB	
8138	2.66E-03	1.63E-02	3	45	95	15439	protein tyrosine/serine/threonine phosphatase activity	DUSP22|DUSP5|SSH2	
5154	2.80E-03	1.68E-02	2	13	95	15439	epidermal growth factor receptor binding	VAV3|VAV2	
4725	3.61E-03	2.13E-02	4	102	95	15439	protein tyrosine phosphatase activity	DUSP22|DUSP5|MTMR8|SSH2	
166	4.29E-03	2.49E-02	24	2255	95	15439	nucleotide binding	ABCC4|PRKCE|ULK4|DAZ4|SORD|ATP11C|DAZ3|DAZ2|DAZ1|PIK3CB|ABCA13|RUNX3|MYLK3|PIK3CG|ABCC12|RUNX1|SEPT3|ITPKB|RALYL|SEPT7|PIK3CA|DNAJA3|PAK3|MAP2K5	
5215	4.63E-03	2.64E-02	15	1159	95	15439	transporter activity	GRIA1|ABCC4|CHRNA3|GABRB1|GRID2|GABRA3|ATP11C|GRIK2|ABCC12|GLRA1|SCN2A|SLC17A1|SLC17A3|GRIA4|SLC17A4	
8201	4.88E-03	2.73E-02	4	111	95	15439	heparin binding	C6ORF15|VEGFB|PGF|VEGFA	
5126	5.82E-03	3.07E-02	5	186	95	15439	cytokine receptor binding	HGF|DNAJA3|VEGFB|VEGFC|VEGFA	CG7432, Pvf1, CG32640, CG32641
4890	5.98E-03	3.07E-02	2	19	95	15439	GABA-A receptor activity	GABRB1|GABRA3	
30487	6.15E-03	3.07E-02	1	1	95	15439	inositol-4,5-bisphosphate 5-phosphatase activity	INPP5D	
3939	6.15E-03	3.07E-02	1	1	95	15439	L-iditol 2-dehydrogenase activity	SORD	
4963	6.15E-03	3.07E-02	1	1	95	15439	follicle-stimulating hormone receptor activity	FSHR	
50038	6.15E-03	3.07E-02	1	1	95	15439	L-xylulose reductase (NADP+) activity	DCXR	
4508	6.15E-03	3.07E-02	1	1	95	15439	steroid 17-alpha-monooxygenase activity	CYP17A1	
16917	7.99E-03	3.92E-02	2	22	95	15439	GABA receptor activity	GABRB1|GABRA3	
46983	9.16E-03	4.43E-02	9	578	95	15439	protein dimerization activity	HGF|PDGFC|PDGFB|VEGFB|ECE1|GRIK2|PGF|RUNX1|VEGFA	

Notes:
File created with bingo (c) on May 20, 2020 at 7:35:34 PM
ontology: namespace
curator: bingo
Selected ontology file : bundle://125.0:1/data/GO_Molecular_Function
Selected annotation file : bundle://125.0:1/data/H_sapiens_default
Discarded evidence codes :

For statistical significance of overrepresentation:
Selected statistical test : Hypergeometric test
Selected correction : Benjamini & Hochberg False Discovery Rate (FDR) correction
Selected significance level : 0.05
Testing option : Use whole annotation as reference set
