# Supplementary material for: Multiple mechanisms drive genomic adaptation to extreme O2 levels in Drosophila melanogaster
Source: Nat Commun. 2021 Feb 12;12:997. doi: 10.1038/s41467-021-21281-6 (PMC7881140; doi:10.1038/s41467-021-21281-6)
Supplement: Supplementary file 3 — Reporting Summary [file 41467_2021_21281_MOESM3_ESM.pdf]

nature research

Corresponding author(s): Gabriel G. Haddad, Dan Zhou, Vineet Bafna

Last updated by author(s): 2020/12/18

Reporting Summary

Nature Research wishes to improve the reproducibility of the work that we publish. This form provides structure for consistency and transparency in reporting. For further information on Nature Research policies, see our Editorial Policies and the Editorial Policy Checklist.

Statistics

For all statistical analyses, confirm that the following items are present in the figure legend, table legend, main text, or Methods section.

n/a	Confirmed	
x		The exact sample size (n) for each experimental group/condition, given as a discrete number and unit of measurement
	x	A statement on whether measurements were taken from distinct samples or whether the same sample was measured repeatedly
	x	The statistical test(s) used AND whether they are one- or two-sided *Only common tests should be described solely by name; describe more complex techniques in the Methods section.*
x		A description of all covariates tested
	x	A description of any assumptions or corrections, such as tests of normality and adjustment for multiple comparisons
	x	A full description of the statistical parameters including central tendency (e.g. means) or other basic estimates (e.g. regression coefficient) AND variation (e.g. standard deviation) or associated estimates of uncertainty (e.g. confidence intervals)
	x	For null hypothesis testing, the test statistic (e.g. F, t, r) with confidence intervals, effect sizes, degrees of freedom and P value noted *Give P values as exact values whenever suitable.*
x		For Bayesian analysis, information on the choice of priors and Markov chain Monte Carlo settings
x		For hierarchical and complex designs, identification of the appropriate level for tests and full reporting of outcomes
	x	Estimates of effect sizes (e.g. Cohen's d, Pearson's r), indicating how they were calculated

Our web collection on statistics for biologists contains articles on many of the points above.

Software and code

Policy information about availability of computer code

Data collection	BWA-MEM software version 0.7.8 was used to align the reads to the reference genome. GATK 3.7 was used for variant calling on pooled sequence reads from evolving fly populations. Picard tools 2.9.0 with CollectVariantCallingMetrics option was used to extract metrics summary. BiNGO (Biological Network Gene Ontology) version 3.0.3 plugin on Cytoscape 3.8.0 was used to determine statistic significance of the enrichment in Gene Ontology (GO) categories.
Data analysis	Custom code is publicly available at https://github.com/airanmehr/ESAP.

For manuscripts utilizing custom algorithms or software that are central to the research but not yet described in published literature, software must be made available to editors and reviewers. We strongly encourage code deposition in a community repository (e.g. GitHub). See the Nature Research guidelines for submitting code & software for further information.

Data

Policy information about availability of data

All manuscripts must include a data availability statement. This statement should provide the following information, where applicable:

- Accession codes, unique identifiers, or web links for publicly available datasets
- A list of figures that have associated raw data
- A description of any restrictions on data availability

Whole genome sequence data of n=59 pooled samples are available at https://trace.ncbi.nlm.nih.gov/Traces/study/?acc=PRJNA657615&o=acc_s%3Aa. Source data are provided with this paper.

Field-specific reporting

Please select the one below that is the best fit for your research. If you are not sure, read the appropriate sections before making your selection.

☒ Life sciences ☐ Behavioural & social sciences ☐ Ecological, evolutionary & environmental sciences

For a reference copy of the document with all sections, see nature.com/documents/nr-reporting-summary-flat.pdf

Life sciences study design

All studies must disclose on these points even when the disclosure is negative.

Sample size	A Wright-Fisher Hidden Markov chain model was used to compute the maximum likelihood estimate of the population size. For the functional assays, the sample size, number of replicates, error bars and statistical tests were chosen based on accepted practices in the field and stated in each figure legend.
Data exclusions	Sequencing reads containing excess of N's (>10%; N represents bases that could not be determined) and the number of low quality (Qscore ≤ 5) over 50% of the total length (reads with poor sequencing quality) were removed. The variants within each sample with coverage outside the (0.5%, 99.5%) range of genomewide coverage and the spurious variants that oscillated between frequency of 0 and 1 were removed. We identified them by calculating absolute sum of increments for each base and filtered those that had absolute increments ≥3 (0.99975th quantile).
Replication	Analysis was done with 3 experimental replicates per condition for the laboratory evolution populations. Each follow-up functional analysis was performed in three biological replicates. The data are reported as standard deviations of the biological replicates. Details on replication of each particular experiment are provided in Figure Legends and method section.
Randomization	For the laboratory evolution experiment, the embryos collected from parental population were randomly devided into room air (control), hypoxia and hyperoxia groups. Randomly picked 100 male and 100 female adult flies from each population were pooled and used for whole genome sequencing.
Blinding	Blinding assessment was performed when applicable, including sequencing.

Reporting for specific materials, systems and methods

We require information from authors about some types of materials, experimental systems and methods used in many studies. Here, indicate whether each material, system or method listed is relevant to your study. If you are not sure if a list item applies to your research, read the appropriate section before selecting a response.

Materials & experimental systems

n/a	Involved in the study	
☒	☐	Antibodies
☒	☐	Eukaryotic cell lines
☒	☐	Palaeontology and archaeology
☐	☒	Animals and other organisms
☒	☐	Human research participants
☒	☐	Clinical data
☒	☐	Dual use research of concern

Methods

n/a	Involved in the study	
☒	☐	ChIP-seq
☒	☐	Flow cytometry
☒	☐	MRI-based neuroimaging

Animals and other organisms

Policy information about studies involving animals; ARRIVE guidelines recommended for reporting animal research

Laboratory animals	Drosophila melanogaster (fruit flies, male and female) from embryo to adult was used for the evolution experiment and functional assays. Adult flies were used for whole genome sequencing.
Wild animals	No wild animals were used in the study.
Field-collected samples	No field collected samples were used in the study.
Ethics oversight	Ethic oversight was not required for studies using Drosophila melanogaster (fruit fly).

Note that full information on the approval of the study protocol must also be provided in the manuscript.
