# Supplementary material for: Multiple mechanisms drive genomic adaptation to extreme O2 levels in Drosophila melanogaster
Source: Nat Commun. 2021 Feb 12;12:997. doi: 10.1038/s41467-021-21281-6 (PMC7881140; doi:10.1038/s41467-021-21281-6)
Supplement: Supplementary file 2 — Peer Review [file 41467_2021_21281_MOESM2_ESM.pdf]

Reviewer #1 (Remarks to the Author):

Obtaining insights in how species adapt to different environments is a key goal of evolutionary biology. In this manuscript, the authors utilized time-sampled Drosophila melanogaster whole genome data to investigate how populations adapt to hypoxic and hyperoxic environments.

There are several major issues that will have to be addressed before publication:

Correctly identifying signals of positive selection from population genetic data remains challenging because demographic events can generate similar patterns in genomic datasets. As the authors note, their populations experienced strong "bottlenecks with severe reduction(s) of population size (...) with every change in O2 level." Their applied method (CLEAR), however, was developed for "fixed-sized populations" and is thus (unless adjusted) not suited for the investigation in question. Given the small effective population sizes (<200) combined with strong bottlenecks, many polymorphisms are indeed expected to fix simply by chance. In addition, purifying selection, known to act broadly across the genome, and the effects of background selection were completely ignored and will need to be taken into account.

The dataset itself requires additional attention as well, as it does not follow the current best practises of the field. For example, no quality control was carried out on the raw data to remove adapters, contamination, and PCR duplications or trim low quality bases – all of which can to lead to spurious variant calls – and, no filters (other than coverage) were applied to the variant call set to mitigate false positives. I would encourage the authors to have a look at GATK's Best Practise recommendations (Van der Auwera et al. 2013) to ensure that they are basing their analyses on a high-quality dataset. Unfortunately, the data itself has not been made available to the reviewers, hindering a more careful assessment of the claims made in the manuscript (i.e., the manuscript states that "Data are available in the supplementary materials" but the supplement merely contains a summary table). For their work to be reproducible, the raw sequence reads should be deposited in NCBI's Sequence Read Archive (SRA) and the variants in a public archive (Dryad, ENA, or similar).

Equally concerning is the inappropriate self-citing of the authors throughout the manuscript (which, with more than 10 of the 30 cited articles, is rather appalling). The authors appear largely unaware of the relevant literature, leading to i) outrageous claims (e.g., "We made the first direct observation of beneficial alleles recombining to form a highly beneficial haplotype.") as well as ii) a deeply rooted misunderstanding of previous population genetic work (e.g., "this result provides one of the only confirmed observations of a recombination event overcoming the Fisher-Muller ratchet" when actually referring to the Hill-Robertson effect). Consequently, several of the (limited) external citations provided are inappropriate (e.g., the observation that "multiple favored variants can also be acquired on a single haplotype via recombination that prevents clonal interference and accelerates adaptation" has been made by Hill and Robertson in 1966 not by the Desai lab in 2016).

Once these issues have been addressed, it would be interesting to place the work in a broader context. For example, how do their findings fit in with previous work (e.g., Van Voorhies (2009) research investigating the "metabolic function in Drosophila melanogaster in response to hypoxia and pure oxygen")?

Lastly, as a side note, the title "A decade on Everest" is misleading – neither have the experiments been carried out at Everest nor have the populations been sampled there.

Reviewer #2 (Remarks to the Author):

Iranmehr et al report an analysis of an amazing evolution experiment: 9 populations propagated in

atmosphere controlled environments for 15 years. There are three treatments 3x reps evolving with low O2, 3x reps under high O2 and 3x control populations under normal O2. They carry out whole genome sequencing of many individuals from each of the nine populations at multiple time points. They have found strong evidence for both hard and soft sweeps in parallel populations in early generations. They have also found the late arrival of favourable de novo sweeps in parallel populations. Finally, the authors report the first direct observation of a recombination event that brings beneficial alleles together in a single haplotype in a multicellular organism. Furthermore, the authors have developed clever statistical methods to examine genomic data collected at generational time scales.

I think that this is a neat experimental study carried out on a large scale, and presents novel findings that will be of interest to all evolutionary biologists. In saying this, I found it difficult to follow in some places and interpreting some of the figures in order to assess the authors claims was challenging.

1. The title and claims need to be revised to suit the experiments that were carried out. I don't mind a clever title, but it is currently misleading- the title suggests a study of natural populations adapting to high altitudes and low oxygen (on Mt Everest), where this was a lab study carried out for 15 years in high and low oxygen concentrations. Also, the authors claim to be the first to observe beneficial alleles recombining into a new haplotype, which has already been done for a diploid sexual organism PMID: 32042122. You will need to refine your claims to better suit what you have done, ie this study is probably the the first to do this in Drosophilia or perhaps any metazoan system.

2.The authors give two reasons for late sweeps- that either recombination or de novo mutations create new haplotypes. However, since the evolution experiment including incremental changes in oxygen concentration it is possible that an extant allele could only be selected once a certain threshold oxygen concentration is crossed during the experiment. This should also be included as a possibility.

3. The figure cited (figure s6) is difficult to reconcile with the claims about the H sweeps. In Fig s6 it is not clear to me that sweeps have happened in parallel, for example HD and HA are only evident in two of the panels. Also, these figures are difficult to interpret- Fig s5 and s6 do not have a label on the y axis and figure legend is only one sentence- please explain the axes and expand the legend. In contrast, in figures s7-s15 the allele frequency time course panels show parallel and synchronicity very clearly, but are not cited as demonstrating parallelism, perhaps it would be good to point the reader to these figures to make the point about sweeps, as well as synchronicity.

Minor points

Main text first paragraph- standing genetic variation is not necessarily drifting, but could be under weak selection or maintained by balancing selection – better to just remove this.

Page 3: "…similar number of embryos…" What were the actual numbers?

Page 4: Why did the authors choose to sequence L-fly, H-fly and N-fly populations at different generations?

Page 5: "…PCA was performed using only extant SNPs…" Please include this information in Figure 1D caption. Have the authors also performed PCA analysis using the whole genetic variation including de novo SNPs? If so do the de novo SNPs change the results?.

Page 9: "Additionally, knocking down cic gene…" How was this gene deletion done? Please include a section in the methods that points to a reference and briefly describes how it was done.

Page 9: "…bnl… …with its expression upregulated > 2-fold in flies when exposed to 5% O2" How did the authors measure this finding? RNAseq? Once again, please include a section explaining this in the

methods.

Page 10: The first two sentences of the discussion, about medical cell preservation techniques, which seems a non-sequitor, I would recommend deleting this. Also “accidental origin” Do you mean non-selected or neural? Please rewrite.
Page 19: Fig 2E, please indicate what the blue and light brown colors refer to. Also, Figure 2 legend: fixed, not fixated

Pages 25-26: “Embryos from this parental population were collected as F1 and subjected to experimental evolution in low or high oxygen environments (oxygen-directed evolutions, 3 populations per condition), or under room air (control experiment for genetic drift, 3 populations)” How many of these F1 embryos were used as parental populations? What were the starting population sizes? Did they vary in size?

Page 26: “Embryos, 3rd instar larvae and adult flies were collected from each generation and stored at 80C for subsequent analyses” How many embryos, 3rd instar larvae and adult flies were frozen at -80 degree freezer? Also change “80C” to -80°C.

Page 65-page 880 of the document I reviewed was a pdf list of genes. In the final version this should be supplied as a spreadsheet supplementary data file since it is not convenient to review or use in its current form.

Reviewer #3 (Remarks to the Author):

This is without doubt an excellent manuscript that I believe can be published as it is (I have very minor comments). The authors have managed to correctly apply complex statistical analyses for the identification of selective sweeps to their experimental system. I am very pleased by the attention the authors have dedicated to disentangling the effects of genetic drift and positive selection. I would like to congratulate the authors for this exciting study.

Minor comments

1 - It would be nice to describe the geographic origin of the 27 lines that were used to found the 9 experimental populations. Were they chosen to maximise genetic diversity? Or to represent extreme natural environments with respect to altitude?

2- the genomic distribution of the main signals of selection seem to indicate a significant effect of the X chromosome. Could this be tested? The X chromosome has the particularity to be hemizygous in males in this species which could favor the fixation of recessive beneficial mutations?

Figure 1D: show percentage of explained variance

Figure S2: what is the dominance coefficient assumed for the selected alleles in Figure S2C?

Figure S3 not clear enough. What is the unit of the y-axis of Figure S3B, is this the test statistic of CLEAR?

Figure 2C: a legend is needed for the color-coding

REVIEWER COMMENTS

Reviewer #1 (Remarks to the Author):

Obtaining insights in how species adapt to different environments is a key goal of evolutionary biology. In this manuscript, the authors utilized time-sampled Drosophila melanogaster whole genome data to investigate how populations adapt to hypoxic and hyperoxic environments.

There are several major issues that will have to be addressed before publication:

Correctly identifying signals of positive selection from population genetic data remains challenging because demographic events can generate similar patterns in genomic datasets. As the authors note, their populations experienced strong "bottlenecks with severe reduction(s) of population size (...) with every change in O2 level." Their applied method (CLEAR), however, was developed for "fixed-sized populations" and is thus (unless adjusted) not suited for the investigation in question. Given the small effective population sizes (<200) combined with strong bottlenecks, many polymorphisms are indeed expected to fix simply by chance.

Response. The reviewer makes a good point. Drift and demographic changes can confound tests of selection. To reduce false-positives due to drift, we had already limited our analysis to relatively short time intervals that were 120 generations, shorter than the 180 generations (see Supplementary Fig. 4). However, to address the concern of this reviewer directly, we have now *applied CLEAR with varying population sizes* (Supplementary Methods: “Adjusting for variable population size”. Briefly, CLEAR first estimates the population size change between sampling (already in Fig. 1c) and uses these estimates to detect selective sweep trajectories. As shown in Supplementary Figure 8, CLEAR statistics remain concordant, even though adjusting population size scales-down the CLEAR statistic. However, since we already had used a stringent cutoff (7 standard deviation from genome-wide average), **all** the significant results in the fixed-population size regime remained significant in the adjusted-population-size regime (Supplementary Fig. 8 and 35).

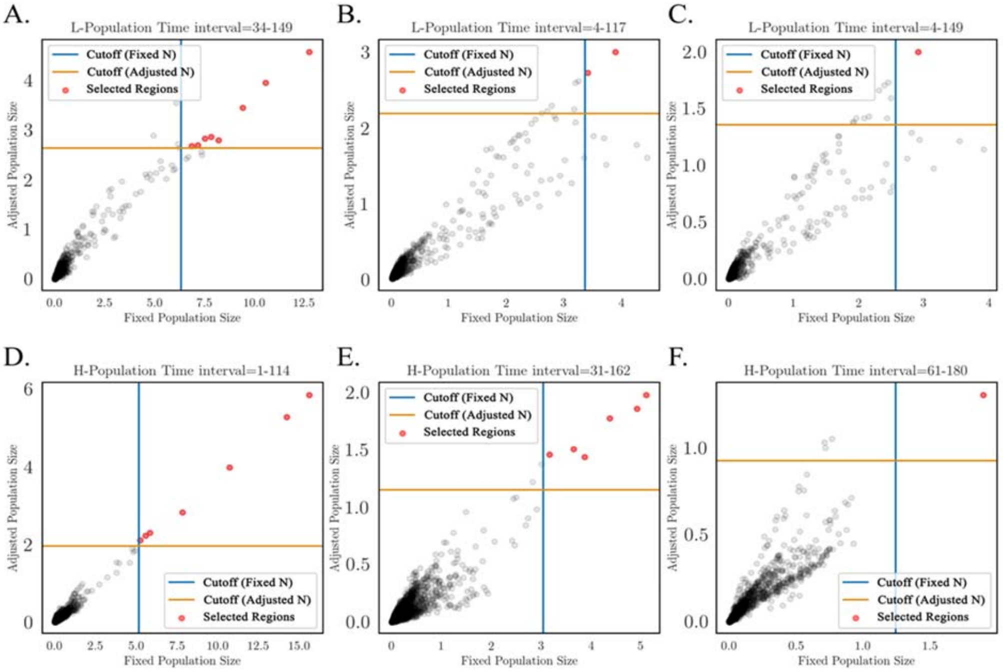

Supplementary Fig 8. Scatter plot depicting absolute CLEAR statistics for fixed vs adjusted population size at different time intervals. (See supplementary figure for detailed caption)

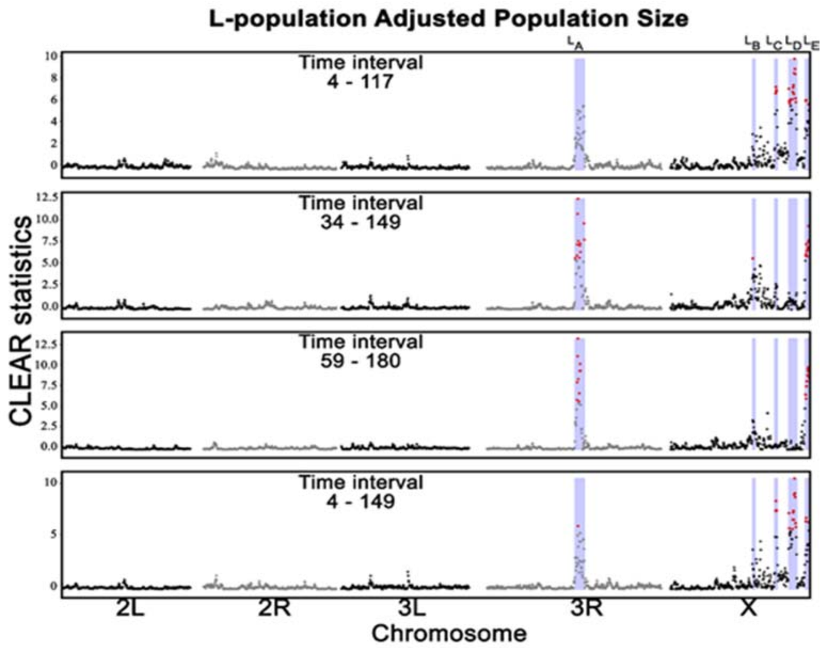

Supplementary Fig. 35. L-Population Replicated Sweeps with adjusted population size. (See supplementary figure for detailed caption)

In addition, purifying selection, known to act broadly across the genome, and the effects of background selection were completely ignored and will need to be taken into account.

Response. We agree that background selection could lead to signals that are not related to extreme oxygen tolerance. One way to distinguish positive selection from background selection is to compare evolution in L and H populations to that in the control N populations. Although we do not have time-series data for the control (N) populations, in our 2011 paper (Zhou, PNAS https://www.ncbi.nlm.nih.gov/pmc/articles/PMC3038716/), we had looked at generation 180 in both normal (N-population) and hypoxia (L-population), and performed a cross population test comparing to controls. We found a strongly replicated signal found in L populations but not in N. The same signals are found in our current study for low oxygen (L populations), which suggests that our signals are not part of background selection.

To address the reviewer's concern further, we picked every region identified in this study as being under selection from L and H populations and we looked at the allele frequency spectrum in generation 180 between the L or H population, and control (N). The results of this analysis are shown in Supplementary Fig. 9 and discussed on page 9, paragraph 1 in the manuscript. We observe a large and significant excess of high-frequency alleles in L and H populations in putatively selected regions and essentially an absence of variants at intermediate frequencies, compared to N populations.

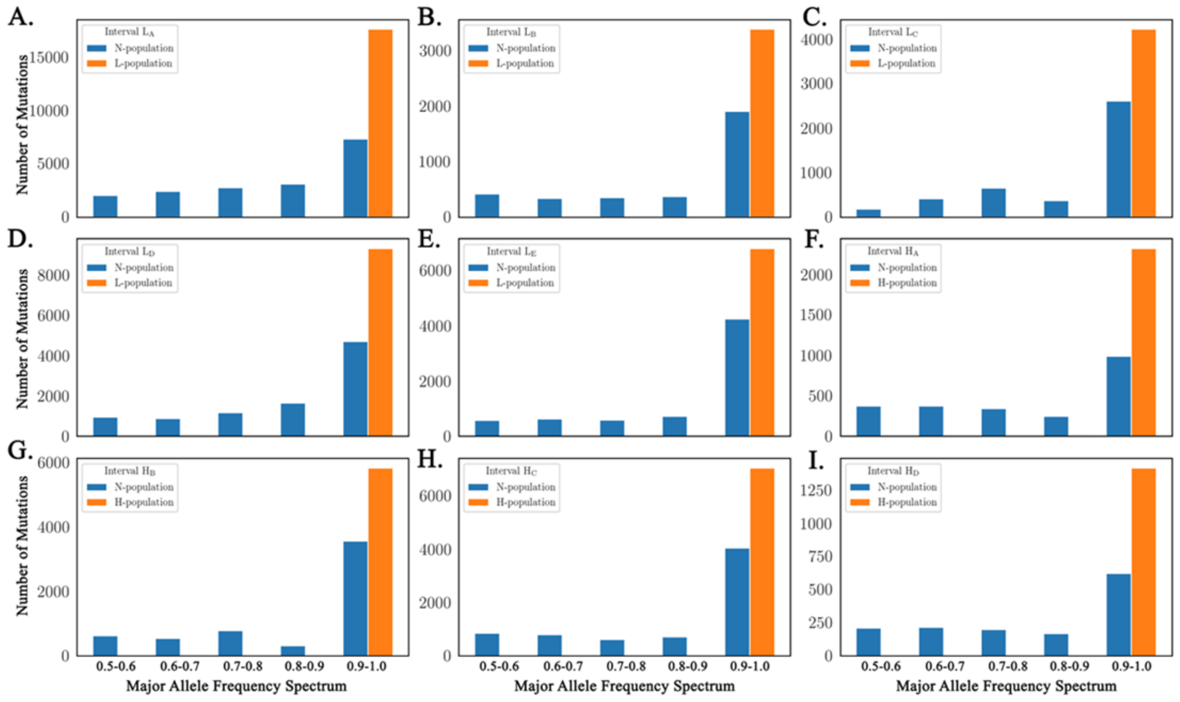

Supplementary Fig 9. Allele frequency spectra (AFS) at generation 180 in every selected region of the L and H populations and the corresponding AFS in the N population at the same locus. (See supplementary figure for detailed caption)

We conclude that background selection is unlikely to have caused fixation at all of the loci that we identified as selected. We thank the reviewer for reminding us of this issue, and we have added the following note in the manuscript.

"Before analyzing these results further, we tested for any confounding factors that could result in a false signal. First, we determined if the use of fixed population size with CLEAR was appropriate for scenarios with varying and small population sizes. To check, we used a version of CLEAR that uses estimated population sizes (Supplementary Methods), and found complete concordance between CLEAR signals with fixed population and adjusted population sizes (Supplementary Fig. 8). Next, we investigated whether background selection as purifying selection can distort the allele frequency spectrum (AFS). Of note, the number of SNPs with *i* mutant alleles is inversely proportional to *i* for neutrally evolving populations[24], suggesting hyperbolically decreasing intermediate allele frequencies, which disappear or diminish under positive selection. Therefore, we investigated the AFS at generation 180 in every selected

region of the L and H populations and compared it to the AFS in the N population at the same locus. Expectedly, we observed intermediate frequency alleles in the N-populations but their complete absence in the corresponding H- and L- populations in every selected region (Supplementary Fig. 9), confirming that the signal in L and H populations was not due to background selection."

The dataset itself requires additional attention as well, as it does not follow the current best practises of the field. For example, no quality control was carried out on the raw data to remove adapters, contamination, and PCR duplications or trim low quality bases – all of which can to lead to spurious variant calls – and, no filters (other than coverage) were applied to the variant call set to mitigate false positives. I would encourage the authors to have a look at GATK's Best Practise recommendations (Van der Auwera et al. 2013) to ensure that they are basing their analyses on a high-quality dataset. Unfortunately, the data itself has not been made available to the reviewers, hindering a more careful assessment of the claims made in the manuscript (i.e., the manuscript states that "Data are available in the supplementary materials" but the supplement merely contains a summary table). For their work to be reproducible, the raw sequence reads should be deposited in NCBI's Sequence Read Archive (SRA) and the variants in a public archive (Dryad, ENA, or similar).

Response. The reviewer is right in that we did omit some details in the supplement, and these have been added now in full detail. A brief description is included here.

1. The sequences were acquired by Novogene, and they followed all the best practices and provided QC on all data-sets including criteria for trimming adapters and removing reads with excess of N's and adapter sequence (See supplemental methods).

2. The sequences were then mapped to a reference *drosophila* genome given by the version

ftp://ftp.flybase.net/genomes/Drosophila_melanogaster/dmel_r5.57_FB2014_03/fasta/ using BWA-Mem

3. We are quite familiar with GATK and have worked extensively on large human data-sets. See for example, Iranmehr, EJHG 2018 (https://www.nature.com/articles/s41431-018-0270-8), and we used portions of the GATK pipeline. However, some of the best practices ***do not apply*** in our case because we are using pooled data from multiple organisms. Thus, while in GATK it is desirable to have equal contribution from two alleles for a heterozygote, in our case, the allele frequencies would match the population frequency of the alleles. A frequency of 0.1 for an allele would be flagged by GATK but would be perfectly OK for us. Therefore, we used GATK to identify the bi-allelic variant locations and used the raw-read counts to estimate allele frequencies in the pooled-seq data, while also eliminating spurious variants (See supplemental data).

4. Finally, it is important to realize that unlike GWAS and other genetic studies, studies involving tests of selection do not make conclusions based on single SNPs, but on aggregating the effect of 10^2-10^4 SNPs in a genomic region. The miscalling of an individual variant has very little impact on the procedure. Thus having a simpler variant calling protocol is quite reasonable for us. In fact, much of the best practices for GATK are related to the special cases of correctly identifying a small number (<5%) of variants, and most methods would work well on the remaining variants. We also note that post-selection, most of our interesting variants are close to fixation, and therefore are supported by hundreds of reads if not thousands.

5. The sequence data is available at https://www.ncbi.nlm.nih.gov/bioproject/PRJNA657615/.

Equally concerning is the inappropriate self-citing of the authors throughout the manuscript (which, with more than 10 of the 30 cited articles, is rather appalling). The authors appear largely unaware of the relevant literature, leading to i) outrageous claims (e.g., "We made the first direct observation of beneficial alleles recombining to form a highly beneficial haplotype.") as well as ii) a deeply rooted misunderstanding of previous population genetic work (e.g., "this result provides one of the only confirmed observations of a recombination event overcoming the Fisher-Muller ratchet" when actually referring to the Hill-Robertson effect). Consequently, several of the (limited) external citations provided are inappropriate (e.g., the observation that "multiple favored variants can also be acquired on a single haplotype via recombination that prevents clonal interference and accelerates adaptation" has been made by Hill and Robertson in 1966 not by the Desai lab in 2016).

Response. It was an oversight on our part not to cite much of the relevant literature, in the original version of the manuscript. The wording of some of our claims could also sound misleading. We certainly did not mean to imply that McDonald et al in 2016 discovered a new mechanism of how recombination increases the efficiency of selection. We thank the reviewer for pointing out all these shortcomings, which we have now corrected. Specifically, we now cite what we believe is a representative sample of the relevant literature (both theoretical and experimental). We also refined our statements throughout the text regarding the novelty of our work. One of the most striking patterns that we observe is shown in Figure 3d, where the time-series data reveal that in fact two beneficial alleles *in close genetic proximity* that arose in different individuals recombined to form a new highly beneficial haplotype. Indeed, independent fixations of beneficial alleles at apparently large genetic distances have been observed before (e.g., by McDonald et al). The interesting aspect of our finding is that even such apparently rare events can be important drivers of specific selective sweeps.

W.r.t the reviewer's specific concerns, we note that Hill and Robertson (cited now) essentially performed a computational validation of Fisher/Muller observation (see https://www.nature.com/articles/6801059). While other authors have explained the potential benefits of recombinations in sexual populations, few experimental evolution

experiments have been carried out in sexually reproducing organisms until very recently. An even smaller number have had the intermediate generations sequenced and we have cited a sampling of the relevant literature, putting our work in context. We would respectfully like to stand by our claim that we are, to our knowledge, the first to observe a recombination event that overcomes the Muller ratchet in action. Note ***that we are not claiming that we are the first to discover the principle***, simply that we are the first to observe this in an experiment. This leaves open the question of what we should call a recombination event that overcomes the accumulation of deleterious mutations. We call these FM recombinations. We thank the reviewer for pointing out the imbalance of citations, and have added 26 other references that better place our work in context. We had to keep a number of our own references as we were responding to the reviewers.

Revised text:

"There has been much debate about the benefits of sexual reproduction despite its obvious costs. Fisher and Muller postulated that in the absence of recombination, deleterious mutations will accumulate by arising on haplotypes carrying a favored mutation, the so-called Muller's ratchet[3]. Hill and Robertson (1966) used two locus computer simulations to confirm Fisher's predictions and show that "selection at one beneficial locus interferes with selection at a second beneficial mutation reducing its probability of fixation. That is, in natural populations (with finite size), selection at more than one site should cause an overall reduction in the effectiveness of selection" [4,5]. Recombination can overcome the Muller ratchet, thereby explaining the benefit of sexual reproduction[6,7].

Multiple experiments on yeast by Gray and Goddard and colleagues have revealed that sex increases the efficacy of natural selection, unlinks beneficial from deleterious mutations and allows yeast to adapt to specific evolutionary niches[8-10]. McDonald et al. found that sex alters the spectrum of mutations that are fixed in yeast and reduces clonal interference to speed up adaptation[2]. More recently, Leu and colleagues studied the dynamics of adaptation in sexual and asexual yeast populations

subjected to extreme temperature over 1400 generations[11]. They found that both sexual and asexual adaptation occurred at similar rates, but showed significant differences between the two. Notably, these previous studies did not directly investigate the molecular mechanisms used for specific selective sweeps. Addressing that question would require time-series sequencing of intermediate generations, and recent efforts aim to do exactly that to better elucidate the mechanisms underlying selection even for complex organisms with longer generation times[12-15].

In this work, we have directly addressed the question of mechanisms underlying genomic adaptation using an experimental model of *Drosophila melanogaster*."

Once these issues have been addressed, it would be interesting to place the work in a broader context. For example, how do their findings fit in with previous work (e.g., Van Voorhies (2009) research investigating the "metabolic function in Drosophila melanogaster in response to hypoxia and pure oxygen")?

Response. We note that our analysis considered the genetic response to extreme oxygen adaptation, and not the metabolic/physiological response, even though the two are connected. In this regard, however, we have revised the discussion to include Voorhees and a few other papers as follows:

"In spite of a voluminous literature on cellular protection against low oxygen supply[34] or high oxidant burden in various tissues[35,36], there have not been major advances for therapeutic interventions to preserve cells, especially in sensitive organs, such as the heart and brain[36,37]. One discovery of the past few decades (at both organismal and molecular levels) that had a potential for therapy was the decrease in metabolism during hypoxia, a response that attempts to minimize the mismatch between O_2 supply and demand[38-41]. This discovery, however, did not materialize into a real effective therapy, as the clinical trials of brain cooling, for example, to lower brain metabolism in patients suffering from brain hypoxia or ischemia, were largely inconclusive[42]."

Lastly, as a side note, the title "A decade on Everest" is misleading – neither have the experiments been carried out at Everest nor have the populations been sampled there.

Response. We accept this critique and have changed the title to

"Multiple mechanisms drive genomic adaptation to extreme O_2 levels in Drosophila melanogaster"

Reviewer #2 (Remarks to the Author):

Iranmehr et al report an analysis of an amazing evolution experiment: 9 populations propagated in atmosphere controlled environments for 15 years. There are three treatments 3x reps evolving with low O2, 3x reps under high O2 and 3x control populations under normal O2. They carry out whole genome sequencing of many individuals from each of the nine populations at multiple time points. They have found strong evidence for both hard and soft sweeps in parallel populations in early generations. They have also found the late arrival of favourable de novo sweeps in parallel populations. Finally, the authors report the first direct observation of a recombination event that brings beneficial alleles together in a single haplotype in a multicellular organism. Furthermore, the authors have developed clever statistical methods to examine genomic data collected at generational time scales.

I think that this is a neat experimental study carried out on a large scale, and presents novel findings that will be of interest to all evolutionary biologists. In saying this, I found it difficult to follow in some places and interpreting some of the figures in order to assess the authors claims was challenging.

Response. We thank the reviewer for a careful reading of the manuscript and have addressed their critique below.

1. *The title and claims need to be revised to suit the experiments that were carried out. I don't mind a clever title, but it is currently misleading- the title suggests a study of*

natural populations adapting to high altitudes and low oxygen (on Mt Everest), where this was a lab study carried out for 15 years in high and low oxygen concentrations.

Response. We have changed the title to “Multiple mechanisms drive genetic adaptation to extreme O_2 levels in Drosophila melanogaster”

Also, the authors claim to be the first to observe beneficial alleles recombining into a new haplotype, which has already been done for a diploid sexual organism PMID: 32042122. You will need to refine your claims to better suit what you have done, ie this study is probably the the first to do this in Drosophilia or perhaps any metazoan system.

Response. Following this comment and also a comment of Reviewer #1, we have substantially expanded our reference list including this new paper. We also refined some of our claims, as suggested by both reviewers. Please see more details in the response to Reviewer #1 (p. 6 of this response letter).

Revised text:

“There has been much debate about the benefits of sexual reproduction despite its obvious costs. Fisher and Muller postulated that in the absence of recombination, deleterious mutations will accumulate by arising on haplotypes carrying a favored mutation, the so-called Muller’s ratchet[3]. Hill and Robertson (1966) used two locus computer simulations to confirm Fisher’s predictions and show that “selection at one beneficial locus interferes with selection at a second beneficial mutation reducing its probability of fixation. That is, in natural populations (with finite size), selection at more than one site should cause an overall reduction in the effectiveness of selection” [4,5]. Recombination can overcome the Muller ratchet, thereby explaining the benefit of sexual reproduction[6,7].

Multiple experiments on yeast by Gray and Goddard and colleagues have revealed that sex increases the efficacy of natural selection, unlinks beneficial from deleterious mutations and allows yeast to adapt to specific evolutionary niches[8-10].

McDonald et al. found that sex alters the spectrum of mutations that are fixed in yeast and reduces clonal interference to speed up adaptation[2]. More recently, Leu and colleagues studied the dynamics of adaptation in sexual and asexual yeast populations subjected to extreme temperature over 1400 generations[11]. They found that both sexual and asexual adaptation occurred at similar rates, but showed significant differences between the two. Notably, these previous studies did not directly investigate the molecular mechanisms used for specific selective sweeps. Addressing that question would require time-series sequencing of intermediate generations, and recent efforts aim to do exactly that to better elucidate the mechanisms underlying selection even for complex organisms with longer generation times[12-15].

In this work, we have directly addressed the question of mechanisms underlying genomic adaptation using an experimental model of *Drosophila melanogaster*."

2.The authors give two reasons for late sweeps- that either recombination or de novo mutations create new haplotypes. However, since the evolution experiment including incremental changes in oxygen concentration it is possible that an extant allele could only be selected once a certain threshold oxygen concentration is crossed during the experiment. This should also be included as a possibility.

Response. We considered this intriguing possibility and have added a note in the discussion as follows:

"The presence of late, replicated sweeps in our data present an interesting and unexpected result. Replication in the three cohorts suggest a favored mutation that was present at the onset of selection, but, surprisingly, did not confer a beneficial advantage until many generations later. We cannot rule out the possibility that changing O_2 concentration had a potential effect and the benefit of an extant variation was realized only upon reaching that concentration or potential threshold. However, we note that O_2 levels in our experiments were fixed after generation 15 for High O_2, and generation 31 for Low O_2, while the designated 'late' sweeps started after generation 60. Therefore,

we conjecture that non-replicated late sweeps are best explained by a *de novo* mutation or recombination while replicated late sweeps suggest that the benefit of an extant mutation manifested only after a previous sweep was completed."

3. The figure cited (figure s6) is difficult to reconcile with the claims about the H sweeps. In Fig s6 it is not clear to me that sweeps have happened in parallel, for example HD and HA are only evident in two of the panels. Also, these figures are difficult to interpret- Fig s5 and s6 do not have a label on the y axis and figure legend is only one sentence- please explain the axes and expand the legend. In contrast, in figures s7-s15 the allele frequency time course panels show parallel and synchronicity very clearly, but are not cited as demonstrating parallelism, perhaps it would be good to point the reader to these figures to make the point about sweeps, as well as synchronicity.

Response. We apologize if the picture was unclear. First, we want to reiterate using Supplementary Fig. 5 that the input to the procedure is 3 dimensional: allele frequencies over a time interval and within a genomic region.

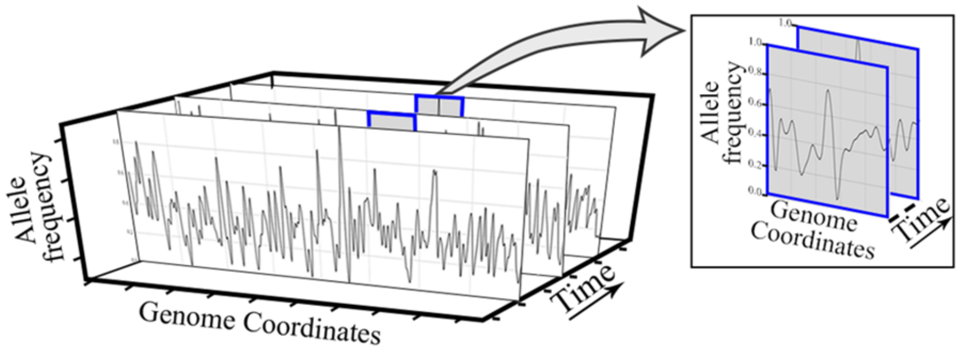

In Supplementary Fig. 6 (now Supplementary Fig. 7, included below), the three panels are independent. They correspond to the entire genome, but cover different time intervals, and the y-axis gives the CLEAR statistic for that time interval. Thus the top panel corresponds to time interval 1-114, middle panel to interval 31-162, and so on. For H_C, the signal is seen in 31-162, but not in the other time intervals. However, the signal is indeed replicated in the three populations, and that time-series data for the

region H_D can be found in Supplementary Fig. 18. We have redrawn the figure by adding the legend CLEAR statistics on the left, and "Time interval" as the heading for each panel (as shown below).

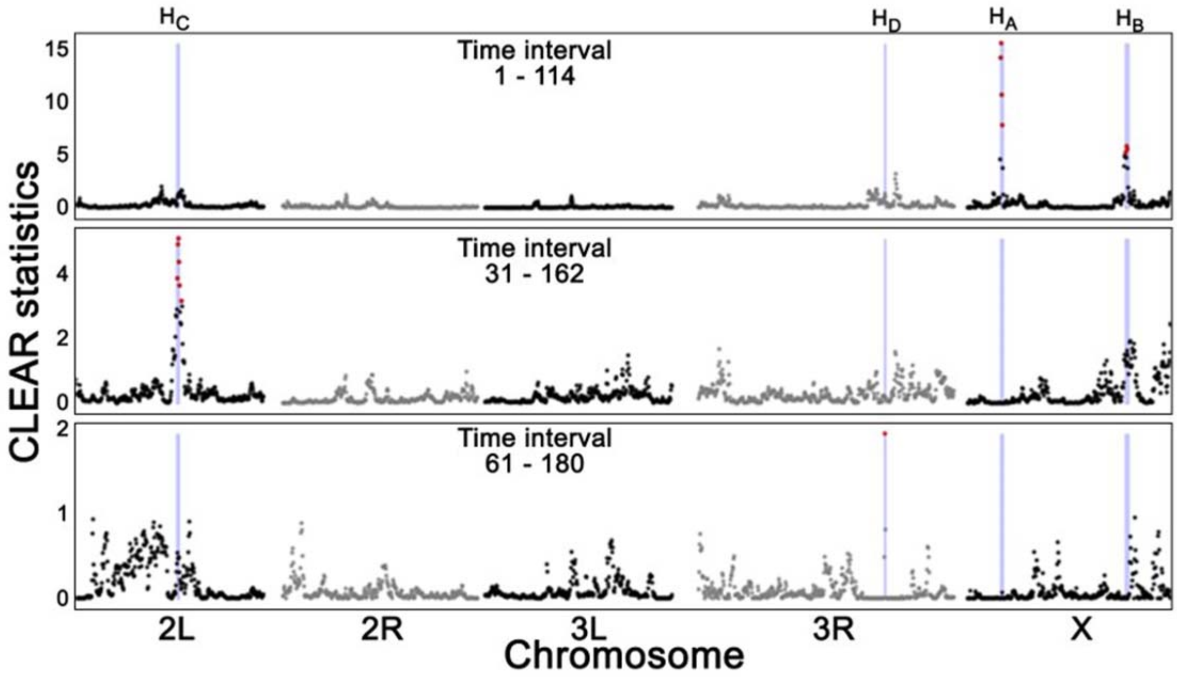

Supplementary Fig. 7. H-Population Replicated Sweeps. The three panels show 3 time intervals and the average of CLEAR statistics (y-axis of each panel) from the three replicates, with peaks corresponding to replicated sweeps in the respective time intervals. Four replicated sweeps i.e., H_A-H_D, were identified in the H-population. Additional details for individual sweeps depicting their allele frequency trajectories are provided in Supplementary Fig. 15-18.

Minor points

Main text first paragraph- standing genetic variation is not necessarily drifting, but could be under weak selection or maintained by balancing selection – better to just remove this.

Page 3: "…similar number of embryos…" What were the actual numbers?

Response. Each population was started with 2000-3000 embryos of the parental cross.

Revised text:

"Nine offspring populations (the F1 generation), containing similar numbers of embryos (2000-3000 embryos), were collected and allowed to evolve independently in the culture chambers supplied with gradually decreasing O_2 levels (L-populations, n=3) or increasing O_2 levels (H-populations, n=3). And three populations were maintained under normal O_2[17-19]"

Page 4: Why did the authors choose to sequence L-fly, H-fly and N-fly populations at different generations?

Response. This is a good question. This was indeed a challenge as we were designing our experiments. We have added the following to the supplemental methods section.

"In determining the time points for sampling, we were guided by some general principles:

1. Increased number of samples in the early part of the cycle (first 100 generations) as selection for such a strong signal would start early and the sweep time would be small based on extensive simulations (Supplementary Fig. 32 which shows the time span of selective sweep (red) versus drift (blue) for multiple values of selection coefficients and starting allele frequencies ν of the favored allele. Most of the fixation happens on very early regimen).

2. Sample the L-populations at some later times relative to H-populations, because it took longer for populations to adapt and stabilize in low-oxygen environments (retrospectively shown in Fig. 1c)

3. Availability of high quality samples at a specific generation.

Based on these considerations, we acquired and sequenced the following samples: N-Population: [4, 17, 180]; H-Population: [1, 7, 12, 31, 61, 114, 162,

180]; and L -Population: [4, 17, 34, 59, 91, 117, 149, 180], each of which had three laboratory replicates. This constitutes a dataset of 56 samples associated with all 3 replicates of L, H and L populations at different generations."

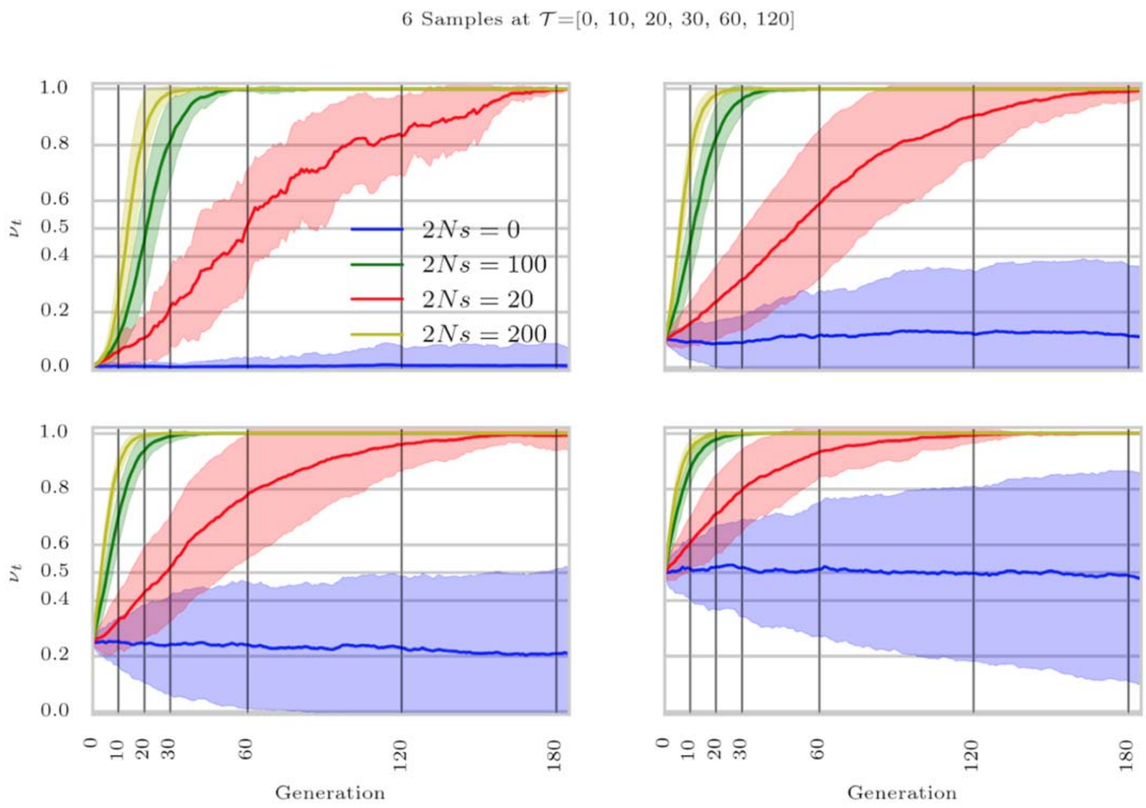

Supplementary Fig. 32. Trajectories (*N*=100) were simulated to capture the time interval of fixation for multiple scenarios including genetic drift (2*Ns*=0), weak selection (2*Ns* = 20), moderate selection (2*Ns* = 100) and strong selection (2*Ns* = 200). Solid line and shaded area represent the mean and 95% confidence intervals based on 100 simulations. Six sampling times were chosen so that dynamics of selection could be capture for multiple initial allele frequencies of 0.01 (A), 0.1 (B), 0.25 (C), 0.5 (D)

Page 5: "…PCA was performed using only extant SNPs…" Please include this information in Figure 1D caption. Have the authors also performed PCA analysis using the whole genetic variation including de novo SNPs? If so do the de novo SNPs change the results?.

Response. We did what the reviewer suggested, and as expected, the results look nearly identical because the number of *de novo* mutations is small relative to extant mutations. We included Supplementary Fig. 1b (PCA analysis using all (*de novo* and extant) SNPs), also provided below.

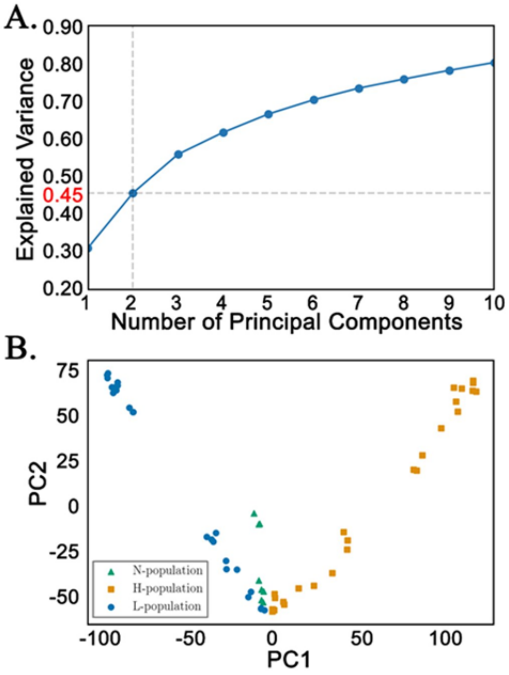

Supplementary Fig. 1. Principal component analysis and the percentage of explained variance. (A) Plot depicting the percentage of explained variance by different numbers of principal components. The percentage of explained variance by PC1+PC2 is 0.454. (B) PCA analysis using all (*de novo* and extant) SNPs.

Page 9: "Additionally, knocking down cic gene…" How was this gene deletion done? Please include a section in the methods that points to a reference and briefly describes how it was done.

Response. We used RNAi lines to perform the deletion. We have added these details in the section entitled "*Drosophila* lines and culture for functional validation" of the supplemental methods section, where this is described in detail, and in the main paper,

we have this text: "...knocking down the *cic* gene, using RNAi lines, led to a higher eclosion rate at 5% O_2 in flies "

Page 9: "…bnl… …with its expression upregulated > 2-fold in flies when exposed to 5% O2" How did the authors measure this finding? RNAseq? Once again, please include a section explaining this in the methods.

Response. The expression levels were measured using the Affymetrix microarray platform and was previously reported in Azad et. al. 2009 PLOS One. We have now added this detail in the main manuscript as,

"its expression measured using Affymetrix microarray was upregulated > 2-fold in flies when exposed to 5% O_2"

Page 10: The first two sentences of the discussion, about medical cell preservation techniques, which seems a non-sequitor, I would recommend deleting this. Also "accidental origin" Do you mean non-selected or neural? Please rewrite.

Response. We removed the phrase, "...and simultaneously rules out the probability of accidental origin" from the statement. Now it reads as,

"The fact that these selected intervals are present only in one type of environment (i.e., either only in L-population or in H-population) plausibly indicates its environment-specific functional significance."

Page 19: Fig 2E, please indicate what the blue and light brown colors refer to. Also, Figure 2 legend: fixed, not fixated

Response. We have clarified Figure 2e by rewriting the caption for the same figure as follows:

“**Fig. 2e:** Simulation of an ‘FM recombination’ that overcomes the Fisher-Mueller ratchet by combining beneficial mutations onto a single haplotype. Starting at generation 80 from a cluster (haplotype) of fixed alleles, and tracing back in time, ESAP identifies two distinct clusters/haplotypes colored blue and orange (top panel). The blue and orange circles in the middle and lower panels provide the allele frequencies and genomic location of the mutations in these haplotypes at generation 1 (middle panel), and generation 80 (lower panel). Note the perfect separation of the two flanking haplotypes around chrX:7,750,000 i.e., the recombination locus.”

Pages 25-26: “Embryos from this parental population were collected as F1 and subjected to experimental evolution in low or high oxygen environments (oxygen-directed evolutions, 3 populations per condition), or under room air (control experiment for genetic drift, 3

populations)” How many of these F1 embryos were used as parental populations? What were the starting population sizes? Did they vary in size?

Response. The parental population was the balanced pool of 27 isofemale lines containing 20 males and 20 virgin females from each line (total 540 males and 540 virgin females). The F1 embryos of this parental cross were used to start the populations with n between 2000 - 3000 per population for all three types of populations. The population sizes were similar among the populations at the beginning of the experiment. For the population size we have included the following statement at the beginning of the method section.

“A total of 27 isofemale DMN (*Drosophila menalogaster* Netherlands) lines descended from individual *Drosophila melanogaster* females caught at fruit baits at a single location in Leiden, Netherlands (52□ 01' N 4□ 29' E) during October 1999 (kindly provided by Dr. Andrew Davis) were used to create a single laboratory cage population (founding population) with 20 males and 20 virgin females from each line (1,080 ies in total).”

The information related to the number of embryos is stated as, “Embryos from this parental population were collected as F1 and subjected to experimental evolution in low or high oxygen (O_2) environments (oxygen-directed evolutions, 3 populations per condition), or under room air (control experiment for genetic drift, 3 populations). This constituted 9 offspring populations (the F1 generation), containing 2000-3000 embryos, that were collected and allowed to evolve independently in the culture chambers supplied with gradually decreasing O_2 levels (L-populations, n=3) or increasing O_2 levels (H-populations, n=3). And three populations were maintained under normal O_2 (normoxic) condition as controls (N-populations, n= 3).”

We have also mentioned the number of embryos in the main text as, “Nine offspring populations (the F1 generation), containing similar numbers of embryos (2000-3000 embryos), ..” in paragraph four of the introduction section.

Page 26: “Embryos, 3rd instar larvae and adult flies were collected from each generation and stored at 80C for subsequent analyses” How many embryos, 3rd instar larvae and adult flies were frozen at -80 degree freezer? Also change “80C” to -80°C.

Response. We have now included this information in the Methods section under ‘Oxygen-directed experimental evolution of *Drosophila melanogaster*’. It now reads as, “Briefly, 200 to 300 embryos, 100 wandering 3rd instar larvae and all adult flies (2000 to 3000) per population were collected at each generation. The adult samples were collected at the end of each generation after they laid eggs to start the next generation. These numbers of sampling did not apply to the bottleneck and some generations right after. The number of adult flies in a population at the end of each generation was used to estimate the physical size of the L-, H- and N-populations.” Thank you for pointing out the error relating to the temperature. We have corrected it accordingly.

Page 65-page 880 of the document I reviewed was a pdf list of genes. In the final version this should be supplied as a spreadsheet supplementary data file since it is not convenient to review or use in its current form.

Response. In the revision, we have uploaded the supplementary tables as a single excel file/spreadsheet where each supplementary table is provided on a separate excel sheet.

Reviewer #3 (Remarks to the Author):

This is without doubt an excellent manuscript that I believe can be published as it is (I have very minor comments). The authors have managed to correctly apply complex statistical analyses for the identification of selective sweeps to their experimental system. I am very pleased by the attention the authors have dedicated to disentangling the effects of genetic drift and positive selection. I would like to congratulate the authors for this exciting study.

Response. We thank the reviewer for his/her support.

Minor comments

1 - It would be nice to describe the geographic origin of the 27 lines that were used to found the 9 experimental populations. Were they chosen to maximise genetic diversity? Or to represent extreme natural environments with respect to altitude?

Response. We have added the following to the supplemental section.

"A total of 27 isofemale DMN (*Drosophila menalogaster* Netherlands) lines descended from individual *Drosophila melanogaster* females caught at fruit baits at a single location in Leiden, Netherlands (52° 01' N 4° 29' E) during October 1999 (kindly provided by Dr. Andrew Davis) were used to create a single laboratory cage population (founding

population) with 20 males and 20 virgin females from each line (1,080 flies in total). As the isofemale lines used in our population were caught in the wild, considerable genetic diversity existed in the founding population. Indeed, as previously described, diverse levels of hypoxia tolerance have been found between these DMN lines[1]."

2- the genomic distribution of the main signals of selection seem to indicate a significant effect of the X chromosome. The X chromosome has the particularity to be hemizygous in males in this species which could favor the fixation of recessive beneficial mutations?

Response. We thank the reviewer for this important point. We included a few sentences in the revised discussion as indicated below.

"It is interesting to note that a significant number of the selected intervals (4 out of 5 in the L-populations and 2 out of 4 in the H-populations) are located on the X chromosome, well in excess of its size, which represents 20% of the *Drosophila* genome. Indeed, previous studies have suggested that, due to its hemizygosity in males, selection acts more efficiently on X -chromosome genes than genes located on the autosomes[48-50]. We speculate that for complex adaptations, multiple genes in a pathway can play an adaptive role, and the most efficient path is chosen."

Figure 1D: show percentage of explained variance

Response. It is 0.454, as illustrated in the figure below. This figure is now included as Supplementary Fig 1a, and we have added a line to the main text as follows:

"We found that the populations were well separated by the top two principal components (Fig. 1d), explaining 45% of the total variance (Supplementary Fig. 1a)."

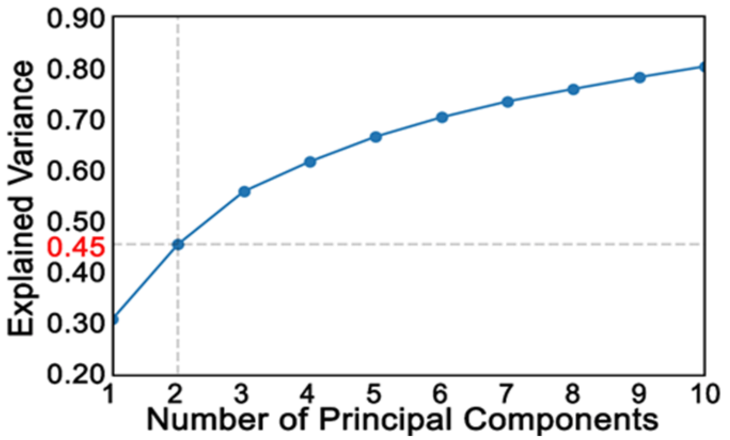

Supplementary Fig. 1. Principal component analysis and the percentage of explained variance. (A) Plot depicting the percentage of explained variance by different numbers of principal components. The percentage of explained variance by PC1+PC2 is 0.454.

Figure S2: what is the dominance coefficient assumed for the selected alleles in Figure S2C?

Response. The dominance coefficient is 0.5. We have added this detail in the figure legend (now Supplementary Fig. 3c) and it now states, "Trajectories of the mutations in the case of genetic drift vs selection sweep in a finite population size. Dominance coefficient = 0.5."

Figure S3 not clear enough. What is the unit of the y-axis of Figure S3B, is this the test statistic of CLEAR?

Response. Y axis represents the 99th percentile of distribution of allele frequencies (after 10, 20, 60, 120 generations) as a function of initial frequency, under genetic drift (Null Hypothesis). This figure is now Supplementary Fig. 4 and has a revised caption as follows:

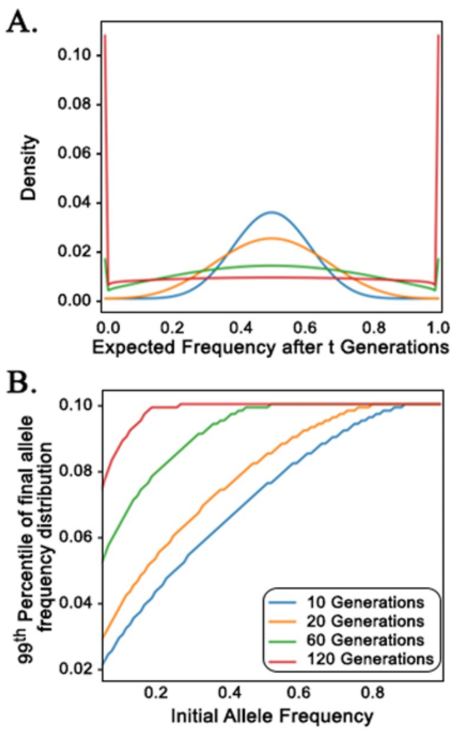

Supplementary Fig. 4. Power of test of selection decreases in longer time-intervals. (A) Expected distribution of genetic drift starting at 0.5 frequency after 10, 20, 60 and 120 generations for Ne=200. (B) Y axis represents the 99th percentile of distribution of allele frequencies (after 10,20,60,120 generations) as a function of initial frequency, under genetic drift (Null Hypothesis) Note that 1% of *de novo* mutations reach a frequency of 0.75 after 120 generations. However, 99% of drifting *de novo* mutations remain below 52% after 60 generations.

Figure 2C: a legend is needed for the color-coding

Response. We have revised the legend of the figure and it now reads as:

“C) The characteristic allele frequency trajectory in simulations of a soft-sweep due to standing variation. The favored mutation itself is fixed along with tightly linked mutations (black lines). However, the favored mutation is carried on multiple carrier haplotypes which drift at intermediate frequencies (orange lines).”

eviewer #1 (Remarks to the Author):

After re-submission, several of my initial critical issues remain:

As pointed out in my initial review, I couldn't disagree any stronger with the author's data interpretation. The authors stick to their claim to be "the first to observe a recombination event that overcomes the Muller ratchet in action". In short, Muller's ratchet refers to a process where, in the absence of recombination, a finite population accumulates deleterious mutations in an irreversible fashion. Given that the authors state in their rebuttal that "two beneficial alleles in close genetic proximity (...) recombined to form a new highly beneficial haplotype", I assume that they would not like to argue for an absence of recombination in Drosophila?! Instead, the authors should be referring to the Hill-Robertson effect - for which there is both theoretical and experimental evidence in the literature.

With regard to the revised methods (i.e., CLEAR with varying population sizes): The authors state that "all significant results in the fixed-population size regime remained significant" but Supplementary Figure 8 highlights sites that were significant under the fixed population size regime and that are no longer significant under the varying population size regime (though the authors decided to display them - somewhat misleadingly - in the same color than the non-significant sites). Unfortunately, Supplementary Figure 35 is not much help in this regard either as it doesn't contain a significance threshold.

Reviewer #2 (Remarks to the Author):

I commend the authors on completing an excellent study. The authors have carried out extensive rewrites, and provided responses, that have addressed all of my concerns.

Minor Comments and Errors

L106 and Fig. 1. Pearson R should be Pearson’s R, and in the figure should be capitalized.

L154 and 162. Subtitles are seemingly randomly used and are not particular informative. The authors might want to consider making them regular and descriptive to make the manuscript more navigable.

L218 “…overcoming Muller’s ratchet.”

L248 and 552 and 207 (that I found) Inconsistent use of “p“ and “p-val” and “p-value” when describing statistics.

Reviewer #3 (Remarks to the Author):

I am satisfied by the answers and modifications provided by the authors.

REVIEWER COMMENTS

Reviewer #1 (Remarks to the Author):

After re-submission, several of my initial critical issues remain:

As pointed out in my initial review, I couldn't disagree any stronger with the author's data interpretation. The authors stick to their claim to be "the first to observe a recombination event that overcomes the Muller ratchet in action". In short, Muller's ratchet refers to a process where, in the absence of recombination, a finite population accumulates deleterious mutations in an irreversible fashion. Given that the authors state in their rebuttal that "two beneficial alleles in close genetic proximity (...) recombined to form a new highly beneficial haplotype", I assume that they would not like to argue for an absence of recombination in Drosophila?! Instead, the authors should be referring to the Hill-Robertson effect - for which there is both theoretical and experimental evidence in the literature.

Response: We have now removed several references to Muller's ratchet throughout the text. We agree that there is theoretical and empirical evidence for the Hill-Robertson effect, and we do not claim otherwise. We have also added the reference provided by the reviewer.

With regard to the revised methods (i.e., CLEAR with varying population sizes): The authors state that "all significant results in the fixed-population size regime remained significant" but Supplementary Figure 8 highlights sites that were significant under the fixed population size regime and that are no longer significant under the varying population size regime (though the authors decided to display them - somewhat misleadingly - in the same color than the non-significant sites). Unfortunately, Supplementary Figure 35 is not much help in this regard either as it doesn't contain a significance threshold.

Response:
We appreciate the reviewer's comment that Supp Figures need clarification. To answer the reviewer's comments regarding Supplementary Fig. 8, we note that each dot in this figure corresponds to a single 50kbp window and multiple such overlapping windows form a region. As the boundaries of the regions predicted by the two methods (using fixed and adjusted population size) do not match exactly, some 50kbp windows that are significant by one method may not be significant in the other, but there is concordance in the broad region. This is explained below.

In Supplementary Figure 35, we have added a threshold line to improve clarity. We note that each of the intervals LA through LE needs to be significant in *some* time windows to be selected using either the fixed population size or the adjusted population size. In fact we do observe complete concordance, even though there are individual differences w.r.t smaller 50kbp windows. We have additionally added a new column to Table 1 showing that every interval treated as significant with the fixed population size is also significant

in the adjusted population size. Also, as the scatter plot and Manhattan plot show the same data, we just replaced the scatter plot with the Manhattan plot from Supplementary Figure 35 (for L-population) and a new Manhattan plot for the H-population, and it is now the new Supplementary Figure 8 (also shown below).

Table 1. Selected intervals that have frequency distribution synchronized in all three biological replicates (replicated sweeps). All intervals picked by the adjusted size regime (Coordinate (Adjusted)) corresponding to intervals selected by the fixed size regime (Coordinate (Fixed)).

**Name**	**Coordinate (Fixed)**	**Length**	**Selection**	**Generations**	**Kind**	**Coordinate (Adjusted)**
L_A	chr3R:15.11-16.47Mb	1360Kb	Low Oxygen	59-91	Hard Sweep	chr3R:15.12-16.48Mb
L_B	chrX:13.01-13.36Mb	355Kb	Low Oxygen	59-91	Hard Sweep	chrX:13.03-13.07Mb
L_C	chrX:16.12-16.57Mb	445Kb	Low Oxygen	4-34	Hard Sweep	chrX:16.27-16.48Mb
L_D	chrX:18.16-19.35Mb	1190Kb	Low Oxygen	4-34	Hard Sweep	chrX:18.17-19.23Mb
L_E	chrX:20.49-21.20Mb	710Kb	Low Oxygen	59-91	Soft Sweep	chrX:20.52-21.13Mb
H_A	chrX:4.51-4.76Mb	248Kb	High Oxygen	1-31	Hard Sweep	chrX:4.52-4.73Mb
H_B	chrX:16.57-16.95Mb	380Kb	High Oxygen	12-61	Hard Sweep	chrX:16.67-17.18Mb
H_C	chr2L:10.35-10.62Mb	273Kb	High Oxygen	31-114	Hard Sweep	chr2L:10.42-10.93Mb
H_D	chr3R:20.66-20.75Mb	90Kb	High Oxygen	12-61	Soft Sweep	chr3R:20.68-20.73Mb

Supplementary Fig. 8. CLEAR statistics Manhattan plot for L-Population (top) and H-Population (bottom) Replicated Sweeps with adjusted population size.

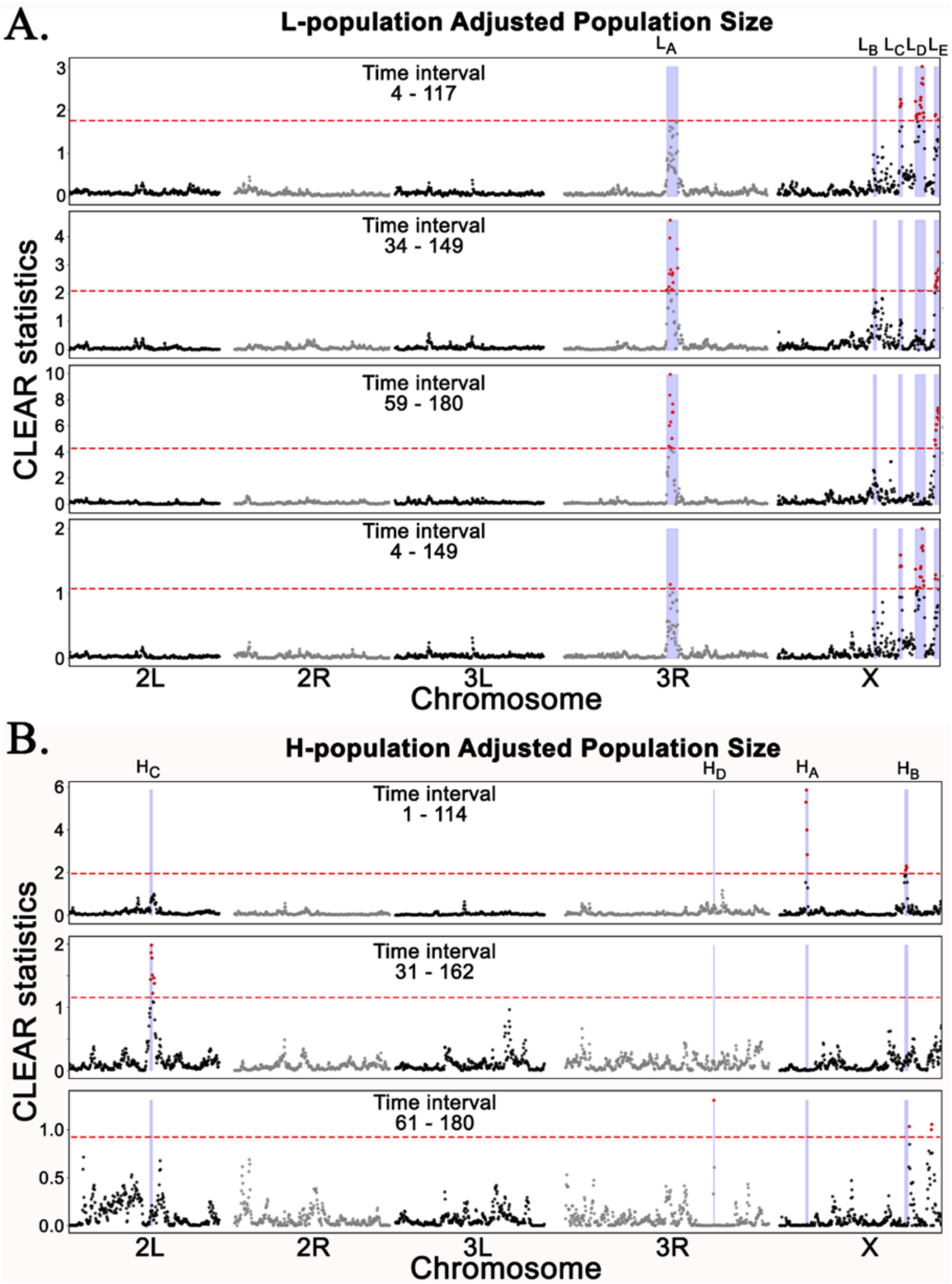

Supplementary Fig. 8. CLEAR statistics for L-Population and H-Population with adjusted population size. The five replicated sweeps for L_A through L_E (A) and four replicated sweeps for H_A through H_D (B) remain significant after adjusting for changes in population size. The cut-offs for both statistics was the 5.5 standard deviation from the mean. All selected regions i.e., above the red dotted line, remained significant in both fixed (as depicted in Supplementary Figure 6 and 7) and adjusted population size regime, due to strength of signal and stringency of cutoffs.

Reviewer #2 (Remarks to the Author):

I commend the authors on completing an excellent study. The authors have carried out extensive rewrites, and provided responses, that have addressed all of my concerns.
Response: Thank you. We truly appreciate it.

Minor Comments and Errors

L106 and Fig. 1. Pearson R should be Pearson's R, and in the figure should be capitalized.
Response: Thank you. We have corrected the errors both in the text and in the figure.

L154 and 162. Subtitles are seemingly randomly used and are not particular informative. The authors might want to consider making them regular and descriptive to make the manuscript more navigable.
Response: The reviewer is correct and for this short paper, we have decided to remove the subtitles.

L218 "…overcoming Muller's ratchet."
Response: As suggested by the other reviewer we have now removed this statement.

L248 and 552 and 207 (that I found) Inconsistent use of "p" and "p-val" and "p-value" when describing statistics.
Response: Thank you. We have changed to "p-value".

Reviewer #3 (Remarks to the Author):

I am satisfied by the answers and modifications provided by the authors.
Response: Thank you.

Reviewer #2 (Remarks to the Author):

I reiterate my earlier praise for this interesting and important experiment.

I have one suggested change. The authors still have a very strong, and incorrect claim to primacy, on page 10:

"To our knowledge, this is the first direct observation of an FM-recombination event."

McDonald et al., 2016 and Leu et al., 2020 both made this observation in their time course sequencing data, can the authors please modify the sentence:

"To our knowledge, this is the first direct observation of an FM-recombination event in a multicellular species."

This corrected claim still very significant and this correction does not diminish the importance of the work.

REVIEWERS' COMMENTS

Reviewer #2 (Remarks to the Author):

I reiterate my earlier praise for this interesting and important experiment.
I have one suggested change. The authors still have a very strong, and incorrect claim to primacy, on page 10:
"To our knowledge, this is the first direct observation of an FM-recombination event."
McDonald et al., 2016 and Leu et al., 2020 both made this observation in their time course sequencing data, can the authors please modify the sentence:
"To our knowledge, this is the first direct observation of an FM-recombination event in a multicellular species."
This corrected claim still very significant and this correction does not diminish the importance of the work.

ANSWER: We thank the reviewer for the suggestion. The sentence is now rephrased as recommended.
